# Supplementary material for: A non-canonical role of the inner kinetochore in regulating sister-chromatid cohesion at centromeres
Source: EMBO J. 2024 May 7;43(12):7. doi: 10.1038/s44318-024-00104-6 (PMC11182772; doi:10.1038/s44318-024-00104-6)
Supplement: Supplementary file 1 — Table EV1 [file 44318_2024_104_MOESM1_ESM.docx]

**Table EV1. X-ray data collection and refinement statistics**

| **SA2-Scc1-CENP-U (PDB 8K4D)** | |
| --- | --- |
| **Data collection** | |
| Space group | *P2_1_2_1_2* |
| Cell dimensions |  |
| a, b, c (Å) | 79.33, 132.82, 148.37 |
| α, β, γ (°) | 90, 90, 90 |
| Resolution (Å) | 50.00-3.55 (3.61-3.55) |
| R _meas_ | 0.14 (1.58) |
| *I/ σI* | 15.21 (1.59) |
| CC _1/2_ | 97.6 (56.3) |
| Completeness (%) | 100.0 (99.9） |
| Redundancy | 6.3 (6.3) |
| **Refinement** |  |
| Resolution (Å) | 49.48-3.55 |
| *R*_work_/*R*_free_ | 0.228/0.262 |
| Unique reflections | 17447 |
| No. atoms |  |
| macromolecules | 8133 |
| Solvent | 0 |
| B factors (mean; Å^2^) | |
| macromolecules | 55.17 |
| R.m.s deviations | |
| Bond lengths (Å) | 0.003 |
| Bond angles (°) | 0.516 |
| Ramachandran favored (%) | 98.07 |
| Ramachandran allowed (%) | 1.93 |
| Ramachandran outliers (%) | 0.00 |

*Statistics for the highest-resolution shell are shown in parentheses.
