# Supplementary material for: A non-canonical role of the inner kinetochore in regulating sister-chromatid cohesion at centromeres
Source: EMBO J. 2024 May 7;43(12):7. doi: 10.1038/s44318-024-00104-6 (PMC11182772; doi:10.1038/s44318-024-00104-6)
Supplement: Supplementary file 3 — Movie EV1 [file 44318_2024_104_MOESM3_ESM.zip › EMBOJ-2023-115677R1_Legend for Movie EV1.docx]

**Figure legend for Movie EV1**.

**Movie EV1 (Related to Fig EV1A and B).** Live imaging of control HeLa cells expressing H2B-GFP, during release from STLC into MG132.
