# Supplementary figures and images for: A non-canonical role of the inner kinetochore in regulating sister-chromatid cohesion at centromeres (part 1 of 3)
Source: EMBO J. 2024 May 7;43(12):7. doi: 10.1038/s44318-024-00104-6 (PMC11182772; doi:10.1038/s44318-024-00104-6)

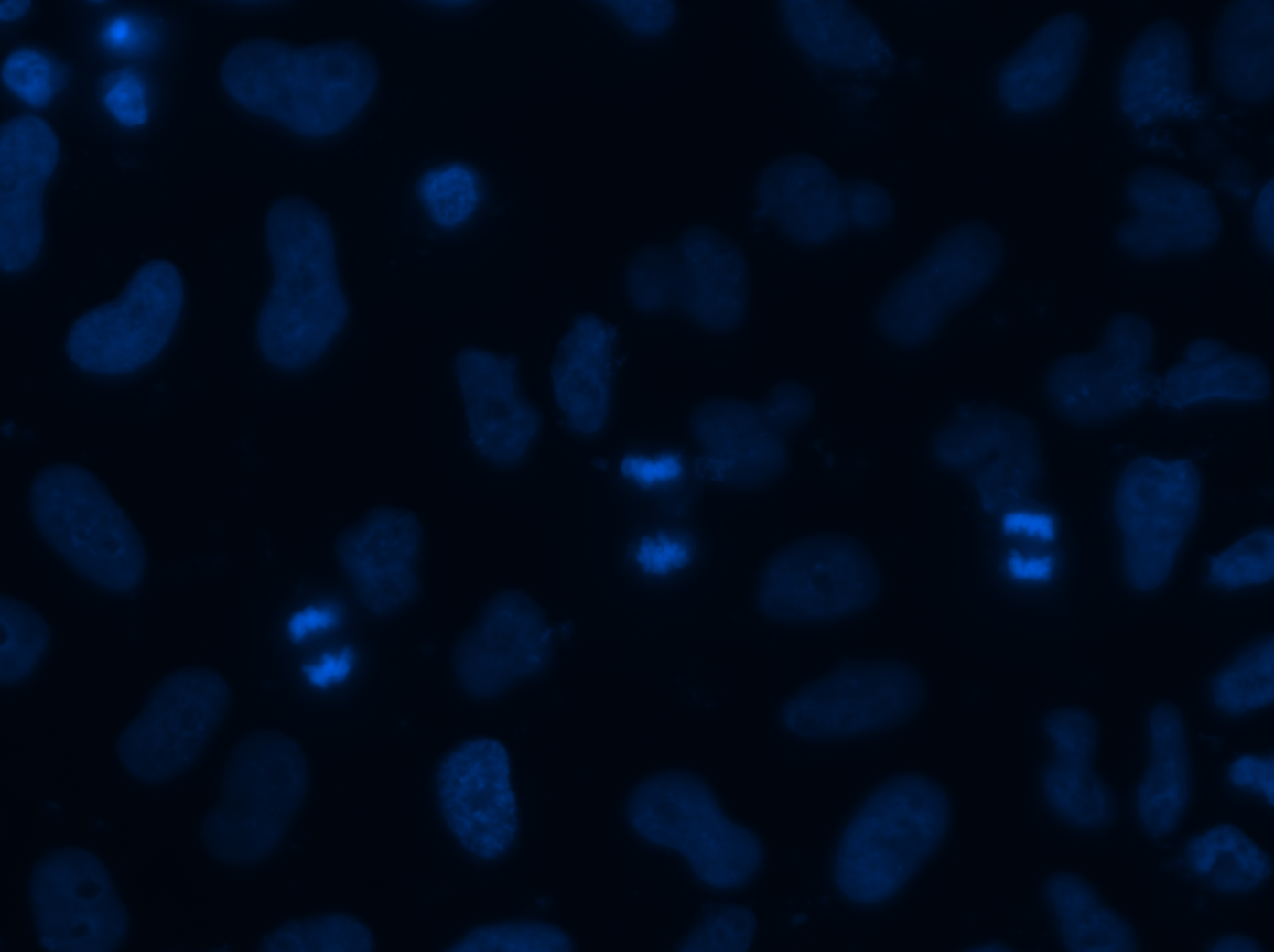

Supplement: Supplementary file 5 — Source data Fig. 1 [file 44318_2024_104_MOESM5_ESM.zip › Figure 1/1C/Anaphase.tif]

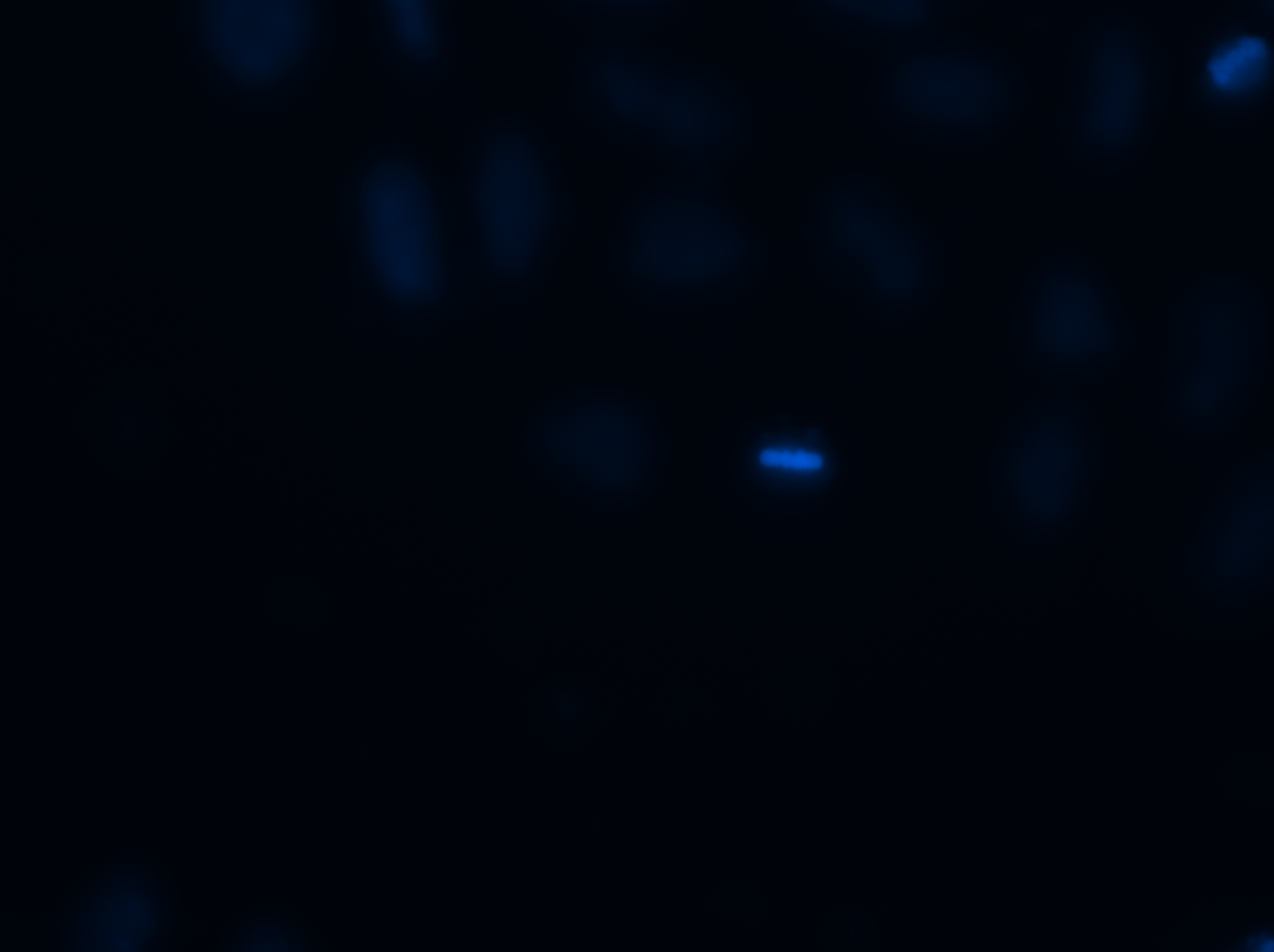

Supplement: Supplementary file 5 — Source data Fig. 1 [file 44318_2024_104_MOESM5_ESM.zip › Figure 1/1C/Metaphase.tif]

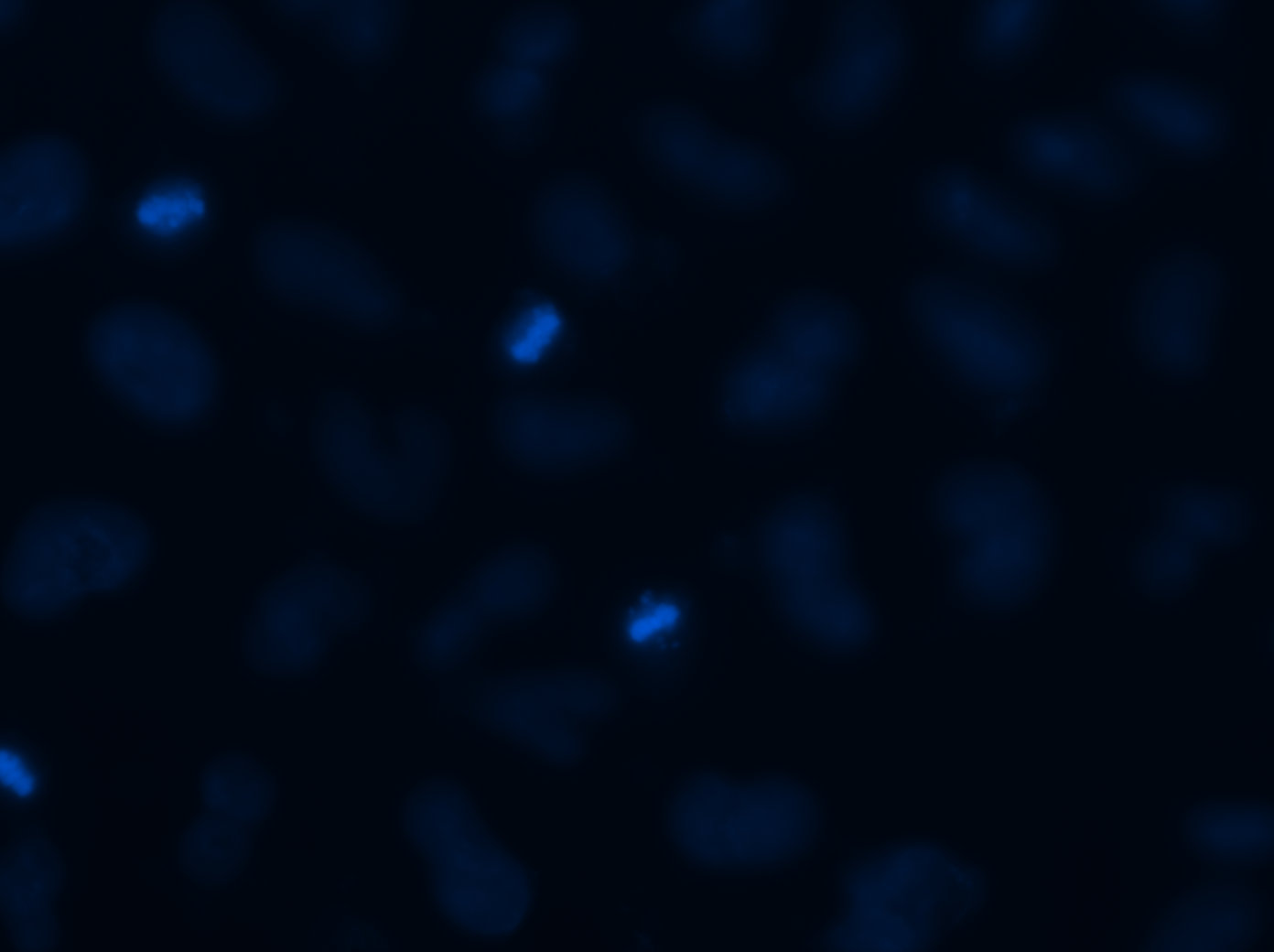

Supplement: Supplementary file 5 — Source data Fig. 1 [file 44318_2024_104_MOESM5_ESM.zip › Figure 1/1C/Misalignment-1.tif]

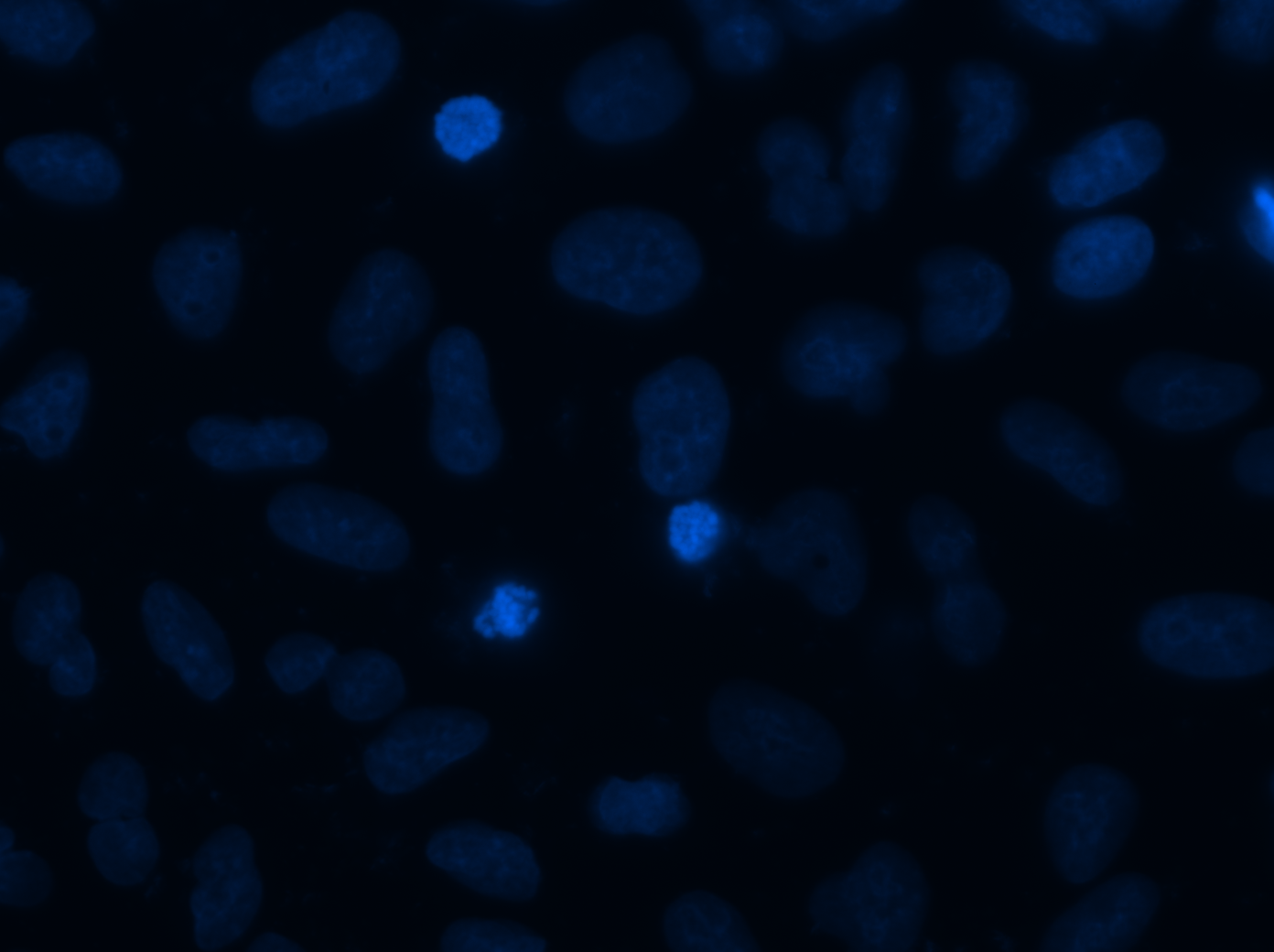

Supplement: Supplementary file 5 — Source data Fig. 1 [file 44318_2024_104_MOESM5_ESM.zip › Figure 1/1C/Misalignment-2.tif]

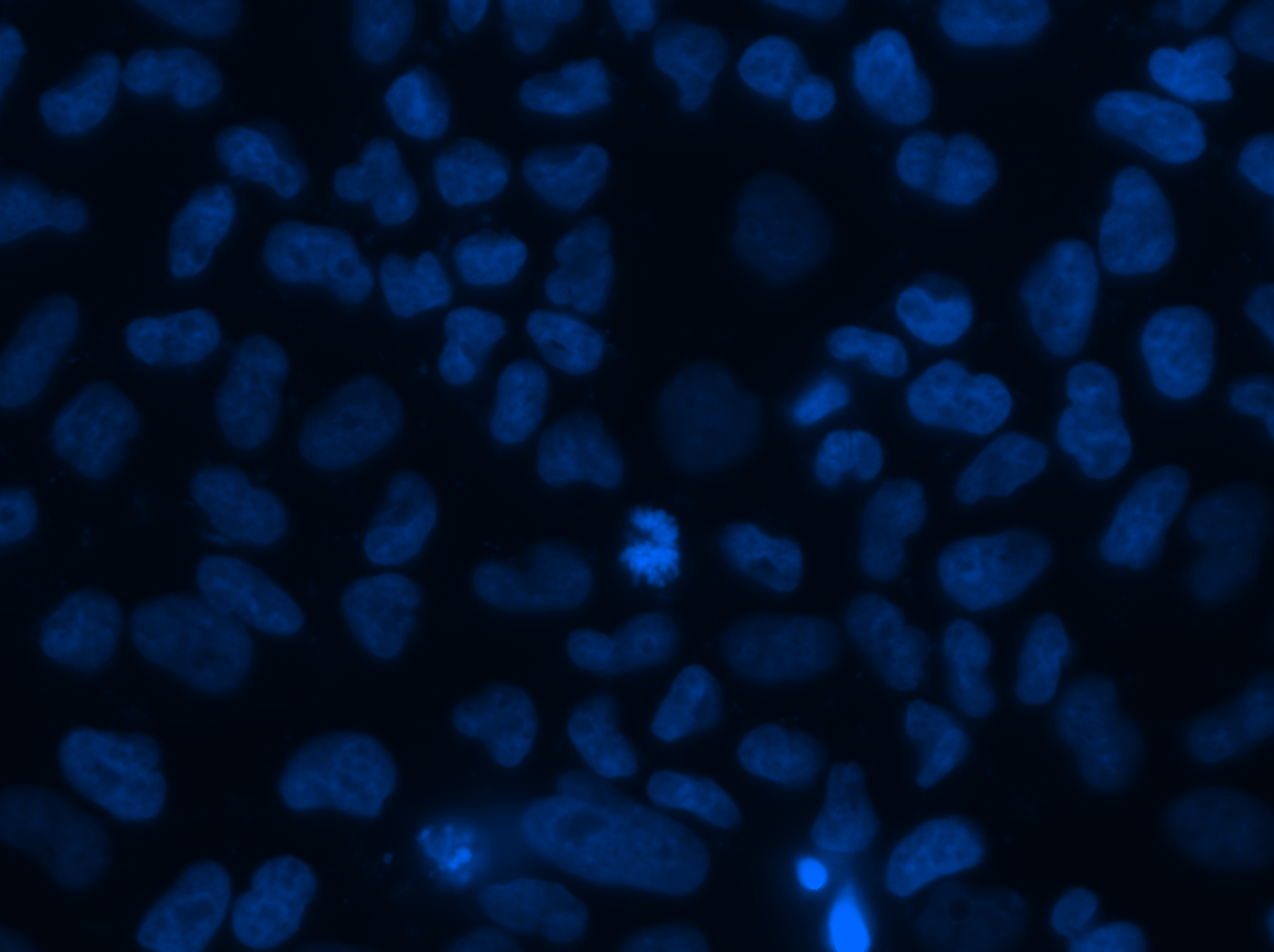

Supplement: Supplementary file 5 — Source data Fig. 1 [file 44318_2024_104_MOESM5_ESM.zip › Figure 1/1C/Prometaphase.tif]

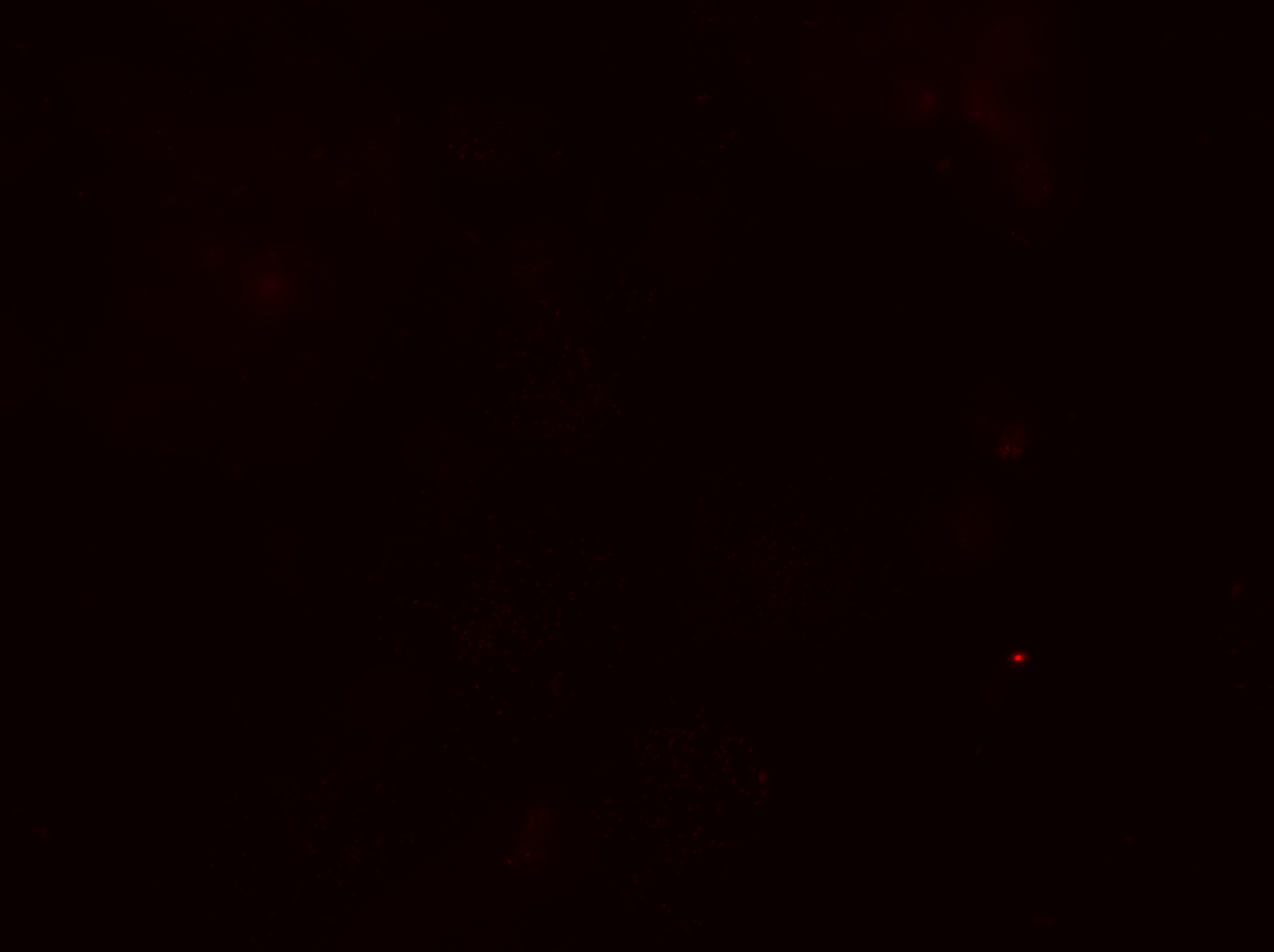

Supplement: Supplementary file 5 — Source data Fig. 1 [file 44318_2024_104_MOESM5_ESM.zip › Figure 1/1E/HeLa siCENP-U#1 completely separated CENP-C.tif]

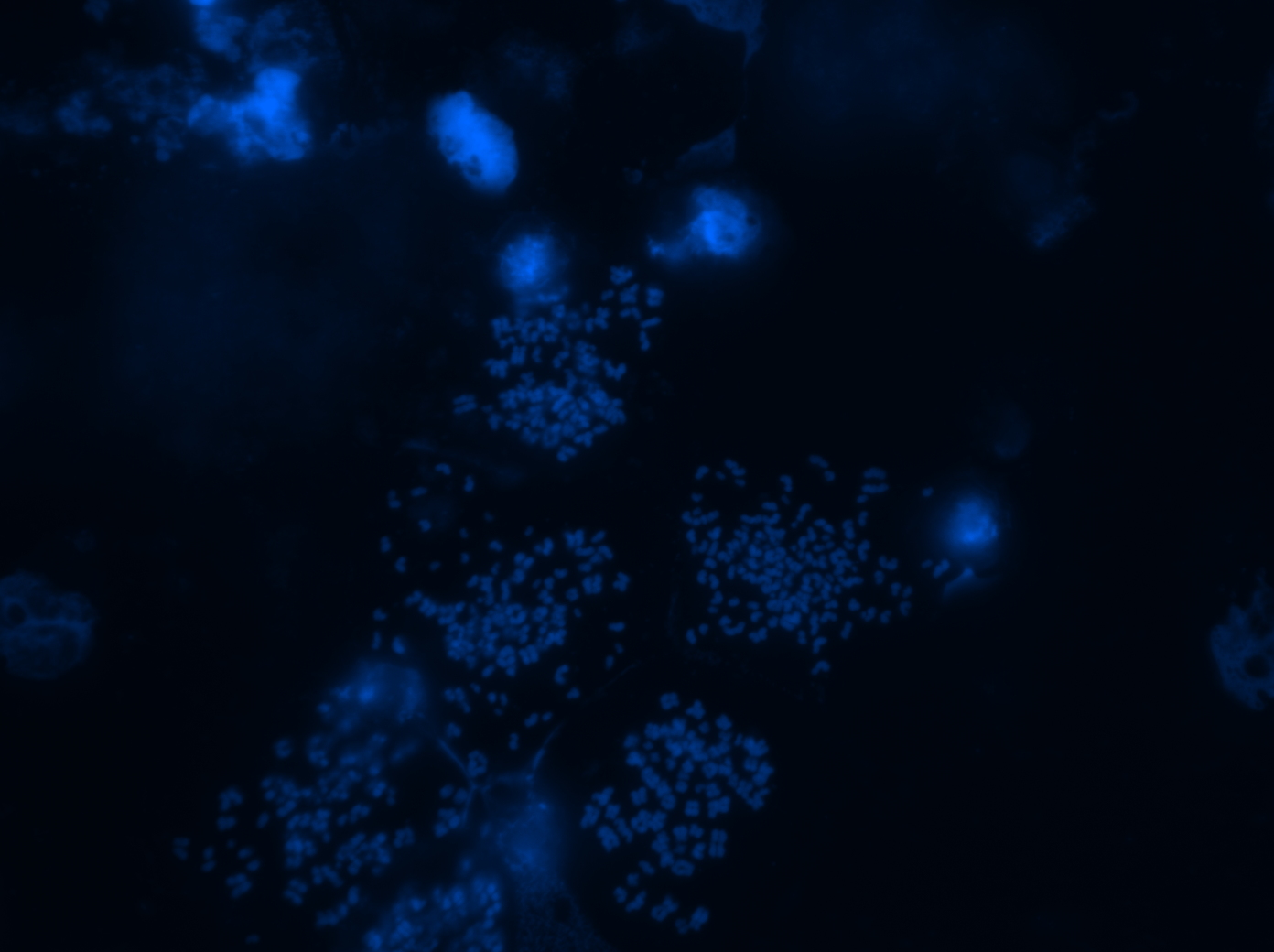

Supplement: Supplementary file 5 — Source data Fig. 1 [file 44318_2024_104_MOESM5_ESM.zip › Figure 1/1E/HeLa siCENP-U#1 completely separated DNA.tif]

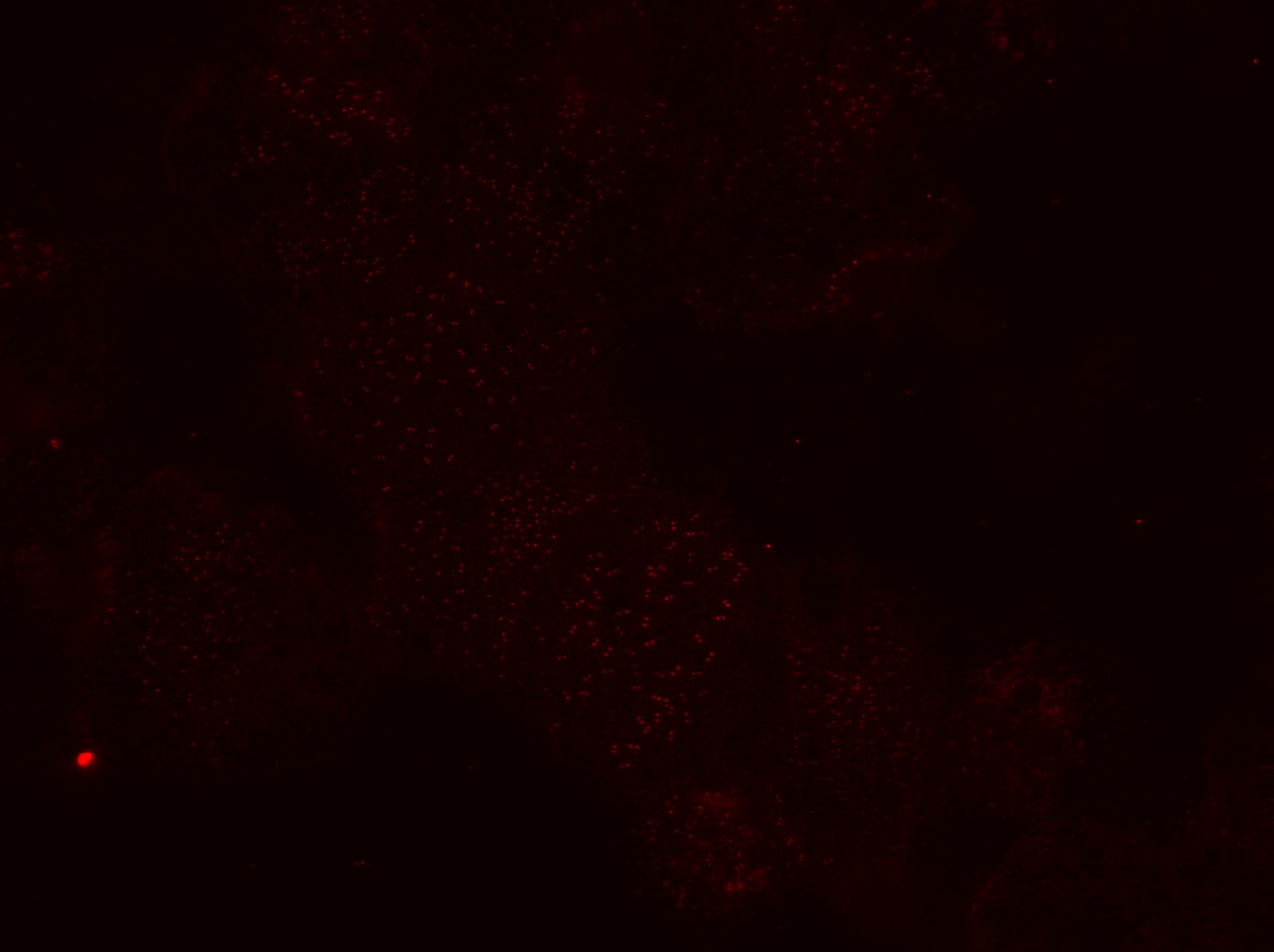

Supplement: Supplementary file 5 — Source data Fig. 1 [file 44318_2024_104_MOESM5_ESM.zip › Figure 1/1E/HeLa siCENP-U#1 partly separated CENP-C.tif]

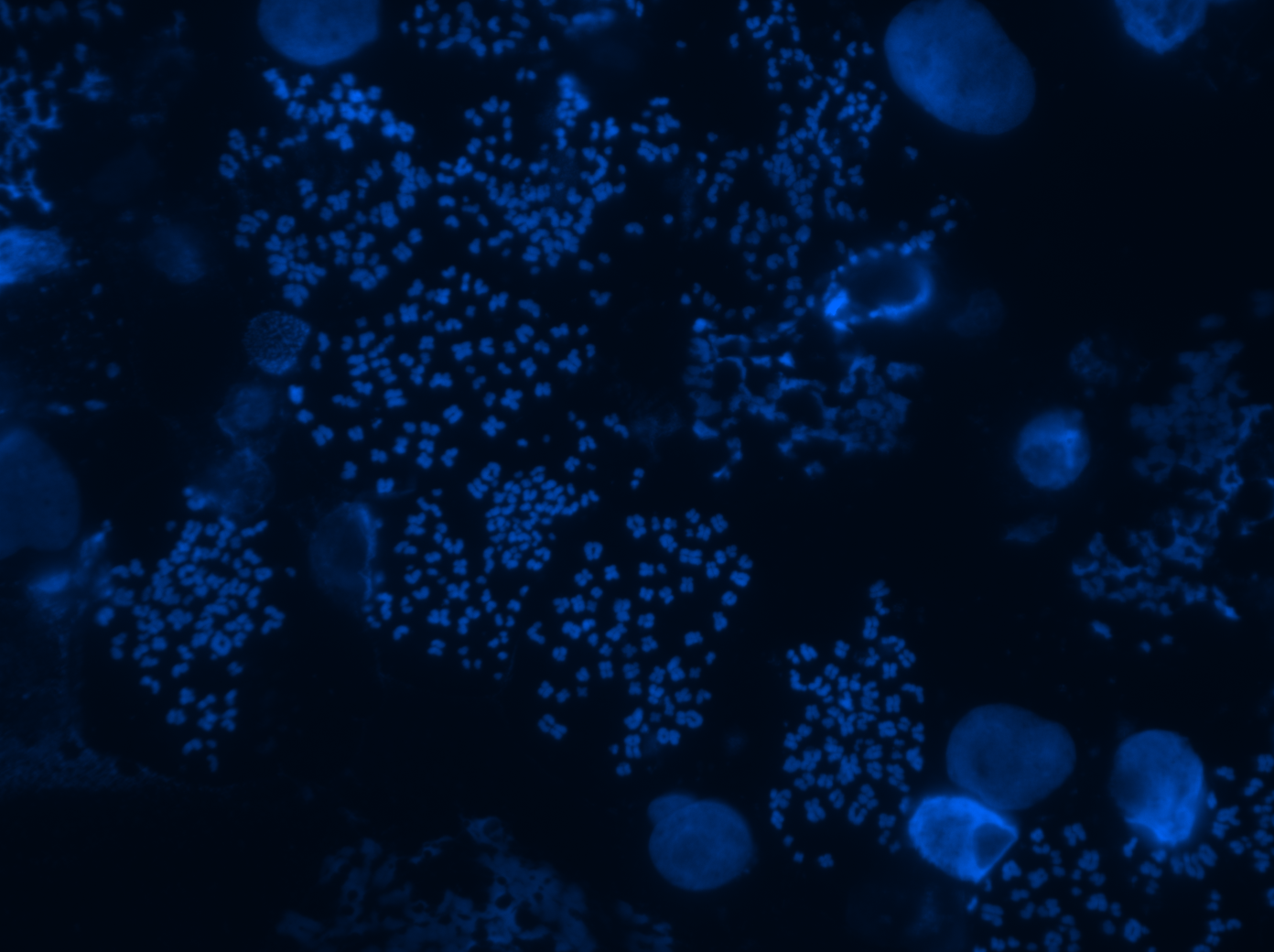

Supplement: Supplementary file 5 — Source data Fig. 1 [file 44318_2024_104_MOESM5_ESM.zip › Figure 1/1E/HeLa siCENP-U#1 partly separated DNA.tif]

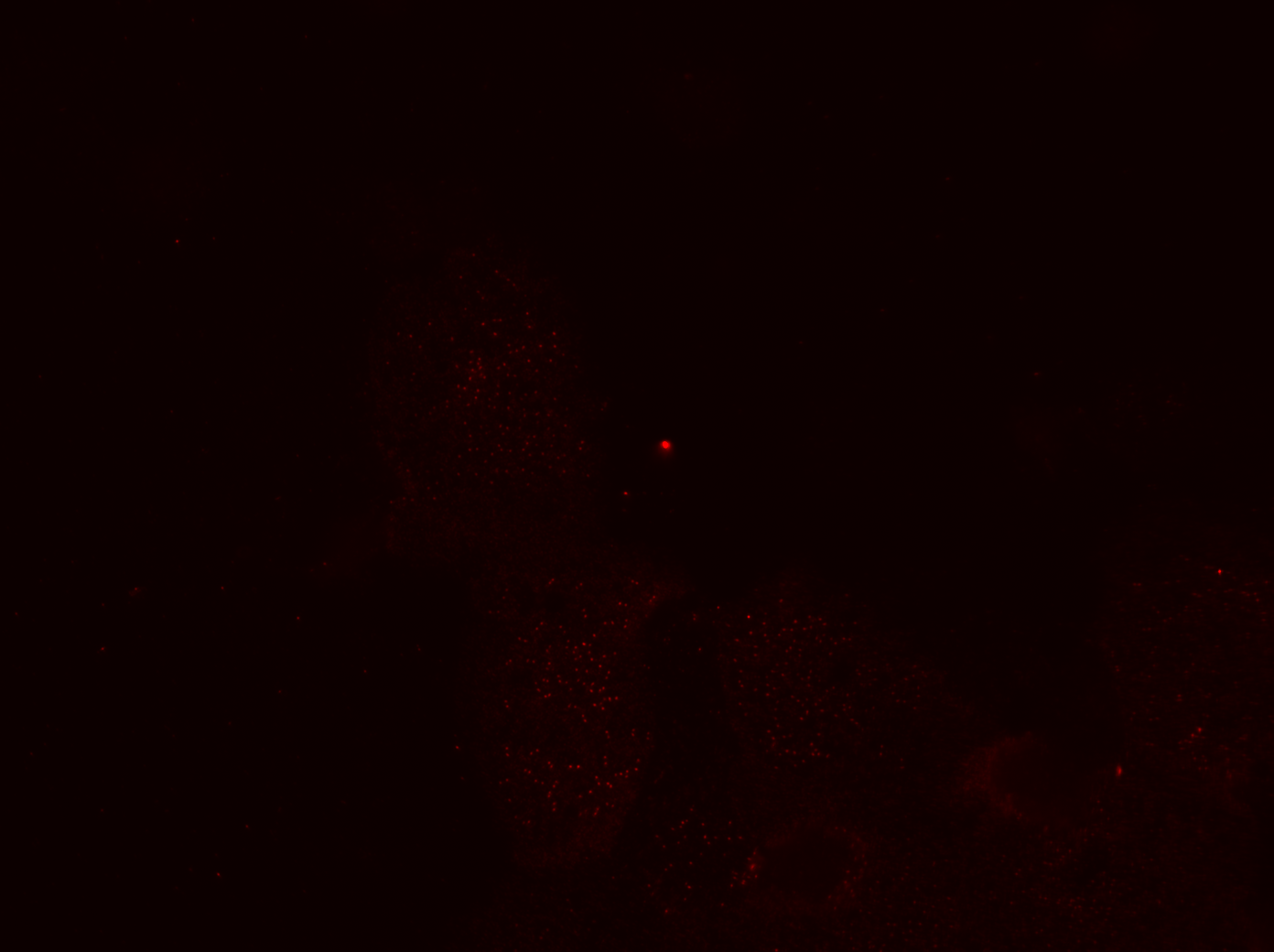

Supplement: Supplementary file 5 — Source data Fig. 1 [file 44318_2024_104_MOESM5_ESM.zip › Figure 1/1E/HeLa siCENP-U#2 completely separated CENP-C.tif]

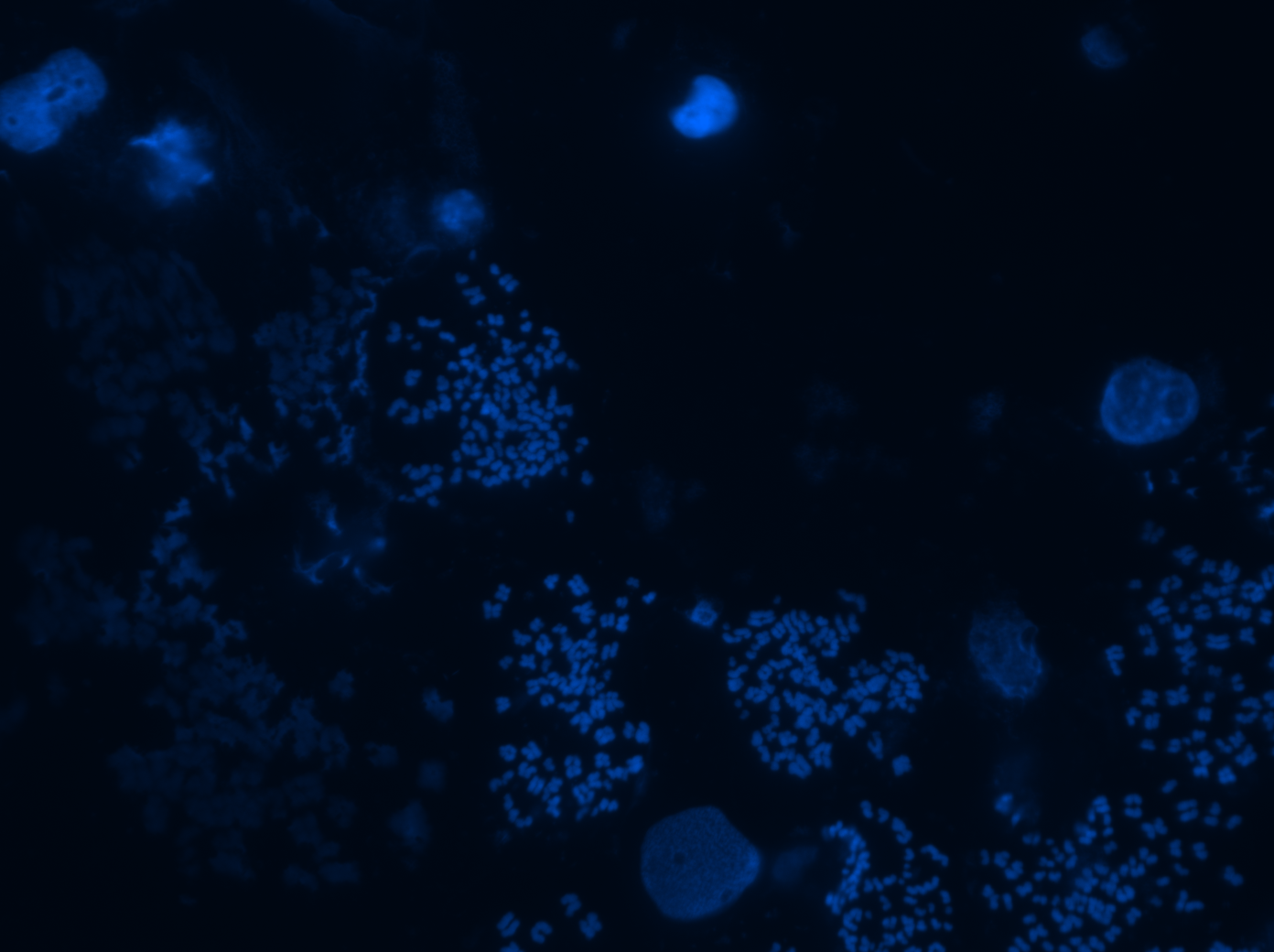

Supplement: Supplementary file 5 — Source data Fig. 1 [file 44318_2024_104_MOESM5_ESM.zip › Figure 1/1E/HeLa siCENP-U#2 completely separated DNA.tif]

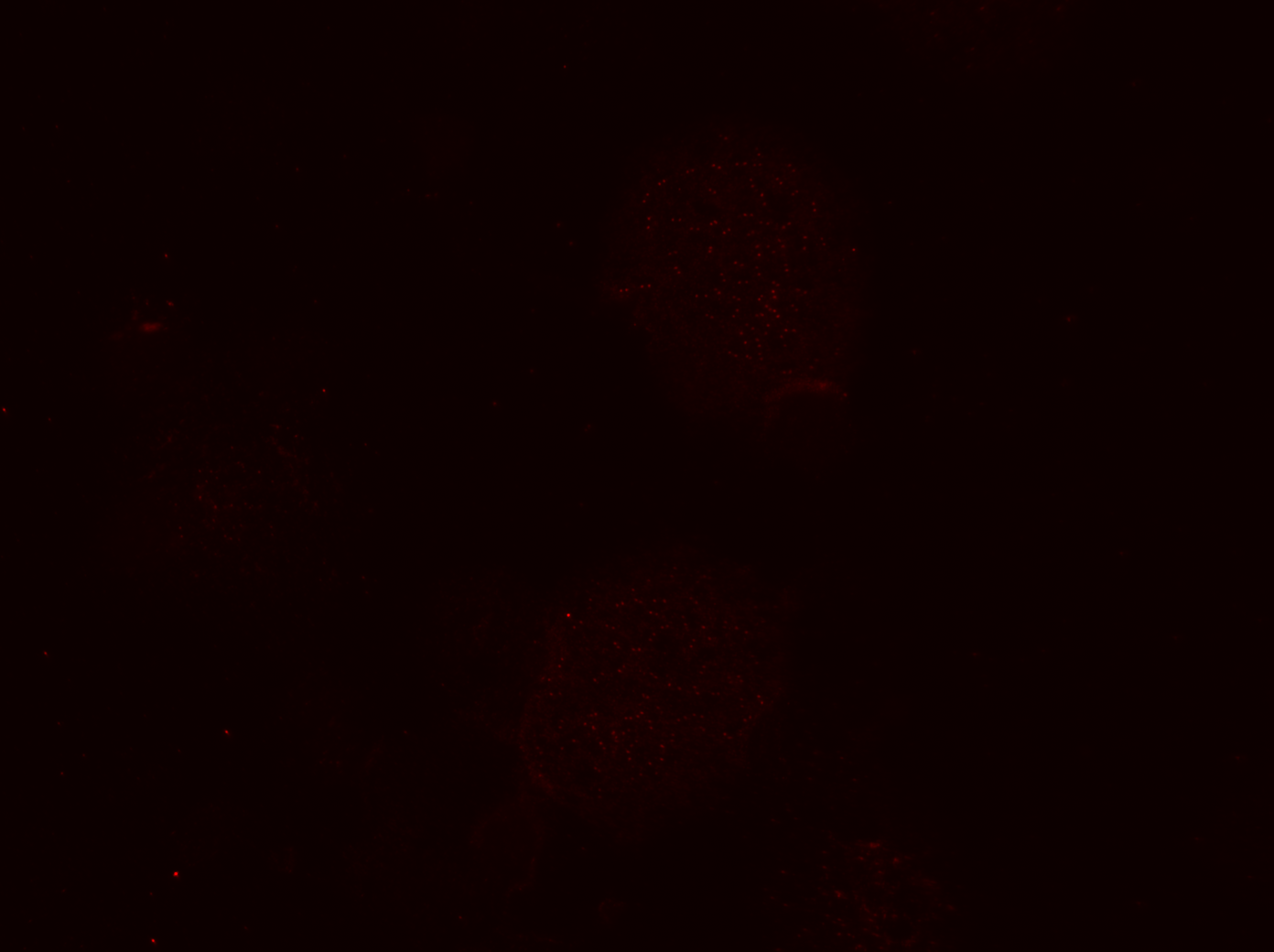

Supplement: Supplementary file 5 — Source data Fig. 1 [file 44318_2024_104_MOESM5_ESM.zip › Figure 1/1E/HeLa siCENP-U#2 partly separated CENP-C.tif]

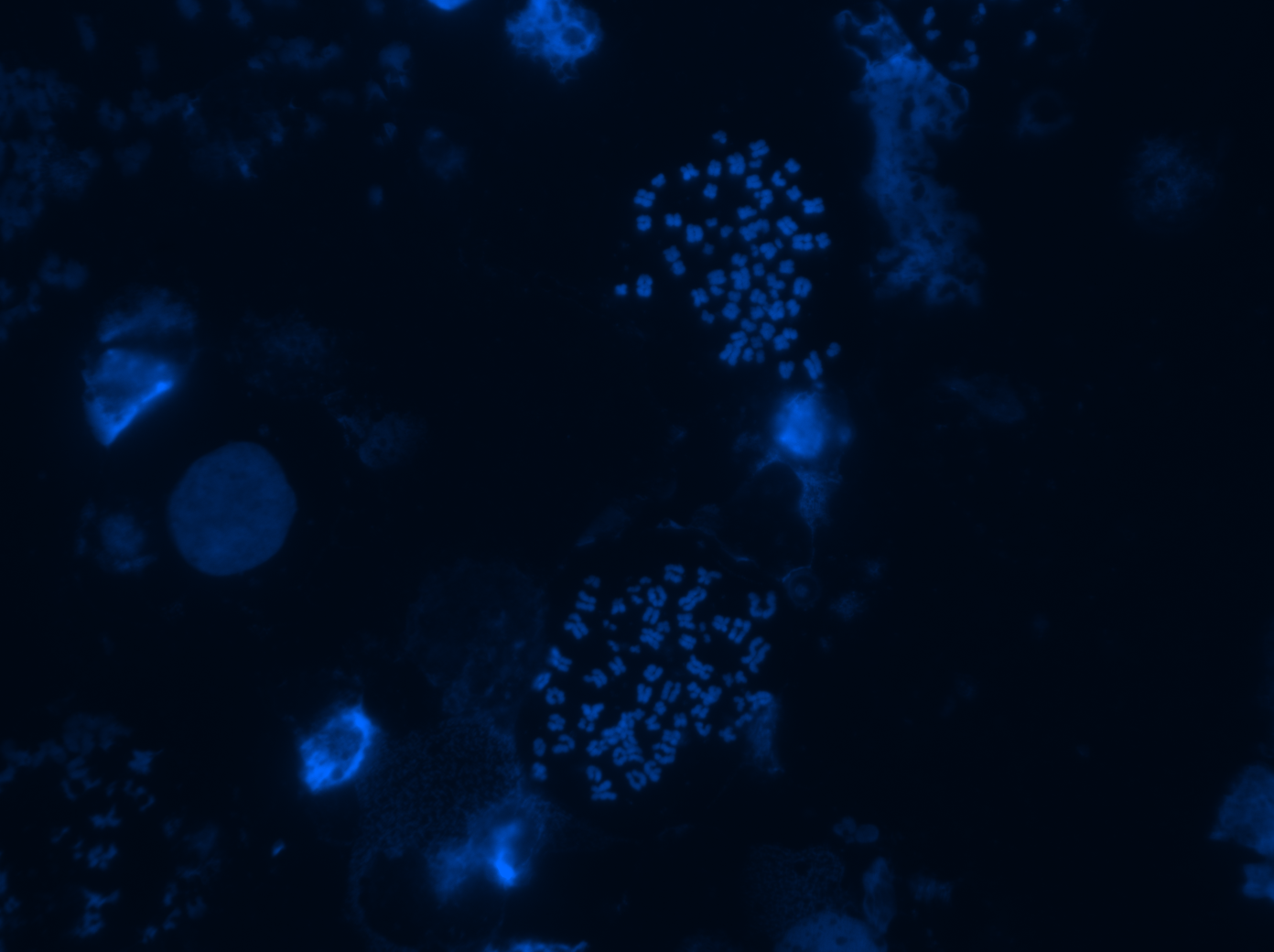

Supplement: Supplementary file 5 — Source data Fig. 1 [file 44318_2024_104_MOESM5_ESM.zip › Figure 1/1E/HeLa siCENP-U#2 partly separated DNA.tif]

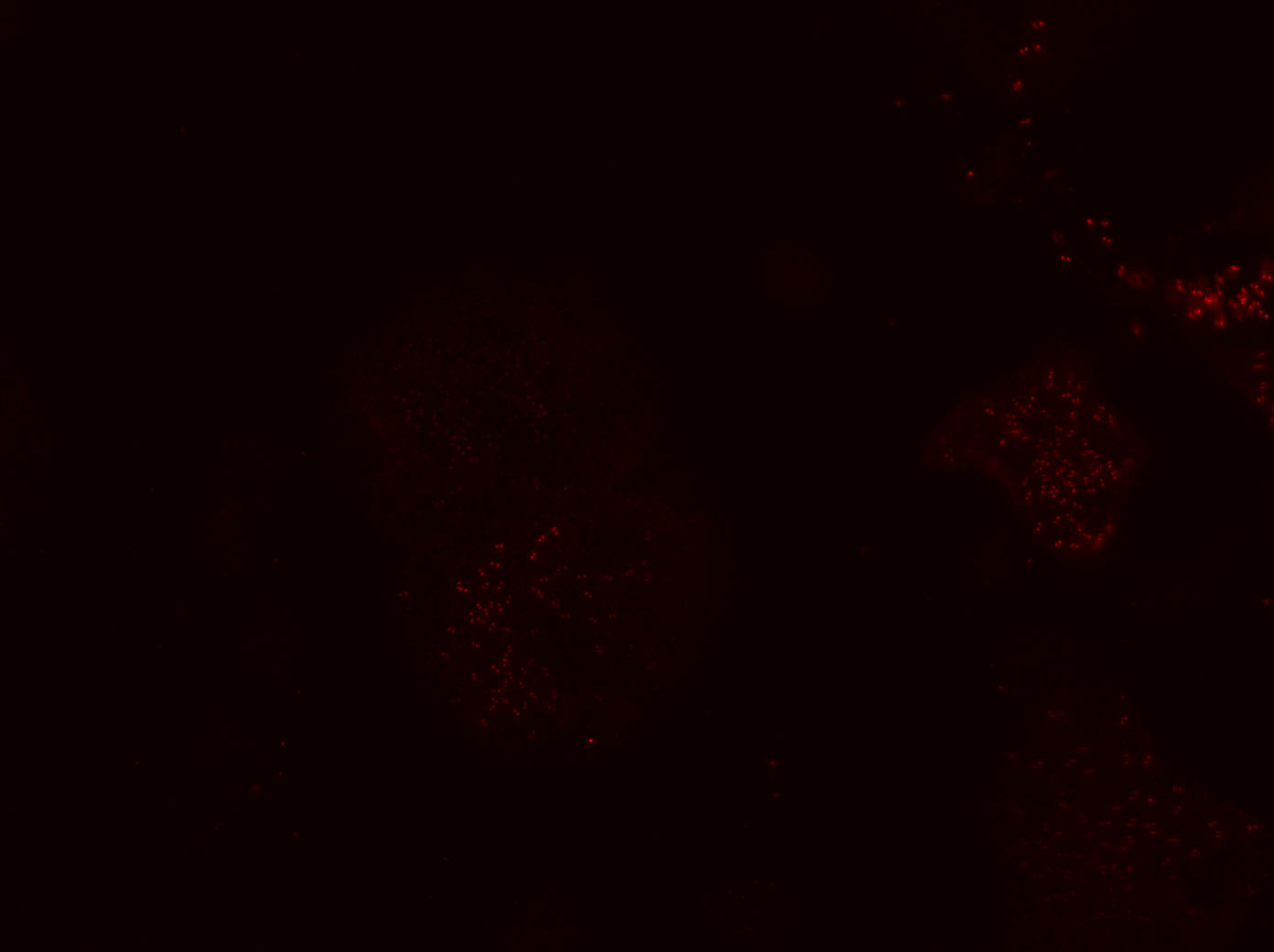

Supplement: Supplementary file 5 — Source data Fig. 1 [file 44318_2024_104_MOESM5_ESM.zip › Figure 1/1E/HeLa+siControl CENP-C.tif]

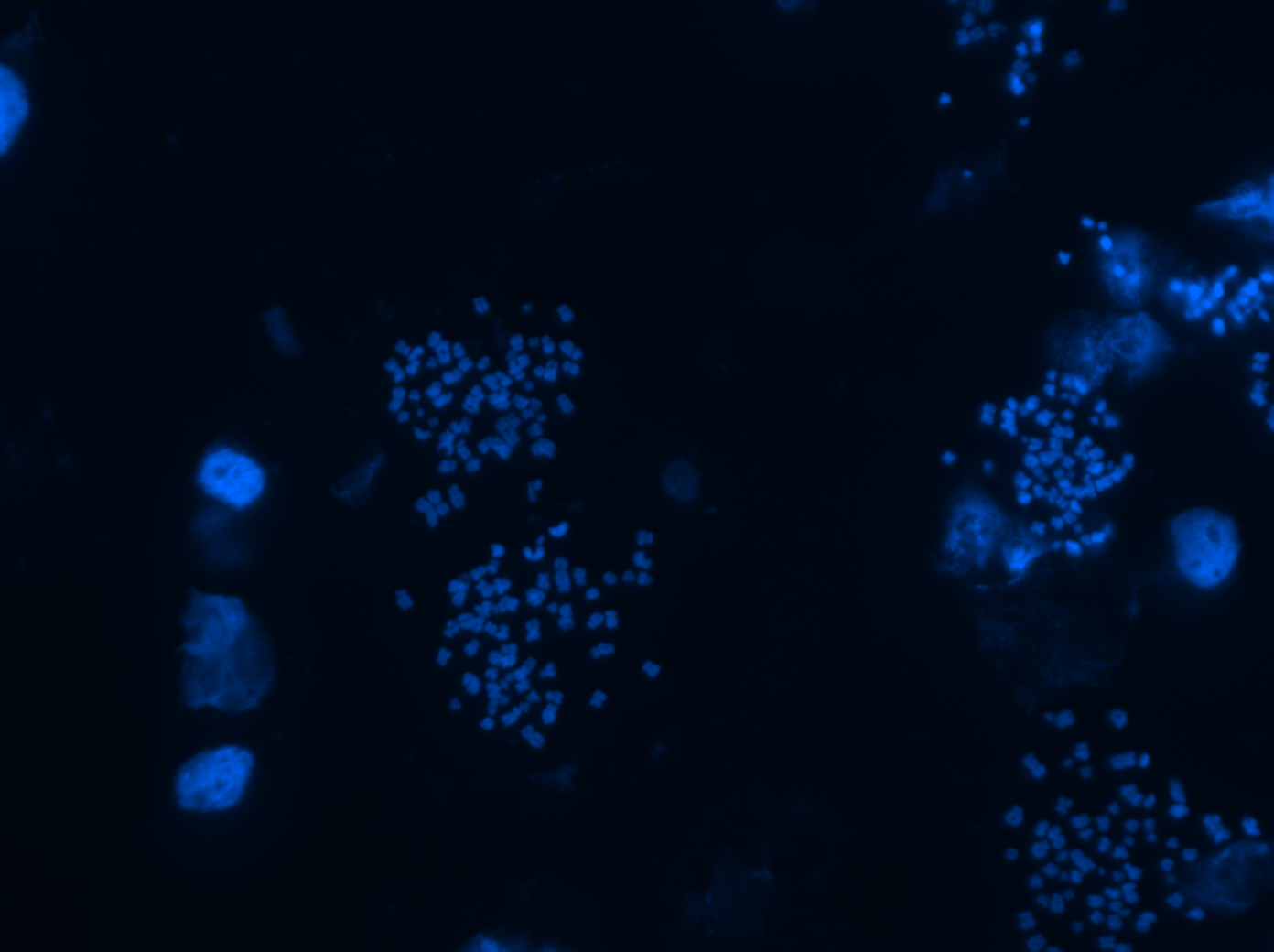

Supplement: Supplementary file 5 — Source data Fig. 1 [file 44318_2024_104_MOESM5_ESM.zip › Figure 1/1E/HeLa+siControl DNA.tif]

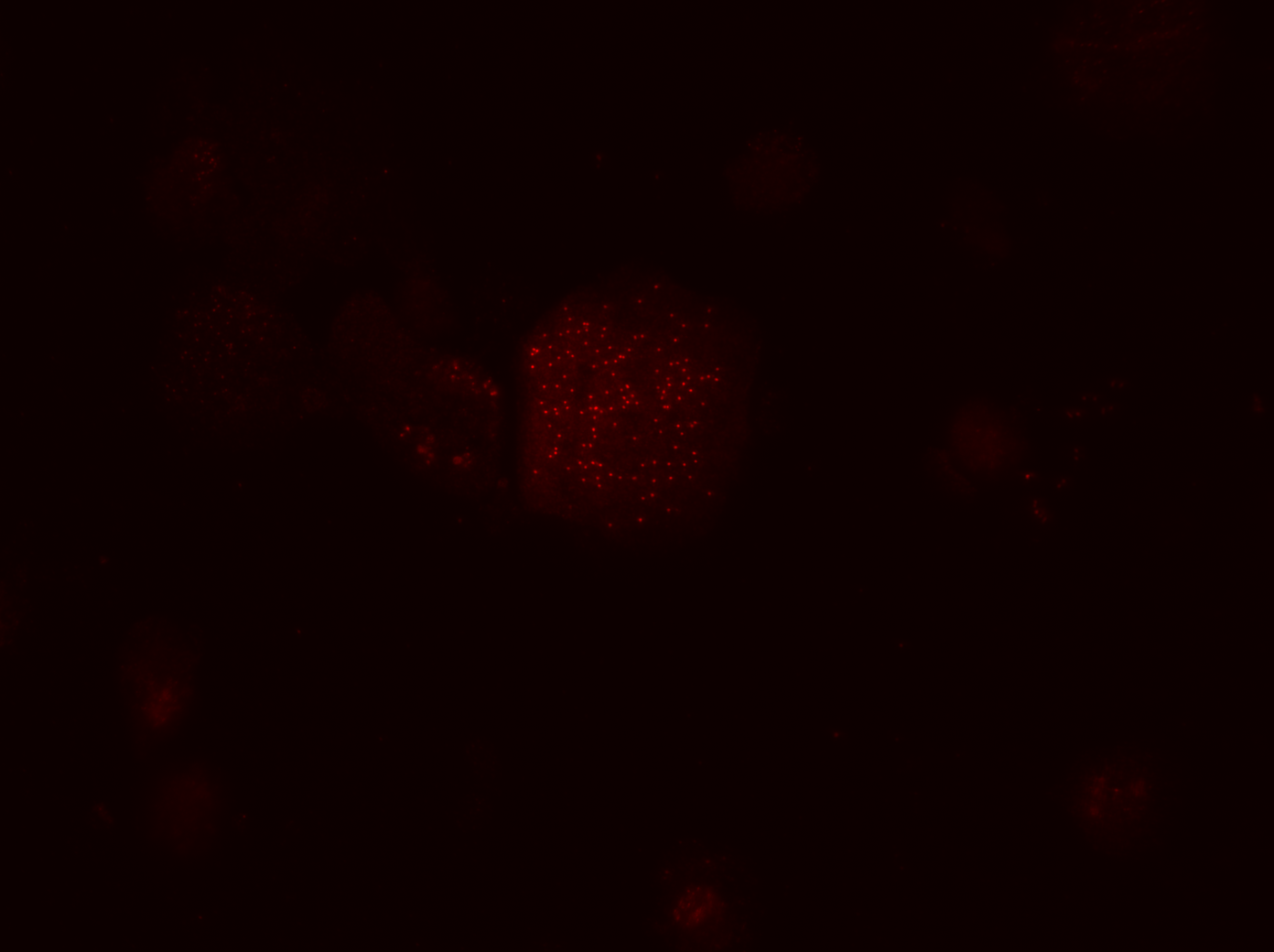

Supplement: Supplementary file 5 — Source data Fig. 1 [file 44318_2024_104_MOESM5_ESM.zip › Figure 1/1G/siCENP-U#1-CENP-C.tif]

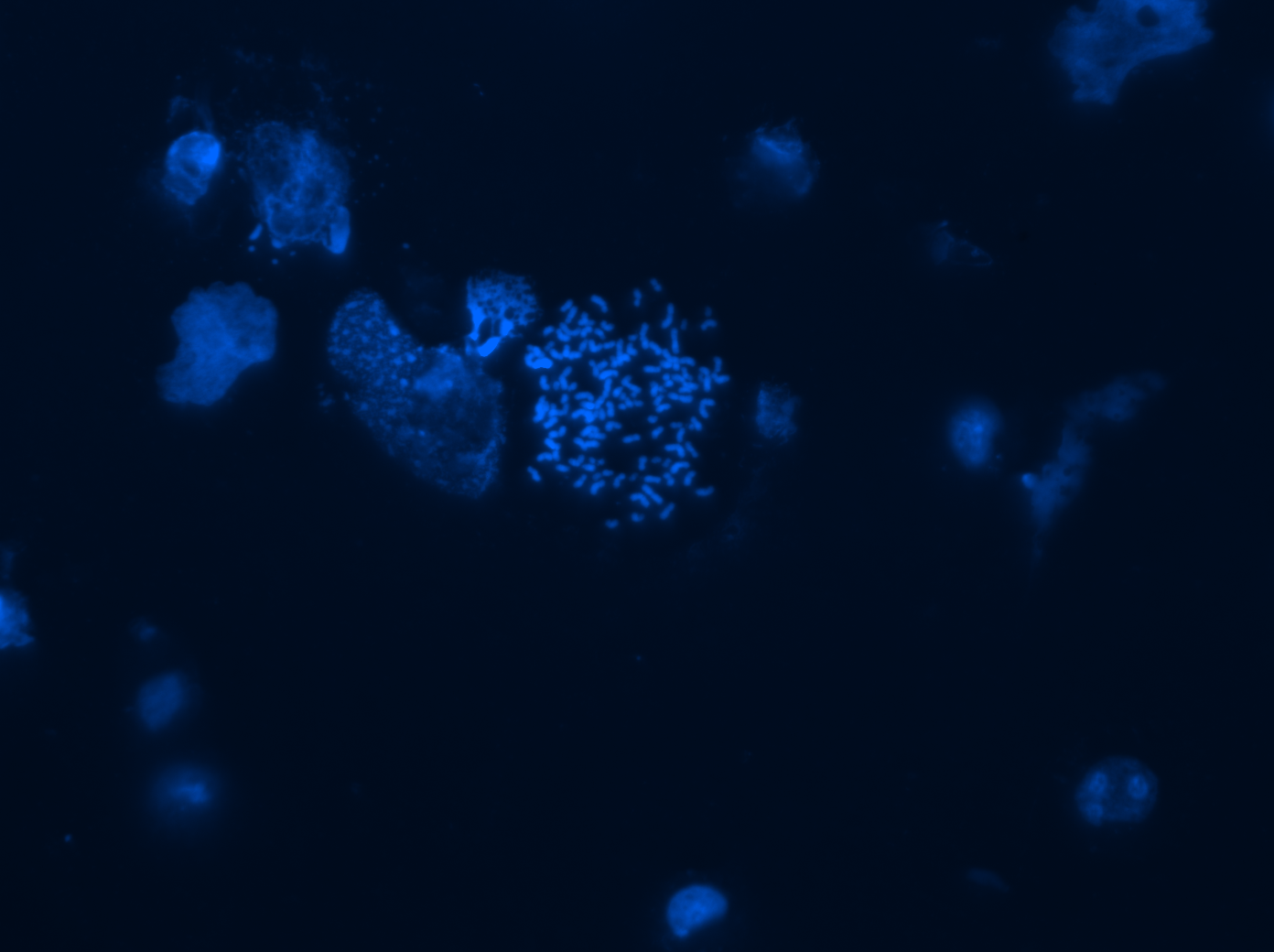

Supplement: Supplementary file 5 — Source data Fig. 1 [file 44318_2024_104_MOESM5_ESM.zip › Figure 1/1G/siCENP-U#1-DNA.tif]

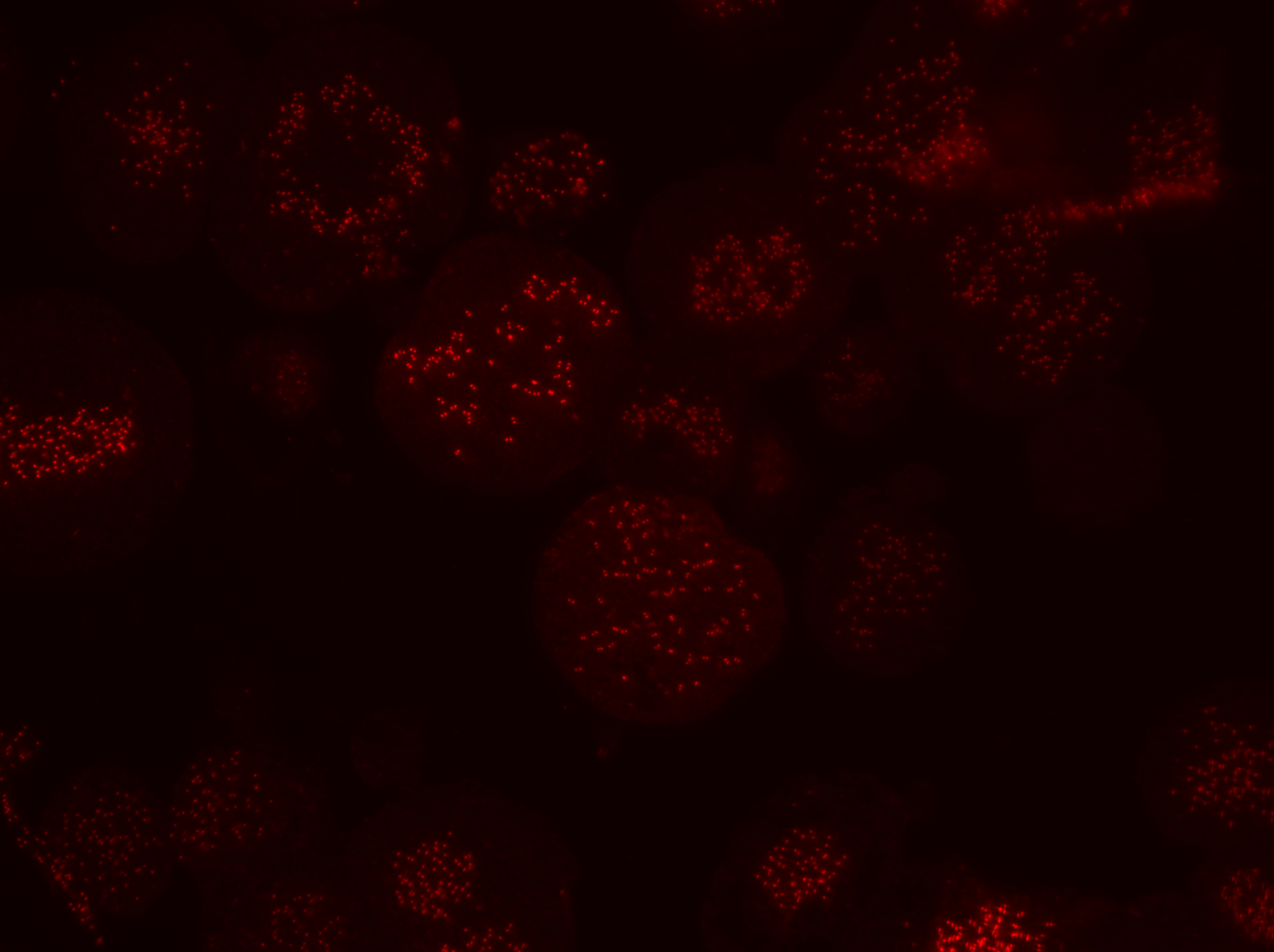

Supplement: Supplementary file 5 — Source data Fig. 1 [file 44318_2024_104_MOESM5_ESM.zip › Figure 1/1G/siControl-CENP-C.tif]

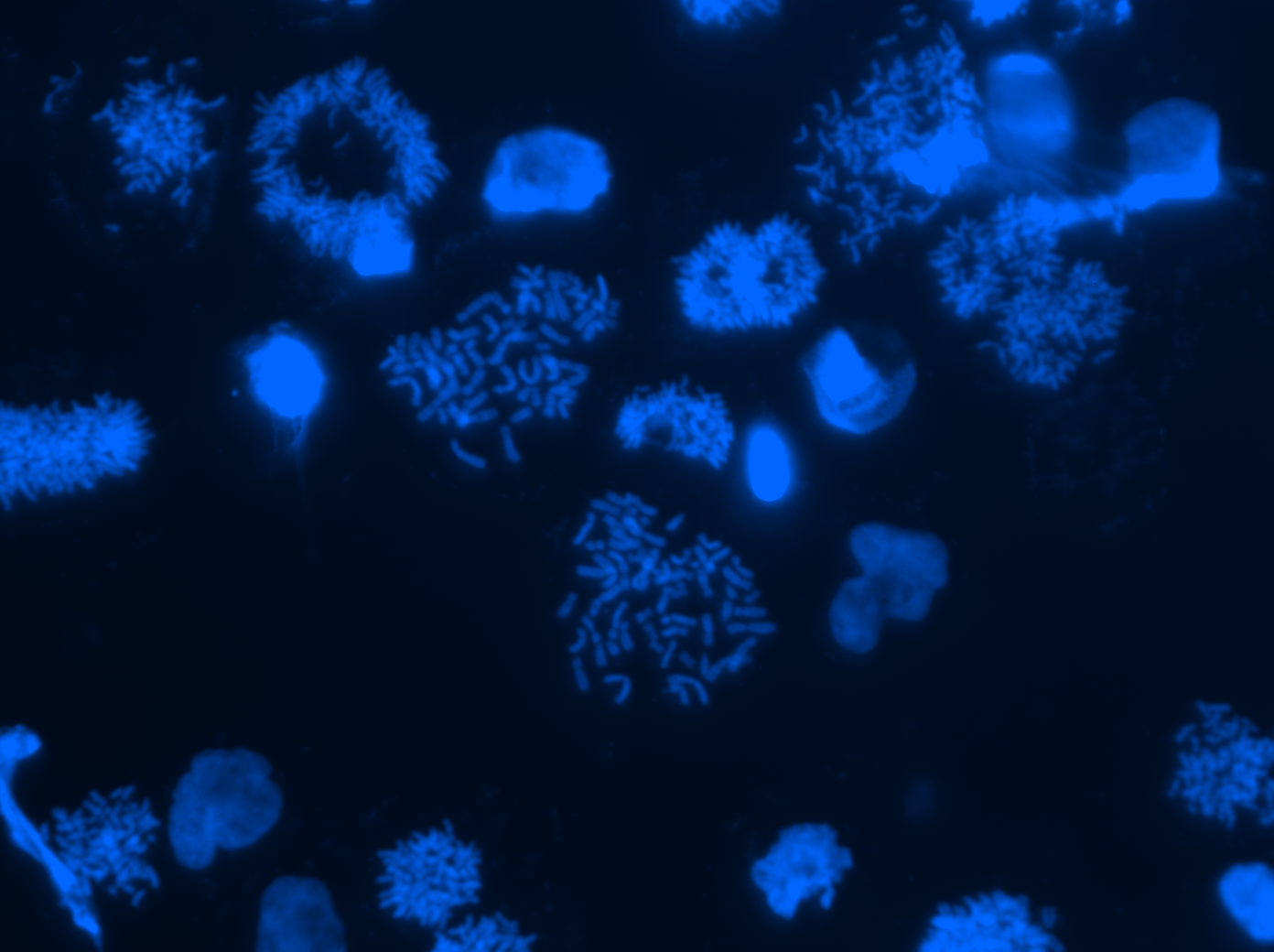

Supplement: Supplementary file 5 — Source data Fig. 1 [file 44318_2024_104_MOESM5_ESM.zip › Figure 1/1G/siControl-DNA.tif]

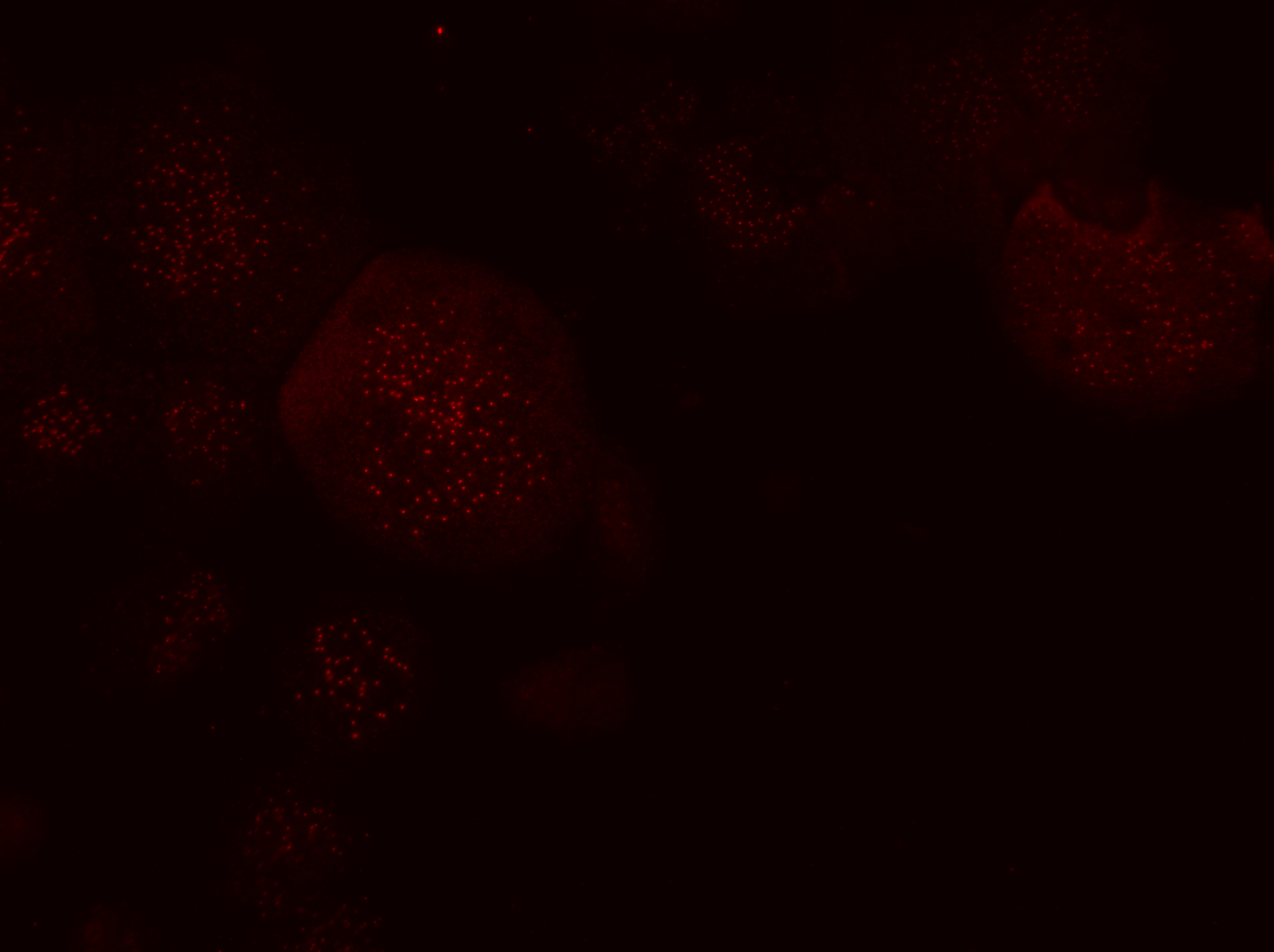

Supplement: Supplementary file 5 — Source data Fig. 1 [file 44318_2024_104_MOESM5_ESM.zip › Figure 1/1I/siCENP-U#2-CENP-C.tif]

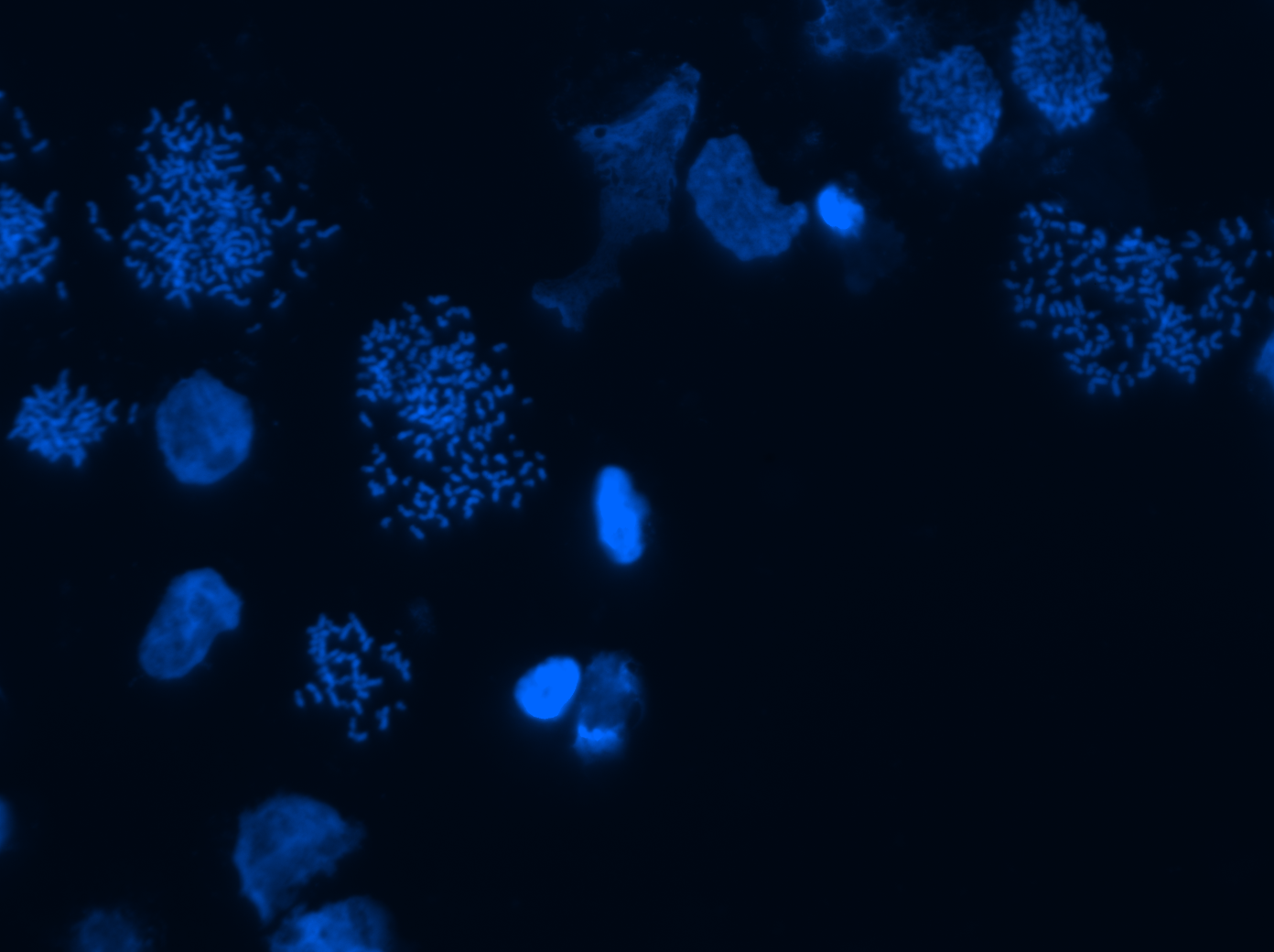

Supplement: Supplementary file 5 — Source data Fig. 1 [file 44318_2024_104_MOESM5_ESM.zip › Figure 1/1I/siCENP-U#2-DNA.tif]

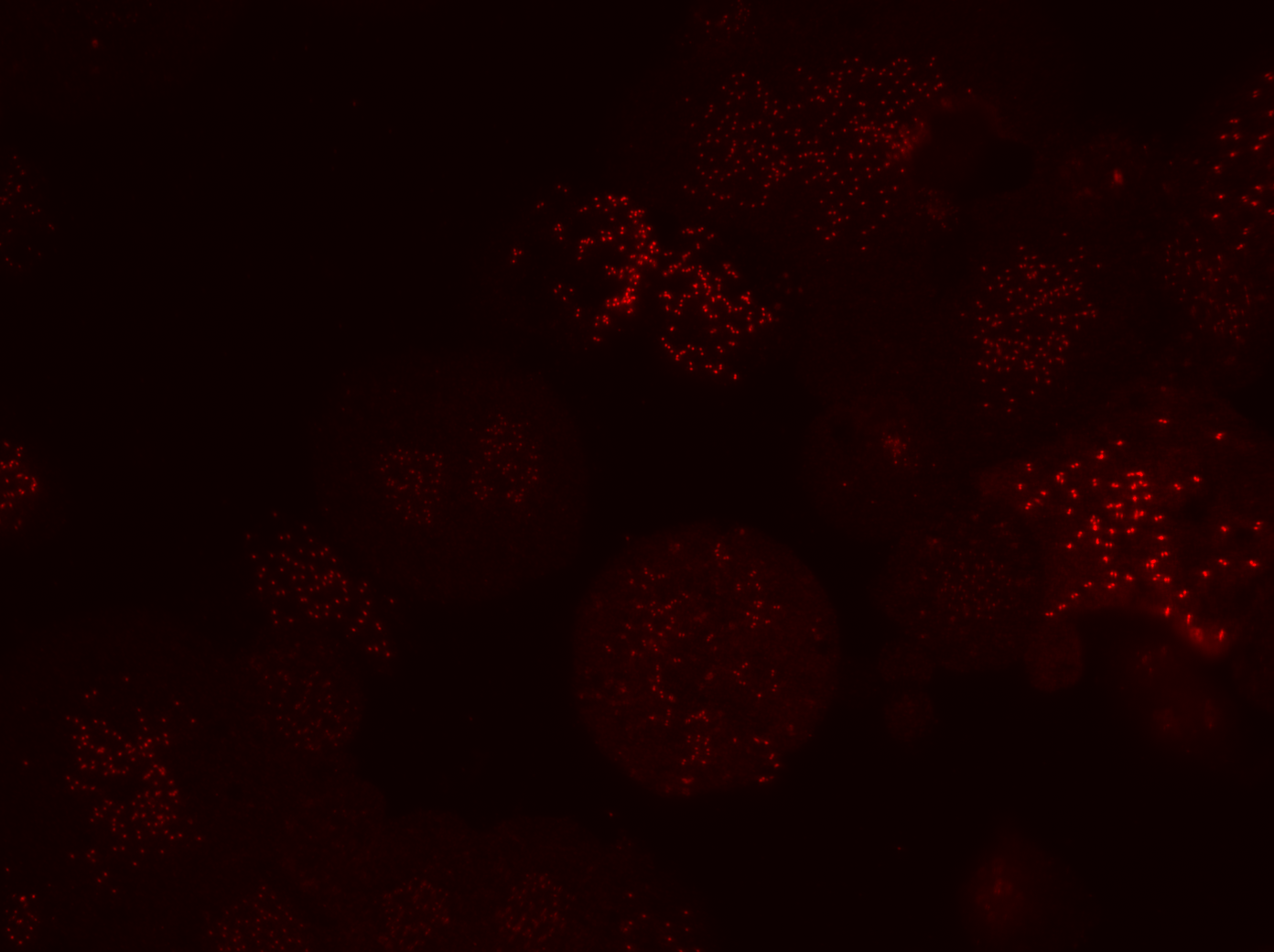

Supplement: Supplementary file 5 — Source data Fig. 1 [file 44318_2024_104_MOESM5_ESM.zip › Figure 1/1I/siControl-CENP-C.tif]

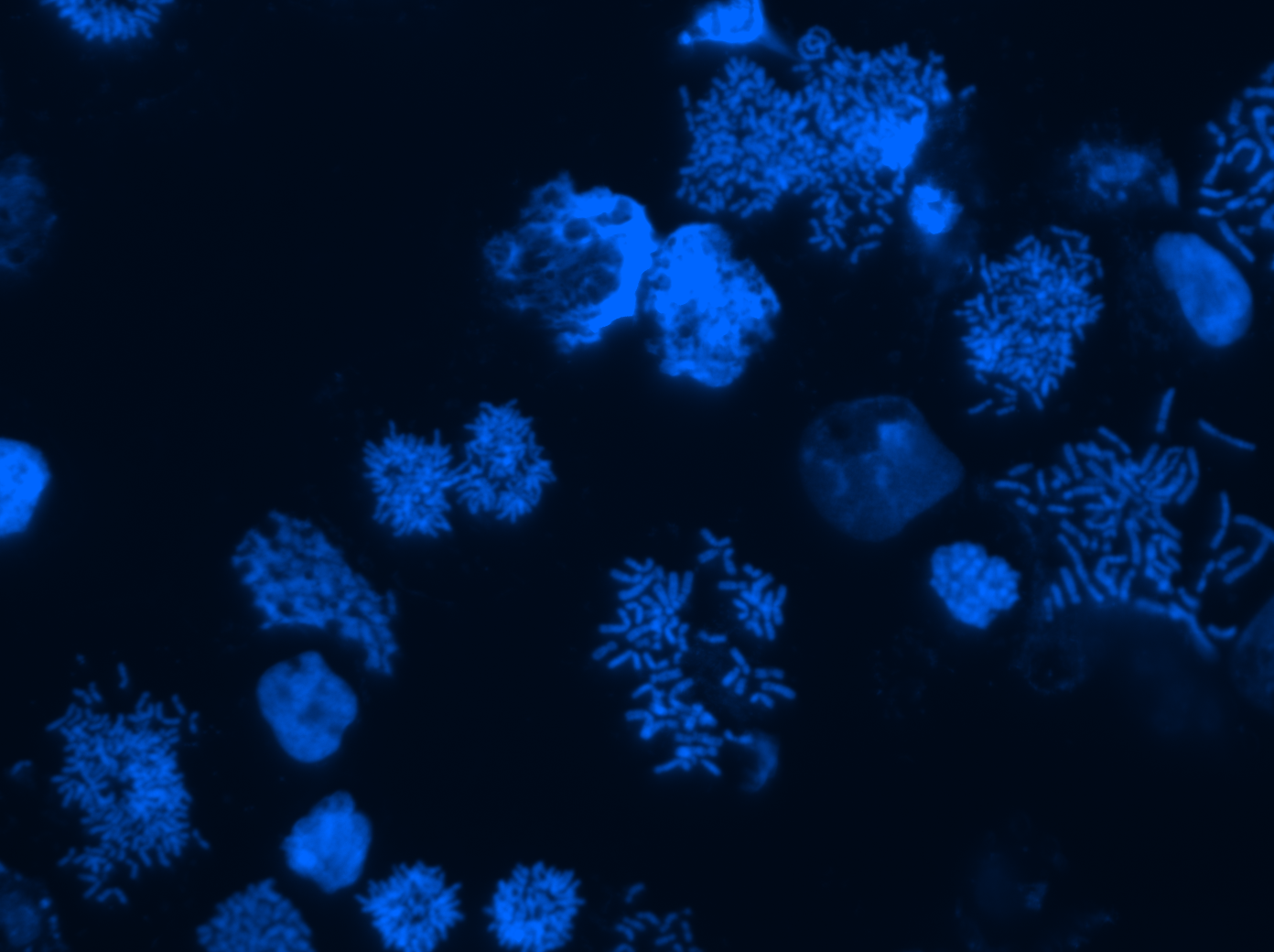

Supplement: Supplementary file 5 — Source data Fig. 1 [file 44318_2024_104_MOESM5_ESM.zip › Figure 1/1I/siControl-DNA.tif]

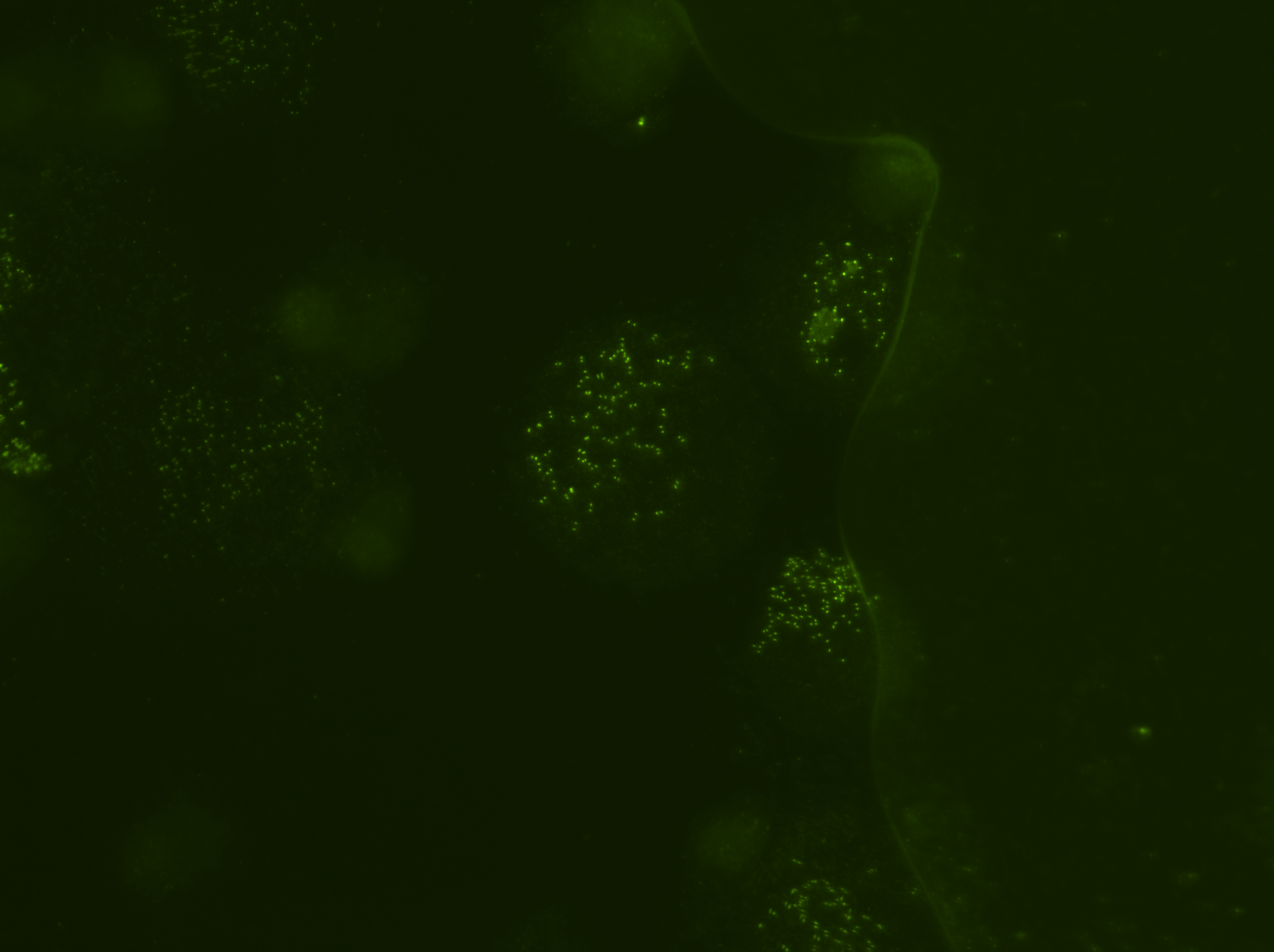

Supplement: Supplementary file 5 — Source data Fig. 1 [file 44318_2024_104_MOESM5_ESM.zip › Figure 1/1L/HeLa+siCENPU#1-CENP-C.tif]

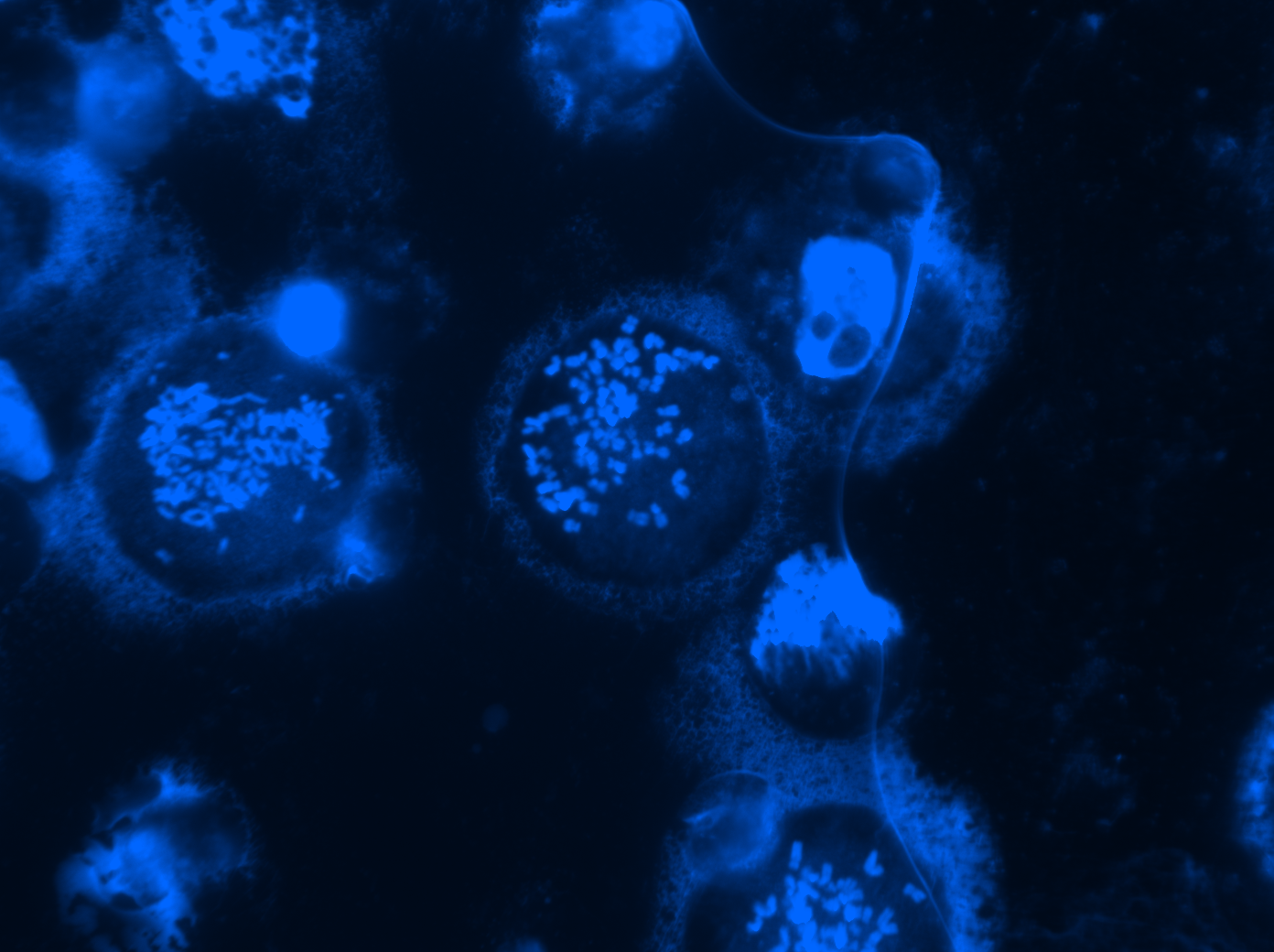

Supplement: Supplementary file 5 — Source data Fig. 1 [file 44318_2024_104_MOESM5_ESM.zip › Figure 1/1L/HeLa+siCENPU#1-DNA.tif]

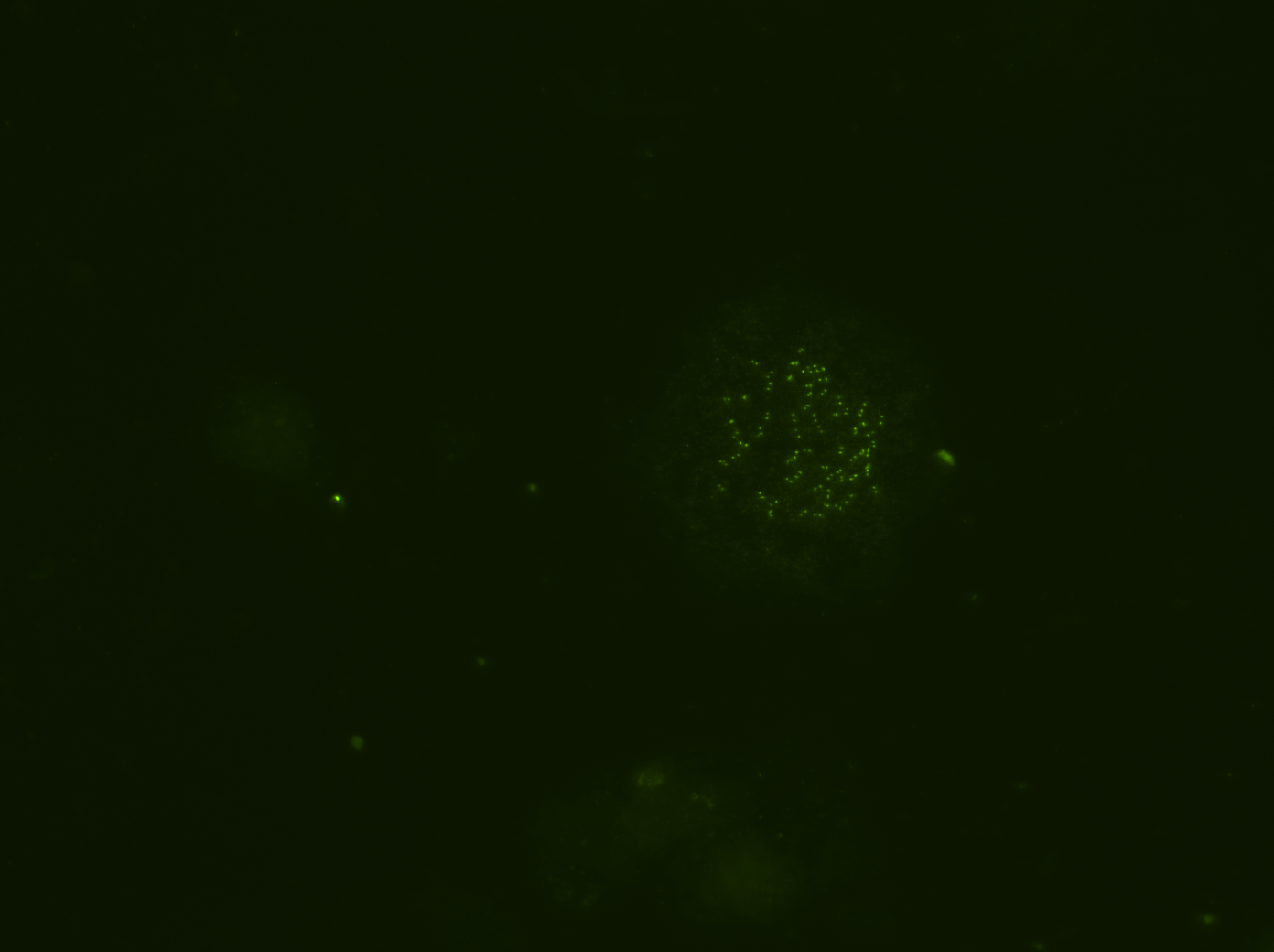

Supplement: Supplementary file 5 — Source data Fig. 1 [file 44318_2024_104_MOESM5_ESM.zip › Figure 1/1L/HeLa+siCENPU#2-CENP-C.tif]

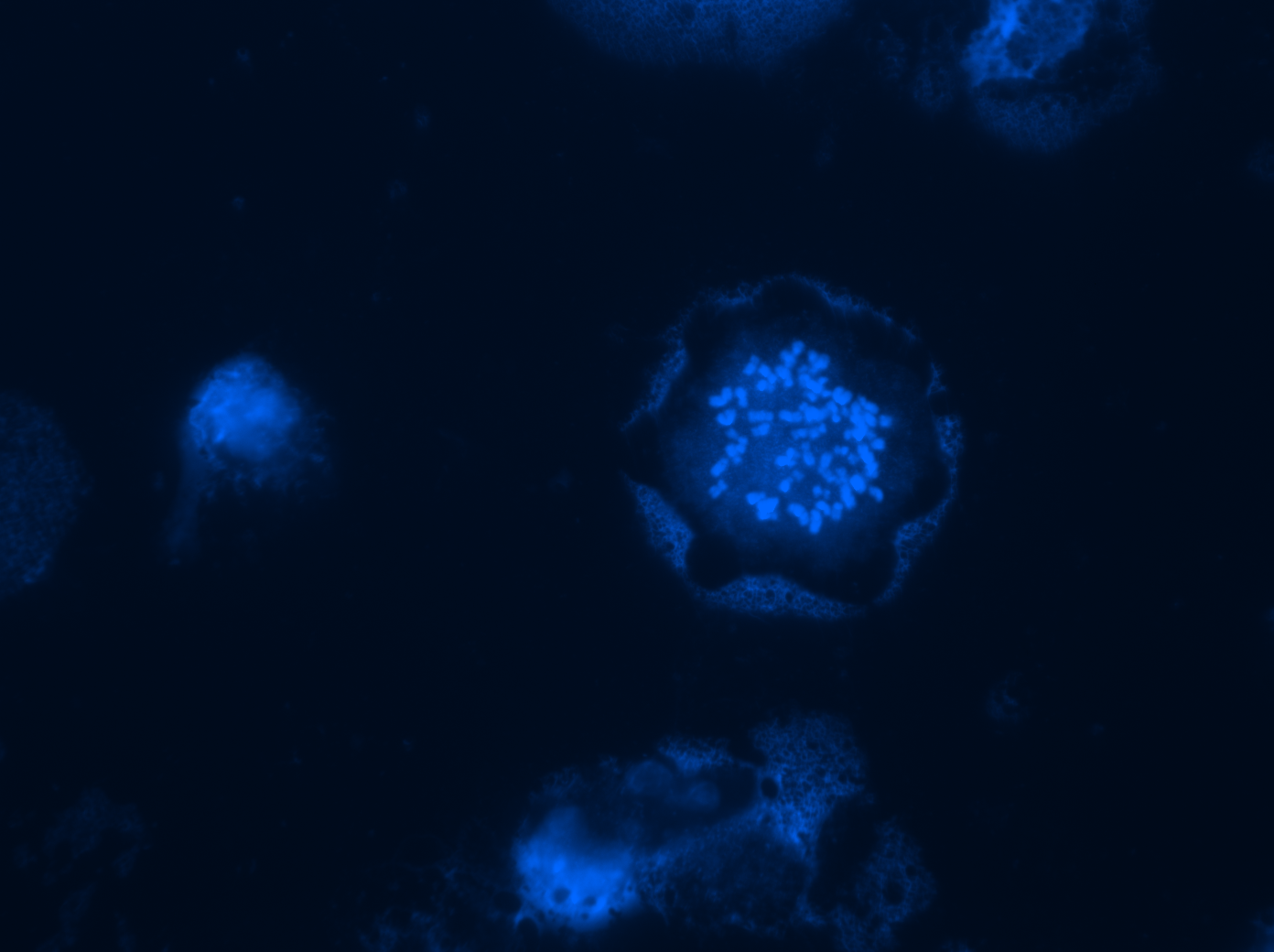

Supplement: Supplementary file 5 — Source data Fig. 1 [file 44318_2024_104_MOESM5_ESM.zip › Figure 1/1L/HeLa+siCENPU#2-DNA.tif]

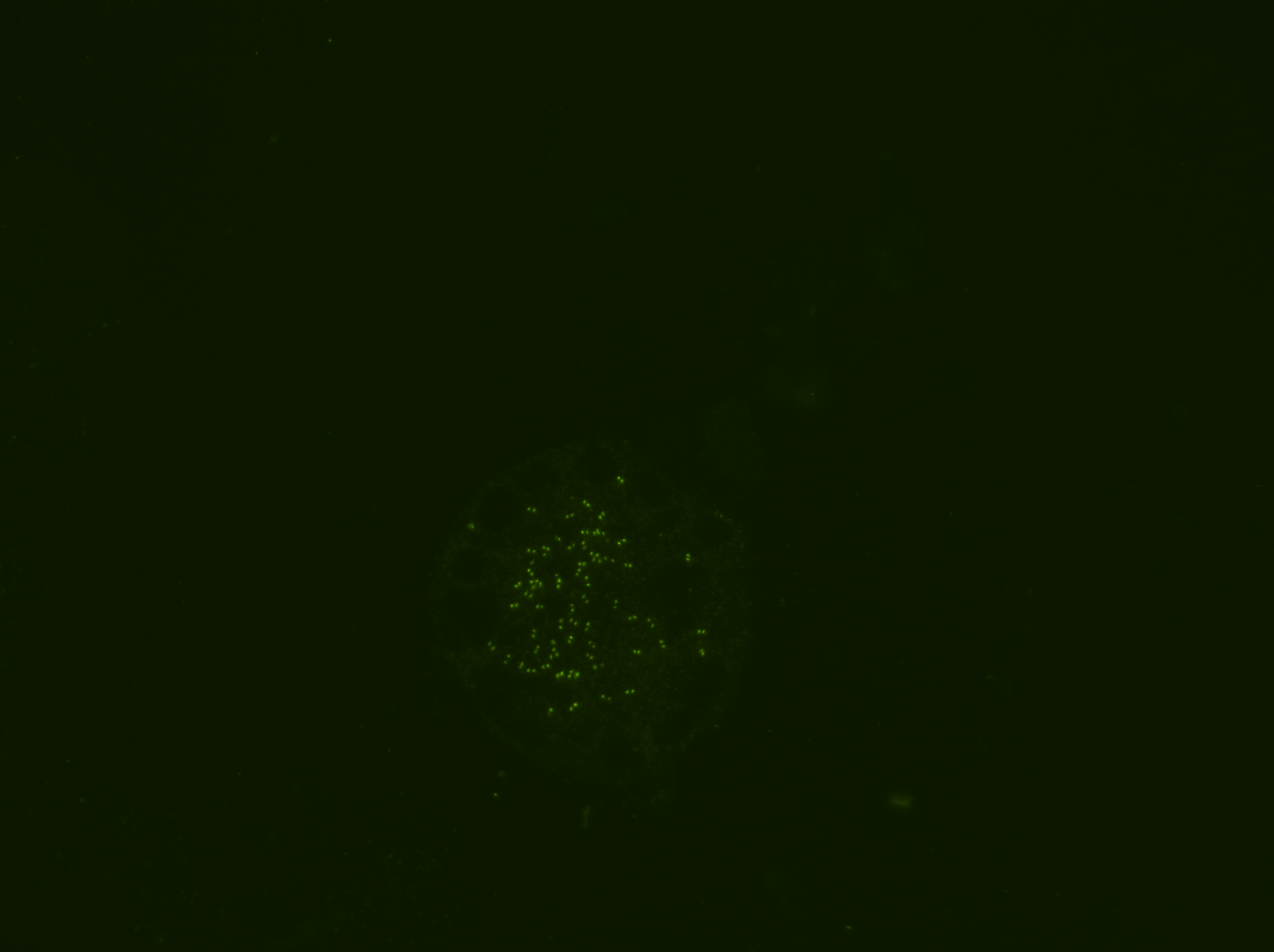

Supplement: Supplementary file 5 — Source data Fig. 1 [file 44318_2024_104_MOESM5_ESM.zip › Figure 1/1L/HeLa+siControl-CENP-C.tif]

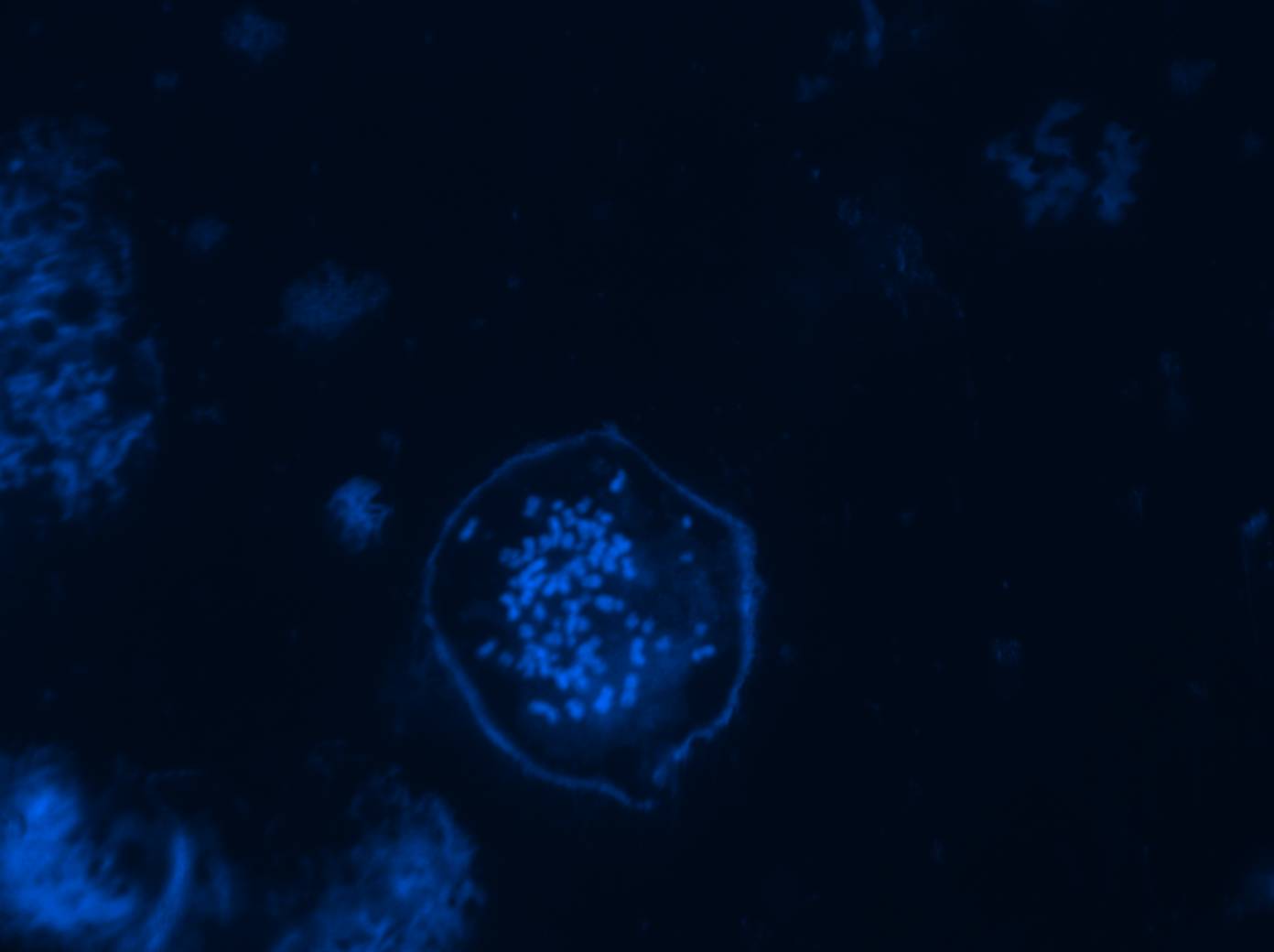

Supplement: Supplementary file 5 — Source data Fig. 1 [file 44318_2024_104_MOESM5_ESM.zip › Figure 1/1L/HeLa+siControl-DNA.tif]

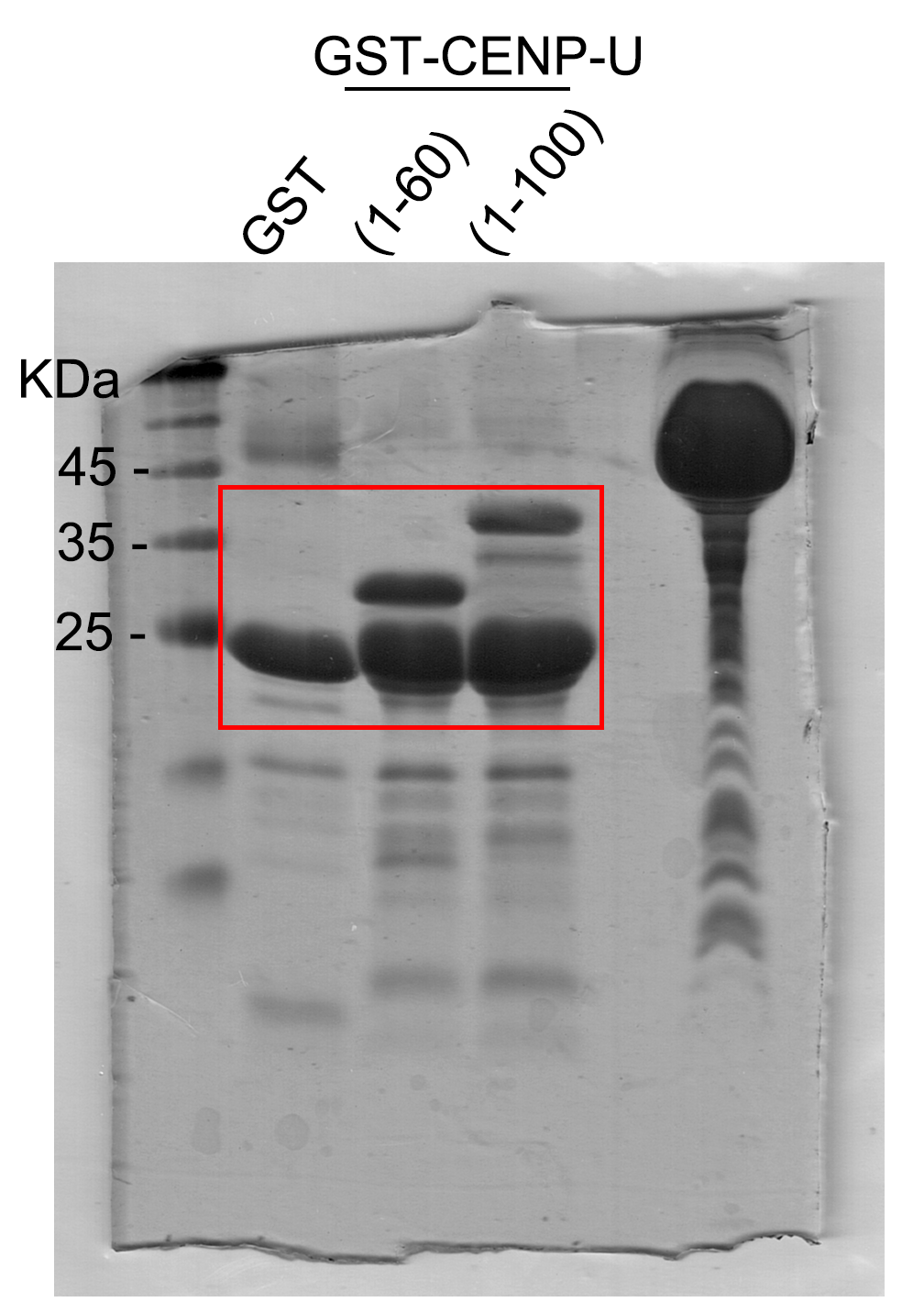

Supplement: Supplementary file 6 — Source data Fig. 2 [file 44318_2024_104_MOESM6_ESM.zip › Figure 2/2A/CBB.tif]

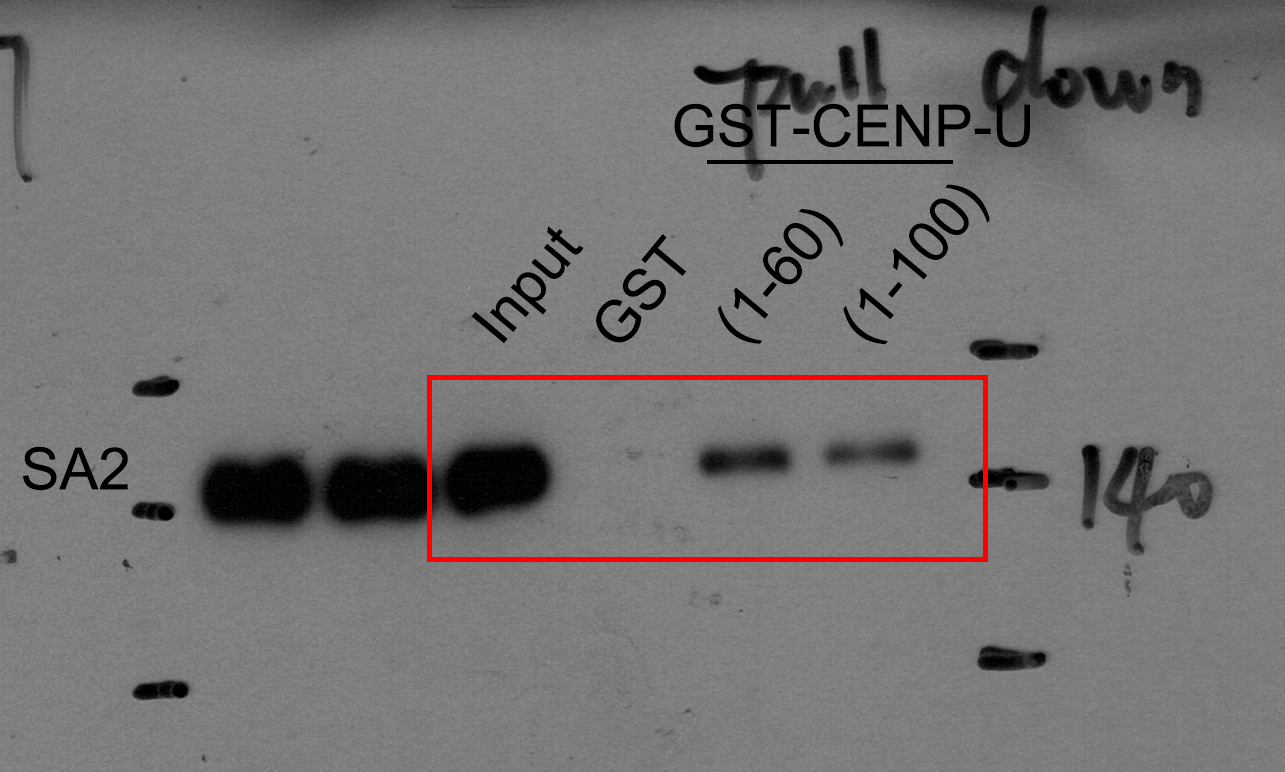

Supplement: Supplementary file 6 — Source data Fig. 2 [file 44318_2024_104_MOESM6_ESM.zip › Figure 2/2A/western SA2.tif]

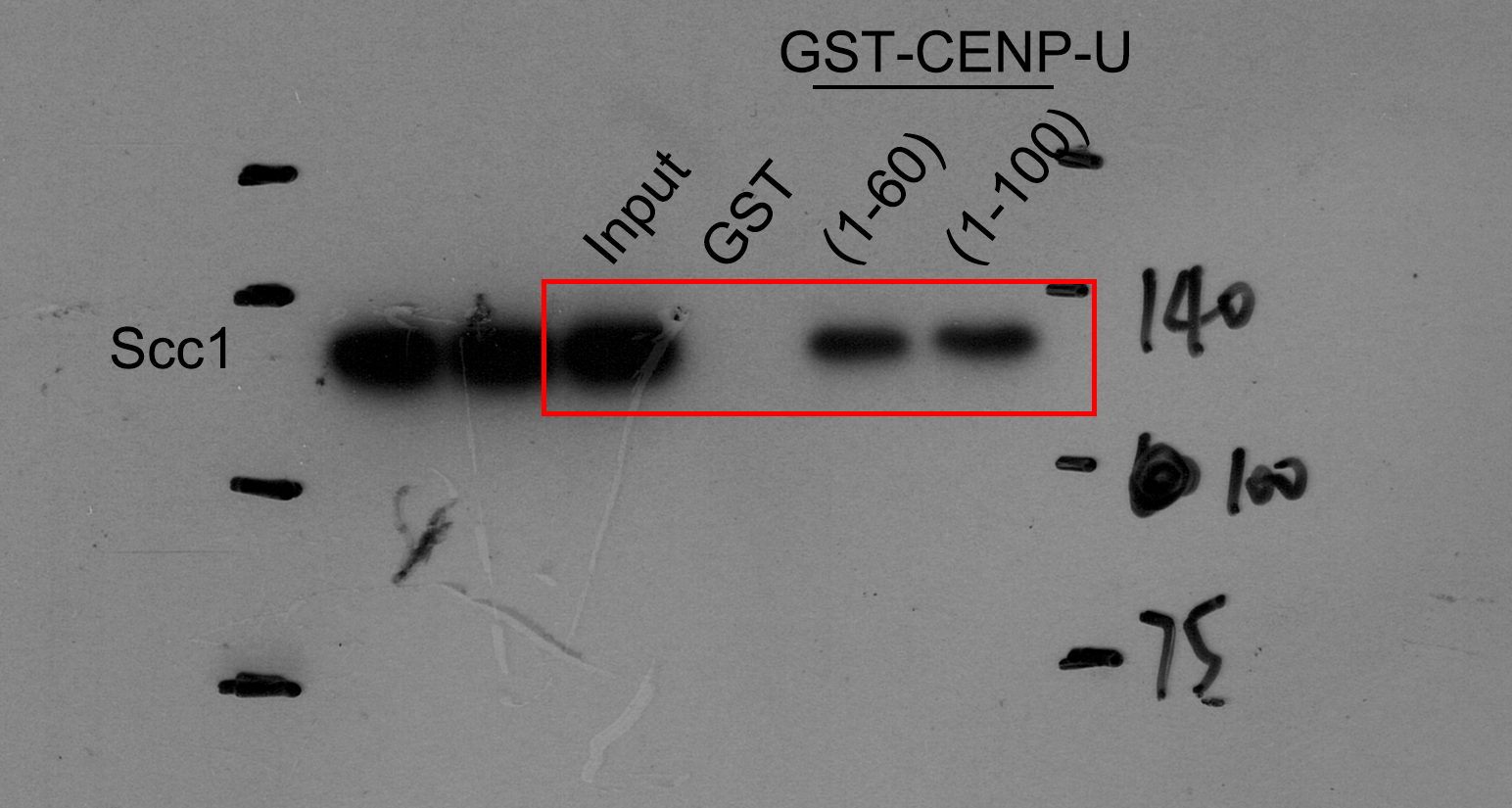

Supplement: Supplementary file 6 — Source data Fig. 2 [file 44318_2024_104_MOESM6_ESM.zip › Figure 2/2A/western Scc1.tif]

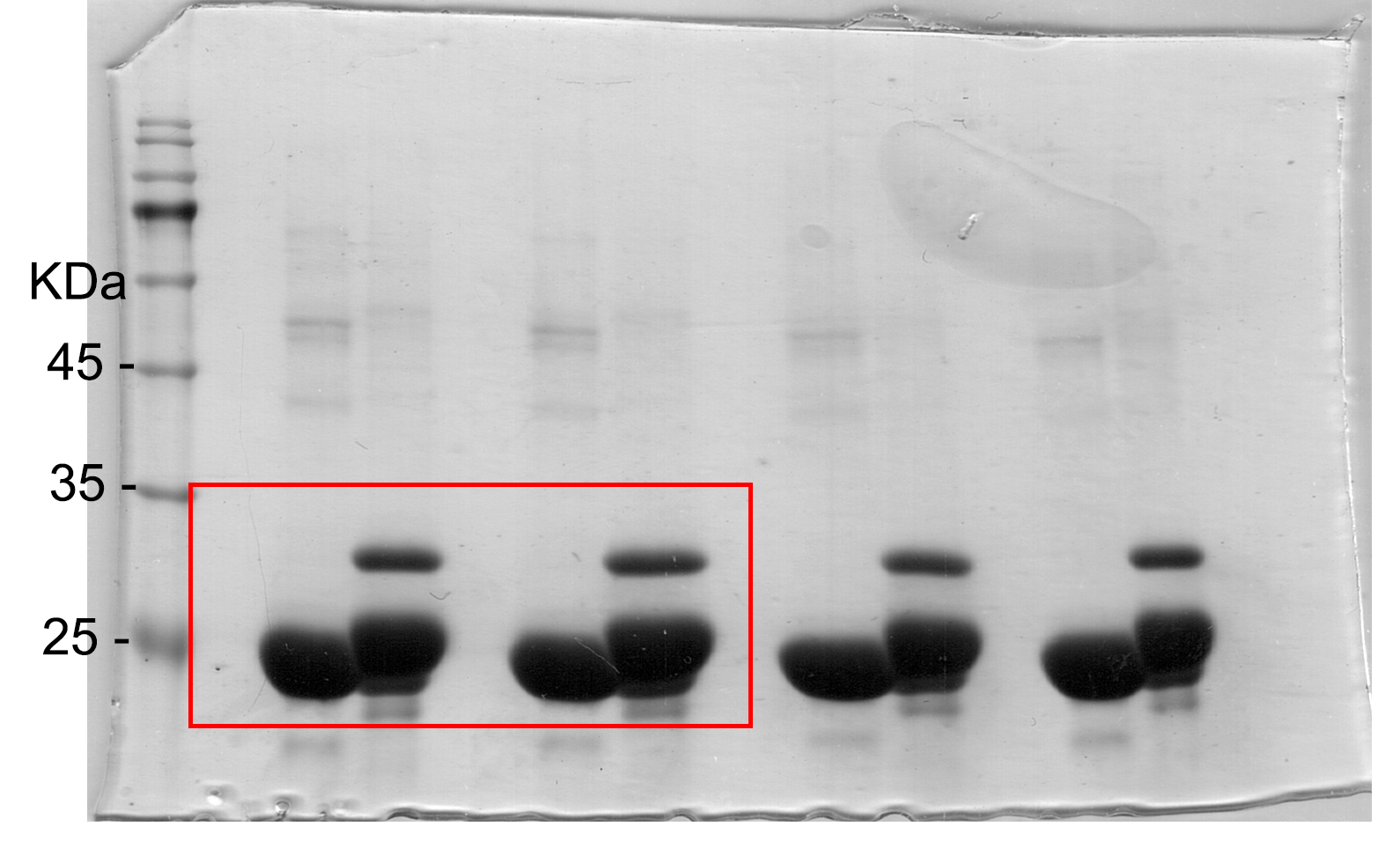

Supplement: Supplementary file 6 — Source data Fig. 2 [file 44318_2024_104_MOESM6_ESM.zip › Figure 2/2B/CBB.tif]

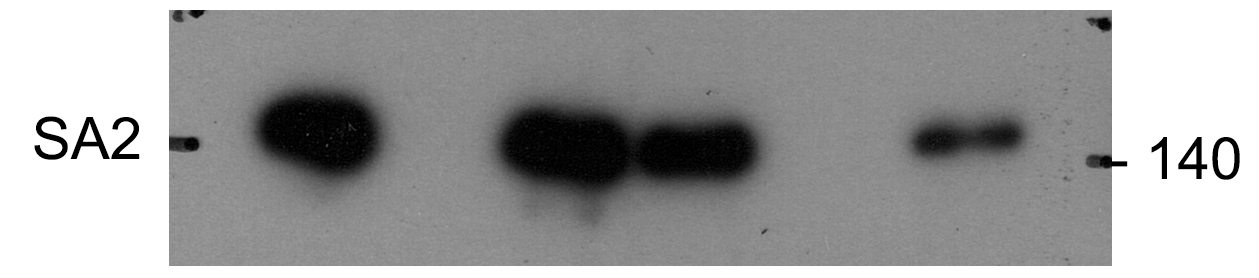

Supplement: Supplementary file 6 — Source data Fig. 2 [file 44318_2024_104_MOESM6_ESM.zip › Figure 2/2B/western SA2.tif]

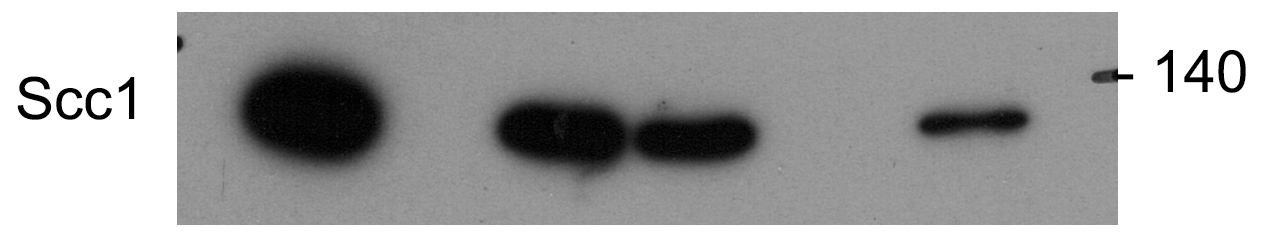

Supplement: Supplementary file 6 — Source data Fig. 2 [file 44318_2024_104_MOESM6_ESM.zip › Figure 2/2B/western Scc1.tif]

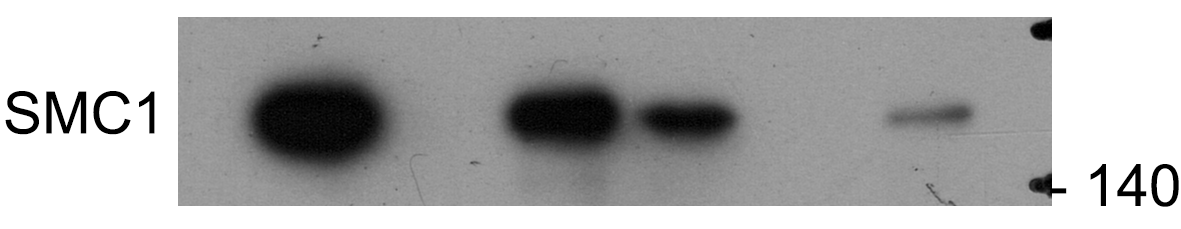

Supplement: Supplementary file 6 — Source data Fig. 2 [file 44318_2024_104_MOESM6_ESM.zip › Figure 2/2B/western SMC1.tif]

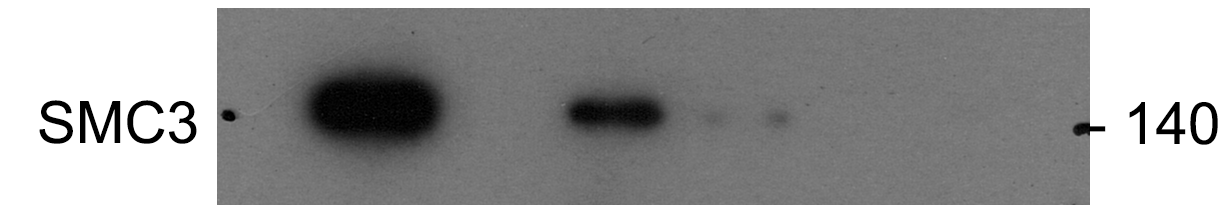

Supplement: Supplementary file 6 — Source data Fig. 2 [file 44318_2024_104_MOESM6_ESM.zip › Figure 2/2B/western SMC3.tif]

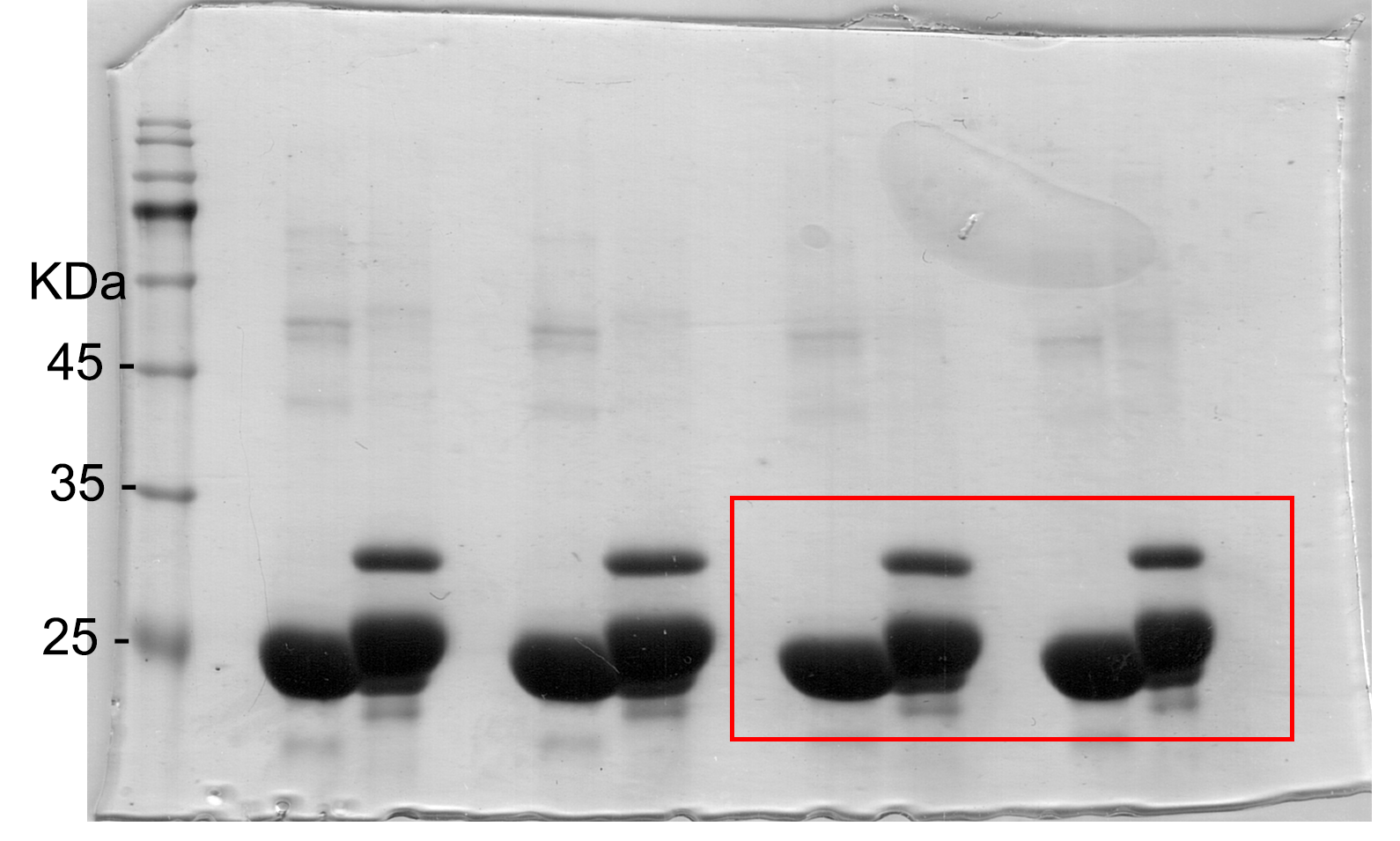

Supplement: Supplementary file 6 — Source data Fig. 2 [file 44318_2024_104_MOESM6_ESM.zip › Figure 2/2C/CBB.tif]

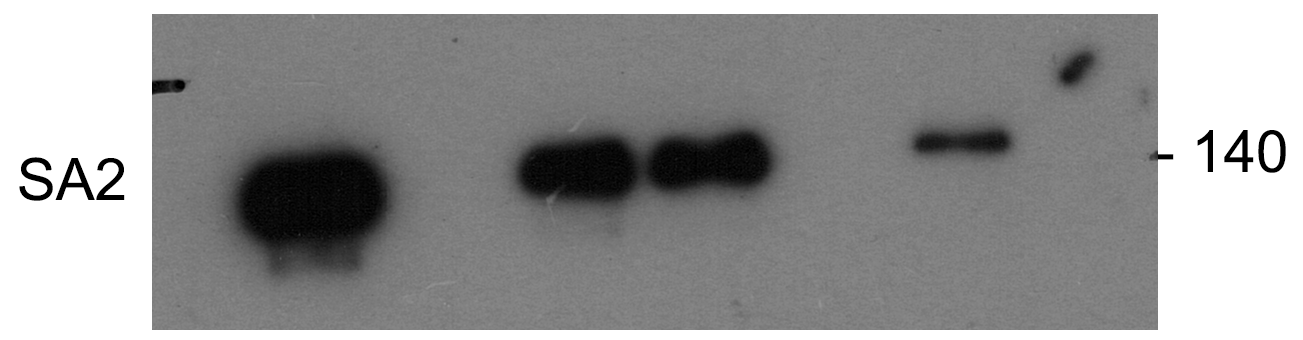

Supplement: Supplementary file 6 — Source data Fig. 2 [file 44318_2024_104_MOESM6_ESM.zip › Figure 2/2C/western SA2.tif]

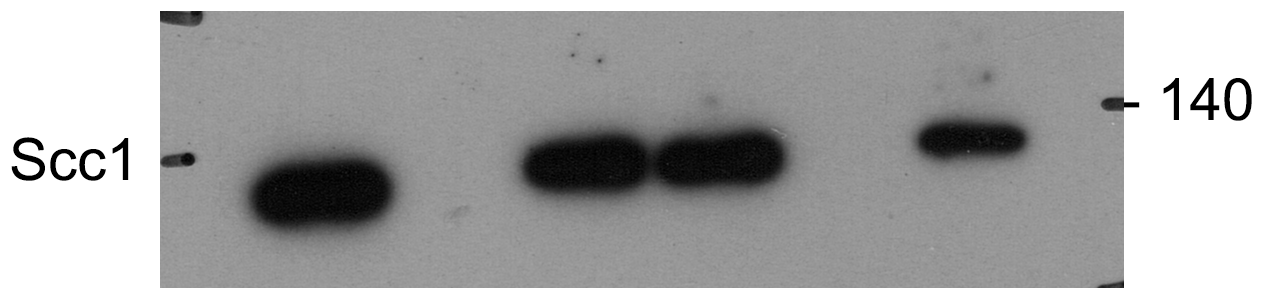

Supplement: Supplementary file 6 — Source data Fig. 2 [file 44318_2024_104_MOESM6_ESM.zip › Figure 2/2C/western Scc1.tif]

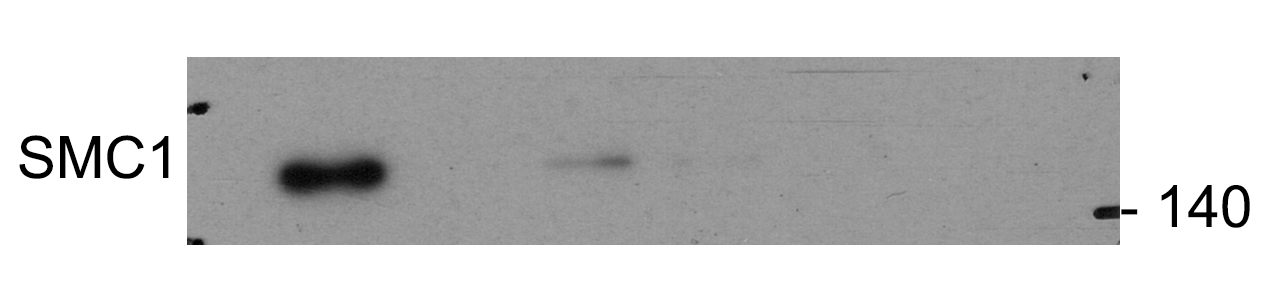

Supplement: Supplementary file 6 — Source data Fig. 2 [file 44318_2024_104_MOESM6_ESM.zip › Figure 2/2C/western SMC1.tif]

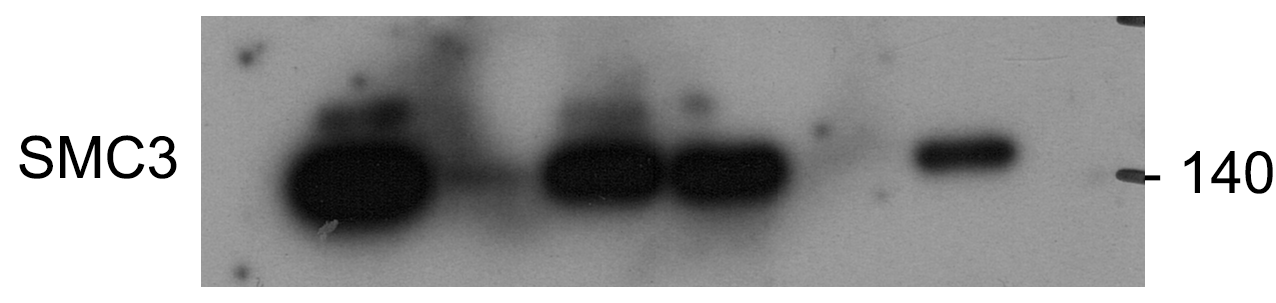

Supplement: Supplementary file 6 — Source data Fig. 2 [file 44318_2024_104_MOESM6_ESM.zip › Figure 2/2C/western SMC3.tif]

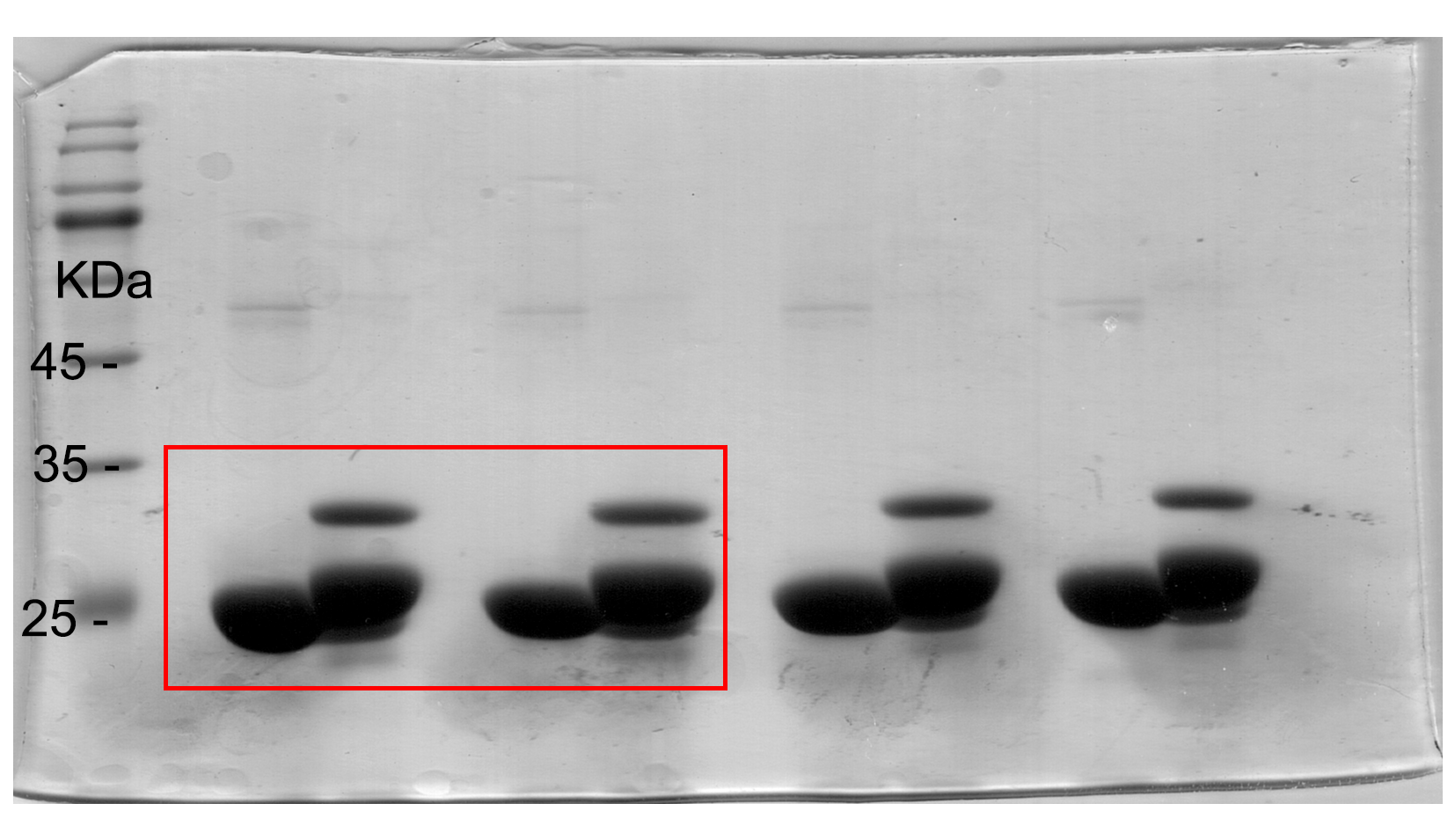

Supplement: Supplementary file 6 — Source data Fig. 2 [file 44318_2024_104_MOESM6_ESM.zip › Figure 2/2D/CBB.tif]

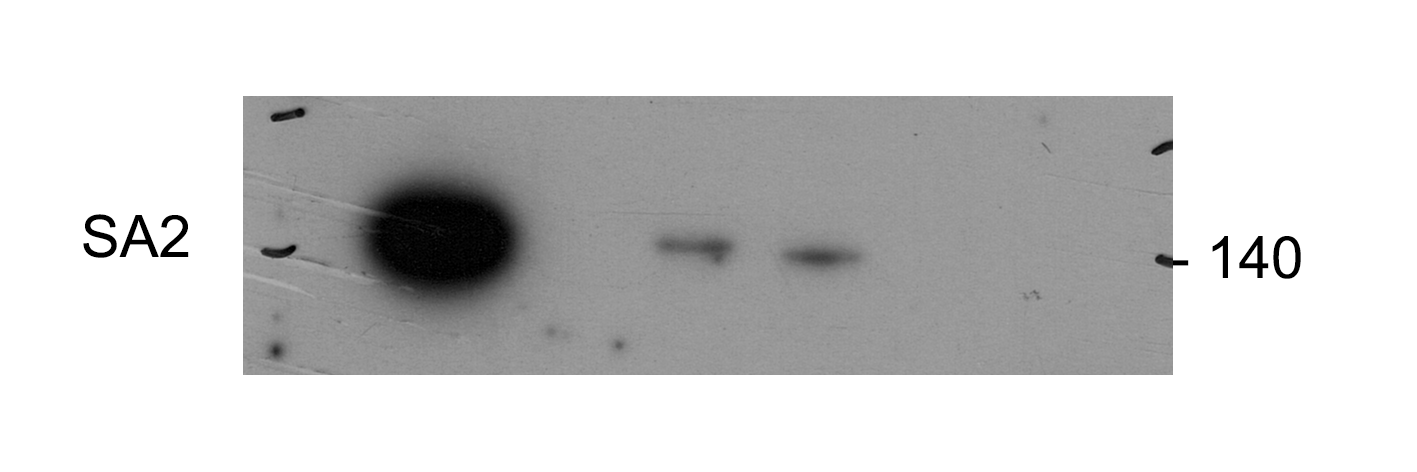

Supplement: Supplementary file 6 — Source data Fig. 2 [file 44318_2024_104_MOESM6_ESM.zip › Figure 2/2D/western SA2.tif]

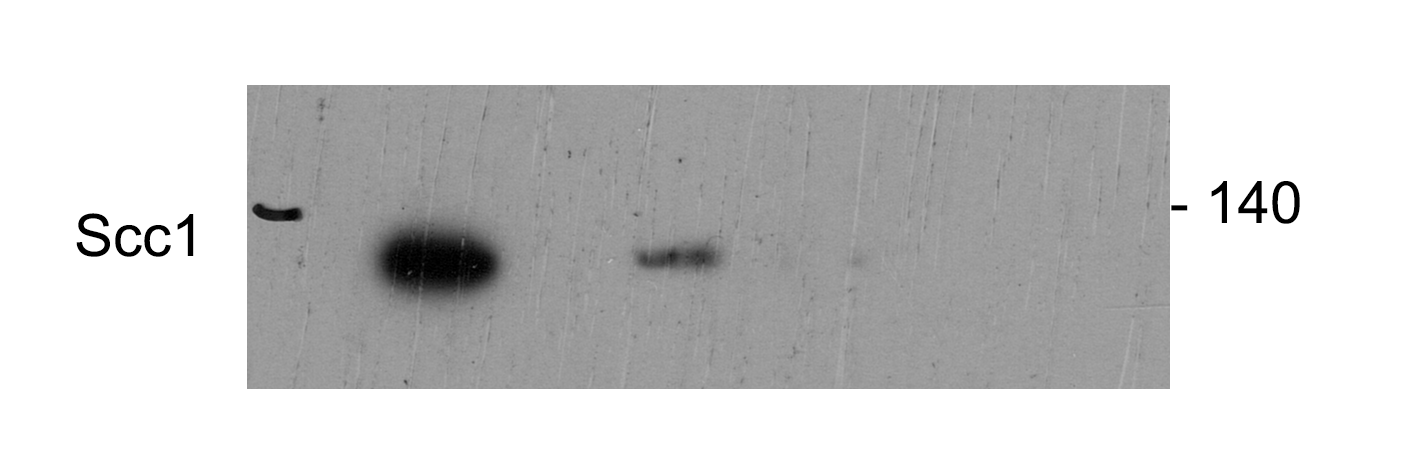

Supplement: Supplementary file 6 — Source data Fig. 2 [file 44318_2024_104_MOESM6_ESM.zip › Figure 2/2D/western Scc1.tif]

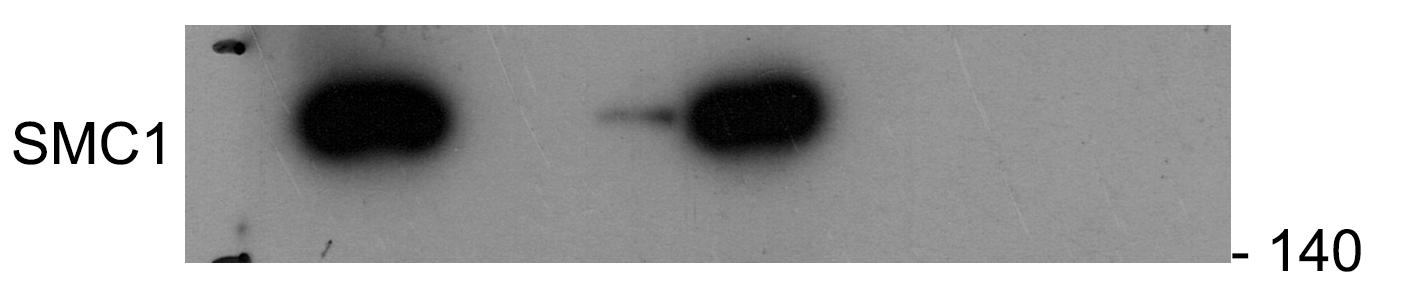

Supplement: Supplementary file 6 — Source data Fig. 2 [file 44318_2024_104_MOESM6_ESM.zip › Figure 2/2D/western SMC1.tif]

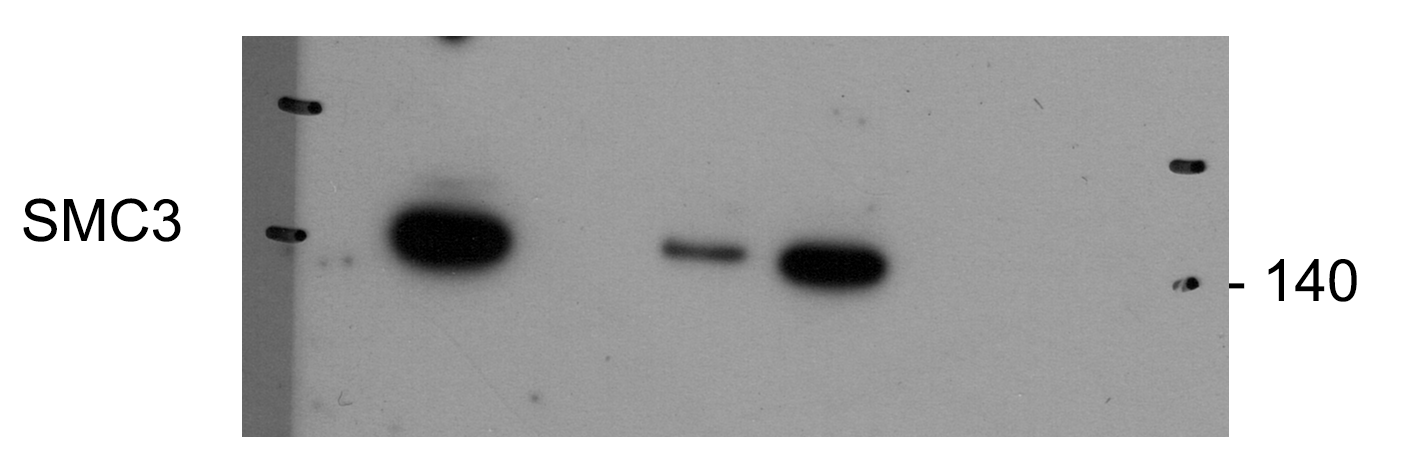

Supplement: Supplementary file 6 — Source data Fig. 2 [file 44318_2024_104_MOESM6_ESM.zip › Figure 2/2D/western SMC3.tif]

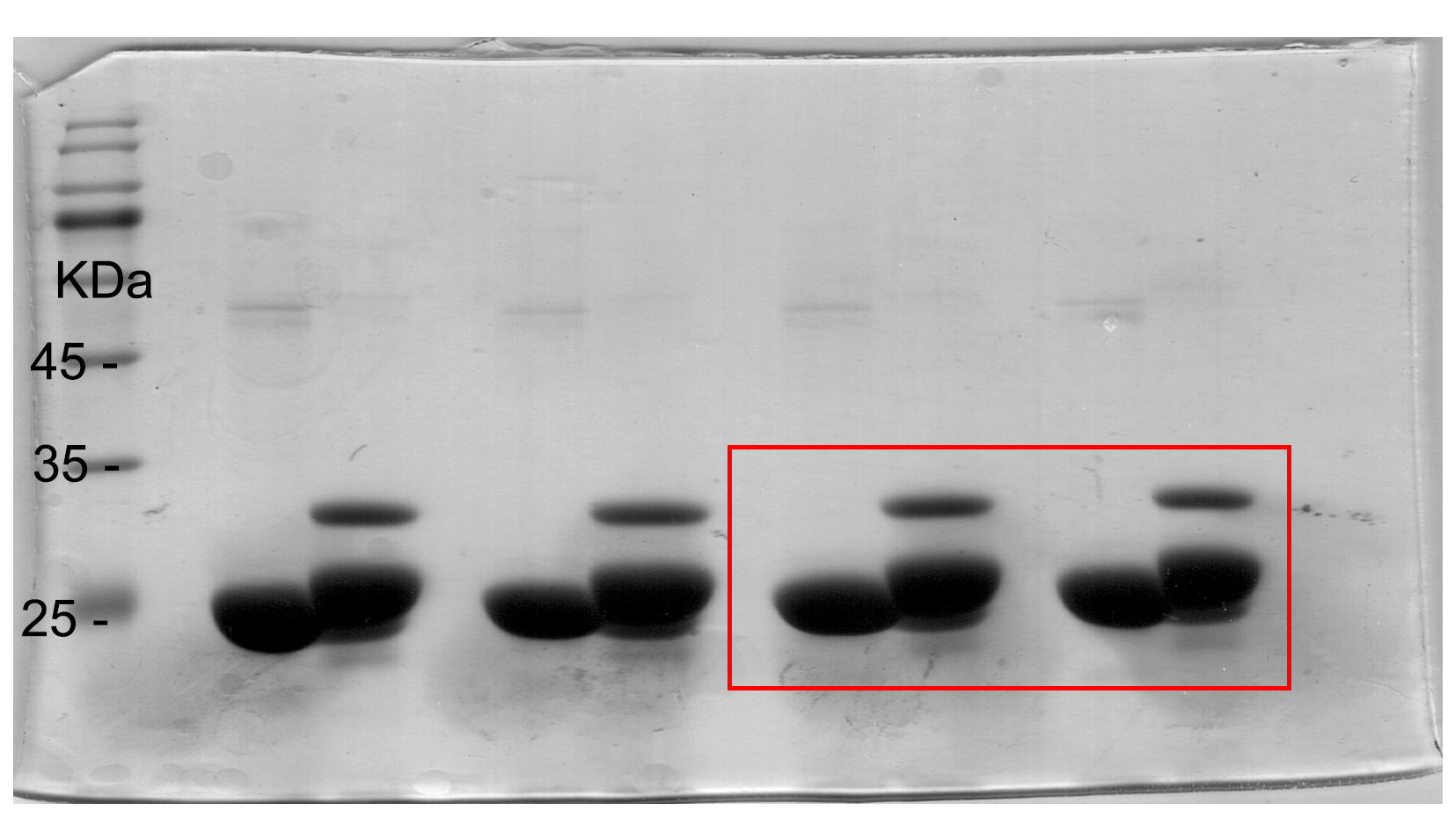

Supplement: Supplementary file 6 — Source data Fig. 2 [file 44318_2024_104_MOESM6_ESM.zip › Figure 2/2E/CBB.tif]

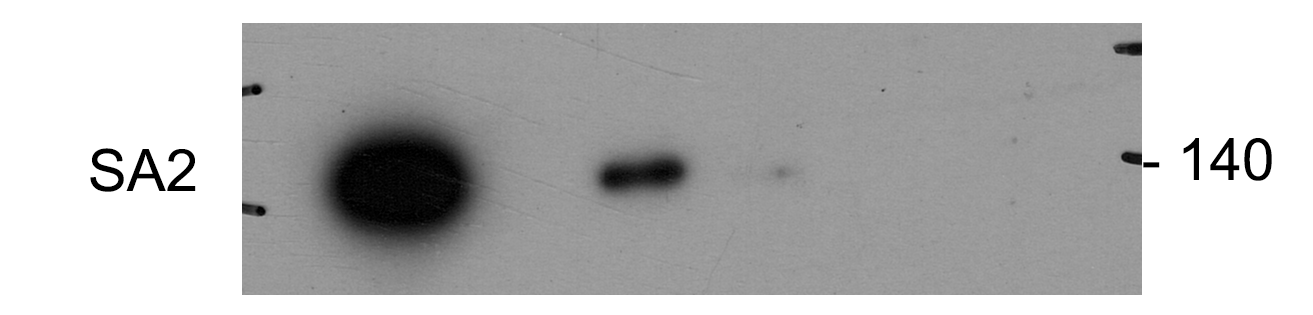

Supplement: Supplementary file 6 — Source data Fig. 2 [file 44318_2024_104_MOESM6_ESM.zip › Figure 2/2E/western SA2.tif]

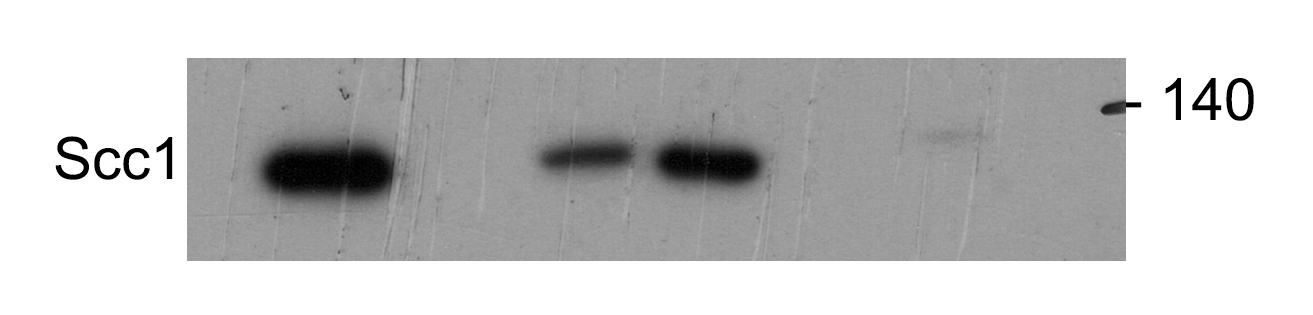

Supplement: Supplementary file 6 — Source data Fig. 2 [file 44318_2024_104_MOESM6_ESM.zip › Figure 2/2E/western Scc1.tif]

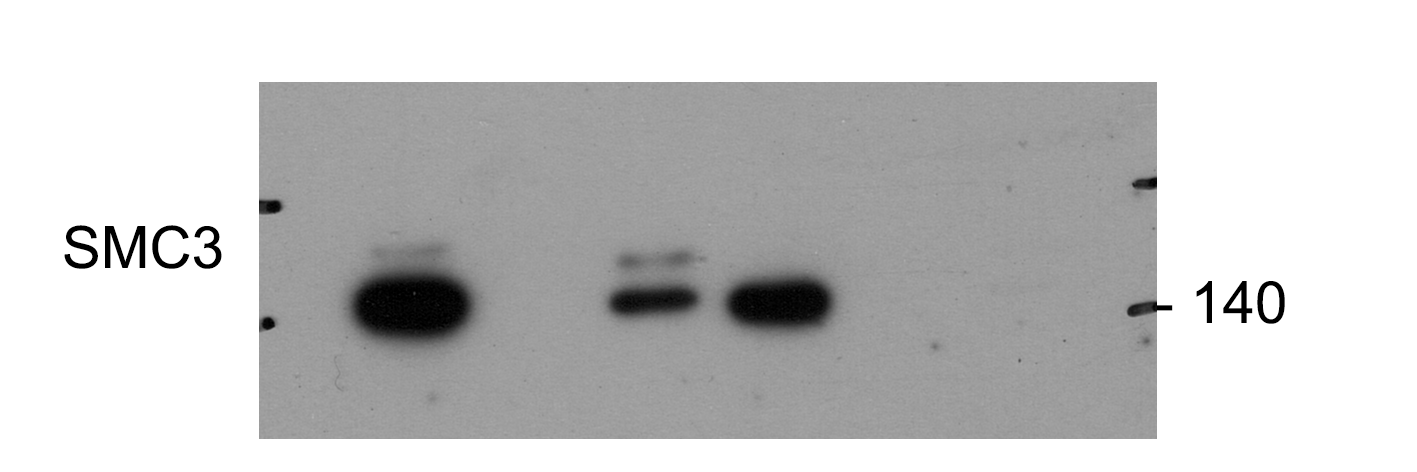

Supplement: Supplementary file 6 — Source data Fig. 2 [file 44318_2024_104_MOESM6_ESM.zip › Figure 2/2E/western SMC3.tif]

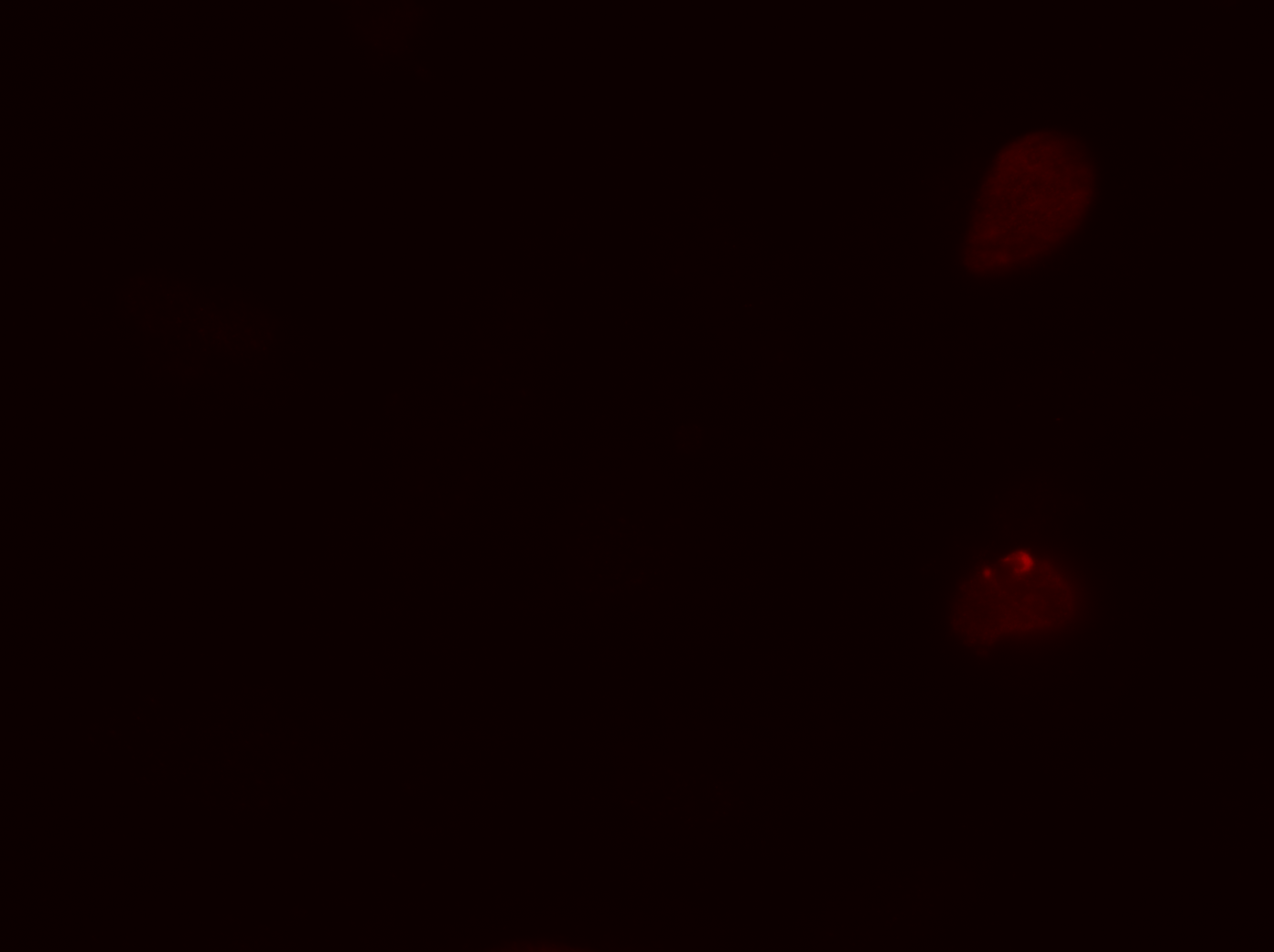

Supplement: Supplementary file 6 — Source data Fig. 2 [file 44318_2024_104_MOESM6_ESM.zip › Figure 2/2F/EGFP-LacI+Myc-SA2+SFB-CENP-U Anti-Myc.tif]

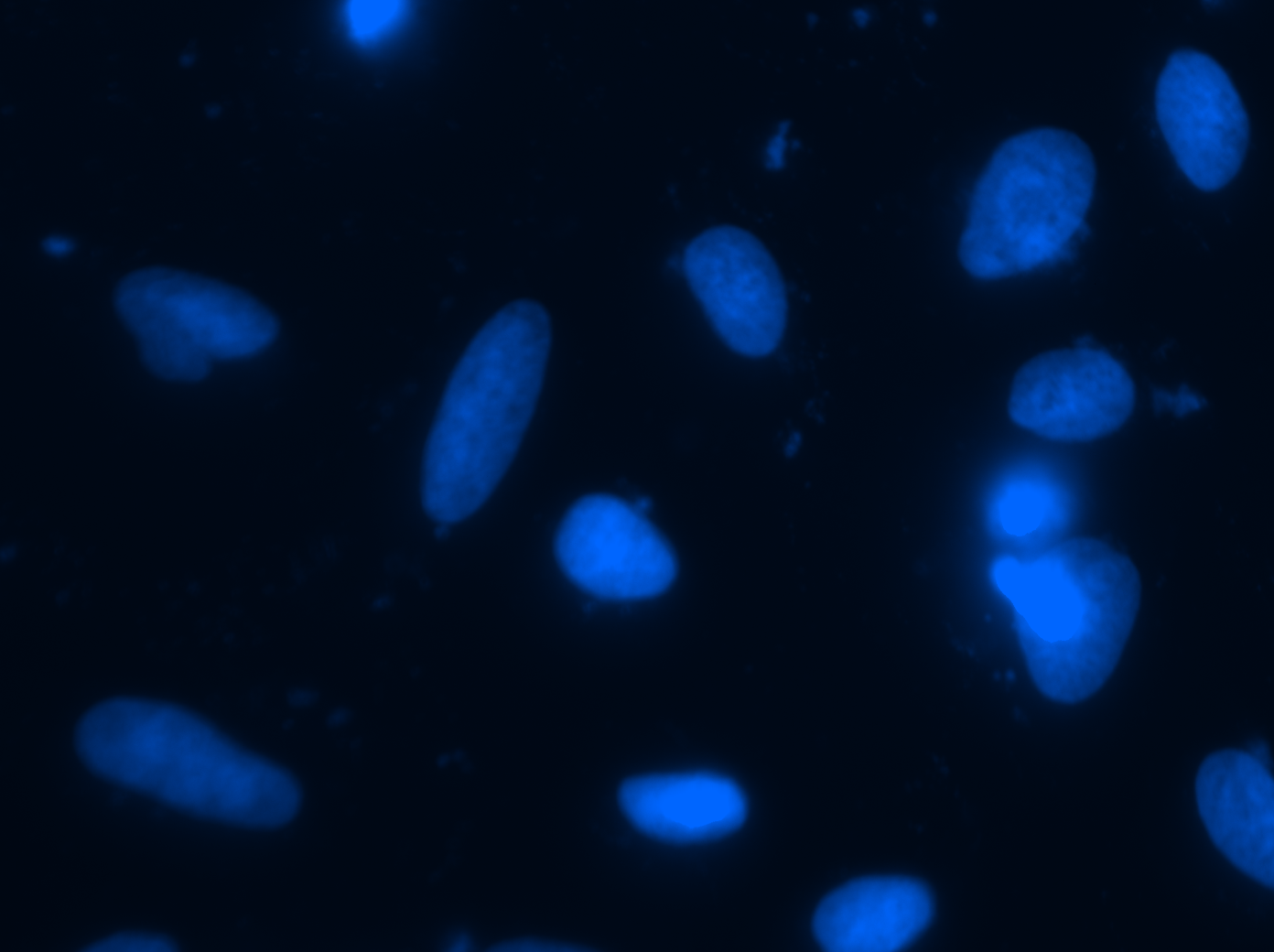

Supplement: Supplementary file 6 — Source data Fig. 2 [file 44318_2024_104_MOESM6_ESM.zip › Figure 2/2F/EGFP-LacI+Myc-SA2+SFB-CENP-U DNA.tif]

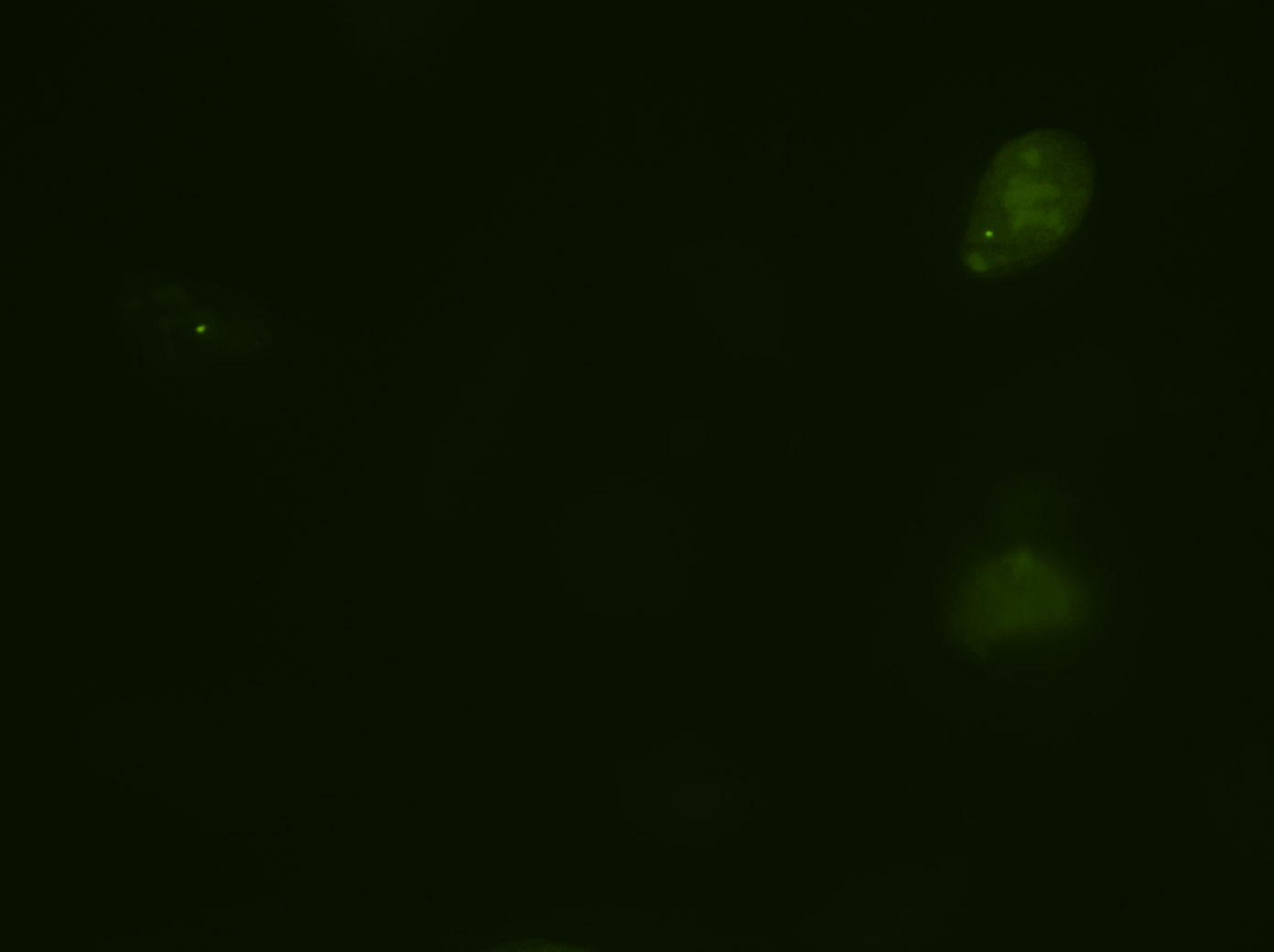

Supplement: Supplementary file 6 — Source data Fig. 2 [file 44318_2024_104_MOESM6_ESM.zip › Figure 2/2F/EGFP-LacI+Myc-SA2+SFB-CENP-U EGFP.tif]

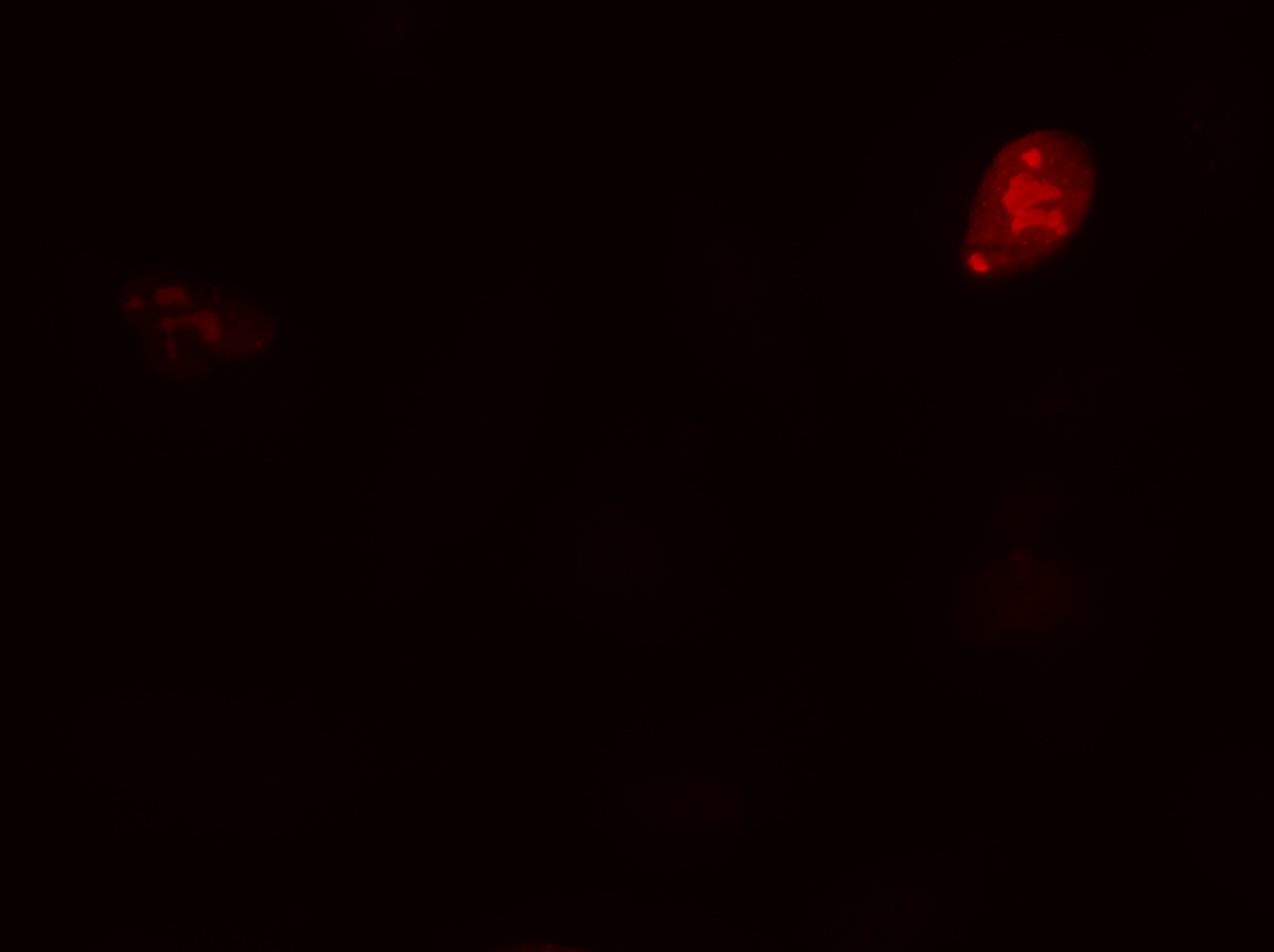

Supplement: Supplementary file 6 — Source data Fig. 2 [file 44318_2024_104_MOESM6_ESM.zip › Figure 2/2F/EGFP-LacI+Myc-SA2+SFB-CENP-U-DNA Anti-Flag.tif]

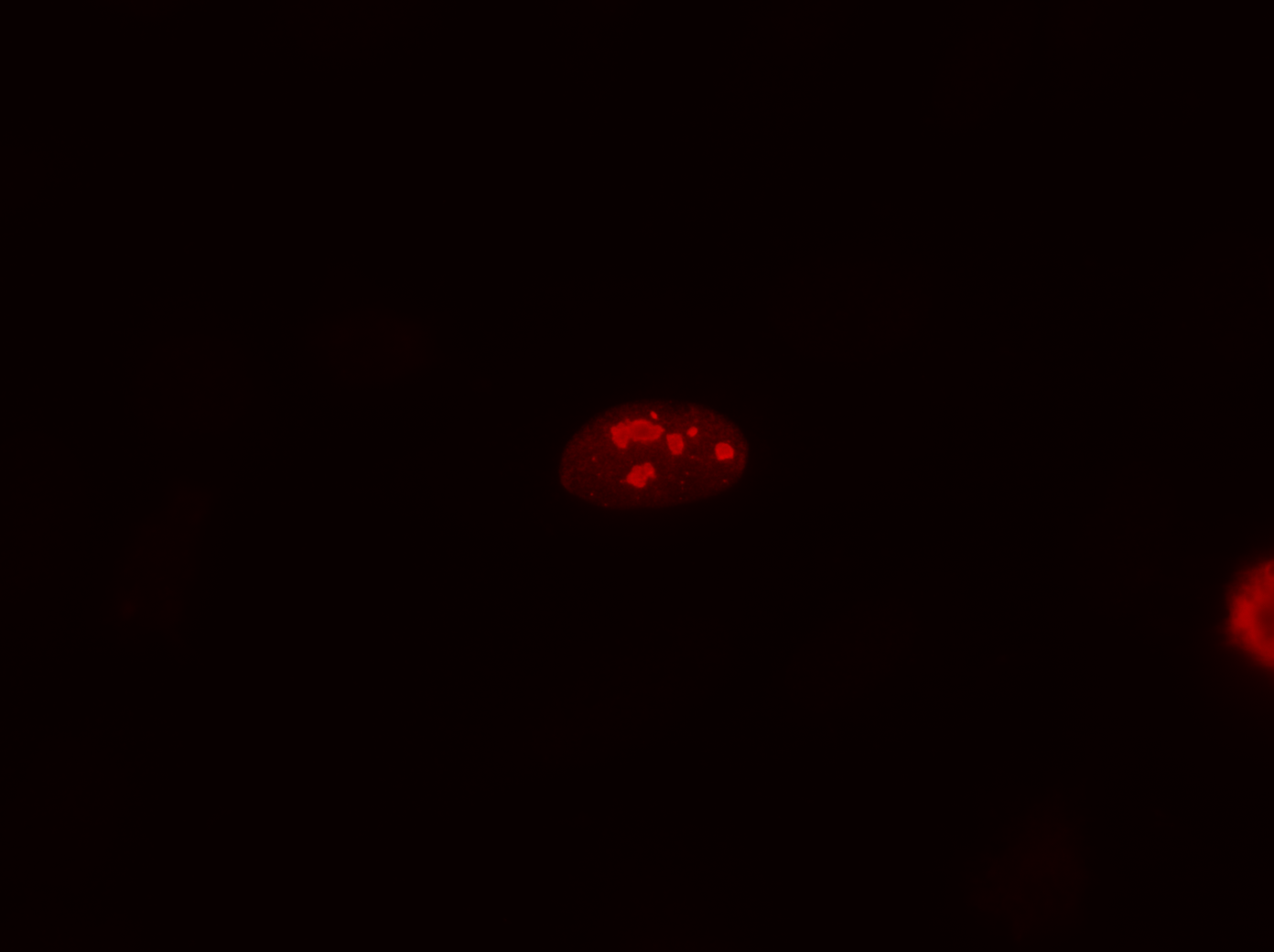

Supplement: Supplementary file 6 — Source data Fig. 2 [file 44318_2024_104_MOESM6_ESM.zip › Figure 2/2F/EGFP-LacI-Scc1+Myc-SA2+SFB-CENP-U Anti-Flag.tif]

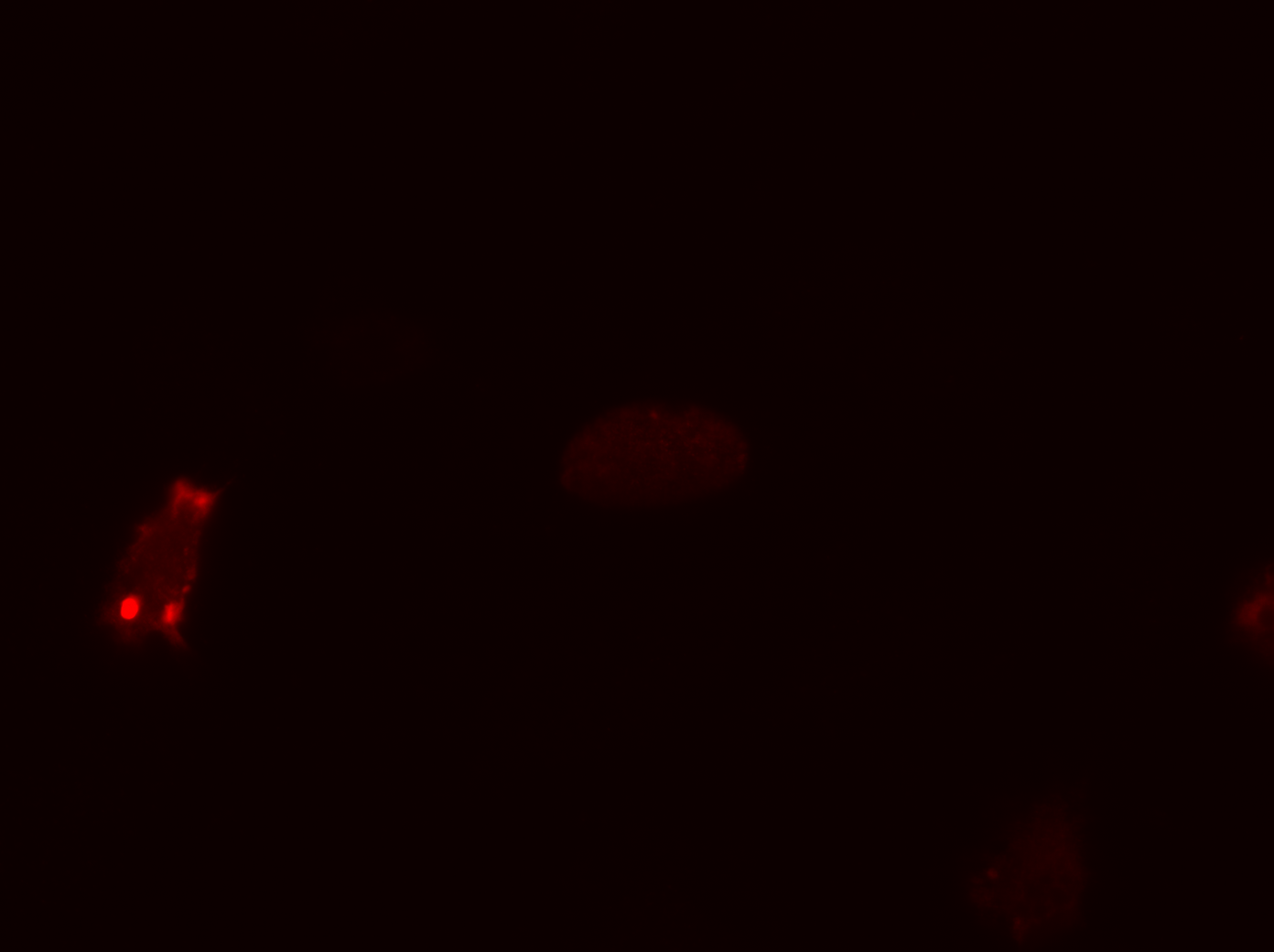

Supplement: Supplementary file 6 — Source data Fig. 2 [file 44318_2024_104_MOESM6_ESM.zip › Figure 2/2F/EGFP-LacI-Scc1+Myc-SA2+SFB-CENP-U Anti-Myc.tif]

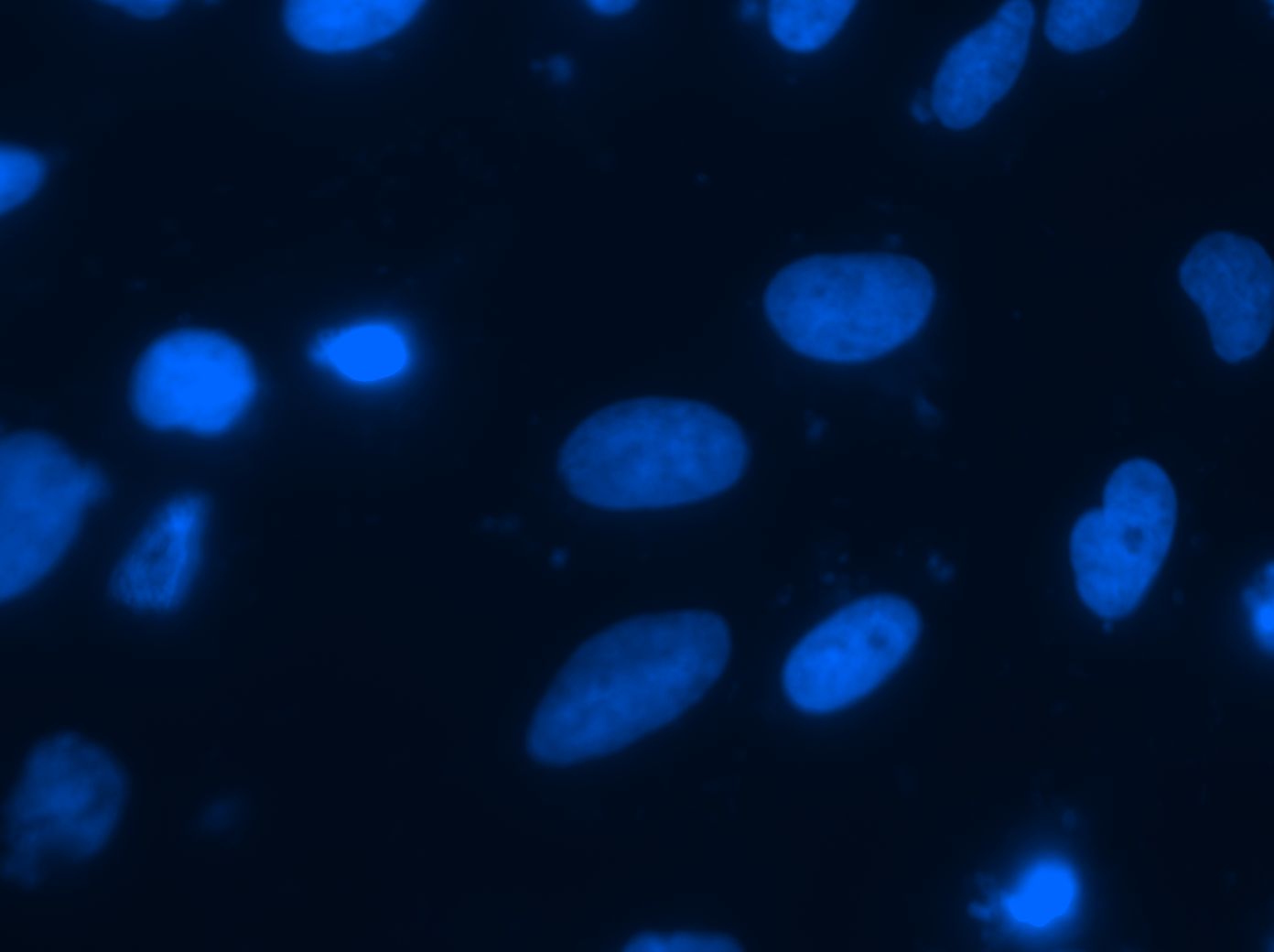

Supplement: Supplementary file 6 — Source data Fig. 2 [file 44318_2024_104_MOESM6_ESM.zip › Figure 2/2F/EGFP-LacI-Scc1+Myc-SA2+SFB-CENP-U DNA.tif]

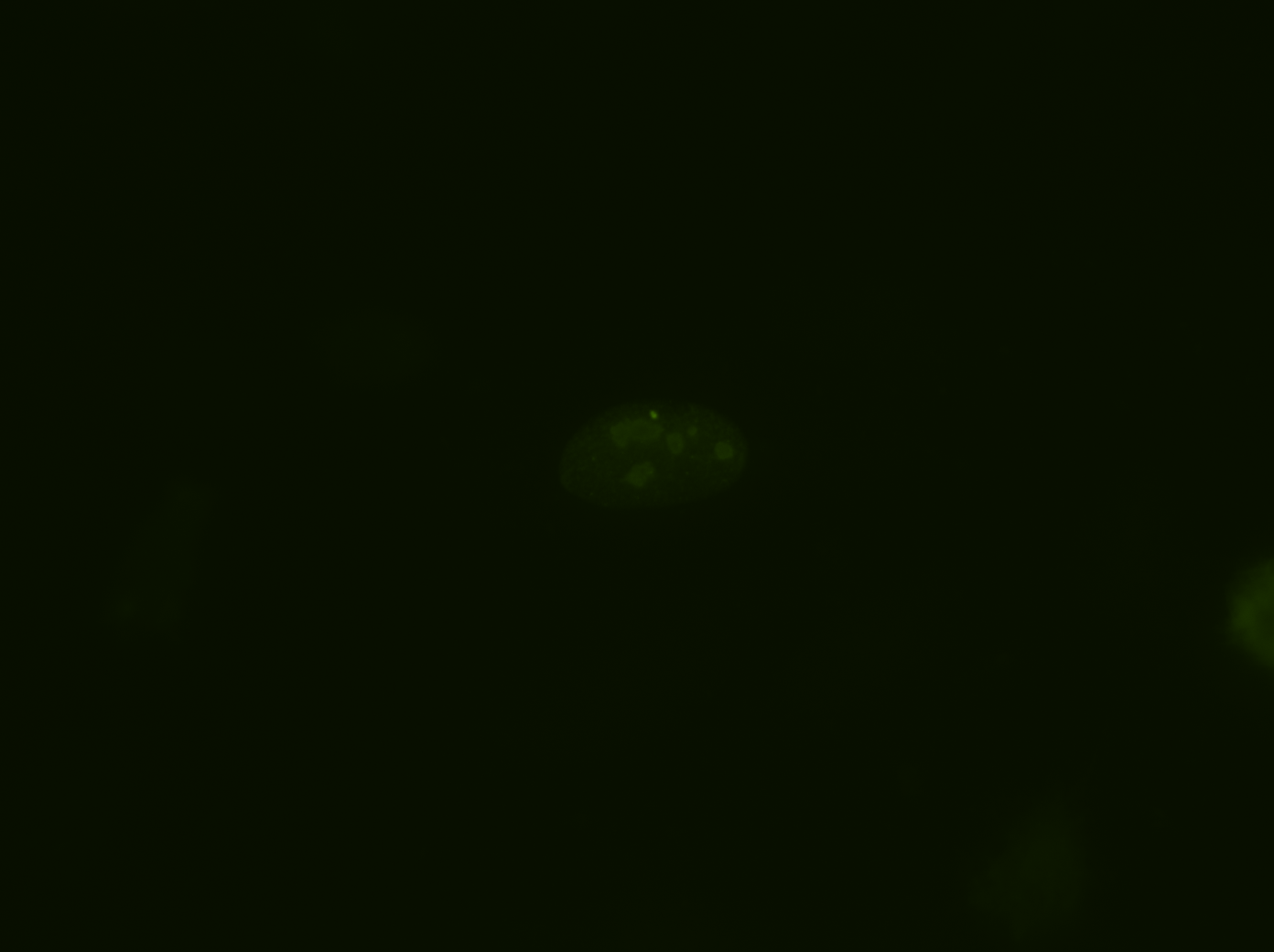

Supplement: Supplementary file 6 — Source data Fig. 2 [file 44318_2024_104_MOESM6_ESM.zip › Figure 2/2F/EGFP-LacI-Scc1+Myc-SA2+SFB-CENP-U EGFP.tif]

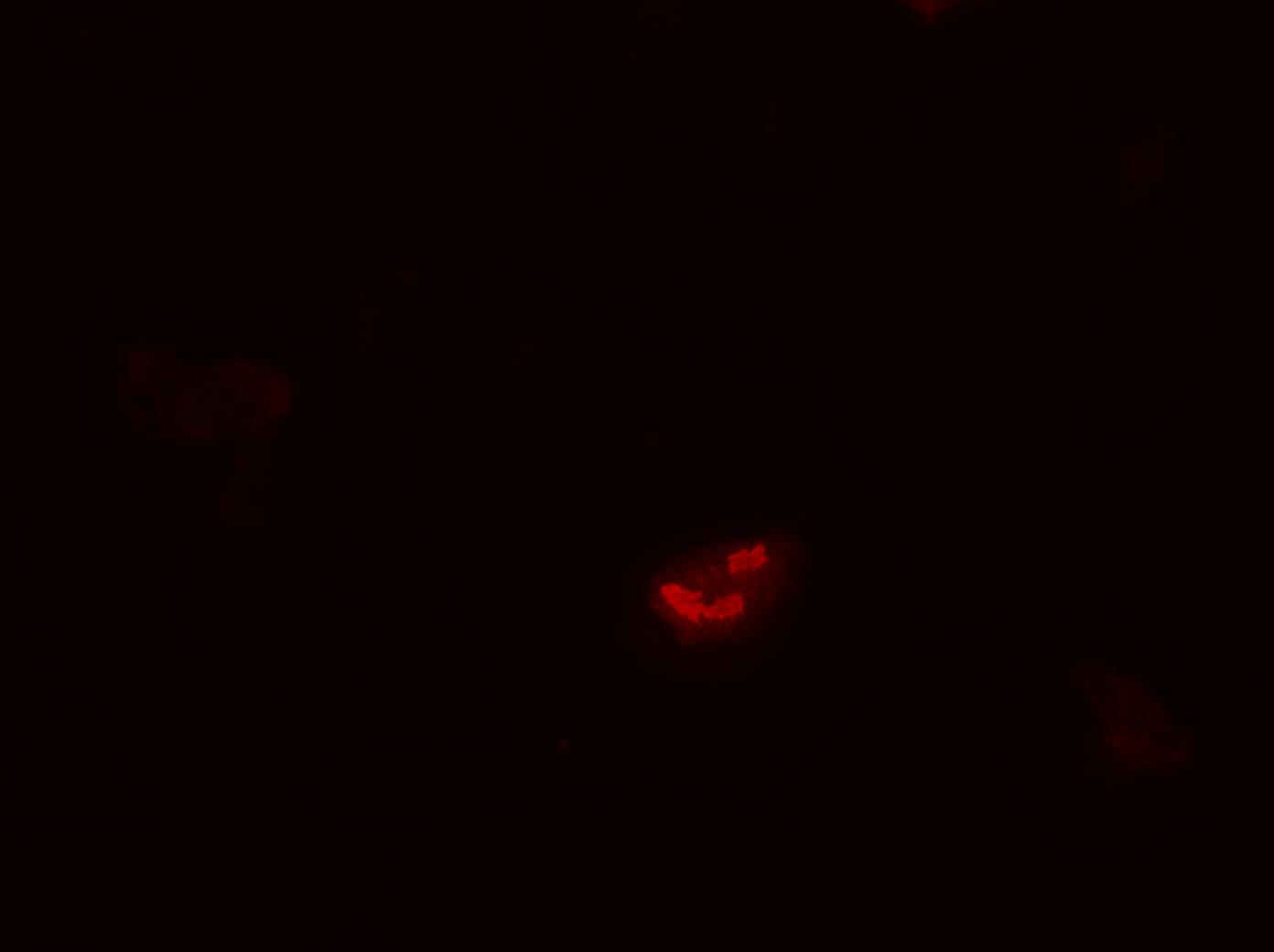

Supplement: Supplementary file 6 — Source data Fig. 2 [file 44318_2024_104_MOESM6_ESM.zip › Figure 2/2F/EGFP-LacI-Scc1+SFB-CENP-U Anti-Flag.tif]

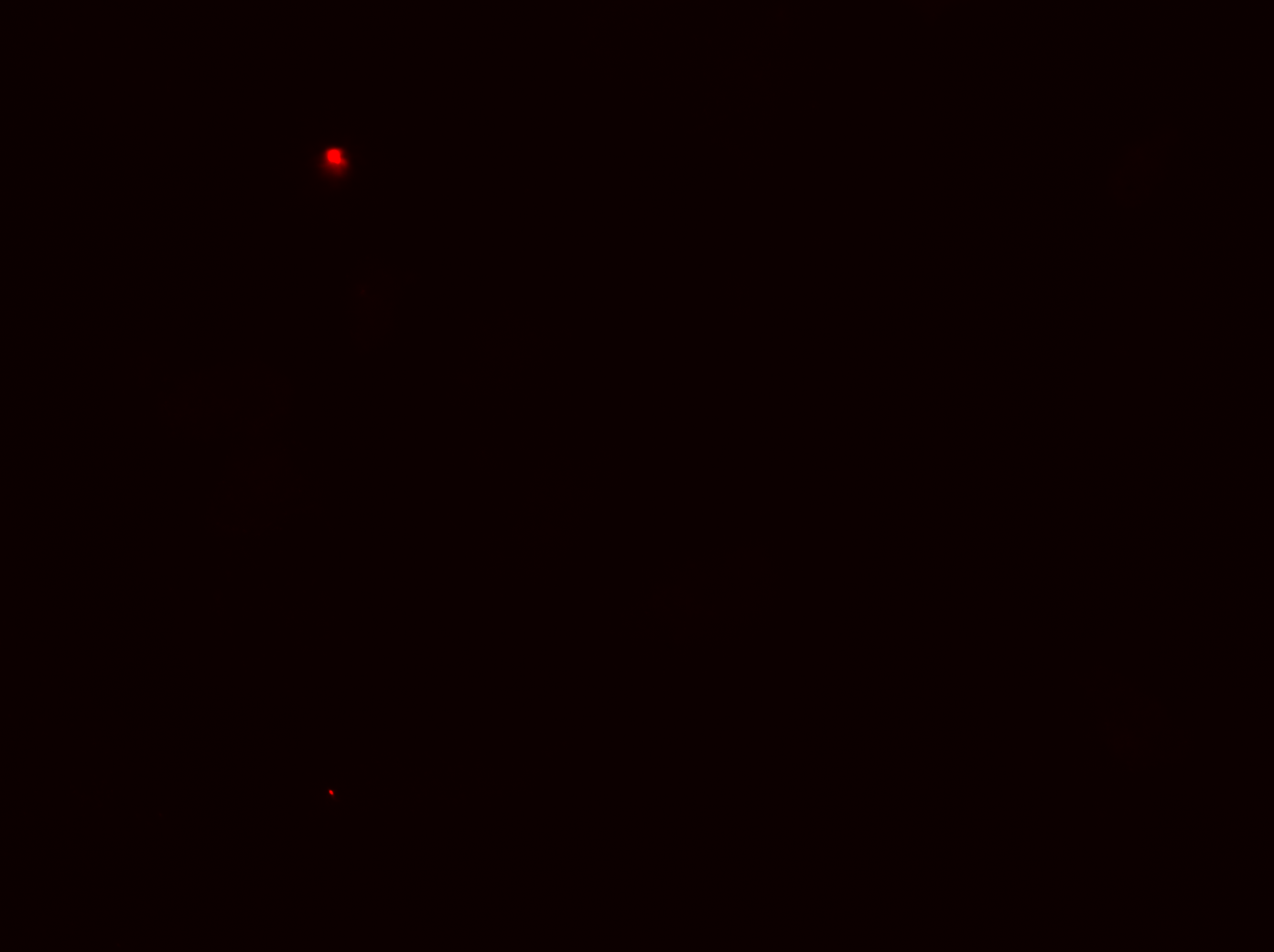

Supplement: Supplementary file 6 — Source data Fig. 2 [file 44318_2024_104_MOESM6_ESM.zip › Figure 2/2F/EGFP-LacI-Scc1+SFB-CENP-U Anti-Myc.tif]

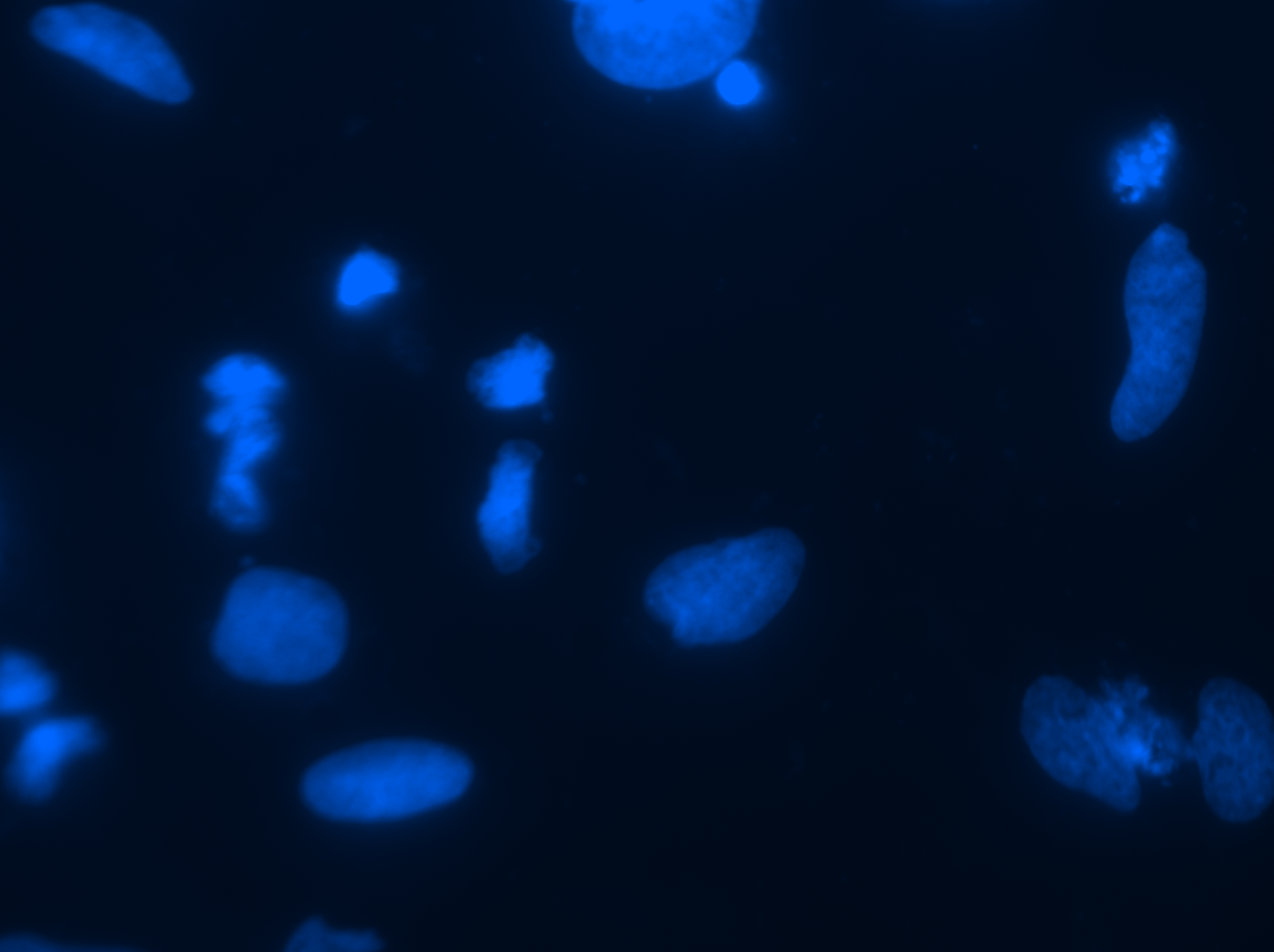

Supplement: Supplementary file 6 — Source data Fig. 2 [file 44318_2024_104_MOESM6_ESM.zip › Figure 2/2F/EGFP-LacI-Scc1+SFB-CENP-U DNA.tif]

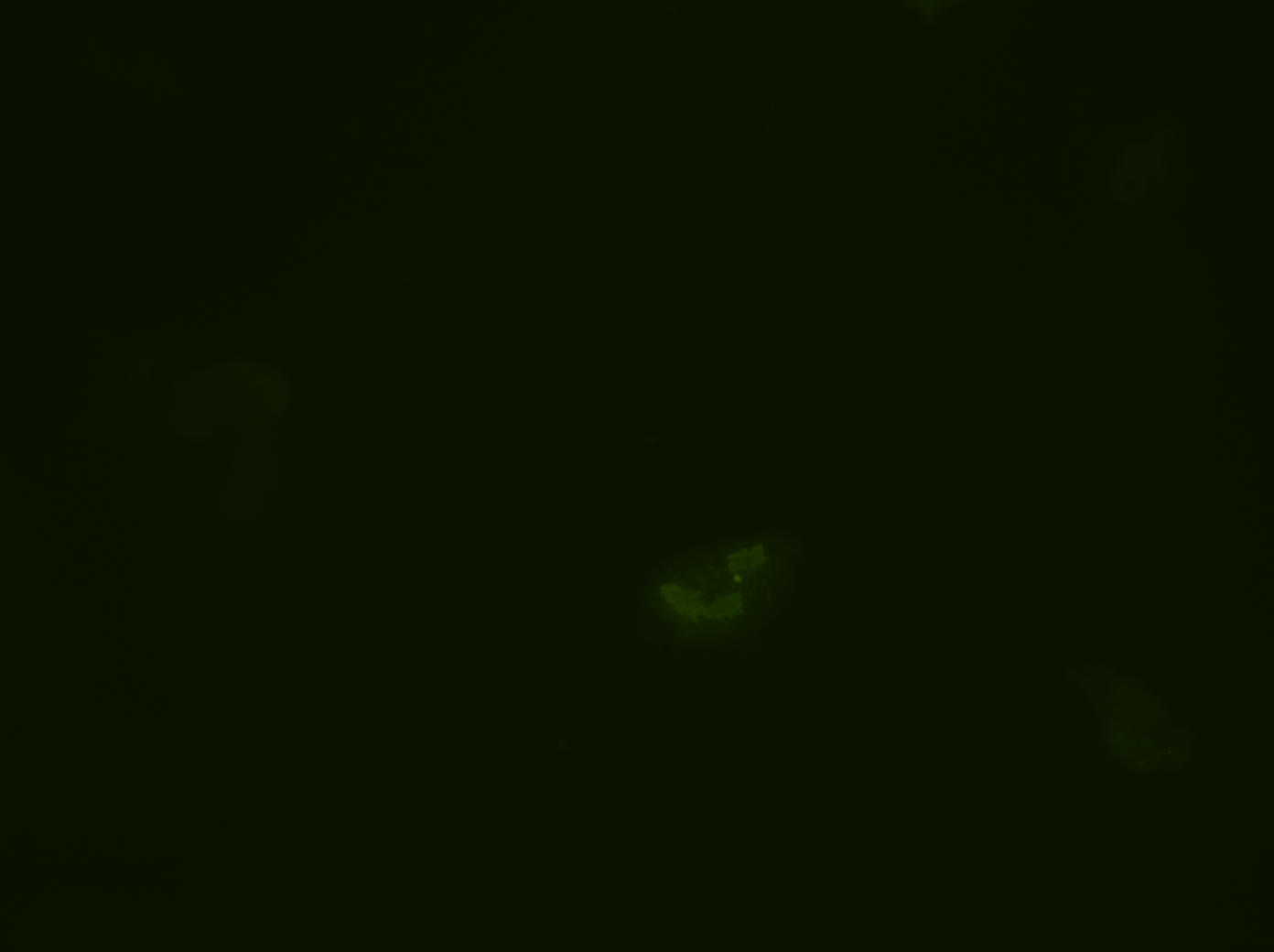

Supplement: Supplementary file 6 — Source data Fig. 2 [file 44318_2024_104_MOESM6_ESM.zip › Figure 2/2F/EGFP-LacI-Scc1+SFB-CENP-U EGFP.tif]

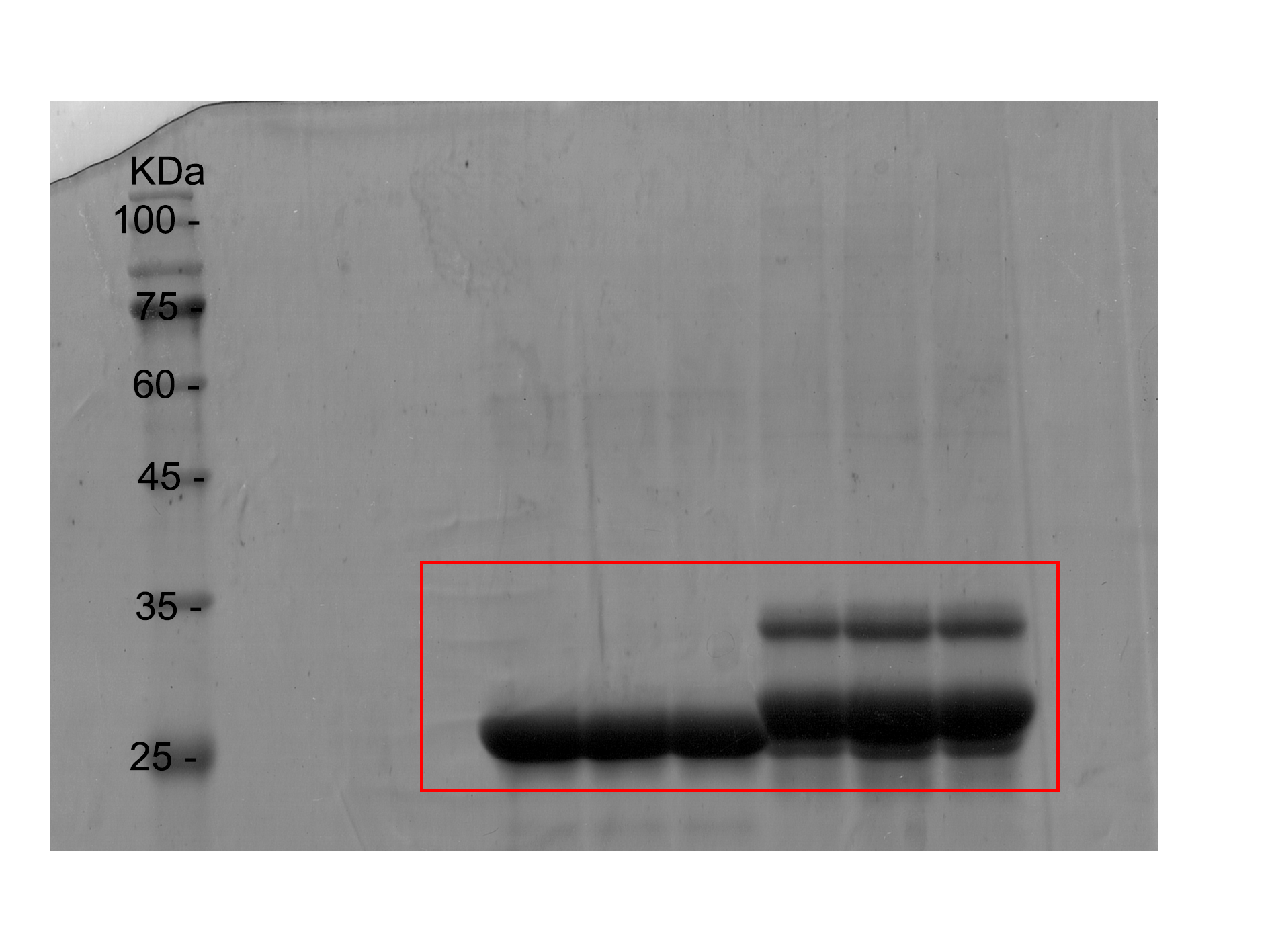

Supplement: Supplementary file 6 — Source data Fig. 2 [file 44318_2024_104_MOESM6_ESM.zip › Figure 2/2H/CBB.tif]

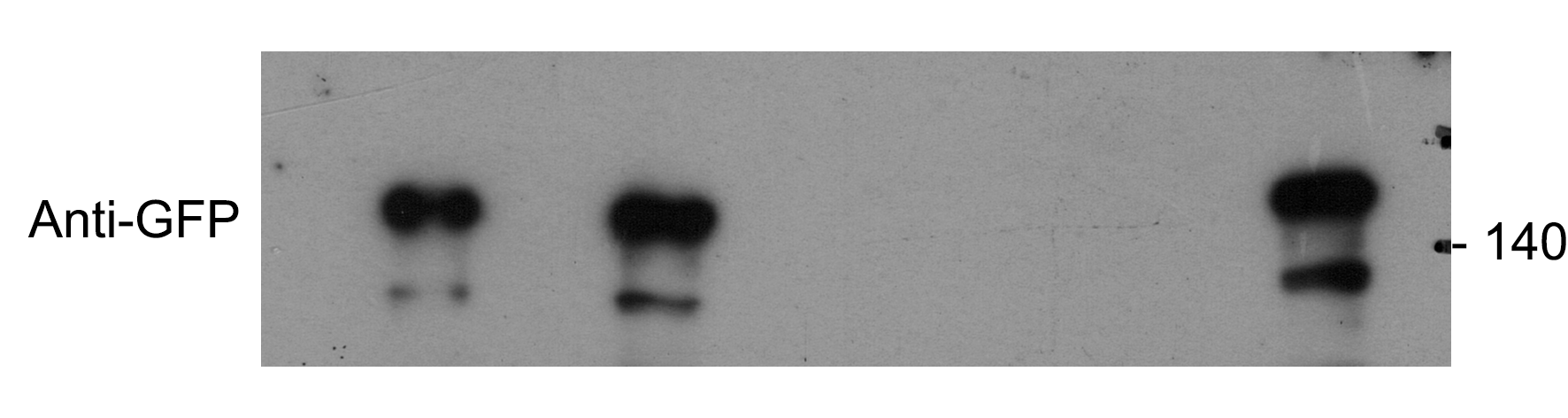

Supplement: Supplementary file 6 — Source data Fig. 2 [file 44318_2024_104_MOESM6_ESM.zip › Figure 2/2H/western GFP.tif]

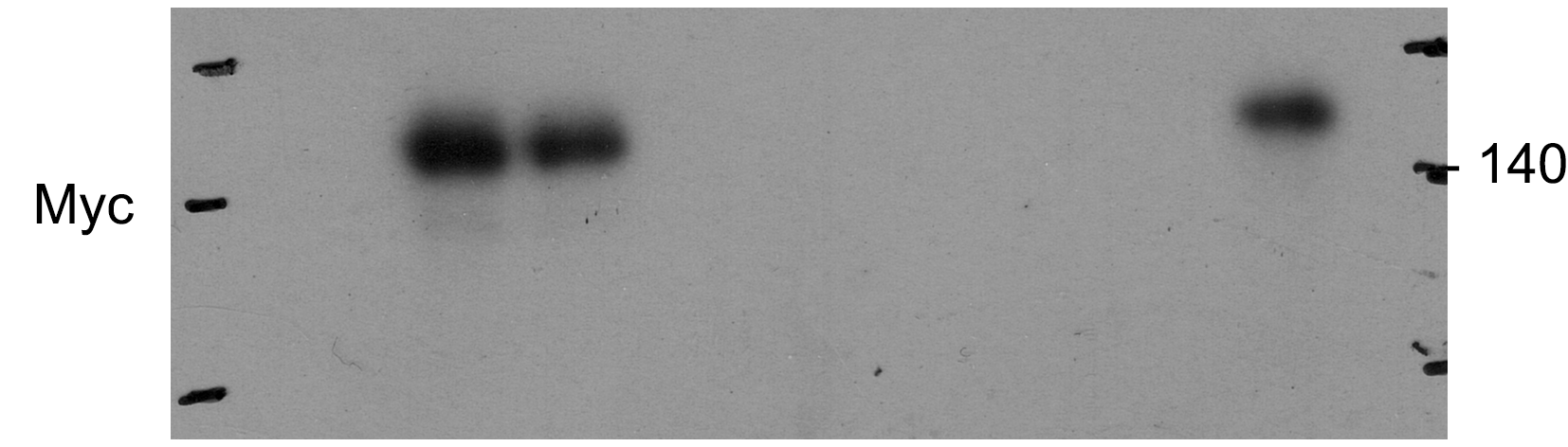

Supplement: Supplementary file 6 — Source data Fig. 2 [file 44318_2024_104_MOESM6_ESM.zip › Figure 2/2H/western Myc.tif]

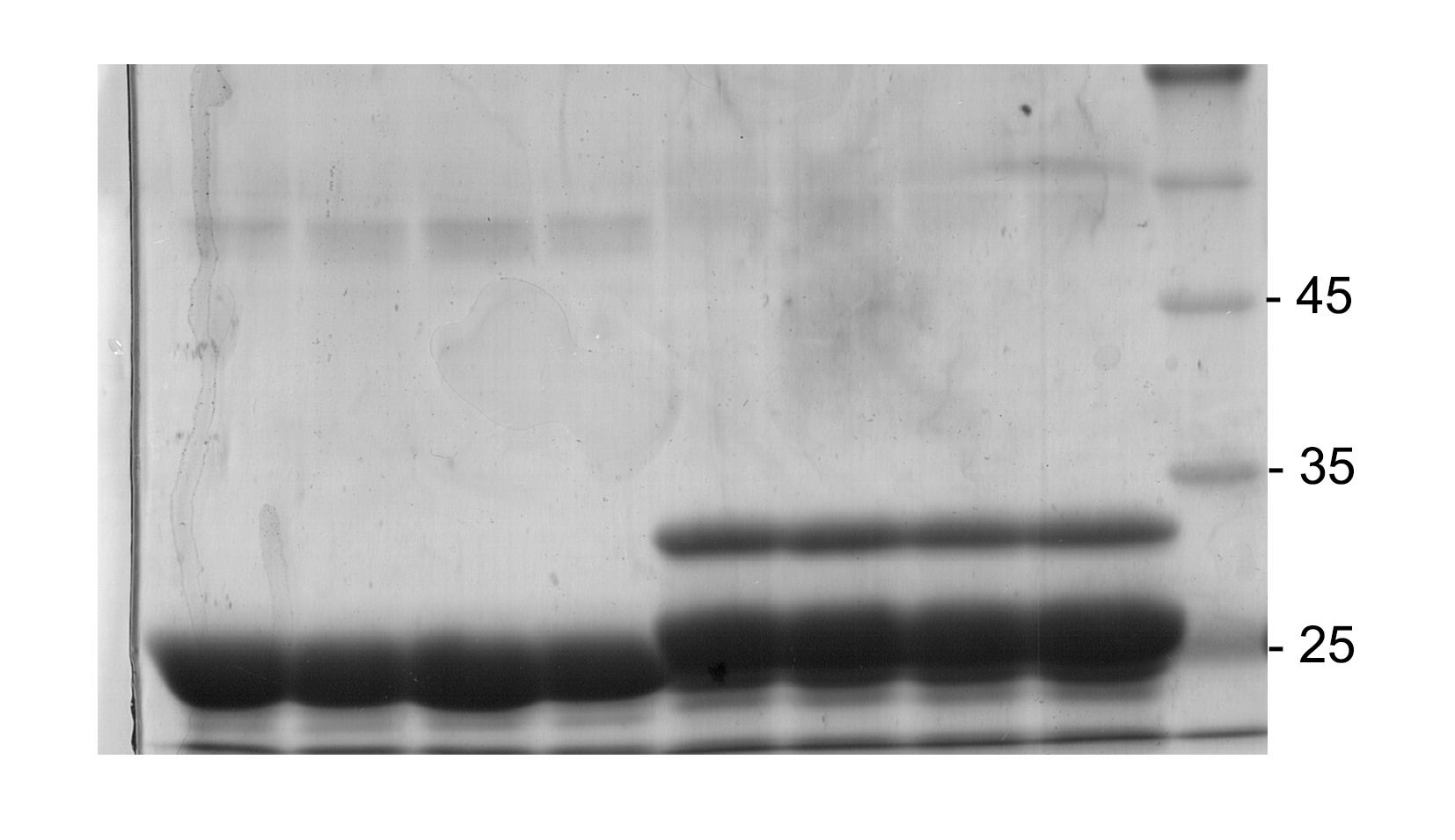

Supplement: Supplementary file 6 — Source data Fig. 2 [file 44318_2024_104_MOESM6_ESM.zip › Figure 2/2I/CBB.tif]

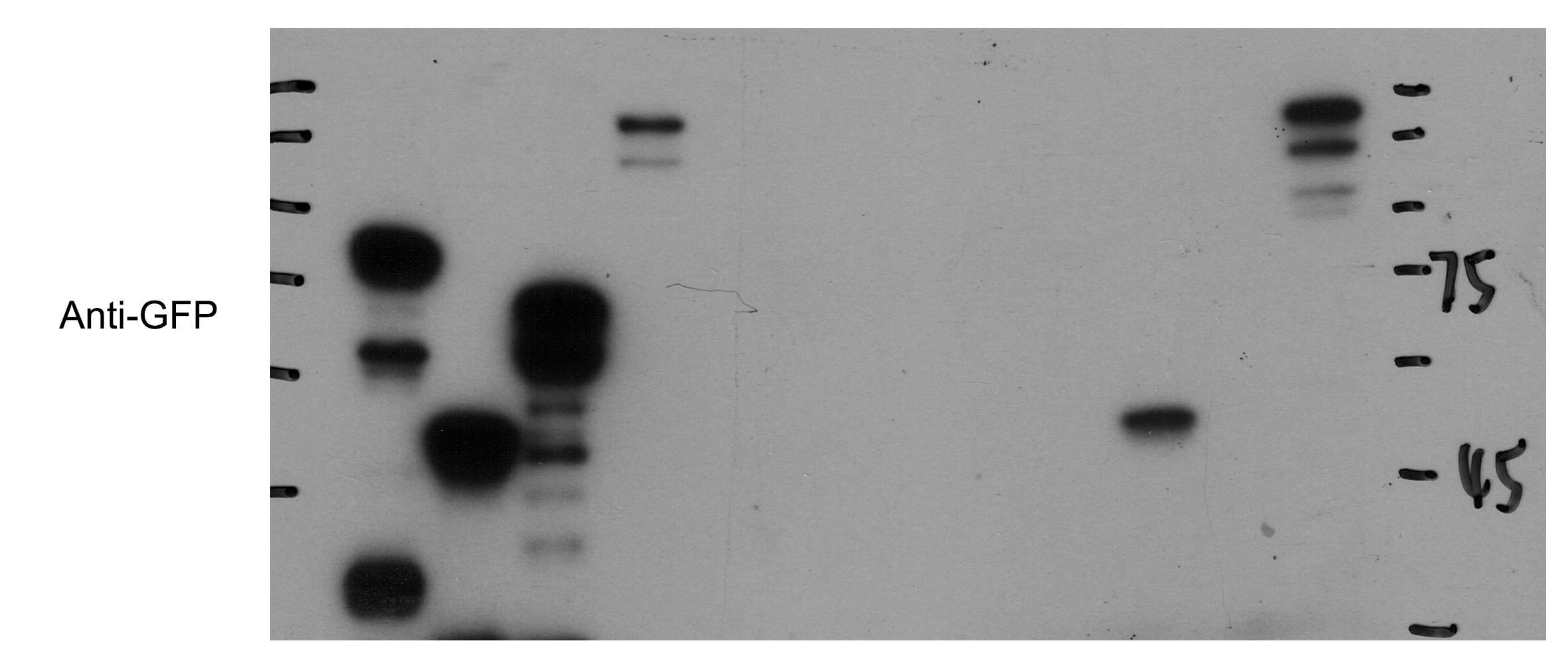

Supplement: Supplementary file 6 — Source data Fig. 2 [file 44318_2024_104_MOESM6_ESM.zip › Figure 2/2I/western GFP.tif]

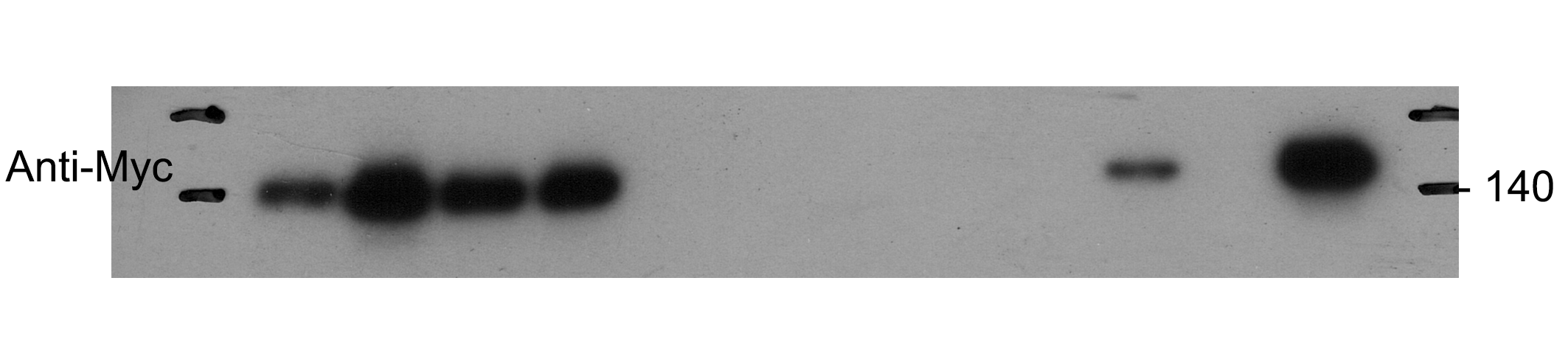

Supplement: Supplementary file 6 — Source data Fig. 2 [file 44318_2024_104_MOESM6_ESM.zip › Figure 2/2I/western Myc.tif]

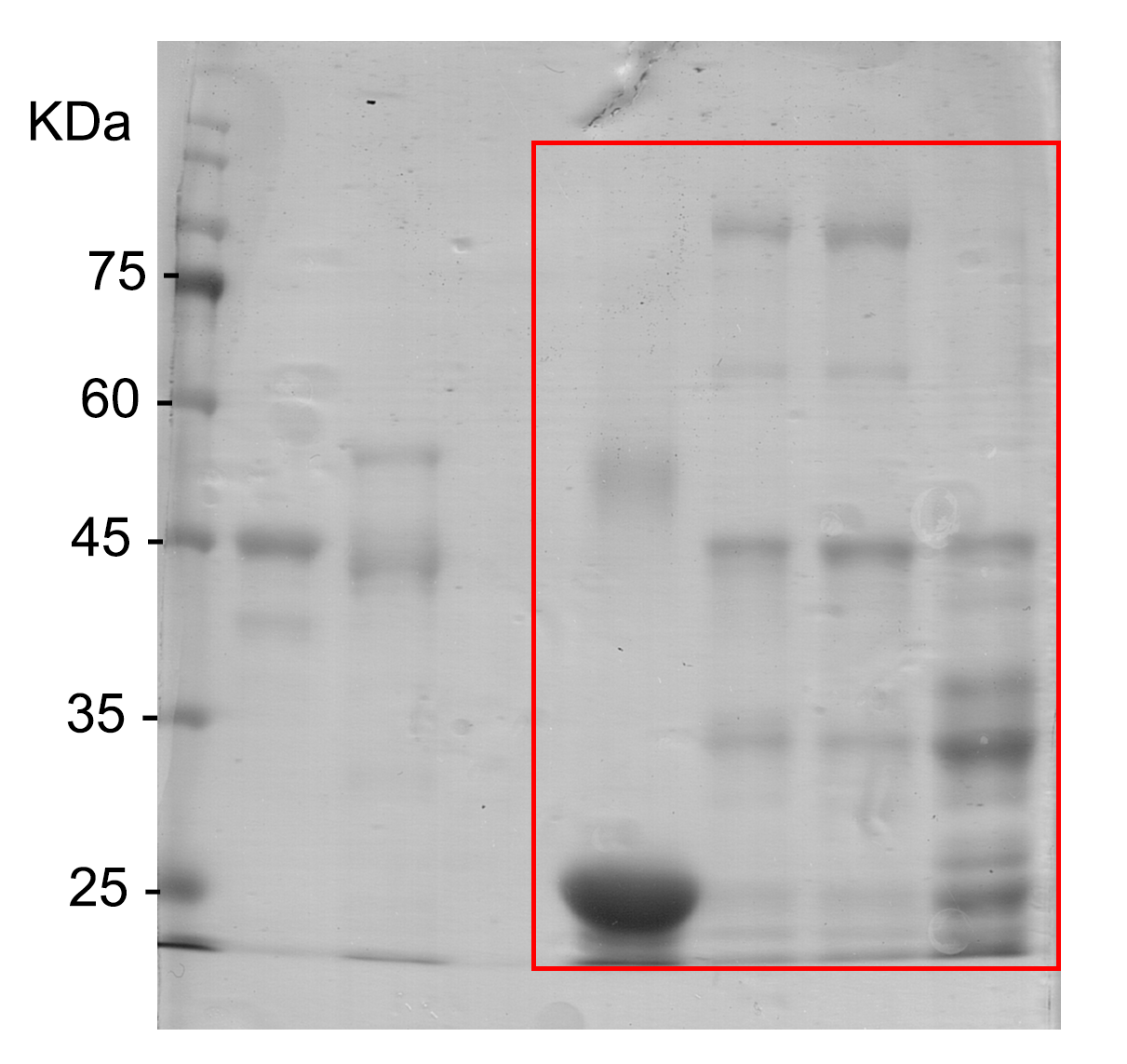

Supplement: Supplementary file 6 — Source data Fig. 2 [file 44318_2024_104_MOESM6_ESM.zip › Figure 2/2J/CBB.tif]

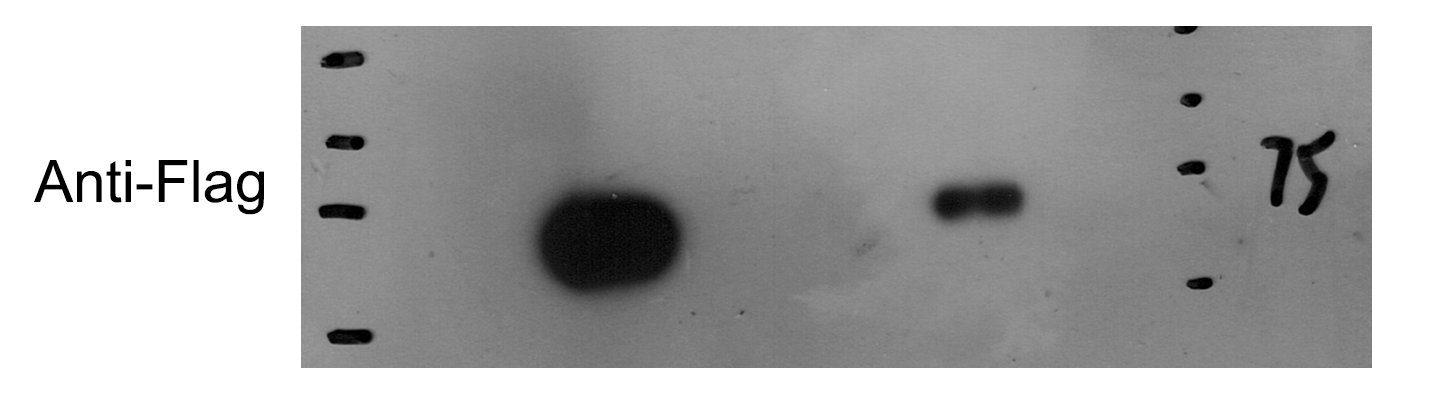

Supplement: Supplementary file 6 — Source data Fig. 2 [file 44318_2024_104_MOESM6_ESM.zip › Figure 2/2J/western Flag.tif]

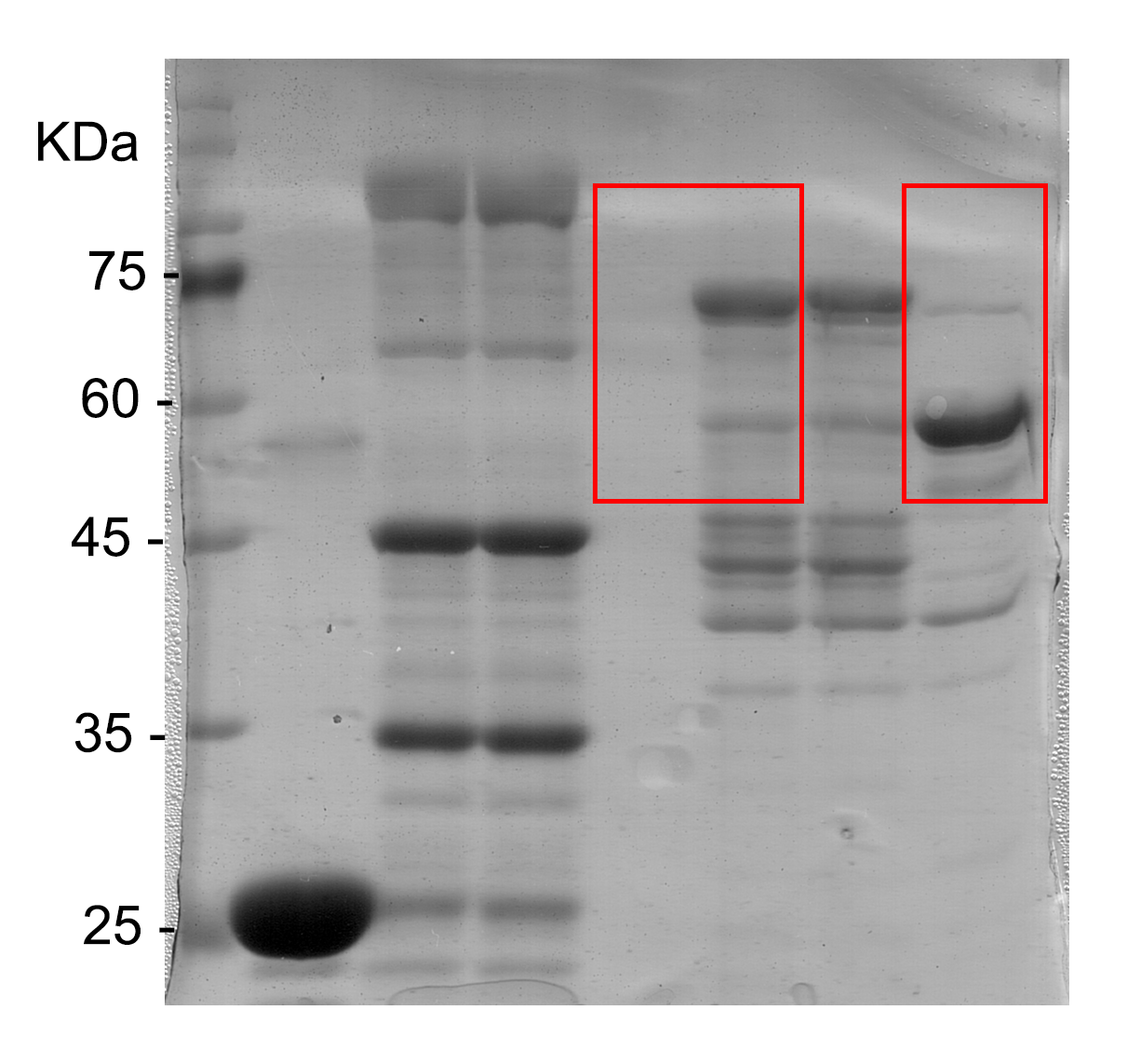

Supplement: Supplementary file 6 — Source data Fig. 2 [file 44318_2024_104_MOESM6_ESM.zip › Figure 2/2K/CBB.tif]

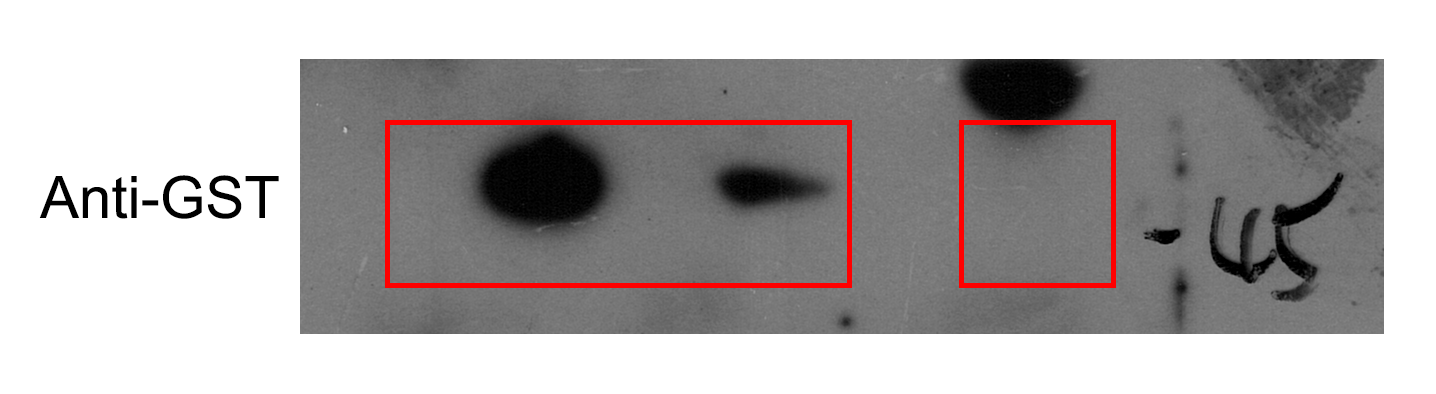

Supplement: Supplementary file 6 — Source data Fig. 2 [file 44318_2024_104_MOESM6_ESM.zip › Figure 2/2K/western GST.tif]

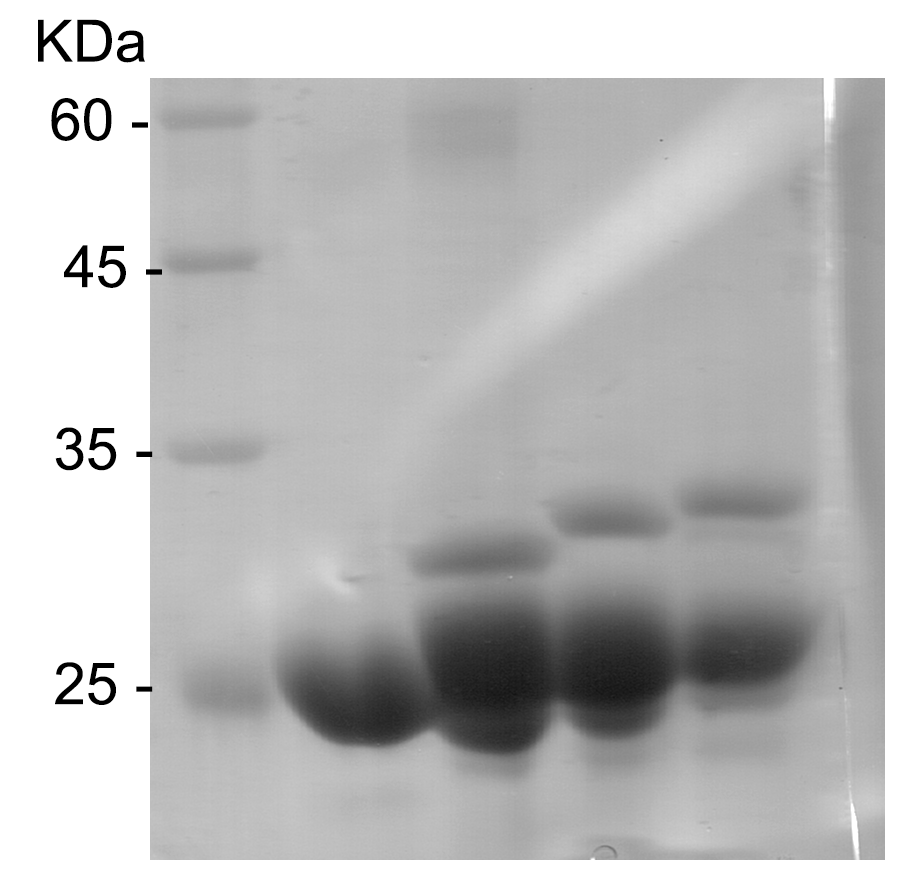

Supplement: Supplementary file 7 — Source data Fig. 3 [file 44318_2024_104_MOESM7_ESM.zip › Figure 3/3A/CBB.tif]

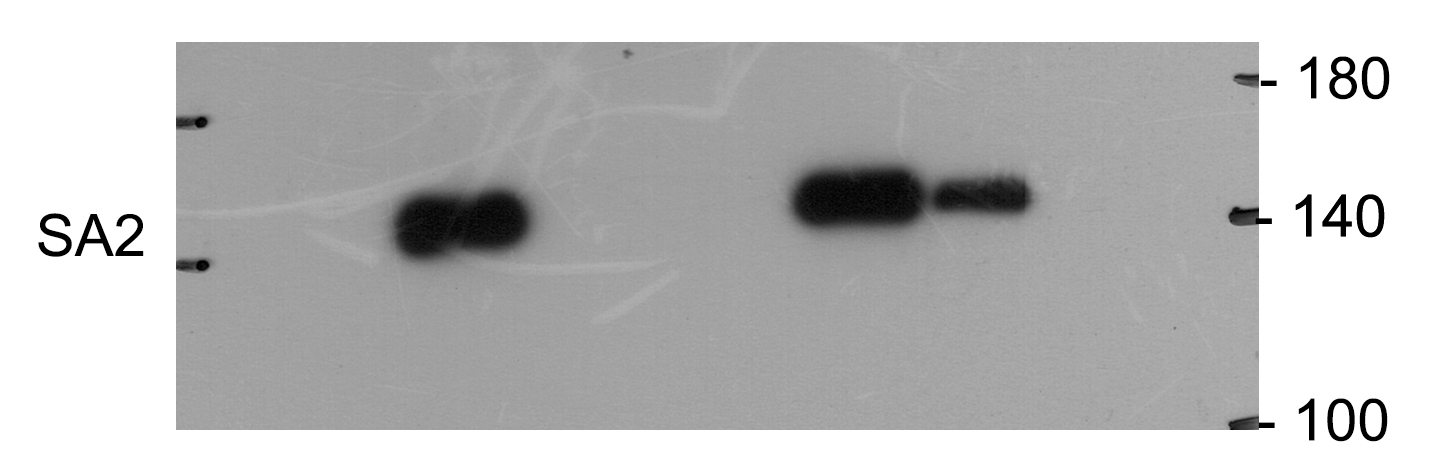

Supplement: Supplementary file 7 — Source data Fig. 3 [file 44318_2024_104_MOESM7_ESM.zip › Figure 3/3A/western SA2.tif]

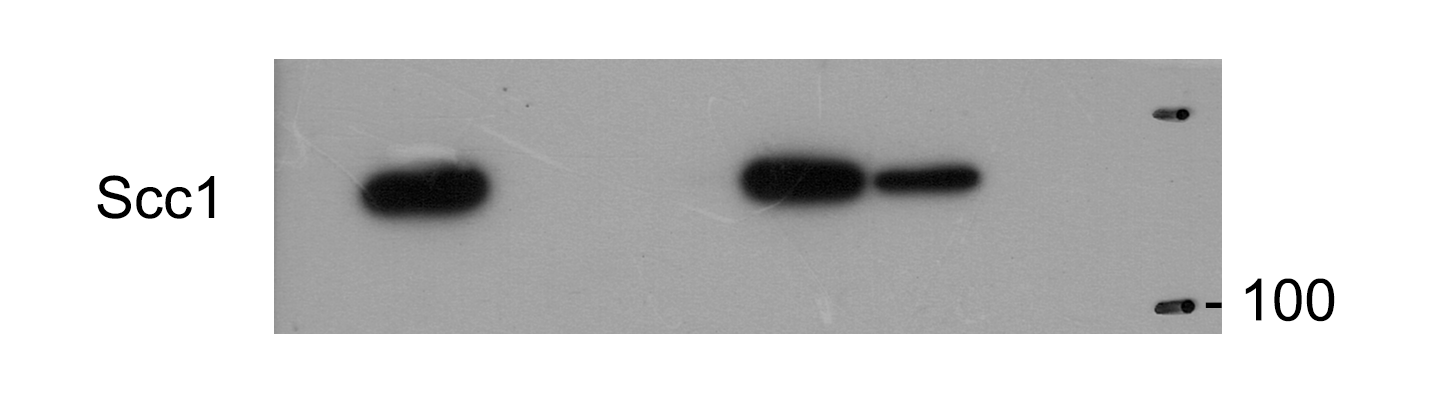

Supplement: Supplementary file 7 — Source data Fig. 3 [file 44318_2024_104_MOESM7_ESM.zip › Figure 3/3A/western Scc1.tif]

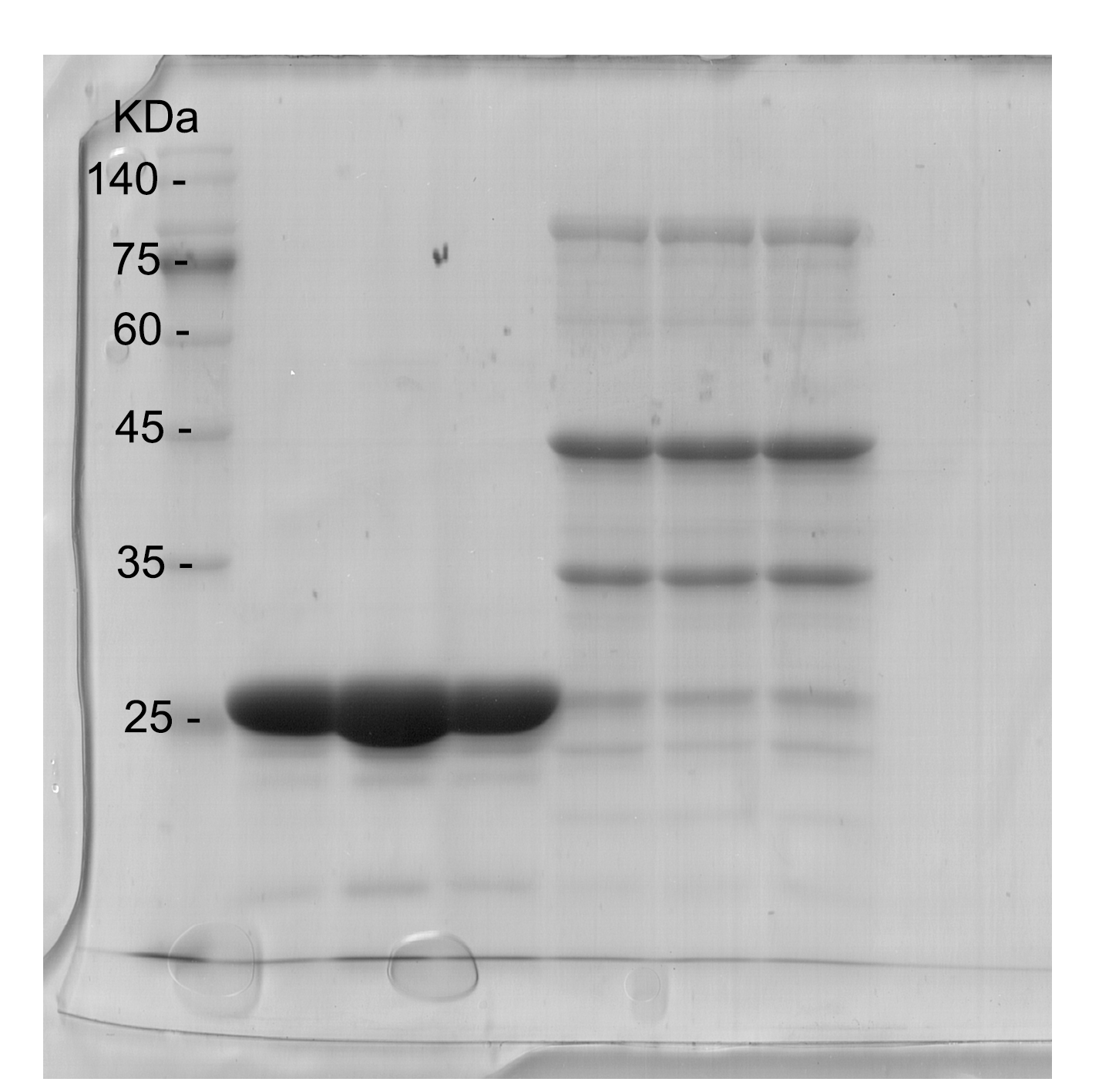

Supplement: Supplementary file 7 — Source data Fig. 3 [file 44318_2024_104_MOESM7_ESM.zip › Figure 3/3C/CBB.tif]

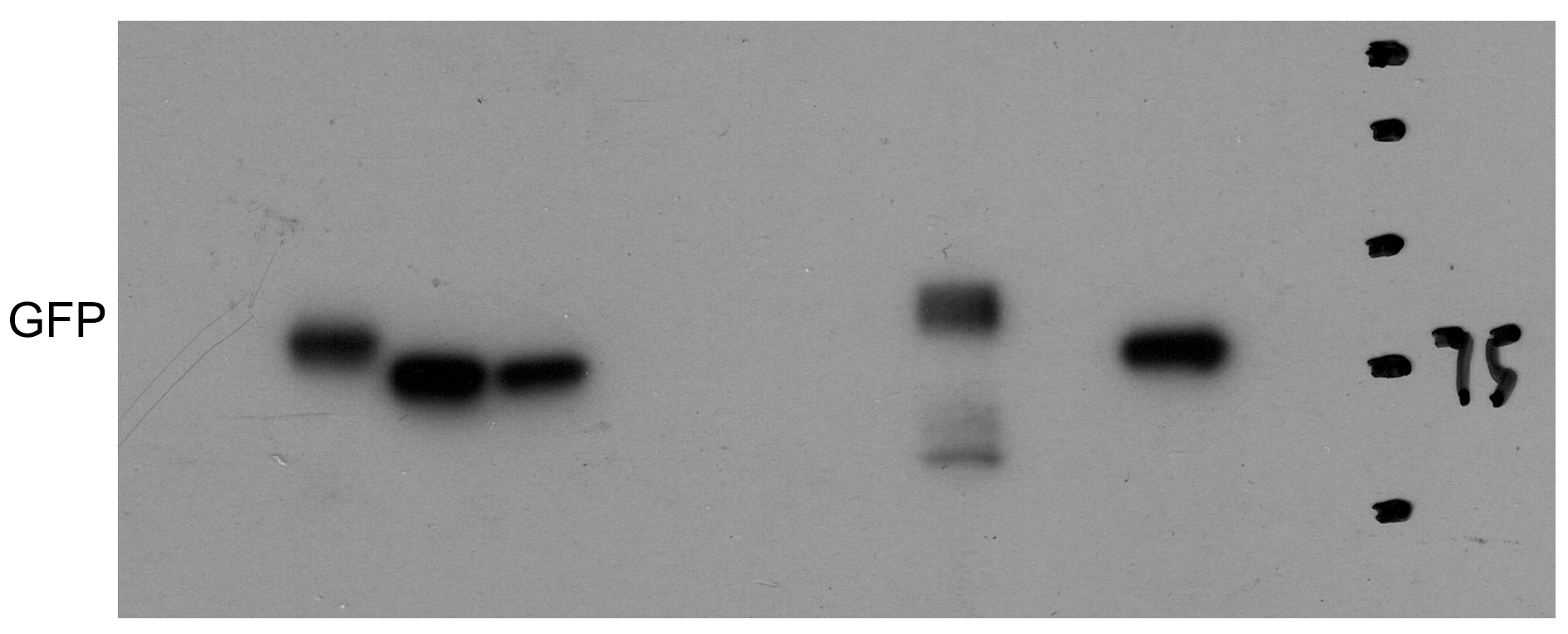

Supplement: Supplementary file 7 — Source data Fig. 3 [file 44318_2024_104_MOESM7_ESM.zip › Figure 3/3C/western GFP.tif]

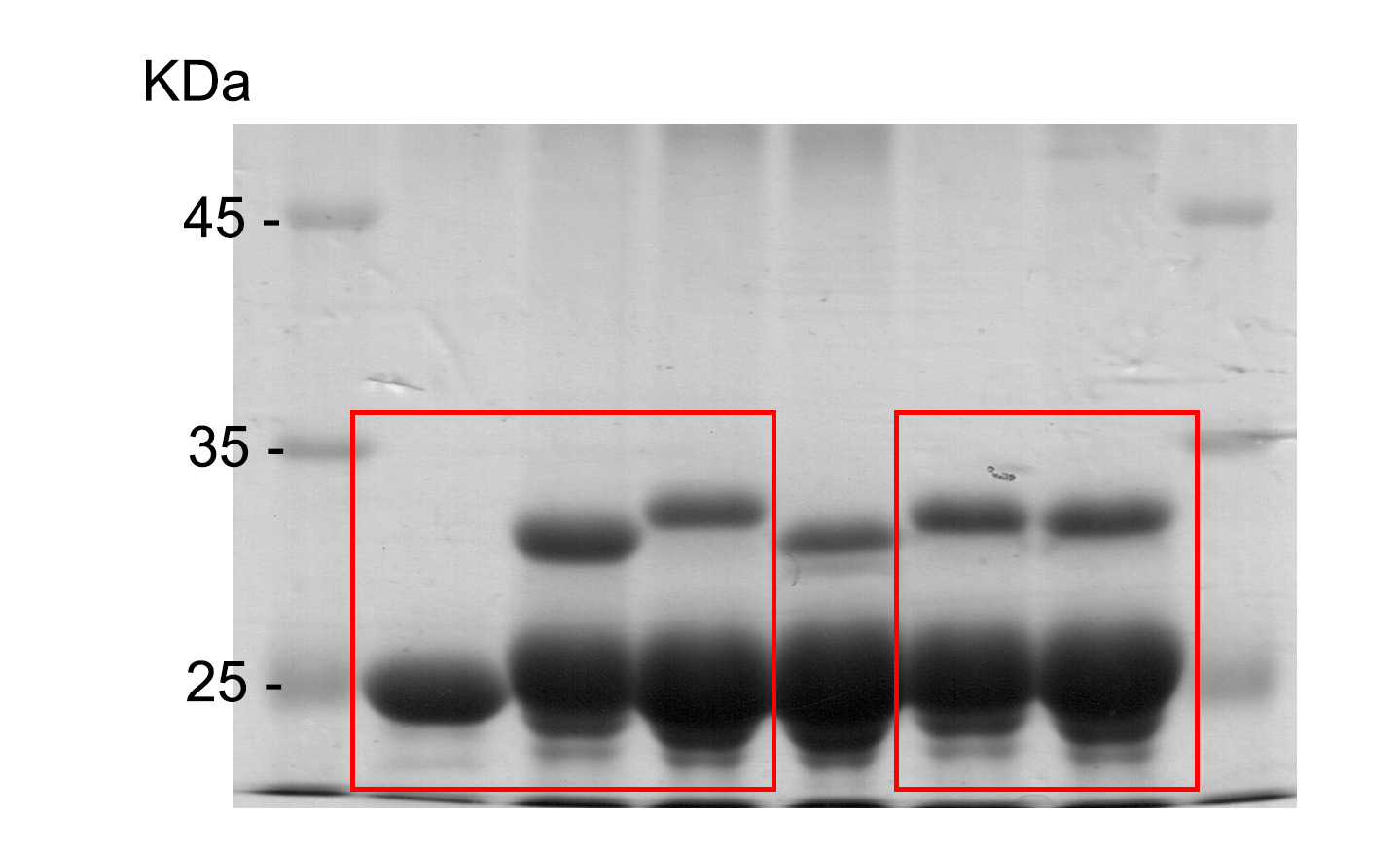

Supplement: Supplementary file 7 — Source data Fig. 3 [file 44318_2024_104_MOESM7_ESM.zip › Figure 3/3E/CBB.tif]

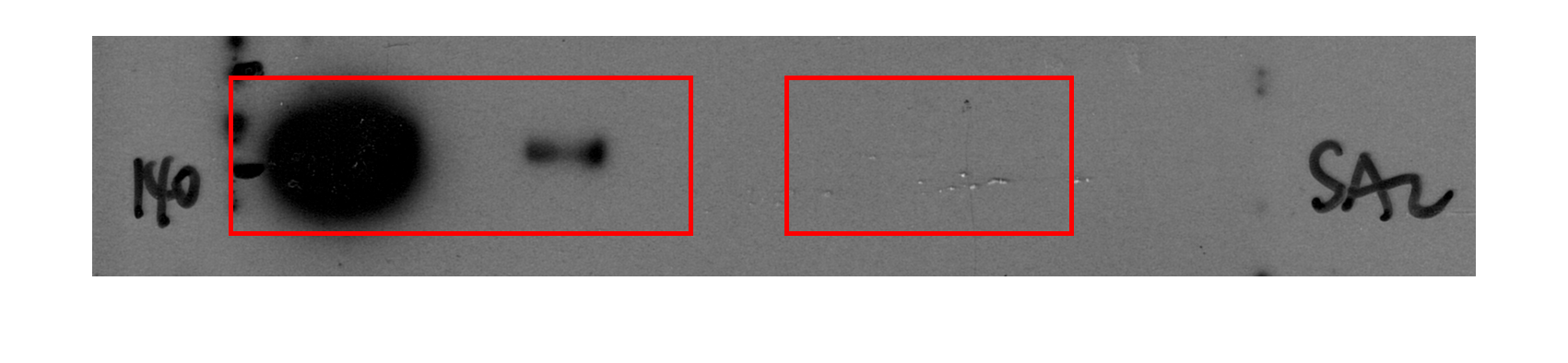

Supplement: Supplementary file 7 — Source data Fig. 3 [file 44318_2024_104_MOESM7_ESM.zip › Figure 3/3E/western SA2.tif]

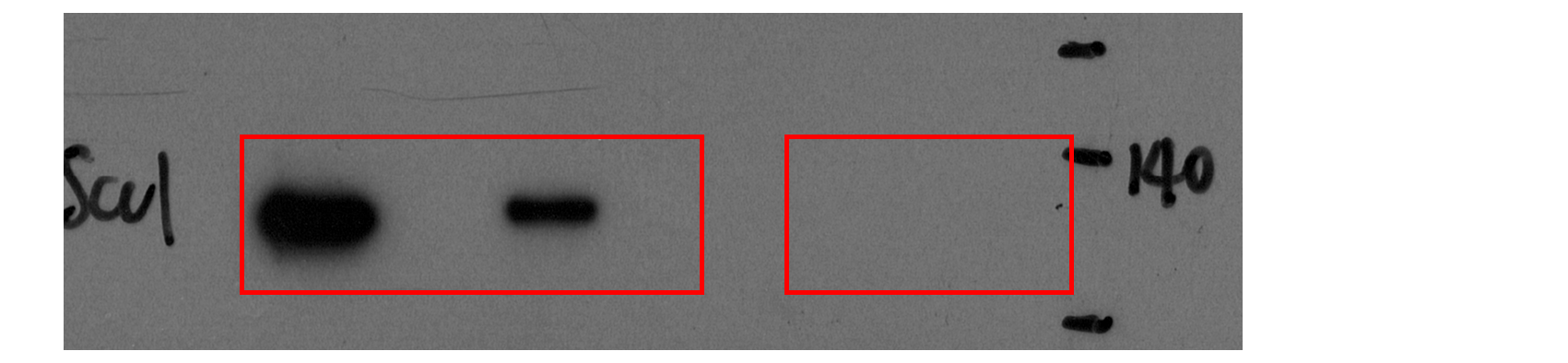

Supplement: Supplementary file 7 — Source data Fig. 3 [file 44318_2024_104_MOESM7_ESM.zip › Figure 3/3E/western Scc1.tif]

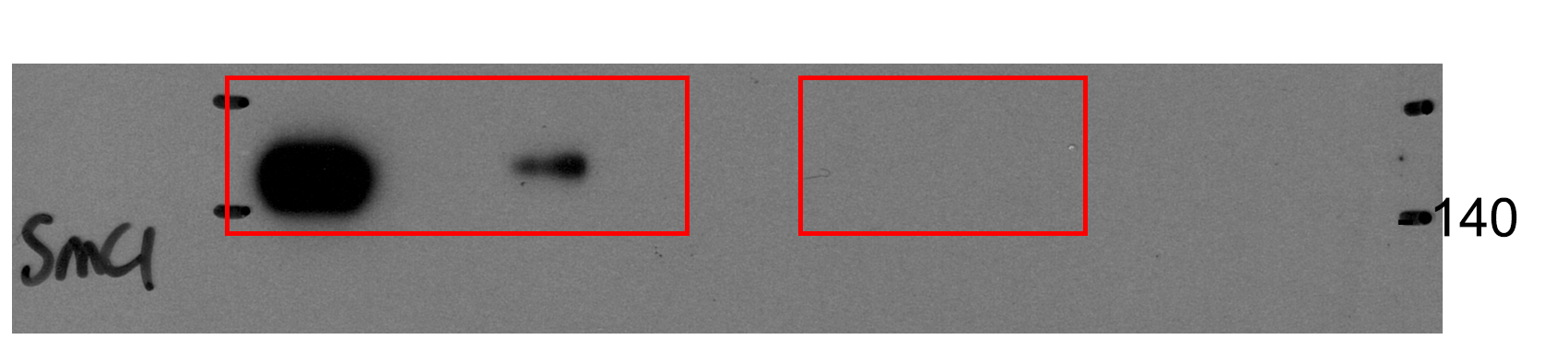

Supplement: Supplementary file 7 — Source data Fig. 3 [file 44318_2024_104_MOESM7_ESM.zip › Figure 3/3E/western SMC1.tif]

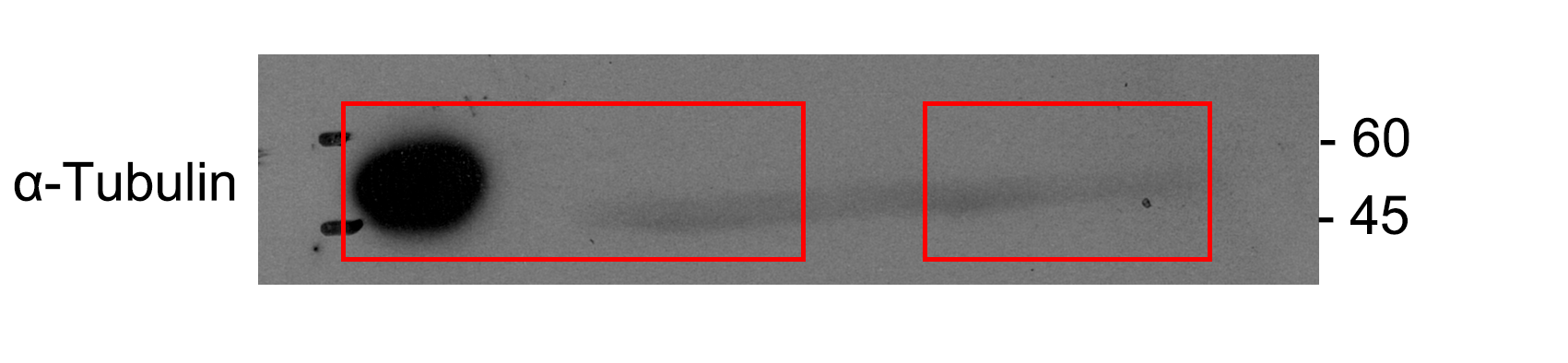

Supplement: Supplementary file 7 — Source data Fig. 3 [file 44318_2024_104_MOESM7_ESM.zip › Figure 3/3E/western a┴-Tubulin.tif]

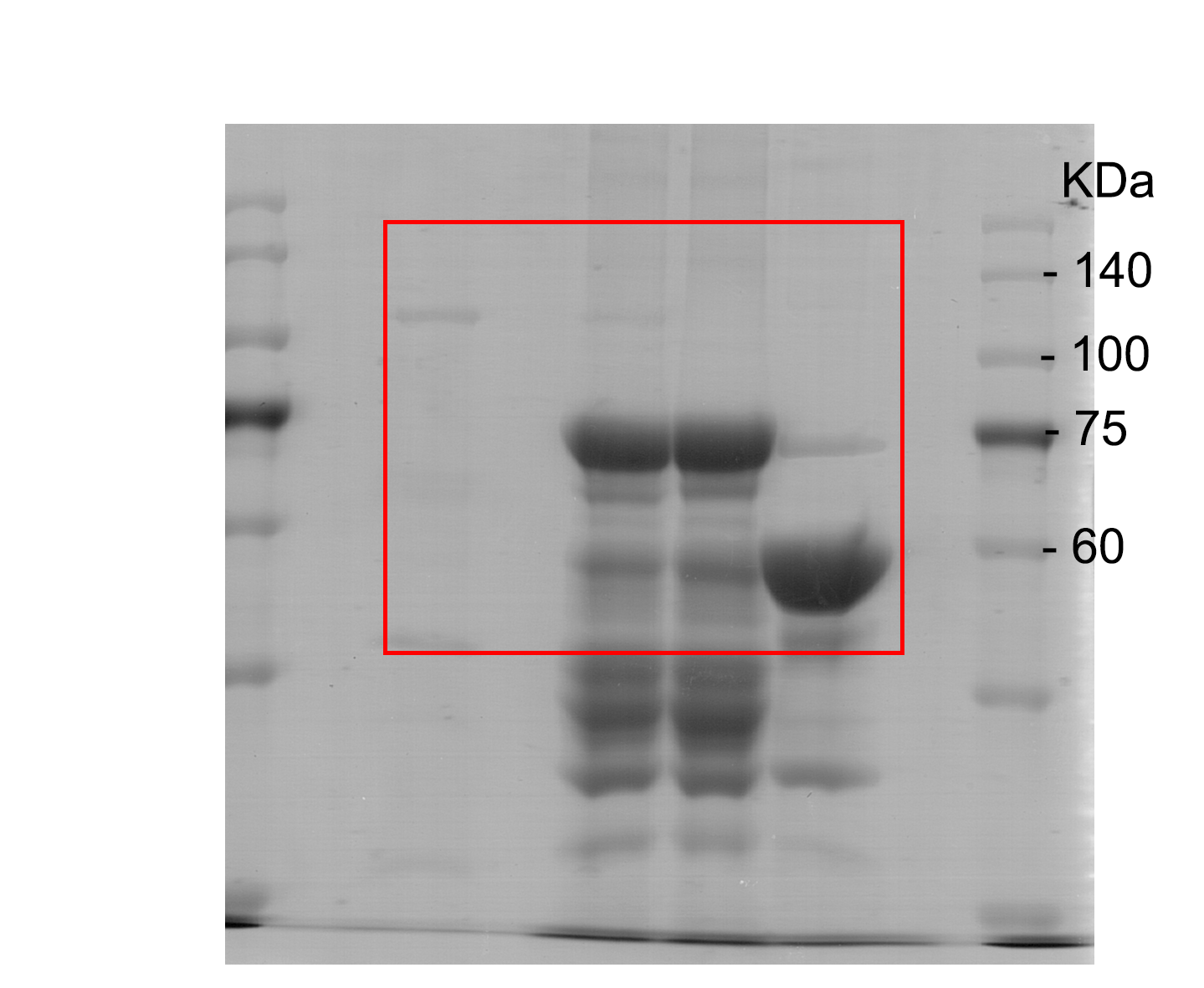

Supplement: Supplementary file 7 — Source data Fig. 3 [file 44318_2024_104_MOESM7_ESM.zip › Figure 3/3F/CBB.tif]

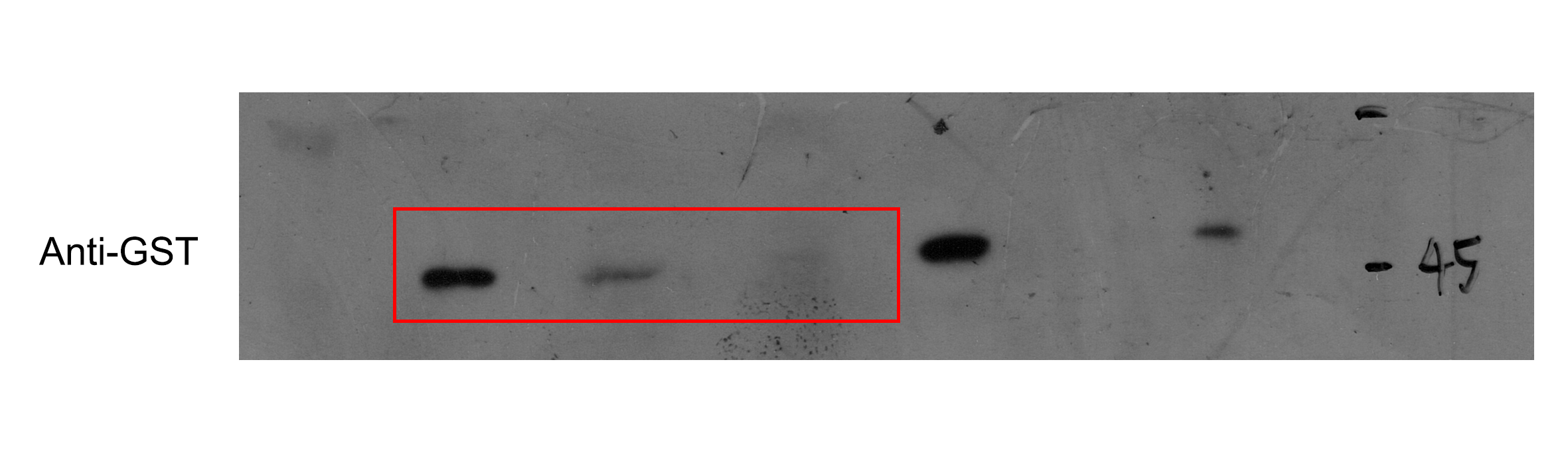

Supplement: Supplementary file 7 — Source data Fig. 3 [file 44318_2024_104_MOESM7_ESM.zip › Figure 3/3F/western GST.tif]

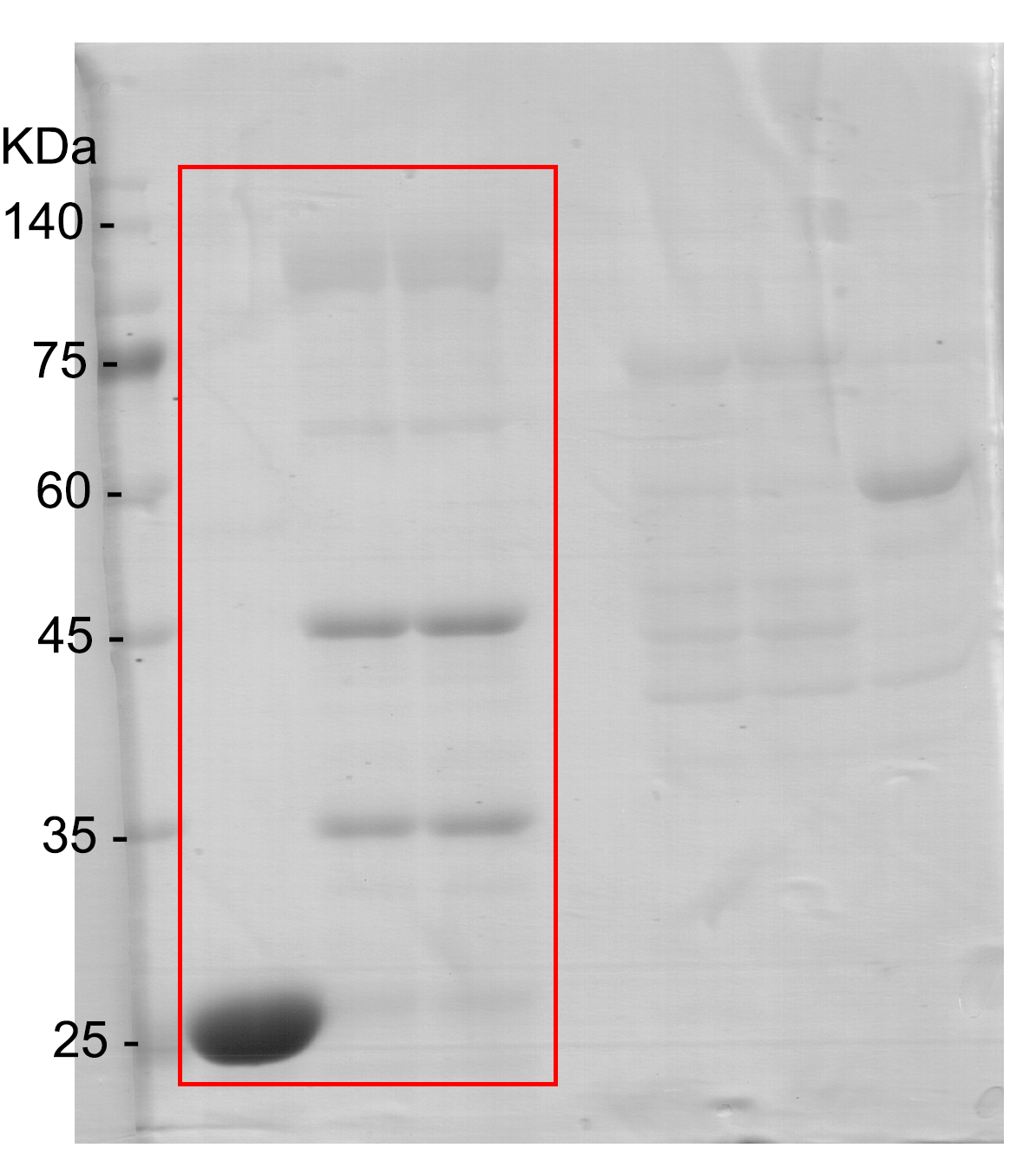

Supplement: Supplementary file 7 — Source data Fig. 3 [file 44318_2024_104_MOESM7_ESM.zip › Figure 3/3G/CBB.tif]

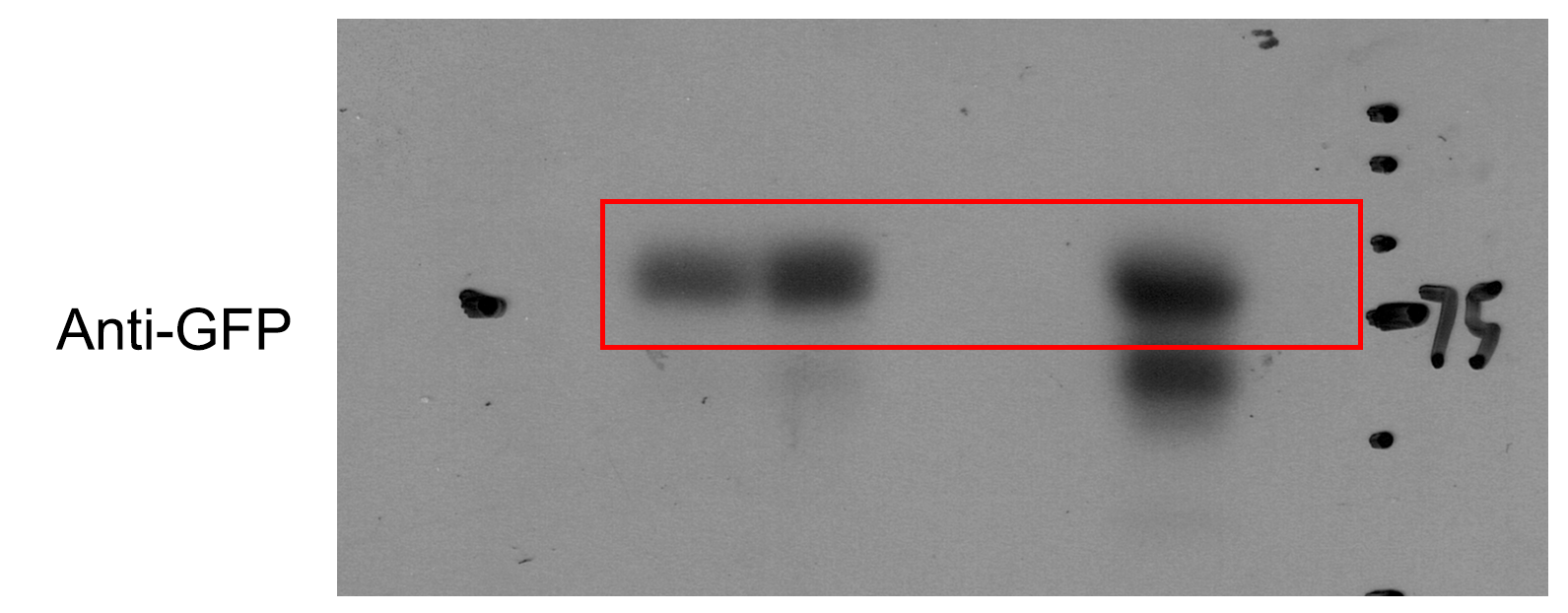

Supplement: Supplementary file 7 — Source data Fig. 3 [file 44318_2024_104_MOESM7_ESM.zip › Figure 3/3G/western GFP.tif]

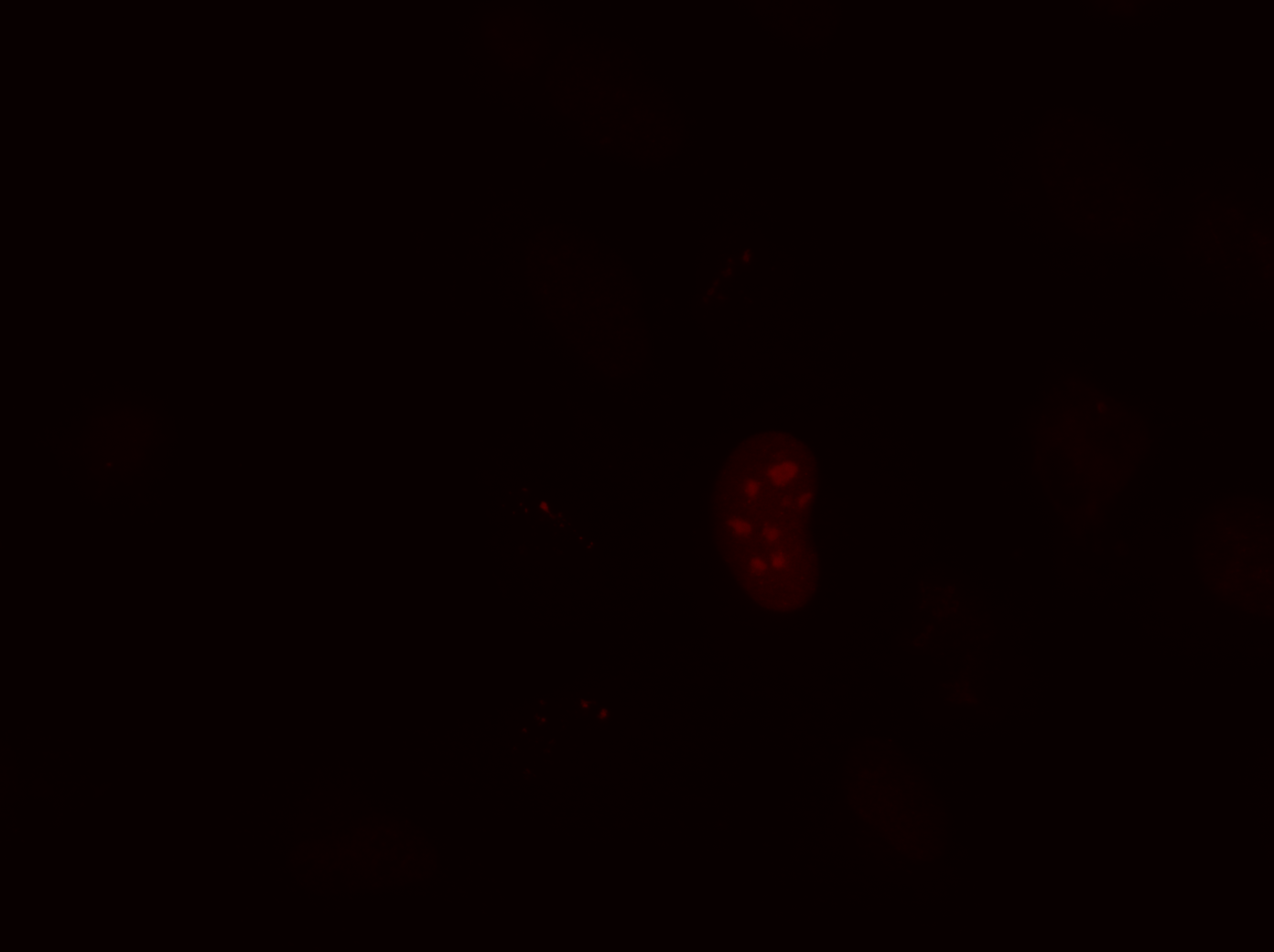

Supplement: Supplementary file 7 — Source data Fig. 3 [file 44318_2024_104_MOESM7_ESM.zip › Figure 3/3H/EGFP-LacI+Myc-SA2+SFB-CENP-U Anti-Flag.tif]

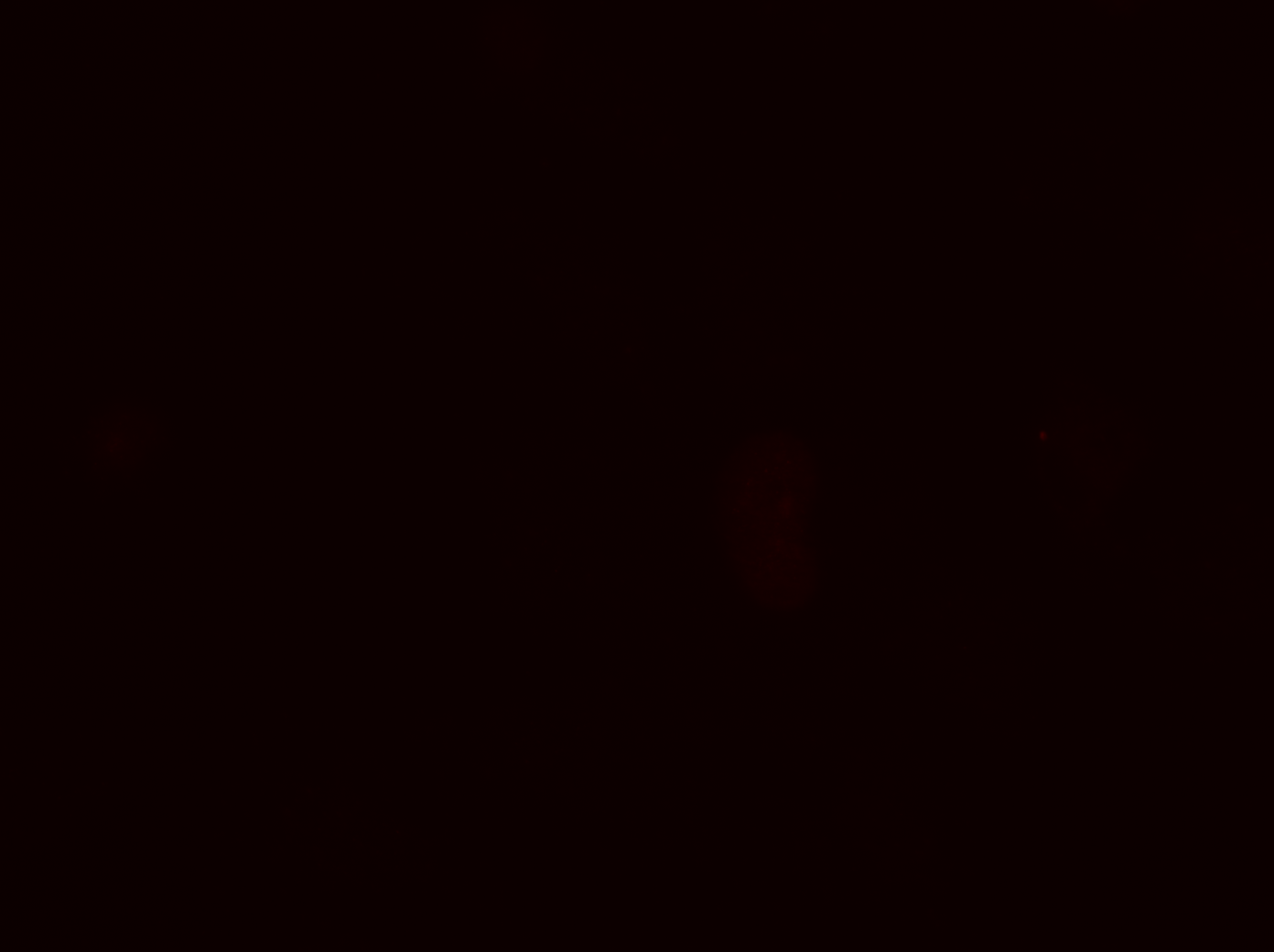

Supplement: Supplementary file 7 — Source data Fig. 3 [file 44318_2024_104_MOESM7_ESM.zip › Figure 3/3H/EGFP-LacI+Myc-SA2+SFB-CENP-U Anti-Myc.tif]

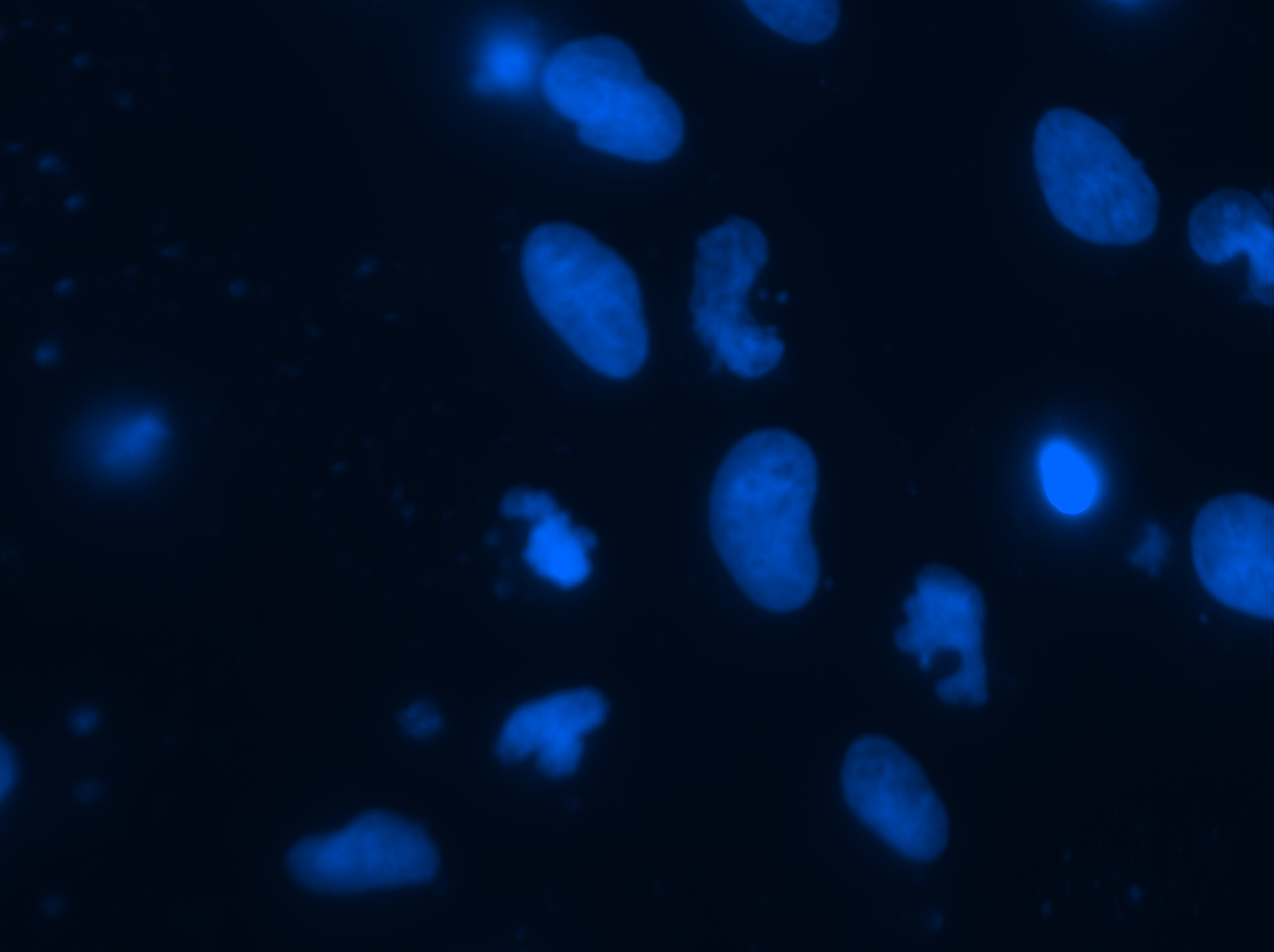

Supplement: Supplementary file 7 — Source data Fig. 3 [file 44318_2024_104_MOESM7_ESM.zip › Figure 3/3H/EGFP-LacI+Myc-SA2+SFB-CENP-U DNA.tif]

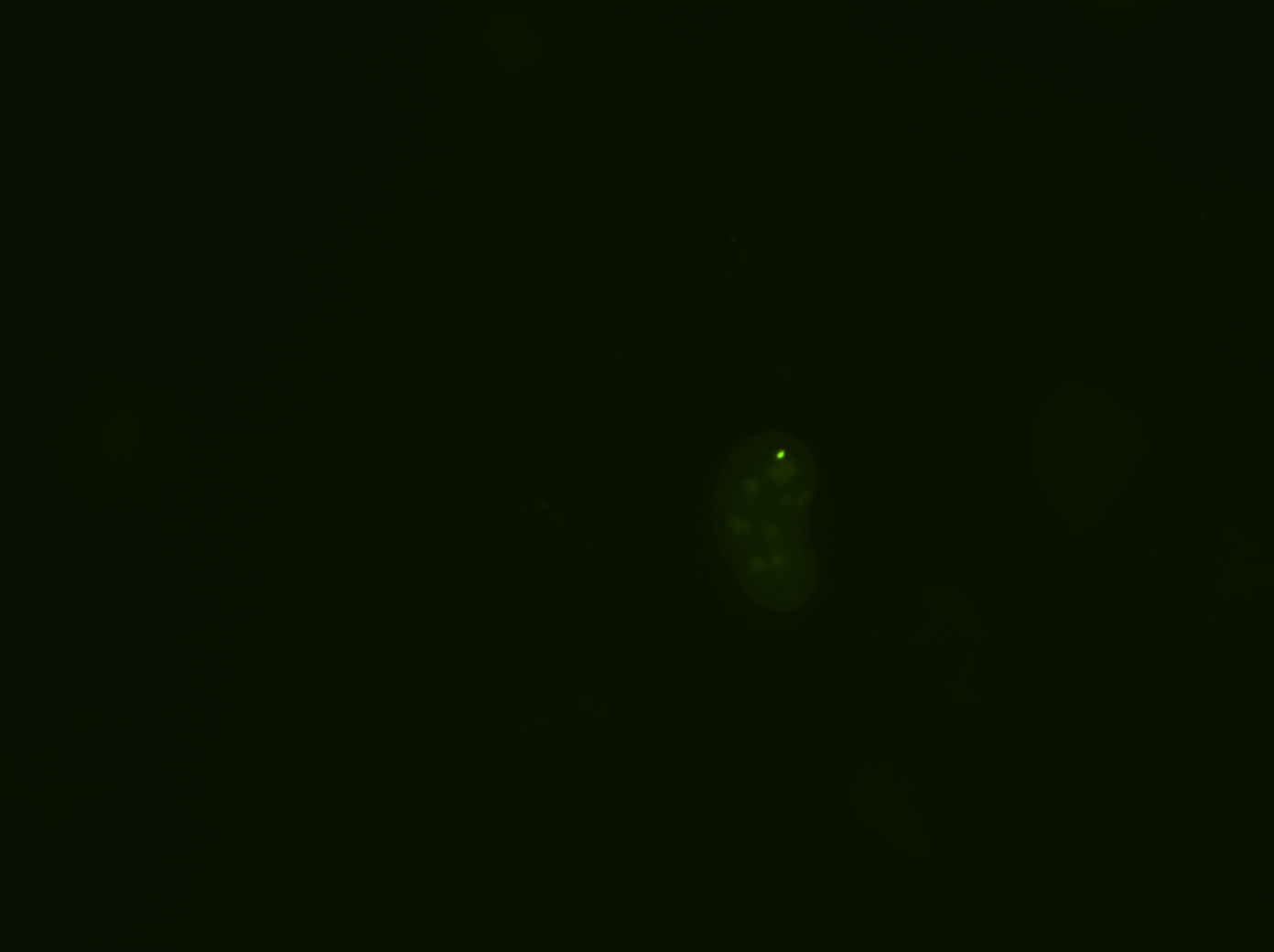

Supplement: Supplementary file 7 — Source data Fig. 3 [file 44318_2024_104_MOESM7_ESM.zip › Figure 3/3H/EGFP-LacI+Myc-SA2+SFB-CENP-U EGFP.tif]

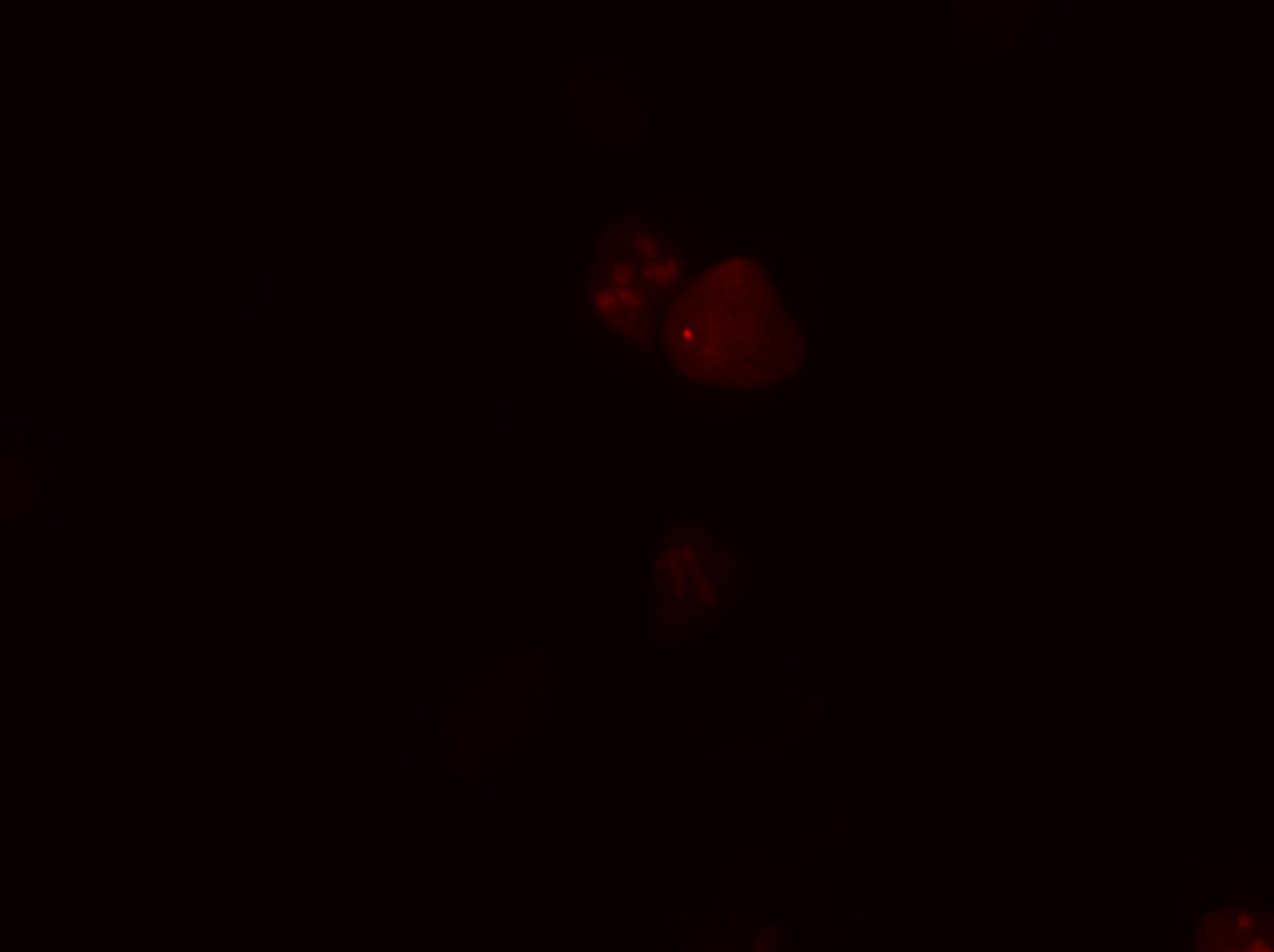

Supplement: Supplementary file 7 — Source data Fig. 3 [file 44318_2024_104_MOESM7_ESM.zip › Figure 3/3H/EGFP-LacI-Scc1+Myc-SA2+SFB-CENP-U Anti-Flag.tif]

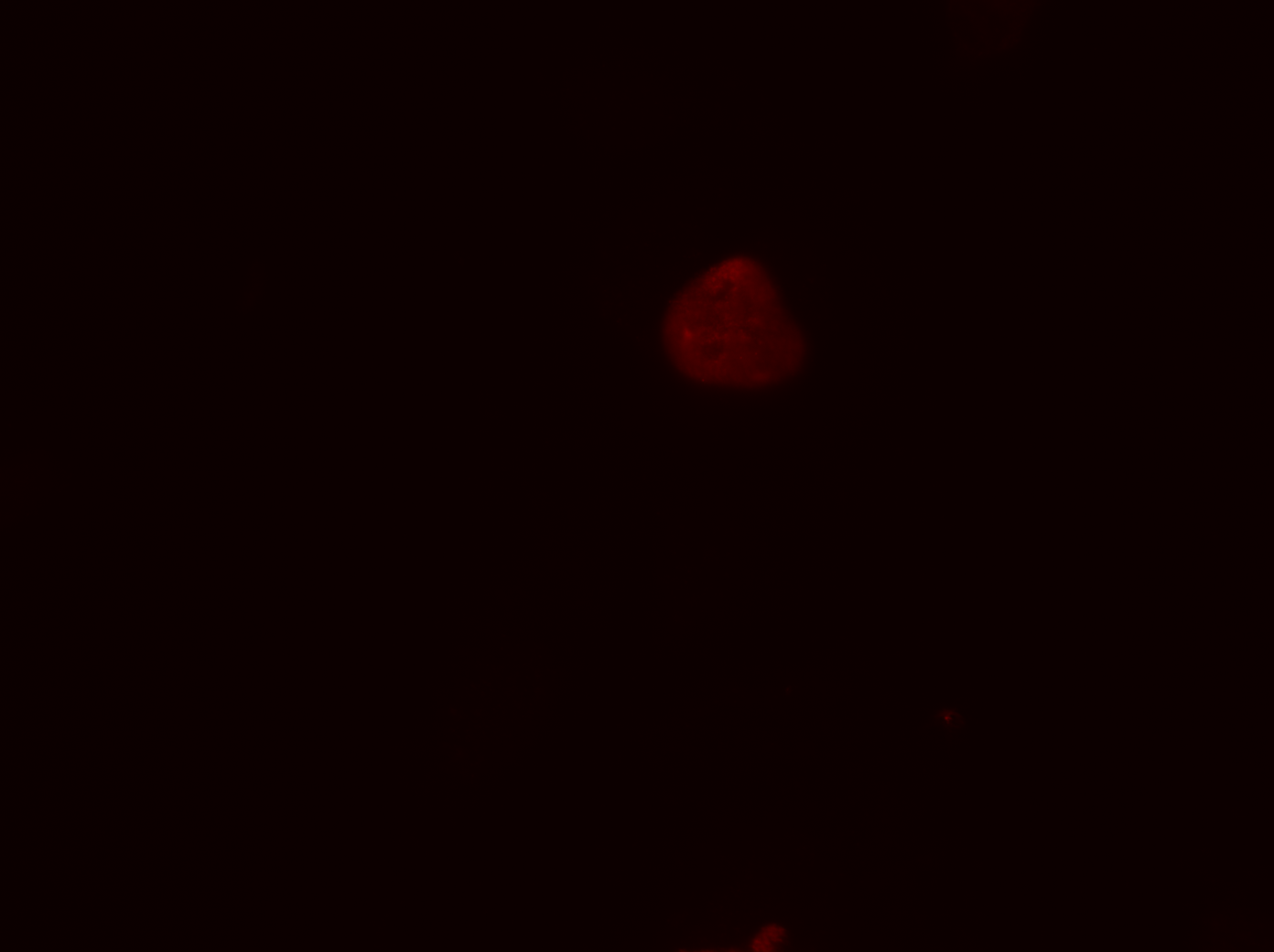

Supplement: Supplementary file 7 — Source data Fig. 3 [file 44318_2024_104_MOESM7_ESM.zip › Figure 3/3H/EGFP-LacI-Scc1+Myc-SA2+SFB-CENP-U Anti-Myc.tif]

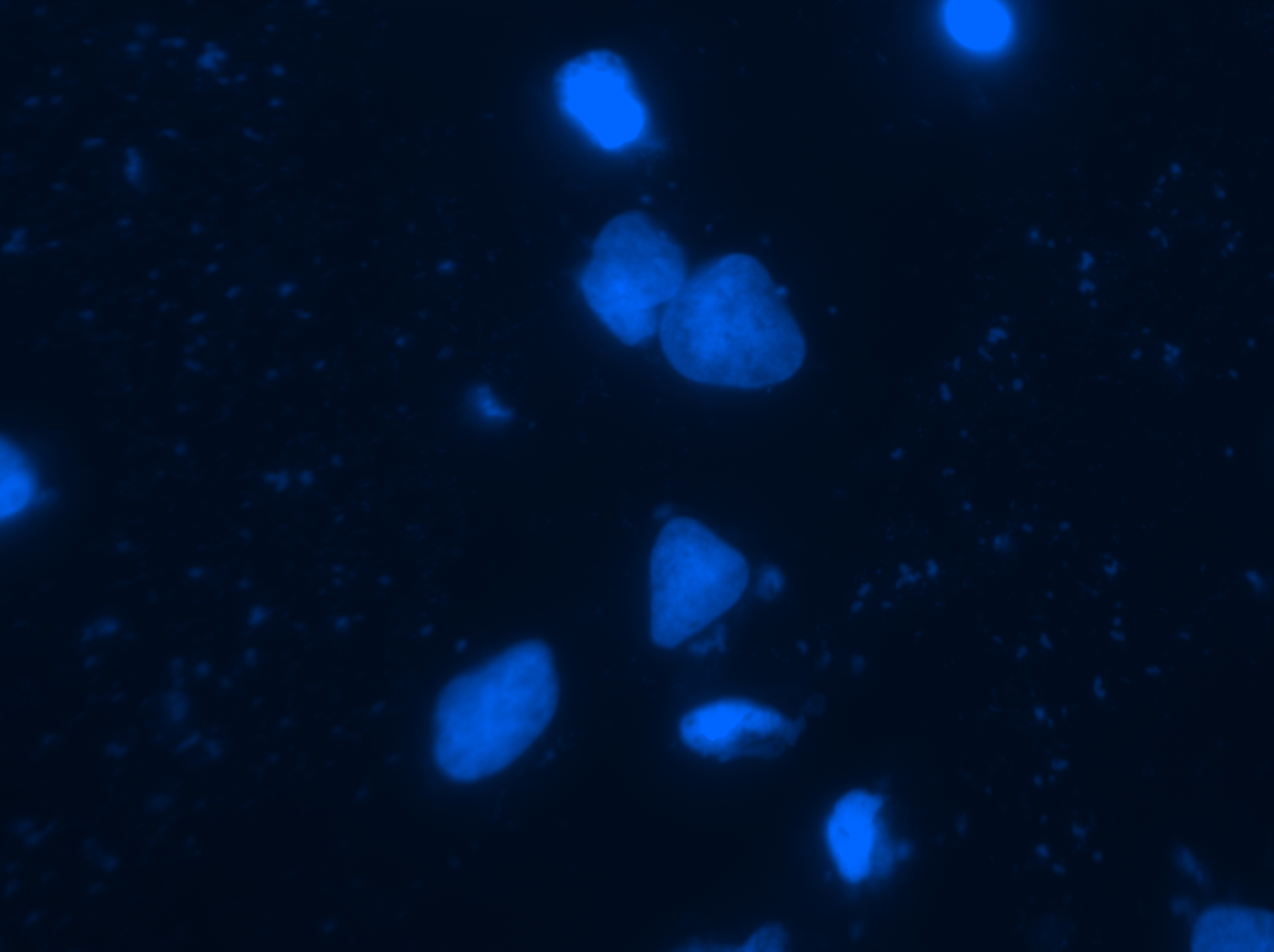

Supplement: Supplementary file 7 — Source data Fig. 3 [file 44318_2024_104_MOESM7_ESM.zip › Figure 3/3H/EGFP-LacI-Scc1+Myc-SA2+SFB-CENP-U DNA.tif]

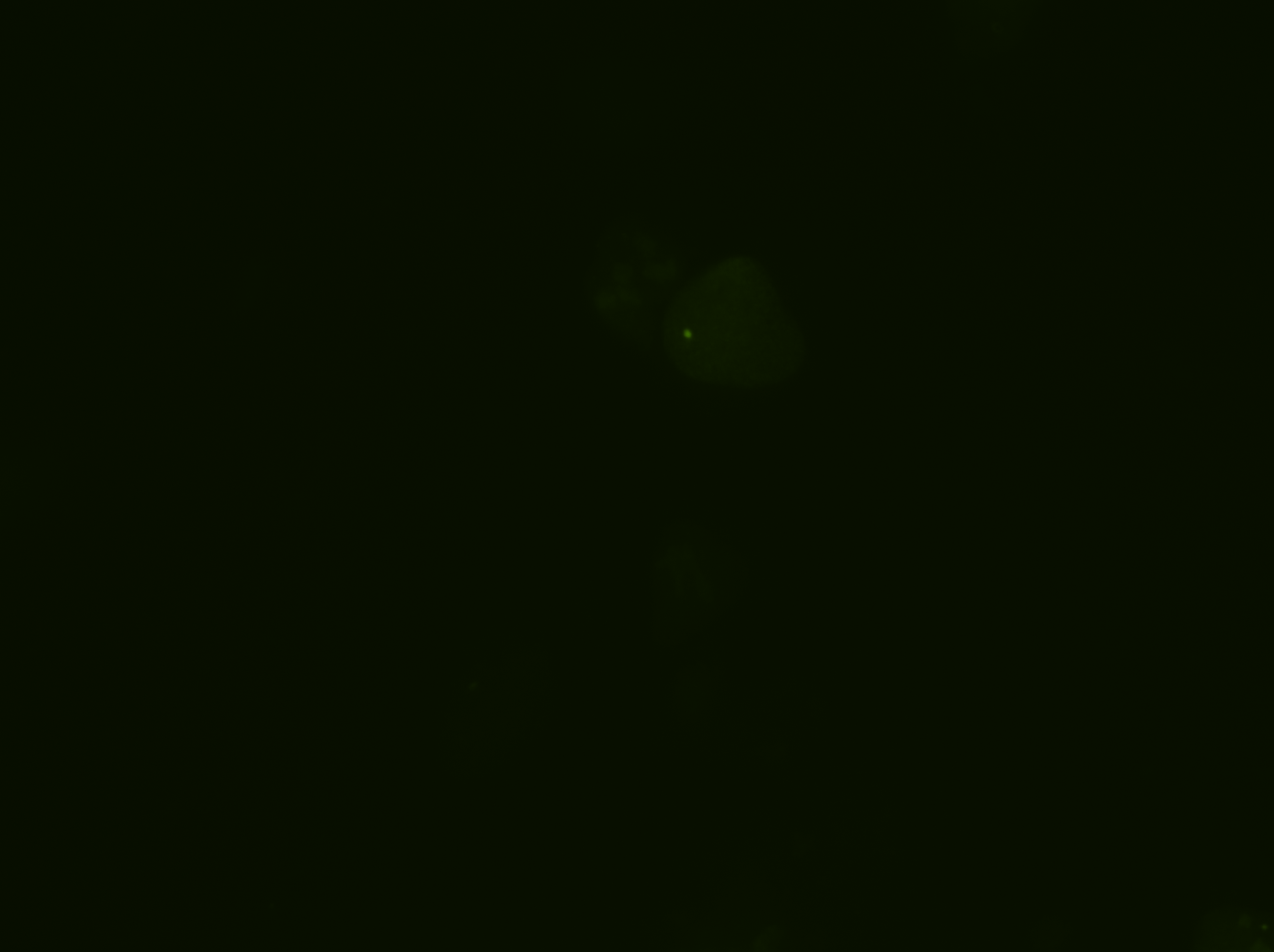

Supplement: Supplementary file 7 — Source data Fig. 3 [file 44318_2024_104_MOESM7_ESM.zip › Figure 3/3H/EGFP-LacI-Scc1+Myc-SA2+SFB-CENP-U EGFP.tif]

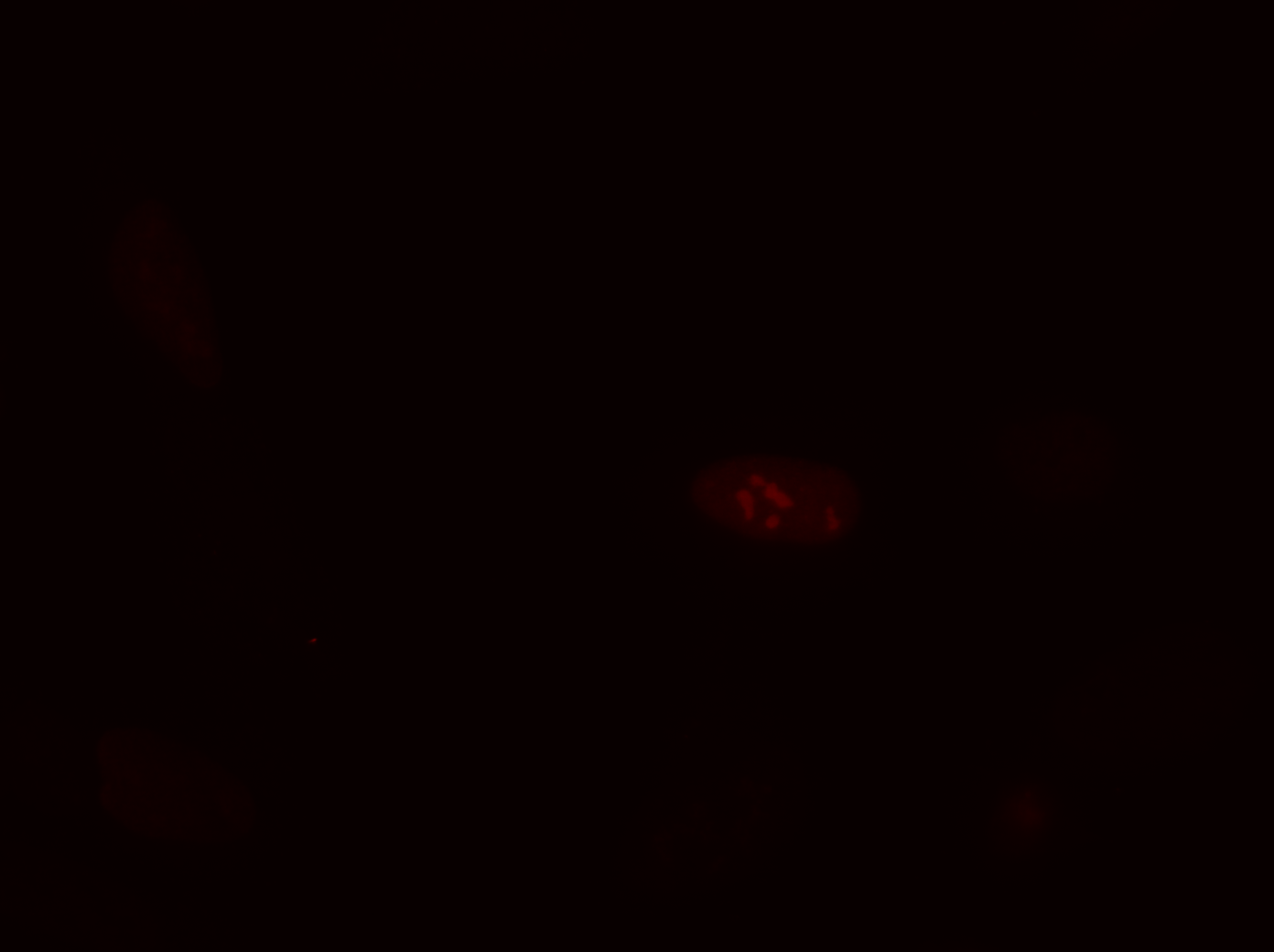

Supplement: Supplementary file 7 — Source data Fig. 3 [file 44318_2024_104_MOESM7_ESM.zip › Figure 3/3H/EGFP-LacI-Scc1+Myc-SA2+SFB-CENP-U-ADA Anti-Flag.tif]

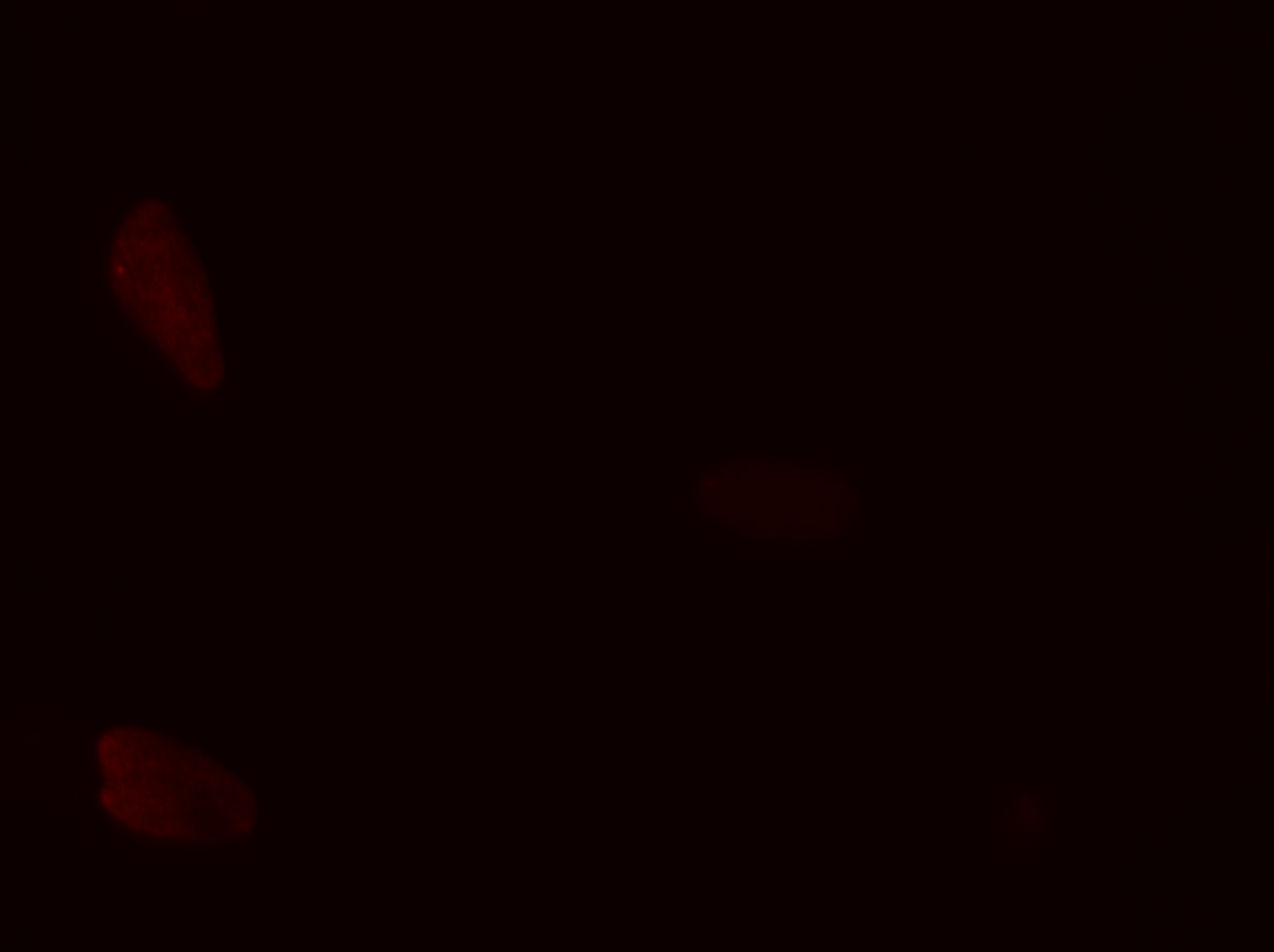

Supplement: Supplementary file 7 — Source data Fig. 3 [file 44318_2024_104_MOESM7_ESM.zip › Figure 3/3H/EGFP-LacI-Scc1+Myc-SA2+SFB-CENP-U-ADA Anti-Myc.tif]

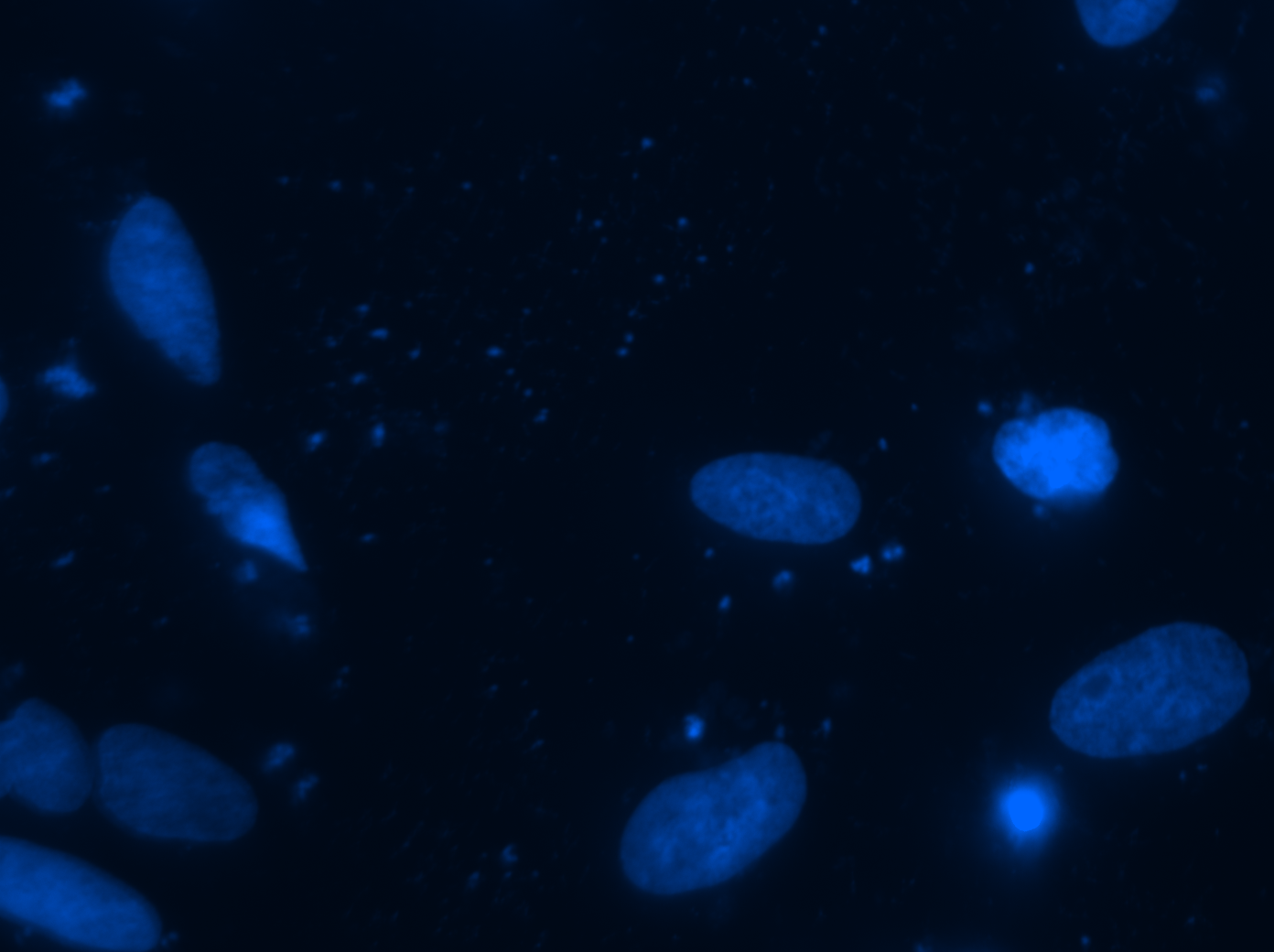

Supplement: Supplementary file 7 — Source data Fig. 3 [file 44318_2024_104_MOESM7_ESM.zip › Figure 3/3H/EGFP-LacI-Scc1+Myc-SA2+SFB-CENP-U-ADA DNA.tif]

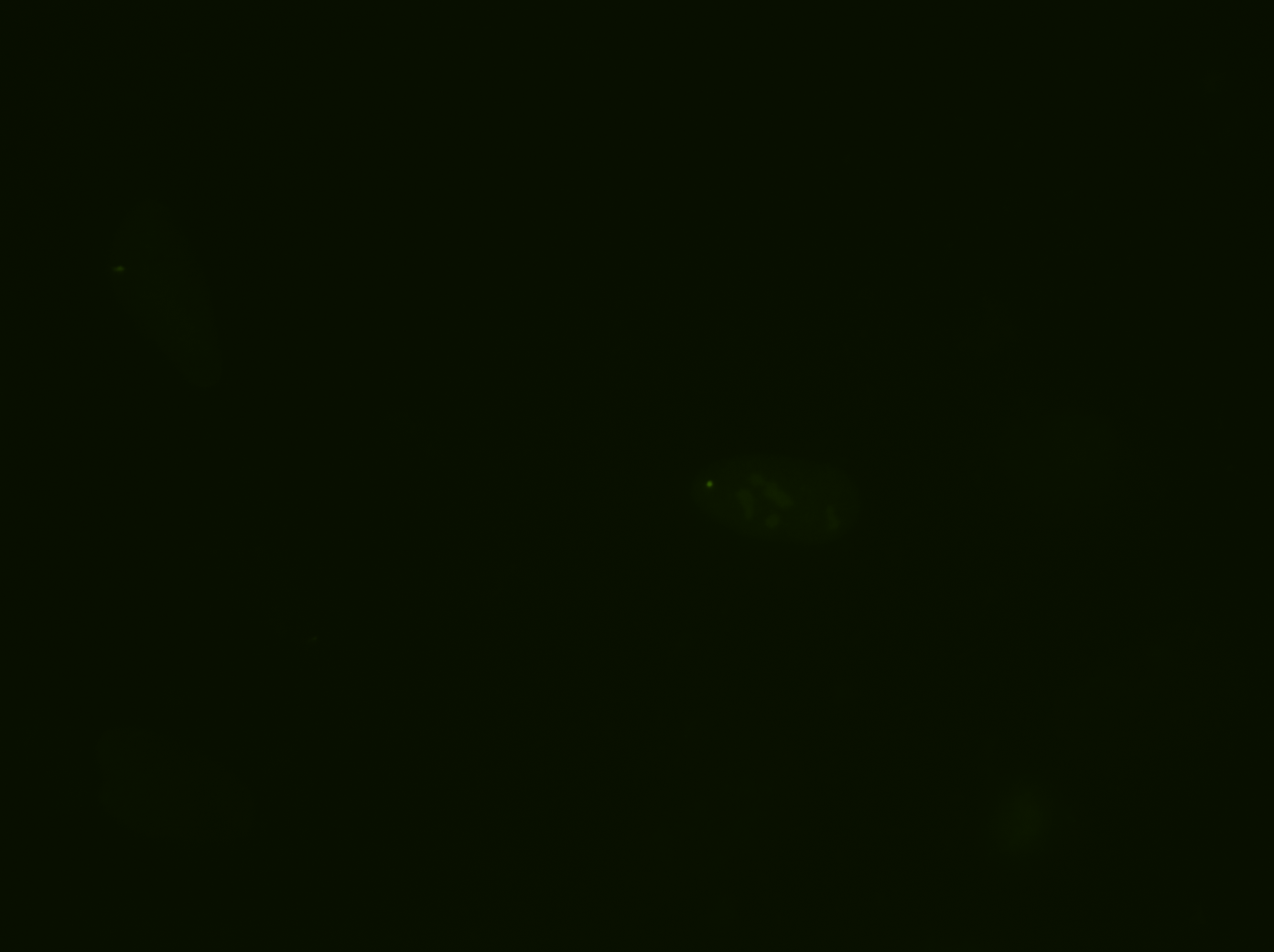

Supplement: Supplementary file 7 — Source data Fig. 3 [file 44318_2024_104_MOESM7_ESM.zip › Figure 3/3H/EGFP-LacI-Scc1+Myc-SA2+SFB-CENP-U-ADA EGFP.tif]

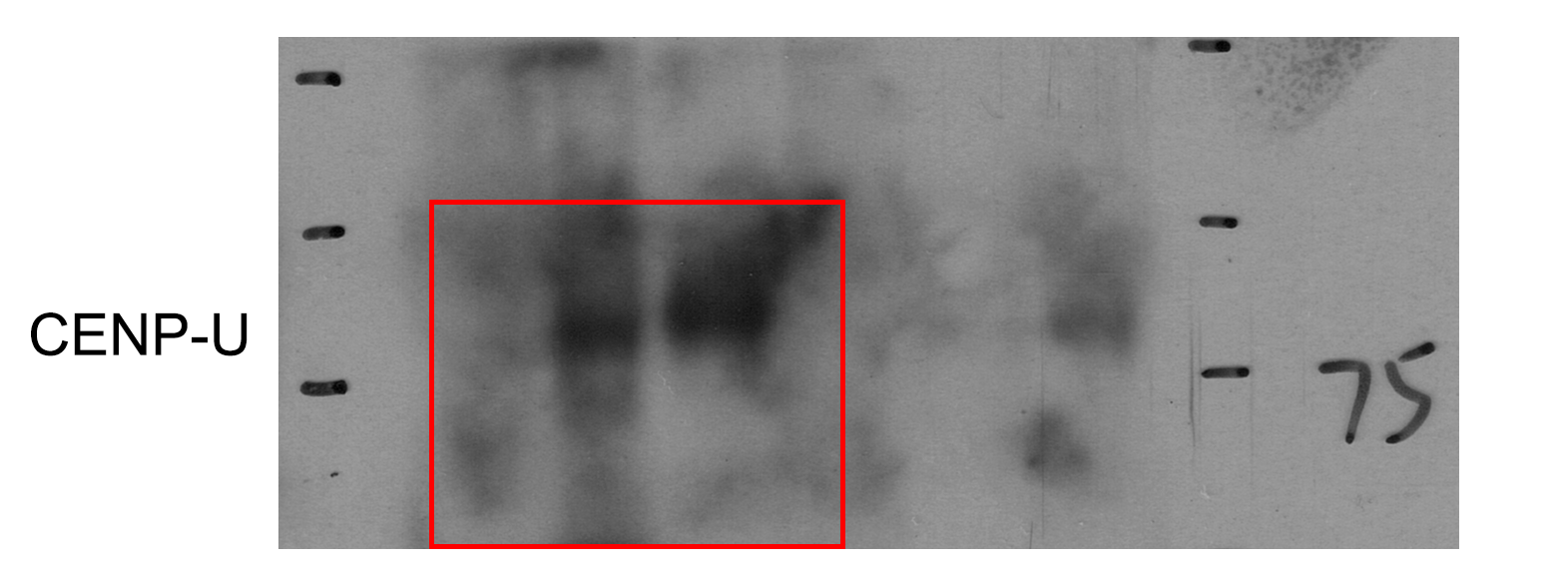

Supplement: Supplementary file 8 — Source data Fig. 4 [file 44318_2024_104_MOESM8_ESM.zip › Figure 4/4A/western CENP-U.tif]
