# Supplementary figures and images for: A non-canonical role of the inner kinetochore in regulating sister-chromatid cohesion at centromeres (part 2 of 3)
Source: EMBO J. 2024 May 7;43(12):7. doi: 10.1038/s44318-024-00104-6 (PMC11182772; doi:10.1038/s44318-024-00104-6)

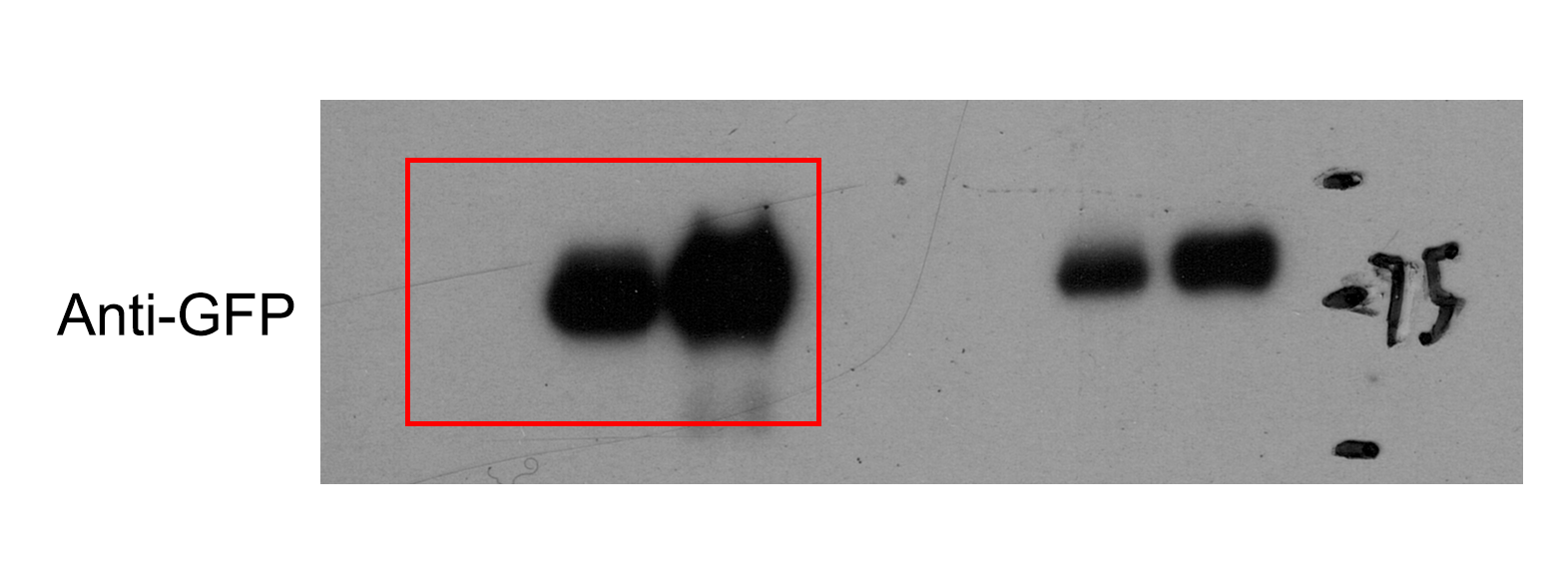

Supplement: Supplementary file 8 — Source data Fig. 4 [file 44318_2024_104_MOESM8_ESM.zip › Figure 4/4A/western GFP.tif]

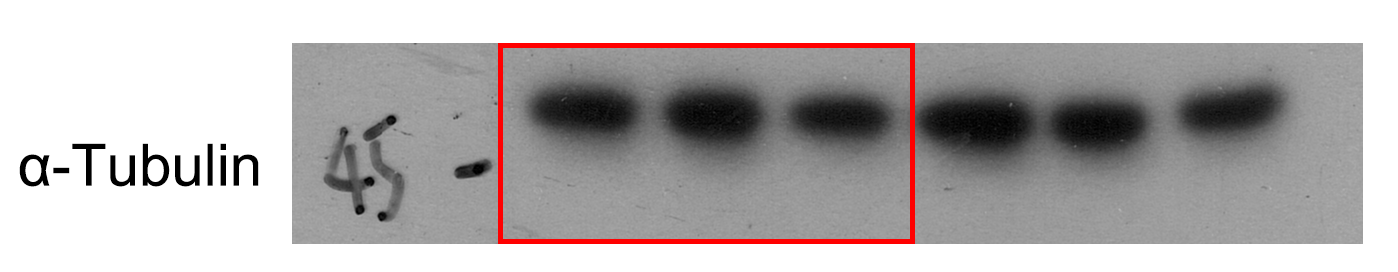

Supplement: Supplementary file 8 — Source data Fig. 4 [file 44318_2024_104_MOESM8_ESM.zip › Figure 4/4A/western a┴-Tubulin.tif]

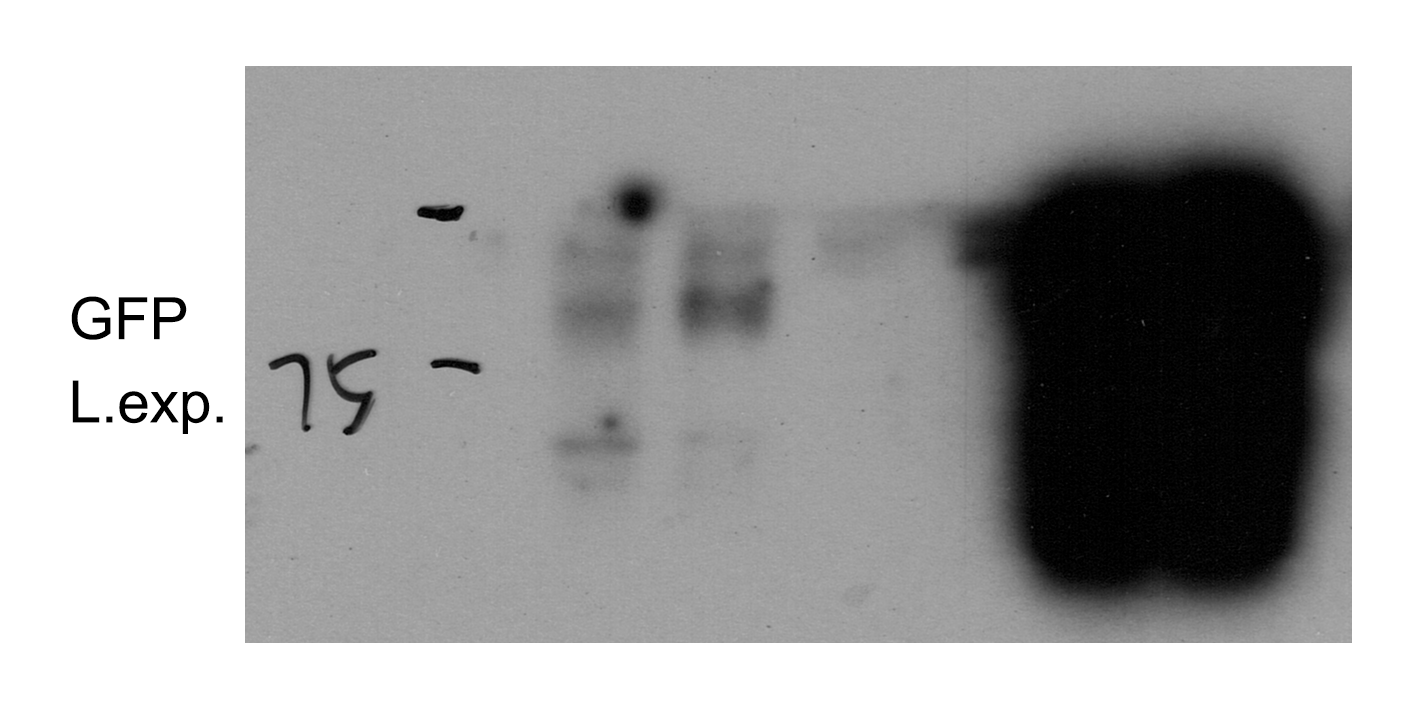

Supplement: Supplementary file 8 — Source data Fig. 4 [file 44318_2024_104_MOESM8_ESM.zip › Figure 4/4B/western GFP L.exp..tif]

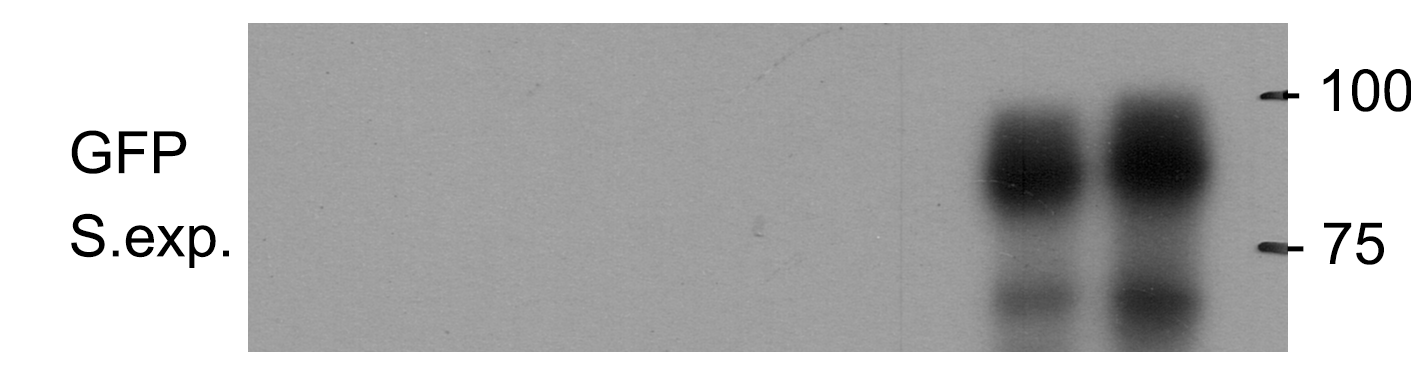

Supplement: Supplementary file 8 — Source data Fig. 4 [file 44318_2024_104_MOESM8_ESM.zip › Figure 4/4B/western GFP S.exp..tif]

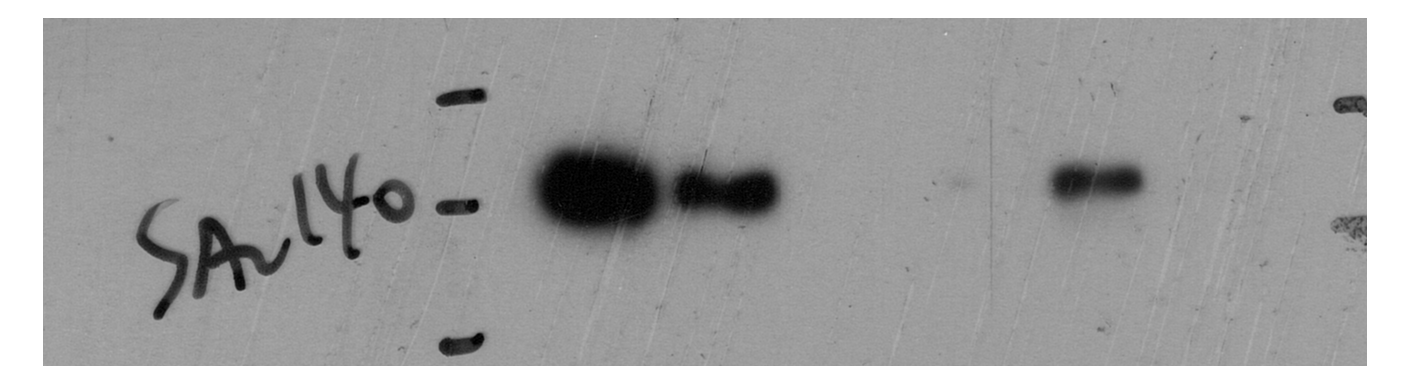

Supplement: Supplementary file 8 — Source data Fig. 4 [file 44318_2024_104_MOESM8_ESM.zip › Figure 4/4B/western SA2.tif]

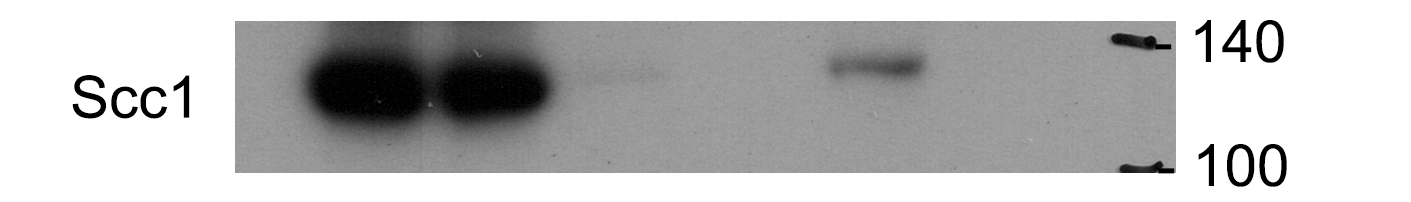

Supplement: Supplementary file 8 — Source data Fig. 4 [file 44318_2024_104_MOESM8_ESM.zip › Figure 4/4B/western Scc1.tif]

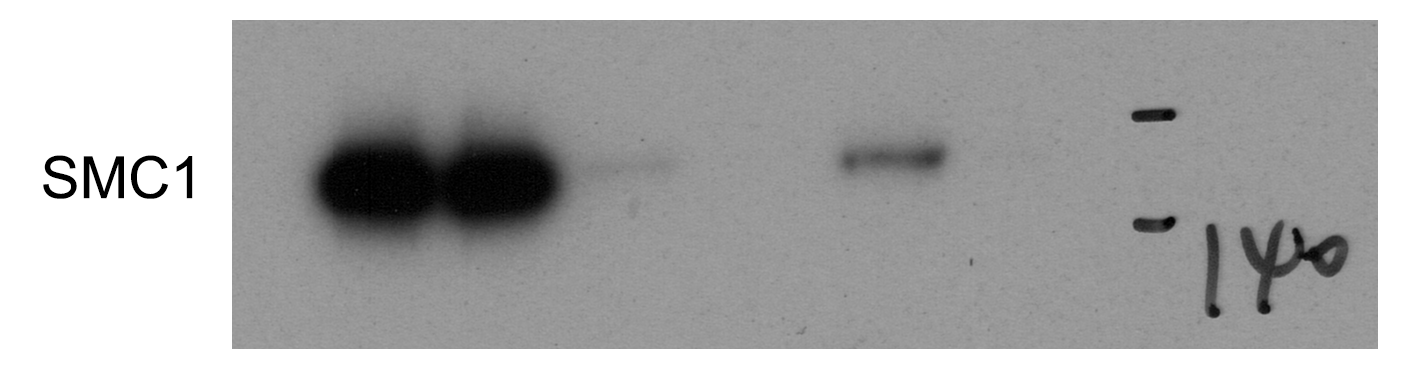

Supplement: Supplementary file 8 — Source data Fig. 4 [file 44318_2024_104_MOESM8_ESM.zip › Figure 4/4B/western SMC1.tif]

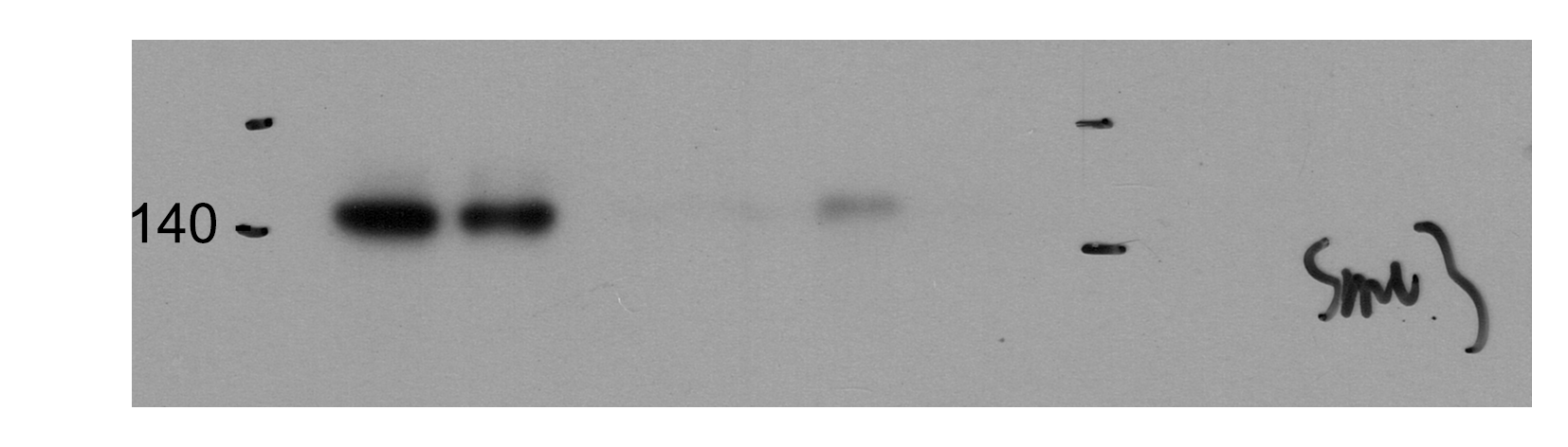

Supplement: Supplementary file 8 — Source data Fig. 4 [file 44318_2024_104_MOESM8_ESM.zip › Figure 4/4B/western SMC3.tif]

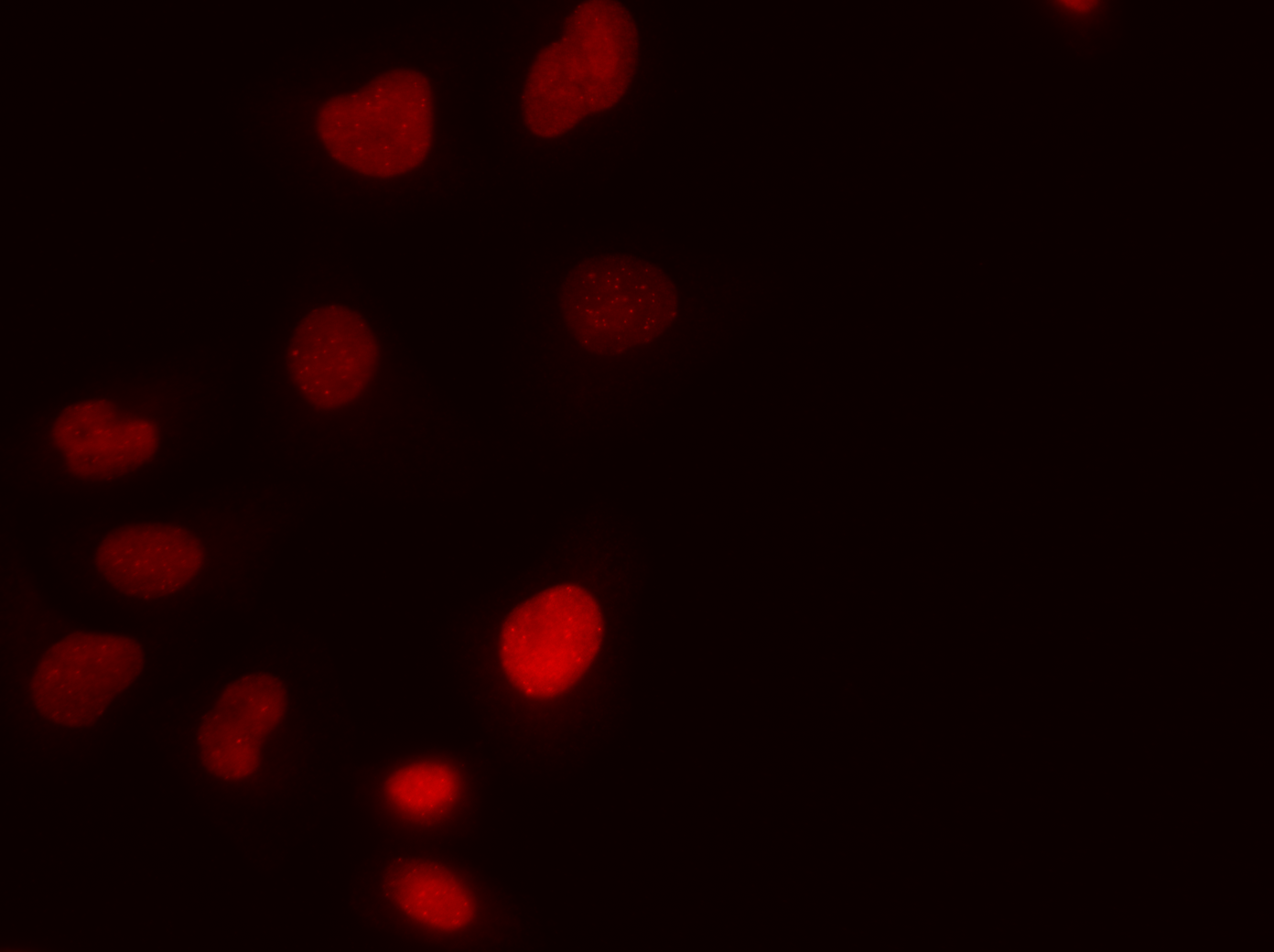

Supplement: Supplementary file 8 — Source data Fig. 4 [file 44318_2024_104_MOESM8_ESM.zip › Figure 4/4C/ADA Anti-GFP.tif]

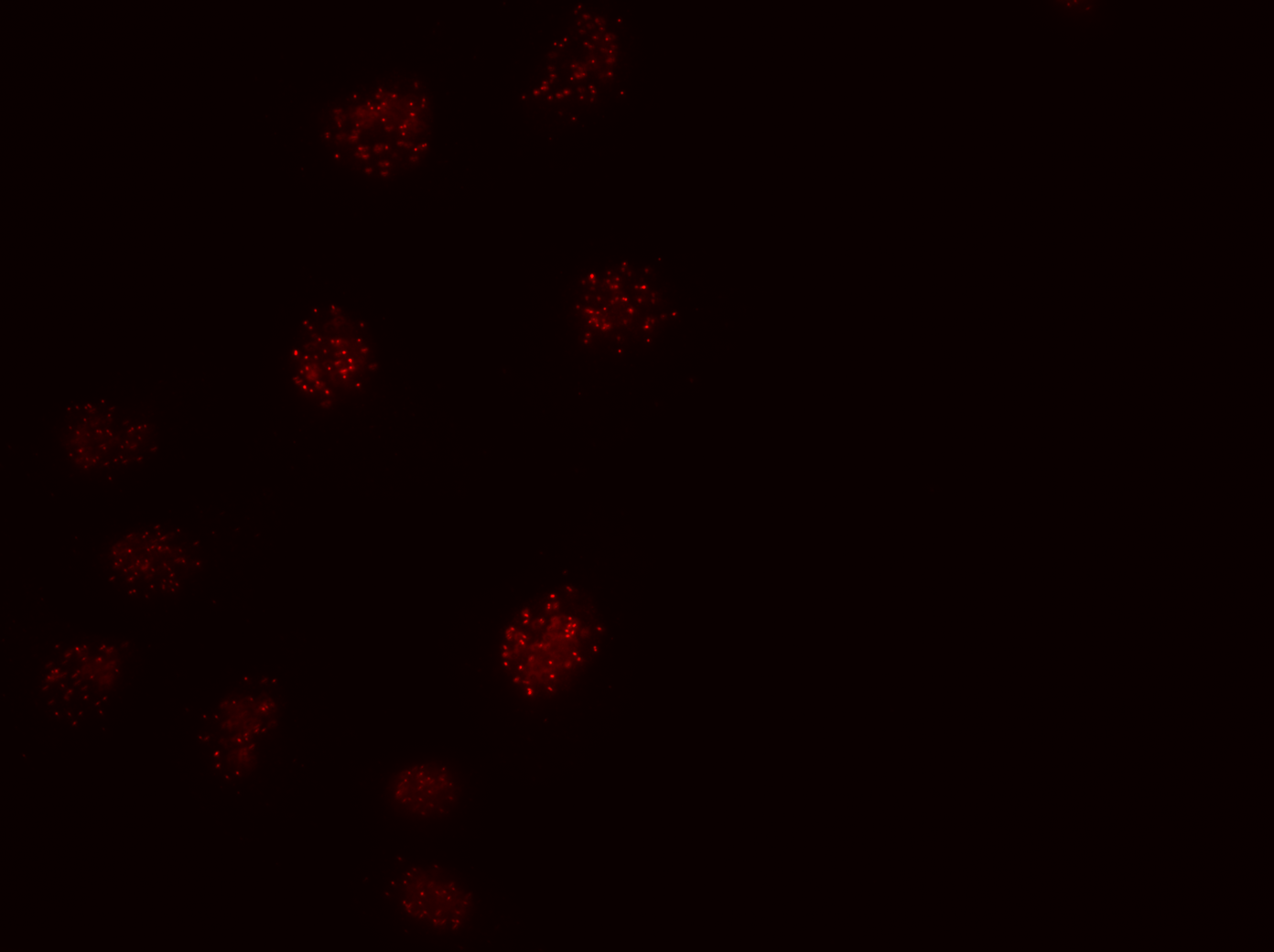

Supplement: Supplementary file 8 — Source data Fig. 4 [file 44318_2024_104_MOESM8_ESM.zip › Figure 4/4C/ADA CENP-C.tif]

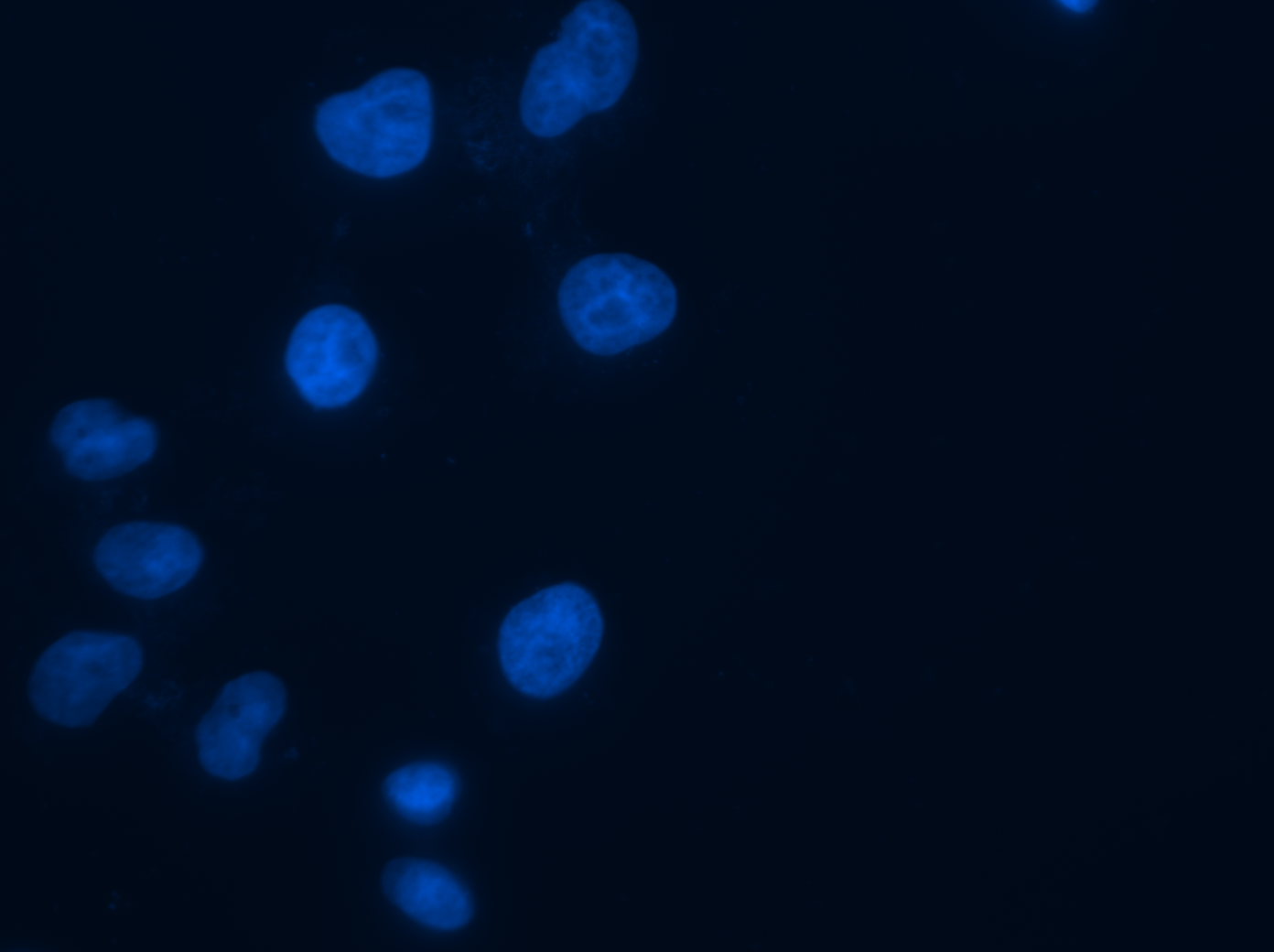

Supplement: Supplementary file 8 — Source data Fig. 4 [file 44318_2024_104_MOESM8_ESM.zip › Figure 4/4C/ADA DNA.tif]

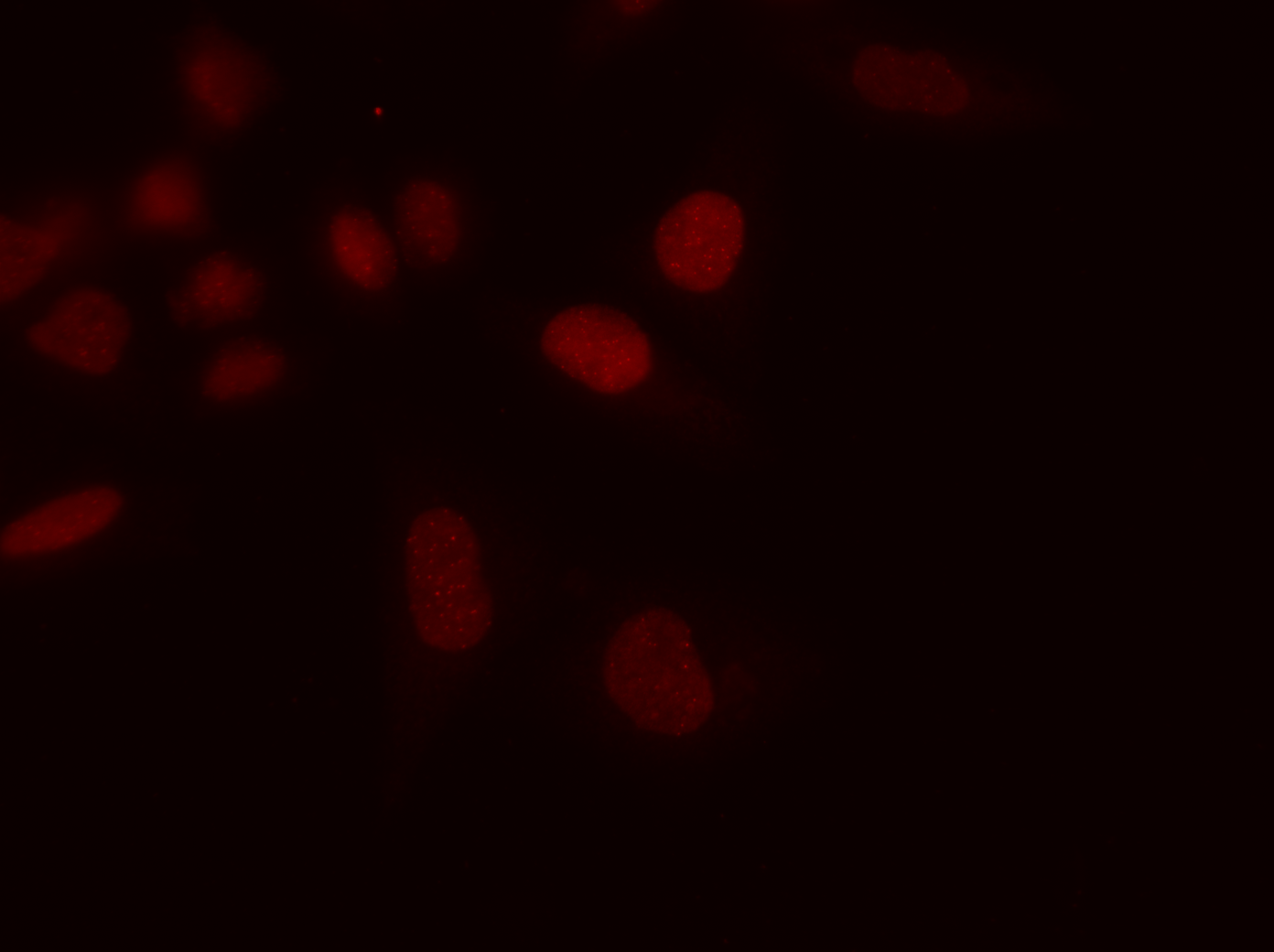

Supplement: Supplementary file 8 — Source data Fig. 4 [file 44318_2024_104_MOESM8_ESM.zip › Figure 4/4C/WT Anti-GFP.tif]

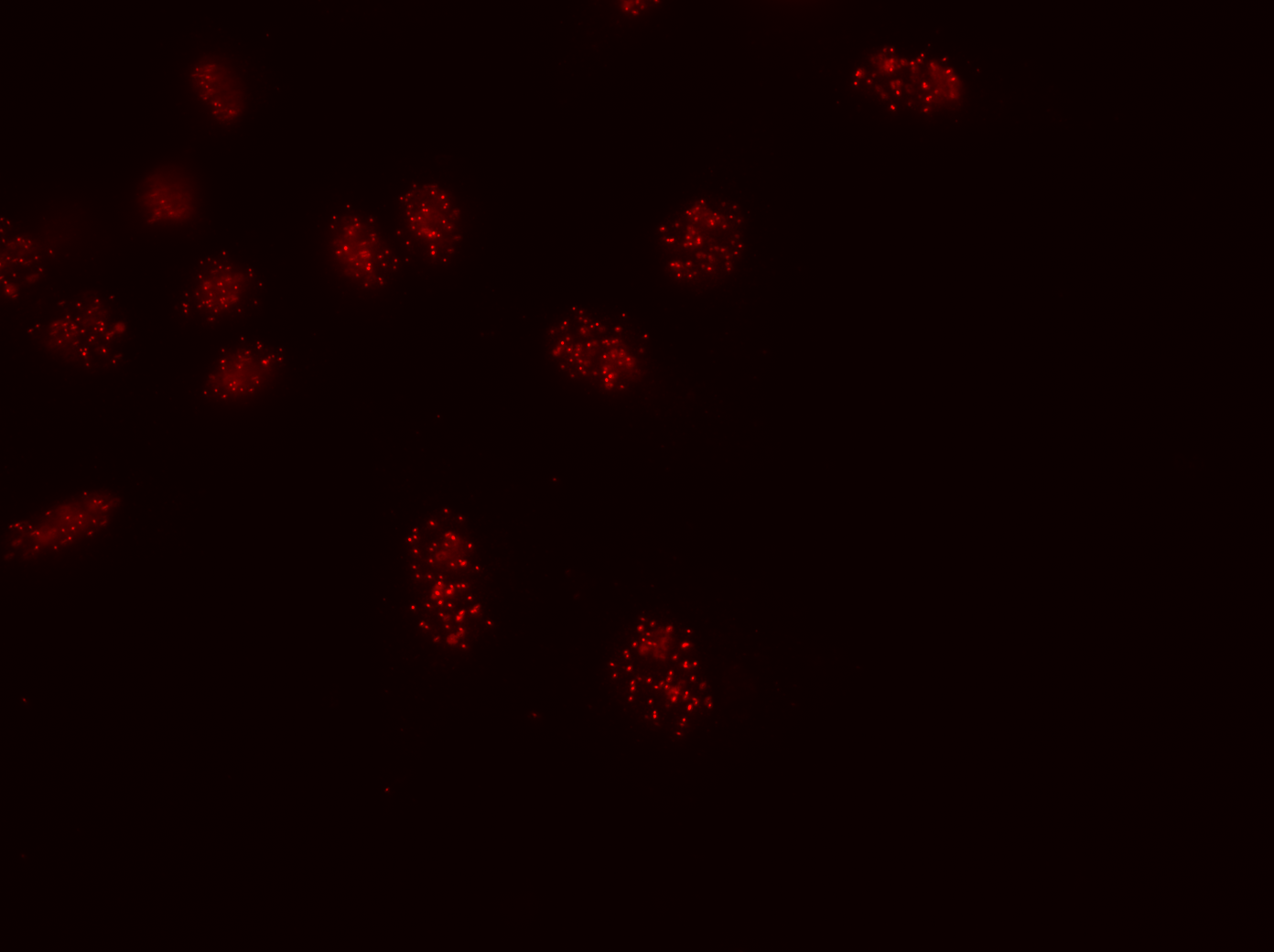

Supplement: Supplementary file 8 — Source data Fig. 4 [file 44318_2024_104_MOESM8_ESM.zip › Figure 4/4C/WT CENP-C.tif]

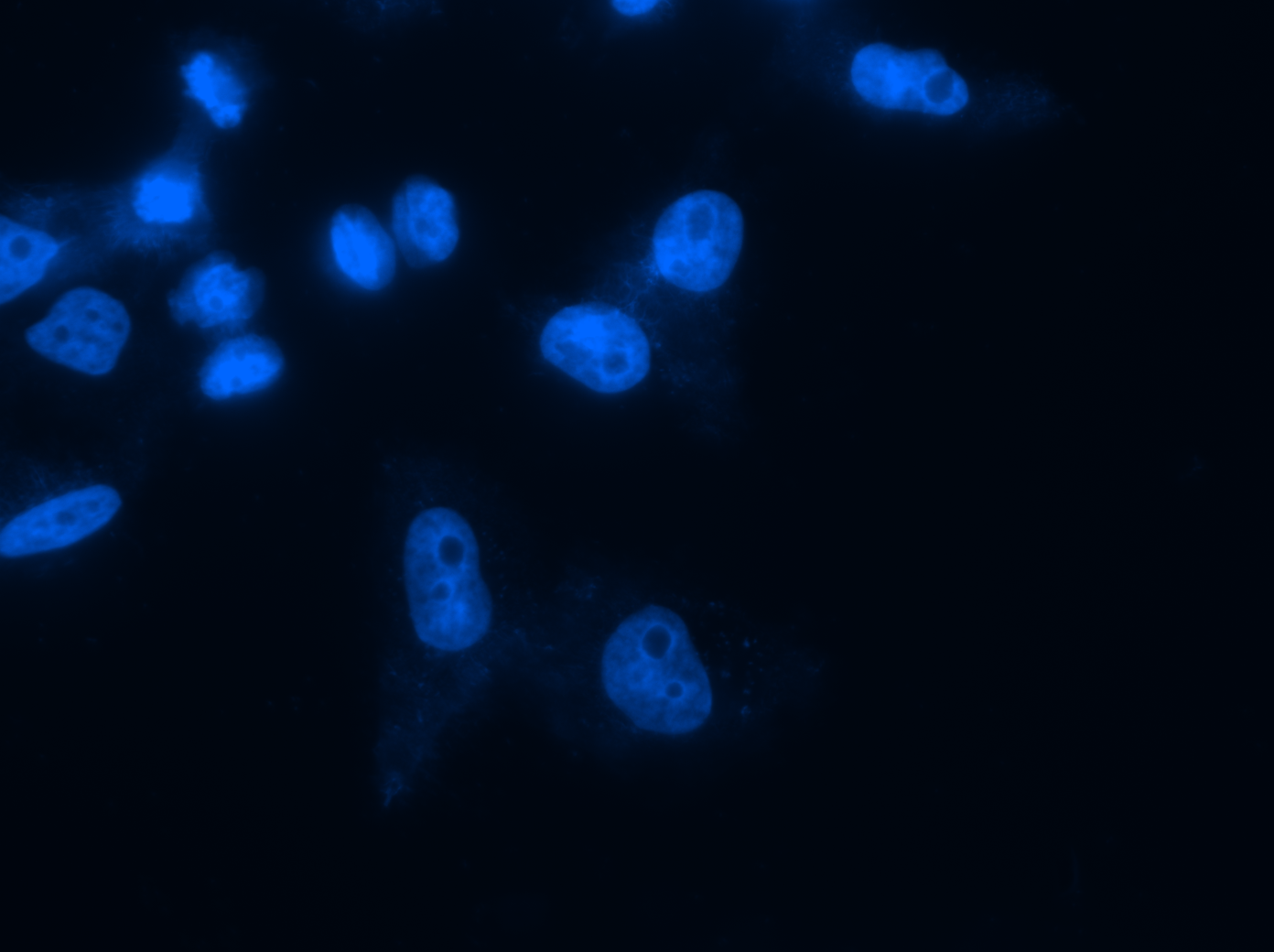

Supplement: Supplementary file 8 — Source data Fig. 4 [file 44318_2024_104_MOESM8_ESM.zip › Figure 4/4C/WT DNA.tif]

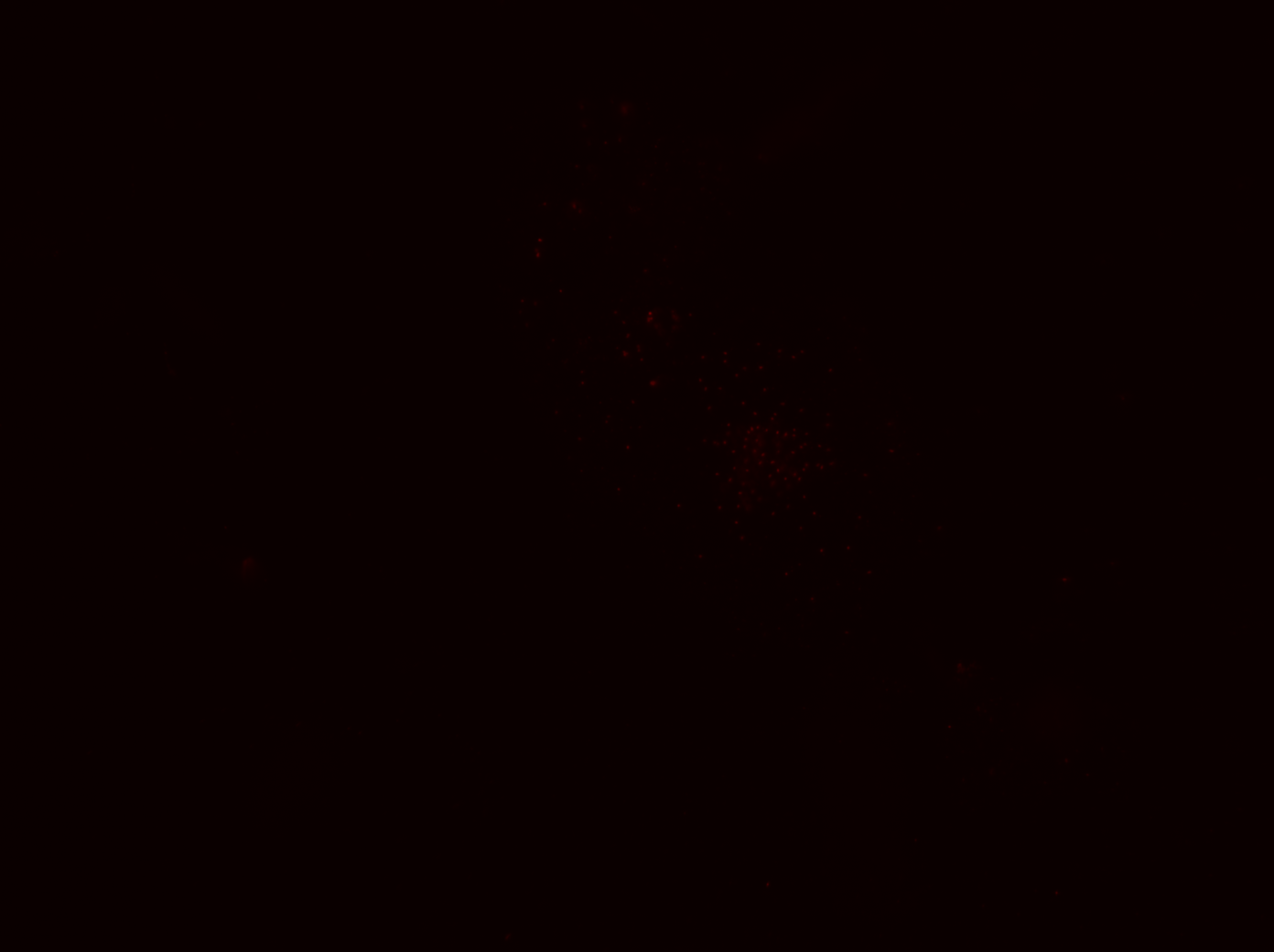

Supplement: Supplementary file 8 — Source data Fig. 4 [file 44318_2024_104_MOESM8_ESM.zip › Figure 4/4F/ADA siCENP-U#1 CENP-C.tif]

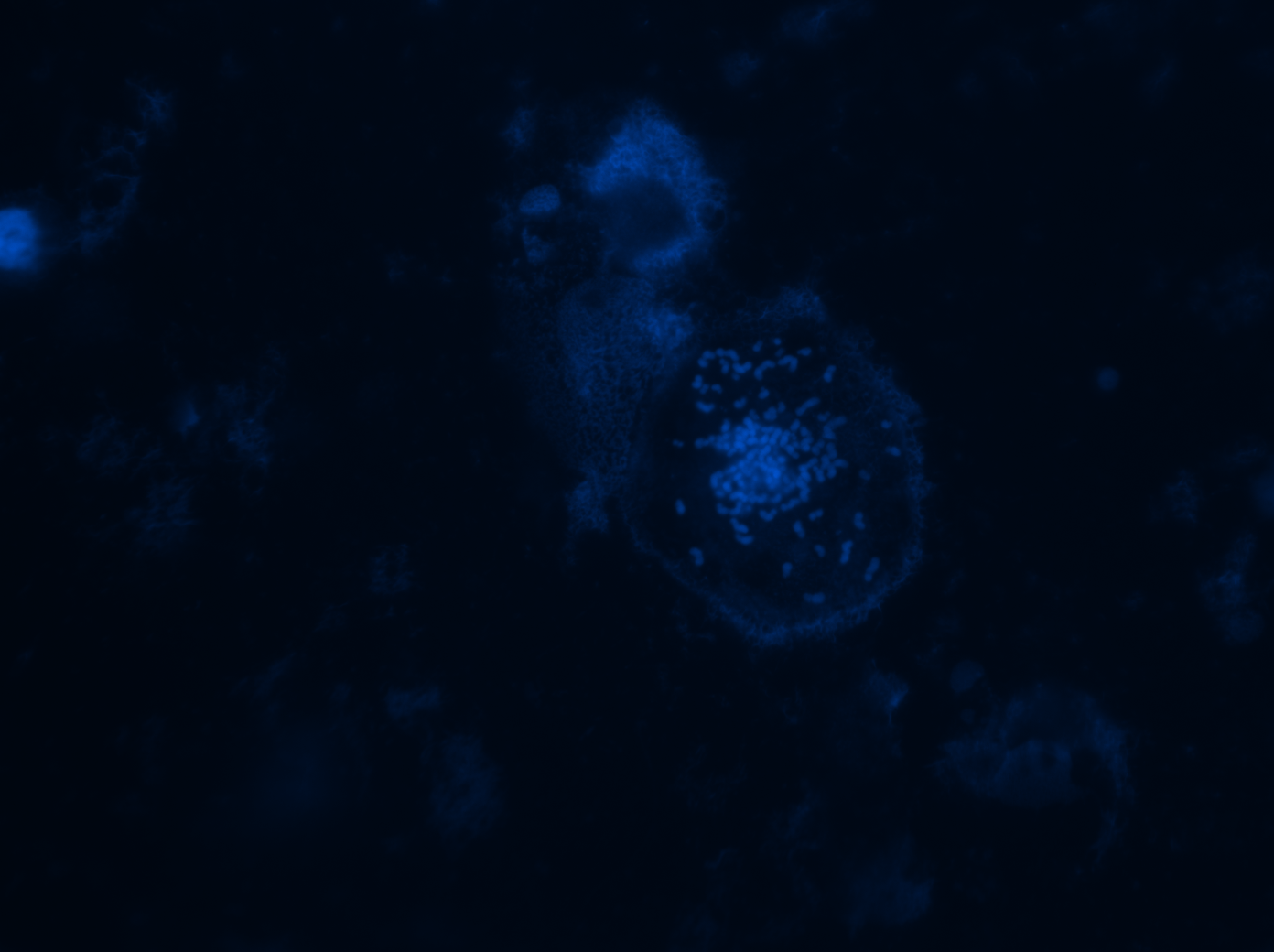

Supplement: Supplementary file 8 — Source data Fig. 4 [file 44318_2024_104_MOESM8_ESM.zip › Figure 4/4F/ADA siCENP-U#1 DNA1.tif]

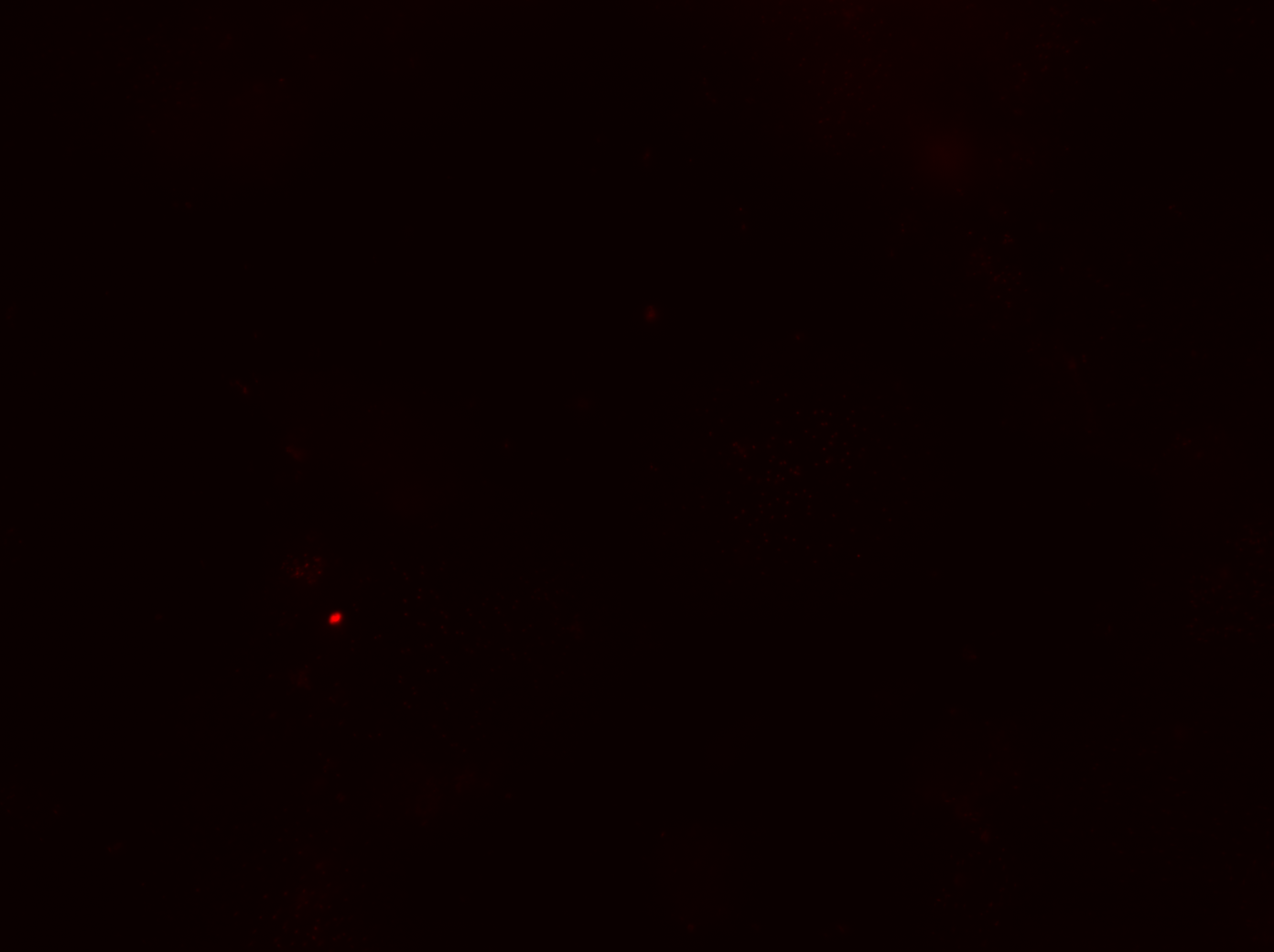

Supplement: Supplementary file 8 — Source data Fig. 4 [file 44318_2024_104_MOESM8_ESM.zip › Figure 4/4F/HeLa siCENP-U#1 CENP-C.tif]

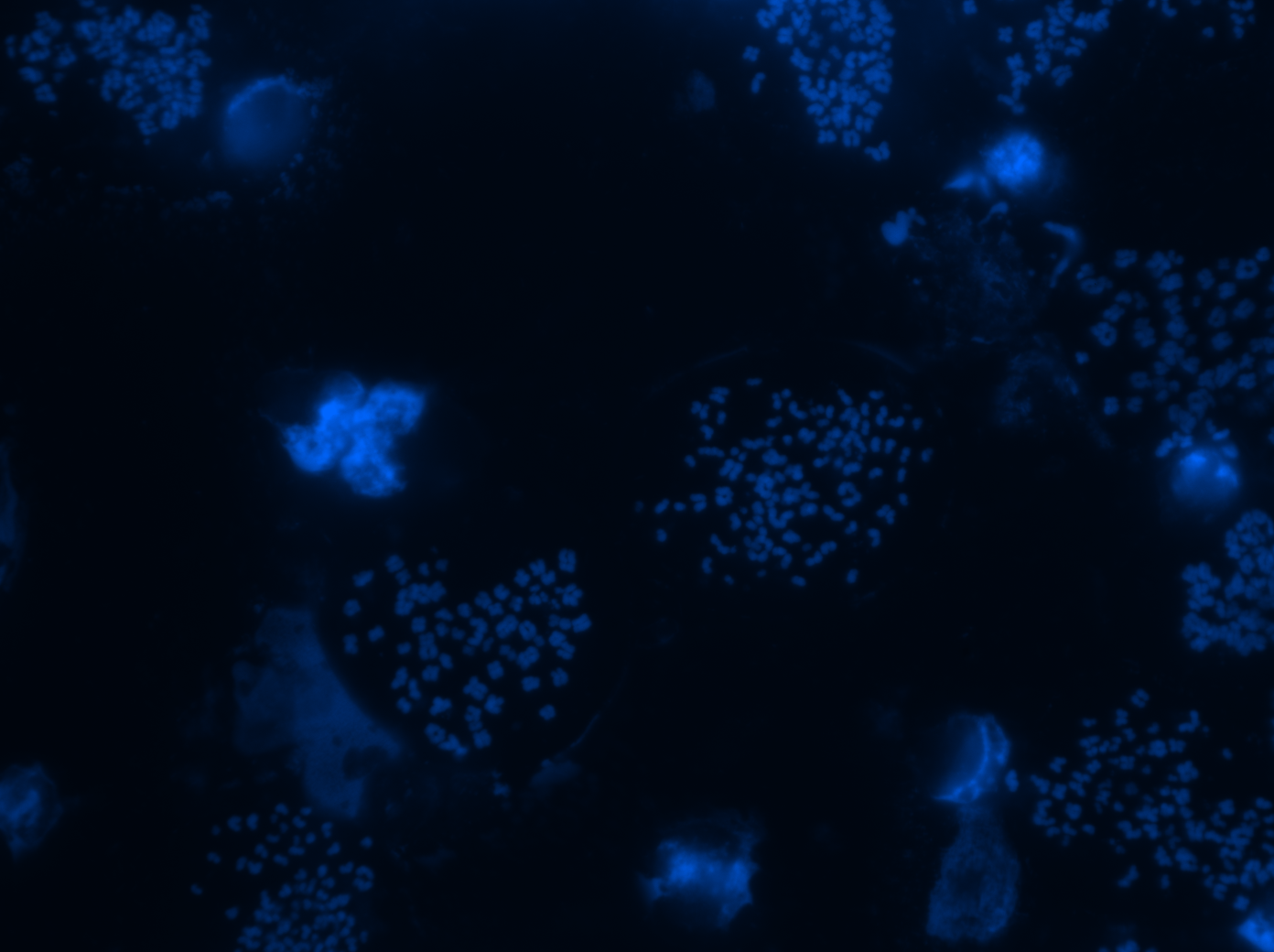

Supplement: Supplementary file 8 — Source data Fig. 4 [file 44318_2024_104_MOESM8_ESM.zip › Figure 4/4F/HeLa siCENP-U#1 DNA.tif]

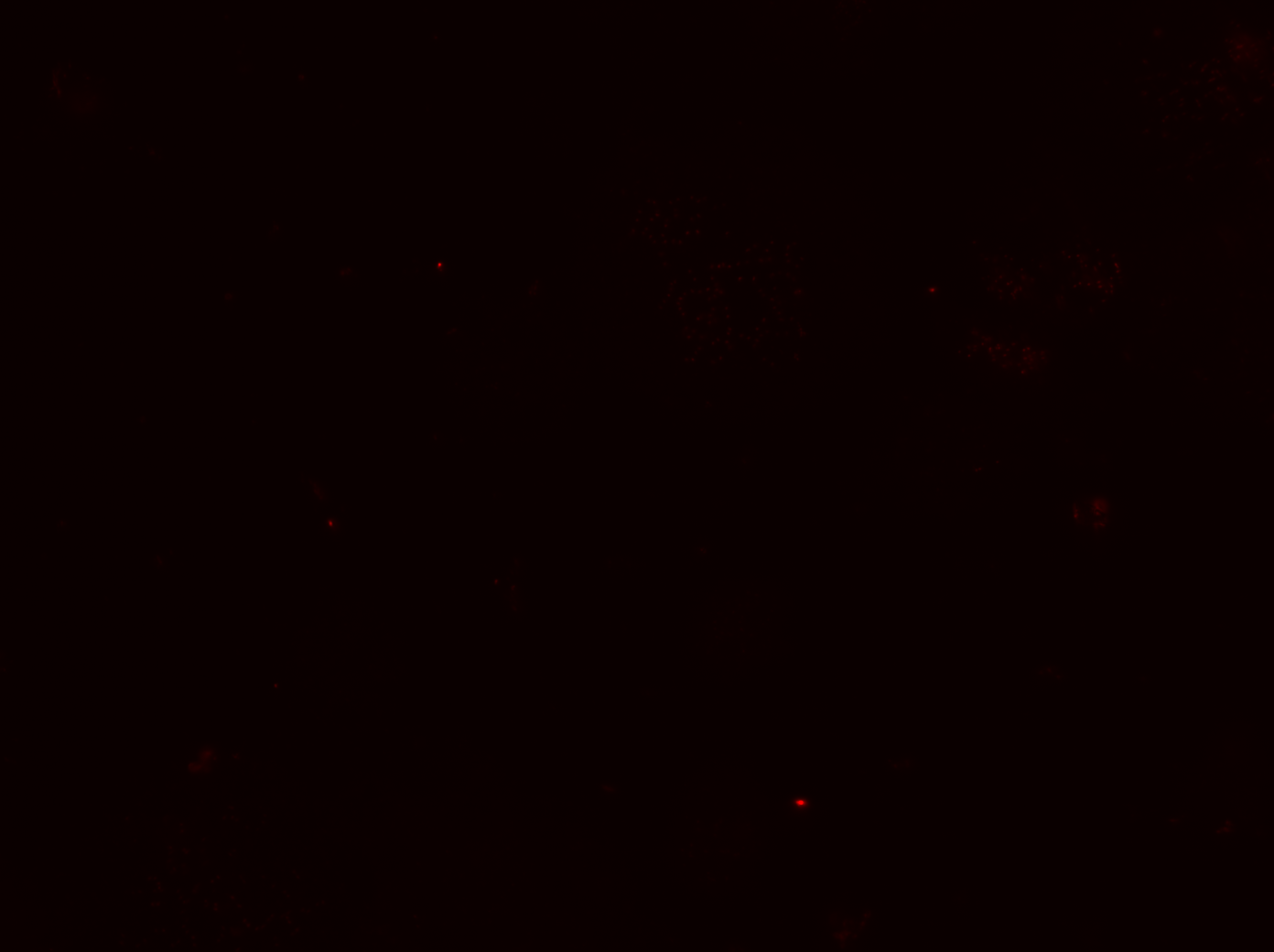

Supplement: Supplementary file 8 — Source data Fig. 4 [file 44318_2024_104_MOESM8_ESM.zip › Figure 4/4F/HeLa sicontrol CENP-C.tif]

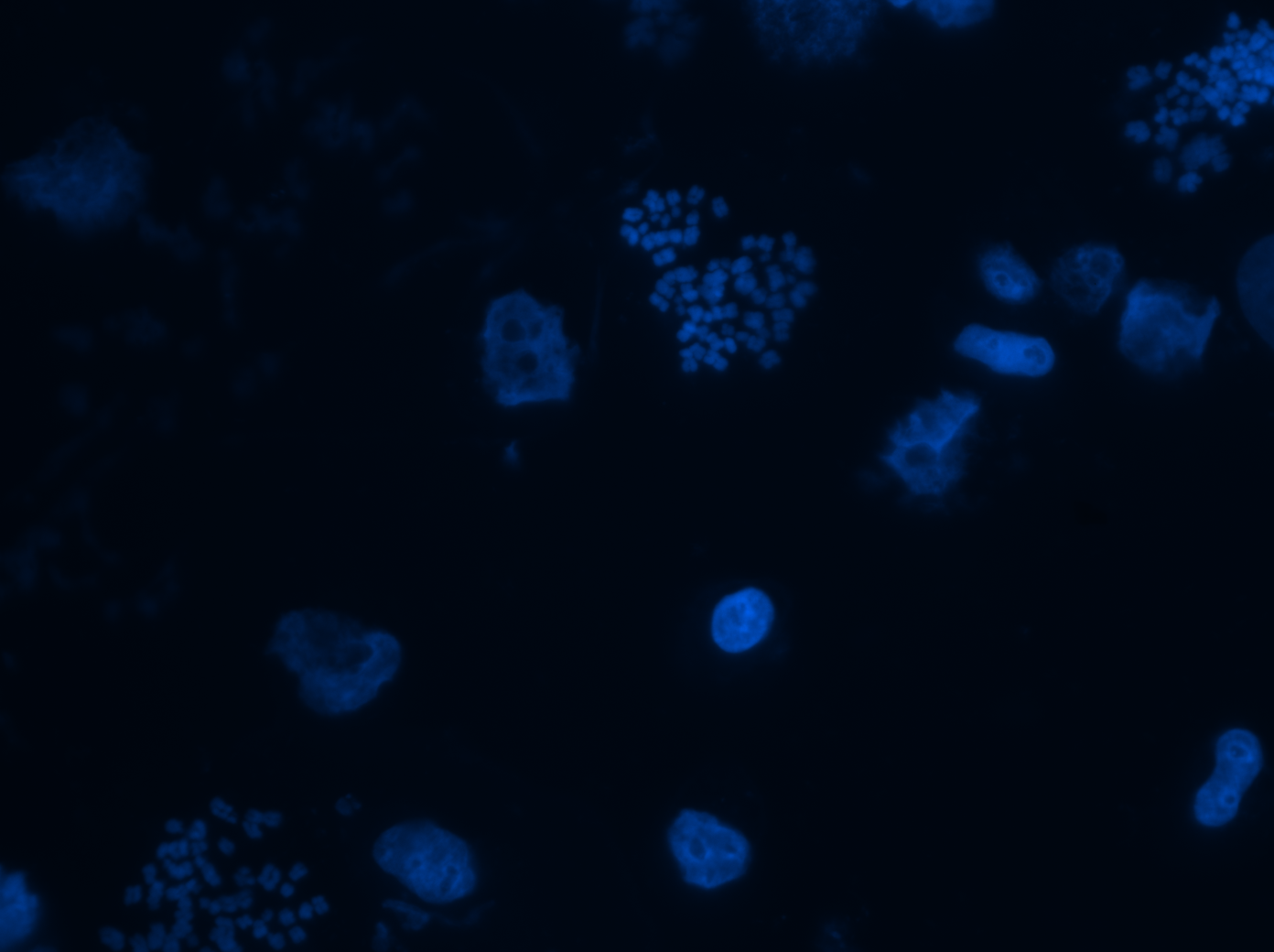

Supplement: Supplementary file 8 — Source data Fig. 4 [file 44318_2024_104_MOESM8_ESM.zip › Figure 4/4F/HeLa sicontrol DNA.tif]

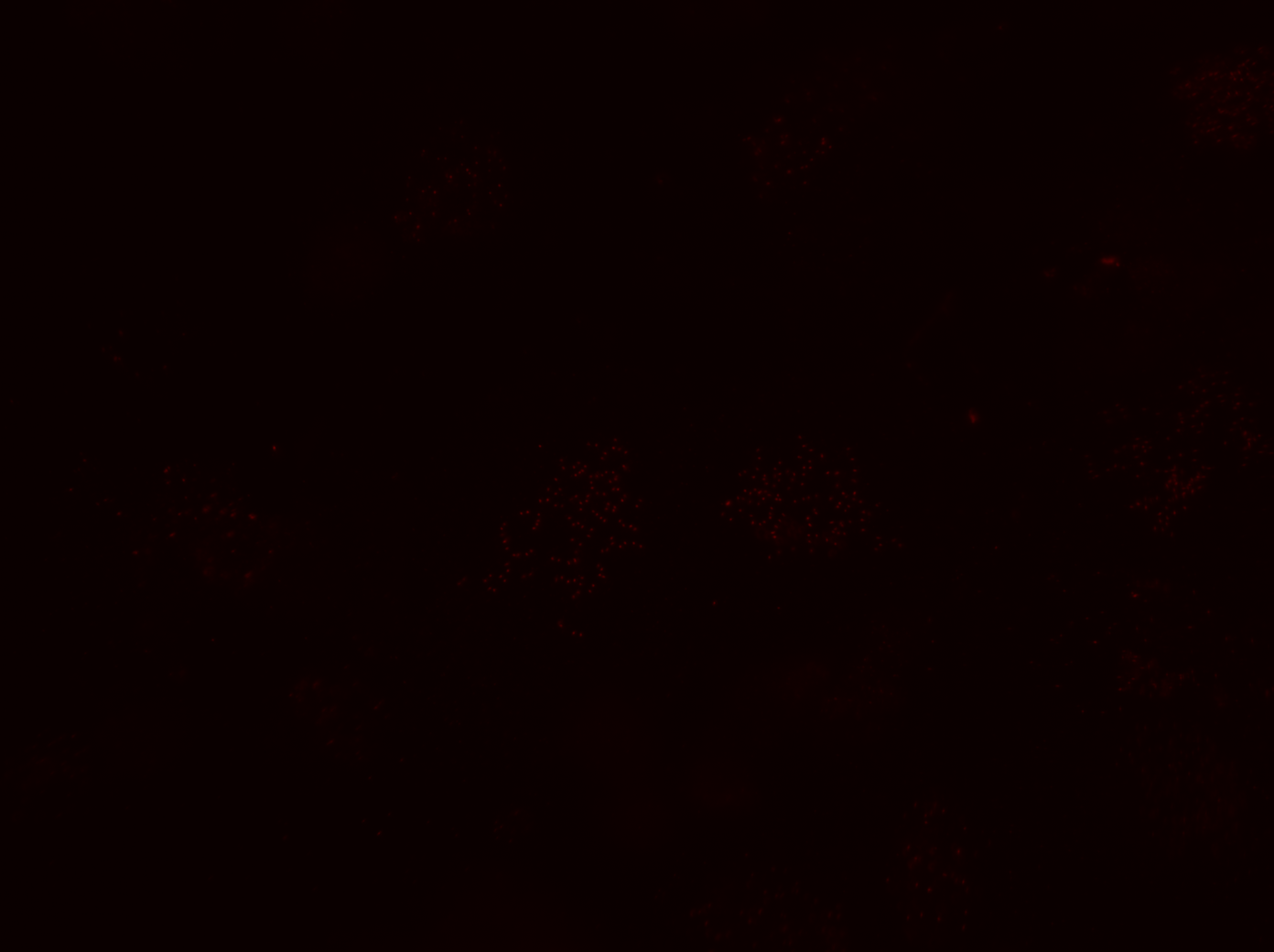

Supplement: Supplementary file 8 — Source data Fig. 4 [file 44318_2024_104_MOESM8_ESM.zip › Figure 4/4F/WT siCENP-U#1 CENP-C.tif]

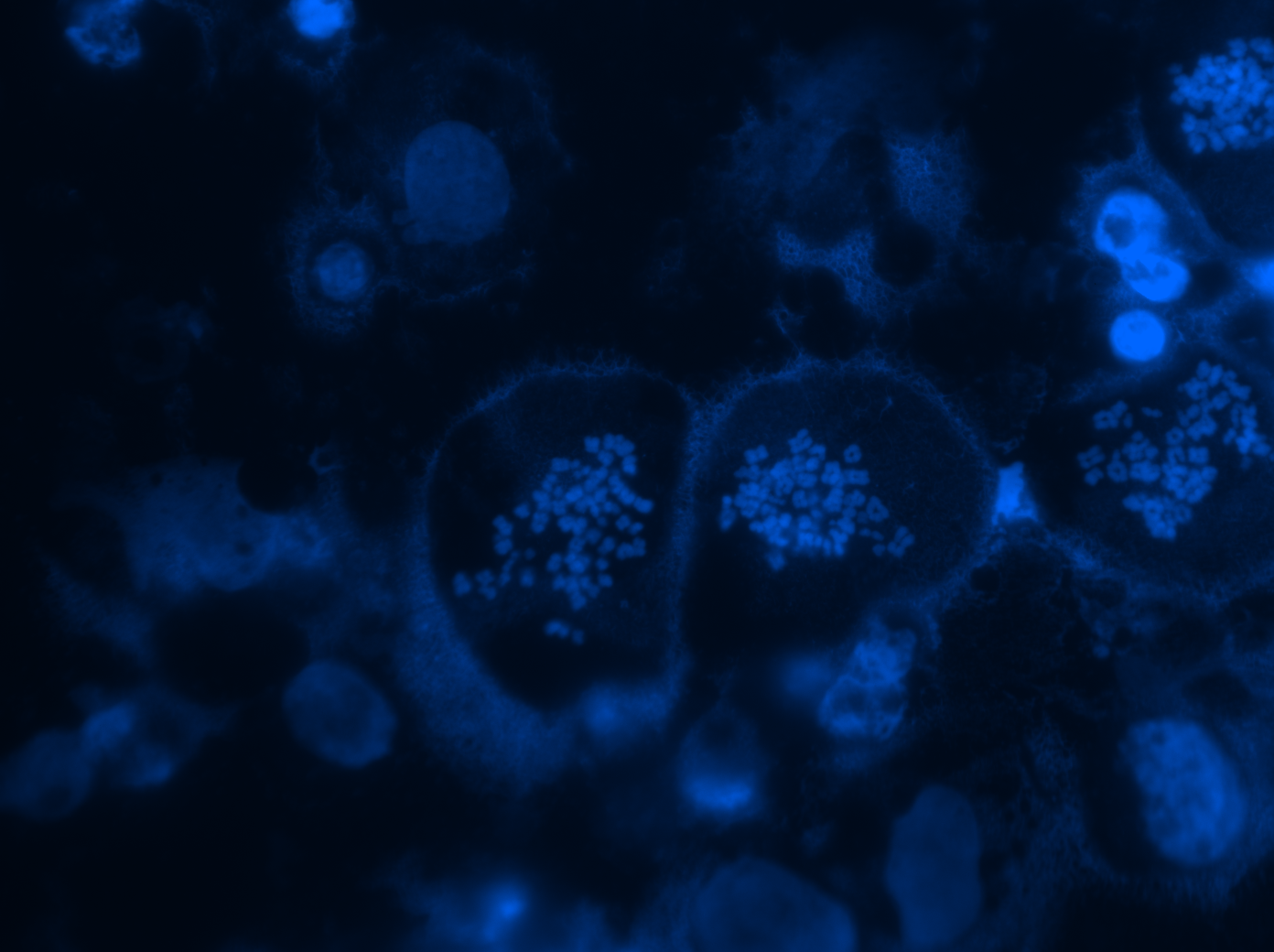

Supplement: Supplementary file 8 — Source data Fig. 4 [file 44318_2024_104_MOESM8_ESM.zip › Figure 4/4F/WT siCENP-U#1 DNA.tif]

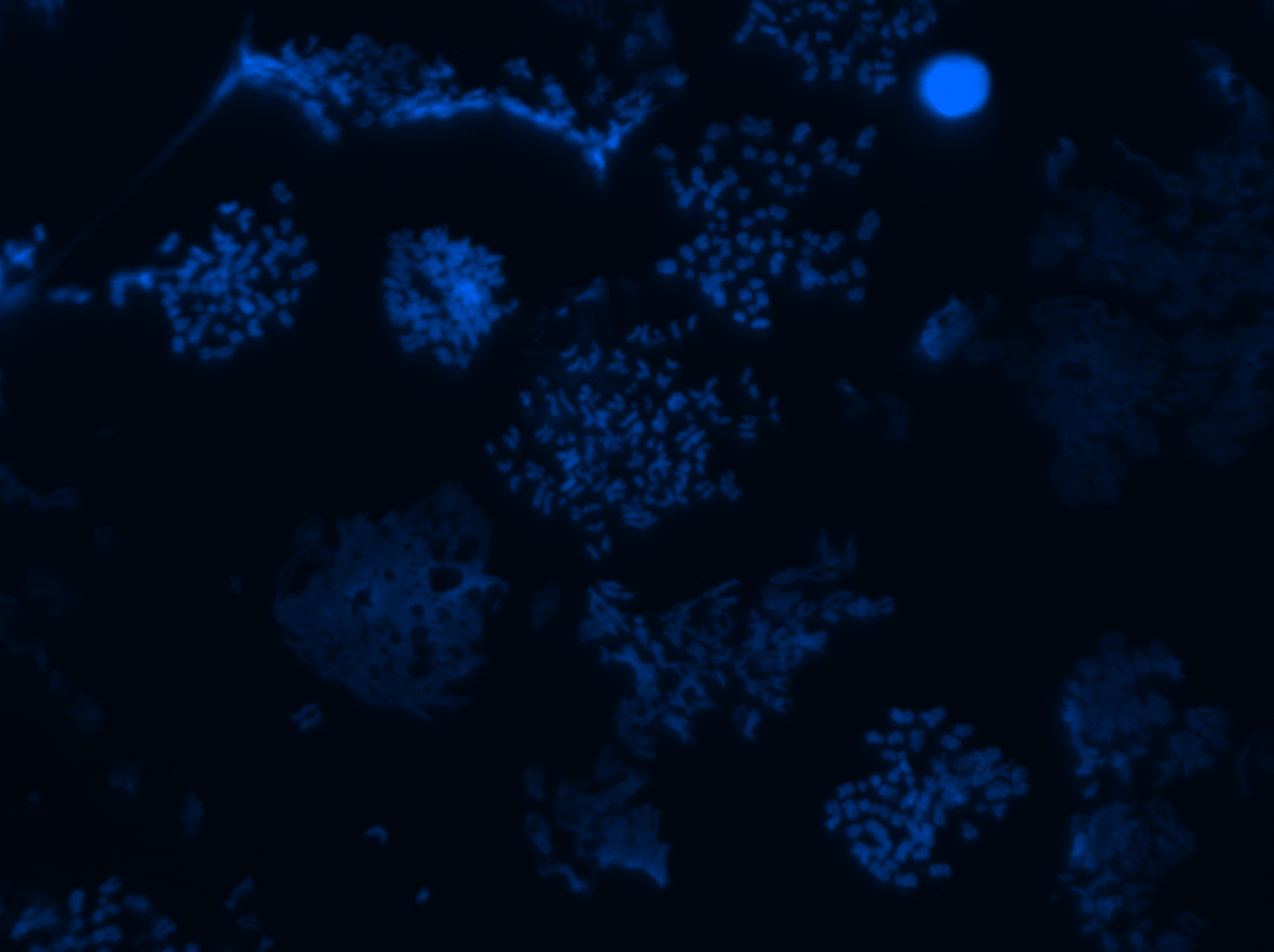

Supplement: Supplementary file 8 — Source data Fig. 4 [file 44318_2024_104_MOESM8_ESM.zip › Figure 4/4I/CENP-U-AVAAA 010c1.tif]

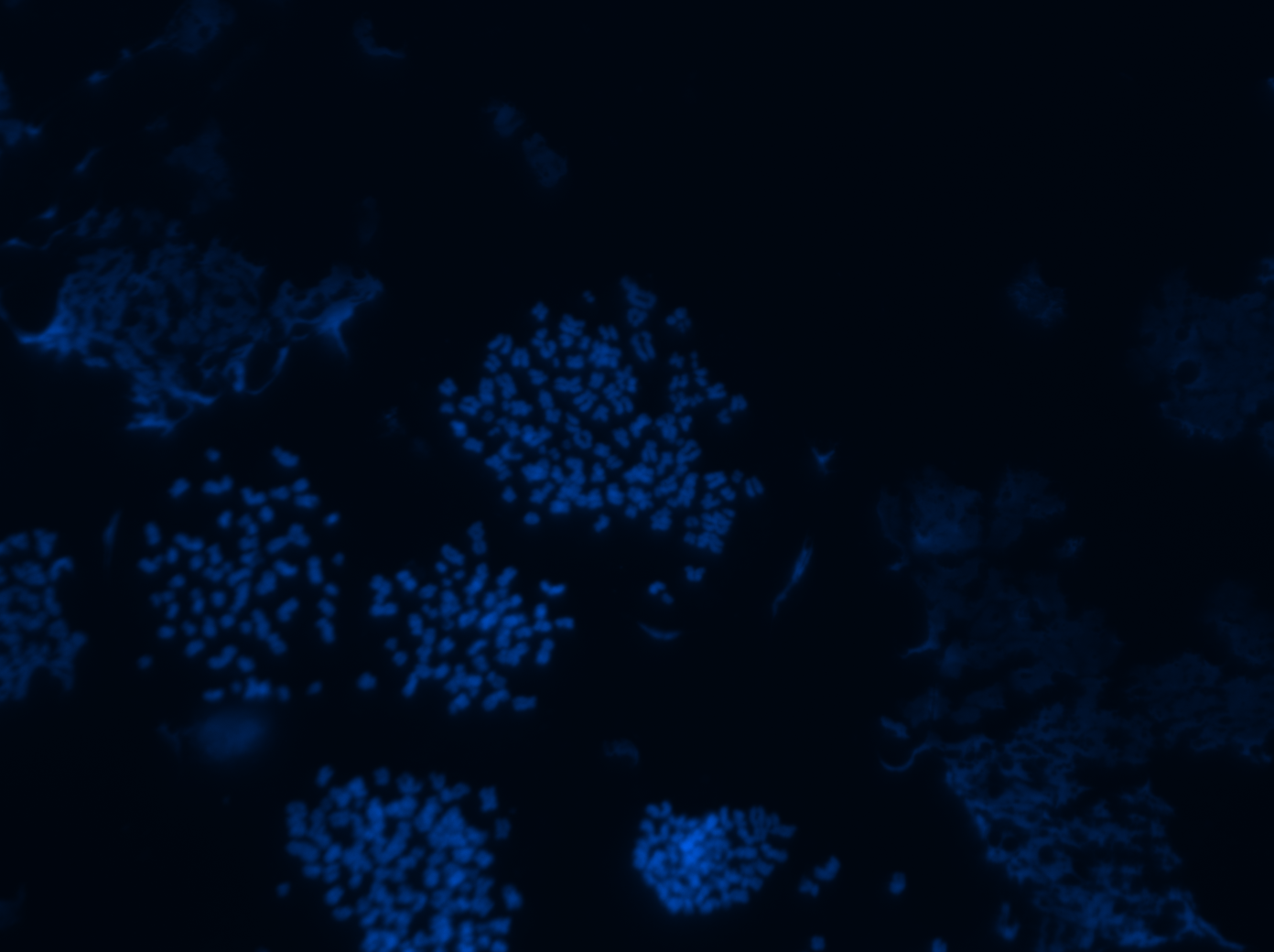

Supplement: Supplementary file 8 — Source data Fig. 4 [file 44318_2024_104_MOESM8_ESM.zip › Figure 4/4I/CENP-U-AVAAA 007c1.tif]

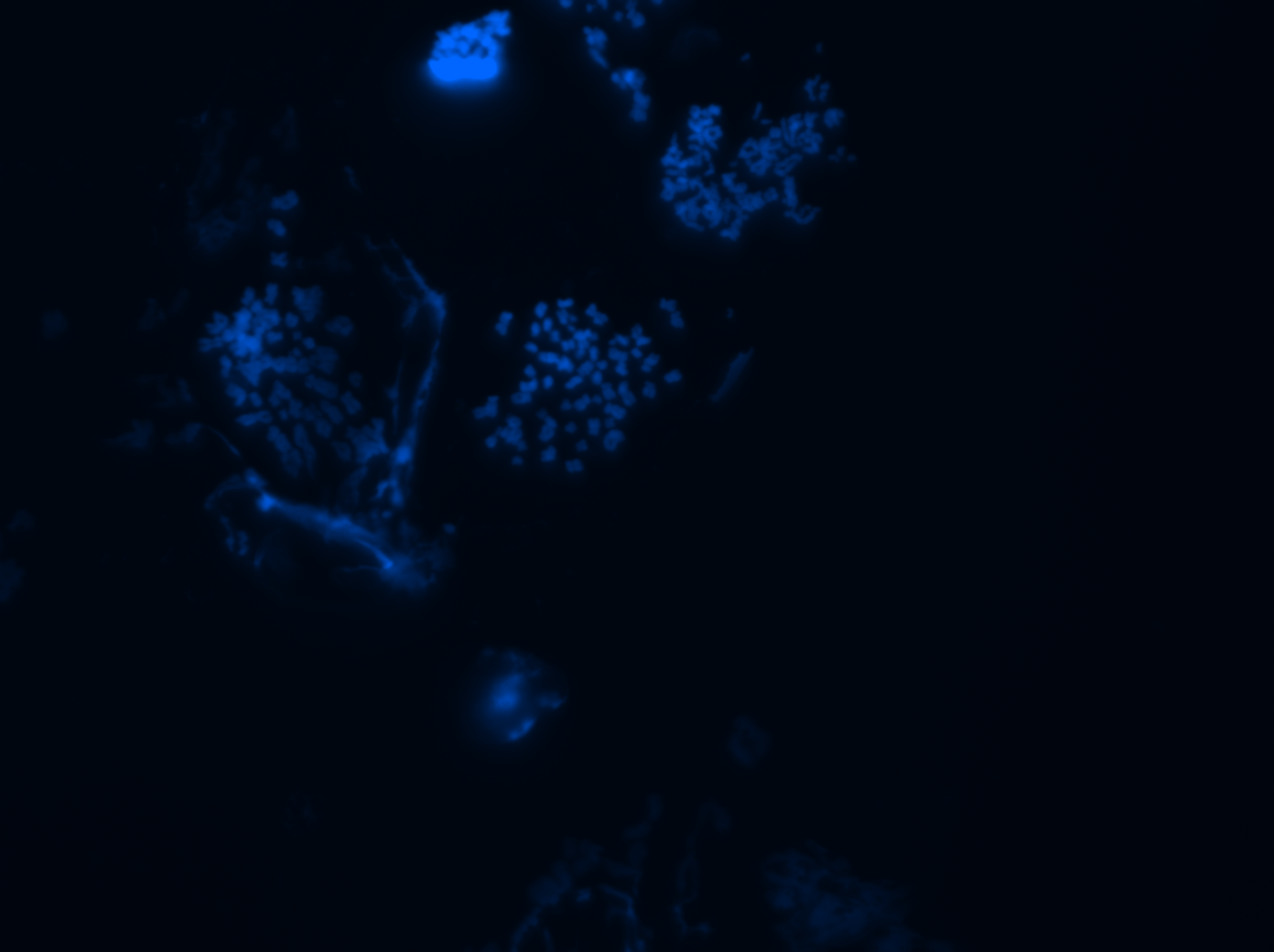

Supplement: Supplementary file 8 — Source data Fig. 4 [file 44318_2024_104_MOESM8_ESM.zip › Figure 4/4I/HELA 002c1.tif]

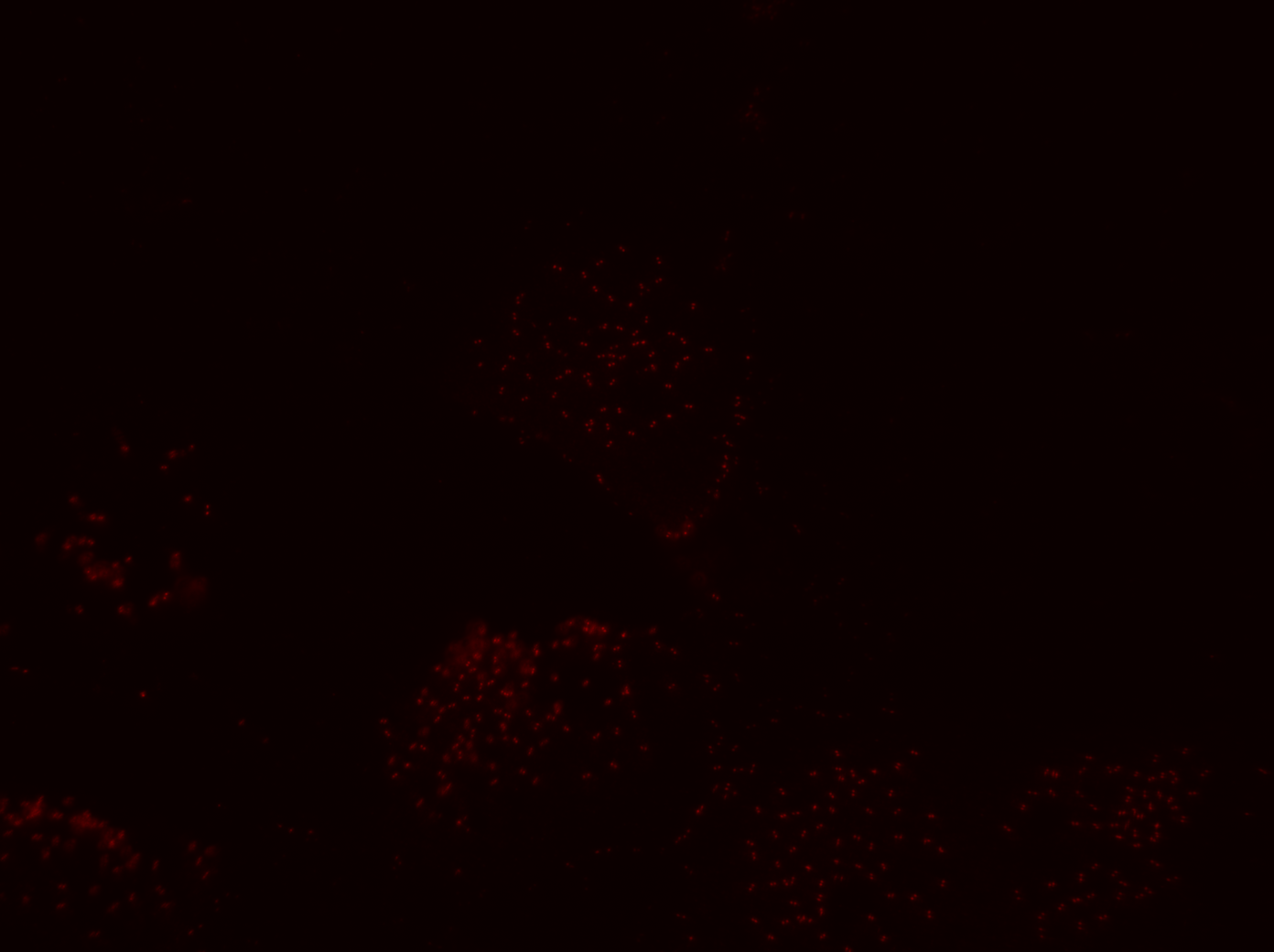

Supplement: Supplementary file 8 — Source data Fig. 4 [file 44318_2024_104_MOESM8_ESM.zip › Figure 4/4K/CENP-U-AVAAA-CENP-C.tif]

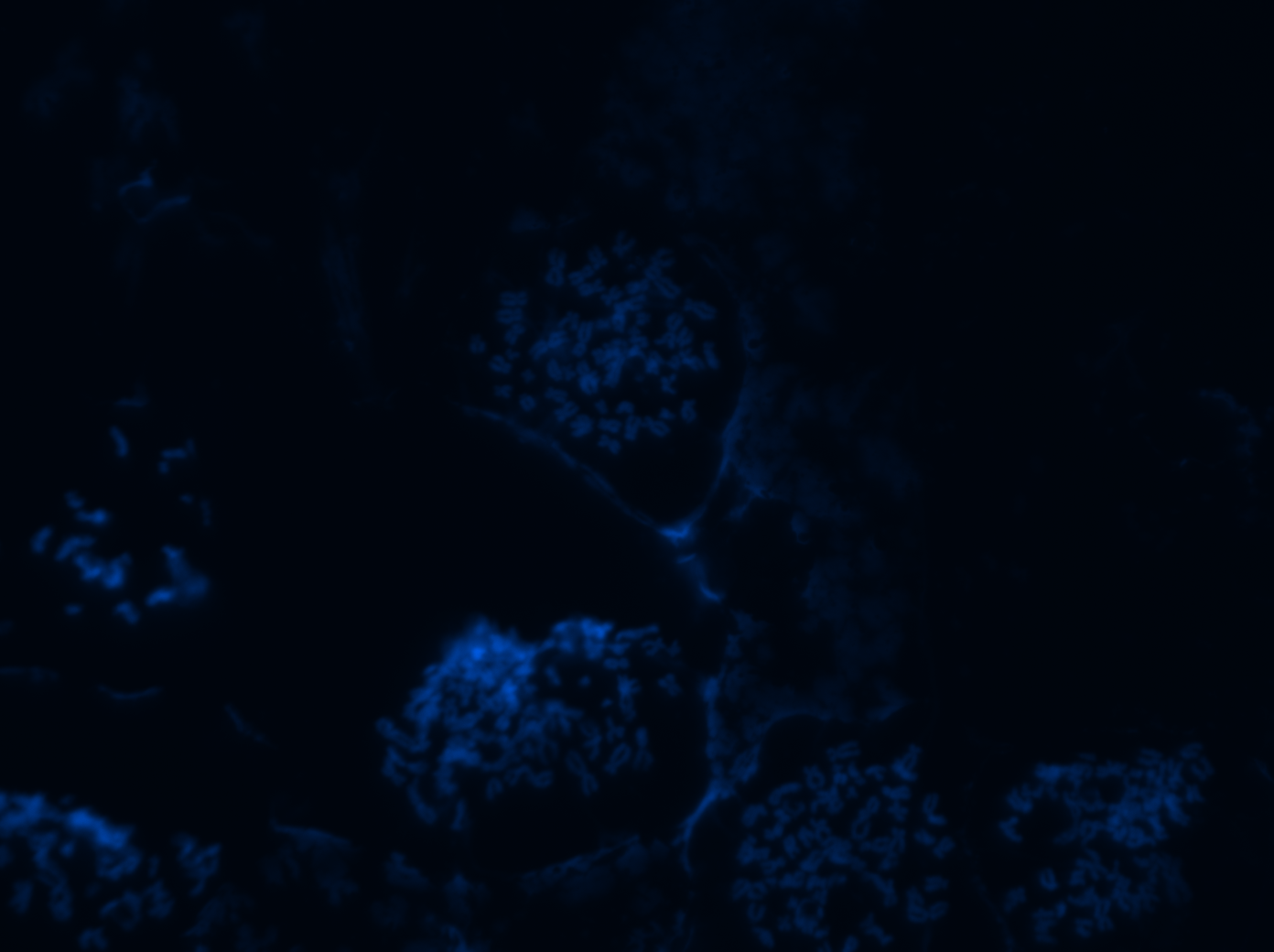

Supplement: Supplementary file 8 — Source data Fig. 4 [file 44318_2024_104_MOESM8_ESM.zip › Figure 4/4K/CENP-U-AVAAA-DNA.tif]

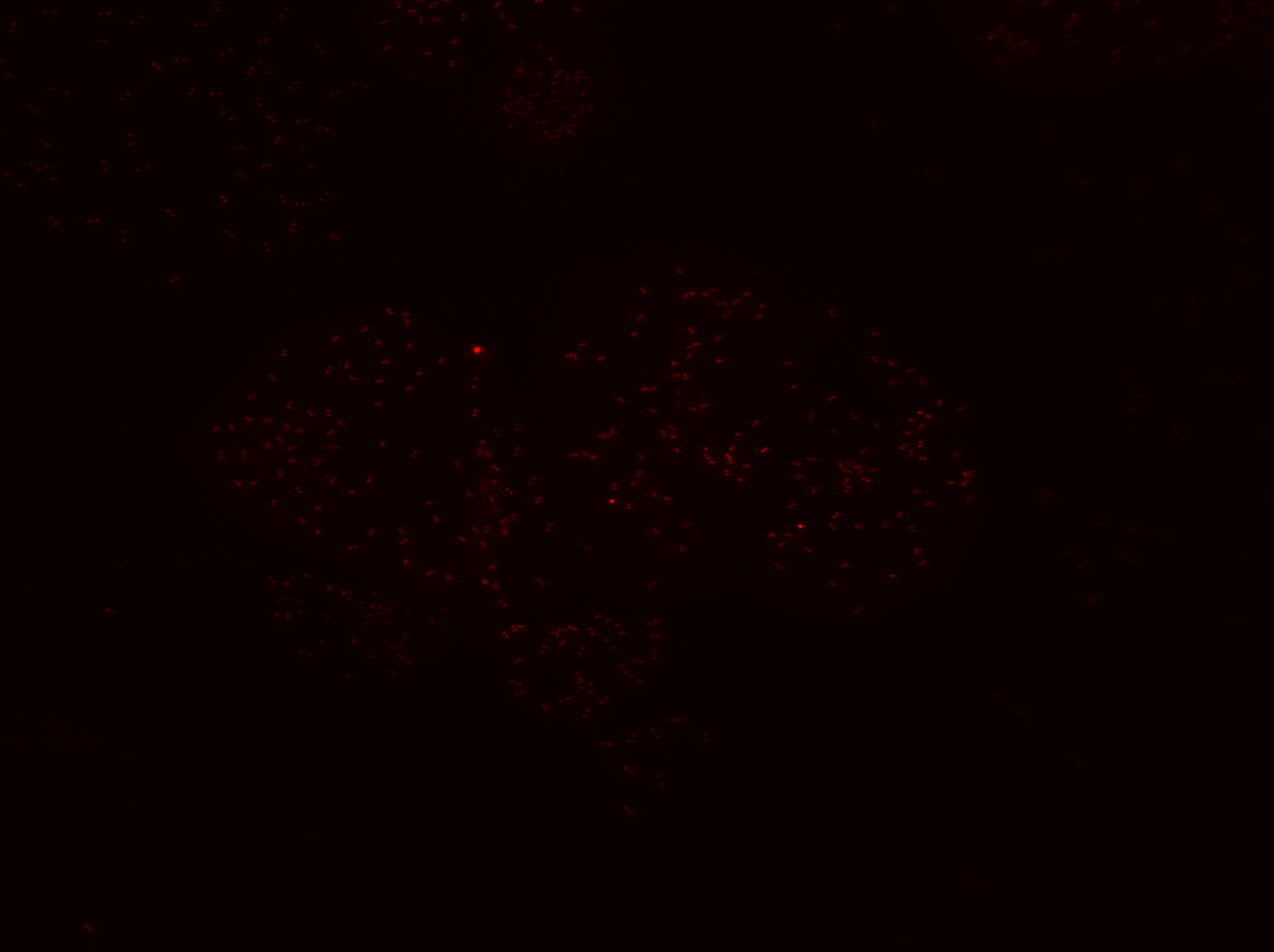

Supplement: Supplementary file 8 — Source data Fig. 4 [file 44318_2024_104_MOESM8_ESM.zip › Figure 4/4K/HeLa-CENP-C.tif]

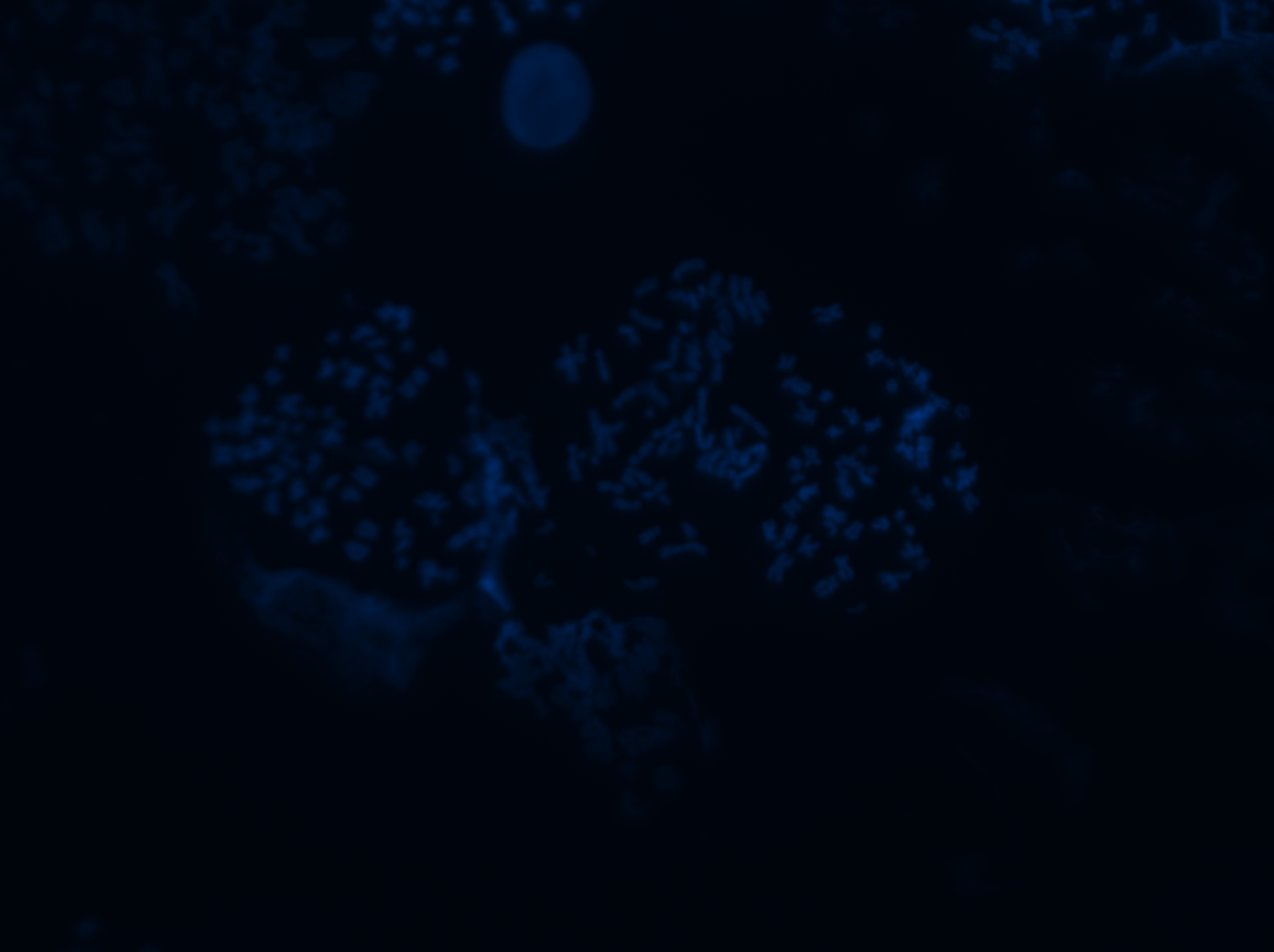

Supplement: Supplementary file 8 — Source data Fig. 4 [file 44318_2024_104_MOESM8_ESM.zip › Figure 4/4K/HeLa-DNA.tif]

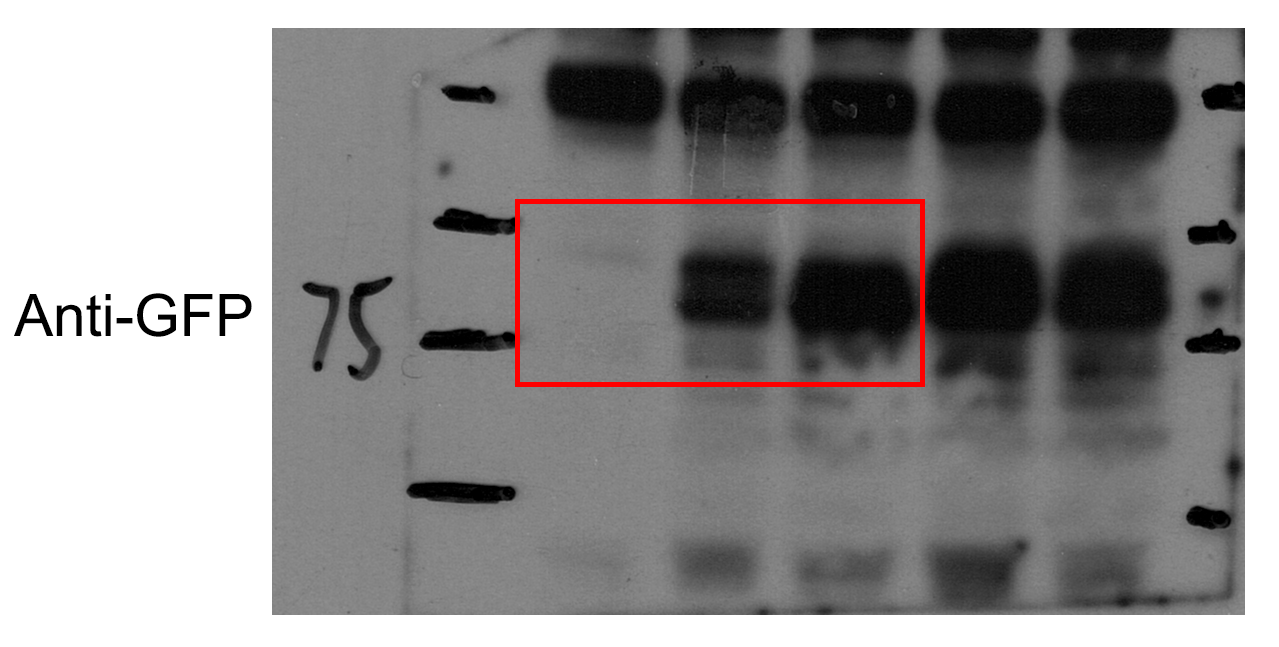

Supplement: Supplementary file 8 — Source data Fig. 4 [file 44318_2024_104_MOESM8_ESM.zip › Figure 4/4L/western Anti-GFP.tif]

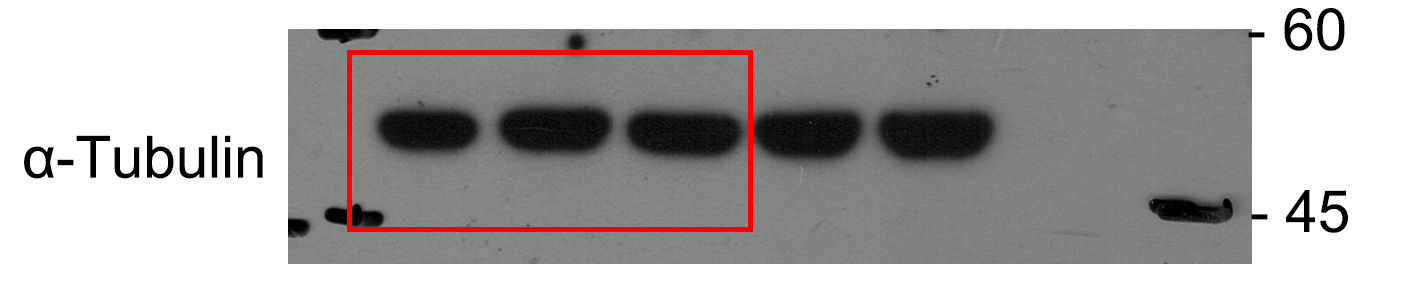

Supplement: Supplementary file 8 — Source data Fig. 4 [file 44318_2024_104_MOESM8_ESM.zip › Figure 4/4L/western a┴-Tubulin.tif]

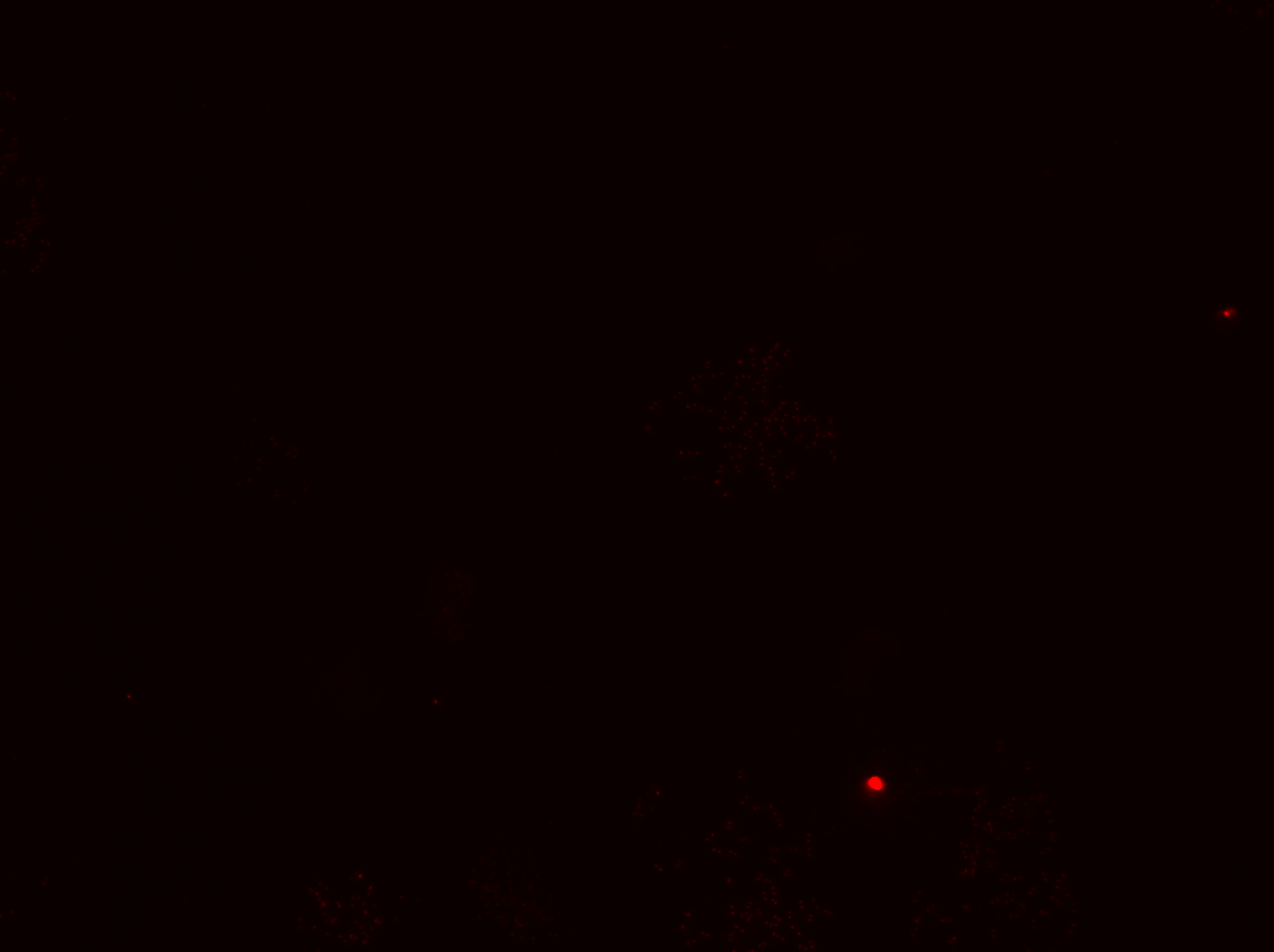

Supplement: Supplementary file 8 — Source data Fig. 4 [file 44318_2024_104_MOESM8_ESM.zip › Figure 4/4N/HeLa CENP-C.tif]

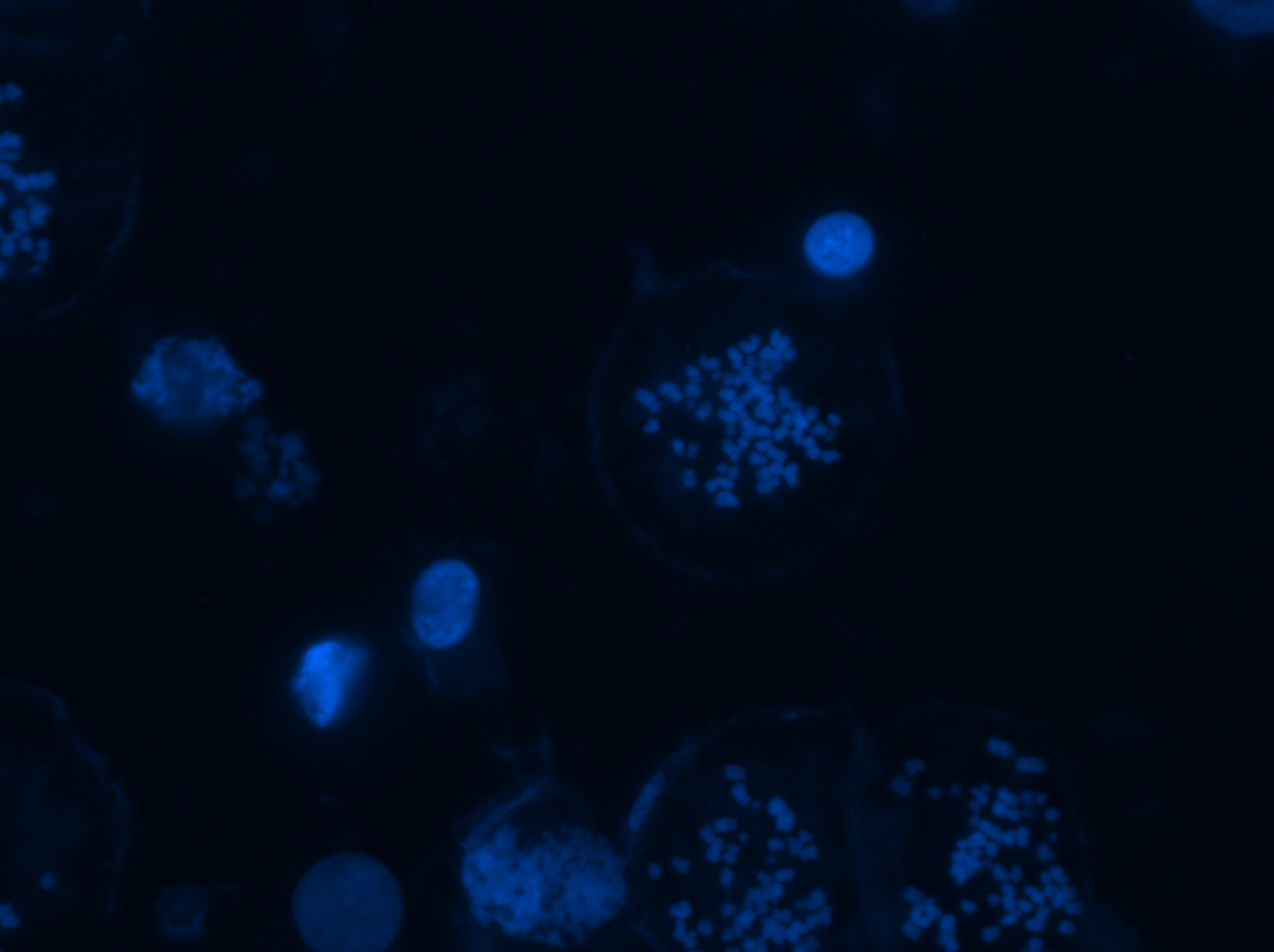

Supplement: Supplementary file 8 — Source data Fig. 4 [file 44318_2024_104_MOESM8_ESM.zip › Figure 4/4N/HeLa DNA.tif]

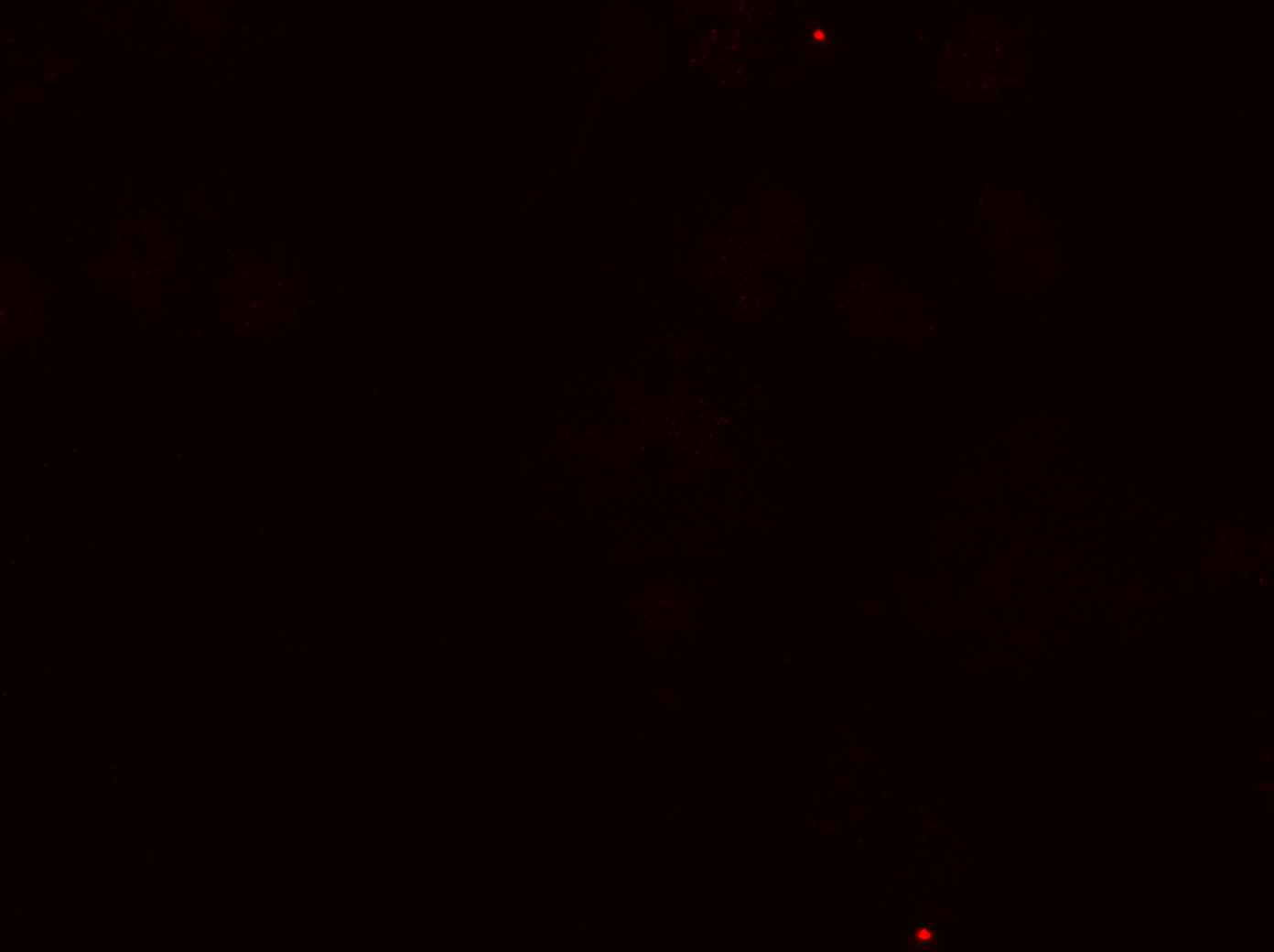

Supplement: Supplementary file 8 — Source data Fig. 4 [file 44318_2024_104_MOESM8_ESM.zip › Figure 4/4N/HeLa+siCENP-U#1 CENP-C.tif]

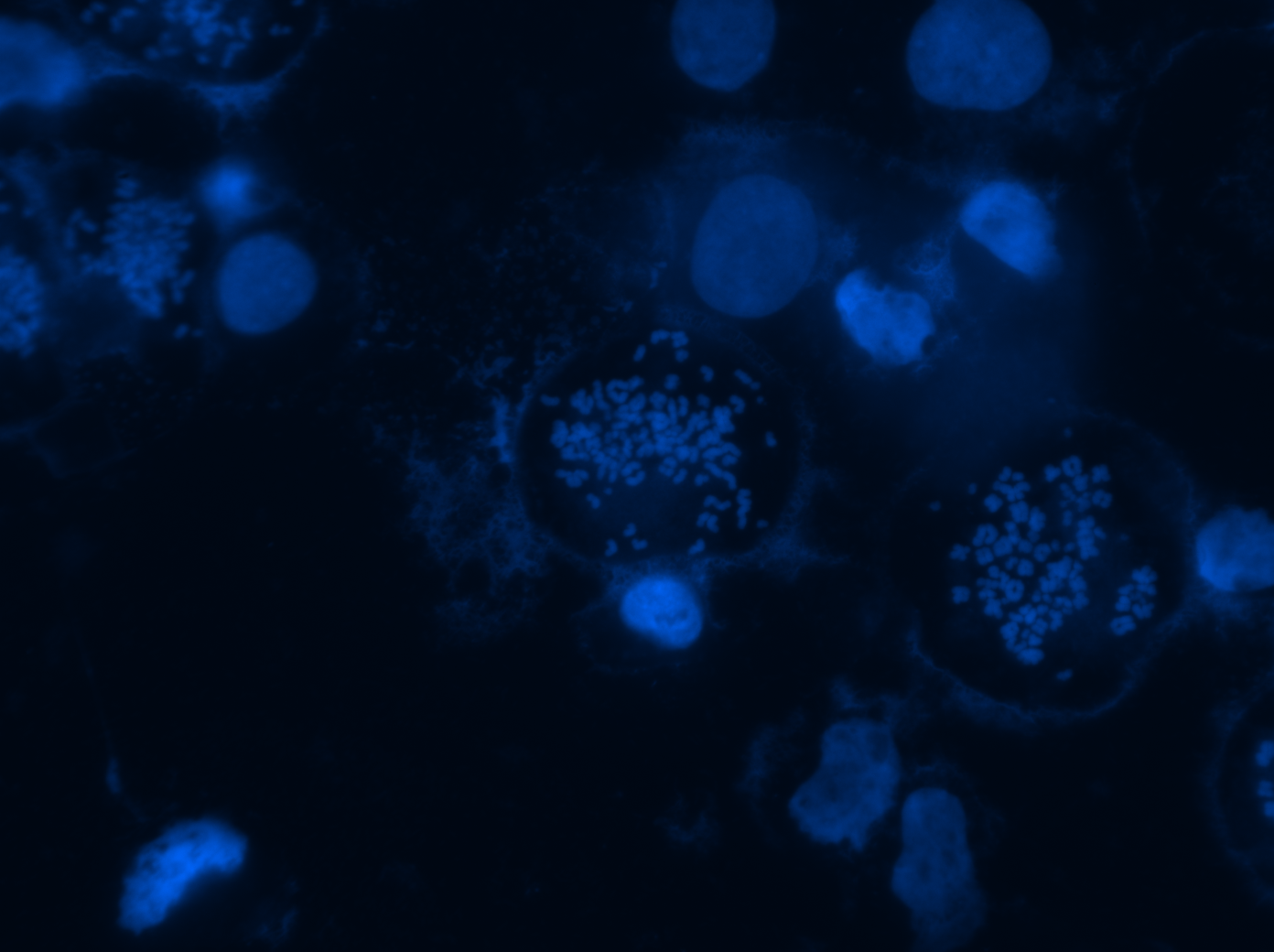

Supplement: Supplementary file 8 — Source data Fig. 4 [file 44318_2024_104_MOESM8_ESM.zip › Figure 4/4N/HeLa+siCENP-U#1 DNA.tif]

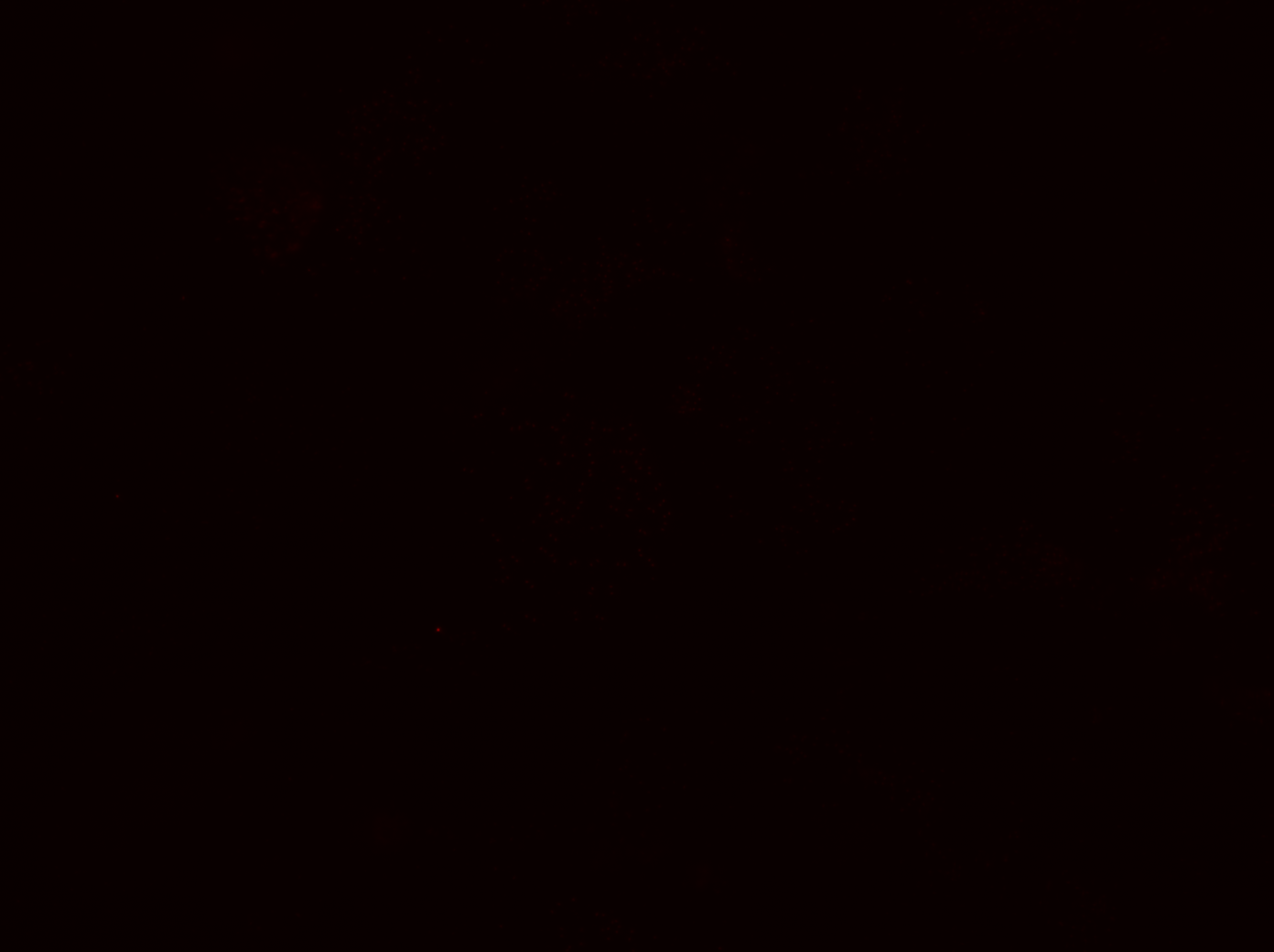

Supplement: Supplementary file 8 — Source data Fig. 4 [file 44318_2024_104_MOESM8_ESM.zip › Figure 4/4N/T78A #C2+siCENP-U#1 CENP-C.tif]

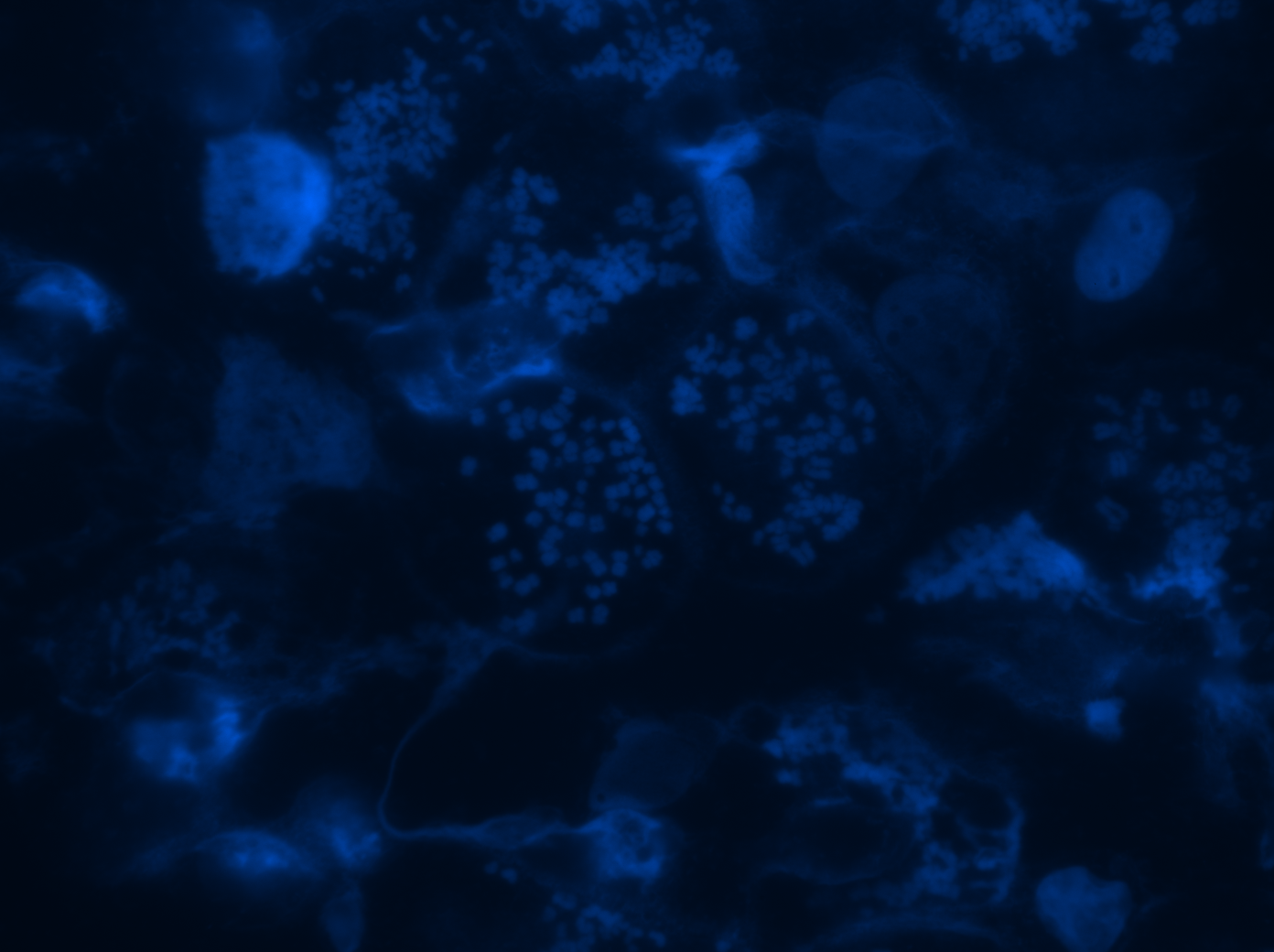

Supplement: Supplementary file 8 — Source data Fig. 4 [file 44318_2024_104_MOESM8_ESM.zip › Figure 4/4N/T78A #C2+siCENP-U#1 DNA.tif]

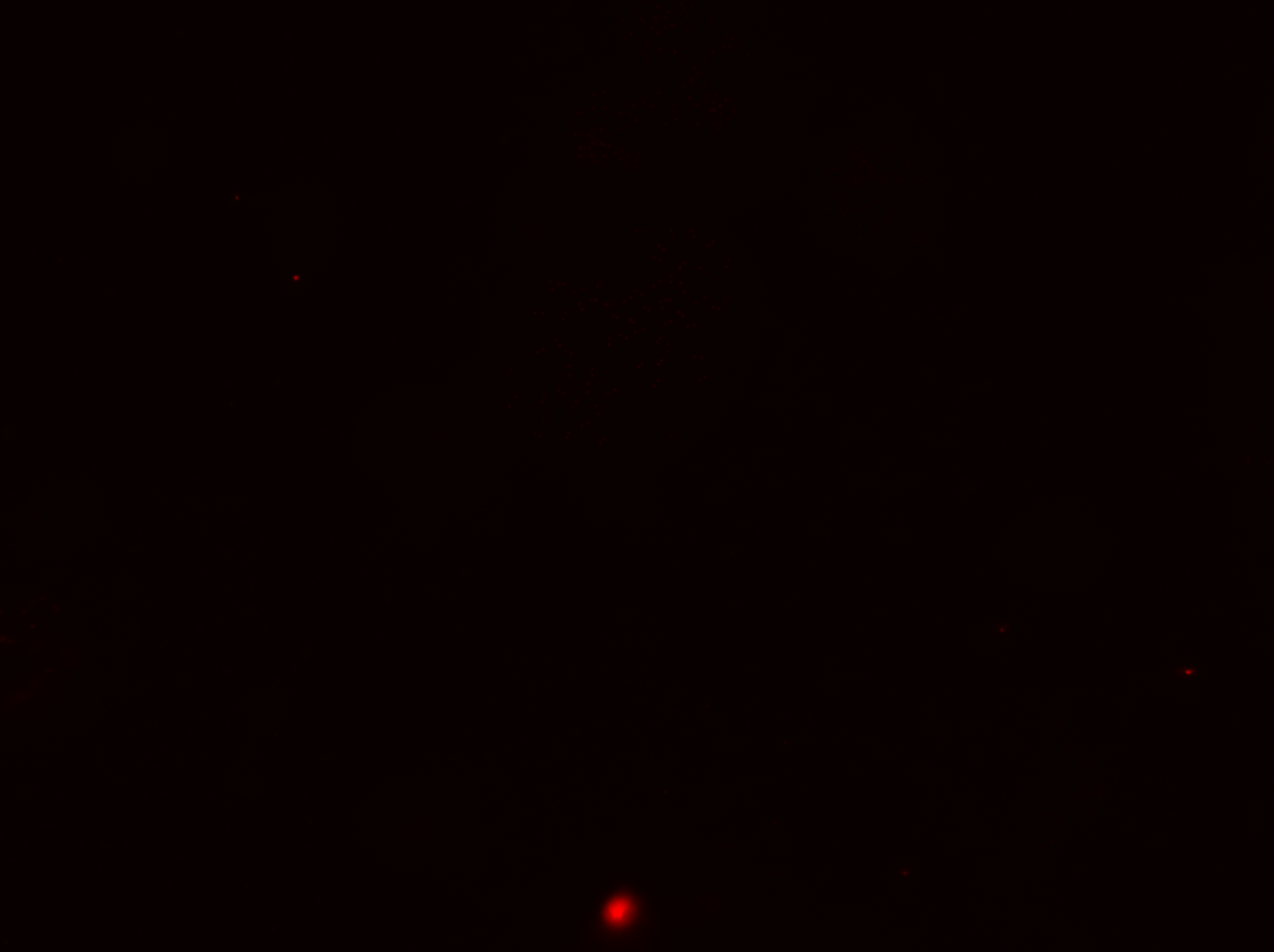

Supplement: Supplementary file 8 — Source data Fig. 4 [file 44318_2024_104_MOESM8_ESM.zip › Figure 4/4N/WT#A3+siCENP-U#1 CENP-C.tif]

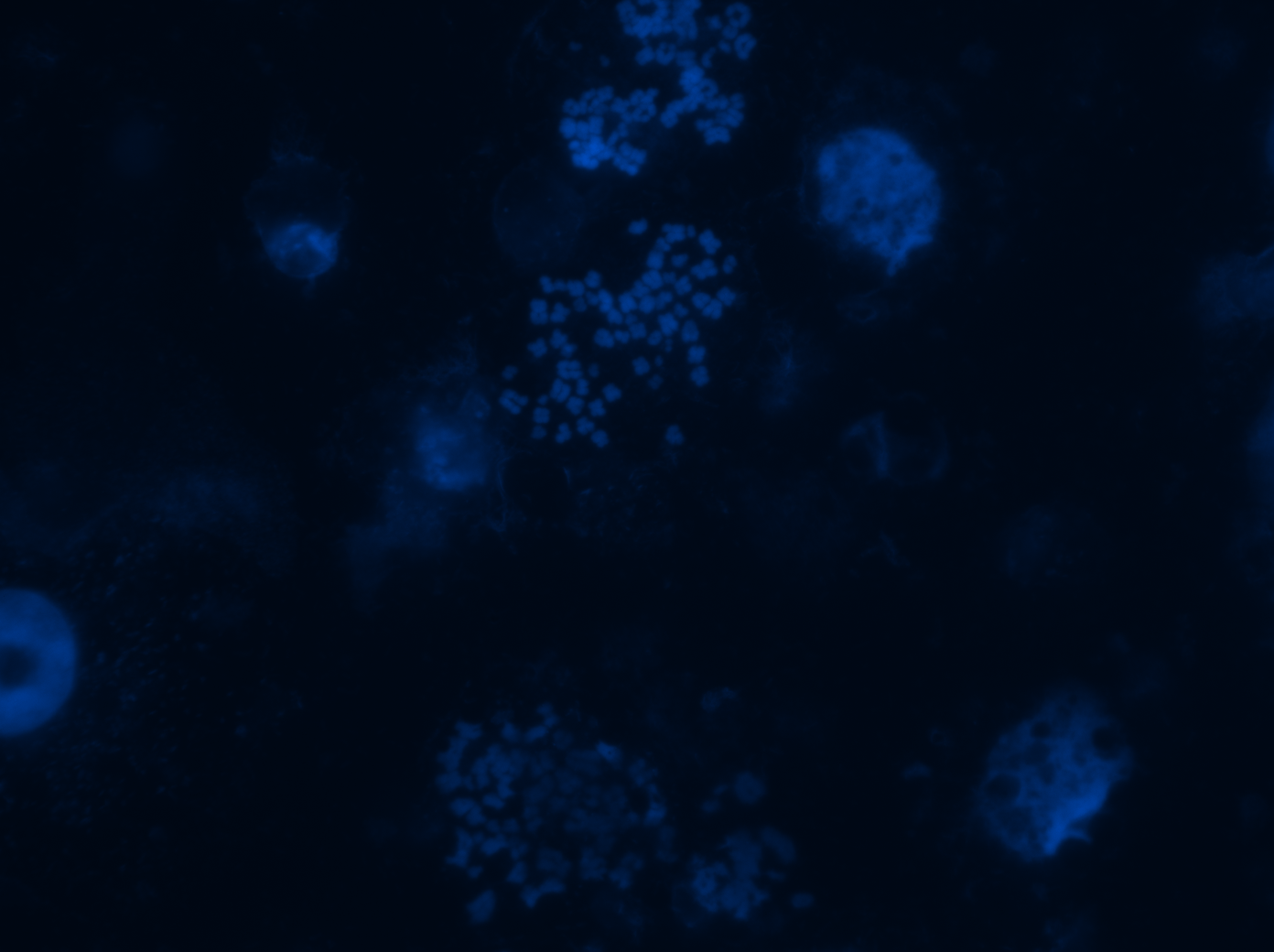

Supplement: Supplementary file 8 — Source data Fig. 4 [file 44318_2024_104_MOESM8_ESM.zip › Figure 4/4N/WT#A3+siCENP-U#1 DNA.tif]

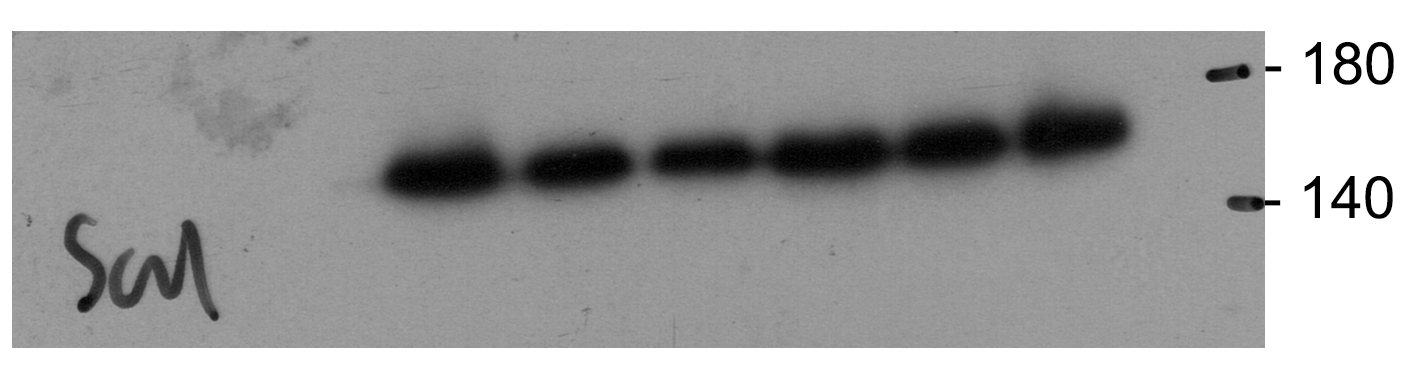

Supplement: Supplementary file 9 — Source data Fig. 5 [file 44318_2024_104_MOESM9_ESM.zip › Figure 5/5A/western Scc1.tif]

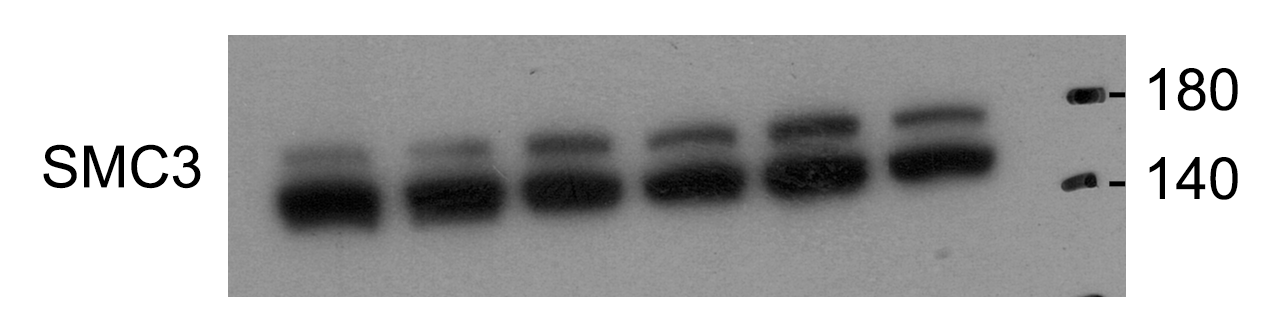

Supplement: Supplementary file 9 — Source data Fig. 5 [file 44318_2024_104_MOESM9_ESM.zip › Figure 5/5A/western SMC3.tif]

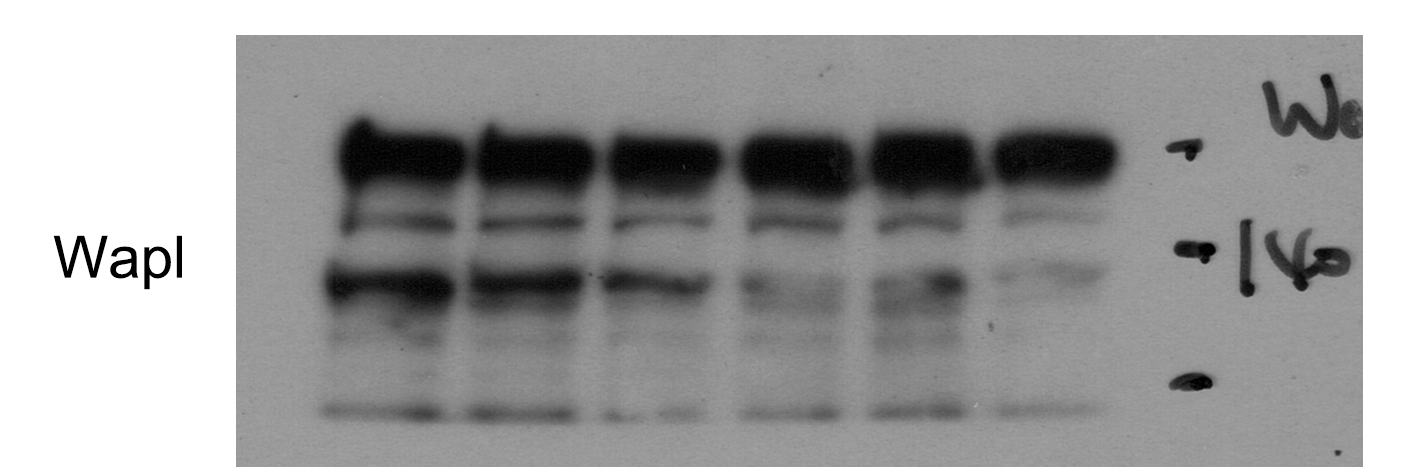

Supplement: Supplementary file 9 — Source data Fig. 5 [file 44318_2024_104_MOESM9_ESM.zip › Figure 5/5A/western Wapl.tif]

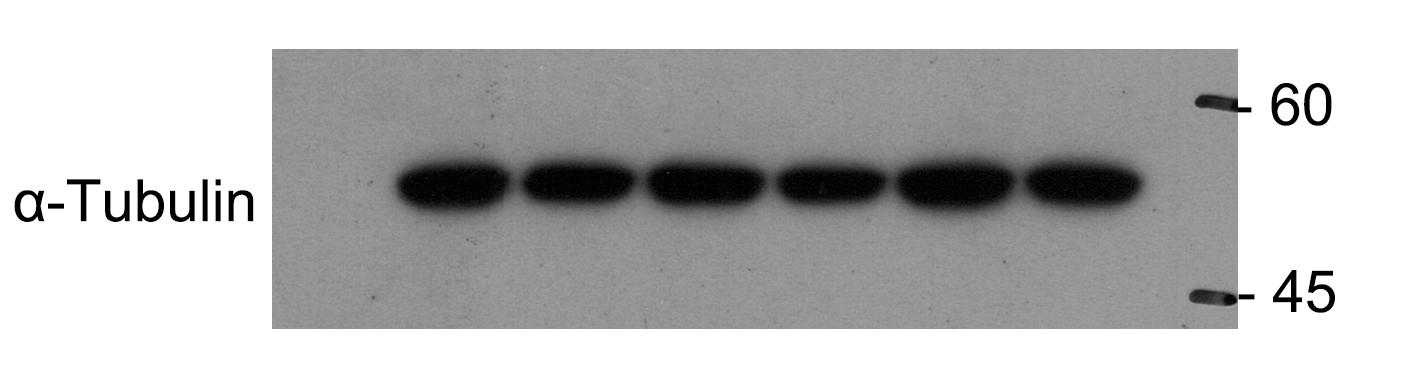

Supplement: Supplementary file 9 — Source data Fig. 5 [file 44318_2024_104_MOESM9_ESM.zip › Figure 5/5A/western a┴-Tubulin.tif]

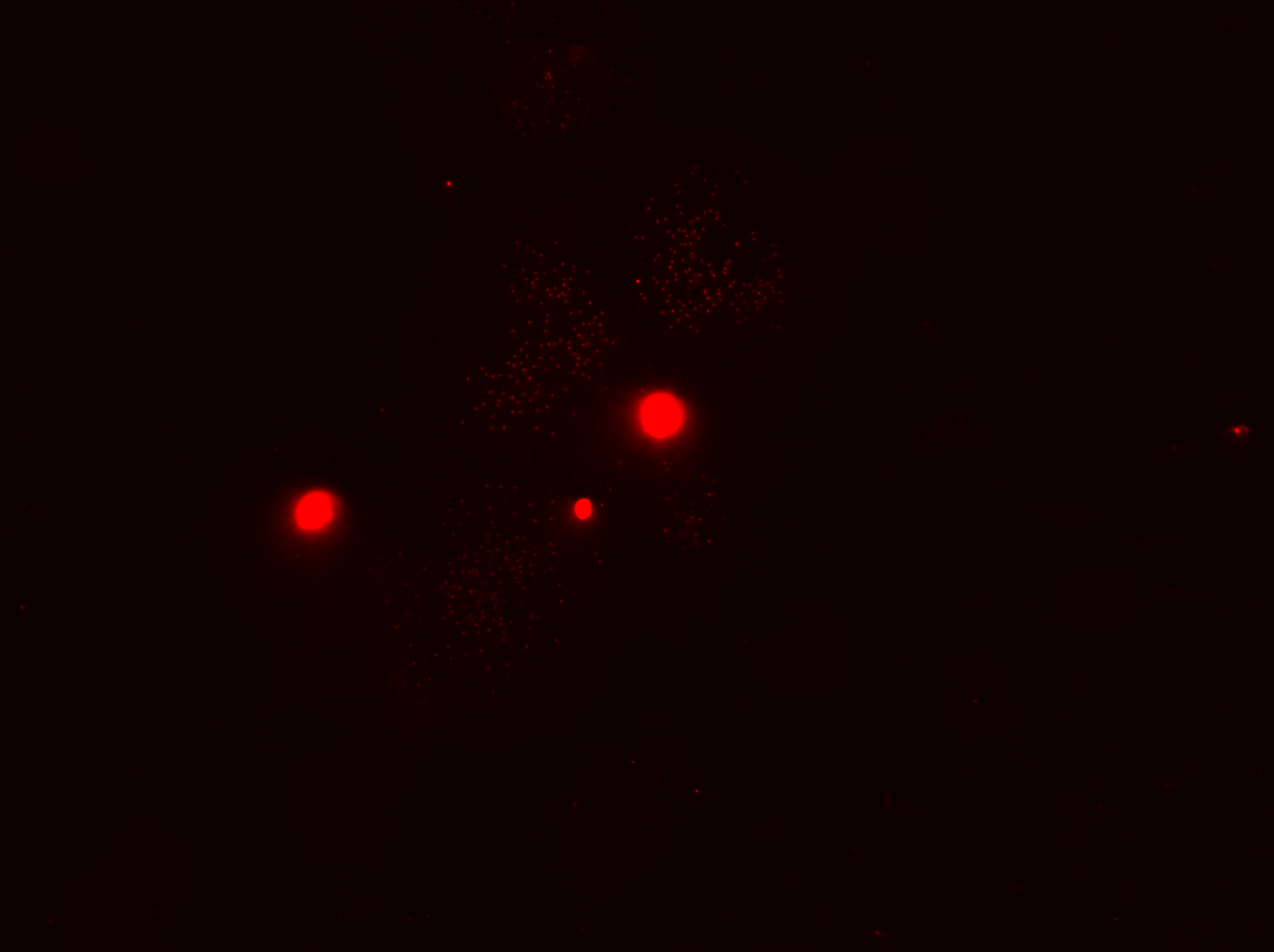

Supplement: Supplementary file 9 — Source data Fig. 5 [file 44318_2024_104_MOESM9_ESM.zip › Figure 5/5C/siCENP-U CENP-C.tif]

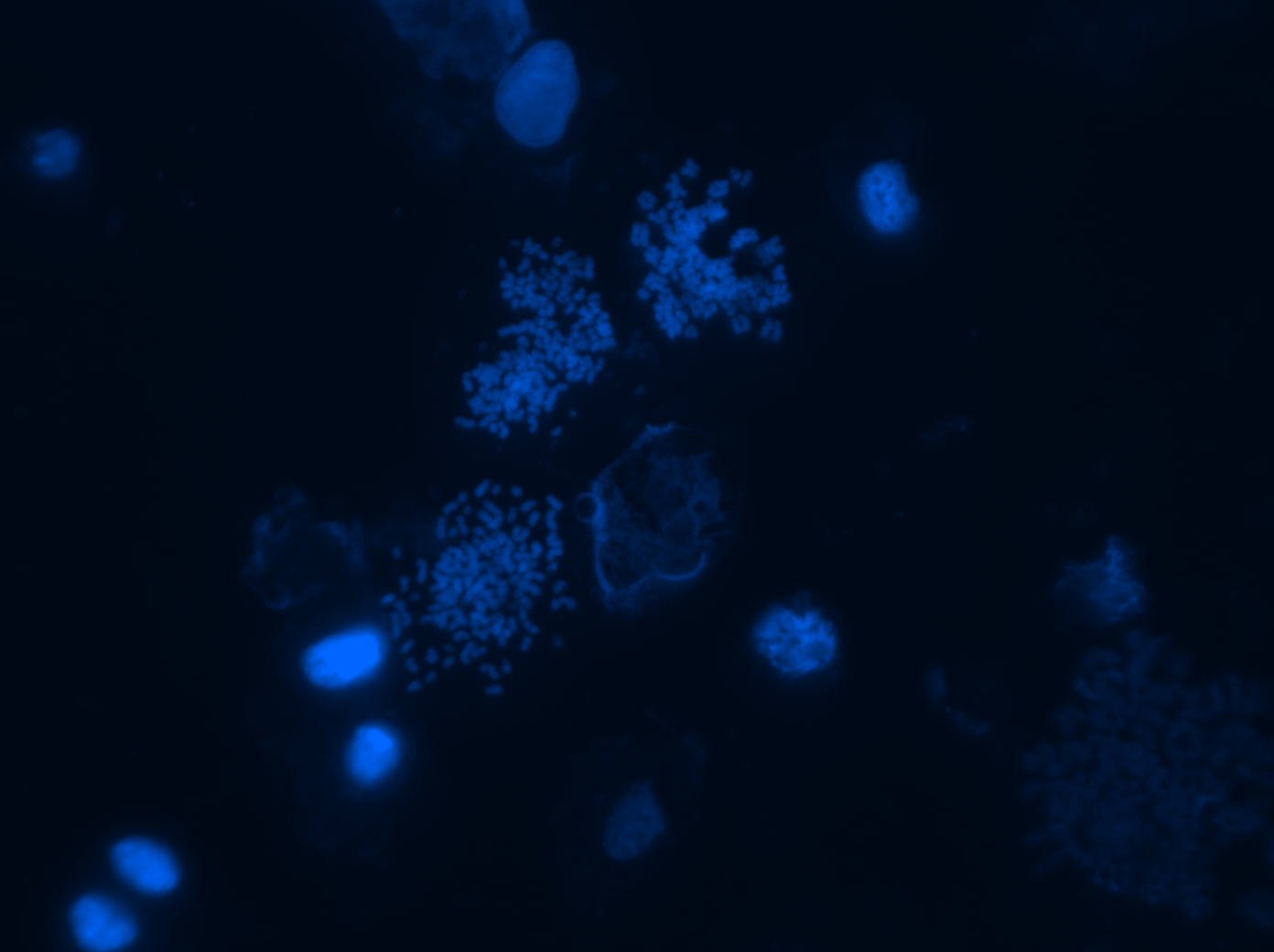

Supplement: Supplementary file 9 — Source data Fig. 5 [file 44318_2024_104_MOESM9_ESM.zip › Figure 5/5C/siCENP-U DNA.tif]

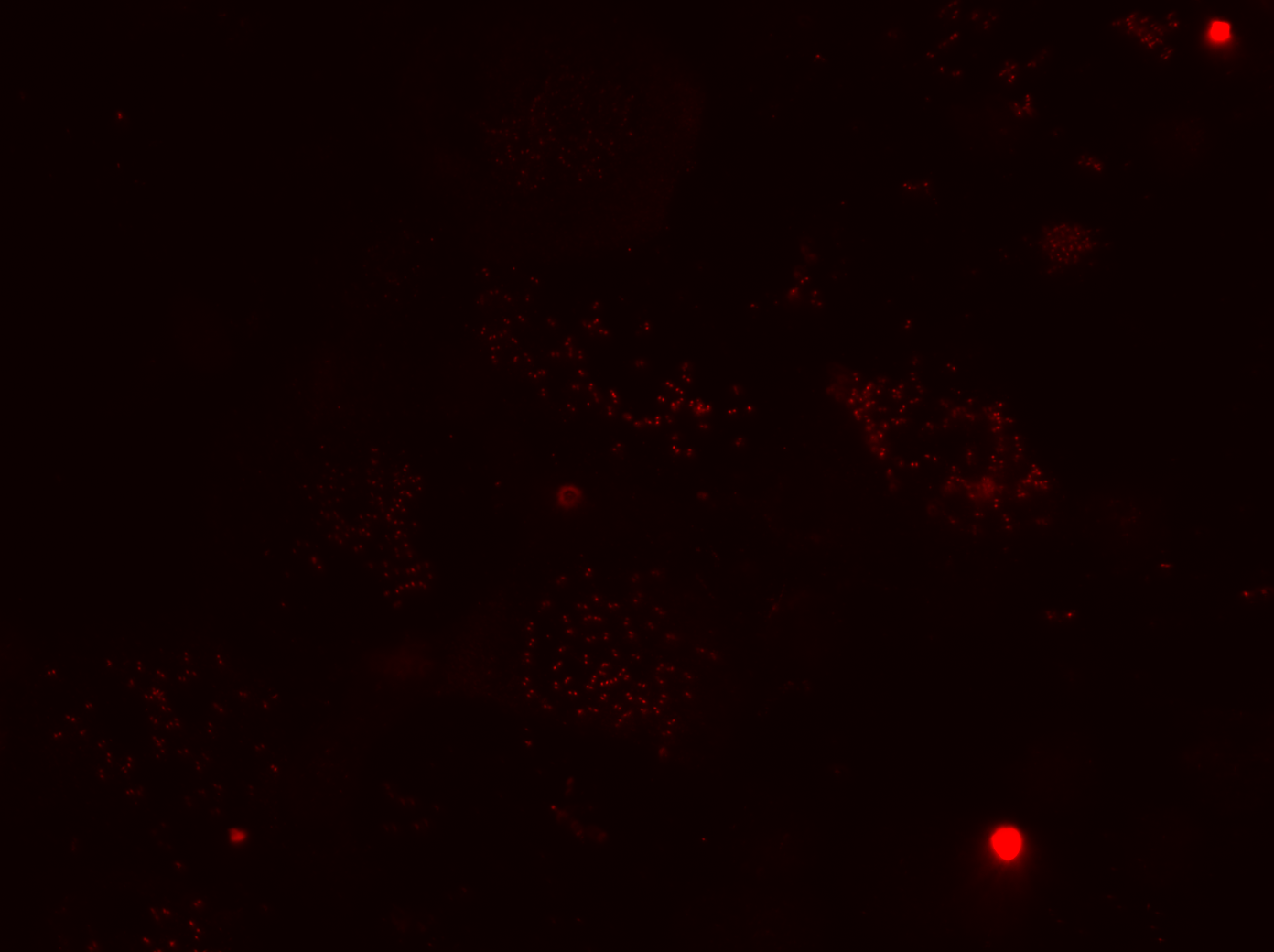

Supplement: Supplementary file 9 — Source data Fig. 5 [file 44318_2024_104_MOESM9_ESM.zip › Figure 5/5C/siCENP-U+siWapl CENP-C.tif]

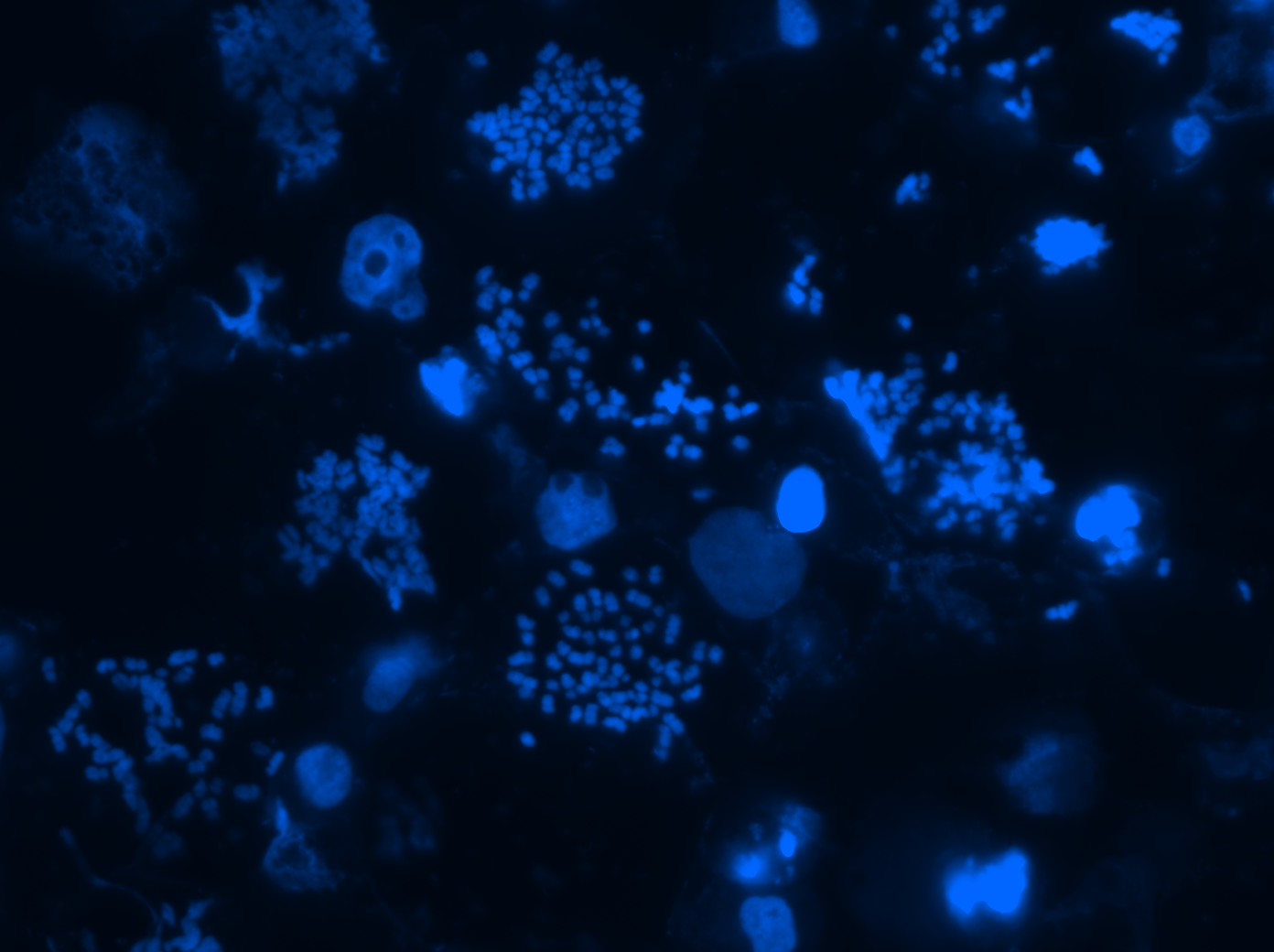

Supplement: Supplementary file 9 — Source data Fig. 5 [file 44318_2024_104_MOESM9_ESM.zip › Figure 5/5C/siCENP-U+siWapl DNA.tif]

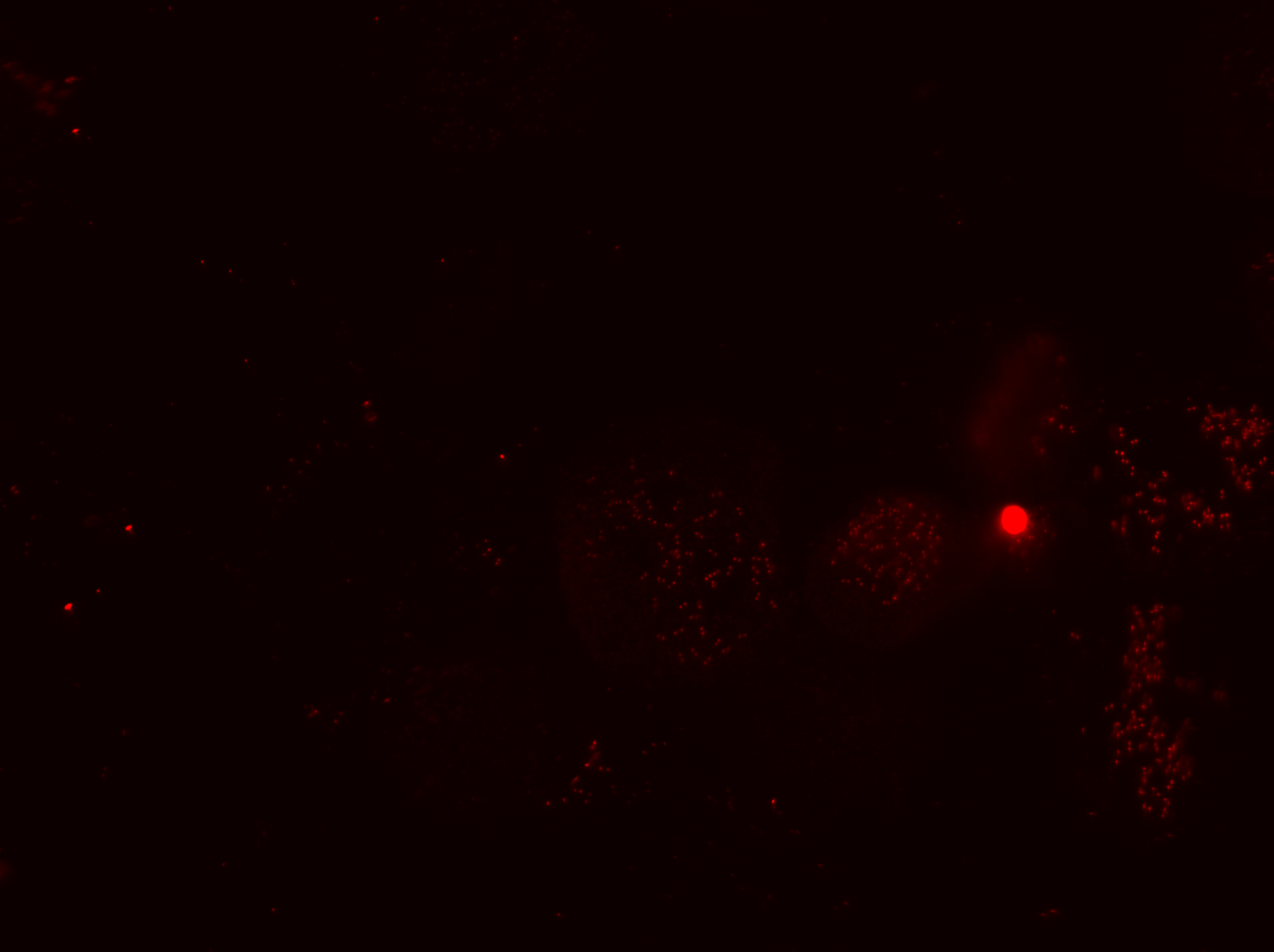

Supplement: Supplementary file 9 — Source data Fig. 5 [file 44318_2024_104_MOESM9_ESM.zip › Figure 5/5C/siControl CENP-C.tif]

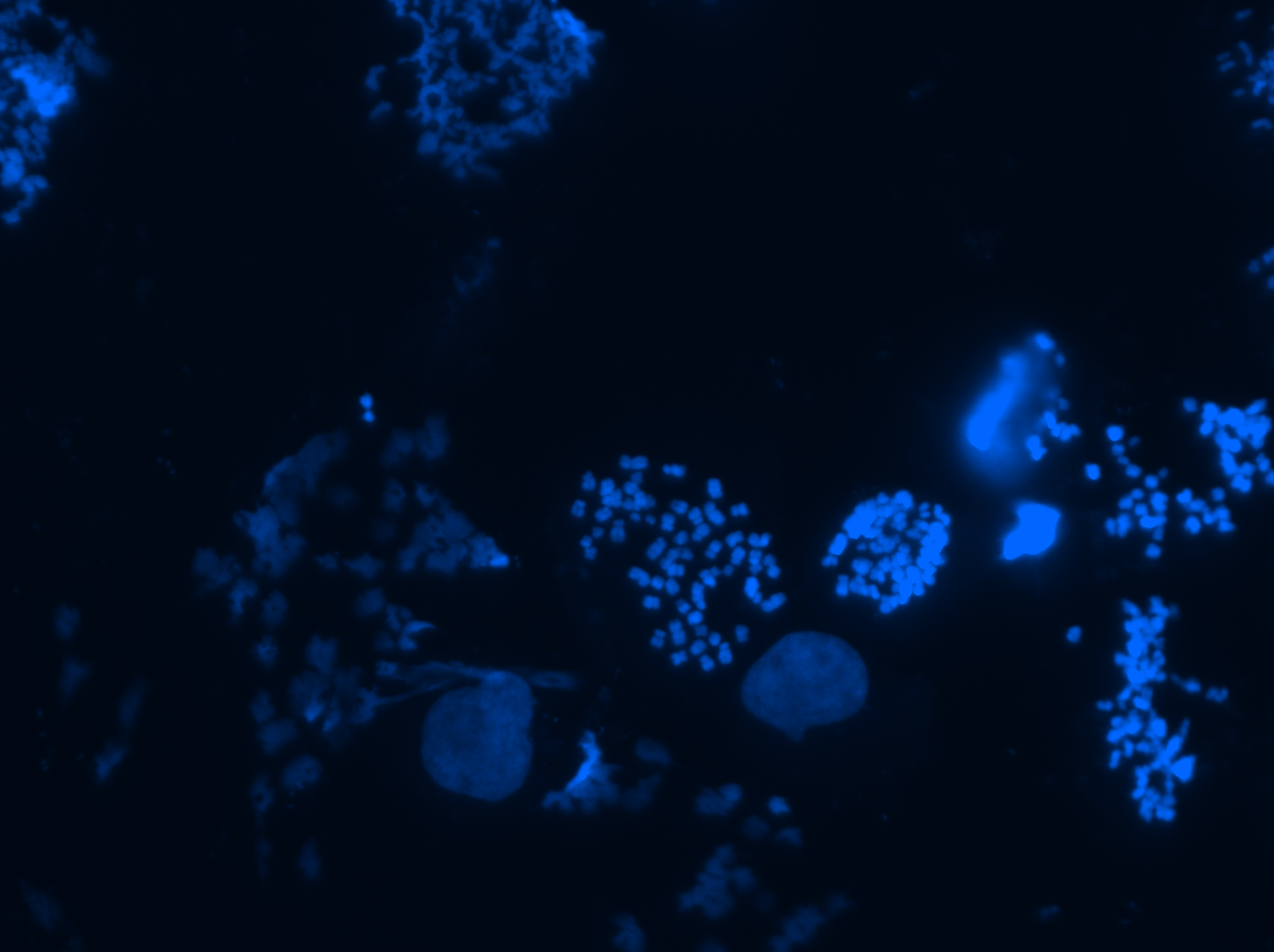

Supplement: Supplementary file 9 — Source data Fig. 5 [file 44318_2024_104_MOESM9_ESM.zip › Figure 5/5C/siControl DNA.tif]

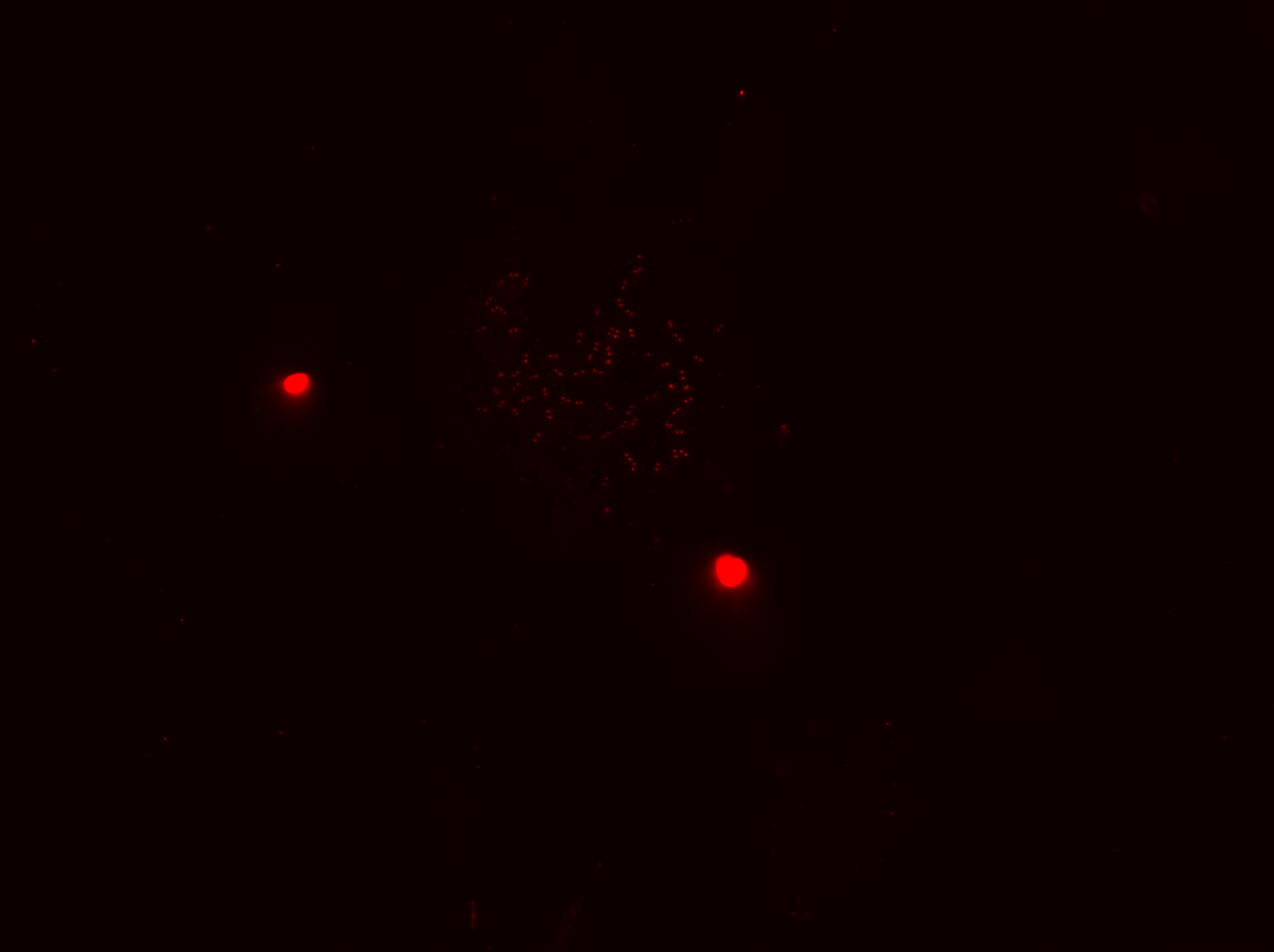

Supplement: Supplementary file 9 — Source data Fig. 5 [file 44318_2024_104_MOESM9_ESM.zip › Figure 5/5C/siWapl CENP-C.tif]

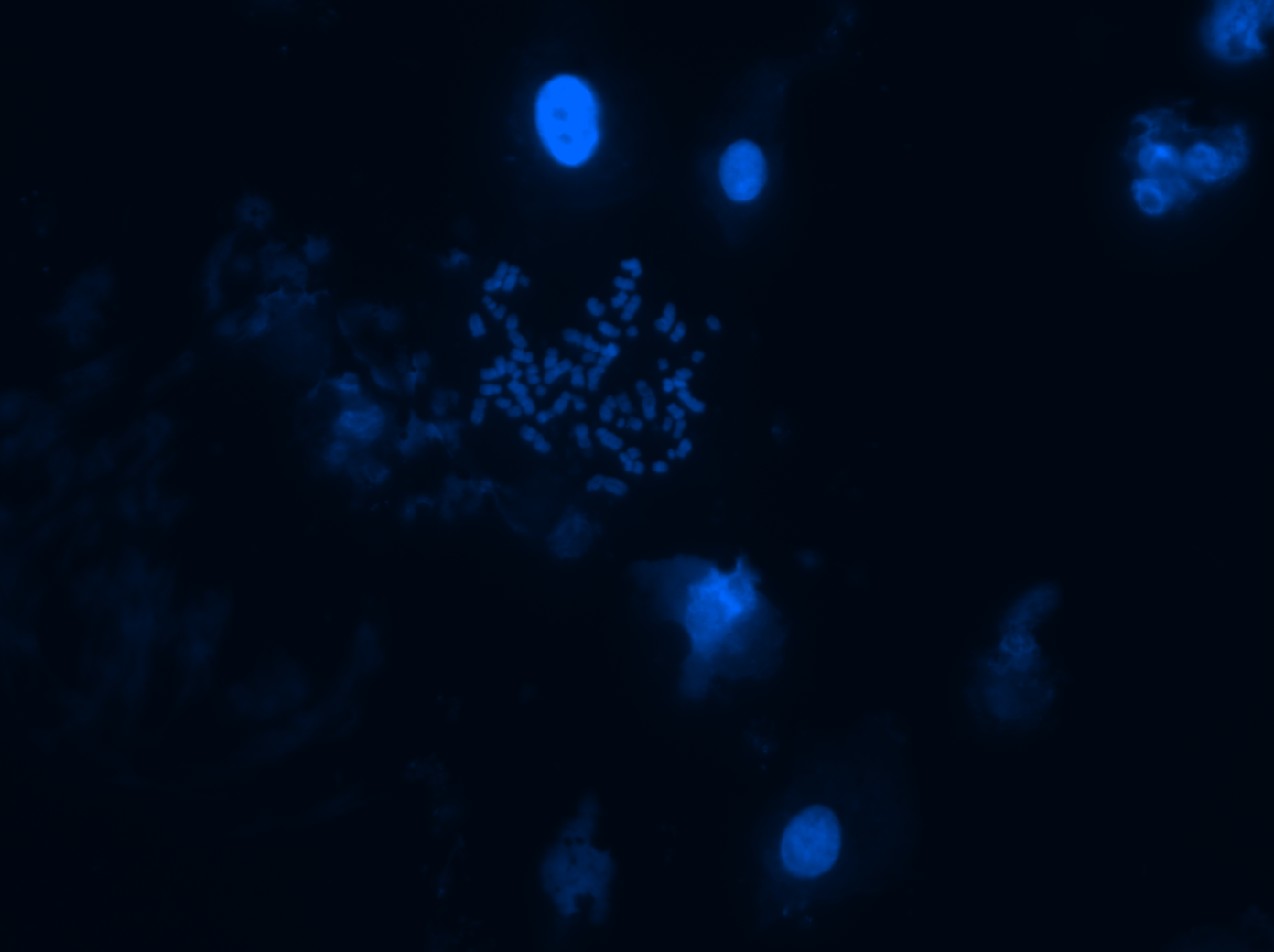

Supplement: Supplementary file 9 — Source data Fig. 5 [file 44318_2024_104_MOESM9_ESM.zip › Figure 5/5C/siWapl DNA.tif]

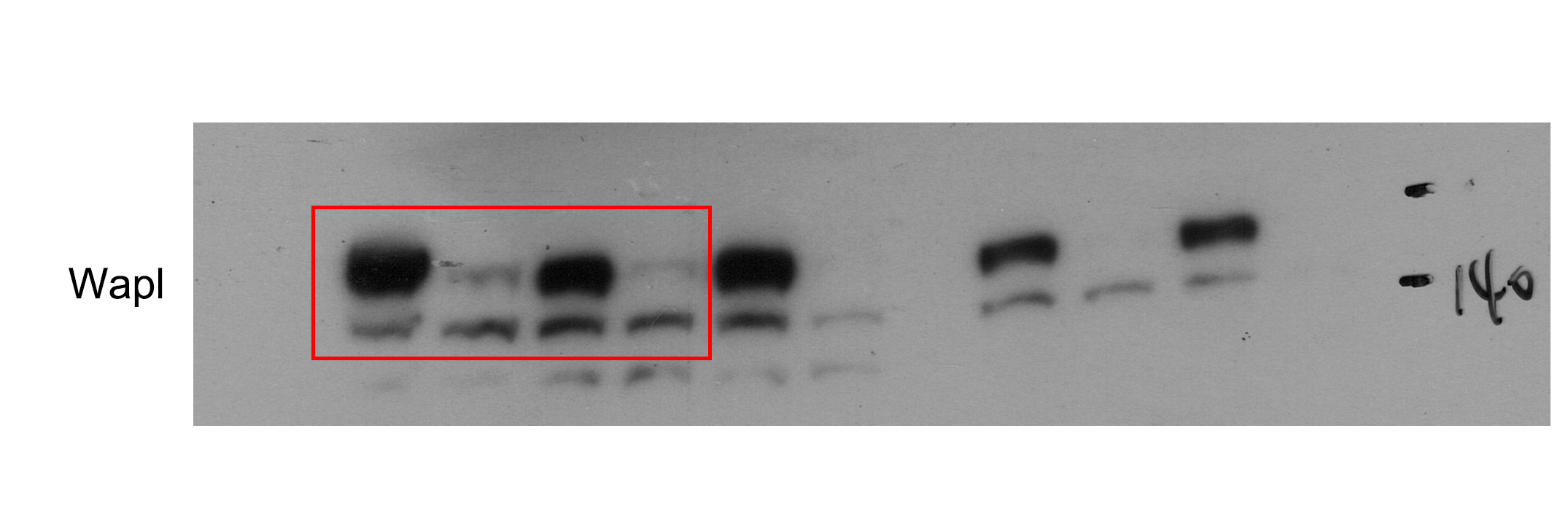

Supplement: Supplementary file 9 — Source data Fig. 5 [file 44318_2024_104_MOESM9_ESM.zip › Figure 5/5D/western Wapl.tif]

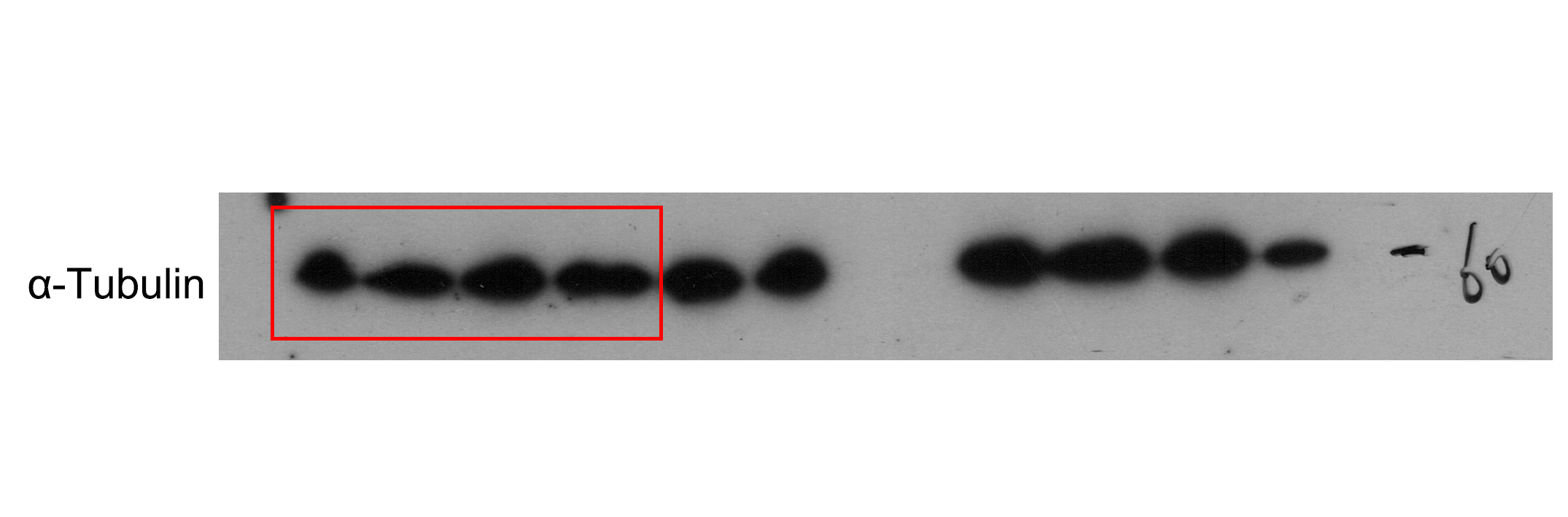

Supplement: Supplementary file 9 — Source data Fig. 5 [file 44318_2024_104_MOESM9_ESM.zip › Figure 5/5D/western a┴-Tubulin.tif]

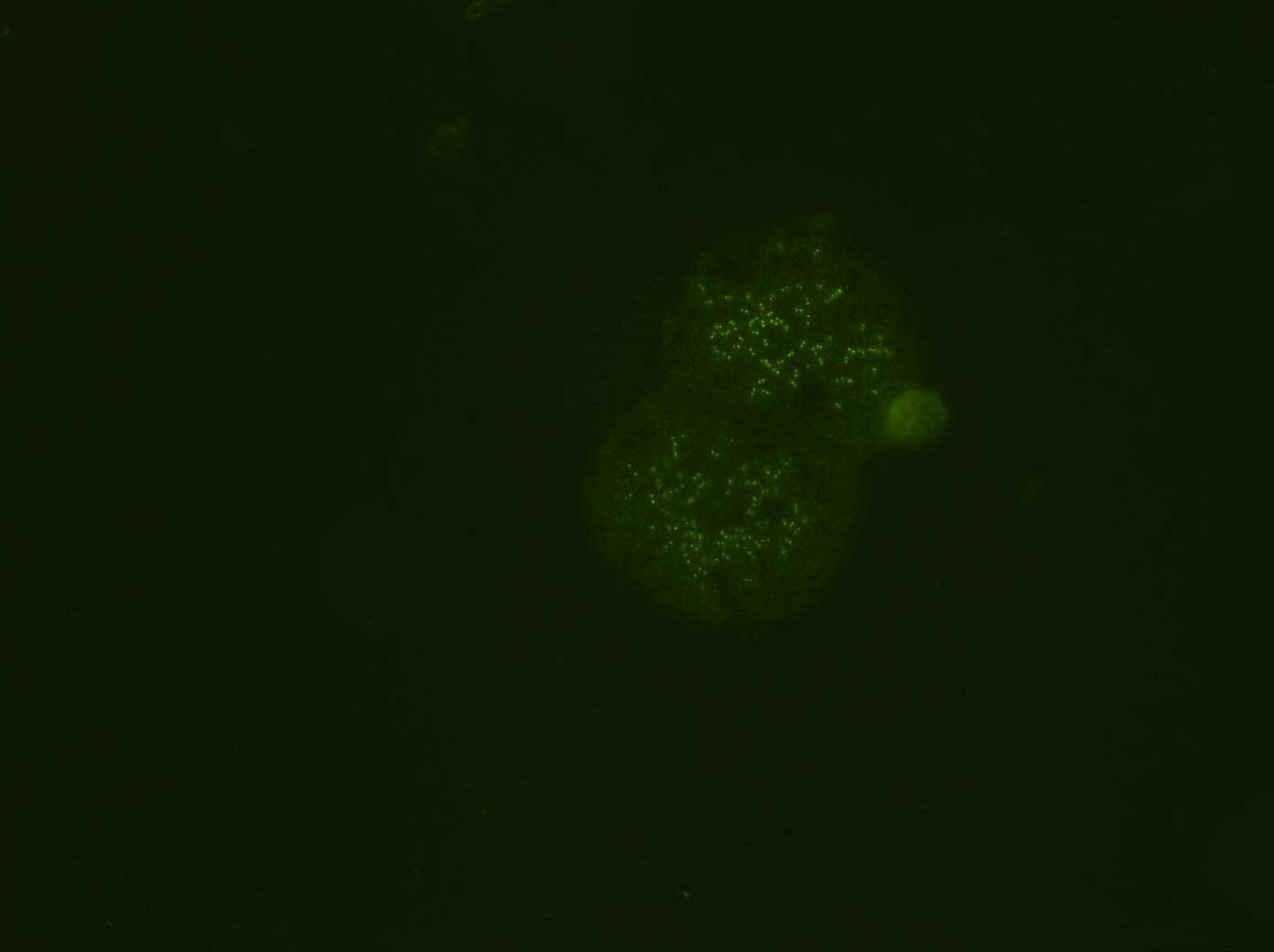

Supplement: Supplementary file 9 — Source data Fig. 5 [file 44318_2024_104_MOESM9_ESM.zip › Figure 5/5G/HeLa+siCENP-U#1 CENP-C.tif]

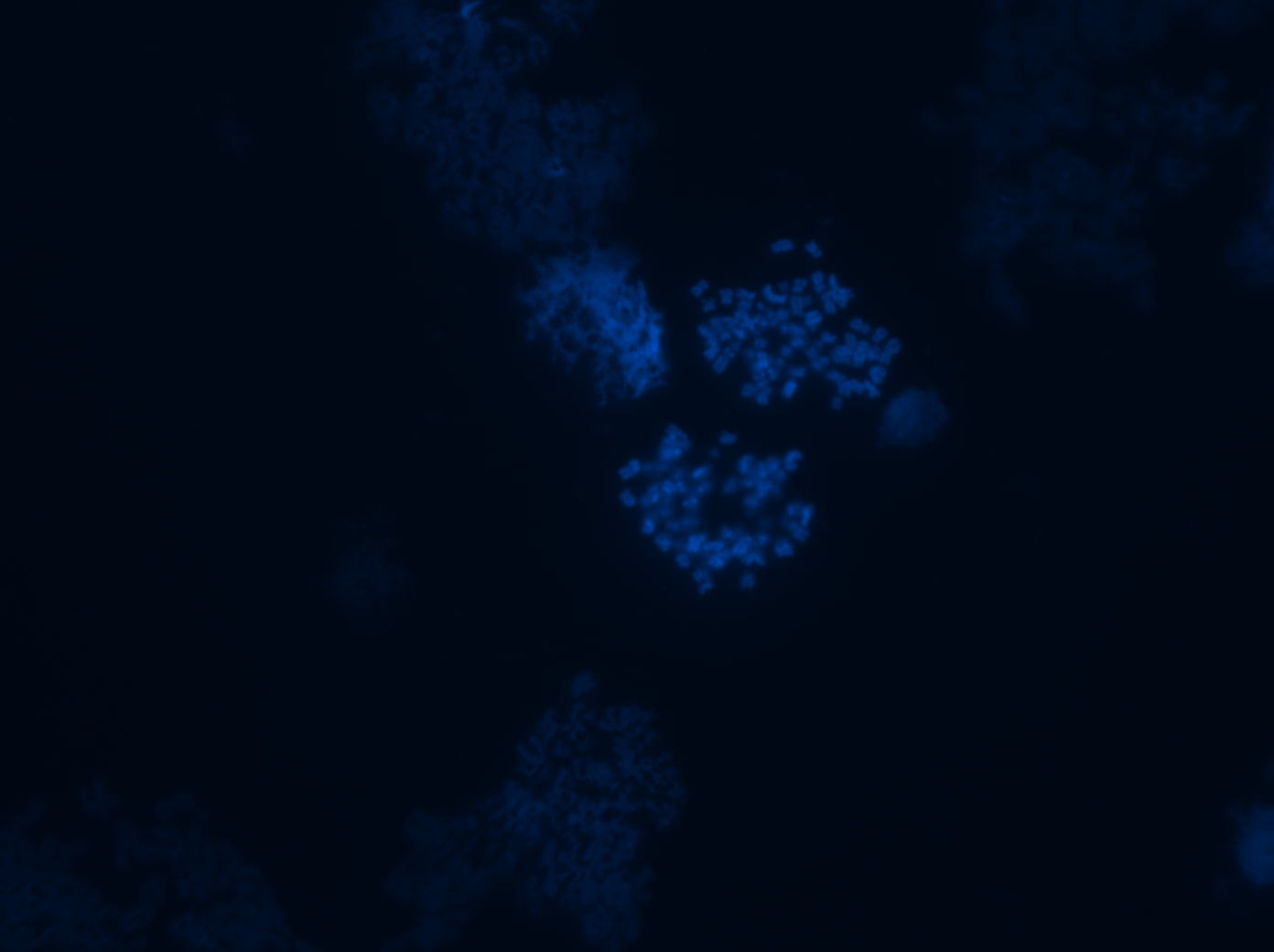

Supplement: Supplementary file 9 — Source data Fig. 5 [file 44318_2024_104_MOESM9_ESM.zip › Figure 5/5G/HeLa+siCENP-U#1 DNA.tif]

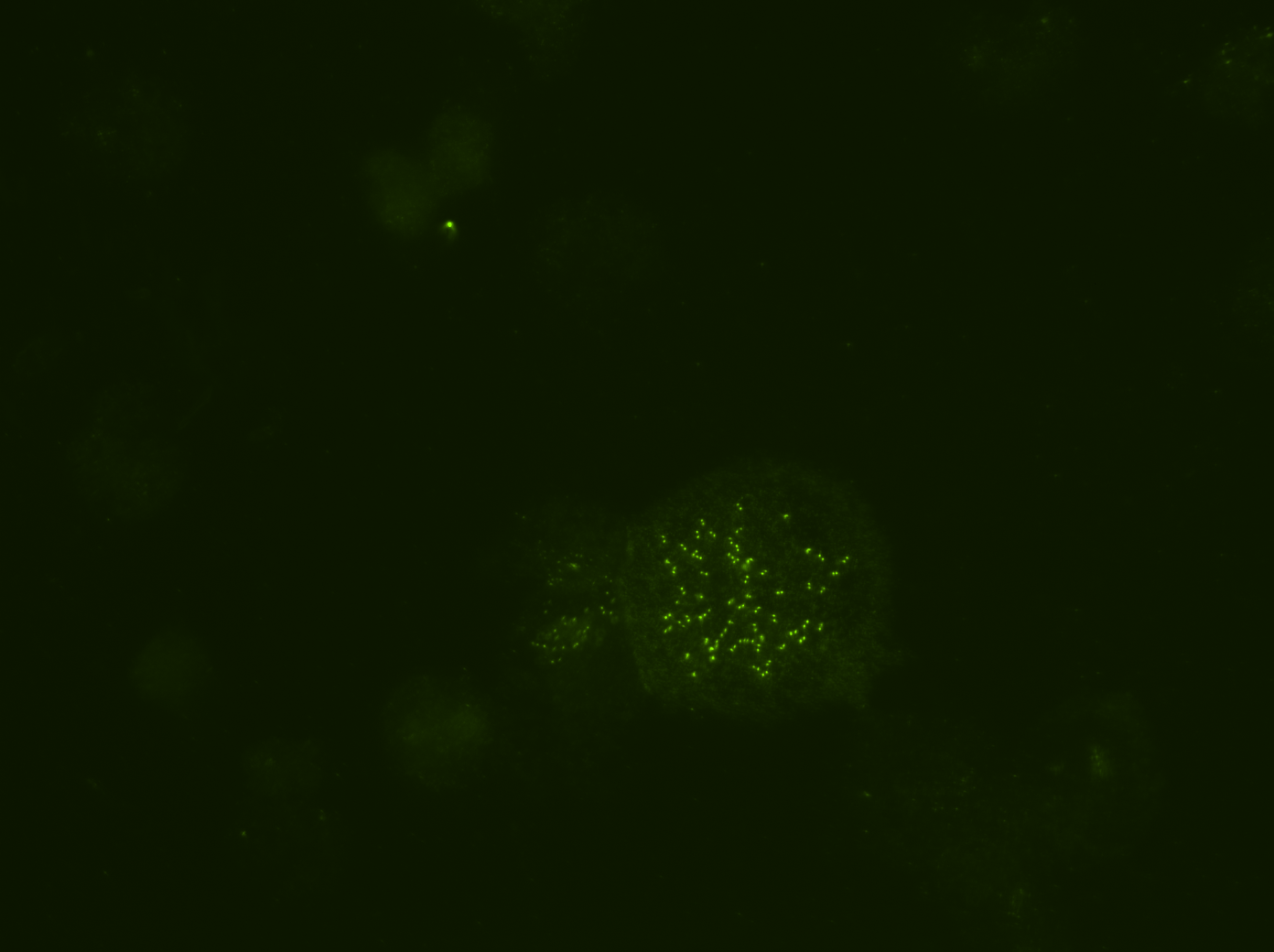

Supplement: Supplementary file 9 — Source data Fig. 5 [file 44318_2024_104_MOESM9_ESM.zip › Figure 5/5G/HeLa+siCENP-U#1+siWapl CENP-C.tif]

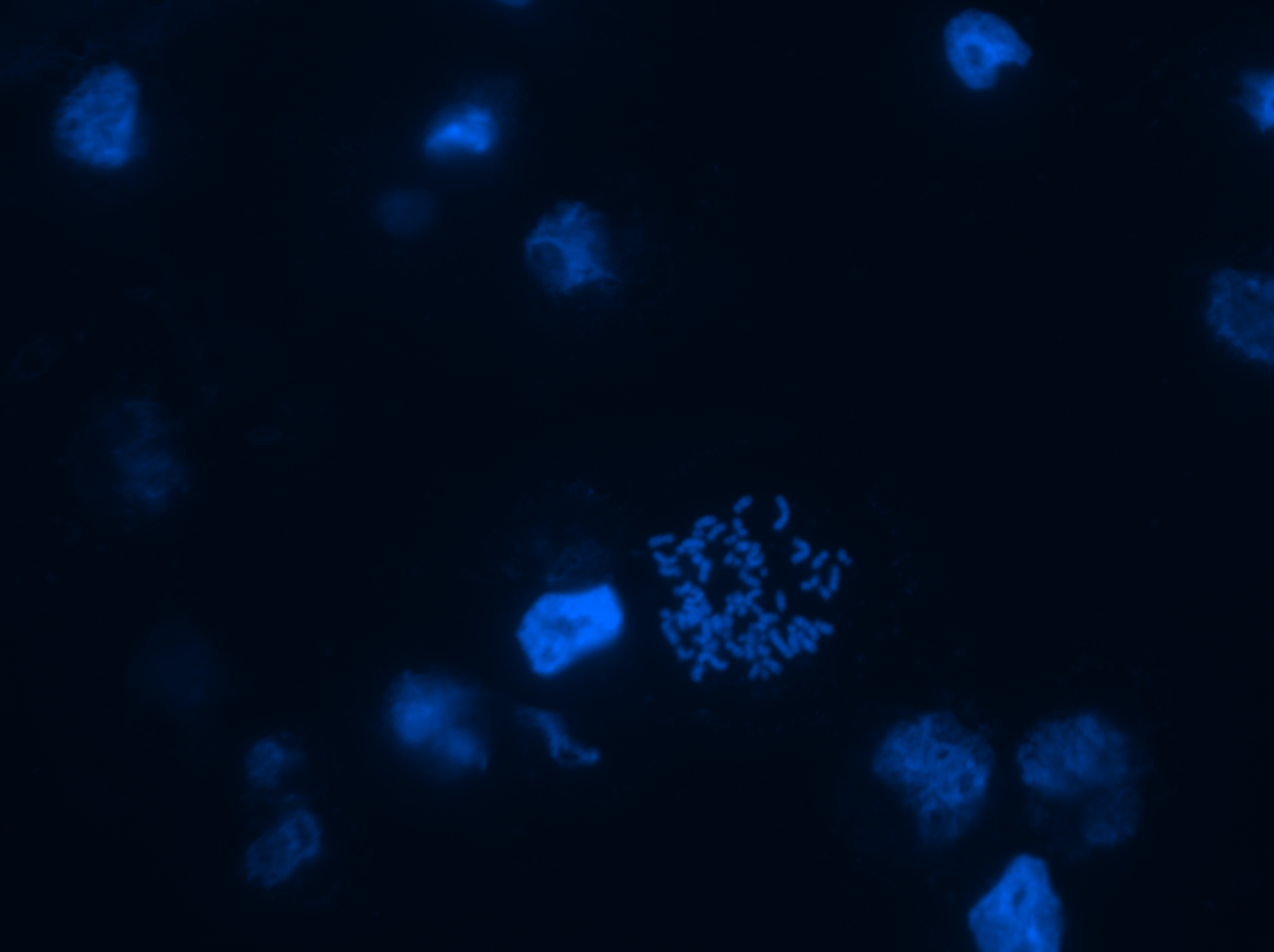

Supplement: Supplementary file 9 — Source data Fig. 5 [file 44318_2024_104_MOESM9_ESM.zip › Figure 5/5G/HeLa+siCENP-U#1+siWapl DNA.tif]

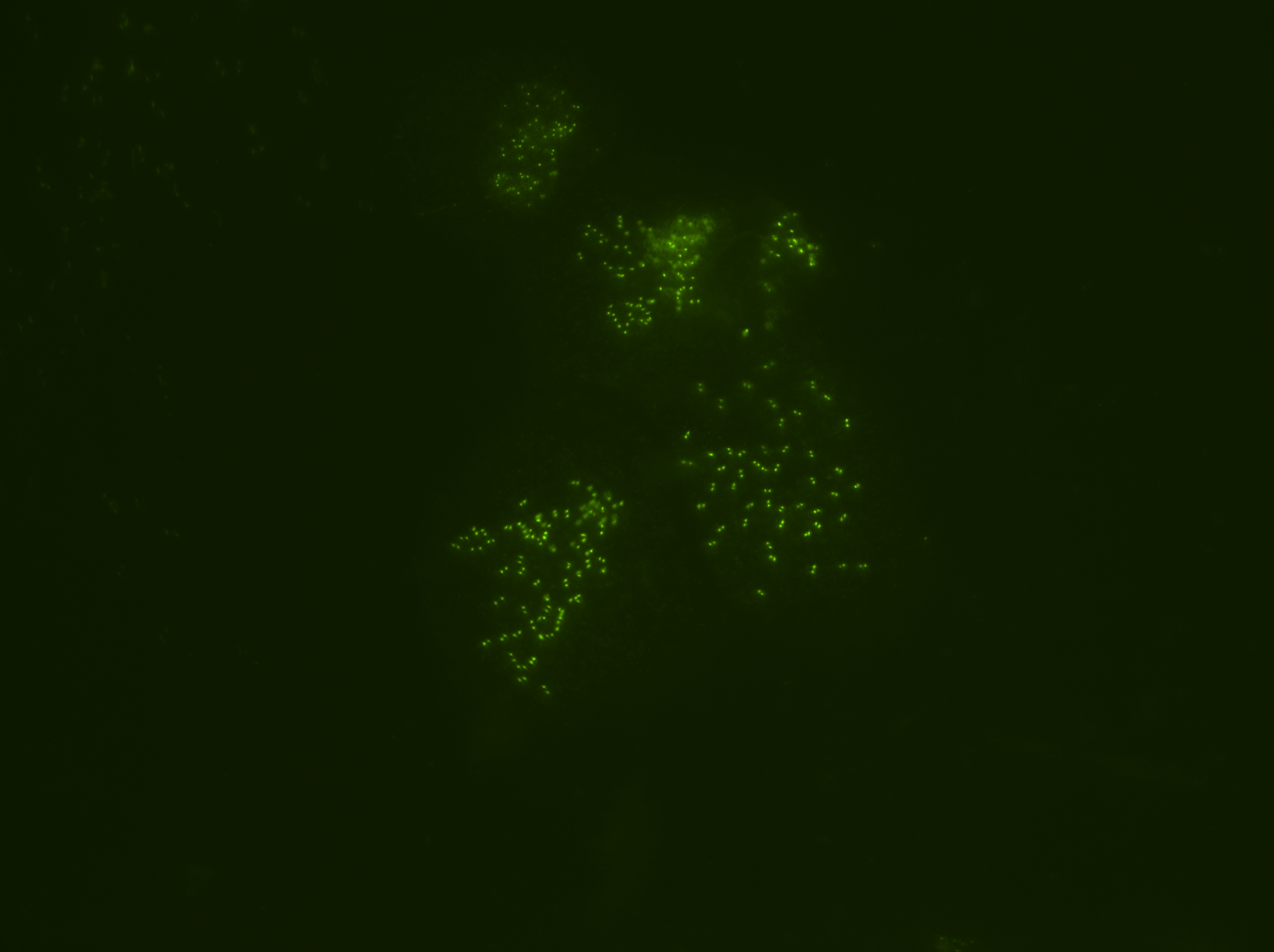

Supplement: Supplementary file 9 — Source data Fig. 5 [file 44318_2024_104_MOESM9_ESM.zip › Figure 5/5G/HeLa+siControl CENP-C.tif]

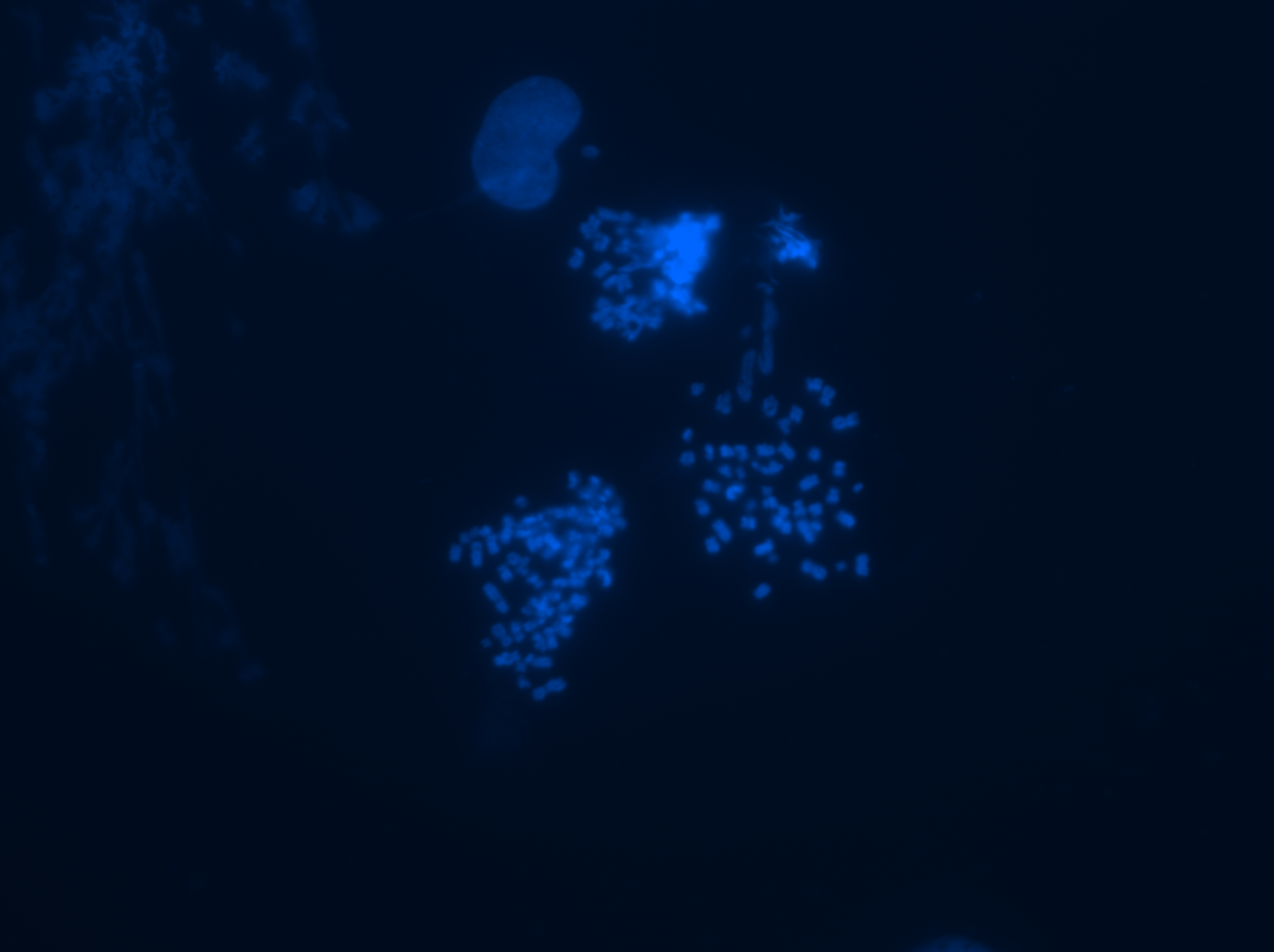

Supplement: Supplementary file 9 — Source data Fig. 5 [file 44318_2024_104_MOESM9_ESM.zip › Figure 5/5G/HeLa+siControl DNA.tif]

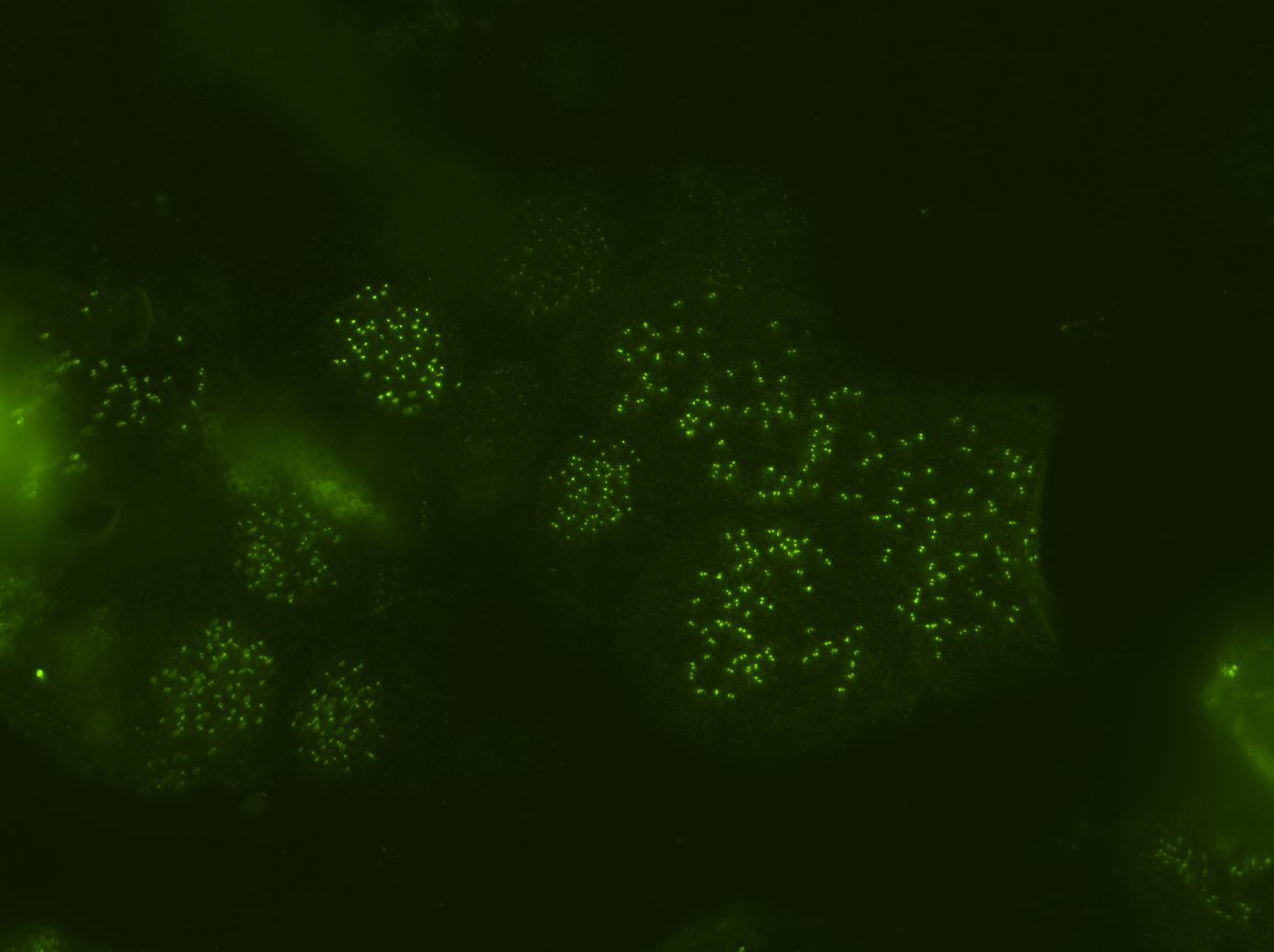

Supplement: Supplementary file 9 — Source data Fig. 5 [file 44318_2024_104_MOESM9_ESM.zip › Figure 5/5G/HeLa+siWapl CENP-C.tif]

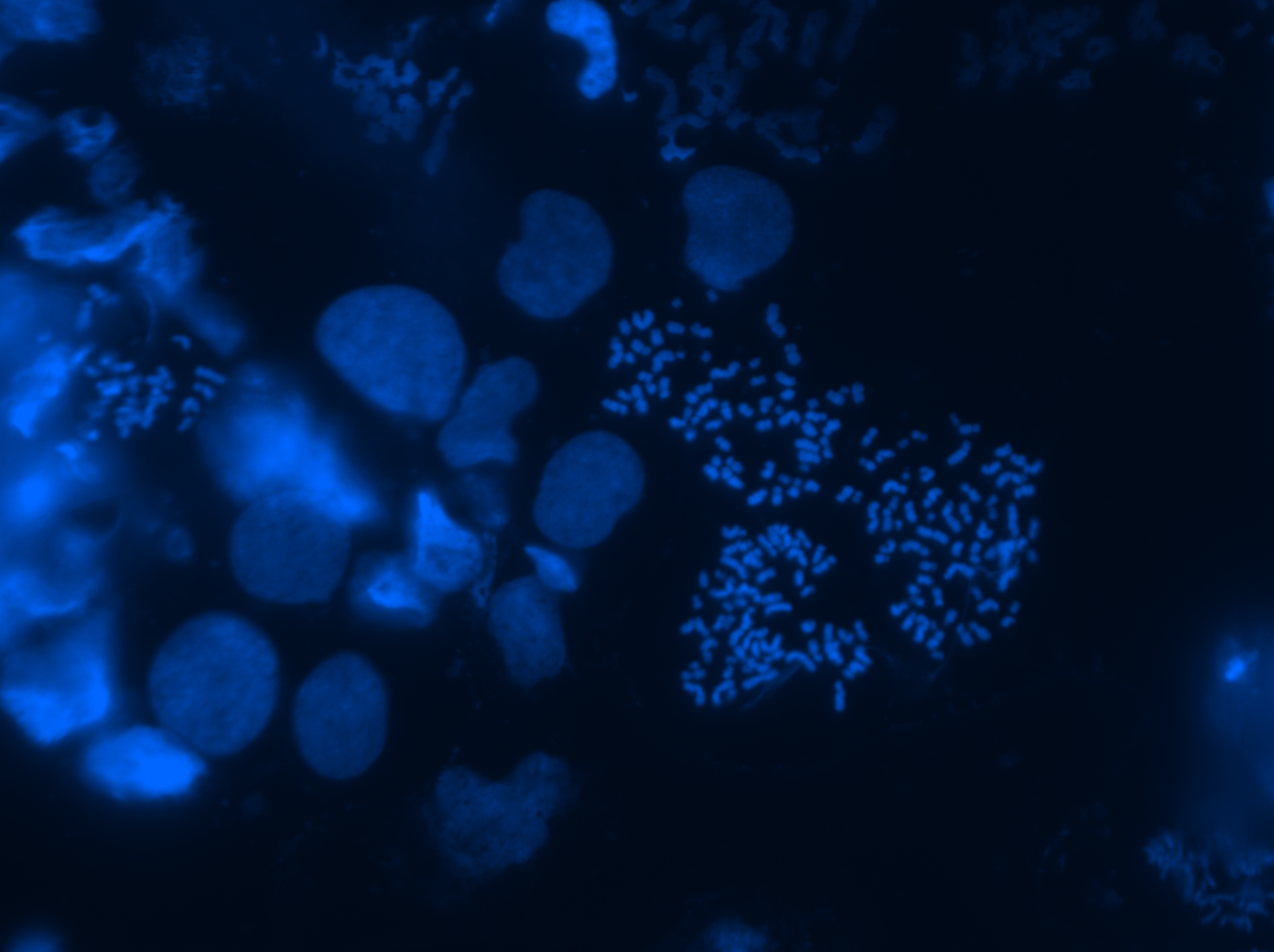

Supplement: Supplementary file 9 — Source data Fig. 5 [file 44318_2024_104_MOESM9_ESM.zip › Figure 5/5G/HeLa+siWapl DNA.tif]

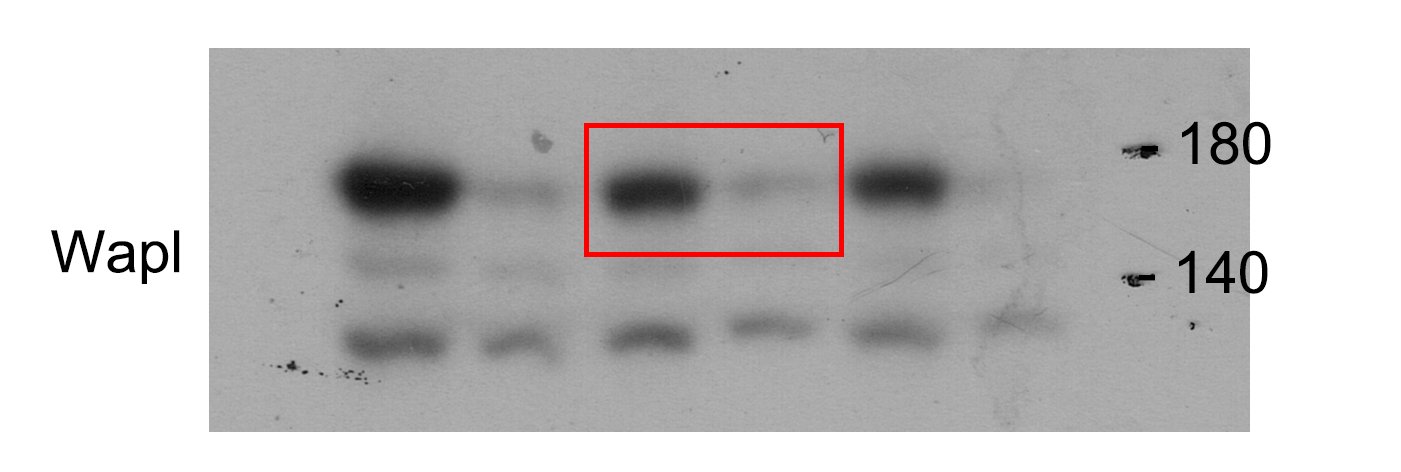

Supplement: Supplementary file 9 — Source data Fig. 5 [file 44318_2024_104_MOESM9_ESM.zip › Figure 5/5H/western Wapl.tif]

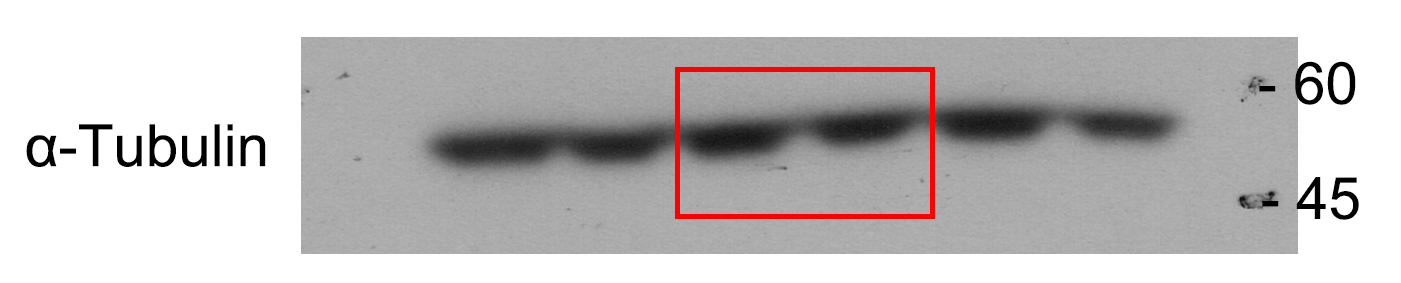

Supplement: Supplementary file 9 — Source data Fig. 5 [file 44318_2024_104_MOESM9_ESM.zip › Figure 5/5H/western a┴-Tubulin.tif]

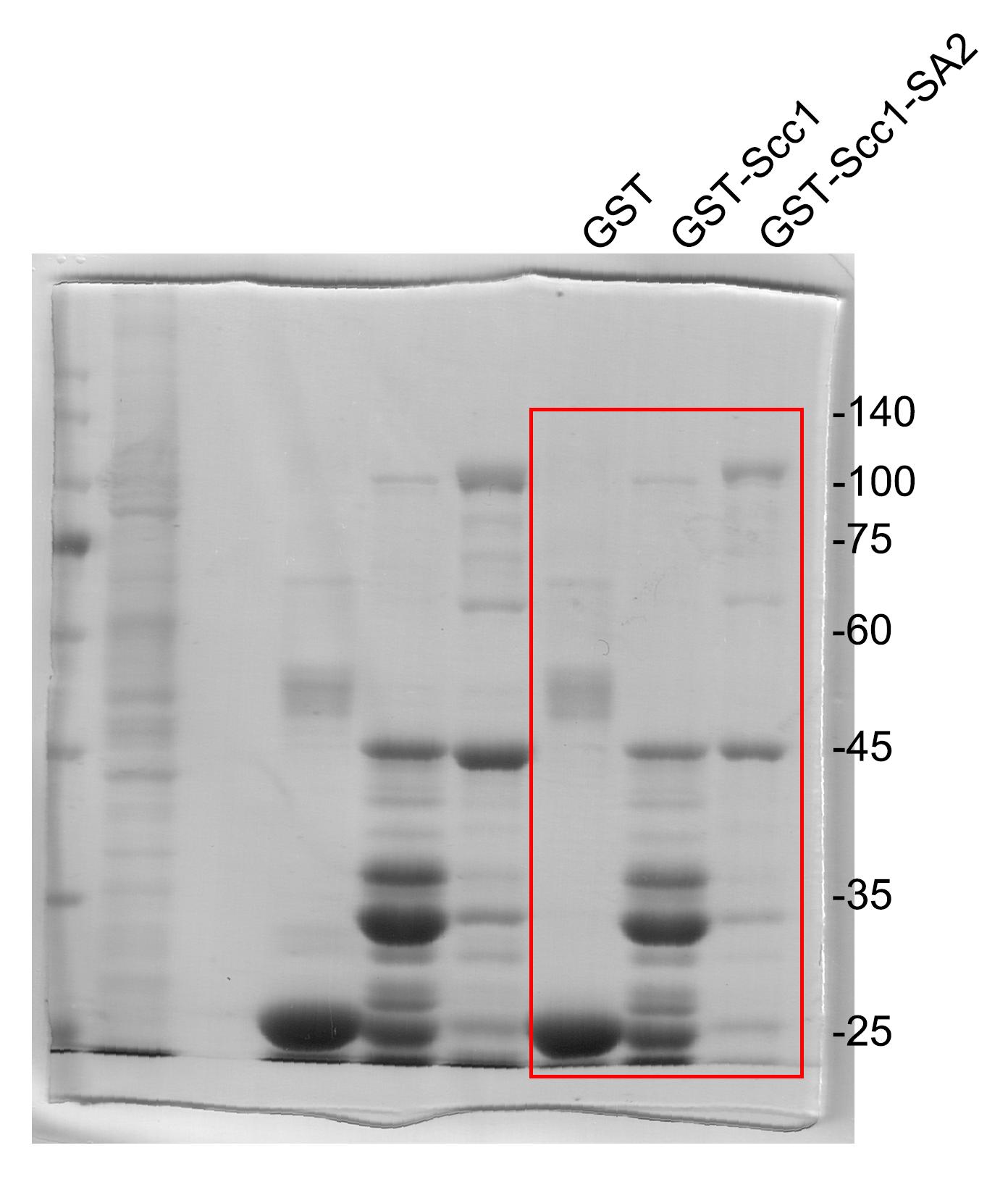

Supplement: Supplementary file 10 — Source data Fig. 6 [file 44318_2024_104_MOESM10_ESM.zip › Figure 6/6A/6A CBB.tif]

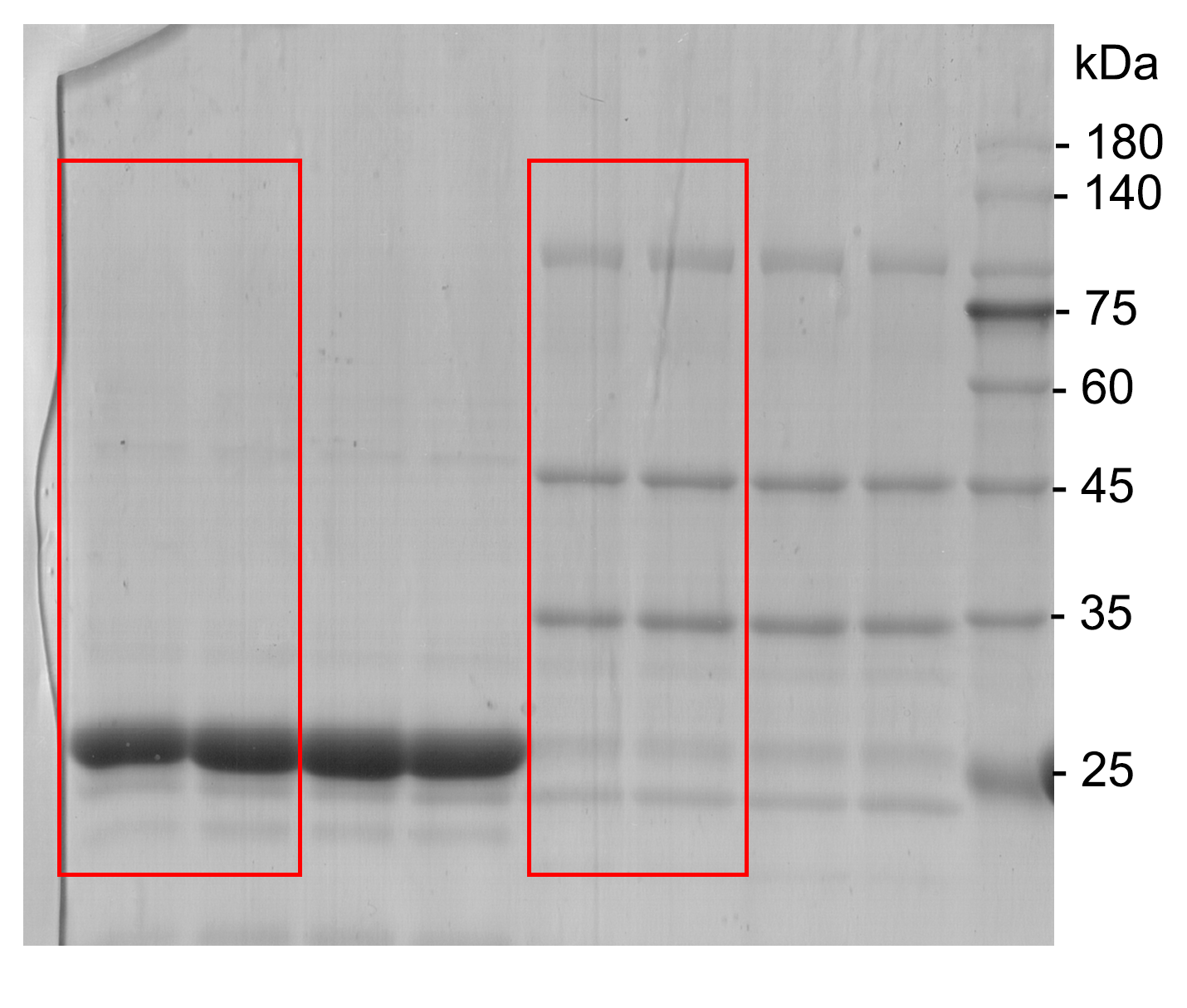

Supplement: Supplementary file 10 — Source data Fig. 6 [file 44318_2024_104_MOESM10_ESM.zip › Figure 6/6B/CBB.tif]

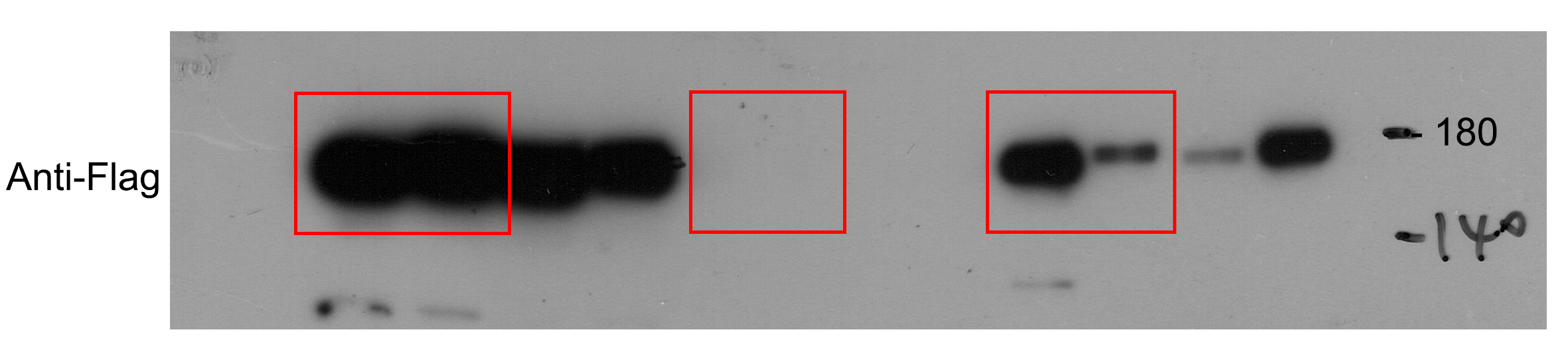

Supplement: Supplementary file 10 — Source data Fig. 6 [file 44318_2024_104_MOESM10_ESM.zip › Figure 6/6B/western Flag.tif]

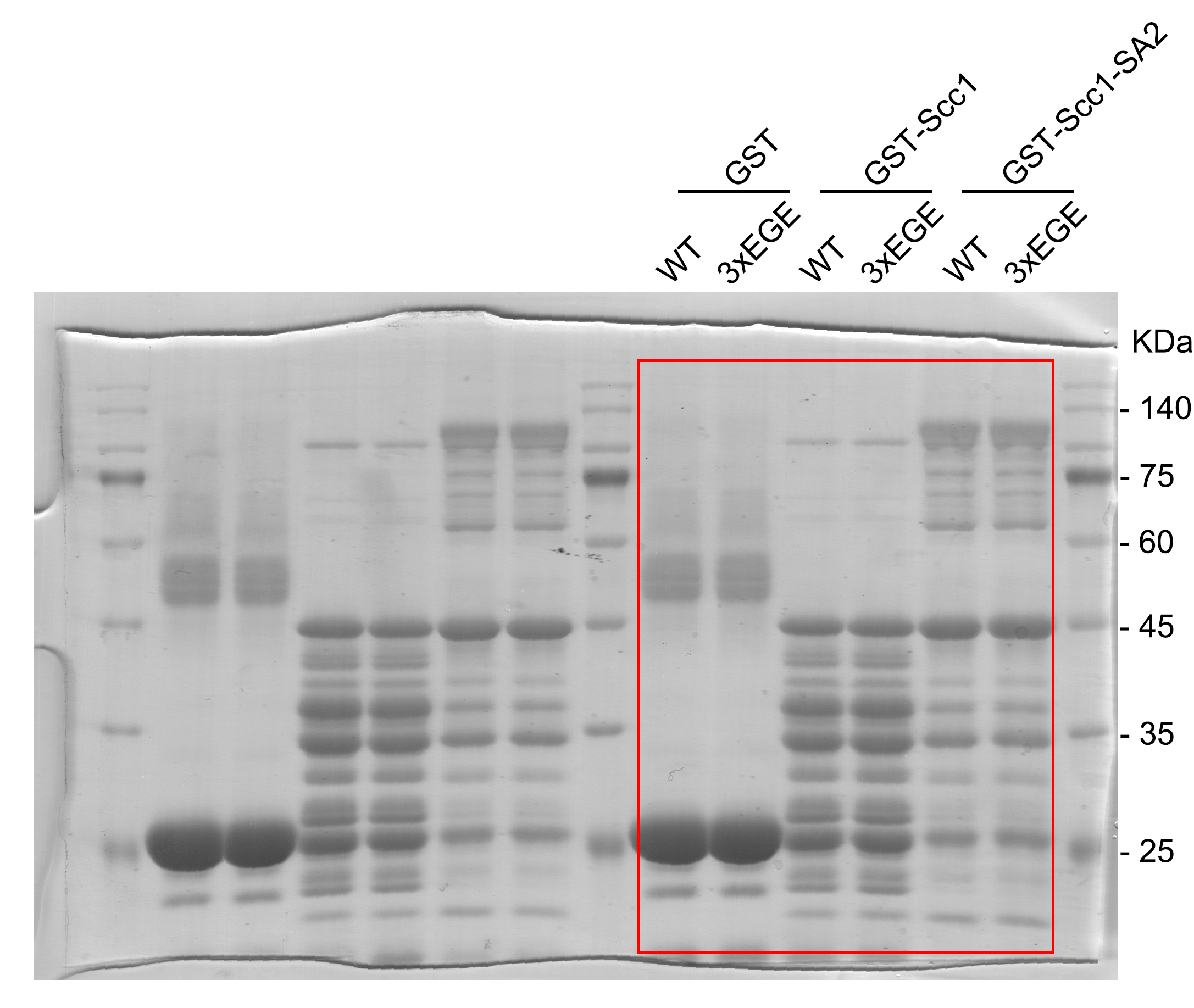

Supplement: Supplementary file 10 — Source data Fig. 6 [file 44318_2024_104_MOESM10_ESM.zip › Figure 6/6C/6C CBB.tif]

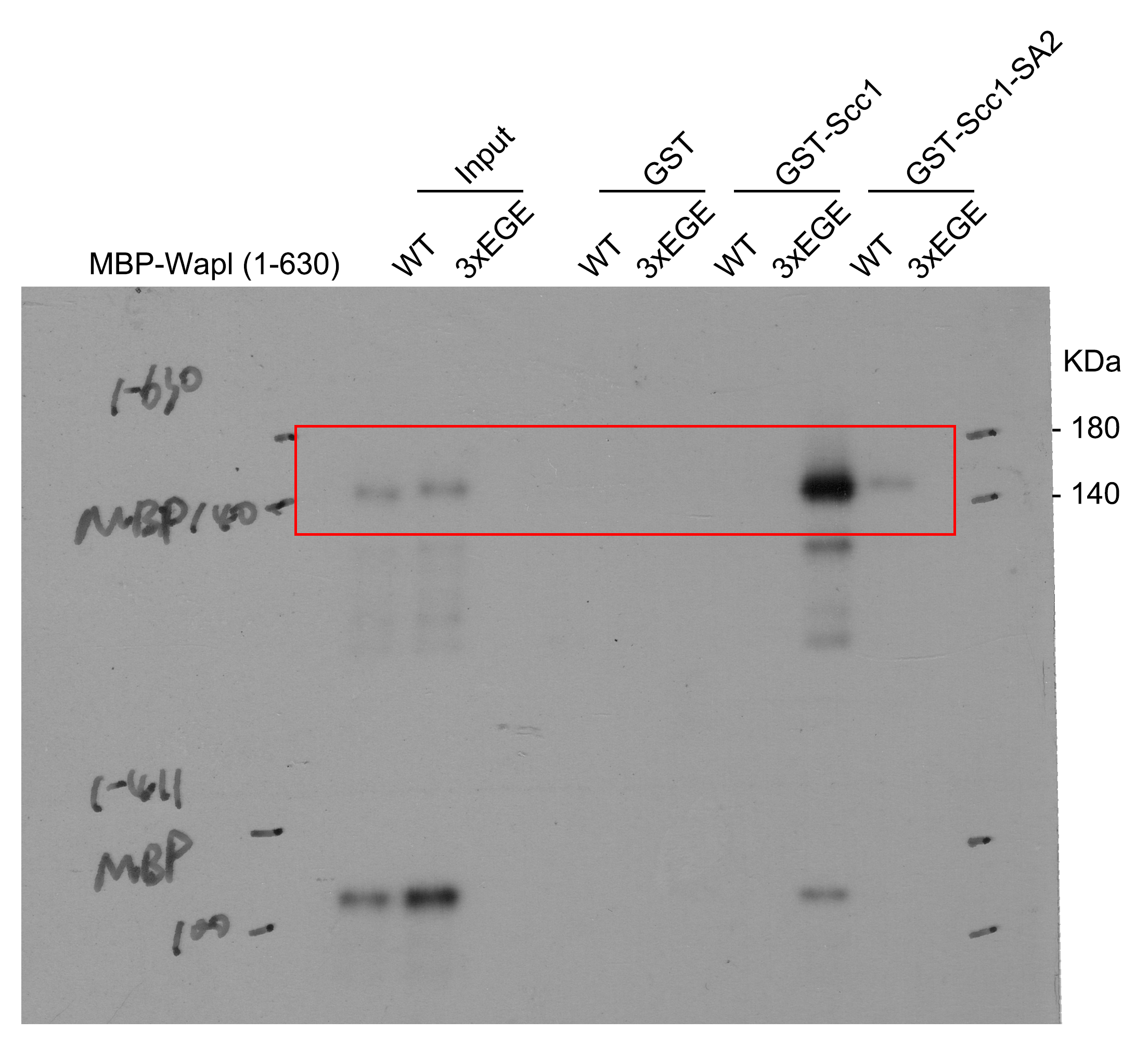

Supplement: Supplementary file 10 — Source data Fig. 6 [file 44318_2024_104_MOESM10_ESM.zip › Figure 6/6C/6C WB.tif]

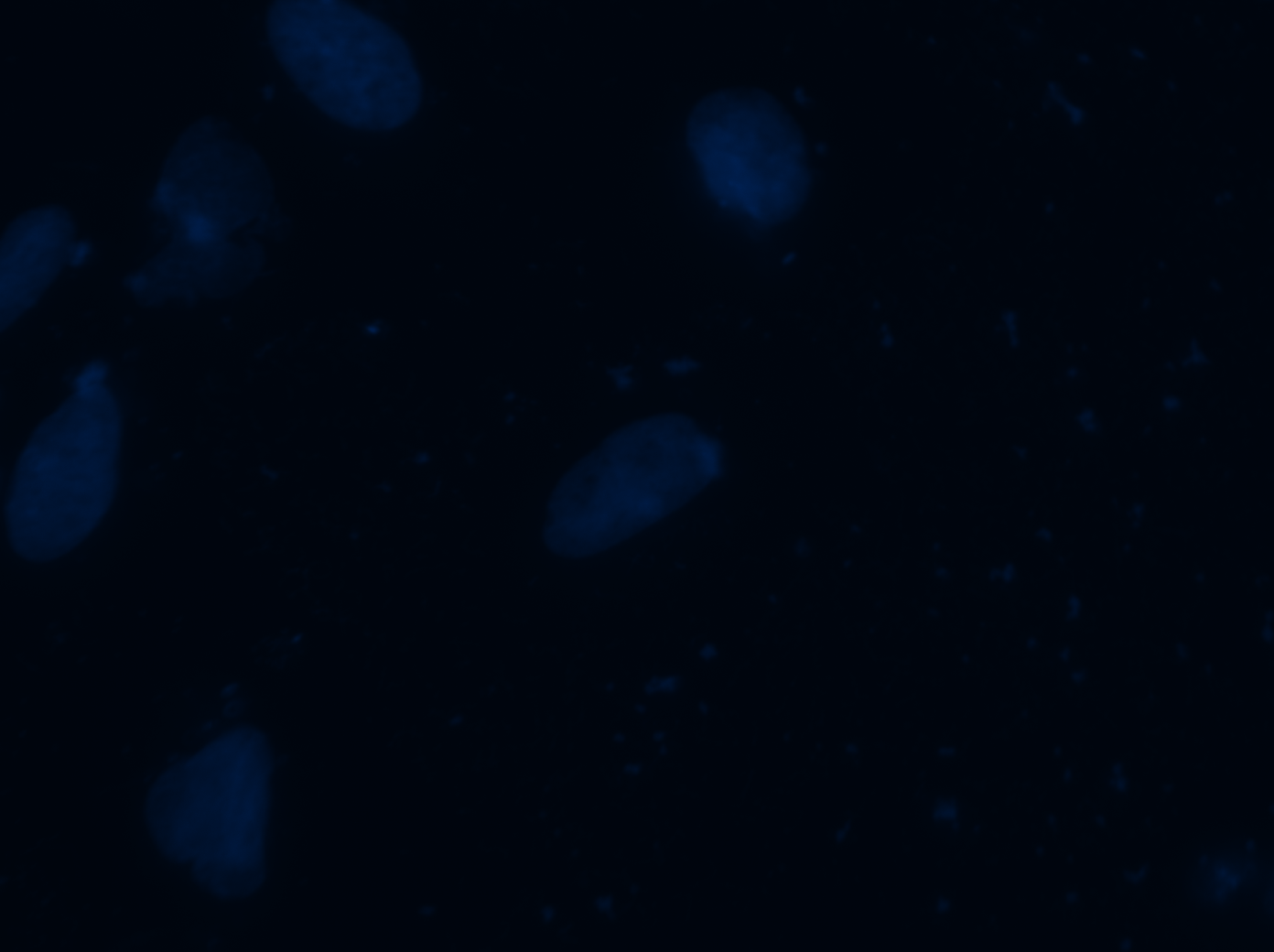

Supplement: Supplementary file 10 — Source data Fig. 6 [file 44318_2024_104_MOESM10_ESM.zip › Figure 6/6D/LacI+Wapl-DNA.tif]

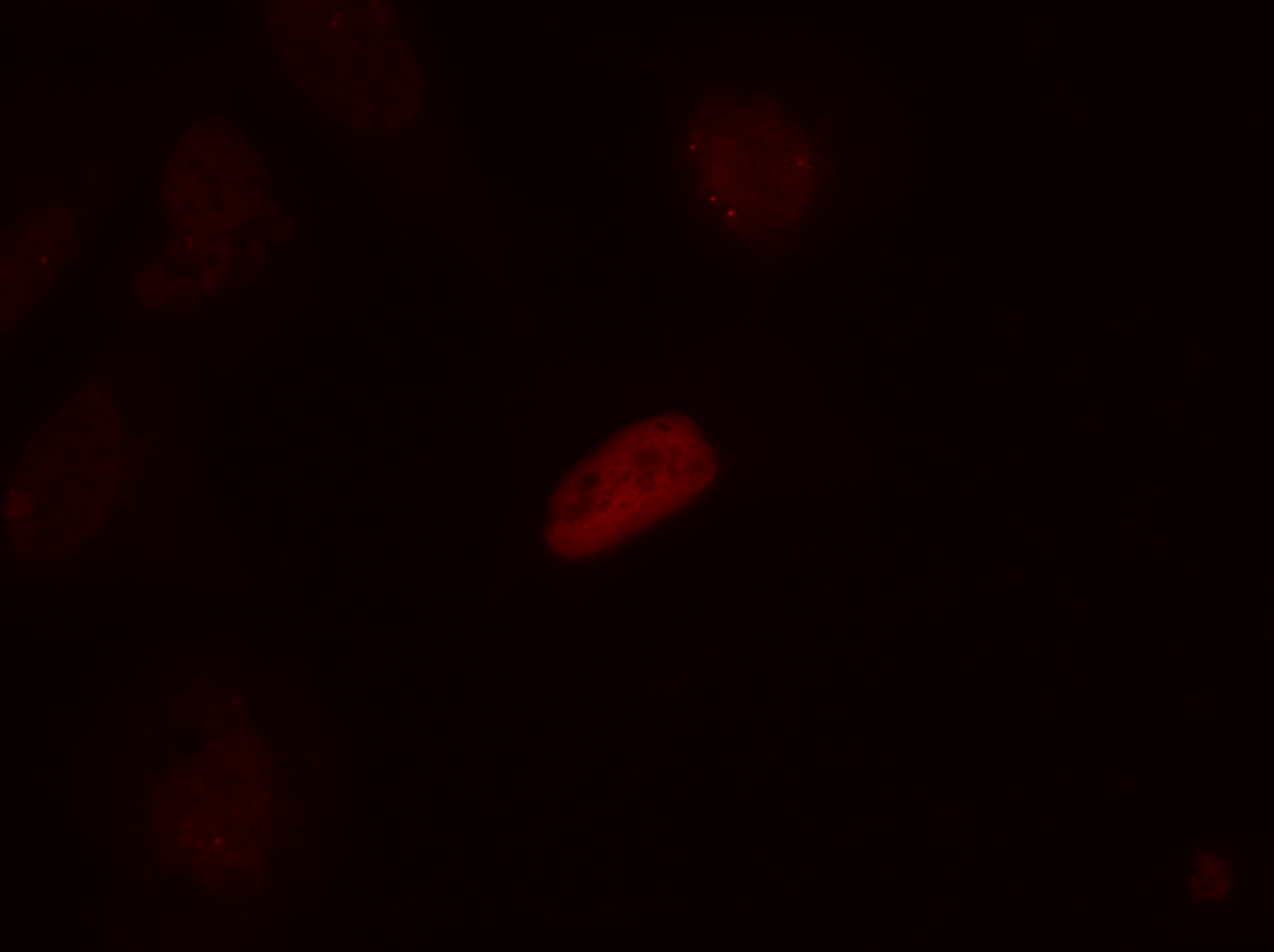

Supplement: Supplementary file 10 — Source data Fig. 6 [file 44318_2024_104_MOESM10_ESM.zip › Figure 6/6D/LacI+Wapl-Flag.tif]

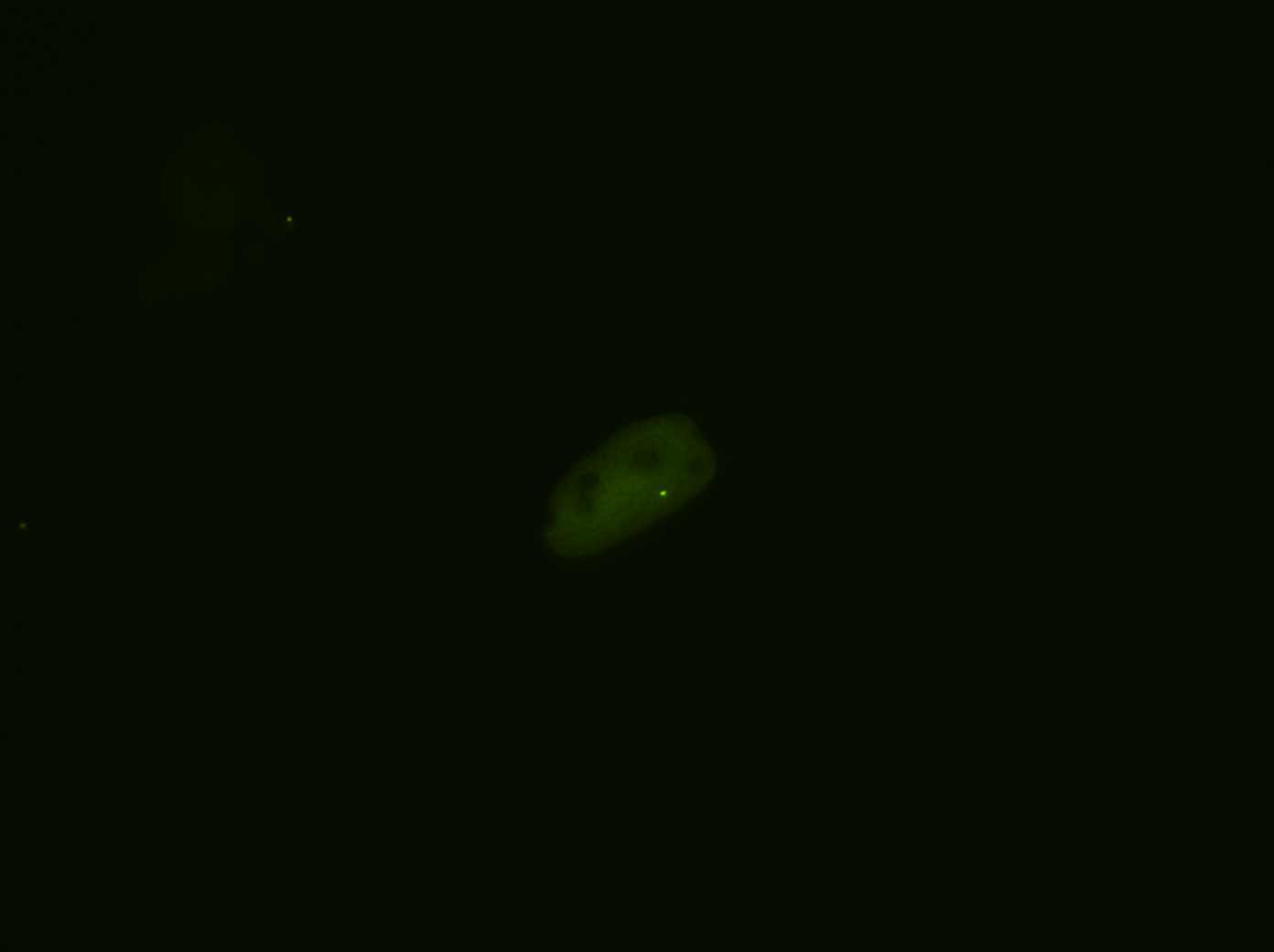

Supplement: Supplementary file 10 — Source data Fig. 6 [file 44318_2024_104_MOESM10_ESM.zip › Figure 6/6D/LacI+Wapl-GFP.tif]

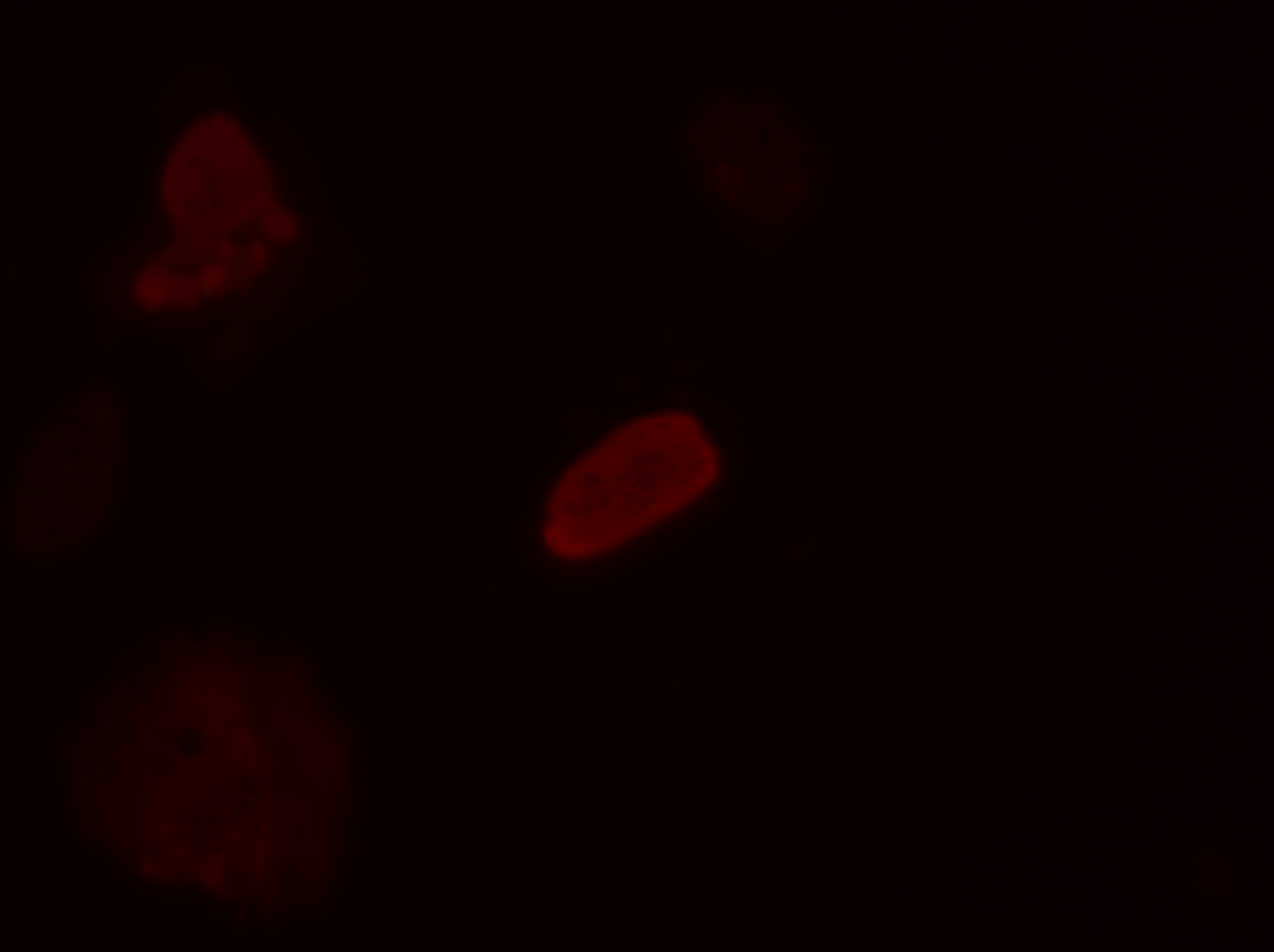

Supplement: Supplementary file 10 — Source data Fig. 6 [file 44318_2024_104_MOESM10_ESM.zip › Figure 6/6D/LacI+Wapl-Myc.tif]

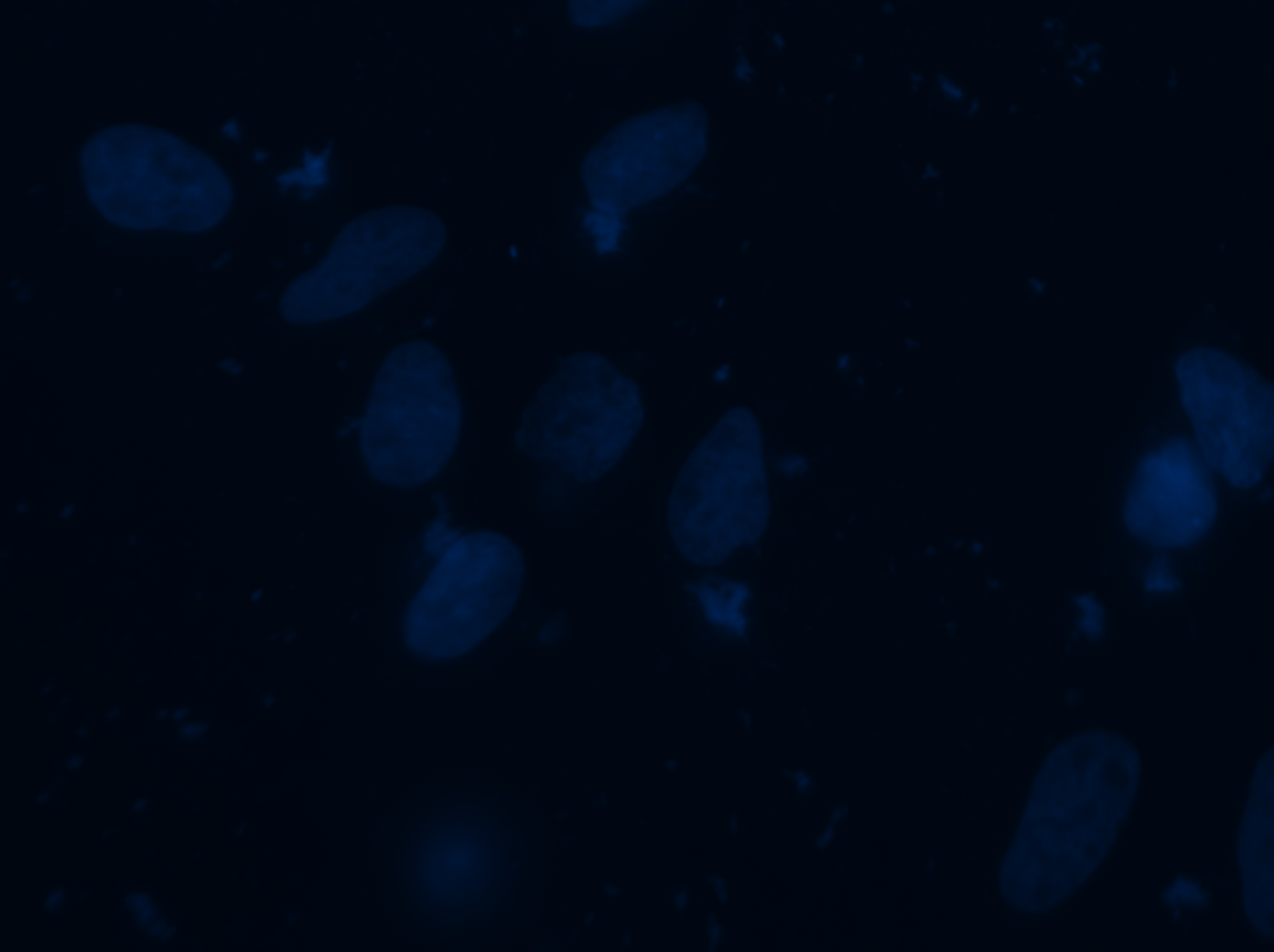

Supplement: Supplementary file 10 — Source data Fig. 6 [file 44318_2024_104_MOESM10_ESM.zip › Figure 6/6D/Scc1 (281-420)+ Wapl-3xEGE-DNA.tif]

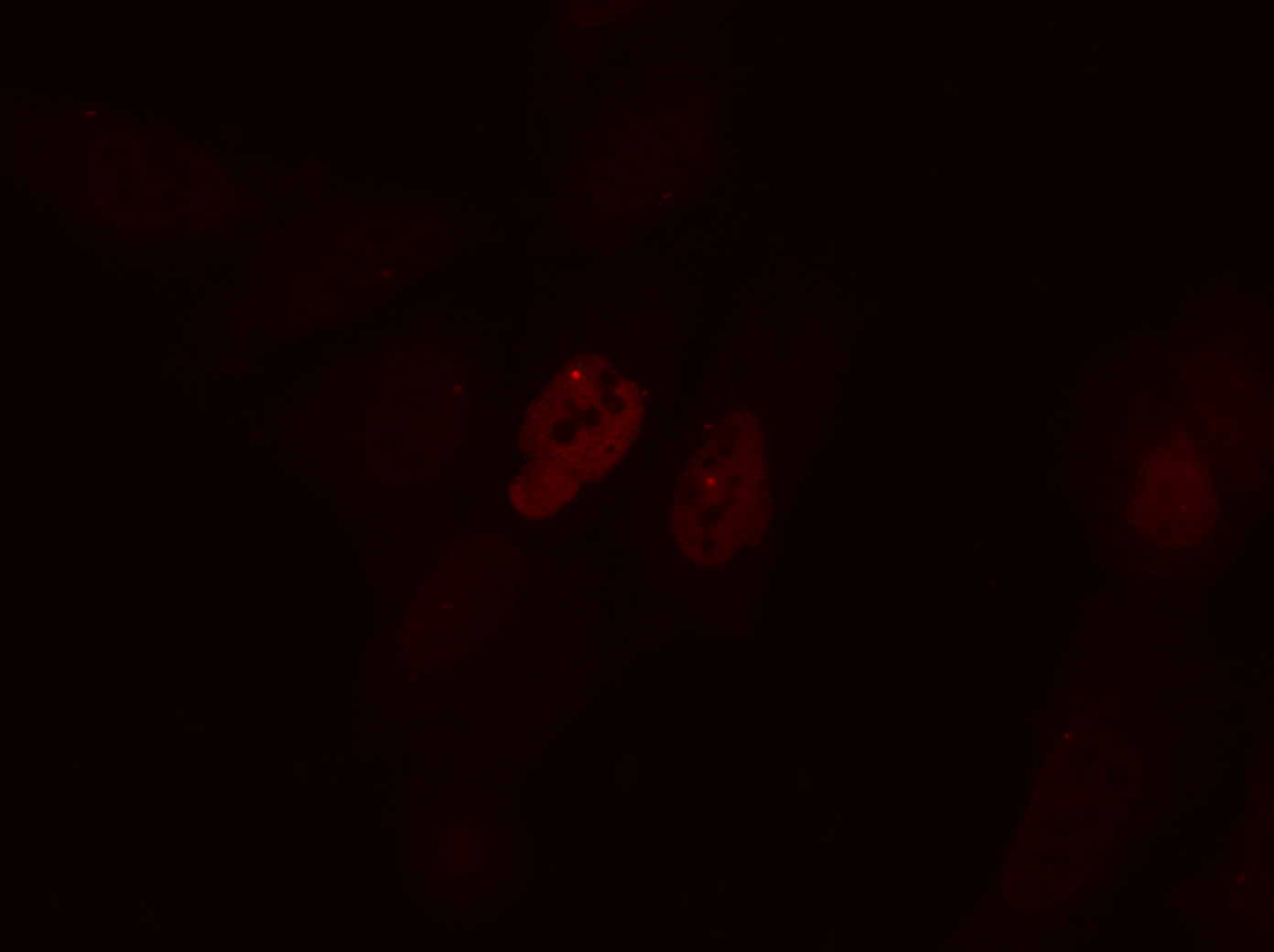

Supplement: Supplementary file 10 — Source data Fig. 6 [file 44318_2024_104_MOESM10_ESM.zip › Figure 6/6D/Scc1 (281-420)+ Wapl-3xEGE-Flag.tif]

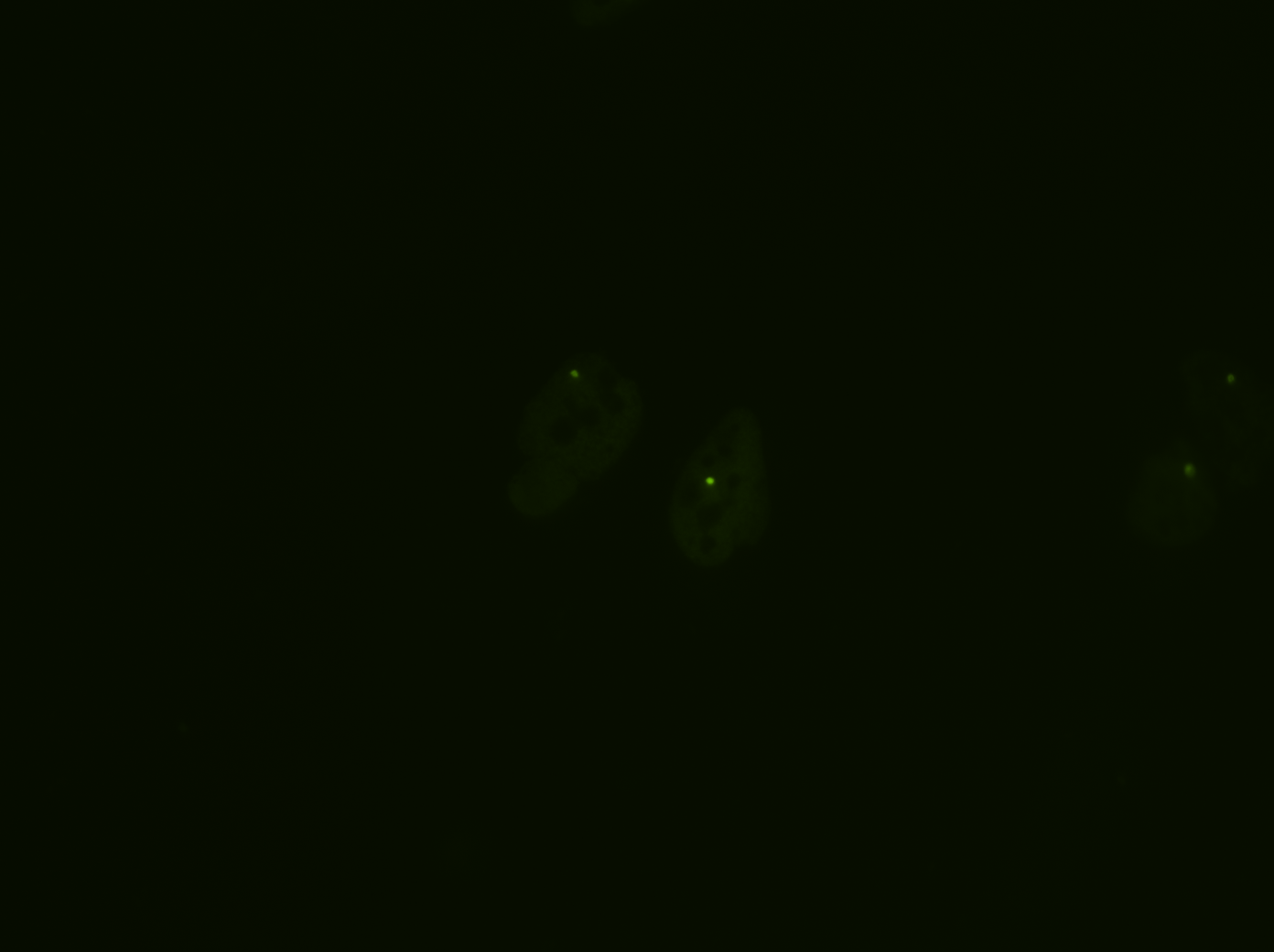

Supplement: Supplementary file 10 — Source data Fig. 6 [file 44318_2024_104_MOESM10_ESM.zip › Figure 6/6D/Scc1 (281-420)+ Wapl-3xEGE-GFP.tif]

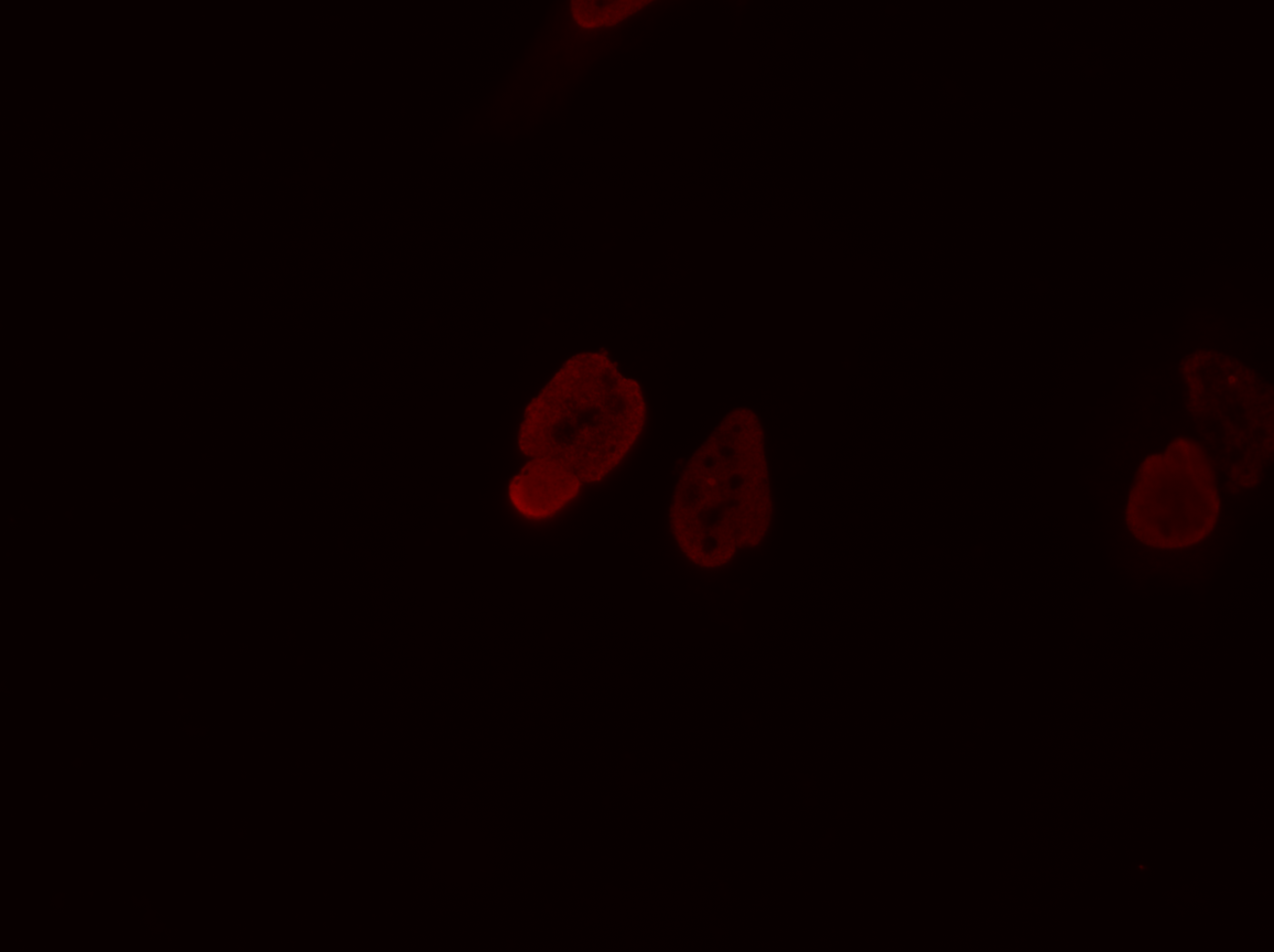

Supplement: Supplementary file 10 — Source data Fig. 6 [file 44318_2024_104_MOESM10_ESM.zip › Figure 6/6D/Scc1 (281-420)+ Wapl-3xEGE-Myc.tif]

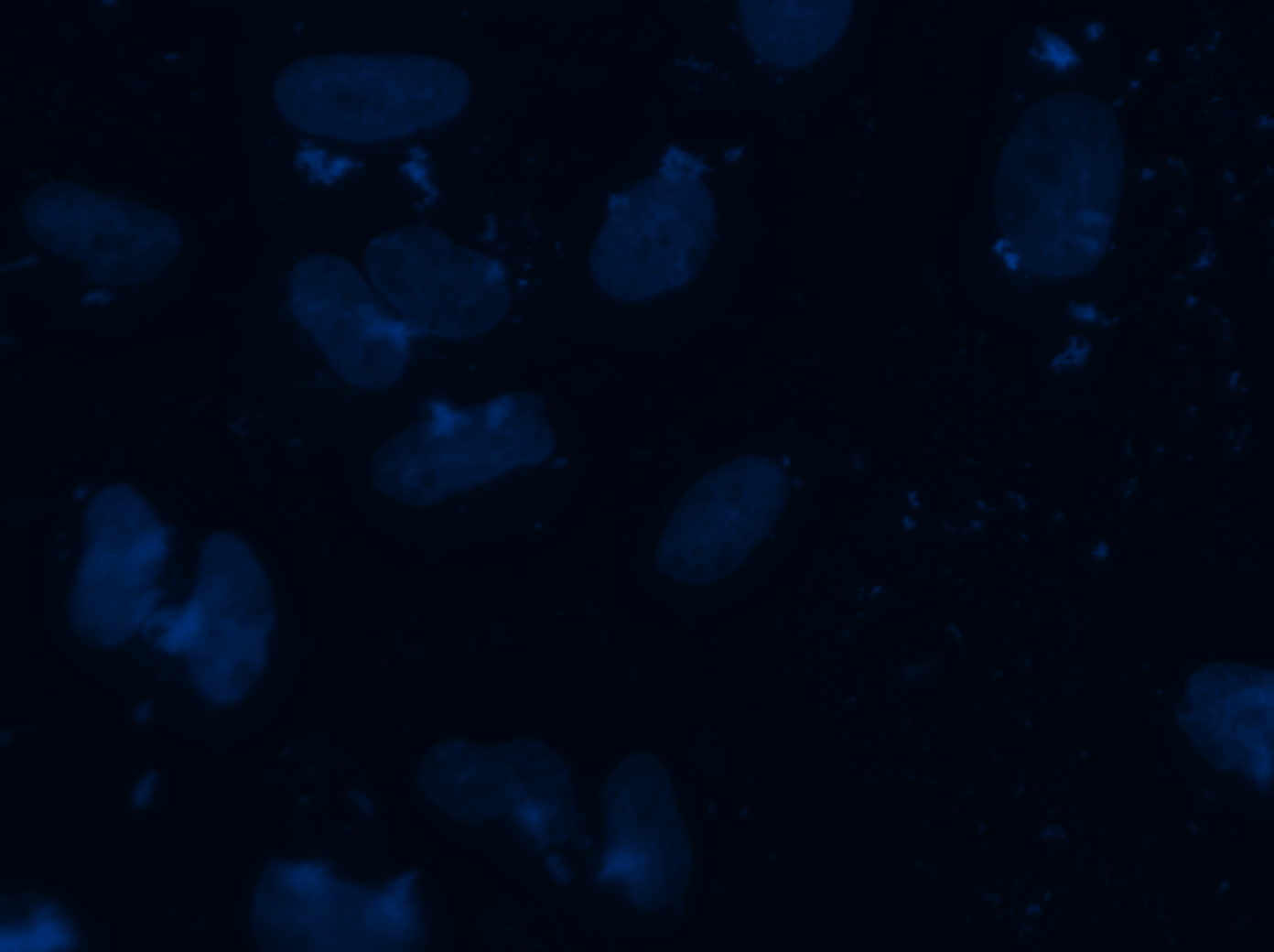

Supplement: Supplementary file 10 — Source data Fig. 6 [file 44318_2024_104_MOESM10_ESM.zip › Figure 6/6D/Scc1 (281-420)+ Wapl-DNA.tif]

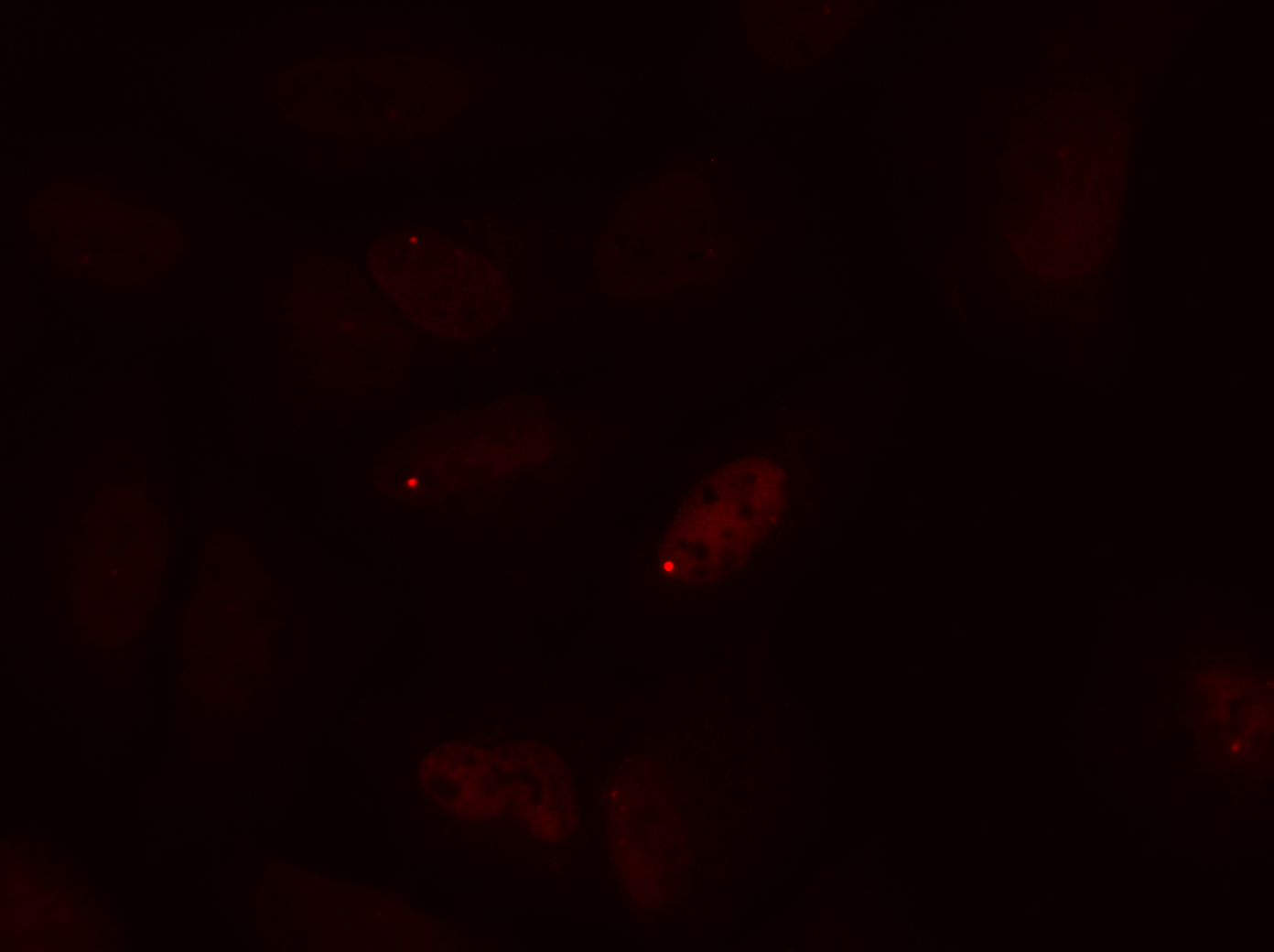

Supplement: Supplementary file 10 — Source data Fig. 6 [file 44318_2024_104_MOESM10_ESM.zip › Figure 6/6D/Scc1 (281-420)+ Wapl-Flag.tif]

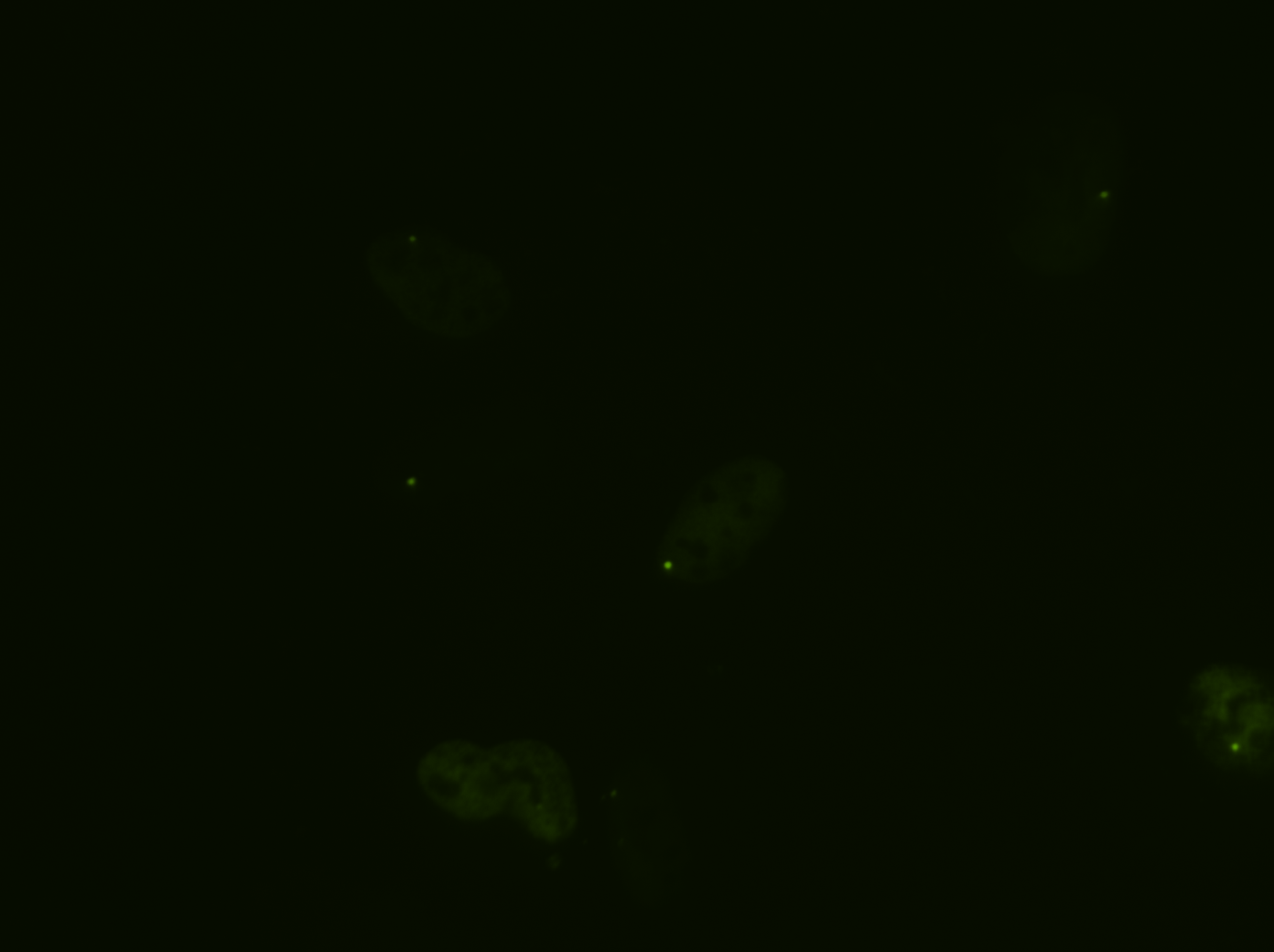

Supplement: Supplementary file 10 — Source data Fig. 6 [file 44318_2024_104_MOESM10_ESM.zip › Figure 6/6D/Scc1 (281-420)+ Wapl-GFP.tif]

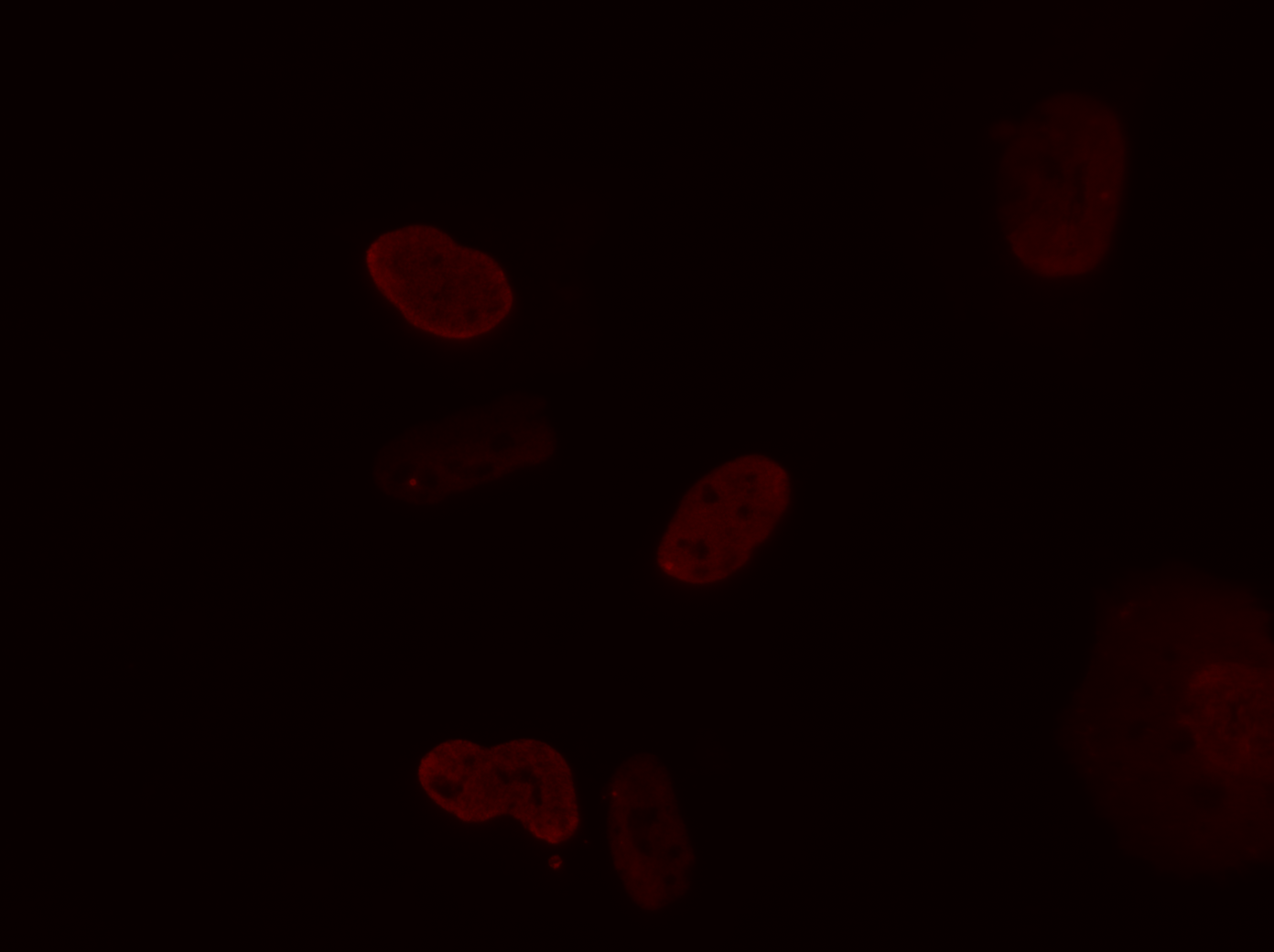

Supplement: Supplementary file 10 — Source data Fig. 6 [file 44318_2024_104_MOESM10_ESM.zip › Figure 6/6D/Scc1 (281-420)+ Wapl-Myc.tif]

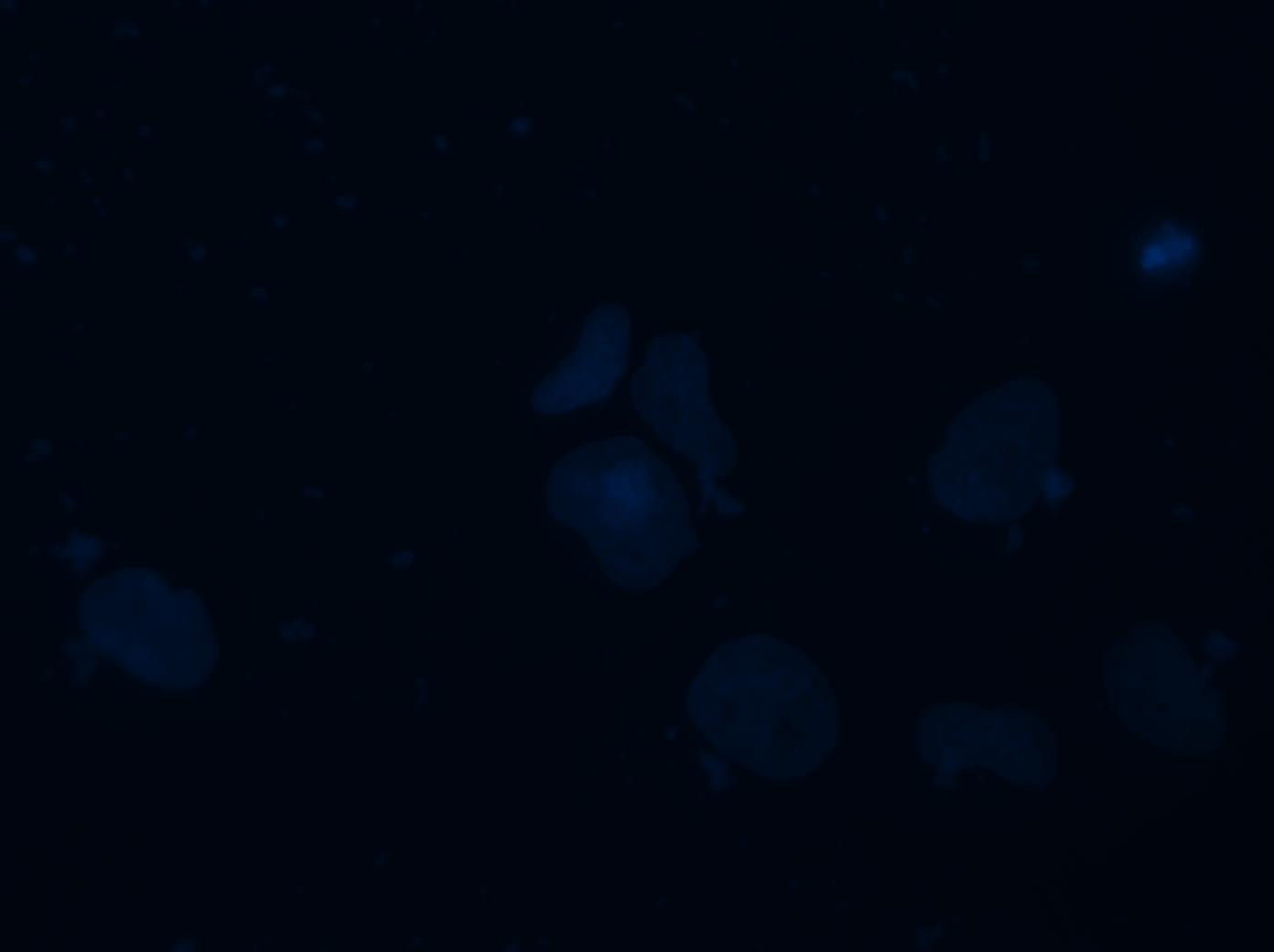

Supplement: Supplementary file 10 — Source data Fig. 6 [file 44318_2024_104_MOESM10_ESM.zip › Figure 6/6F/EGFP-LacI+CENP-U-DNA.tif]

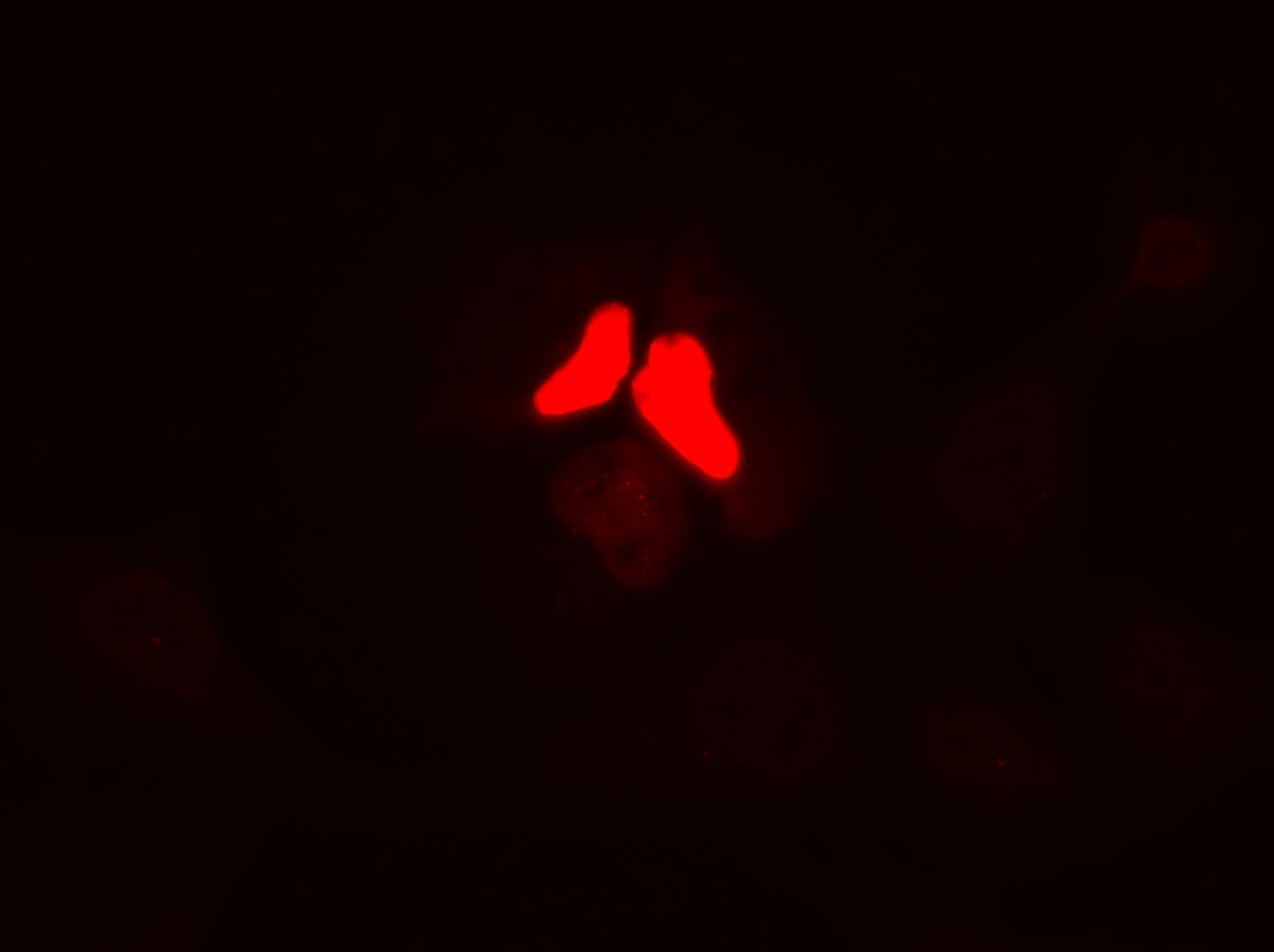

Supplement: Supplementary file 10 — Source data Fig. 6 [file 44318_2024_104_MOESM10_ESM.zip › Figure 6/6F/EGFP-LacI+CENP-U-Flag.tif]

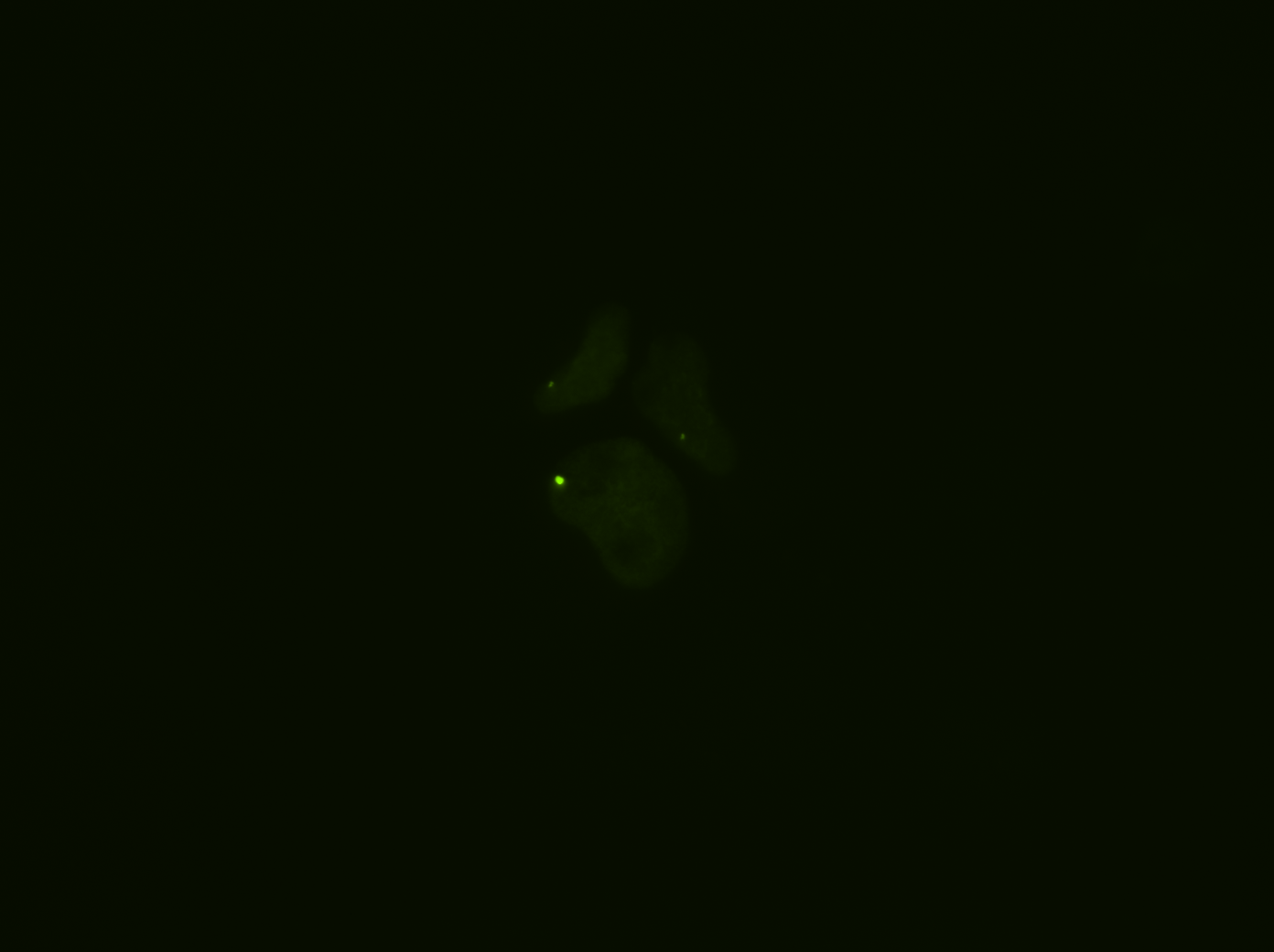

Supplement: Supplementary file 10 — Source data Fig. 6 [file 44318_2024_104_MOESM10_ESM.zip › Figure 6/6F/EGFP-LacI+CENP-U-GFP.tif]

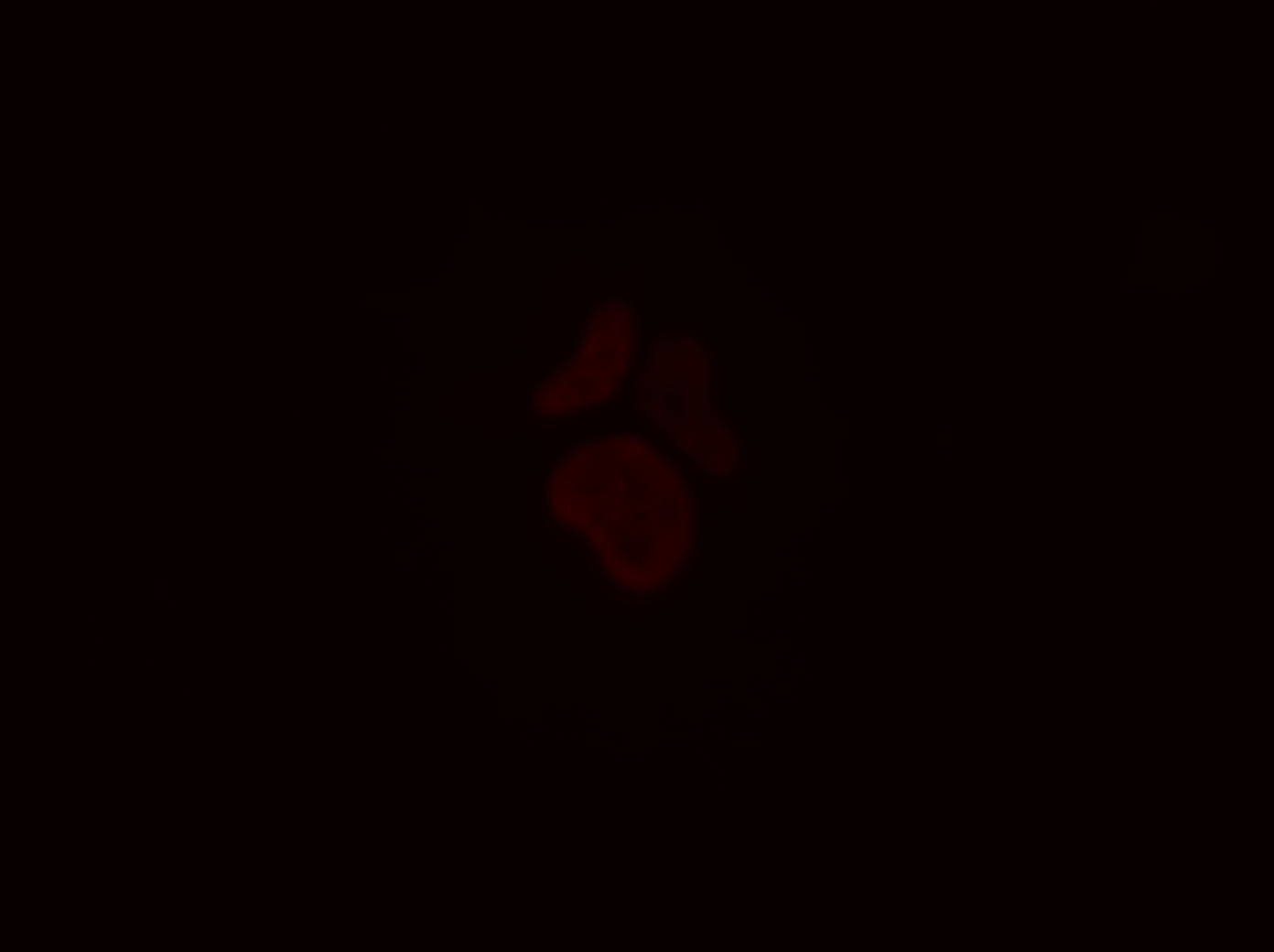

Supplement: Supplementary file 10 — Source data Fig. 6 [file 44318_2024_104_MOESM10_ESM.zip › Figure 6/6F/EGFP-LacI+CENP-U-Myc.tif]

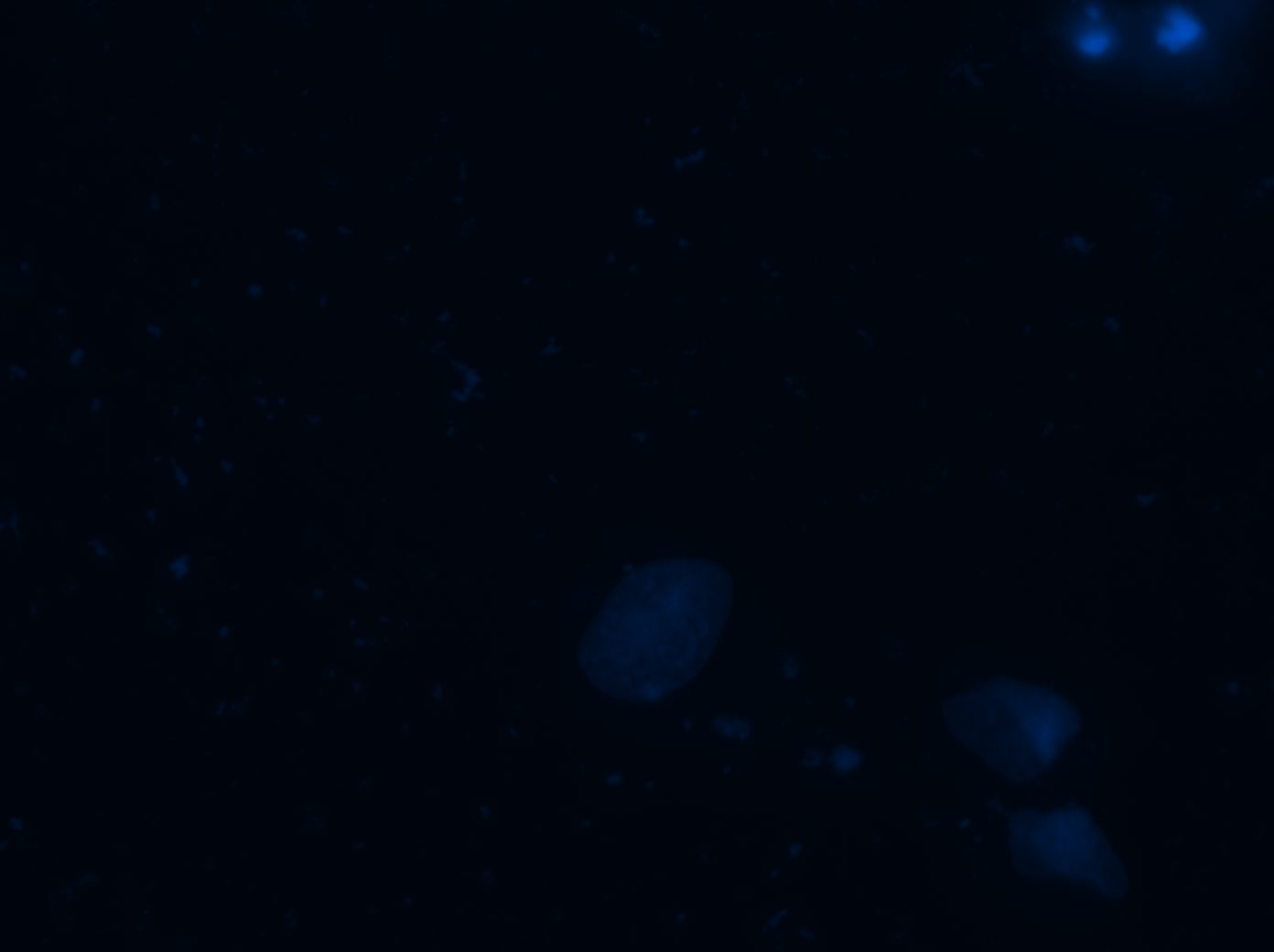

Supplement: Supplementary file 10 — Source data Fig. 6 [file 44318_2024_104_MOESM10_ESM.zip › Figure 6/6F/Scc1 (281-420)+CENP-U-ADA-DNA.tif]

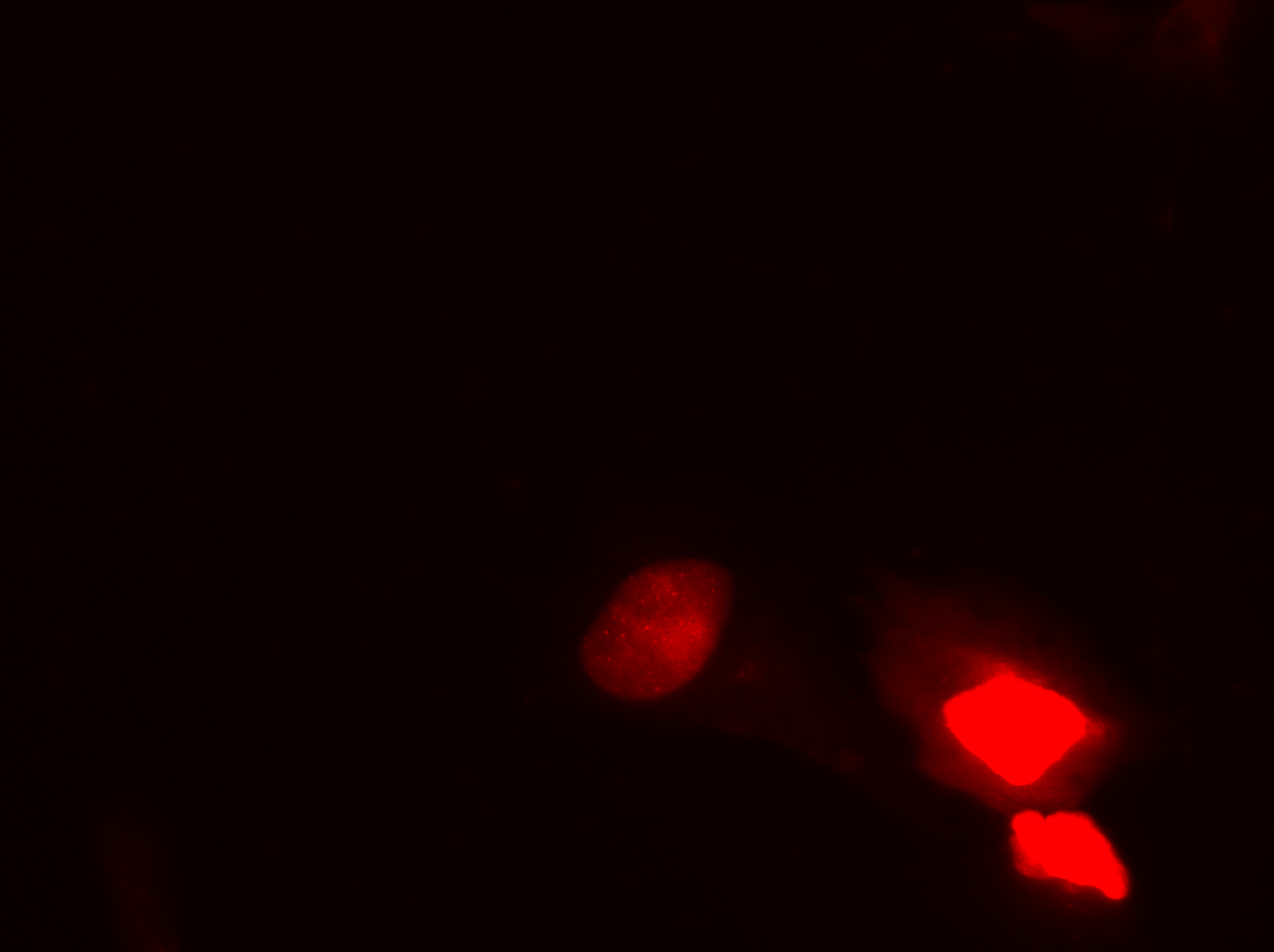

Supplement: Supplementary file 10 — Source data Fig. 6 [file 44318_2024_104_MOESM10_ESM.zip › Figure 6/6F/Scc1 (281-420)+CENP-U-ADA-Flag.tif]

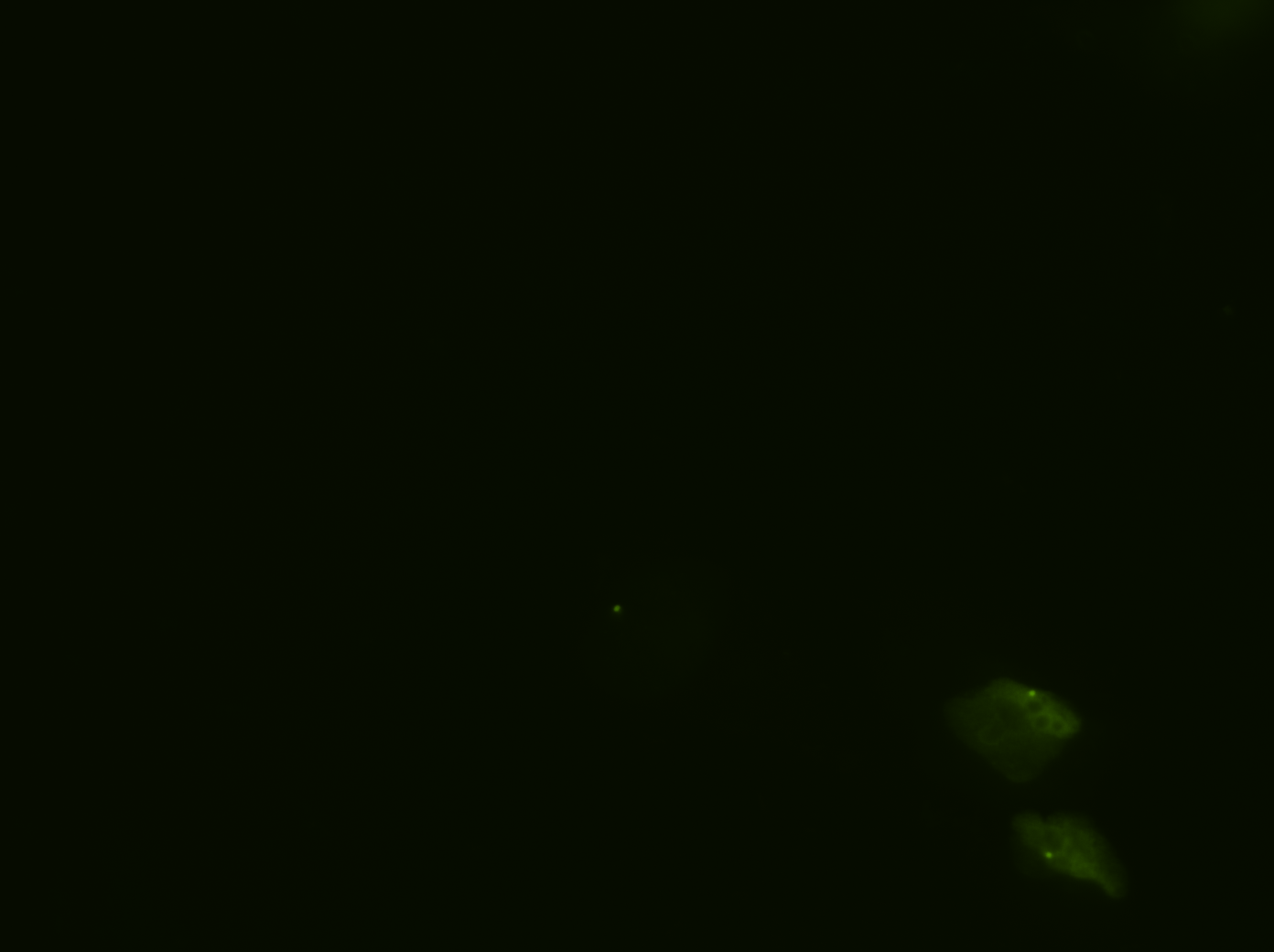

Supplement: Supplementary file 10 — Source data Fig. 6 [file 44318_2024_104_MOESM10_ESM.zip › Figure 6/6F/Scc1 (281-420)+CENP-U-ADA-GFP.tif]

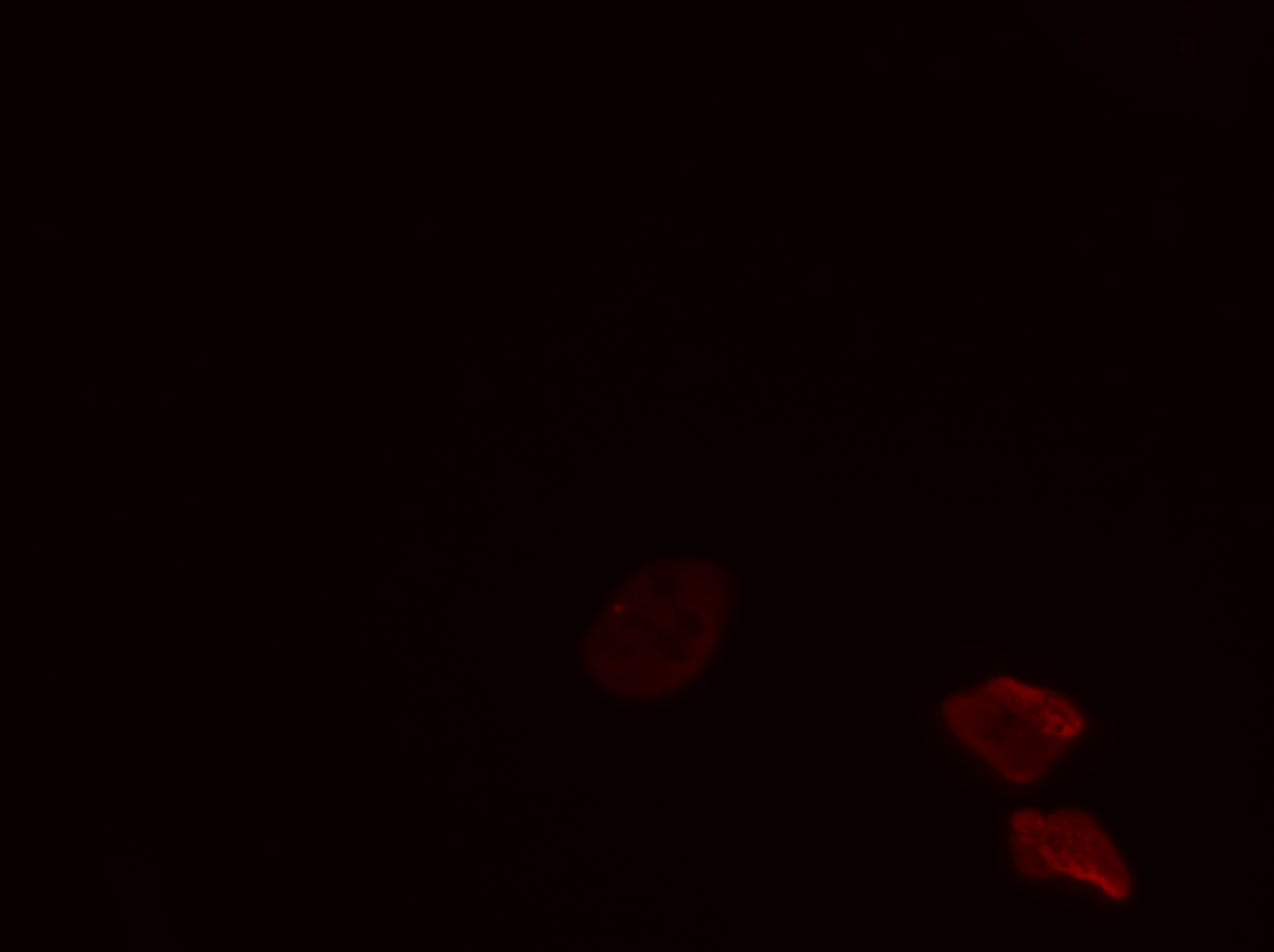

Supplement: Supplementary file 10 — Source data Fig. 6 [file 44318_2024_104_MOESM10_ESM.zip › Figure 6/6F/Scc1 (281-420)+CENP-U-ADA-Myc.tif]

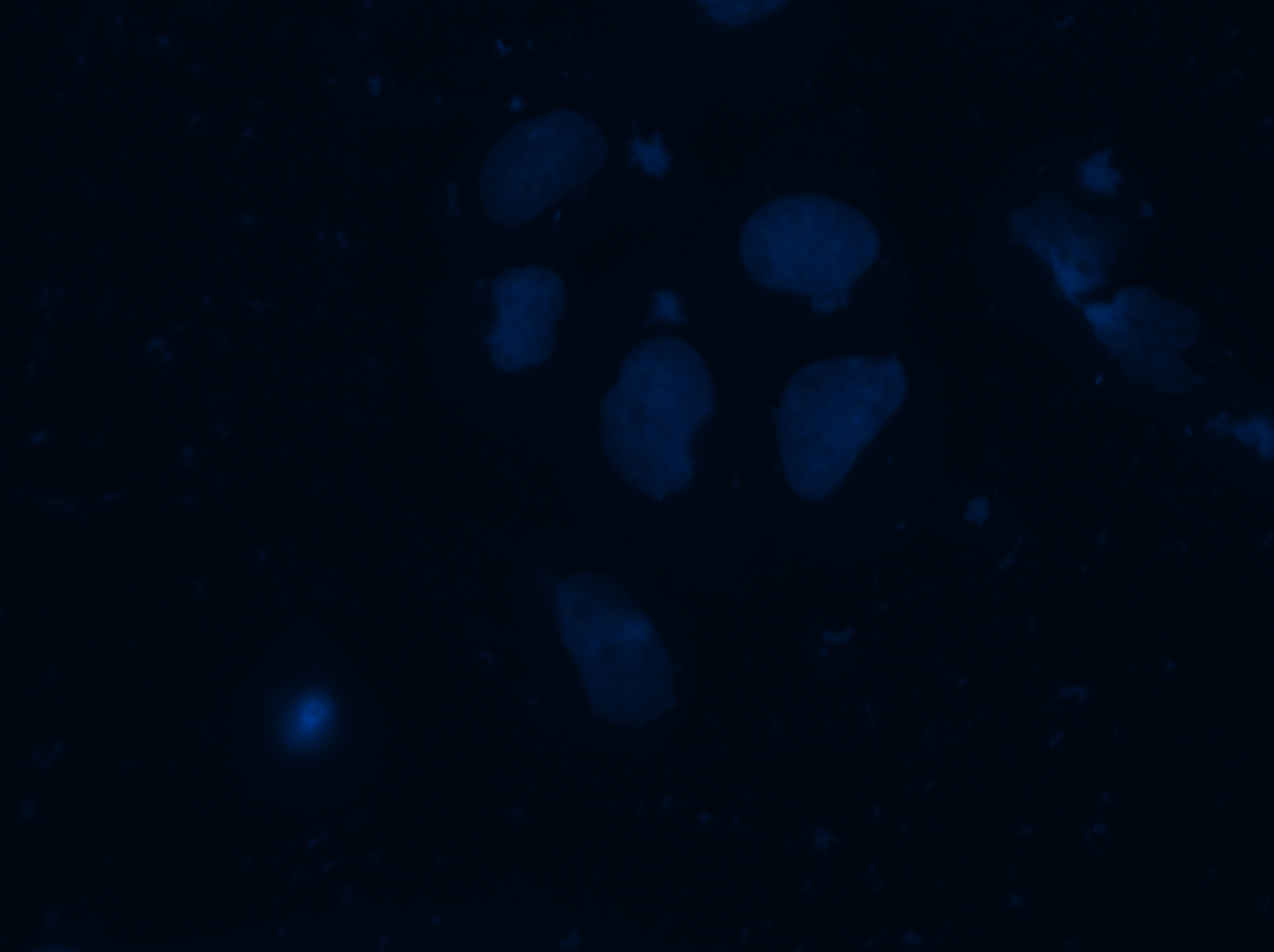

Supplement: Supplementary file 10 — Source data Fig. 6 [file 44318_2024_104_MOESM10_ESM.zip › Figure 6/6F/Scc1 (281-420)+CENP-U-DNA.tif]

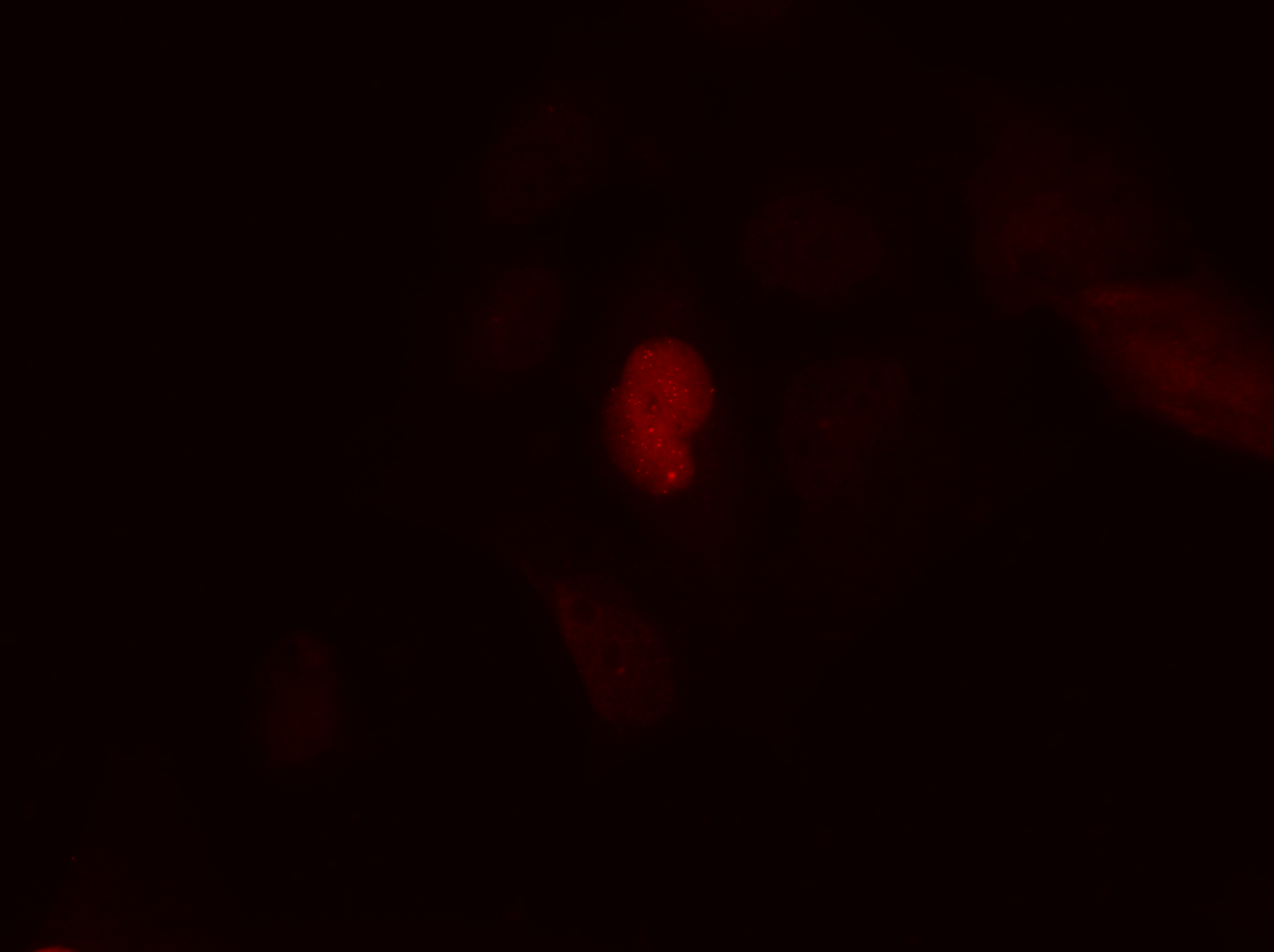

Supplement: Supplementary file 10 — Source data Fig. 6 [file 44318_2024_104_MOESM10_ESM.zip › Figure 6/6F/Scc1 (281-420)+CENP-U-Flag.tif]

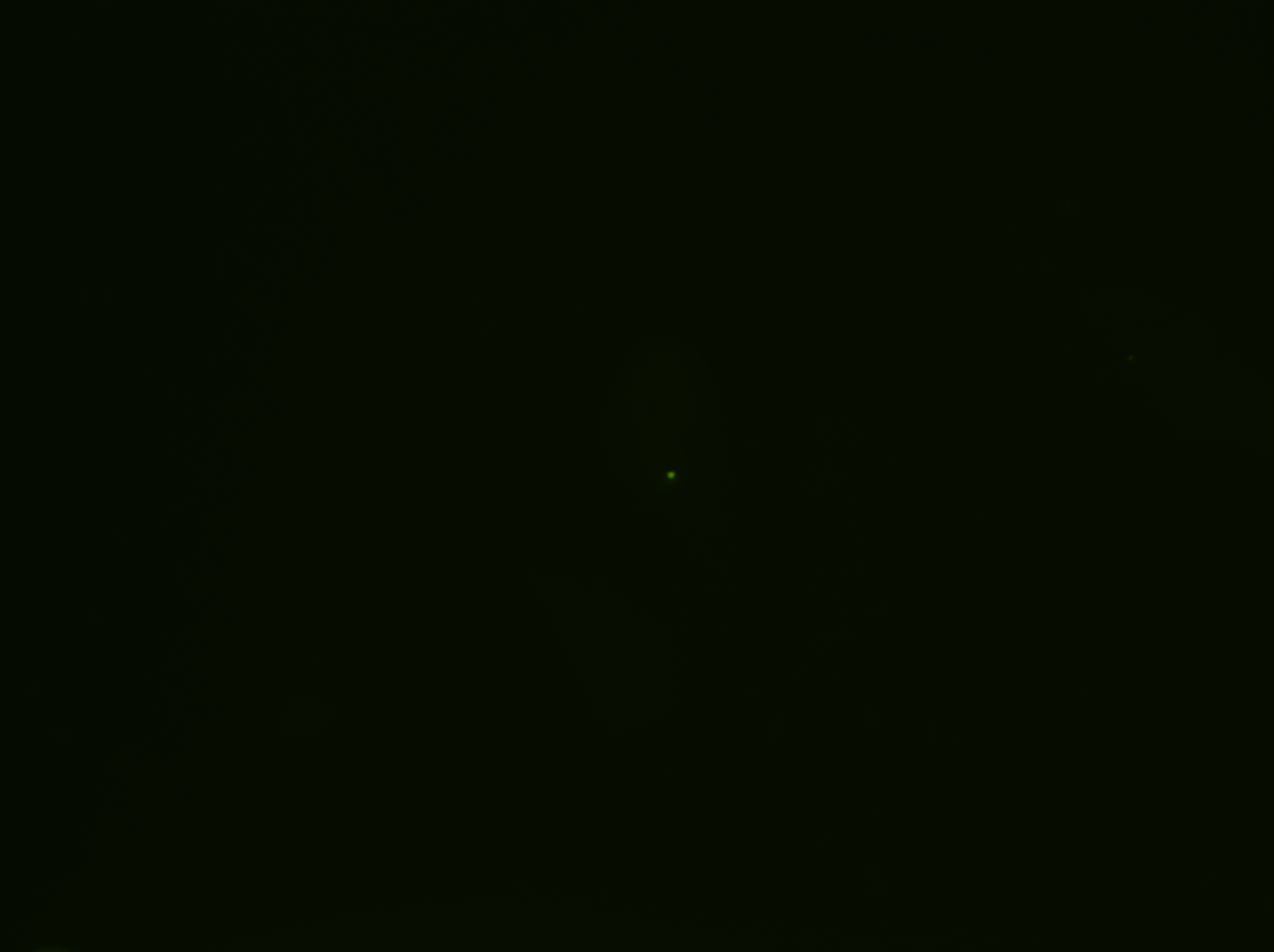

Supplement: Supplementary file 10 — Source data Fig. 6 [file 44318_2024_104_MOESM10_ESM.zip › Figure 6/6F/Scc1 (281-420)+CENP-U-GFP.tif]

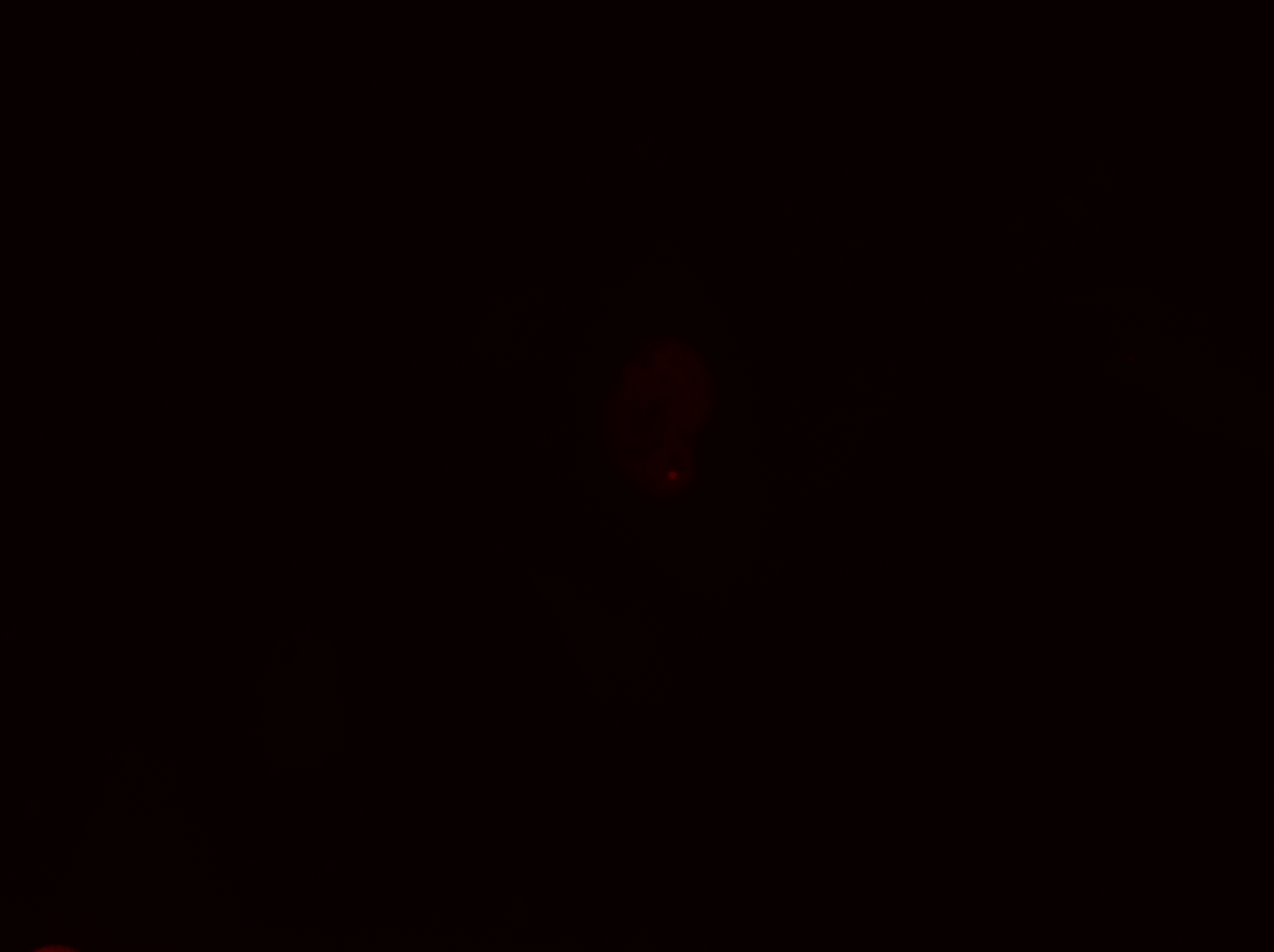

Supplement: Supplementary file 10 — Source data Fig. 6 [file 44318_2024_104_MOESM10_ESM.zip › Figure 6/6F/Scc1 (281-420)+CENP-U-Myc.tif]

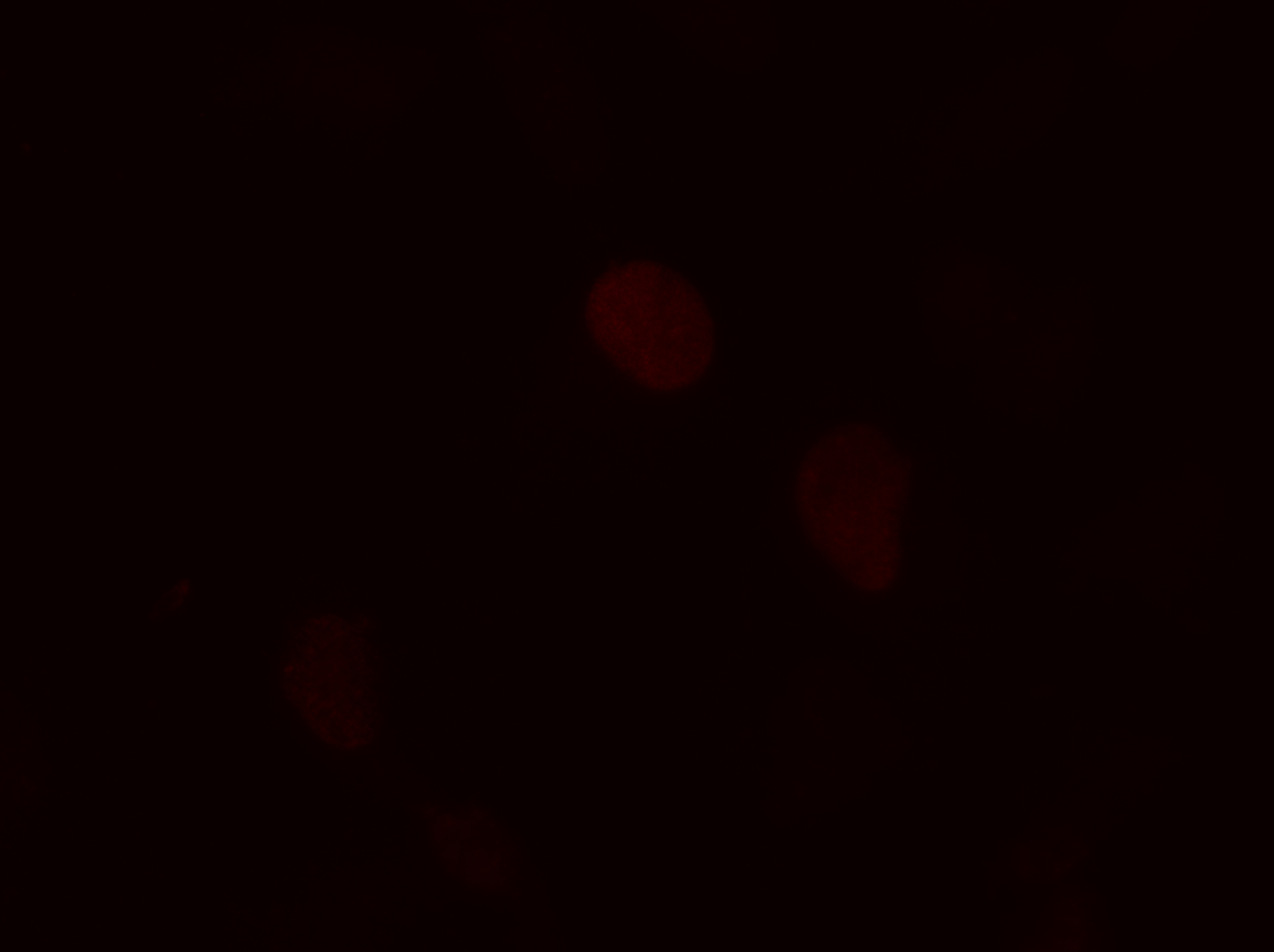

Supplement: Supplementary file 11 — Source data Fig. 7 [file 44318_2024_104_MOESM11_ESM.zip › Figure 7/7A/EGFP-LacI+Myc-SA2 Anti-Myc.tif]

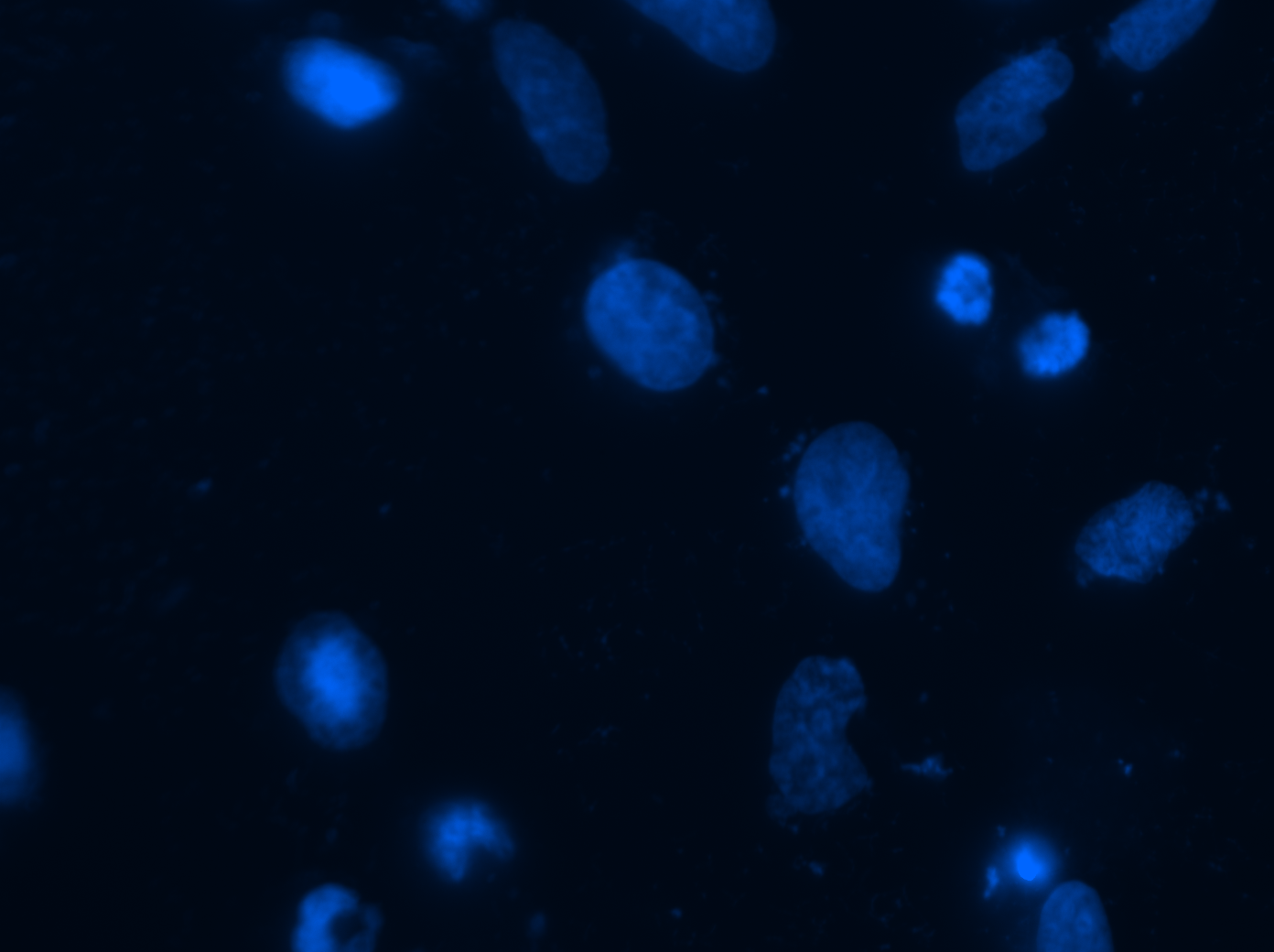

Supplement: Supplementary file 11 — Source data Fig. 7 [file 44318_2024_104_MOESM11_ESM.zip › Figure 7/7A/EGFP-LacI+Myc-SA2 DNA.tif]

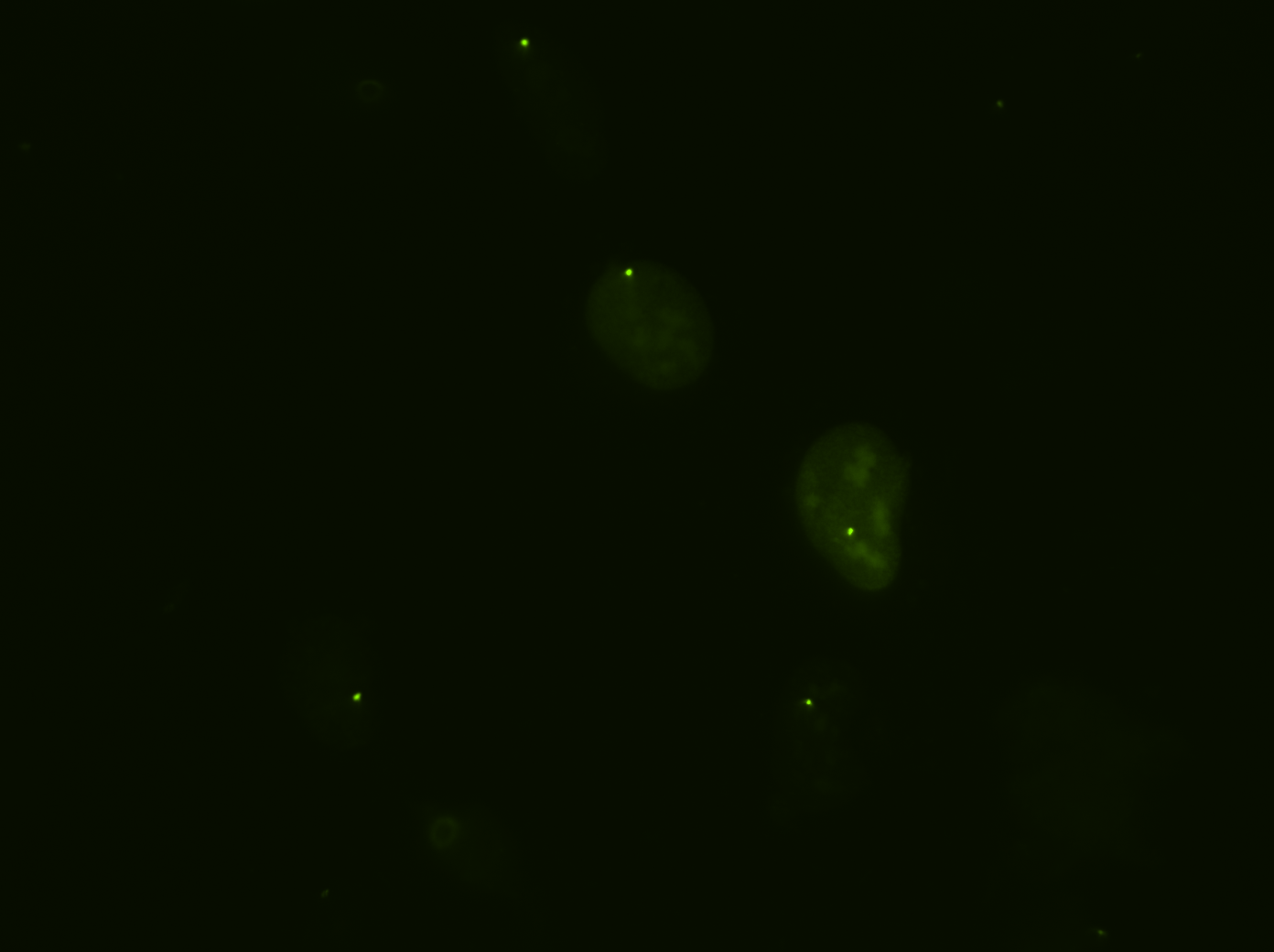

Supplement: Supplementary file 11 — Source data Fig. 7 [file 44318_2024_104_MOESM11_ESM.zip › Figure 7/7A/EGFP-LacI+Myc-SA2 EGFP.tif]

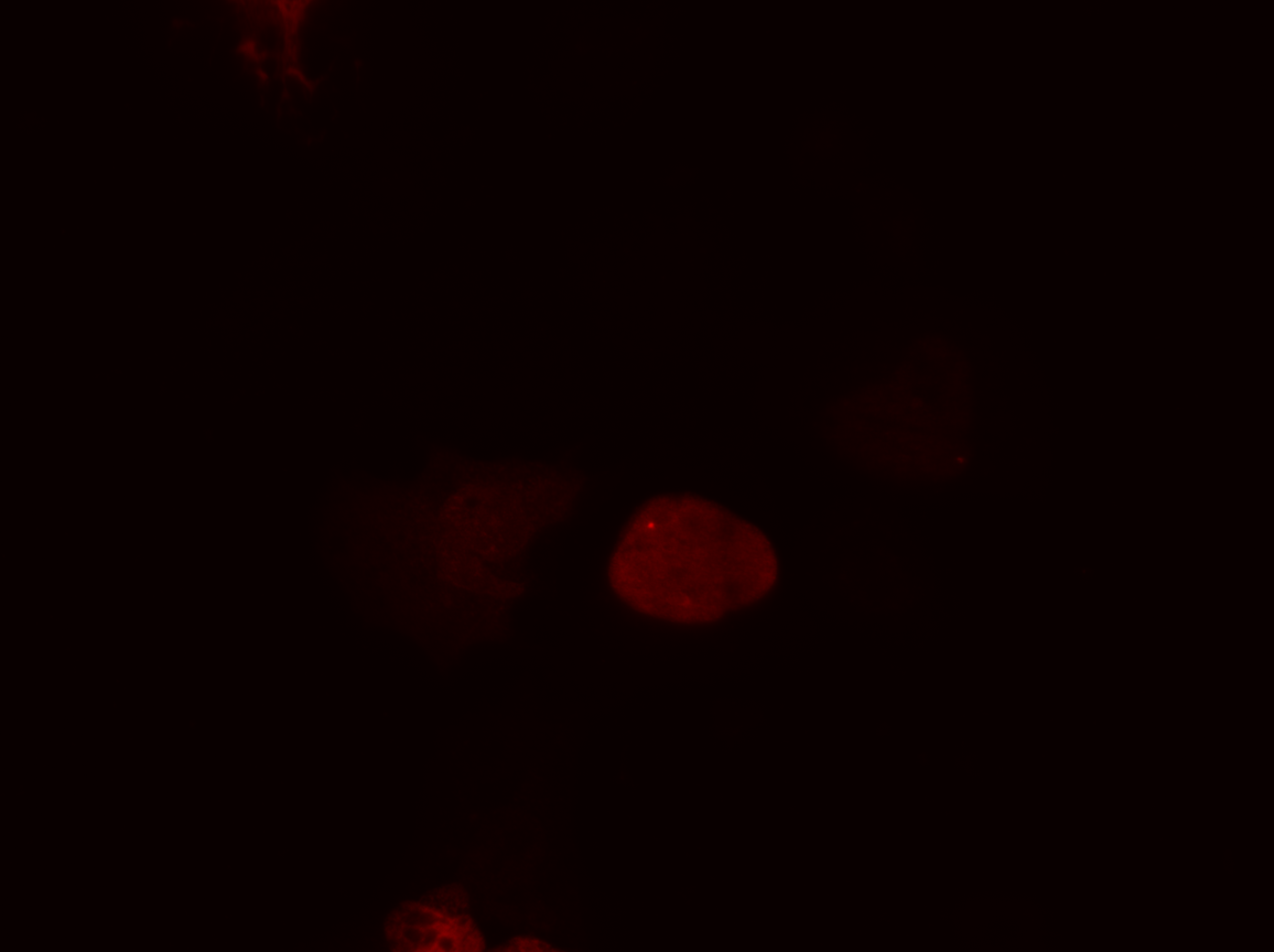

Supplement: Supplementary file 11 — Source data Fig. 7 [file 44318_2024_104_MOESM11_ESM.zip › Figure 7/7A/EGFP-LacI-Scc1+Myc-SA2 Anti-Myc.tif]

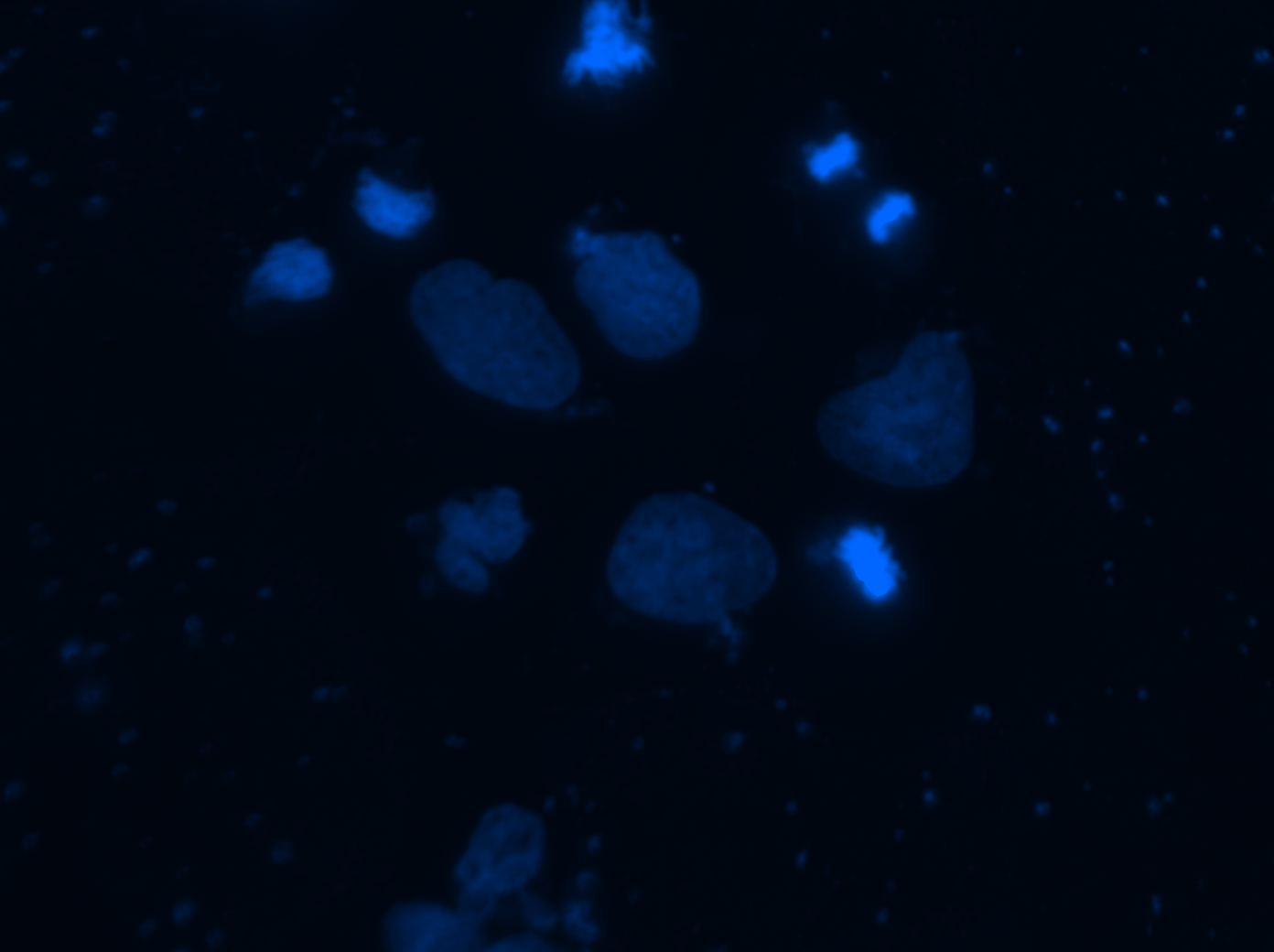

Supplement: Supplementary file 11 — Source data Fig. 7 [file 44318_2024_104_MOESM11_ESM.zip › Figure 7/7A/EGFP-LacI-Scc1+Myc-SA2 DNA.tif]

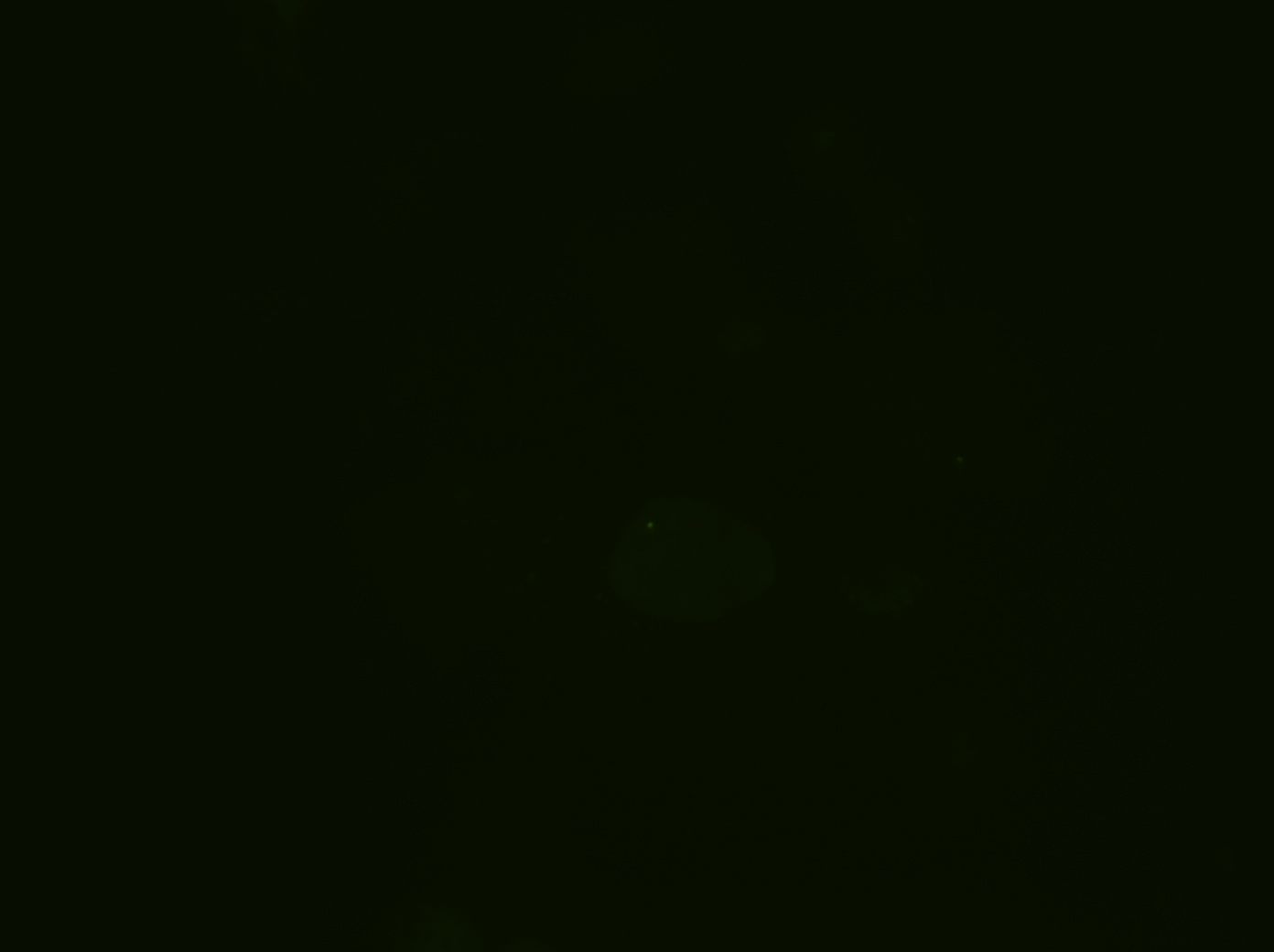

Supplement: Supplementary file 11 — Source data Fig. 7 [file 44318_2024_104_MOESM11_ESM.zip › Figure 7/7A/EGFP-LacI-Scc1+Myc-SA2 EGFP.tif]

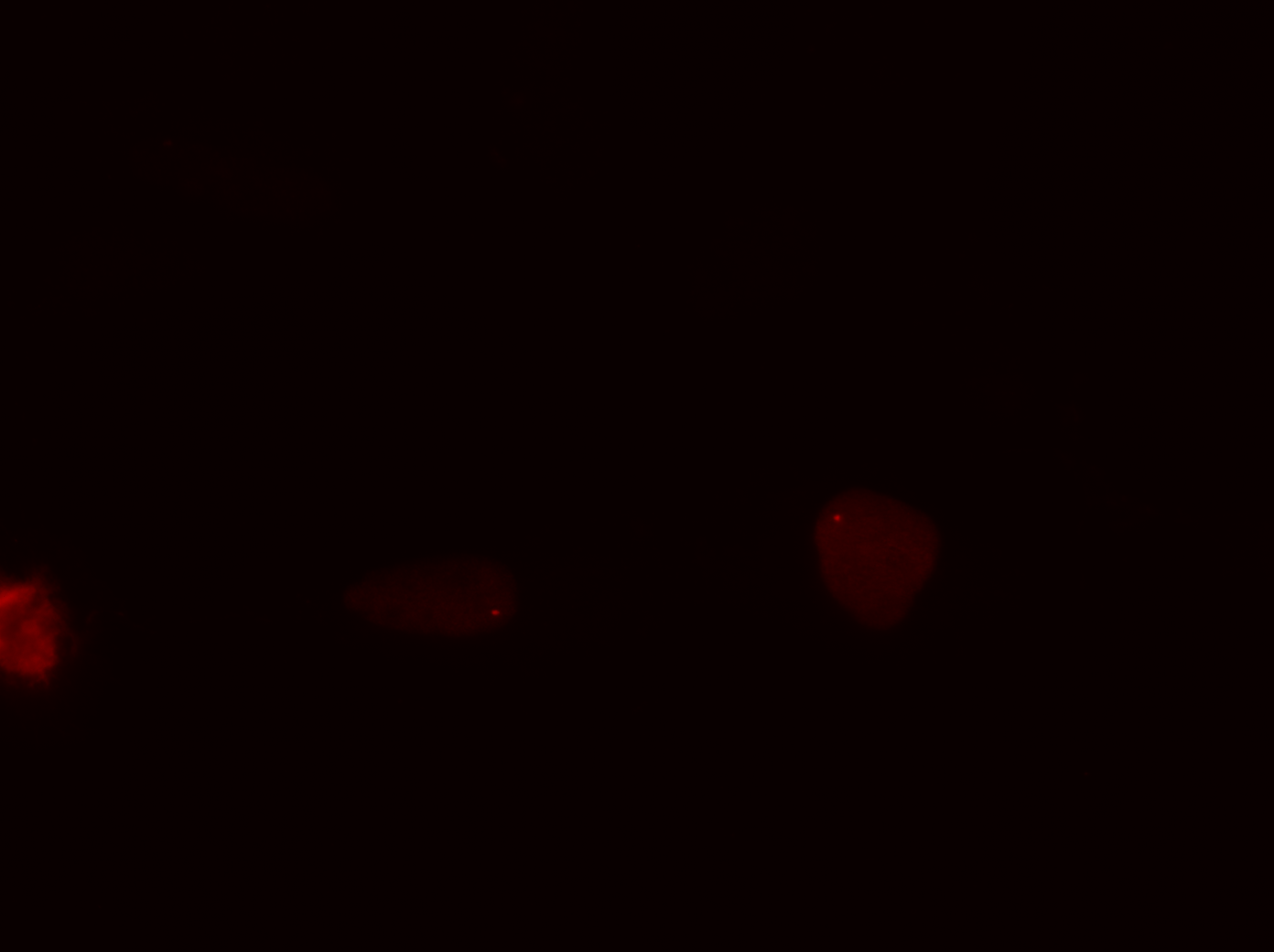

Supplement: Supplementary file 11 — Source data Fig. 7 [file 44318_2024_104_MOESM11_ESM.zip › Figure 7/7A/EGFP-LacI-Scc1+Myc-SA2-W334A Anti-Myc.tif]

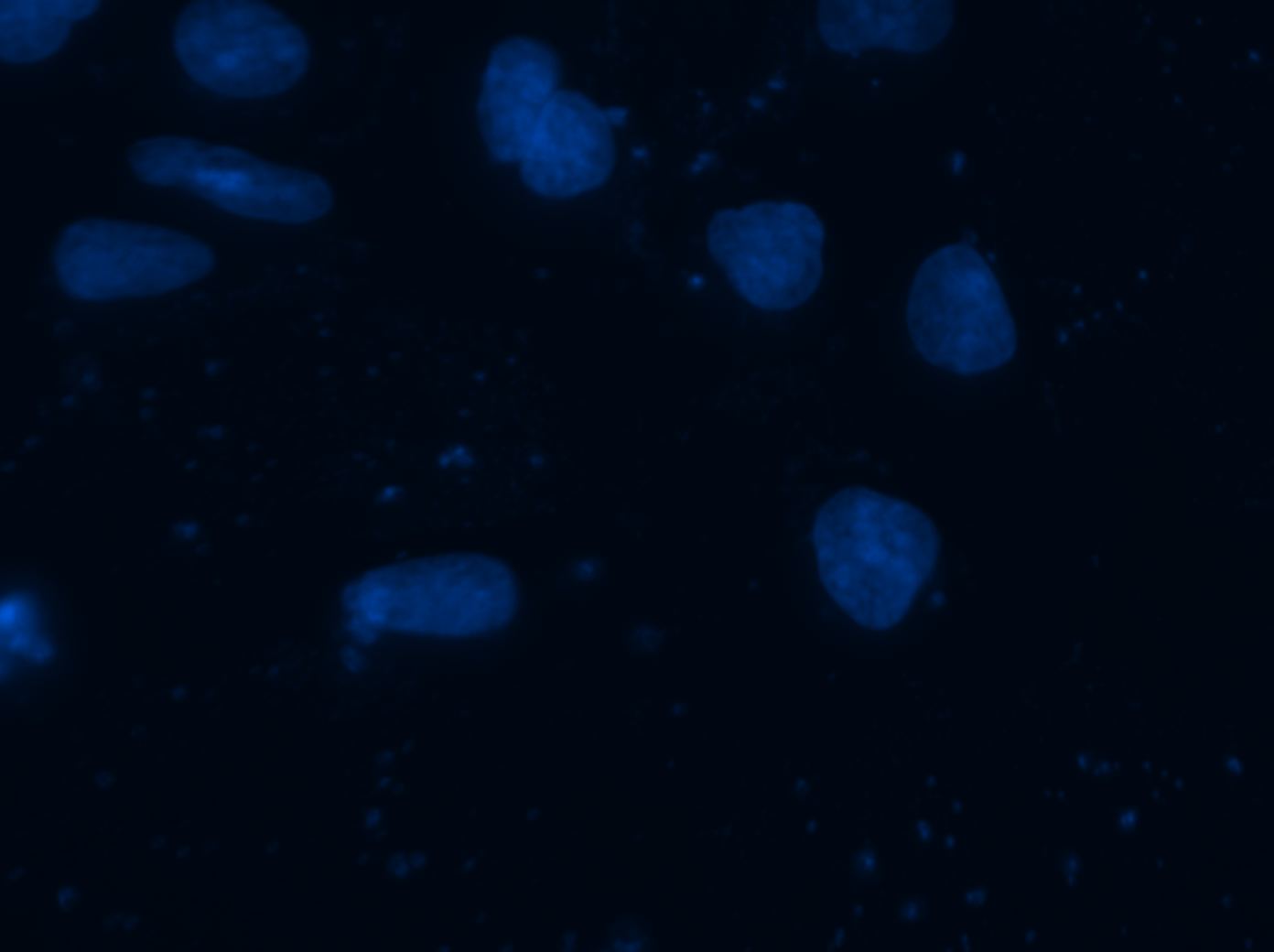

Supplement: Supplementary file 11 — Source data Fig. 7 [file 44318_2024_104_MOESM11_ESM.zip › Figure 7/7A/EGFP-LacI-Scc1+Myc-SA2-W334A DNA.tif]
