# Supplementary figures and images for: A non-canonical role of the inner kinetochore in regulating sister-chromatid cohesion at centromeres (part 3 of 3)
Source: EMBO J. 2024 May 7;43(12):7. doi: 10.1038/s44318-024-00104-6 (PMC11182772; doi:10.1038/s44318-024-00104-6)

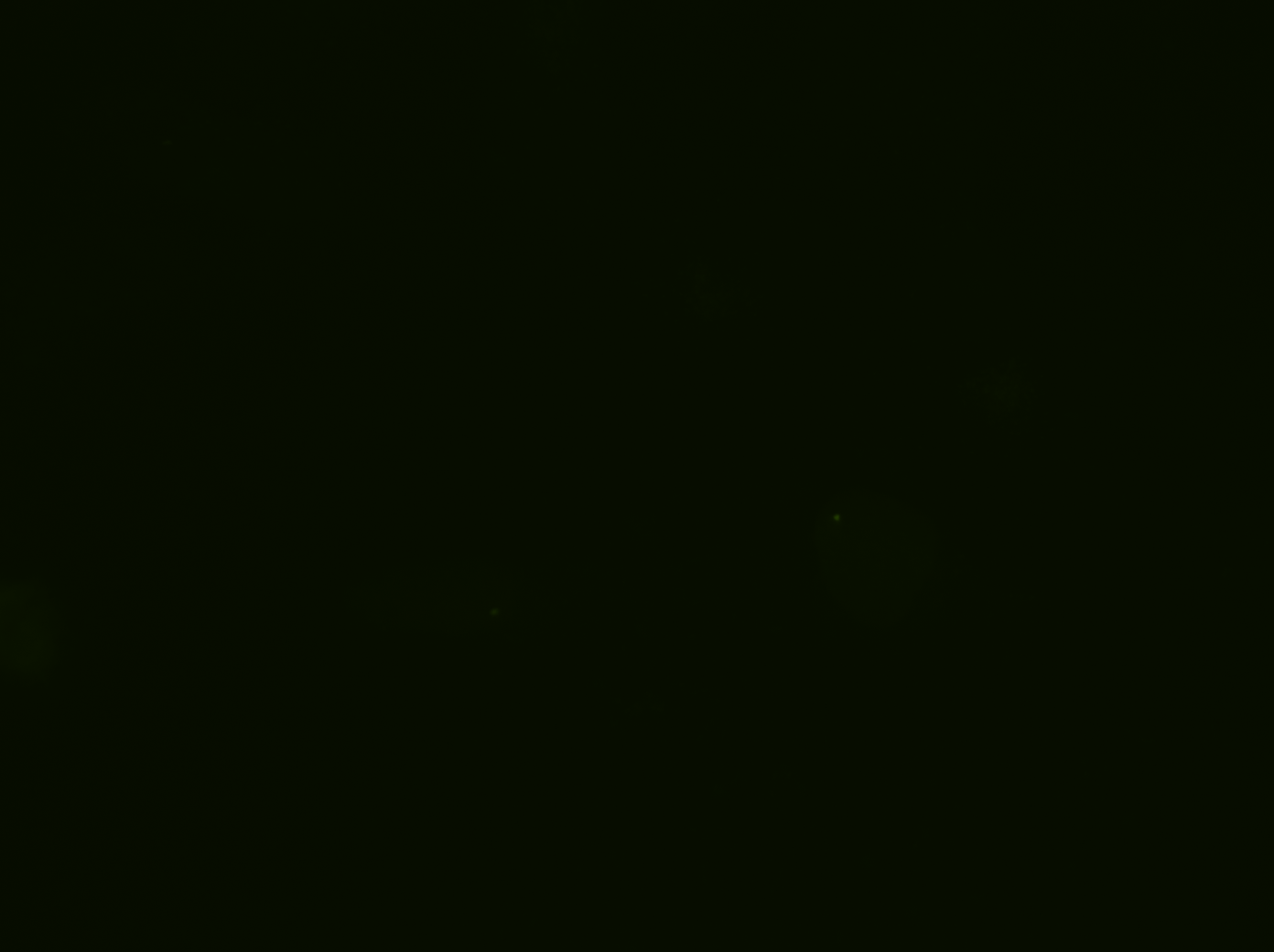

Supplement: Supplementary file 11 — Source data Fig. 7 [file 44318_2024_104_MOESM11_ESM.zip › Figure 7/7A/EGFP-LacI-Scc1+Myc-SA2-W334A EGFP.tif]

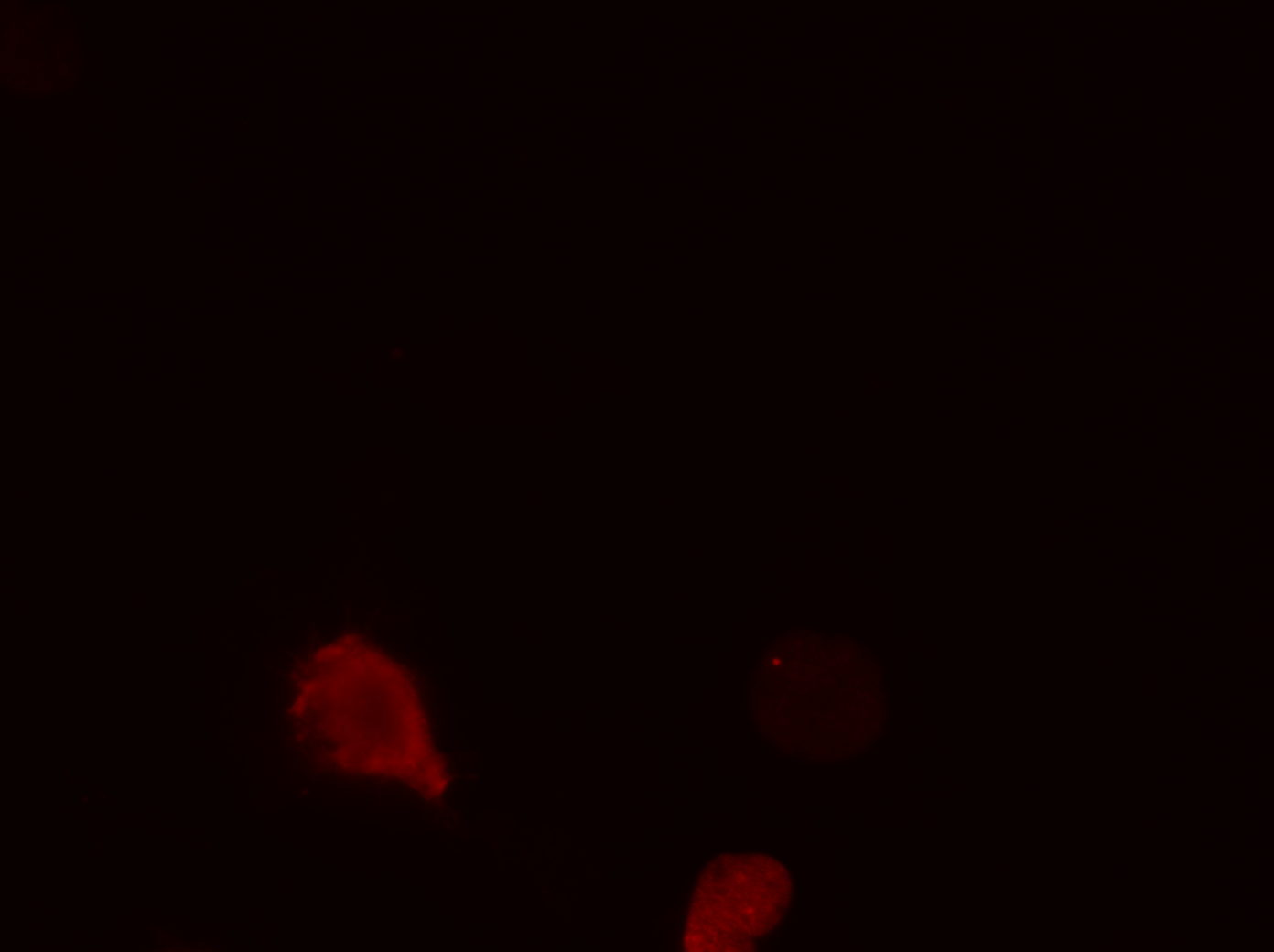

Supplement: Supplementary file 11 — Source data Fig. 7 [file 44318_2024_104_MOESM11_ESM.zip › Figure 7/7A/EGFP-LacI-Scc1-I337A+L341A+Myc-SA2 Anti-Myc.tif]

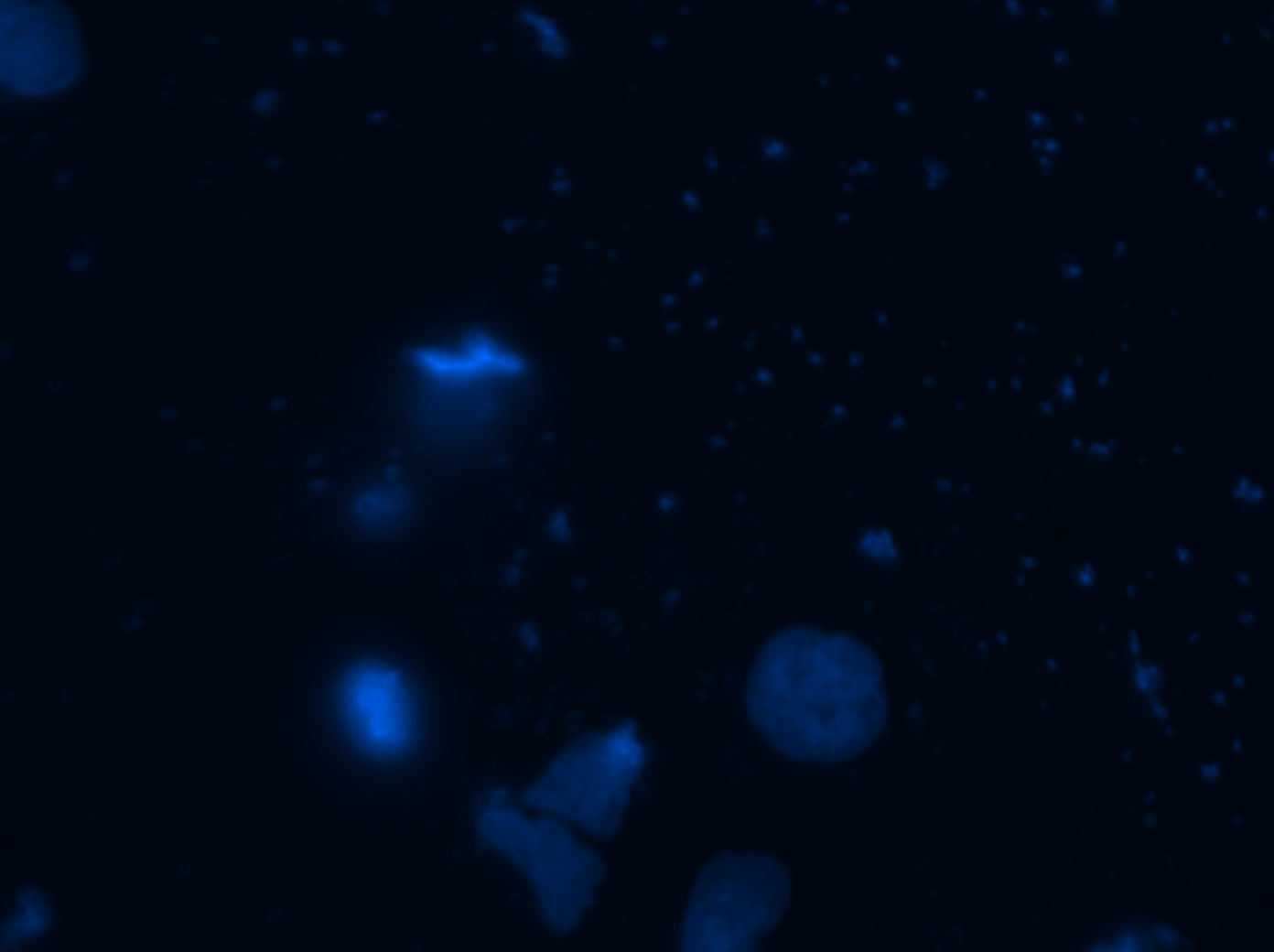

Supplement: Supplementary file 11 — Source data Fig. 7 [file 44318_2024_104_MOESM11_ESM.zip › Figure 7/7A/EGFP-LacI-Scc1-I337A+L341A+Myc-SA2 DNA.tif]

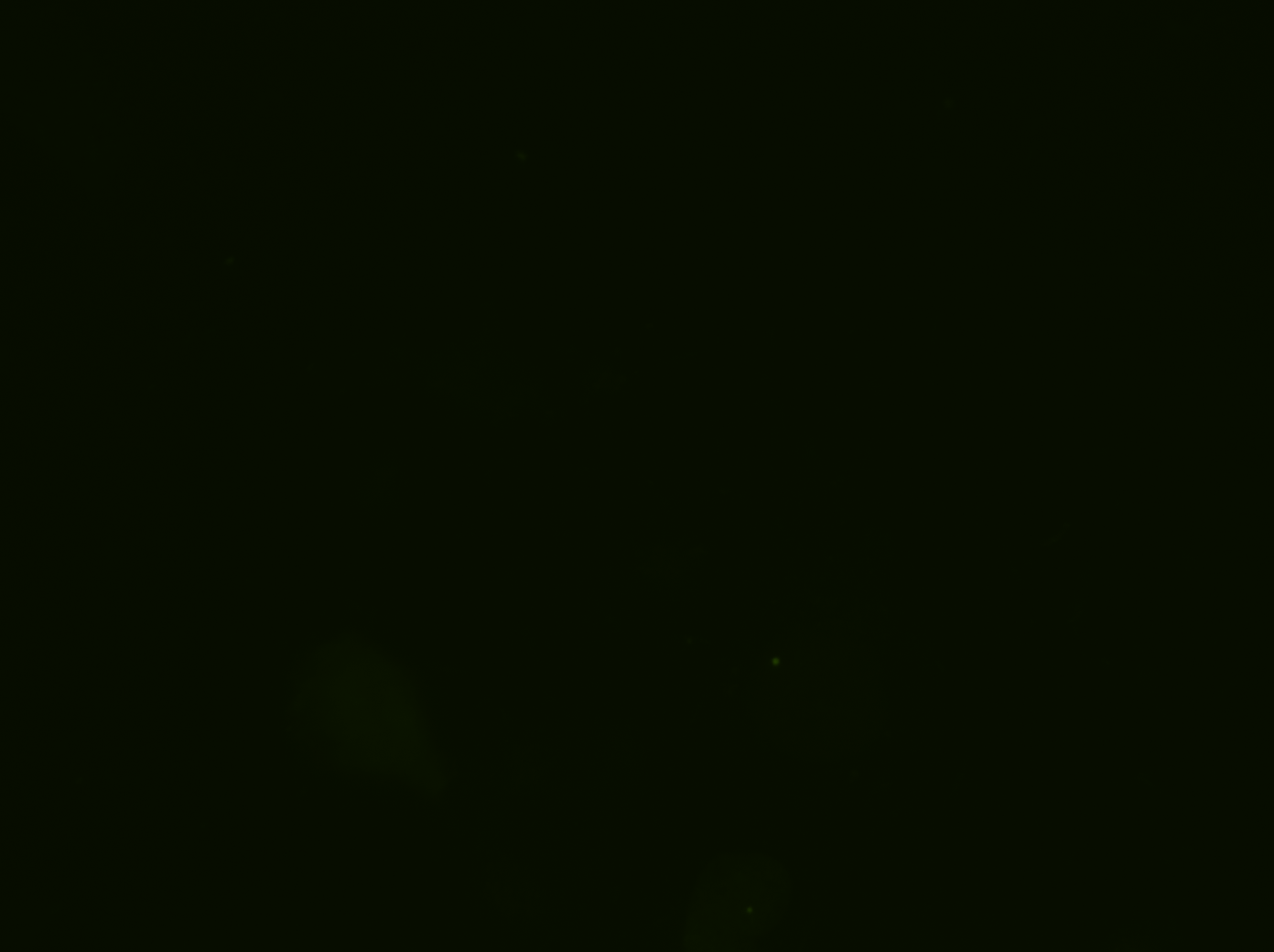

Supplement: Supplementary file 11 — Source data Fig. 7 [file 44318_2024_104_MOESM11_ESM.zip › Figure 7/7A/EGFP-LacI-Scc1-I337A+L341A+Myc-SA2 EGFP.tif]

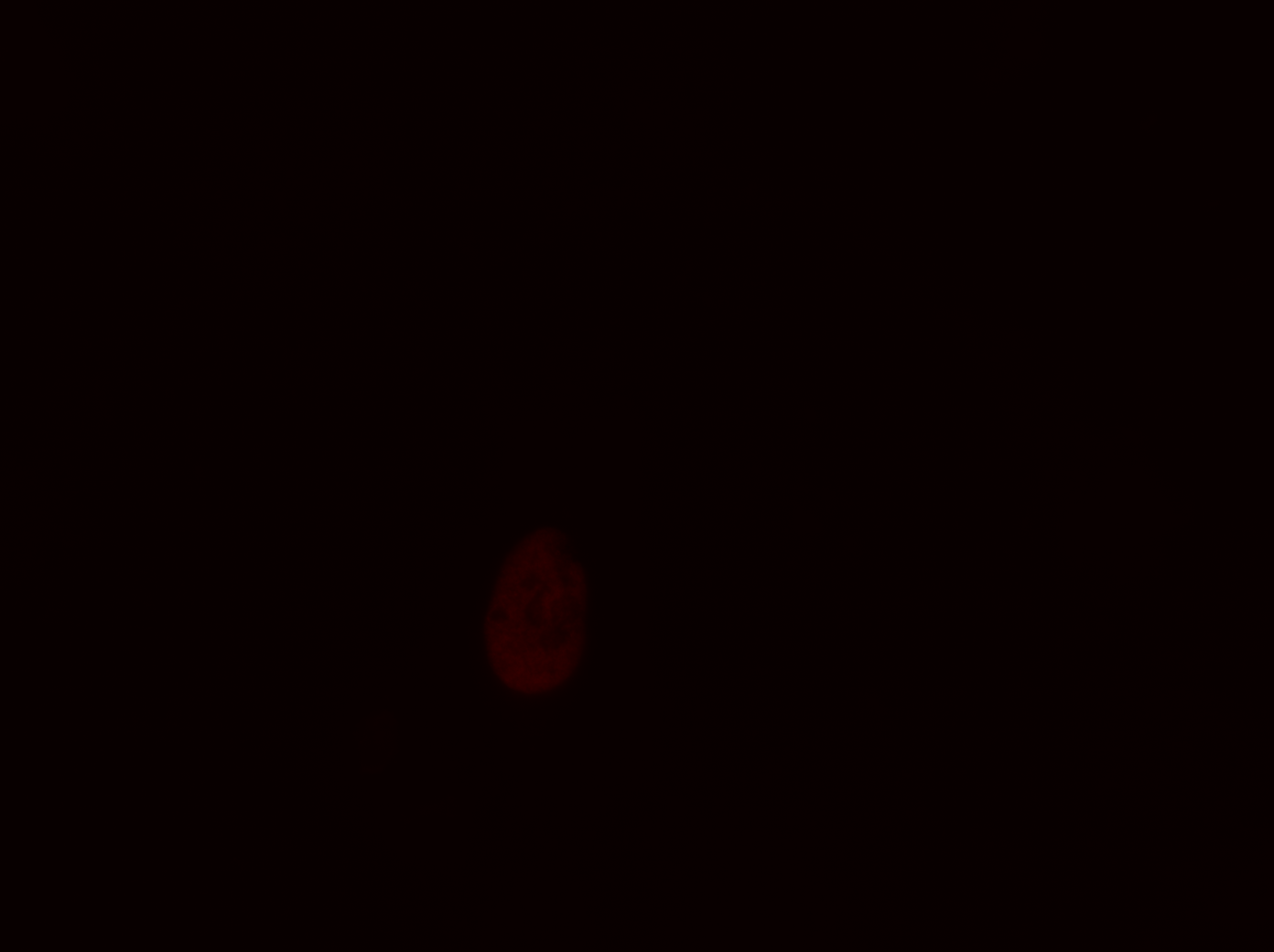

Supplement: Supplementary file 11 — Source data Fig. 7 [file 44318_2024_104_MOESM11_ESM.zip › Figure 7/7B/EGFP-LacI+Myc-SA2 Anti-Myc.tif]

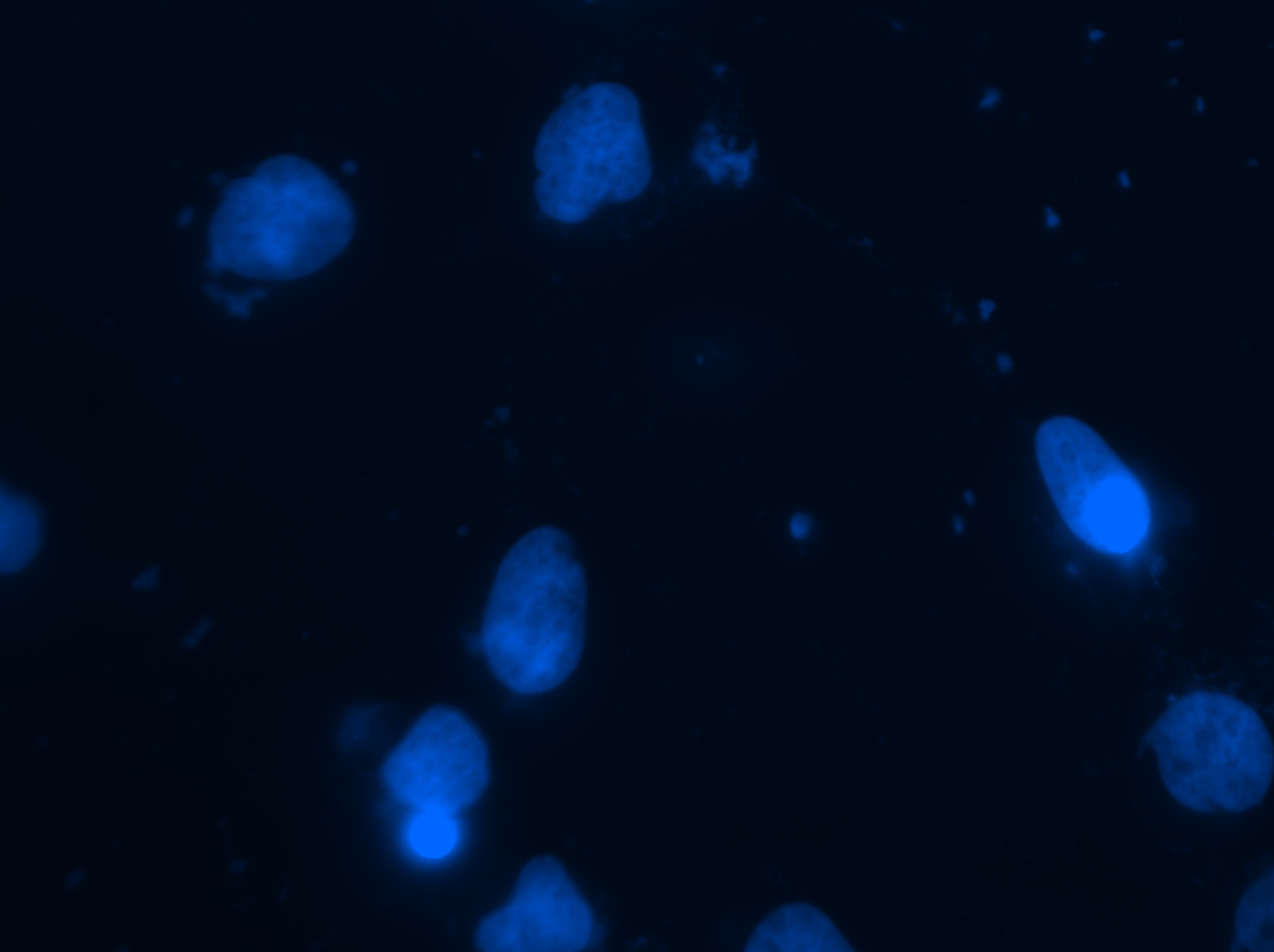

Supplement: Supplementary file 11 — Source data Fig. 7 [file 44318_2024_104_MOESM11_ESM.zip › Figure 7/7B/EGFP-LacI+Myc-SA2 DNA.tif]

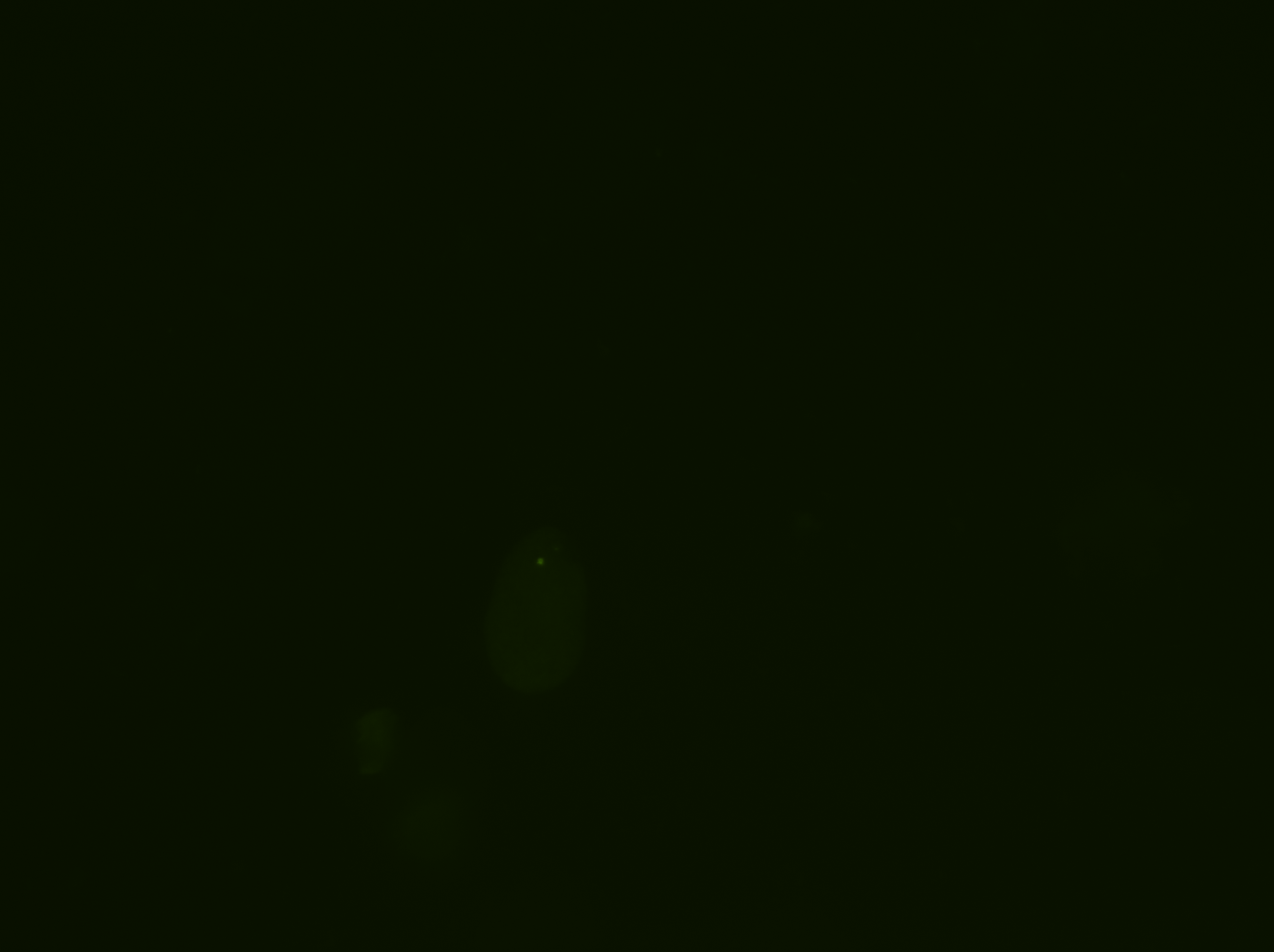

Supplement: Supplementary file 11 — Source data Fig. 7 [file 44318_2024_104_MOESM11_ESM.zip › Figure 7/7B/EGFP-lacI+Myc-SA2 EGFP.tif]

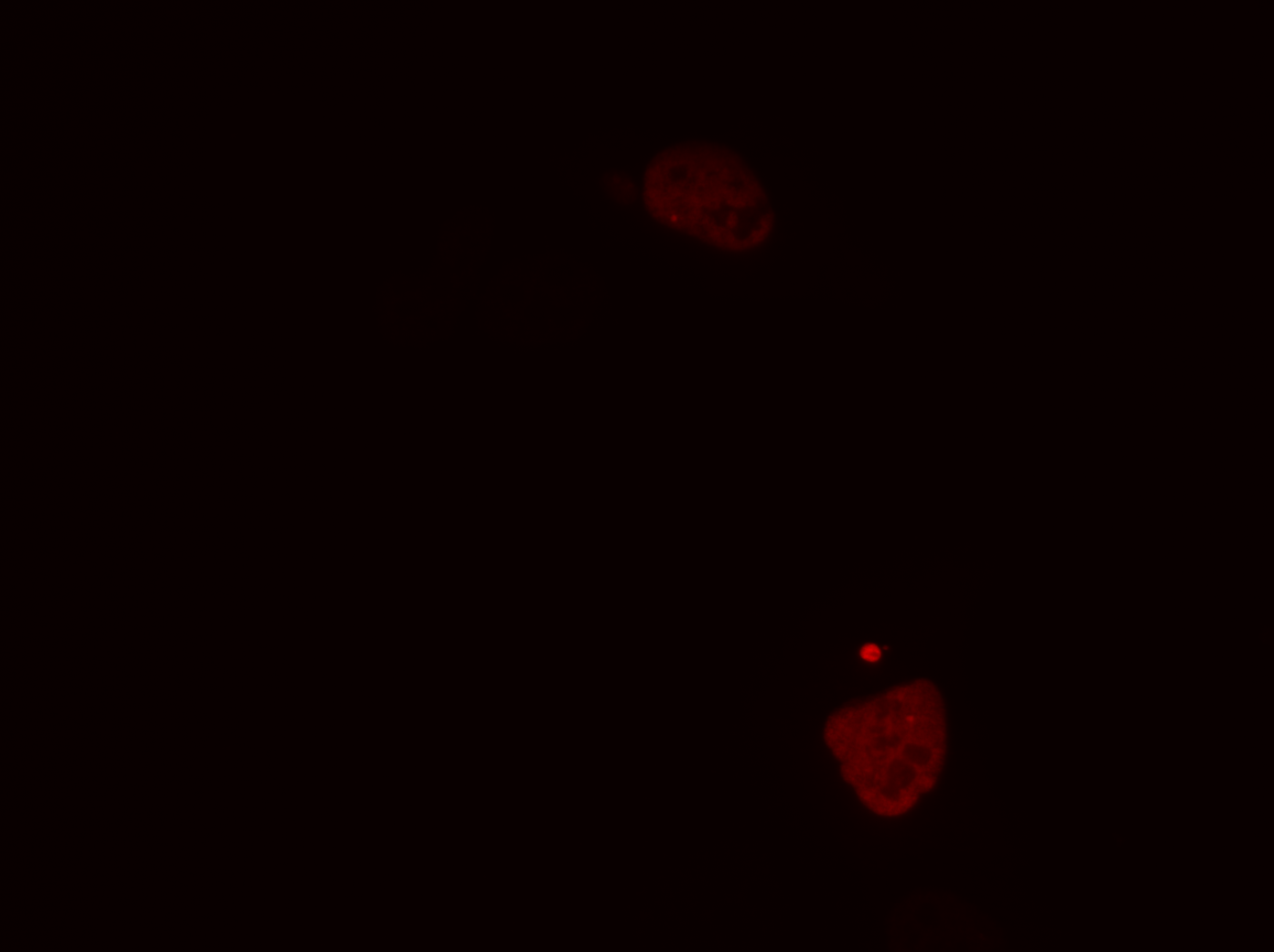

Supplement: Supplementary file 11 — Source data Fig. 7 [file 44318_2024_104_MOESM11_ESM.zip › Figure 7/7B/EGFP-LacI-Scc1+Myc-SA2 Anti-Myc.tif]

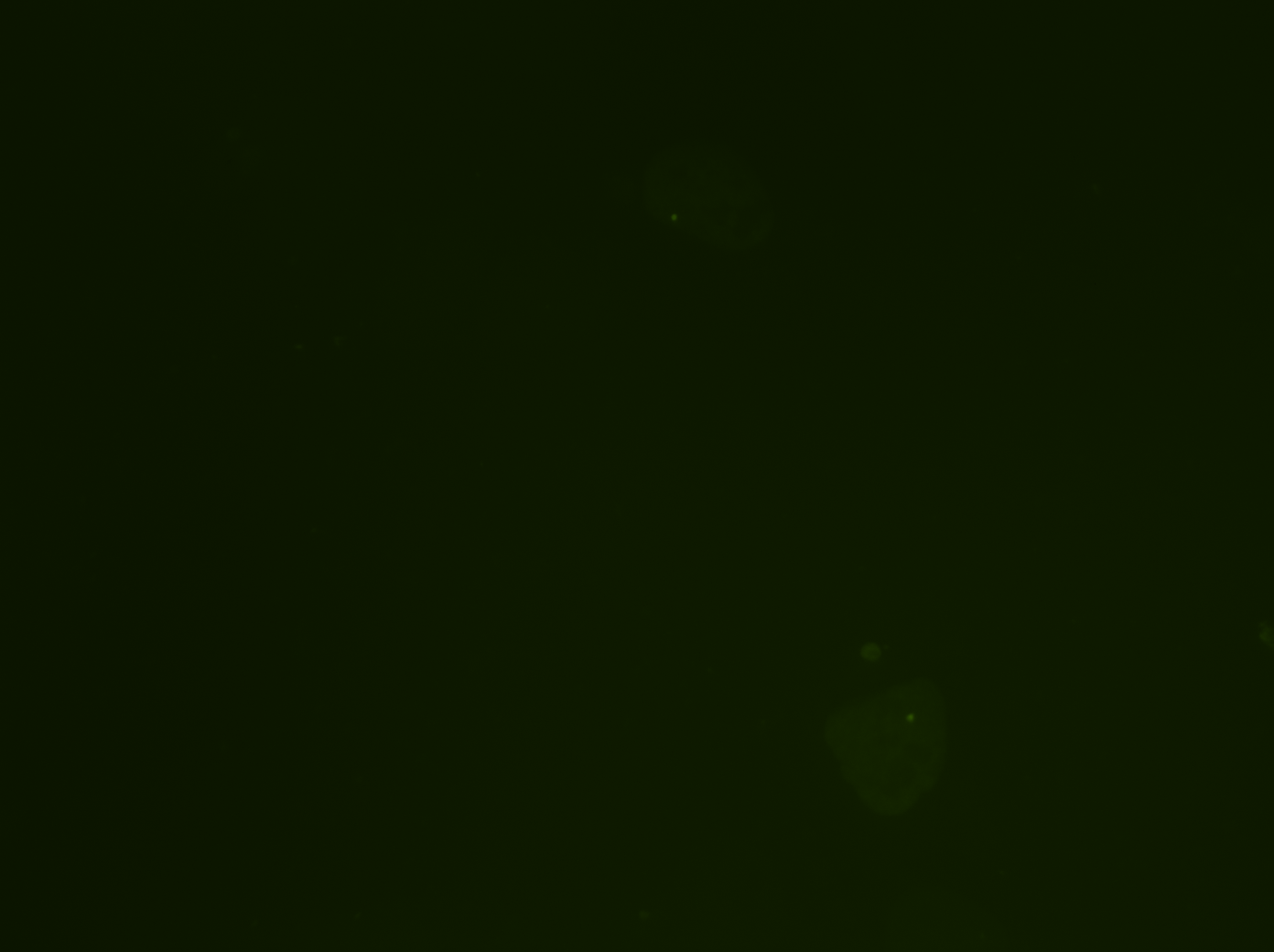

Supplement: Supplementary file 11 — Source data Fig. 7 [file 44318_2024_104_MOESM11_ESM.zip › Figure 7/7B/EGFP-LacI-Scc1+Myc-SA2 DNA EGFP.tif]

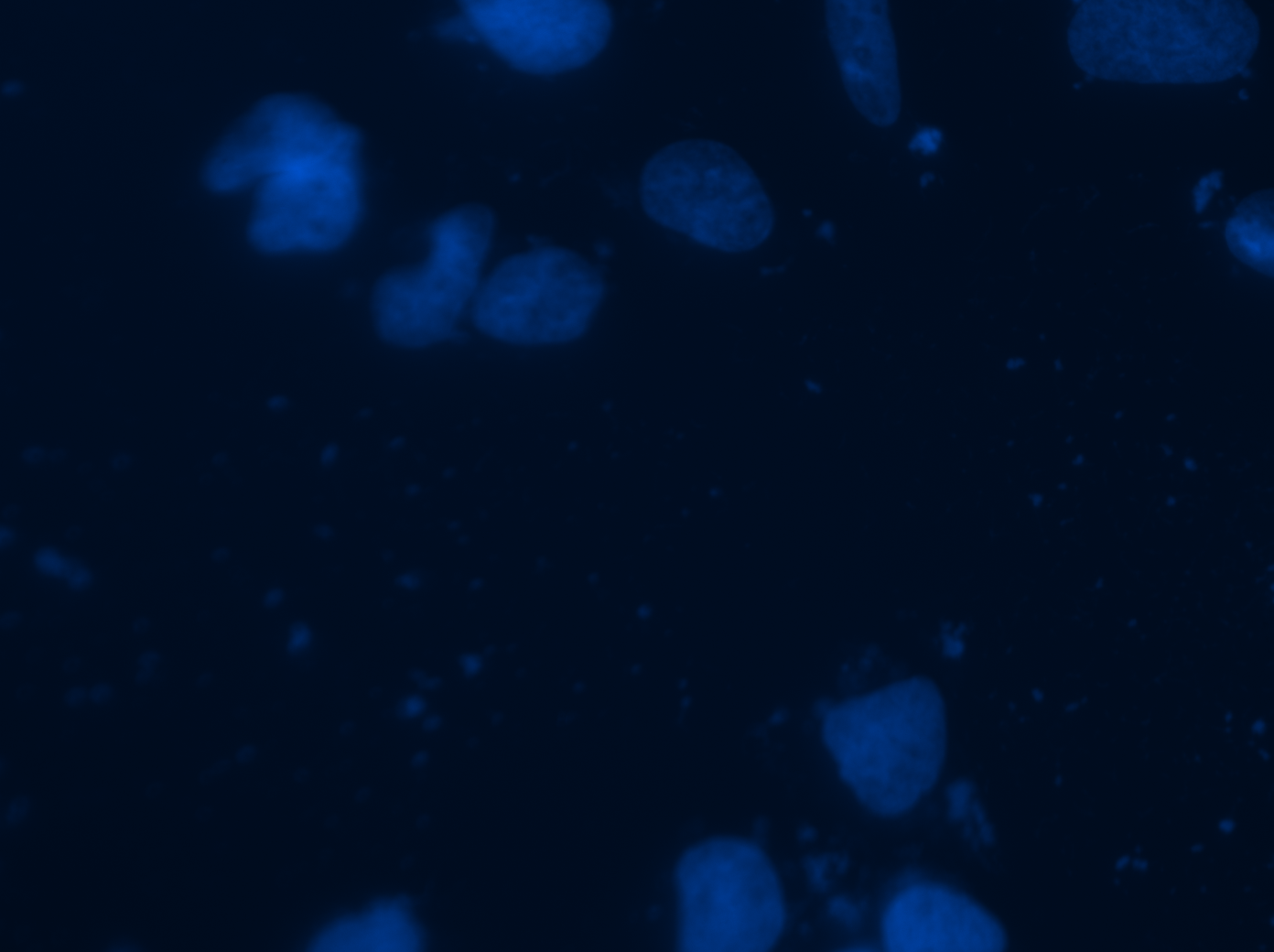

Supplement: Supplementary file 11 — Source data Fig. 7 [file 44318_2024_104_MOESM11_ESM.zip › Figure 7/7B/EGFP-LacI-Scc1+Myc-SA2 DNA.tif]

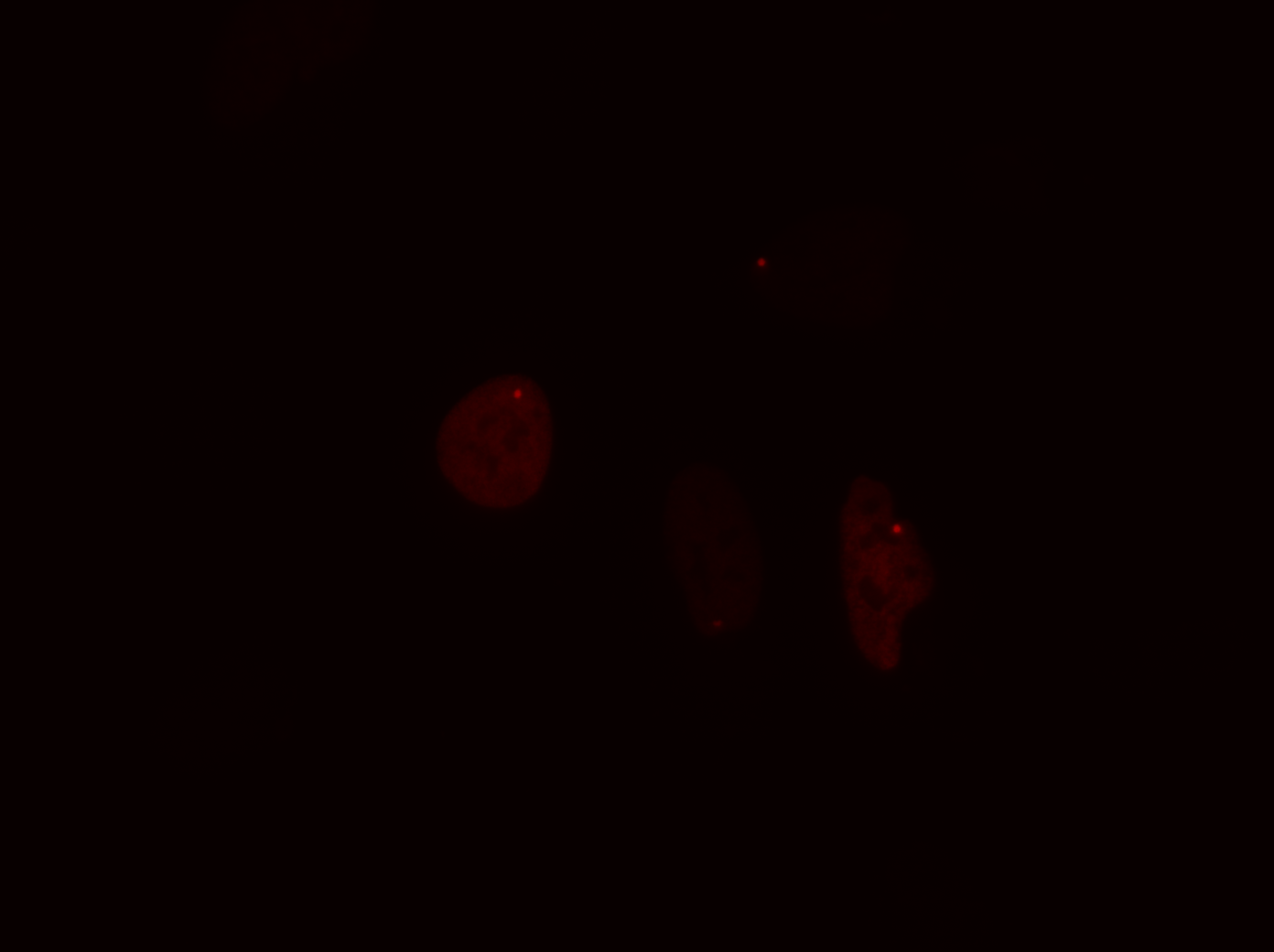

Supplement: Supplementary file 11 — Source data Fig. 7 [file 44318_2024_104_MOESM11_ESM.zip › Figure 7/7B/EGFP-LacI-Scc1+Myc-SA2-F367A Anti-Myc.tif]

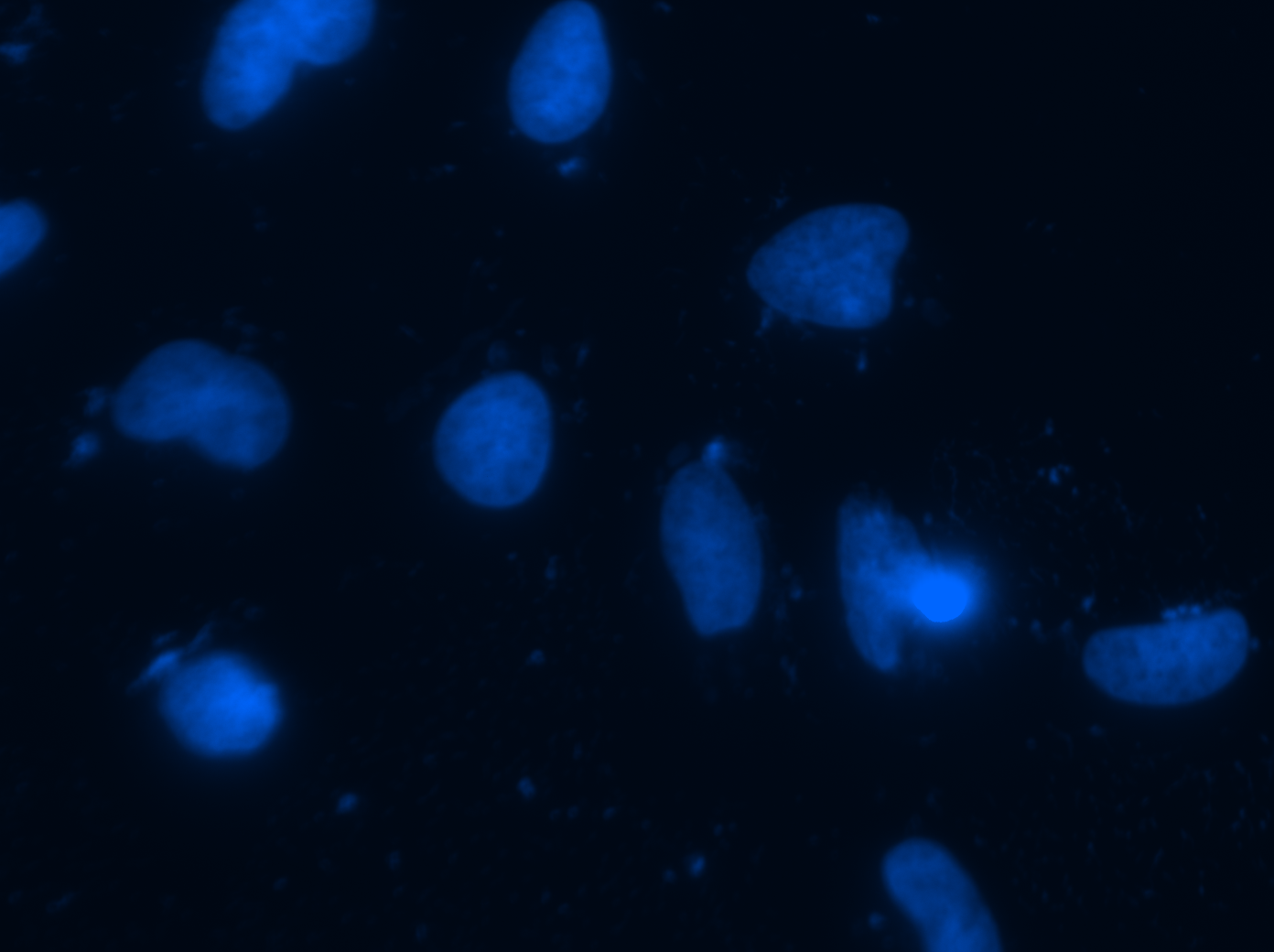

Supplement: Supplementary file 11 — Source data Fig. 7 [file 44318_2024_104_MOESM11_ESM.zip › Figure 7/7B/EGFP-LacI-Scc1+Myc-SA2-F367A DNA.tif]

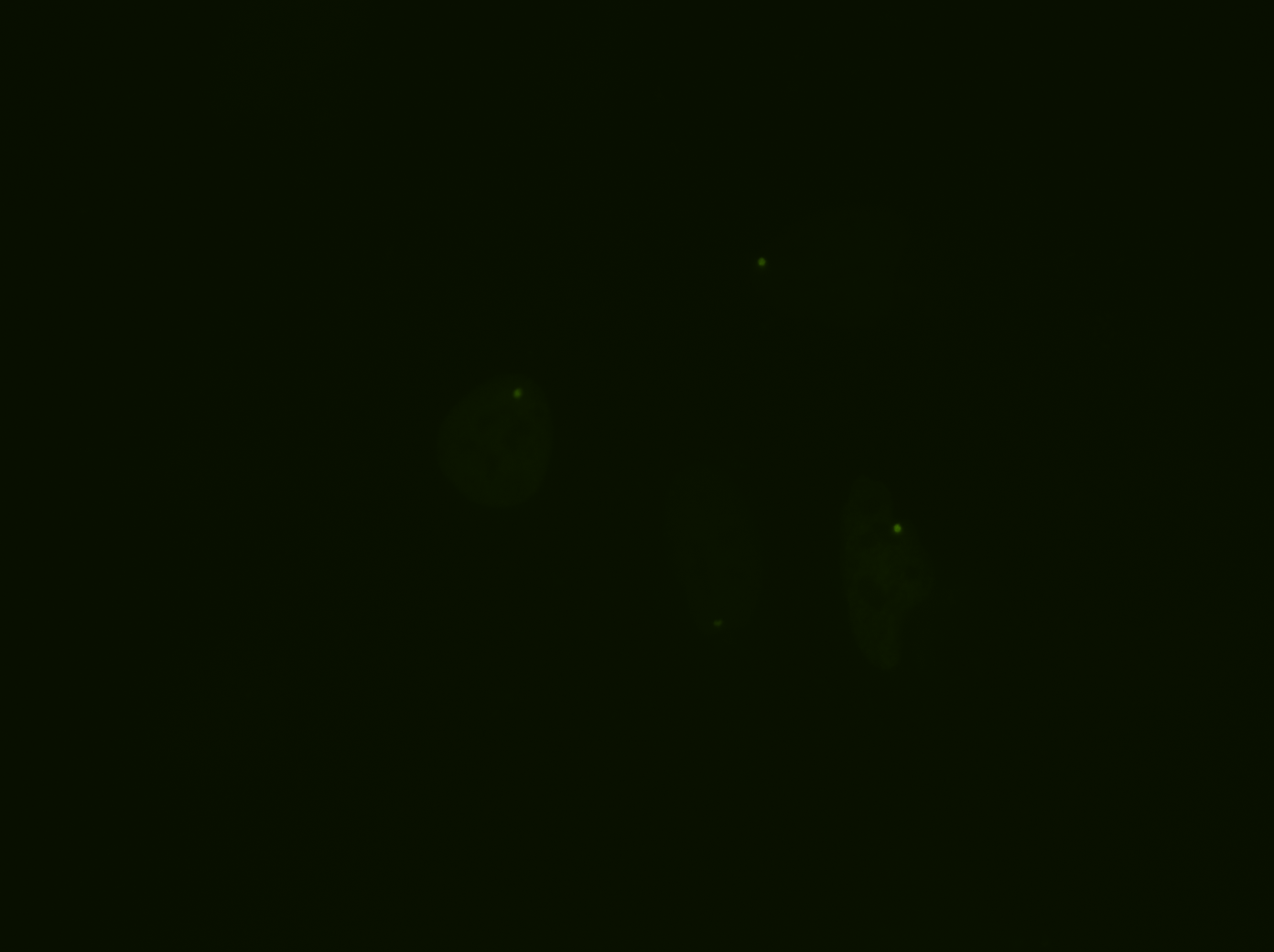

Supplement: Supplementary file 11 — Source data Fig. 7 [file 44318_2024_104_MOESM11_ESM.zip › Figure 7/7B/EGFP-LacI-Scc1+Myc-SA2-F367A EGFP.tif]

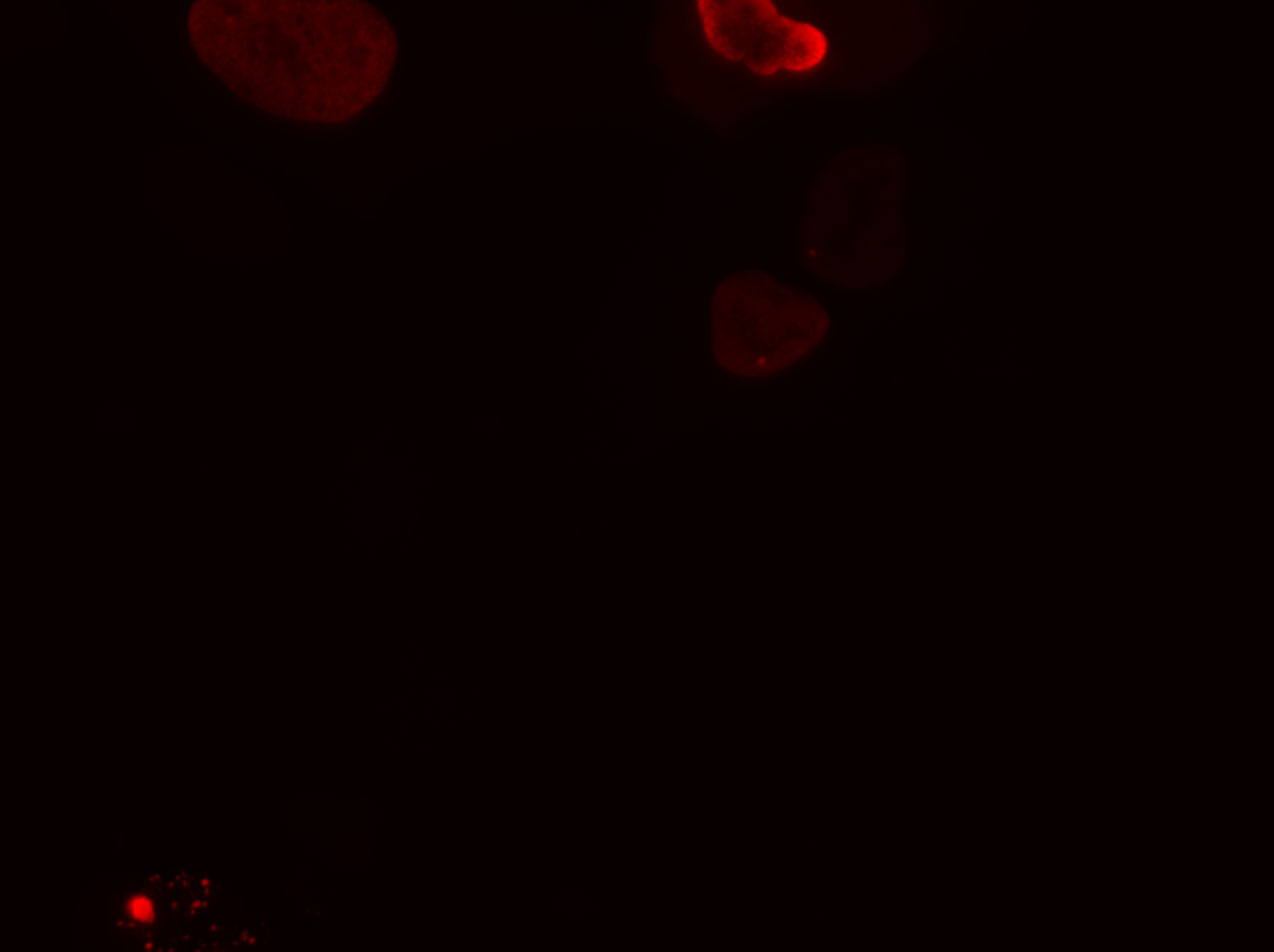

Supplement: Supplementary file 11 — Source data Fig. 7 [file 44318_2024_104_MOESM11_ESM.zip › Figure 7/7B/EGFP-LacI-Scc1+Myc-SA2-F371A Anti-Myc.tif]

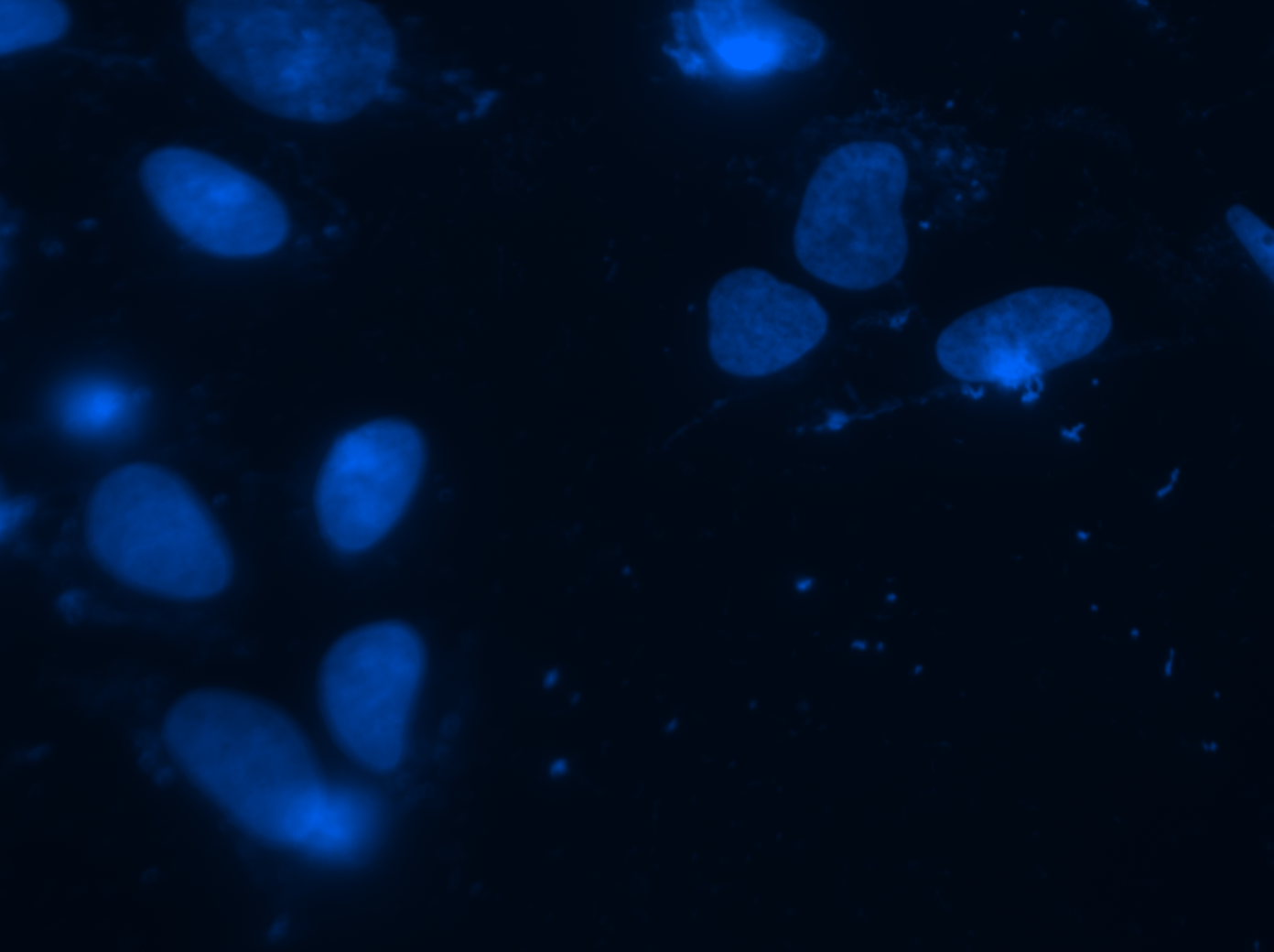

Supplement: Supplementary file 11 — Source data Fig. 7 [file 44318_2024_104_MOESM11_ESM.zip › Figure 7/7B/EGFP-LacI-Scc1+Myc-SA2-F371A DNA.tif]

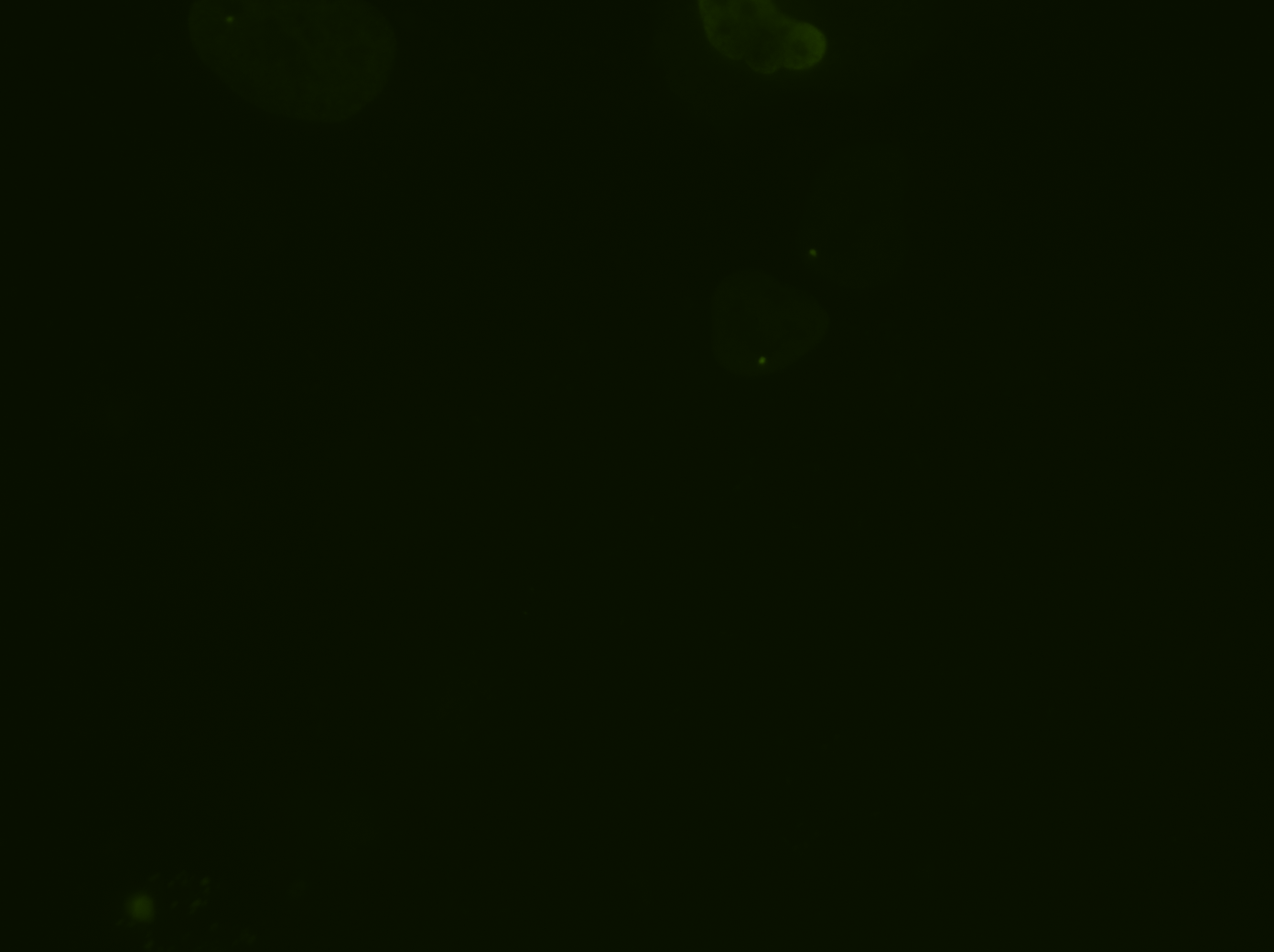

Supplement: Supplementary file 11 — Source data Fig. 7 [file 44318_2024_104_MOESM11_ESM.zip › Figure 7/7B/EGFP-LacI-Scc1+Myc-SA2-F371A EGFP.tif]

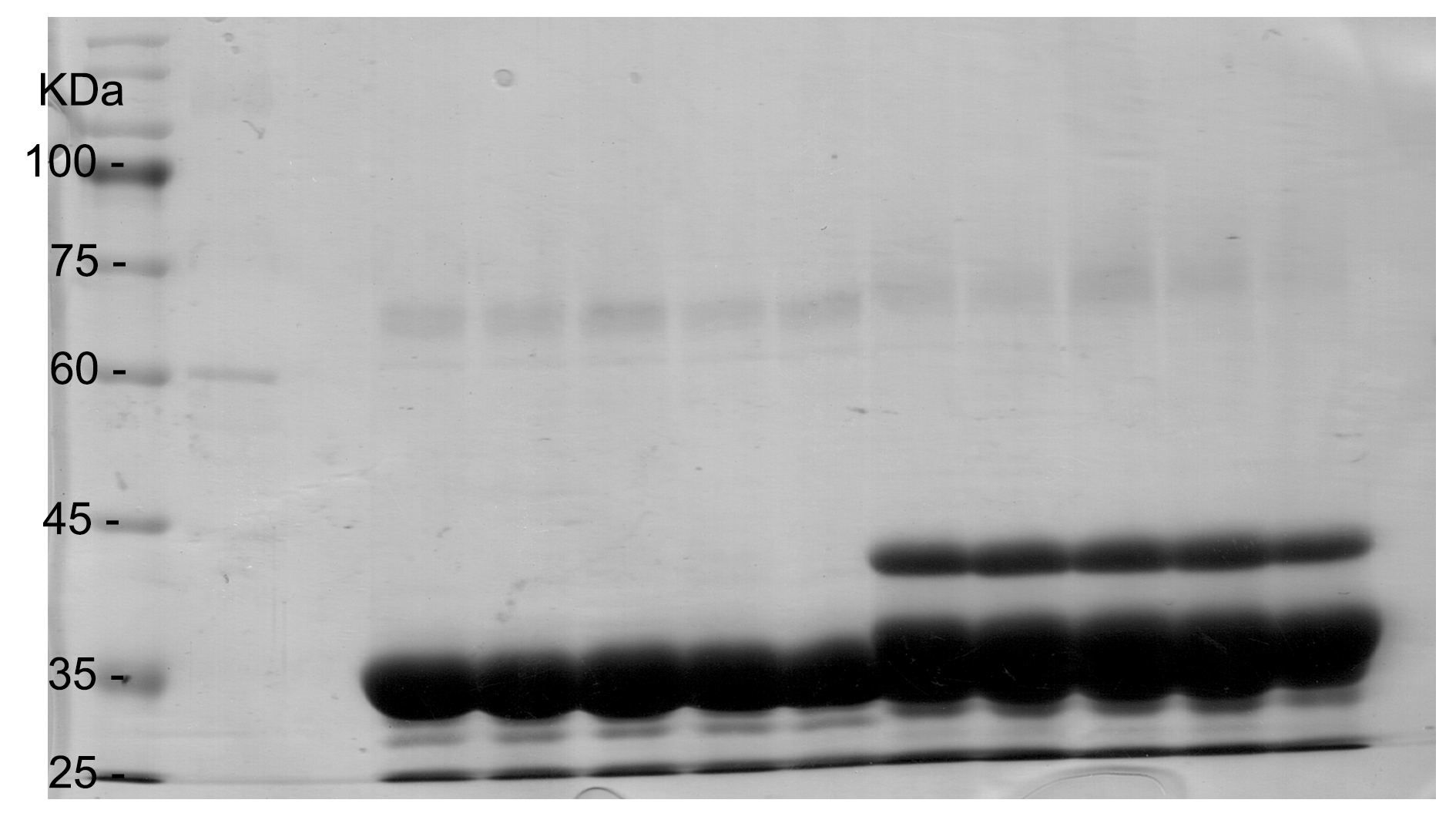

Supplement: Supplementary file 11 — Source data Fig. 7 [file 44318_2024_104_MOESM11_ESM.zip › Figure 7/7C/CBB.tif]

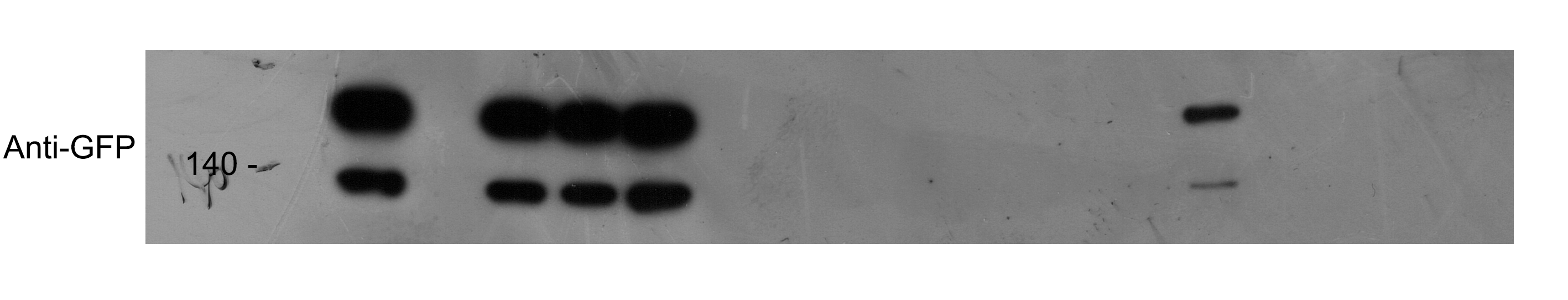

Supplement: Supplementary file 11 — Source data Fig. 7 [file 44318_2024_104_MOESM11_ESM.zip › Figure 7/7C/western GFP.tif]

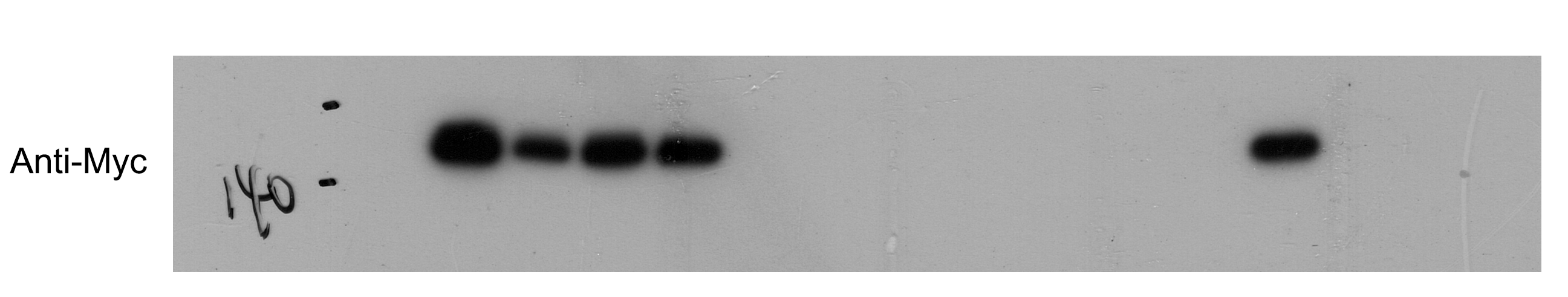

Supplement: Supplementary file 11 — Source data Fig. 7 [file 44318_2024_104_MOESM11_ESM.zip › Figure 7/7C/western Myc.tif]

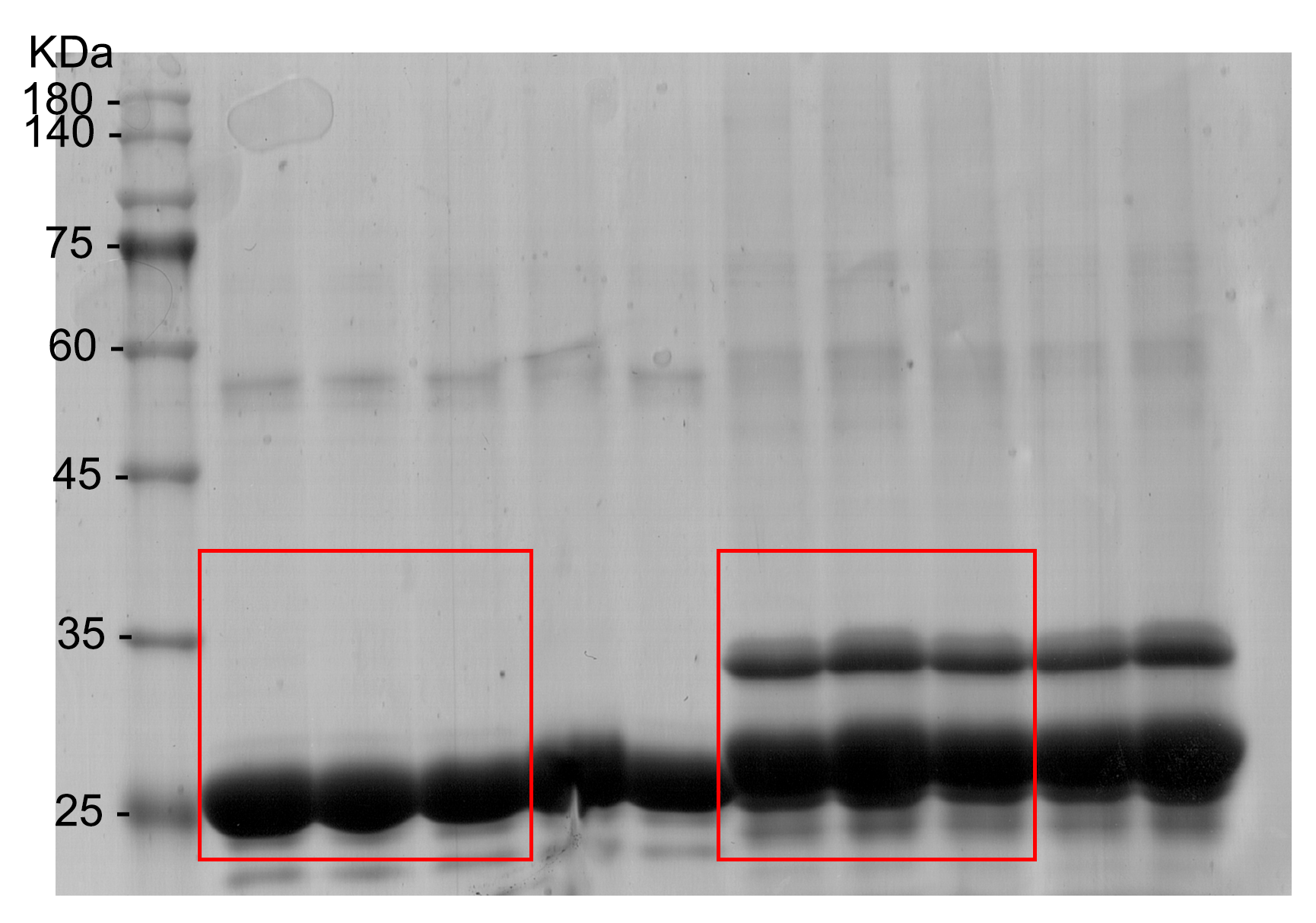

Supplement: Supplementary file 11 — Source data Fig. 7 [file 44318_2024_104_MOESM11_ESM.zip › Figure 7/7D/CBB.tif]

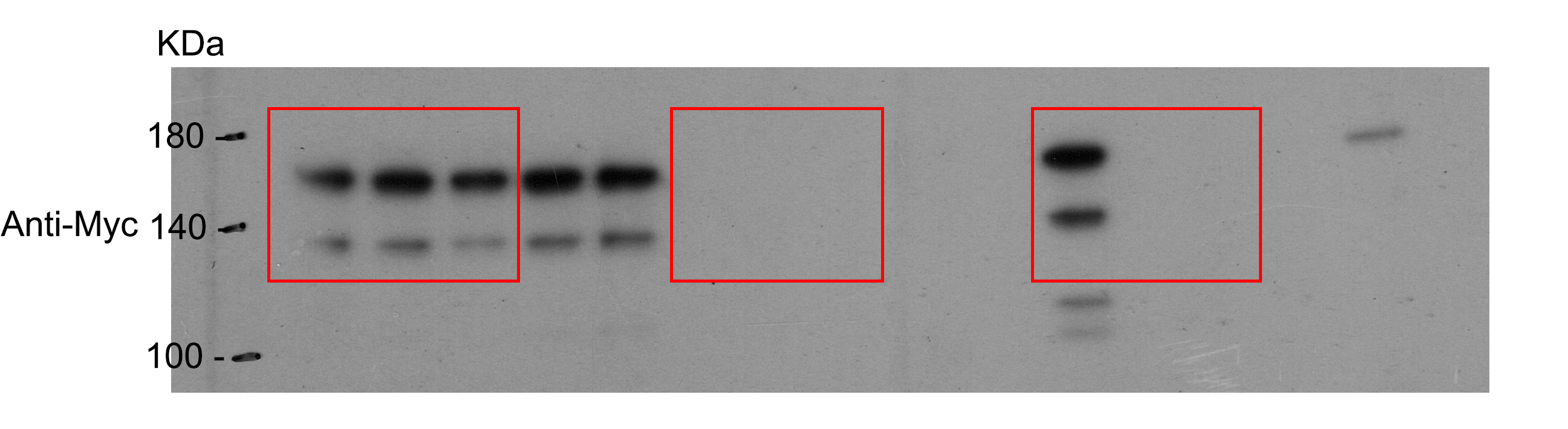

Supplement: Supplementary file 11 — Source data Fig. 7 [file 44318_2024_104_MOESM11_ESM.zip › Figure 7/7D/western GFP.tif]

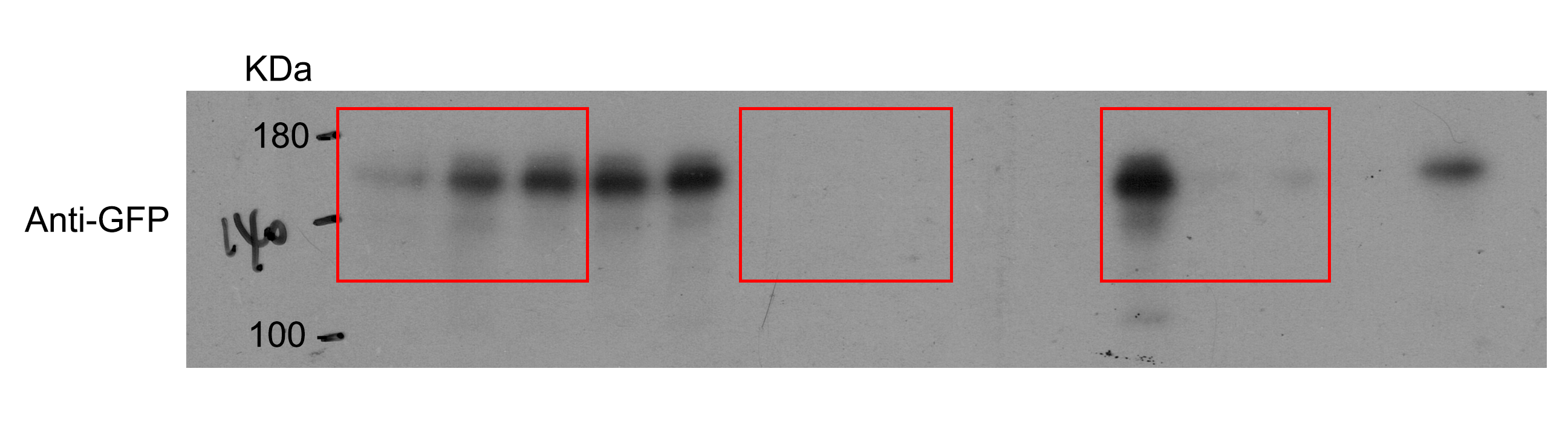

Supplement: Supplementary file 11 — Source data Fig. 7 [file 44318_2024_104_MOESM11_ESM.zip › Figure 7/7D/western Myc.tif]

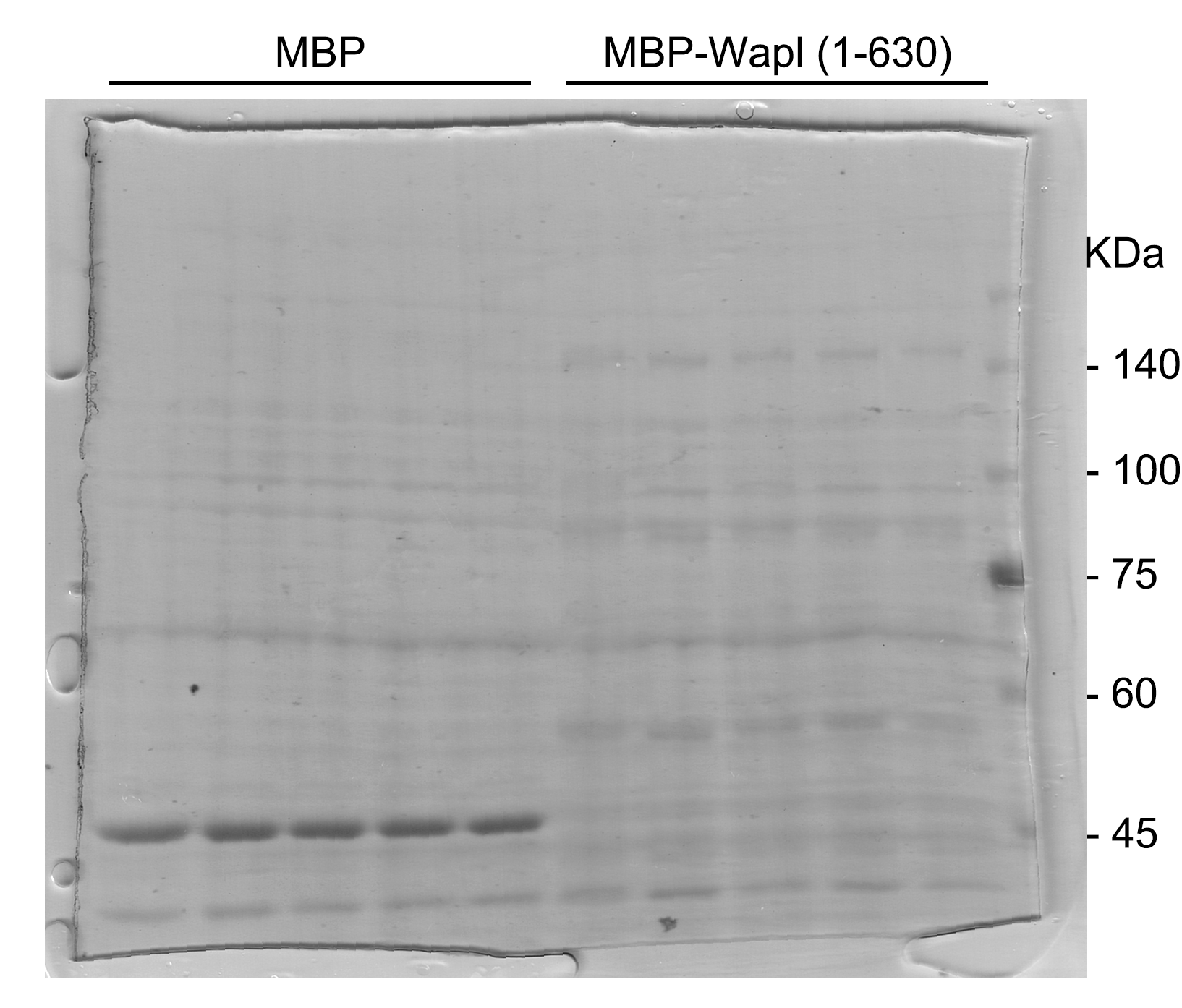

Supplement: Supplementary file 11 — Source data Fig. 7 [file 44318_2024_104_MOESM11_ESM.zip › Figure 7/7E/7E CBB.tif]

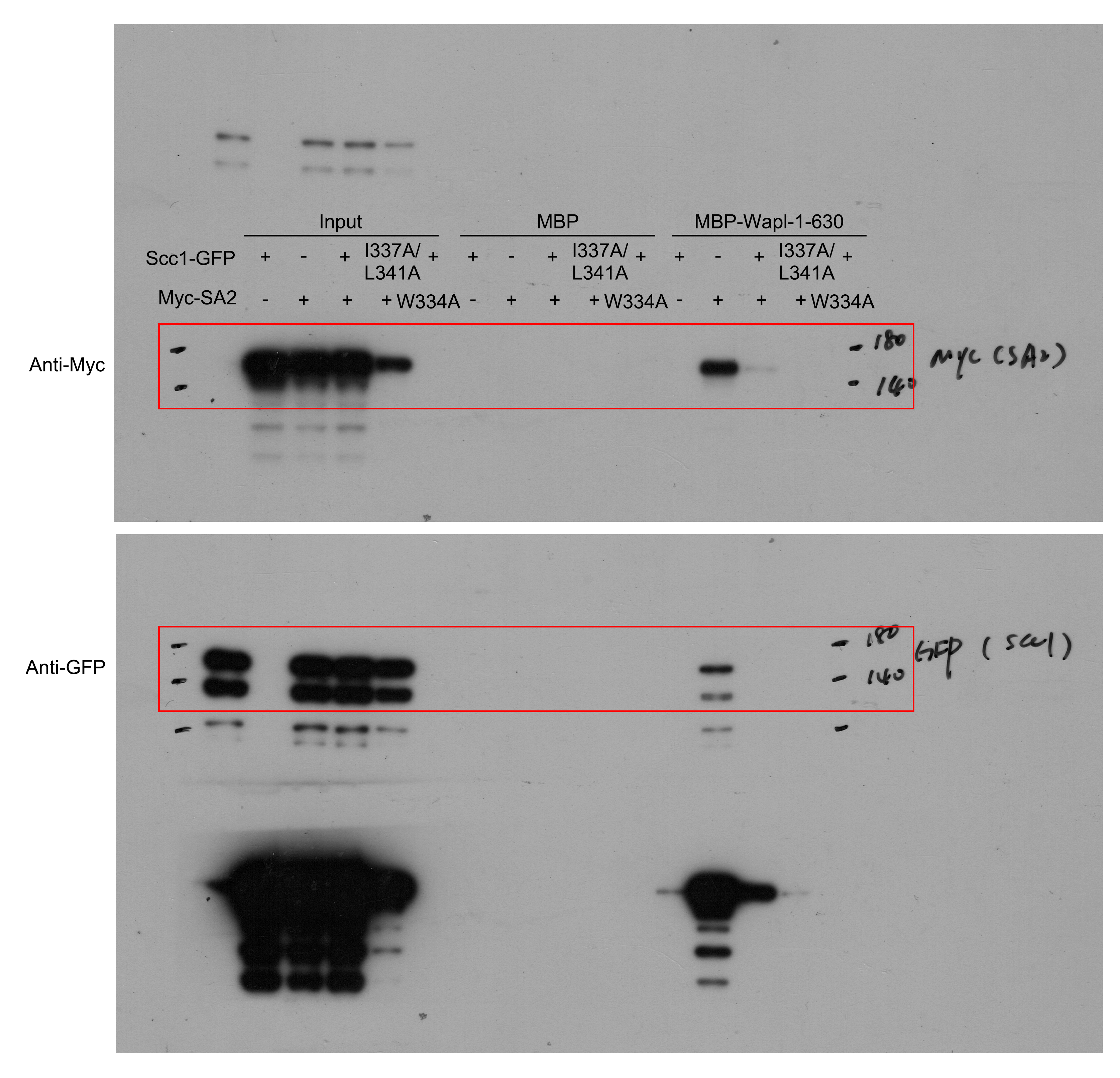

Supplement: Supplementary file 11 — Source data Fig. 7 [file 44318_2024_104_MOESM11_ESM.zip › Figure 7/7E/7E WB.tif]

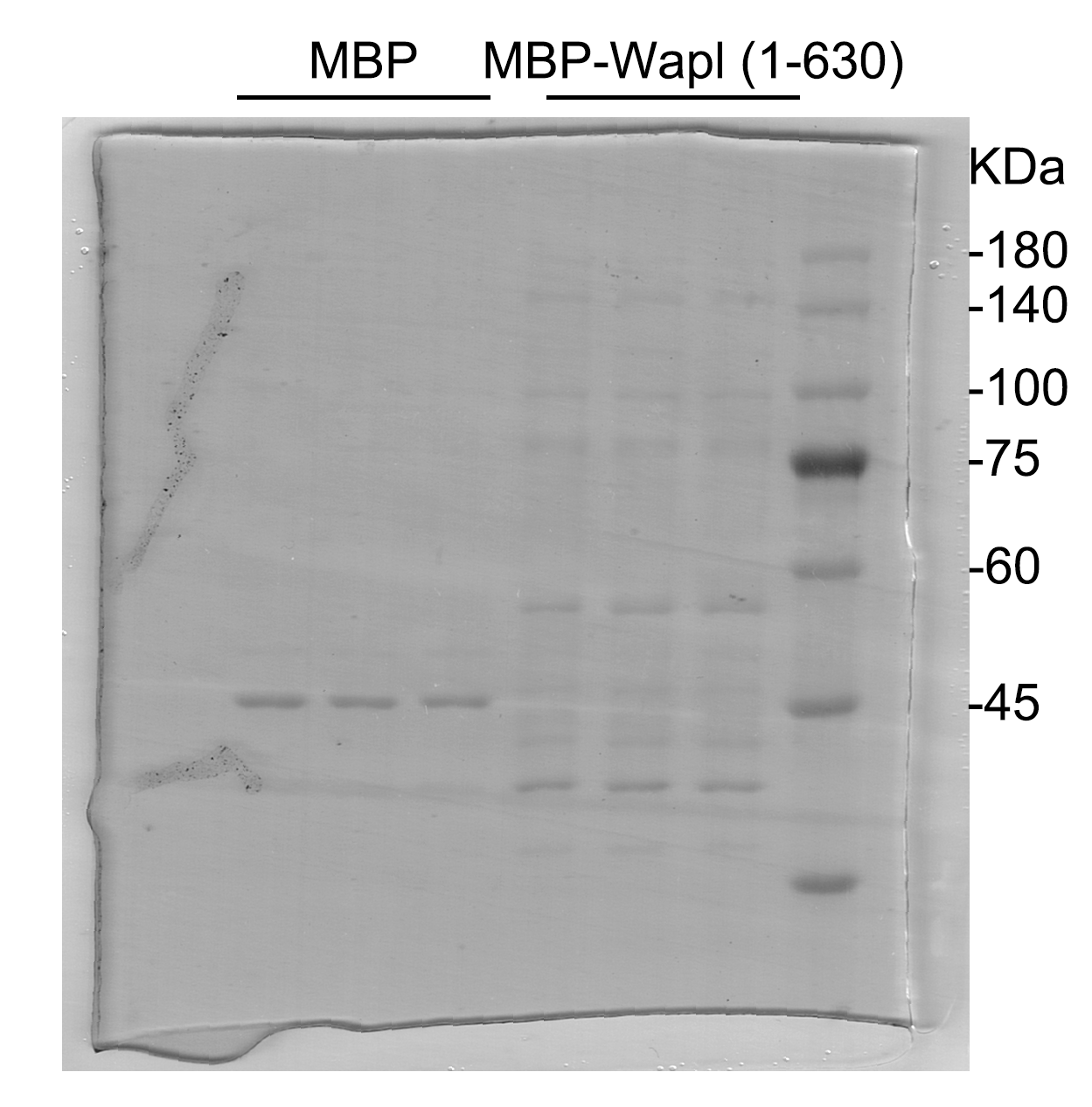

Supplement: Supplementary file 11 — Source data Fig. 7 [file 44318_2024_104_MOESM11_ESM.zip › Figure 7/7F/7F CBB.tif]

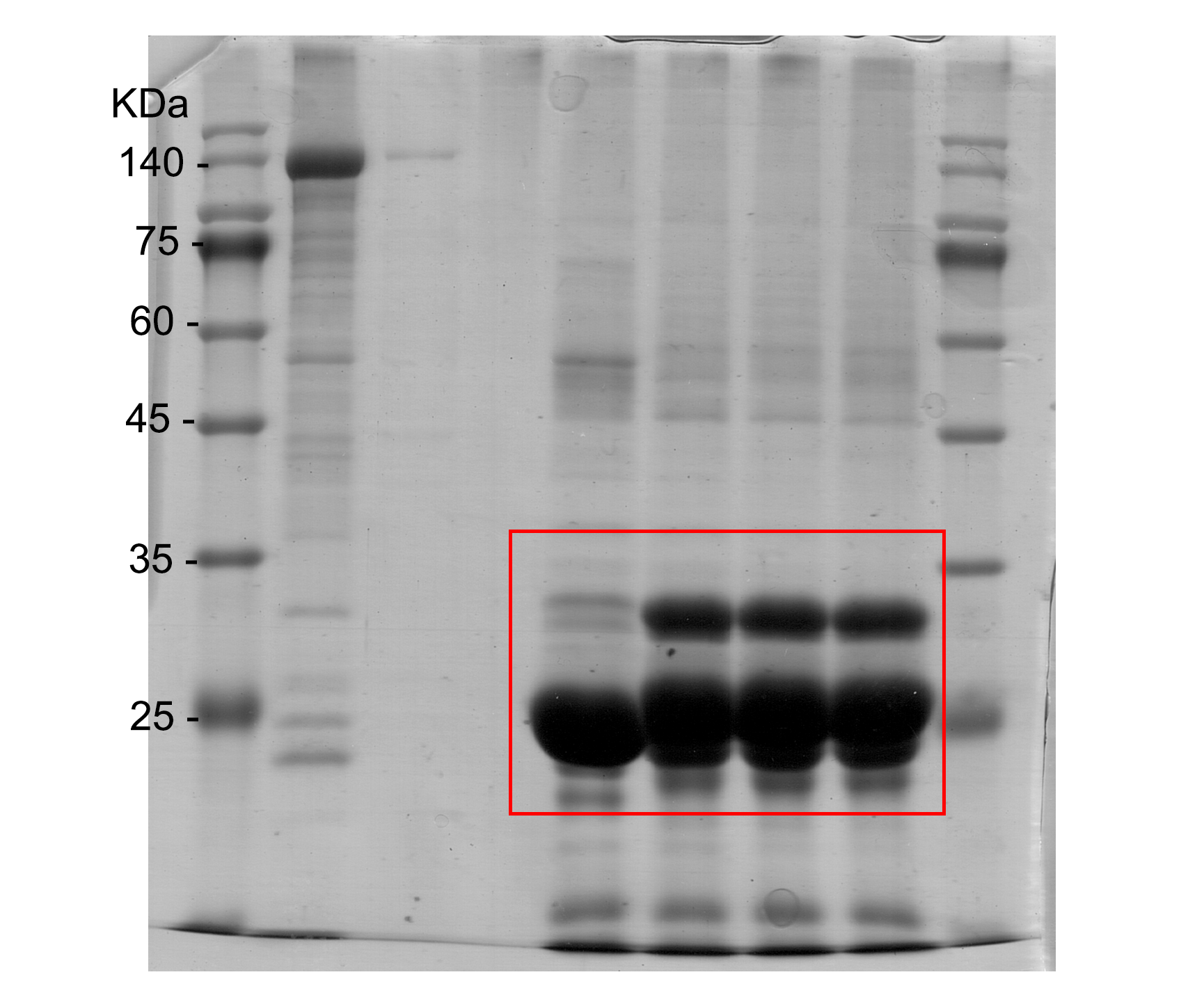

Supplement: Supplementary file 11 — Source data Fig. 7 [file 44318_2024_104_MOESM11_ESM.zip › Figure 7/7G/CBB.tif]

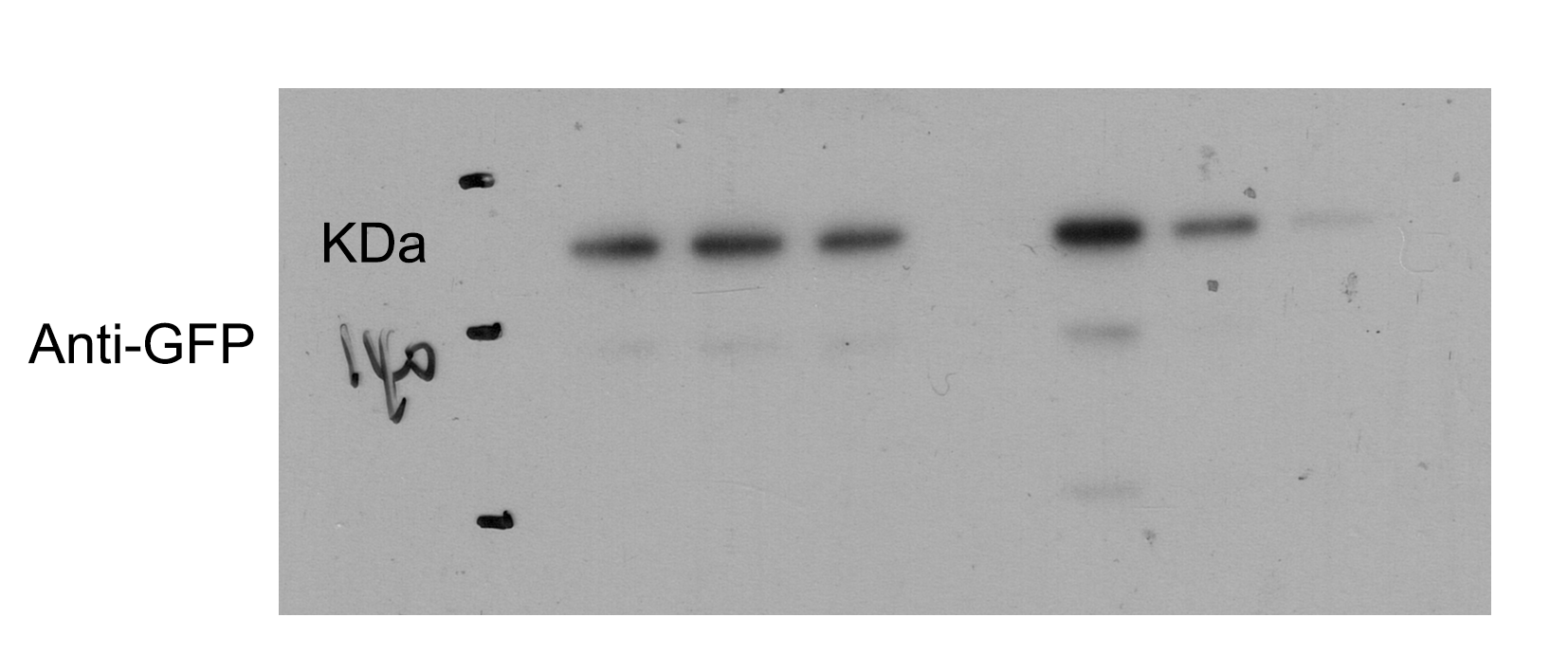

Supplement: Supplementary file 11 — Source data Fig. 7 [file 44318_2024_104_MOESM11_ESM.zip › Figure 7/7G/western GFP.tif]

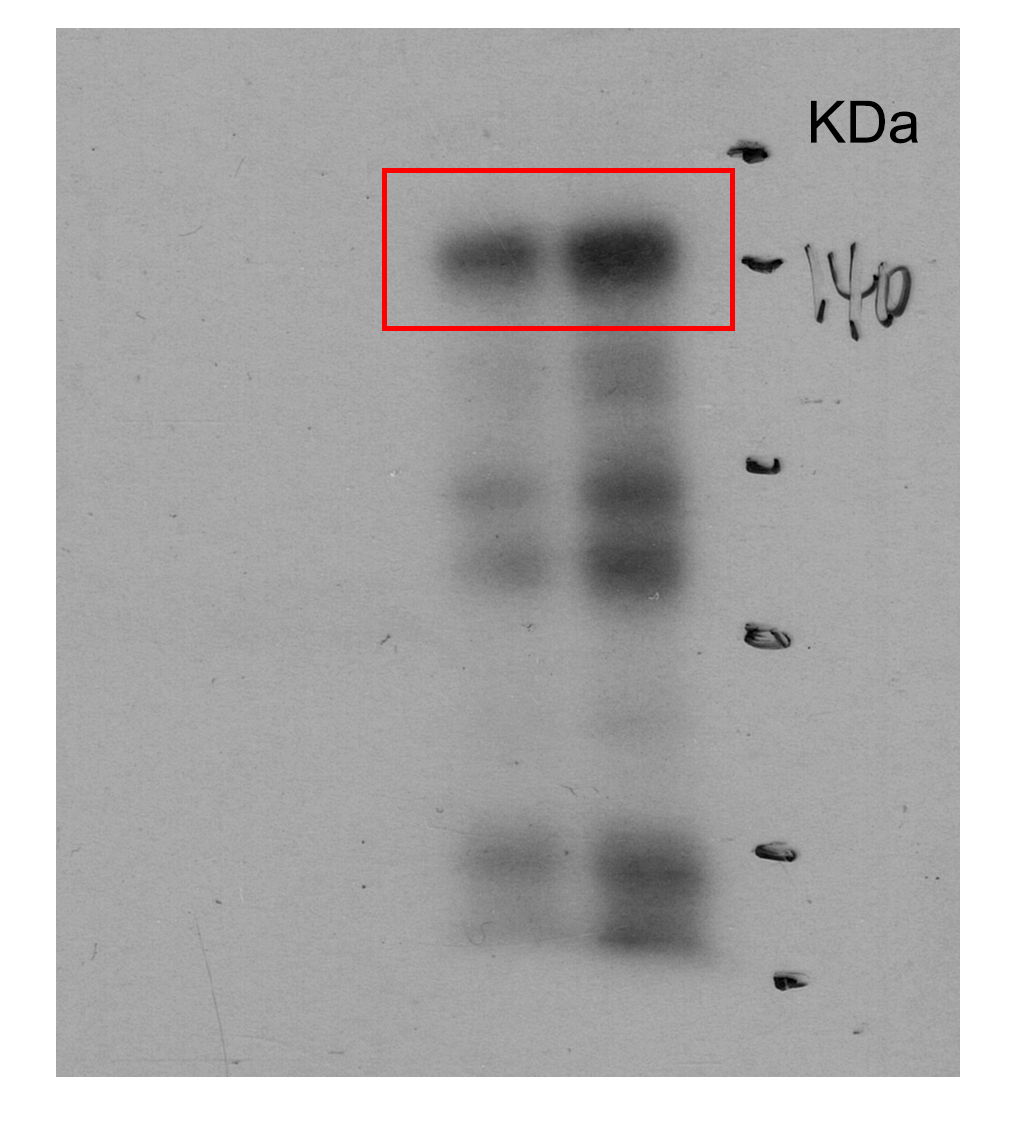

Supplement: Supplementary file 11 — Source data Fig. 7 [file 44318_2024_104_MOESM11_ESM.zip › Figure 7/7G/western MBP.tif]

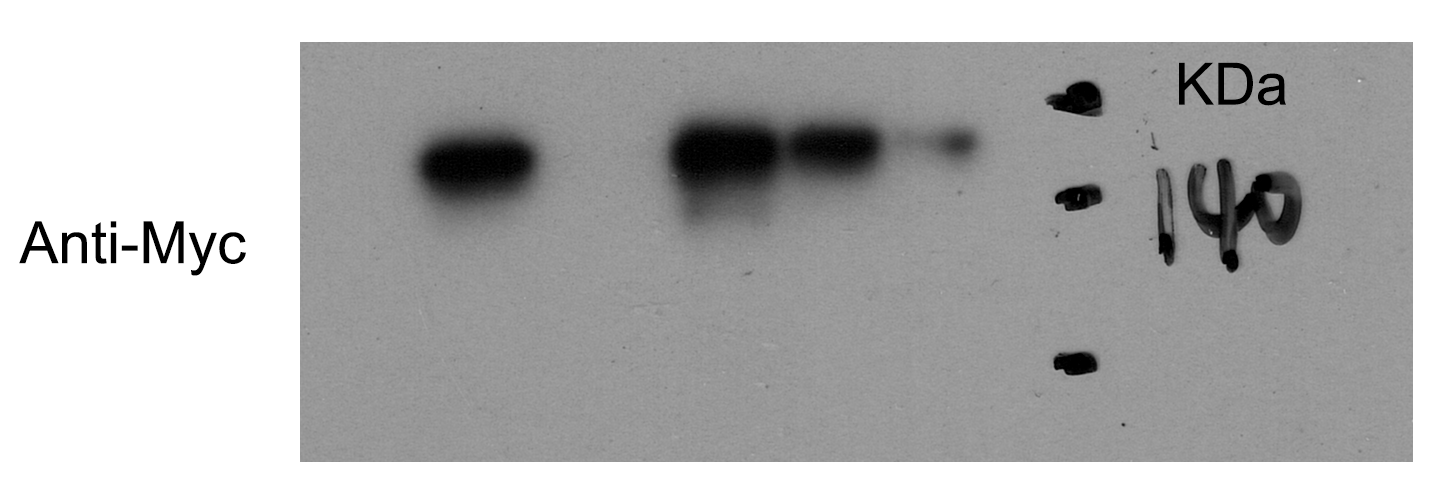

Supplement: Supplementary file 11 — Source data Fig. 7 [file 44318_2024_104_MOESM11_ESM.zip › Figure 7/7G/western Myc.tif]

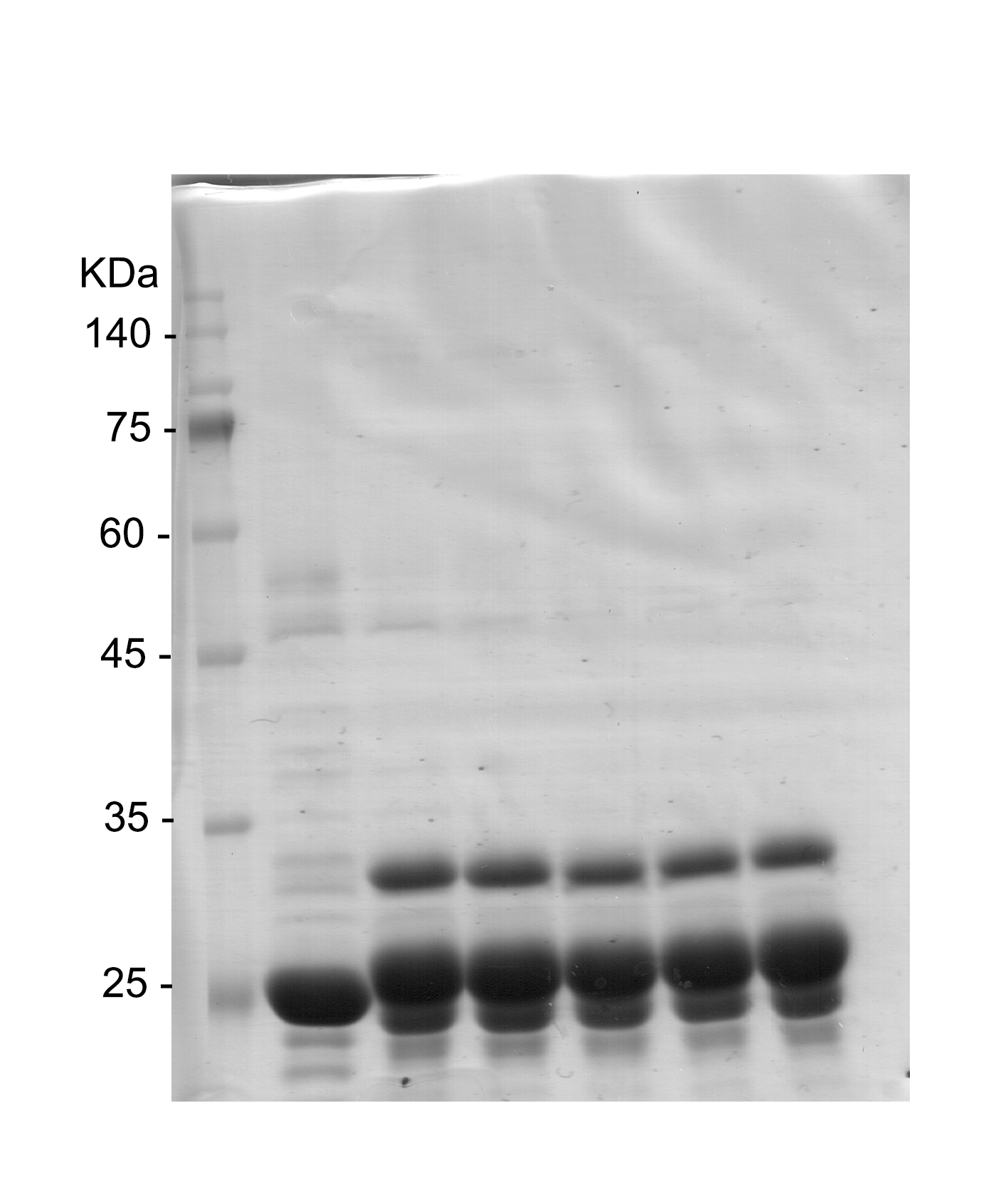

Supplement: Supplementary file 11 — Source data Fig. 7 [file 44318_2024_104_MOESM11_ESM.zip › Figure 7/7H/CBB.tif]

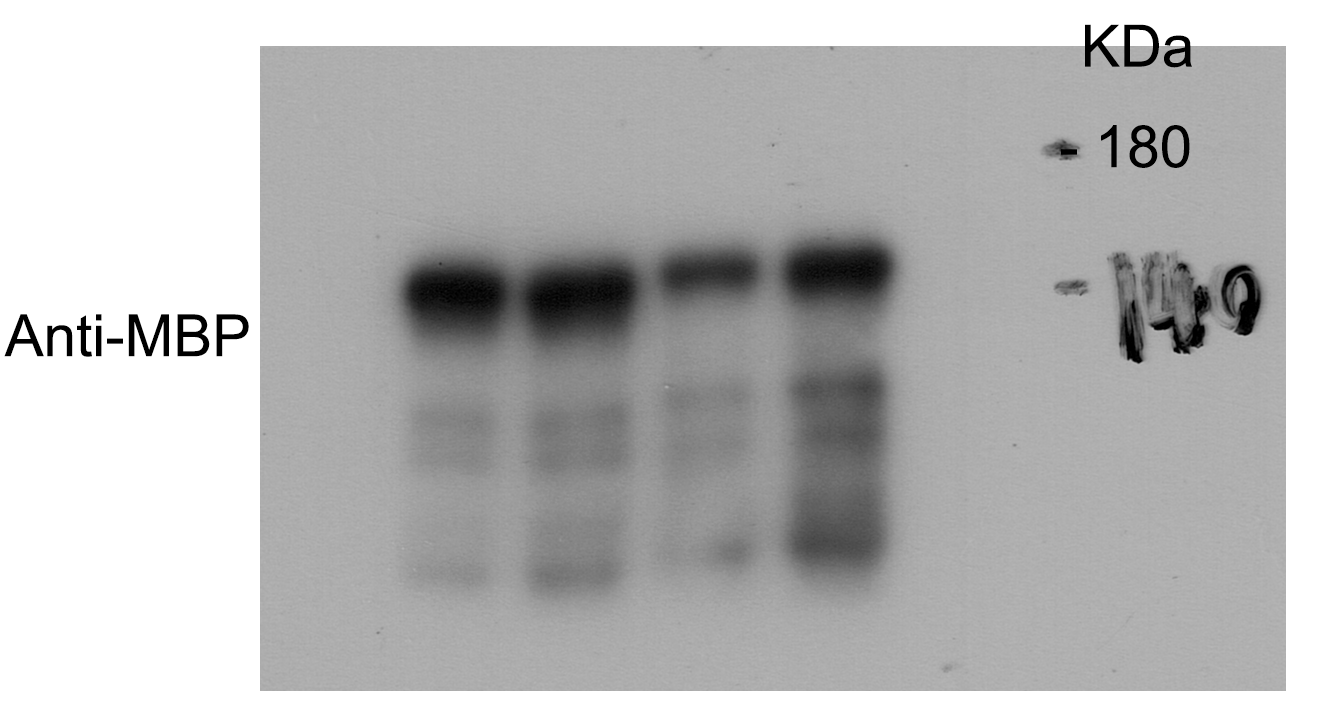

Supplement: Supplementary file 11 — Source data Fig. 7 [file 44318_2024_104_MOESM11_ESM.zip › Figure 7/7H/western MBP.tif]

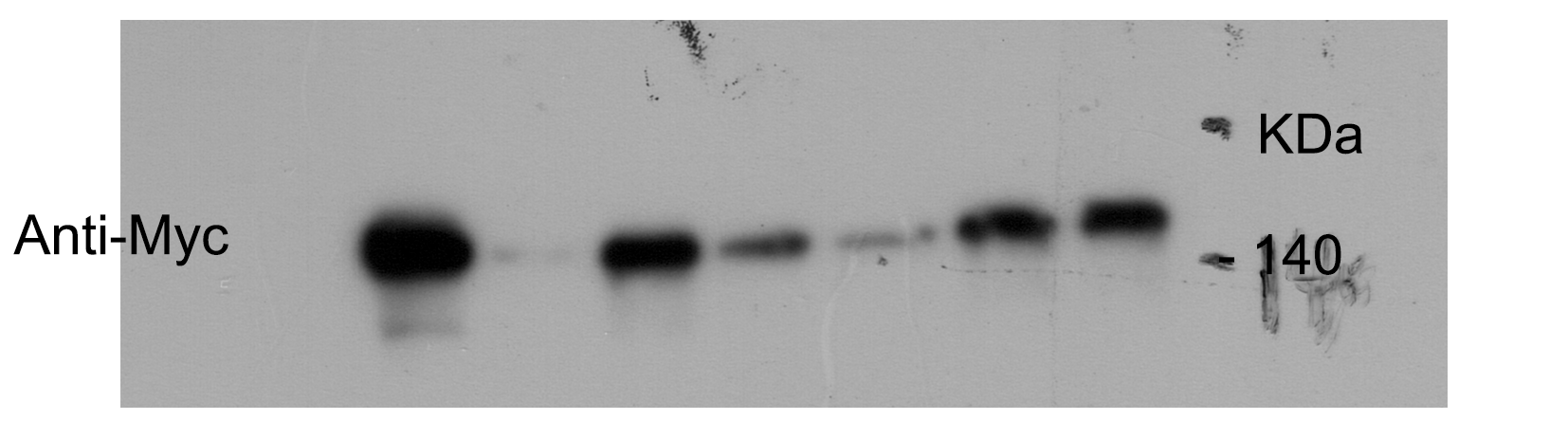

Supplement: Supplementary file 11 — Source data Fig. 7 [file 44318_2024_104_MOESM11_ESM.zip › Figure 7/7H/western Myc.tif]

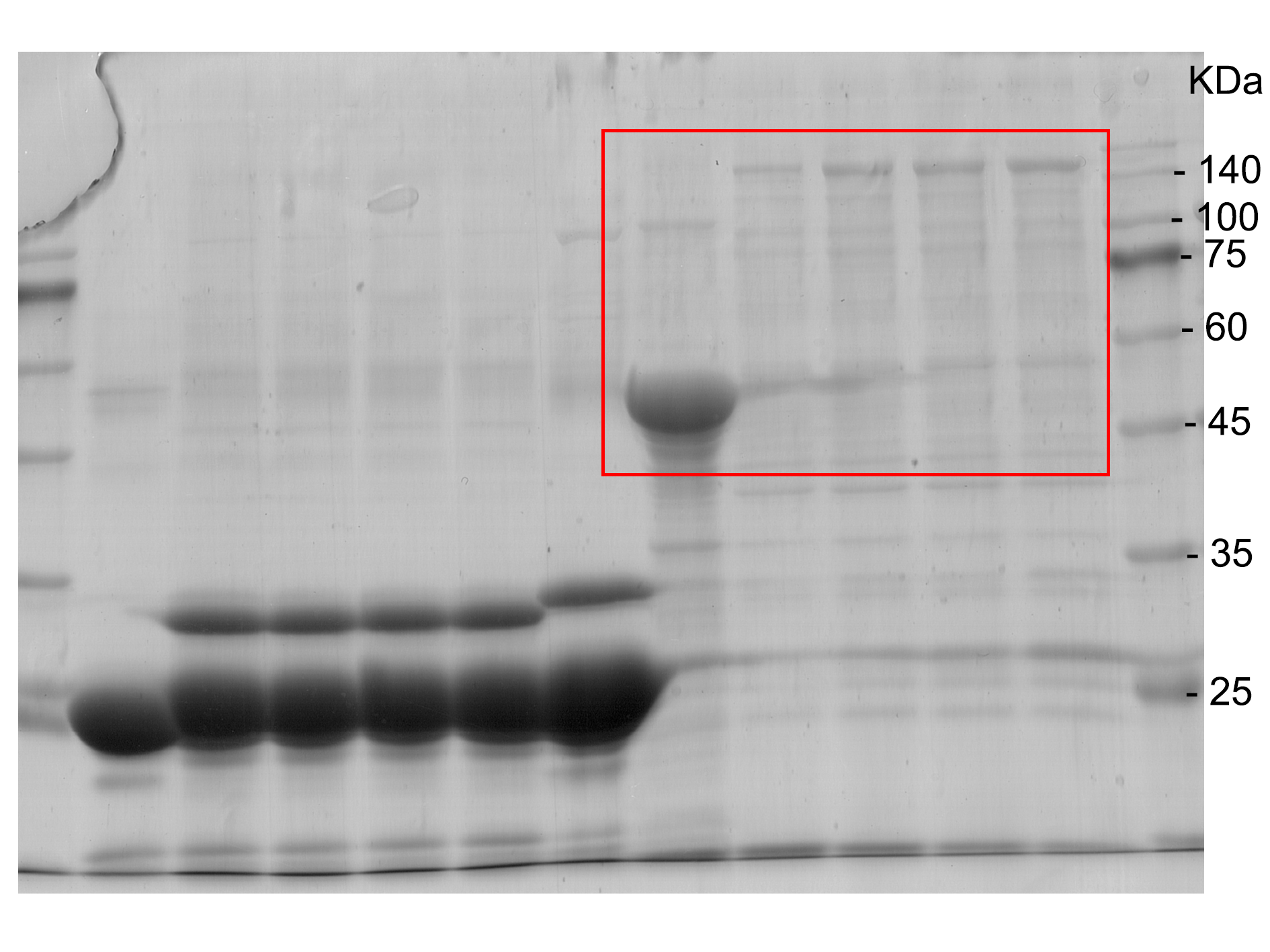

Supplement: Supplementary file 11 — Source data Fig. 7 [file 44318_2024_104_MOESM11_ESM.zip › Figure 7/7I/CBB.tif]

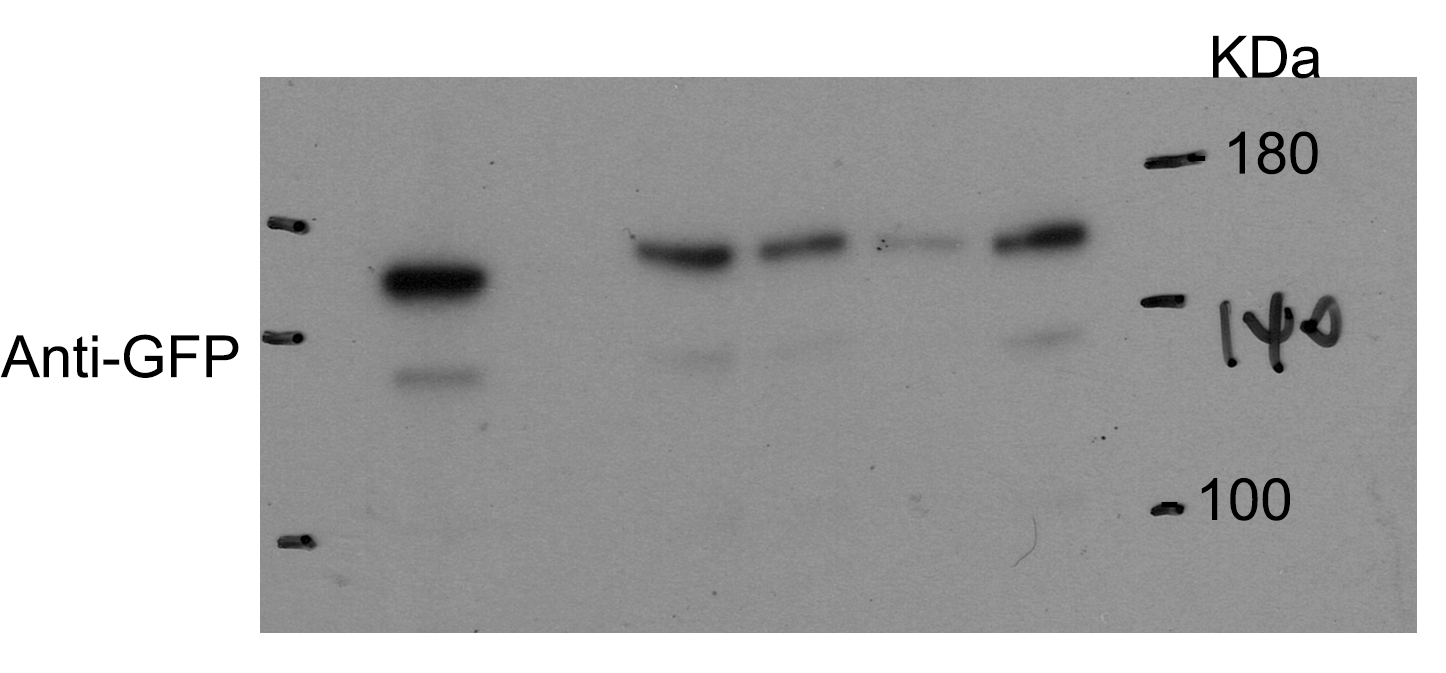

Supplement: Supplementary file 11 — Source data Fig. 7 [file 44318_2024_104_MOESM11_ESM.zip › Figure 7/7I/western GFP.tif]

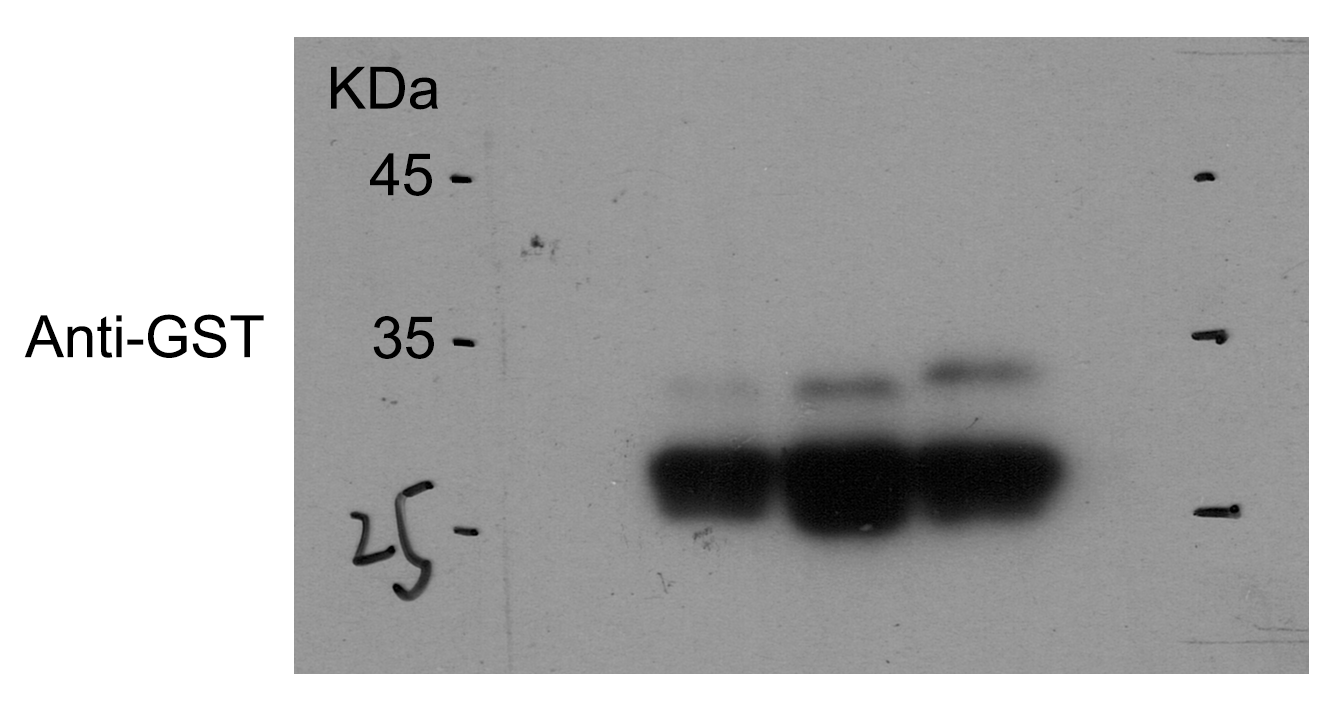

Supplement: Supplementary file 11 — Source data Fig. 7 [file 44318_2024_104_MOESM11_ESM.zip › Figure 7/7I/western GST.tif]

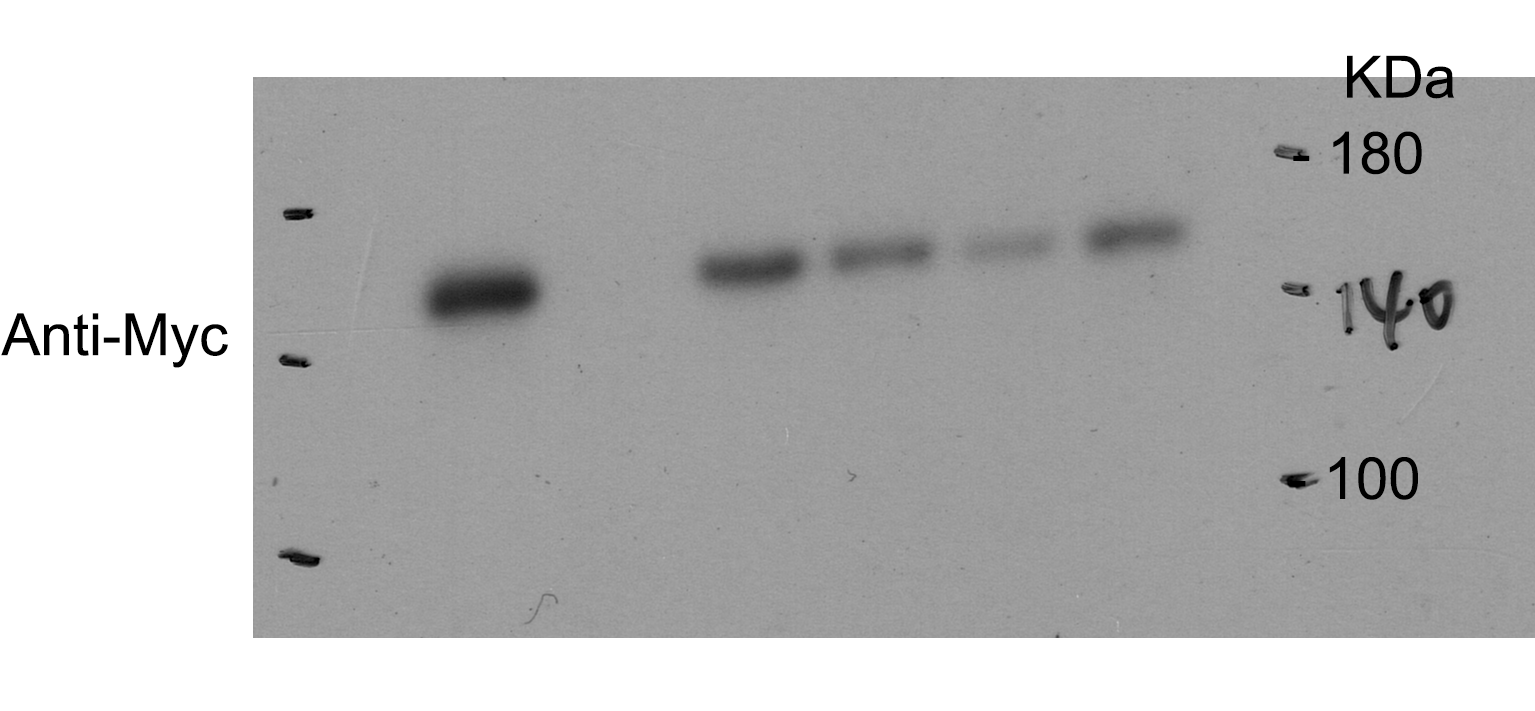

Supplement: Supplementary file 11 — Source data Fig. 7 [file 44318_2024_104_MOESM11_ESM.zip › Figure 7/7I/western Myc.tif]

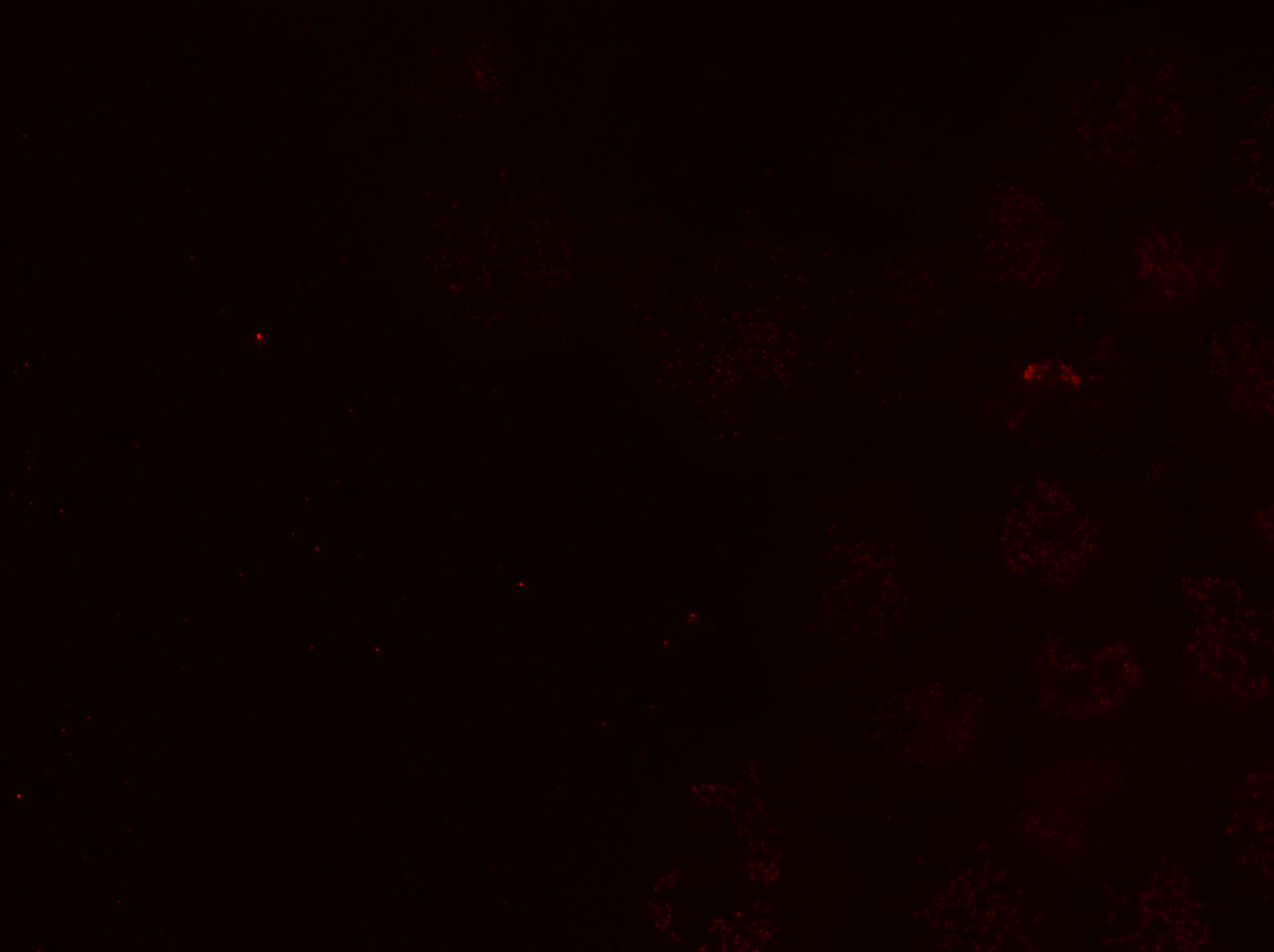

Supplement: Supplementary file 12 — Source data Fig. 8 [file 44318_2024_104_MOESM12_ESM.zip › Figure 8/8B/Sgo1-K492A cells-Completely separated sample picture-CENP-C.tif]

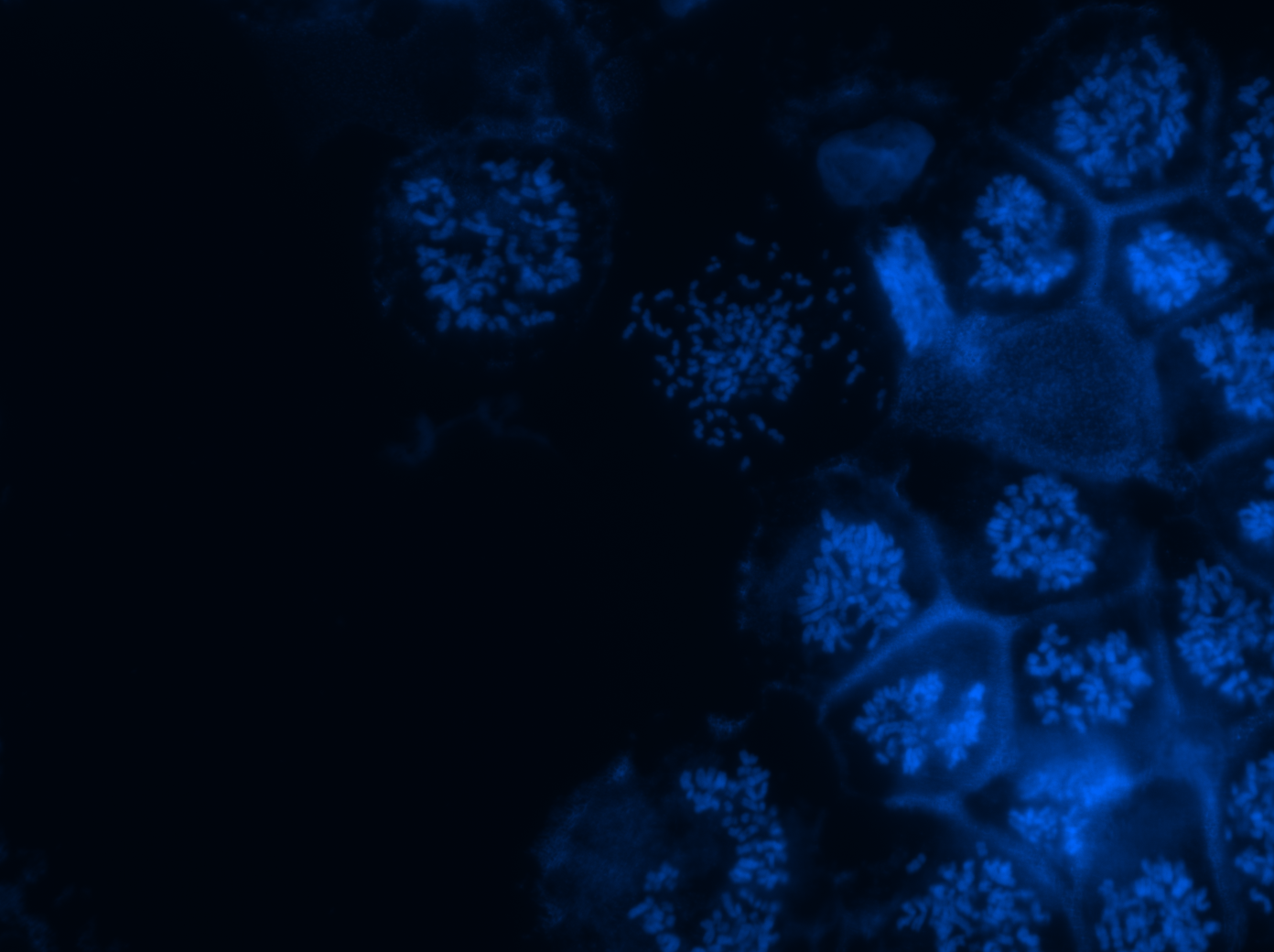

Supplement: Supplementary file 12 — Source data Fig. 8 [file 44318_2024_104_MOESM12_ESM.zip › Figure 8/8B/Sgo1-K492A cells-Completely separated sample picture-DNA.tif]

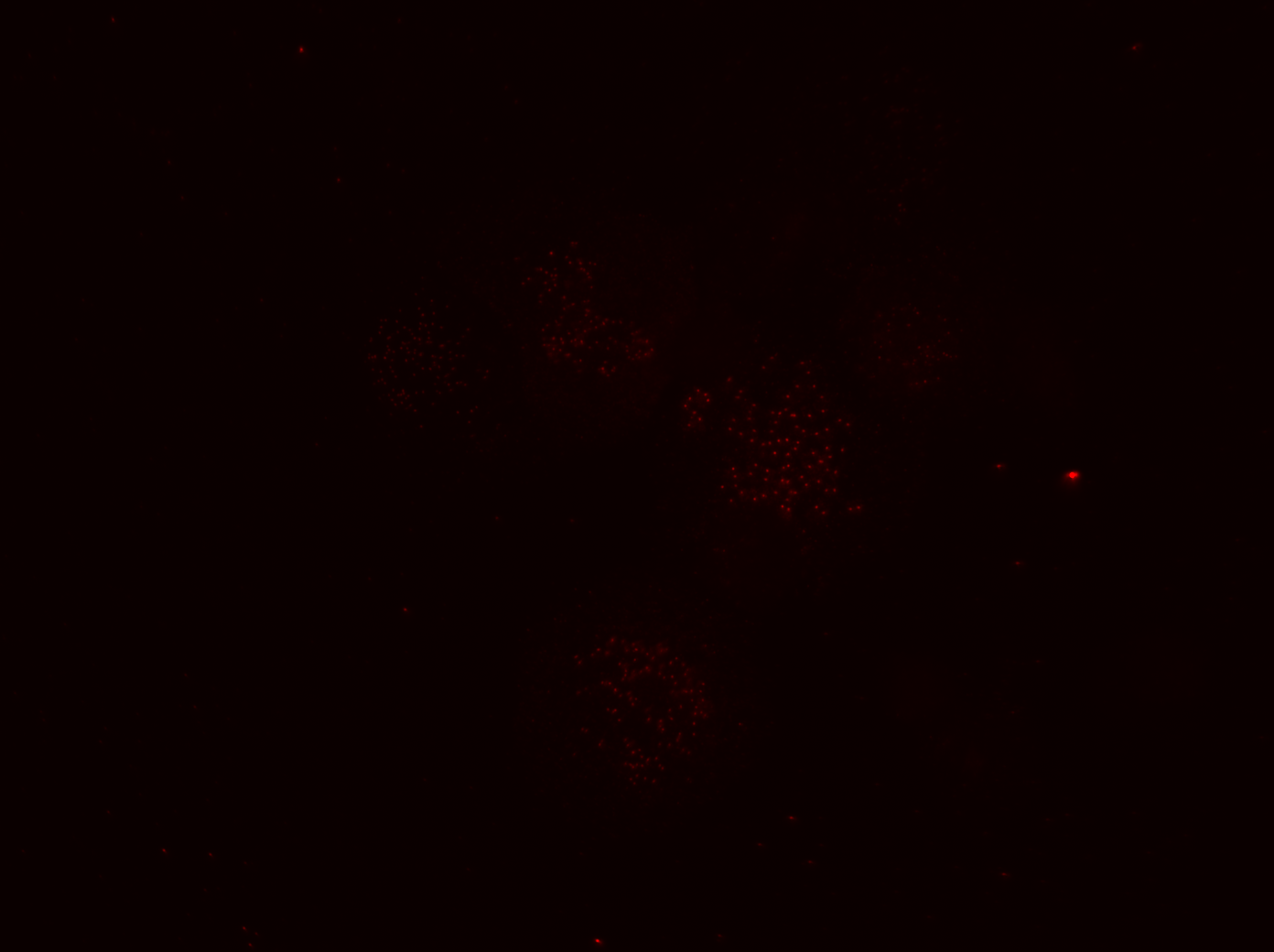

Supplement: Supplementary file 12 — Source data Fig. 8 [file 44318_2024_104_MOESM12_ESM.zip › Figure 8/8B/Sgo1-K492A cells-Partly separated sample picture-CENP-C.tif]

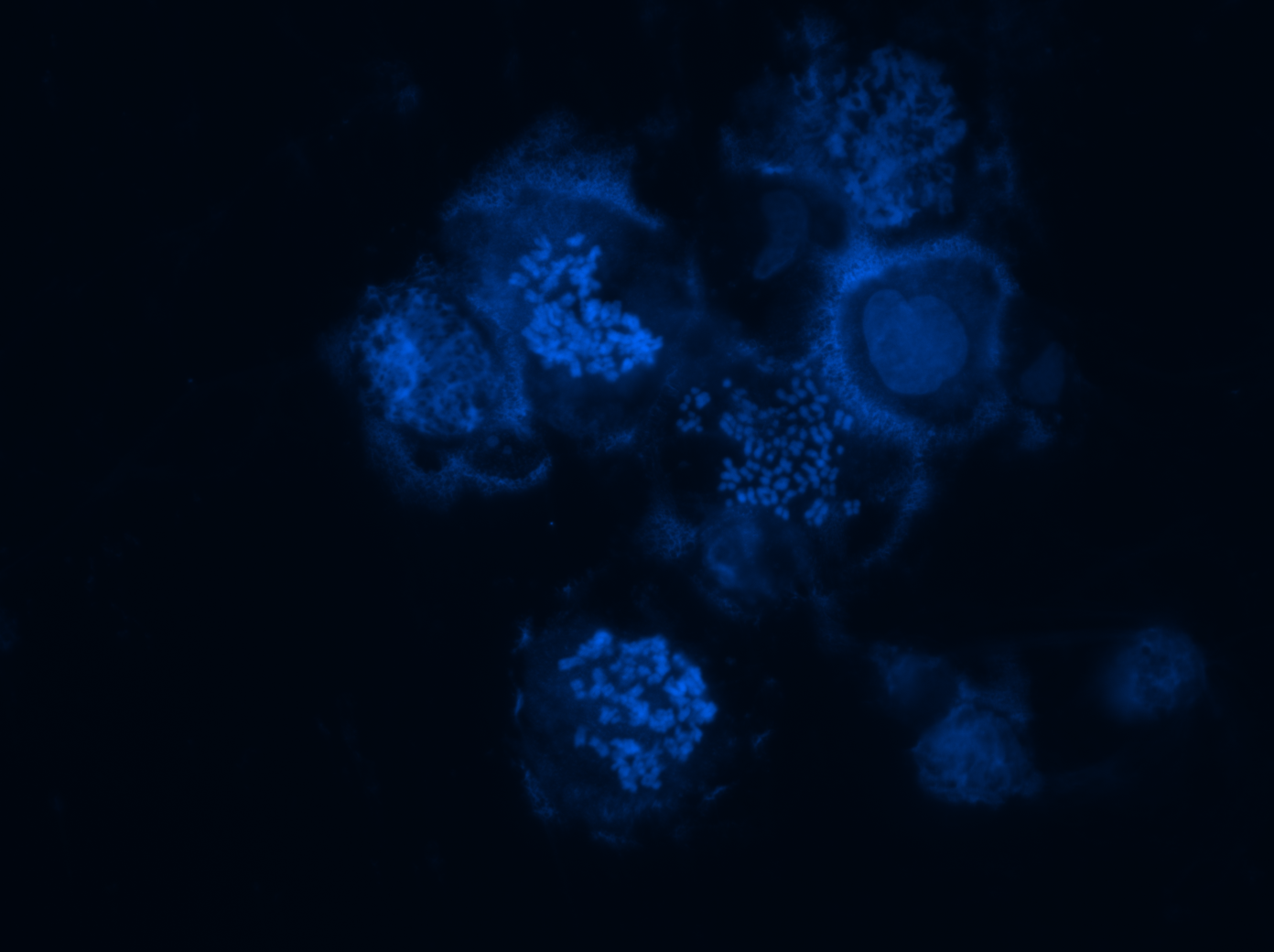

Supplement: Supplementary file 12 — Source data Fig. 8 [file 44318_2024_104_MOESM12_ESM.zip › Figure 8/8B/Sgo1-K492A cells-Partly separated sample picture-DNA.tif]

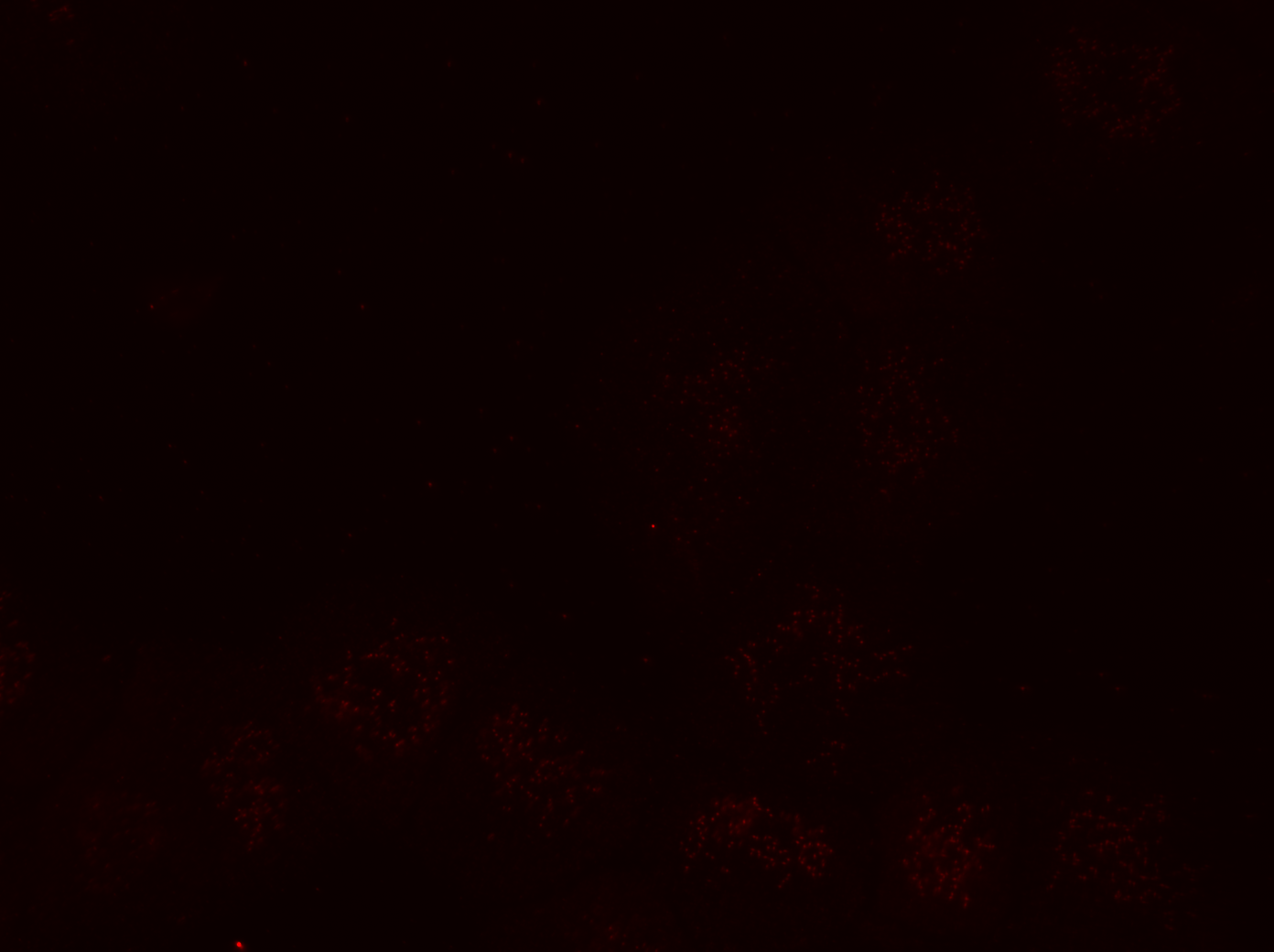

Supplement: Supplementary file 12 — Source data Fig. 8 [file 44318_2024_104_MOESM12_ESM.zip › Figure 8/8B/Sgo1-K492A cells-Unseparated sample picture-CENP-C.tif]

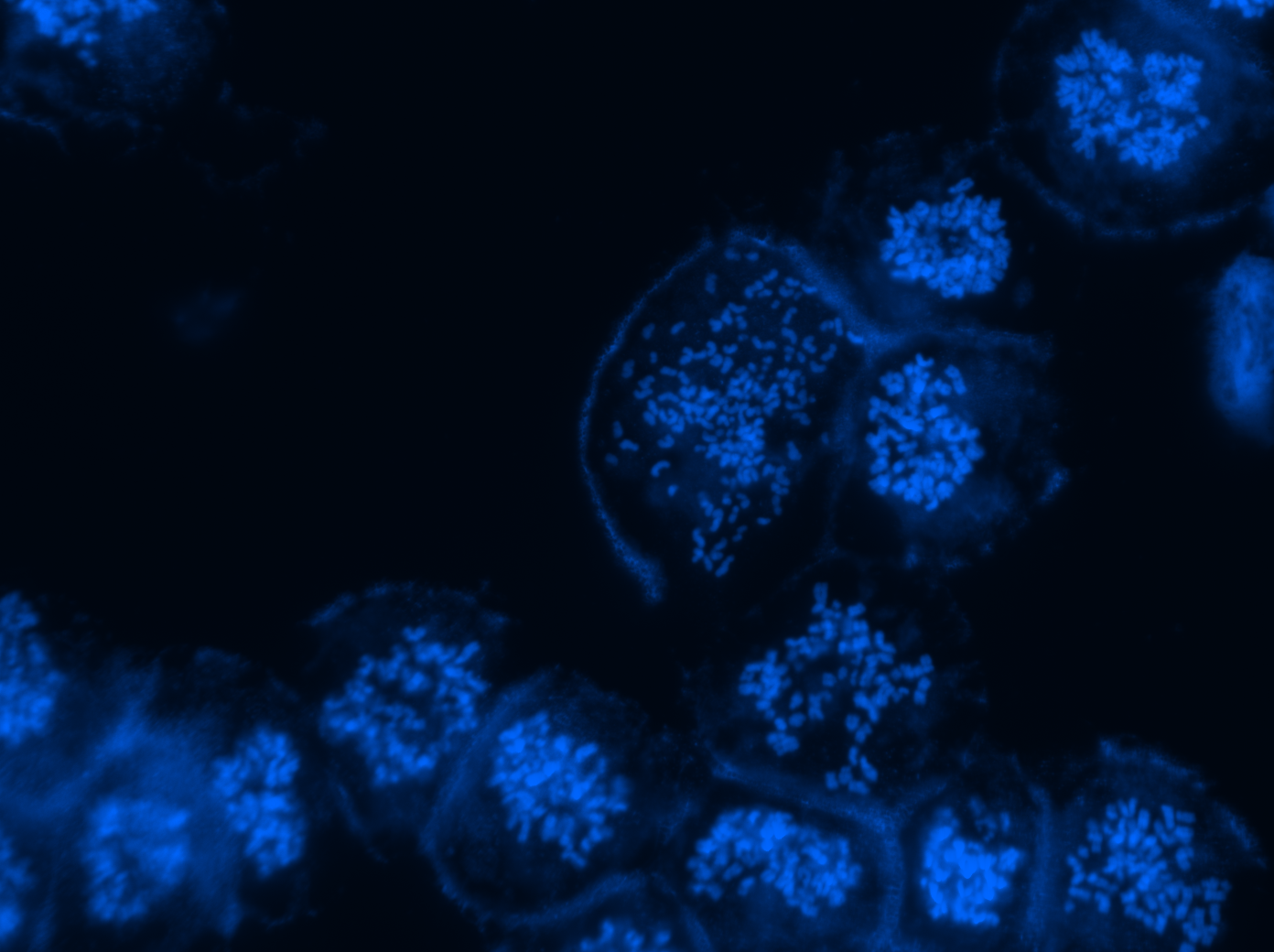

Supplement: Supplementary file 12 — Source data Fig. 8 [file 44318_2024_104_MOESM12_ESM.zip › Figure 8/8B/Sgo1-K492A cells-Unseparated sample picture-DNA.tif]

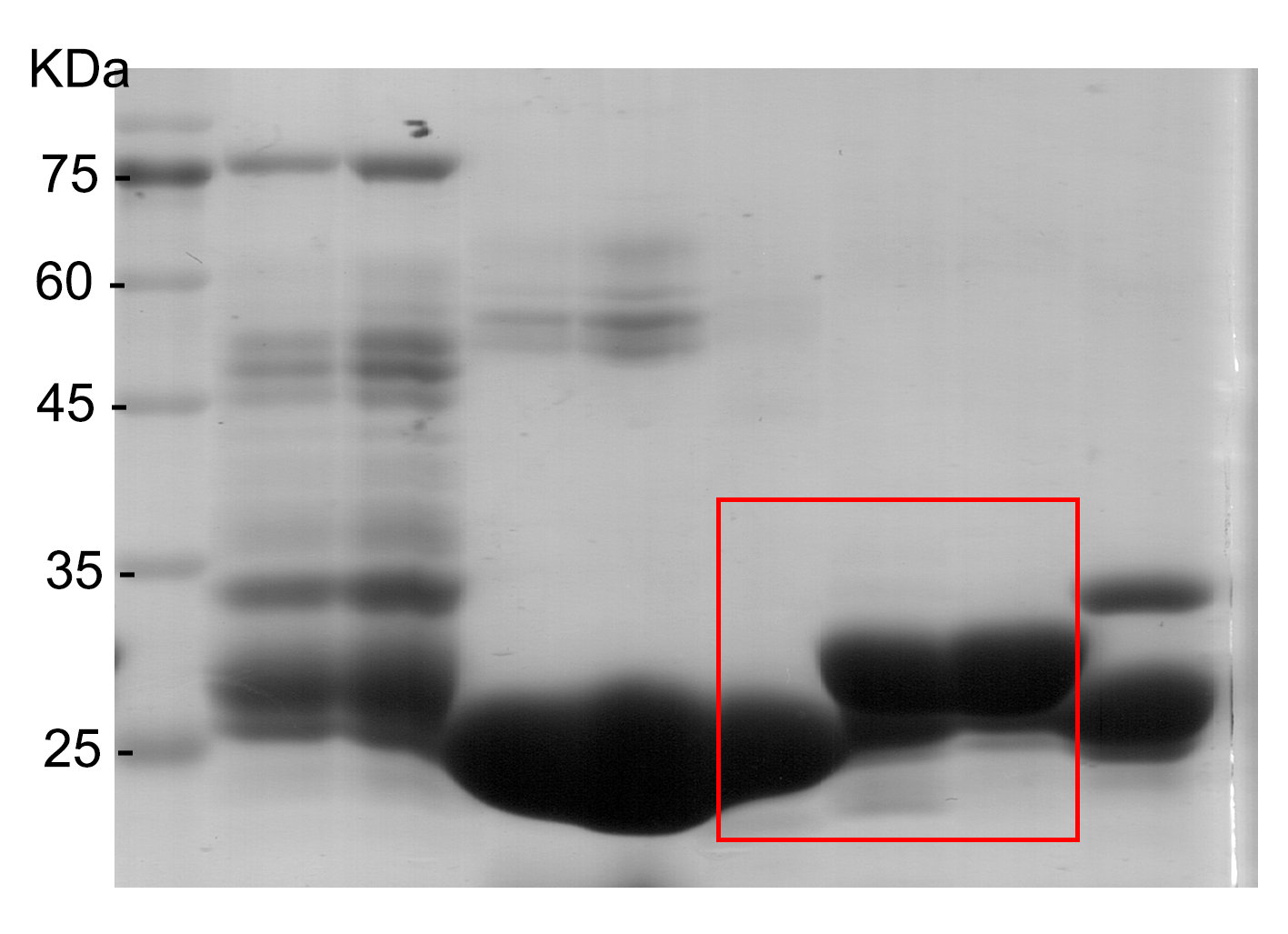

Supplement: Supplementary file 12 — Source data Fig. 8 [file 44318_2024_104_MOESM12_ESM.zip › Figure 8/8D/CBB.tif]

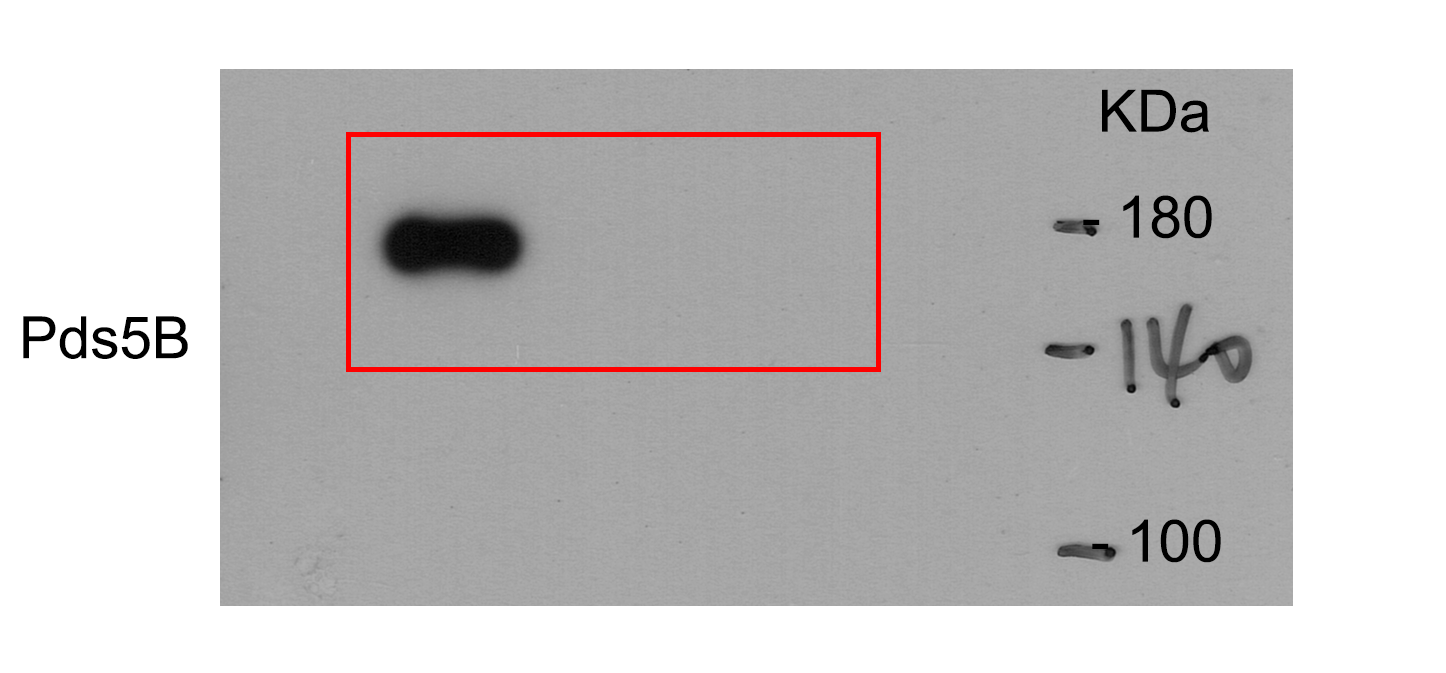

Supplement: Supplementary file 12 — Source data Fig. 8 [file 44318_2024_104_MOESM12_ESM.zip › Figure 8/8D/western Pds5B.tif]

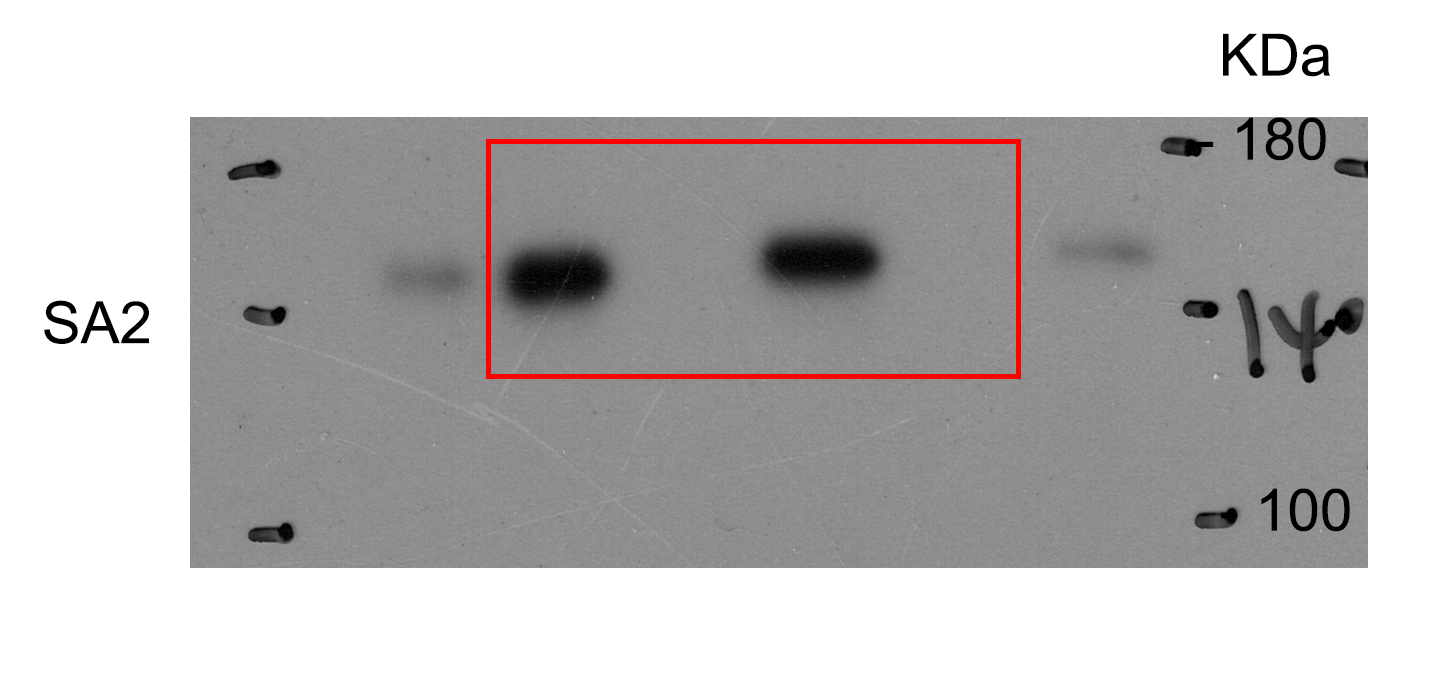

Supplement: Supplementary file 12 — Source data Fig. 8 [file 44318_2024_104_MOESM12_ESM.zip › Figure 8/8D/western SA2.tif]

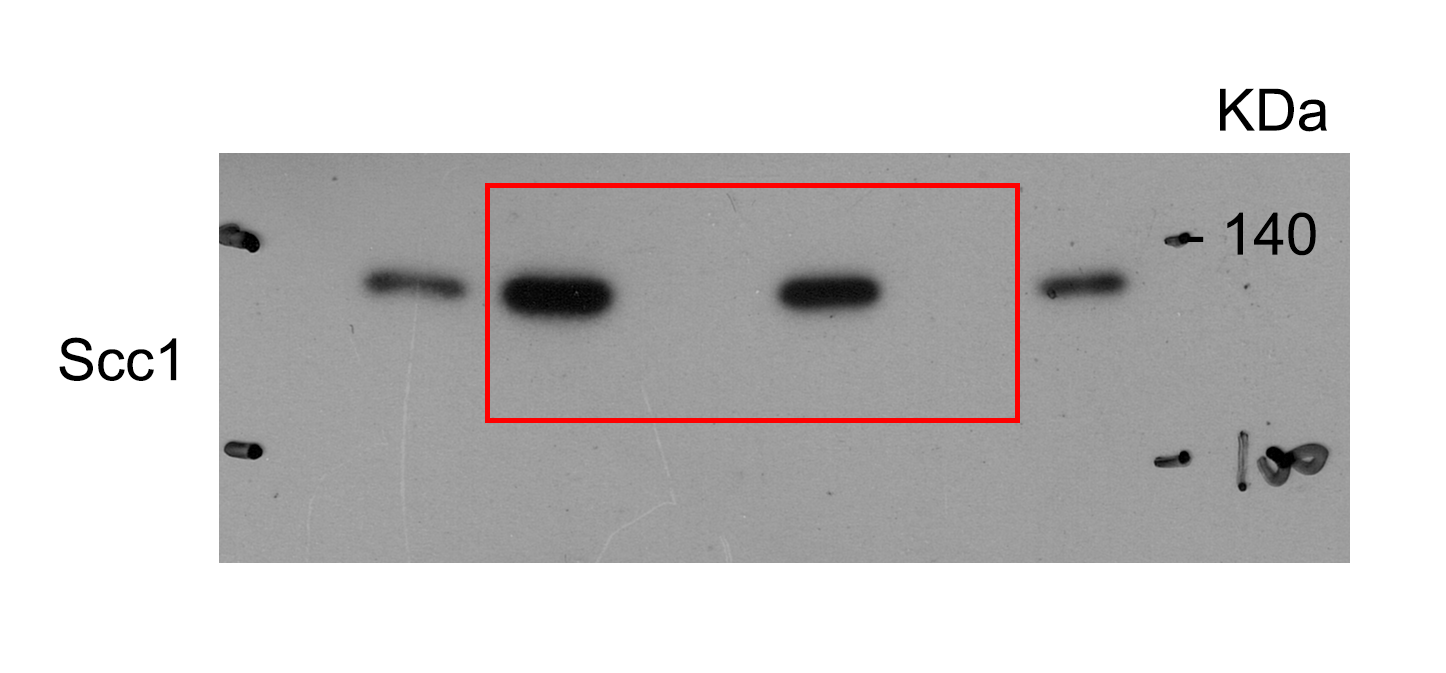

Supplement: Supplementary file 12 — Source data Fig. 8 [file 44318_2024_104_MOESM12_ESM.zip › Figure 8/8D/western Scc1.tif]

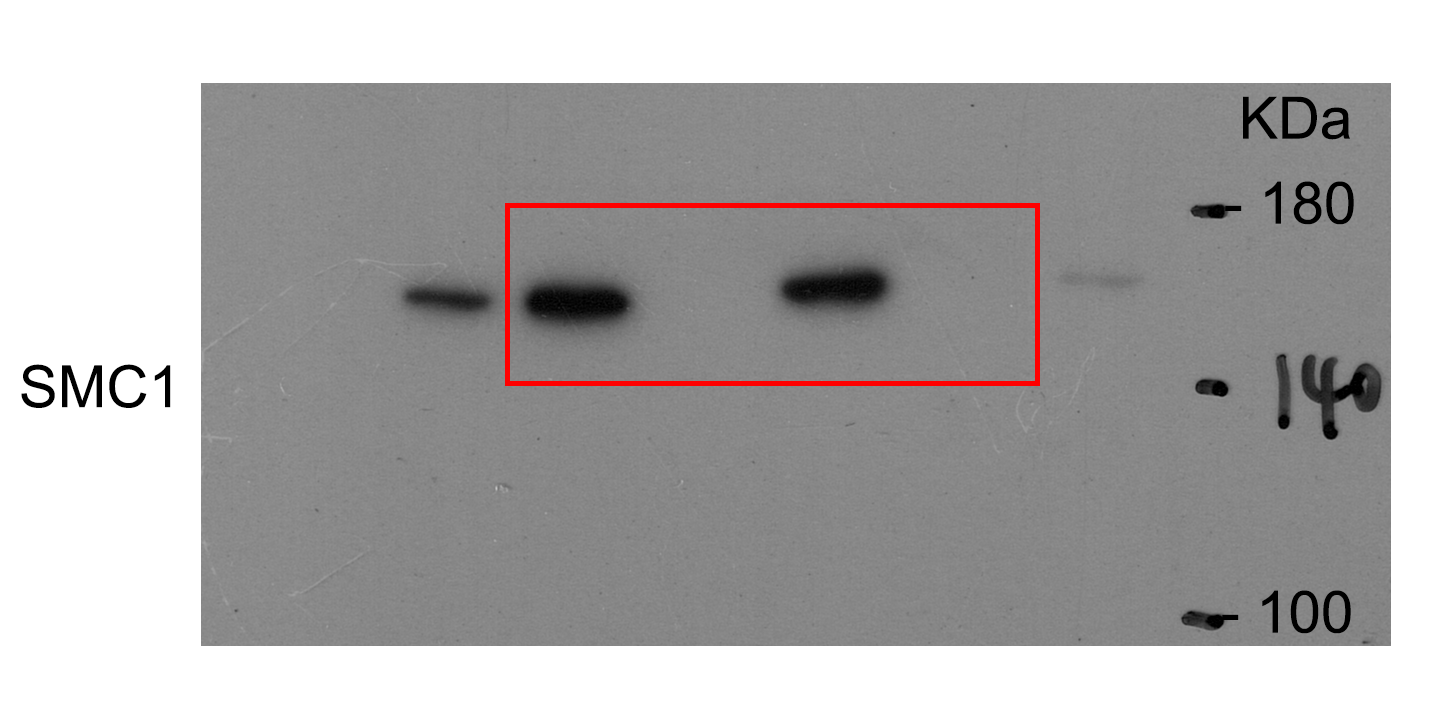

Supplement: Supplementary file 12 — Source data Fig. 8 [file 44318_2024_104_MOESM12_ESM.zip › Figure 8/8D/western SMC1.tif]

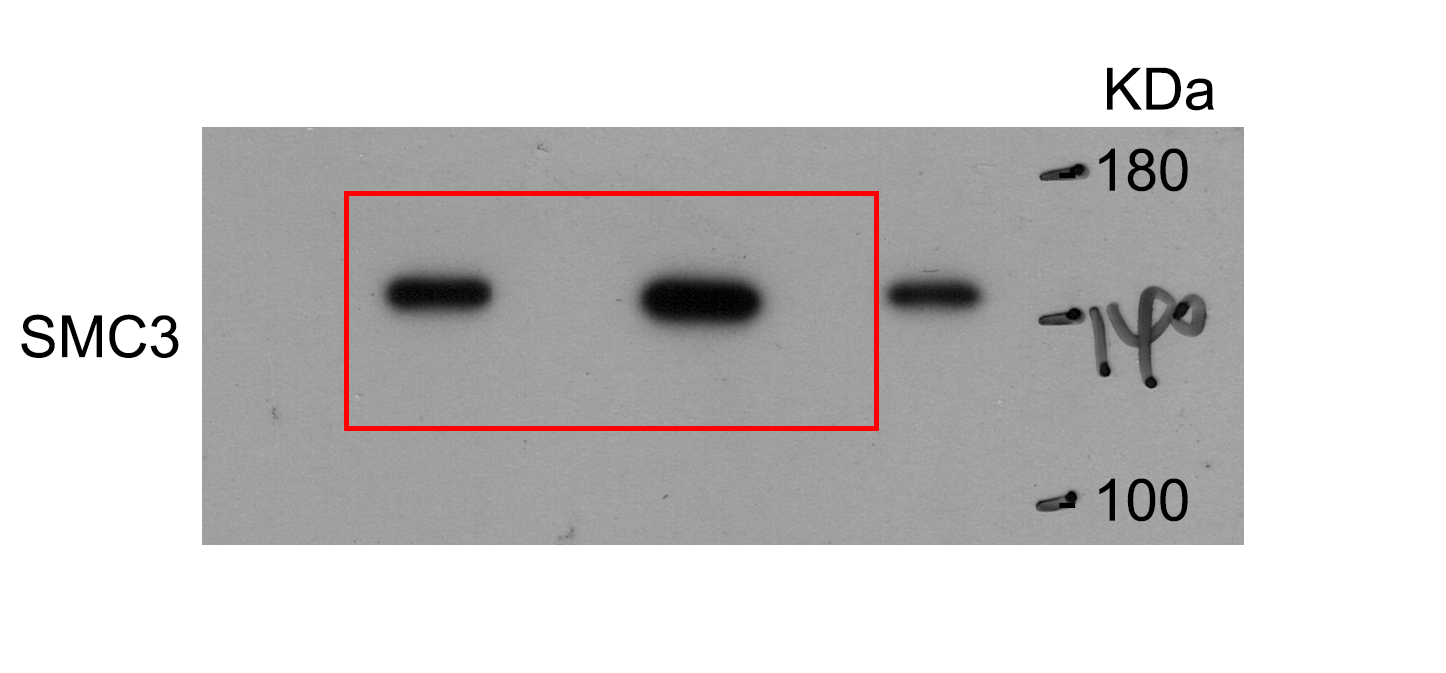

Supplement: Supplementary file 12 — Source data Fig. 8 [file 44318_2024_104_MOESM12_ESM.zip › Figure 8/8D/western SMC3.tif]

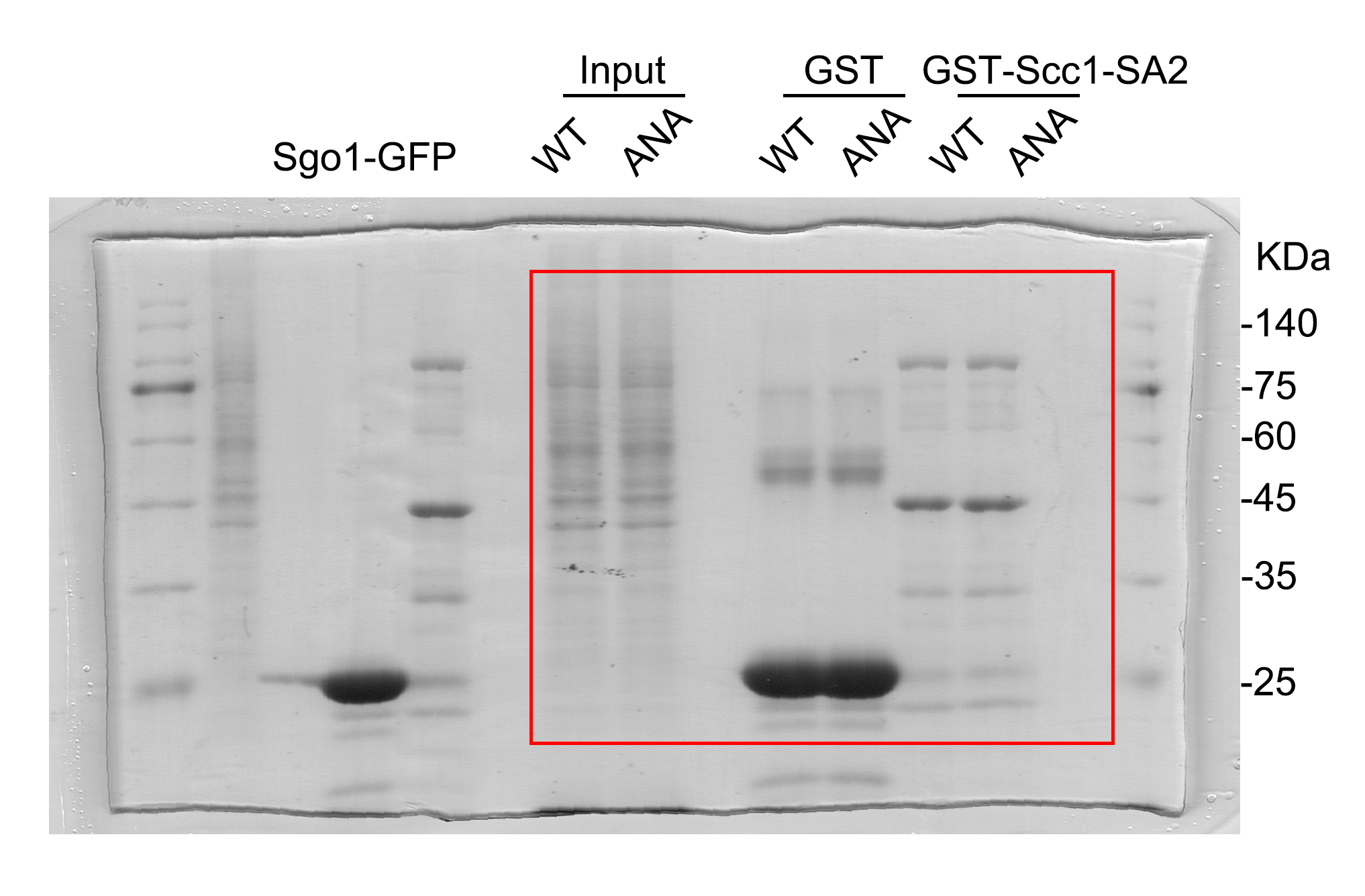

Supplement: Supplementary file 12 — Source data Fig. 8 [file 44318_2024_104_MOESM12_ESM.zip › Figure 8/8E/8E CBB.tif]

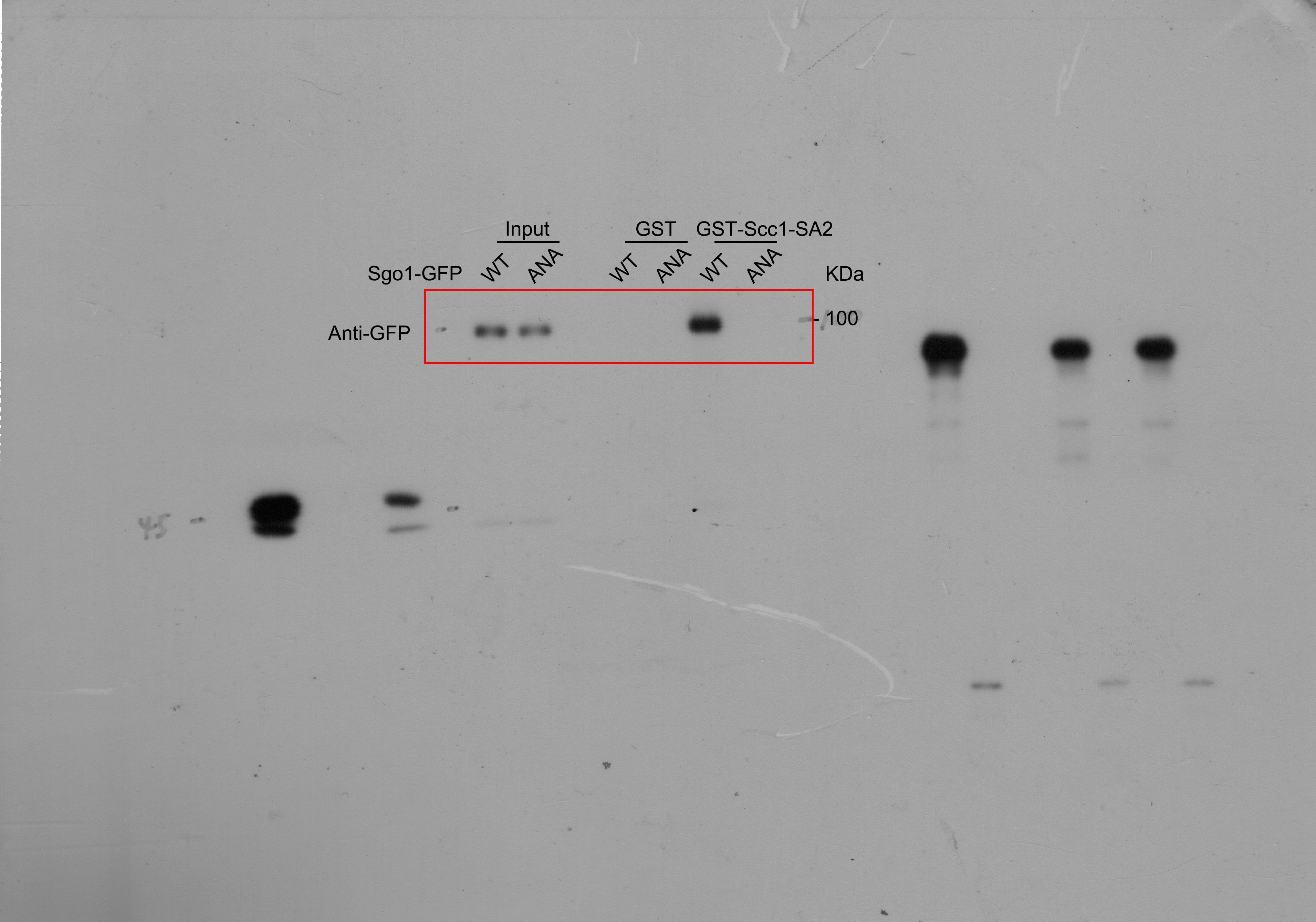

Supplement: Supplementary file 12 — Source data Fig. 8 [file 44318_2024_104_MOESM12_ESM.zip › Figure 8/8E/8E WB.tif]

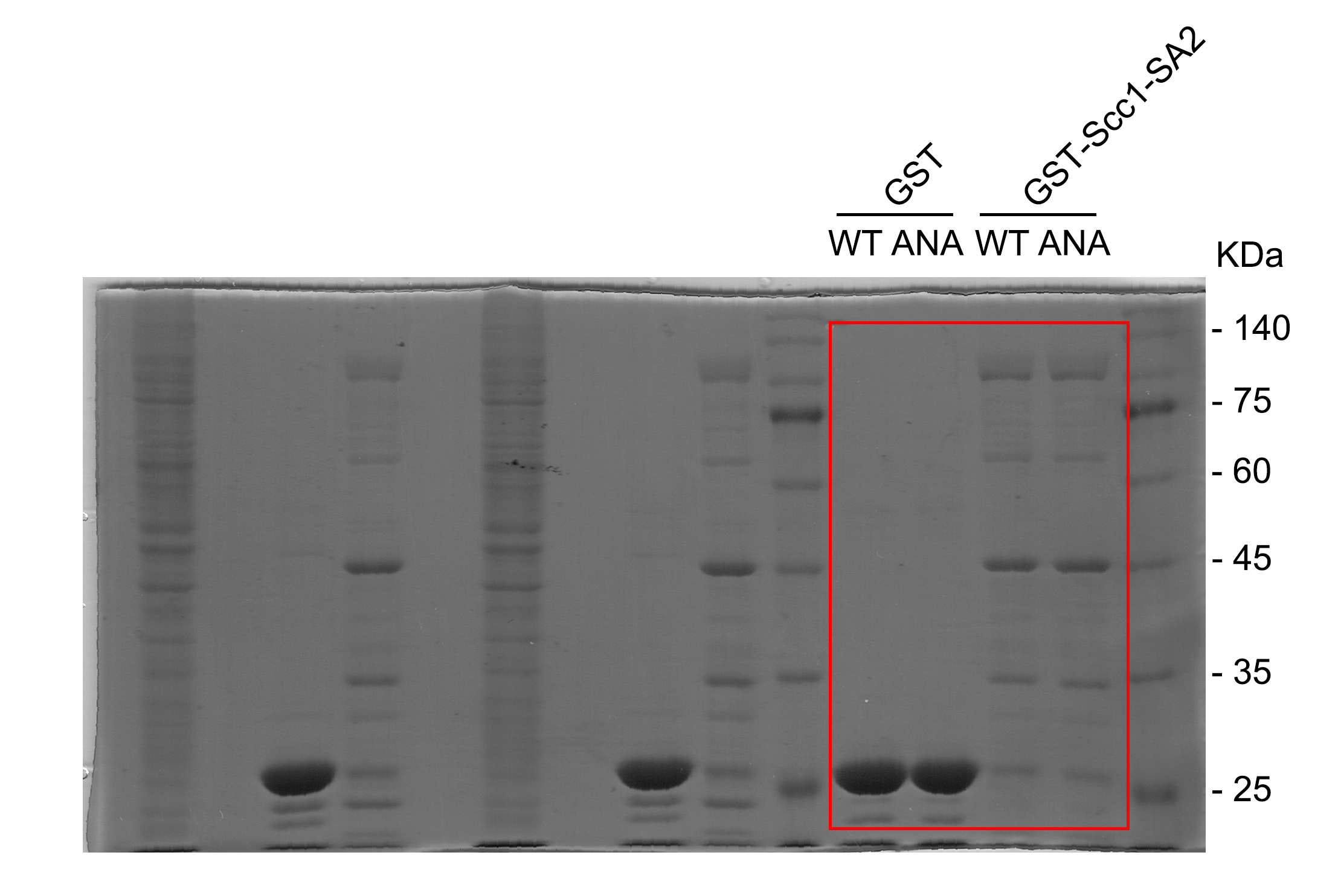

Supplement: Supplementary file 12 — Source data Fig. 8 [file 44318_2024_104_MOESM12_ESM.zip › Figure 8/8F/8F CBB.tif]

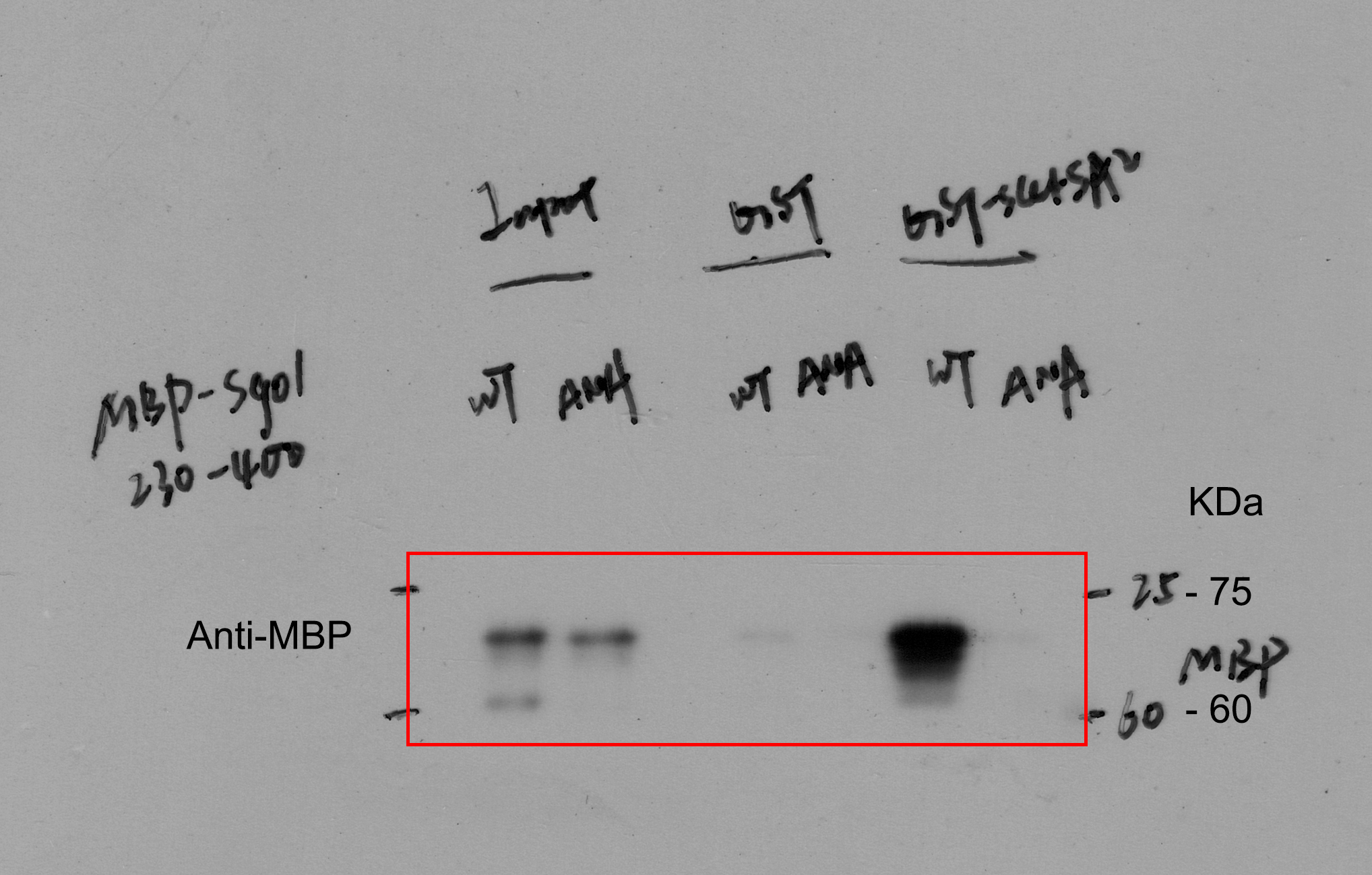

Supplement: Supplementary file 12 — Source data Fig. 8 [file 44318_2024_104_MOESM12_ESM.zip › Figure 8/8F/8F WB.tif]

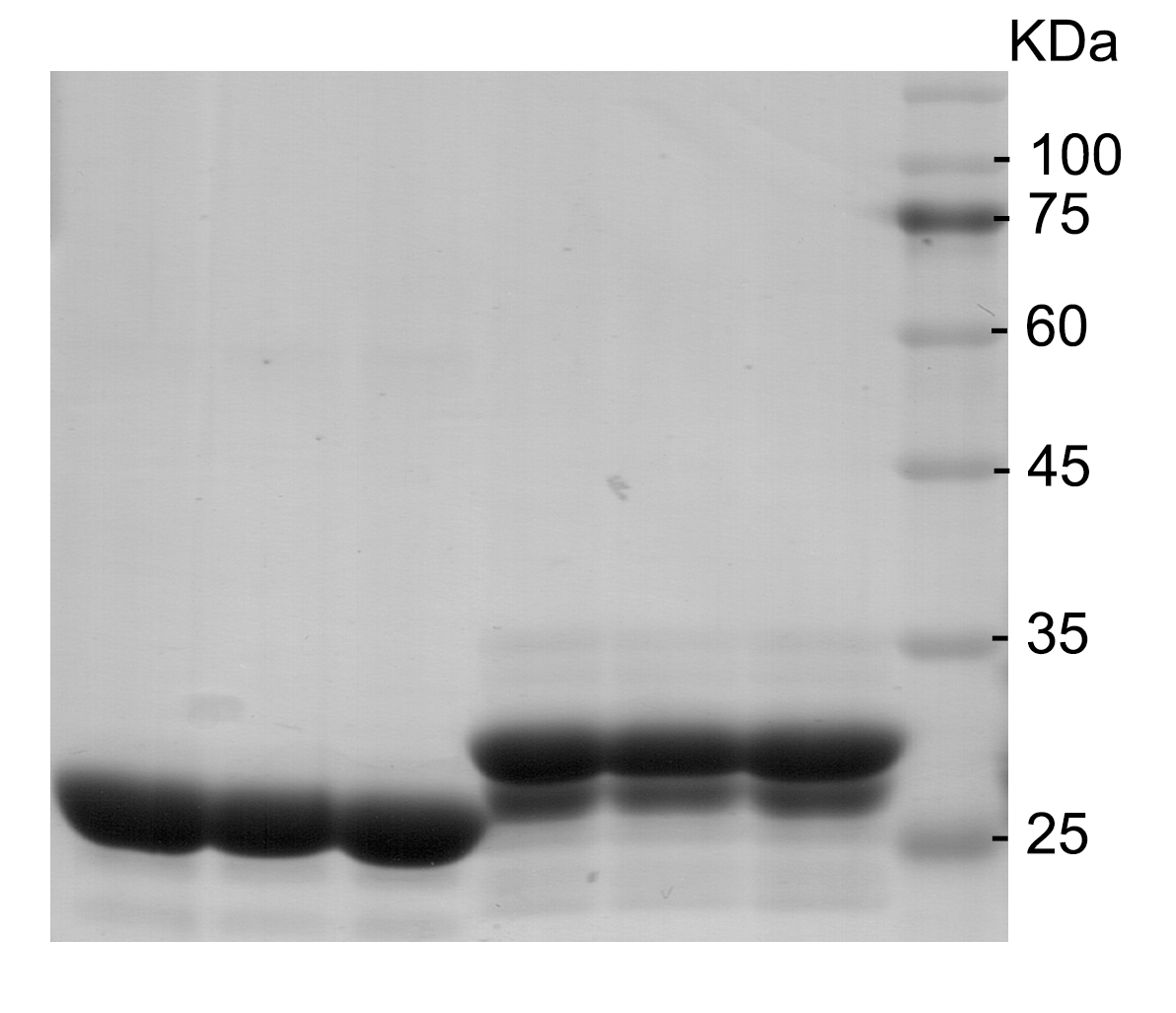

Supplement: Supplementary file 12 — Source data Fig. 8 [file 44318_2024_104_MOESM12_ESM.zip › Figure 8/8G/CBB.tif]

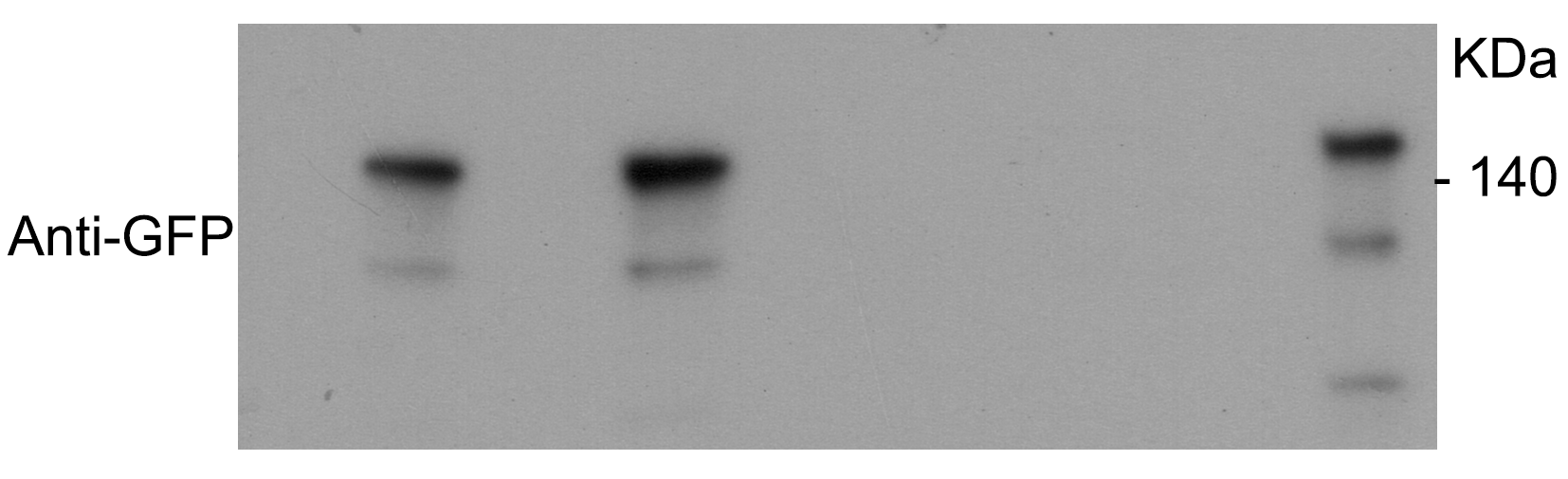

Supplement: Supplementary file 12 — Source data Fig. 8 [file 44318_2024_104_MOESM12_ESM.zip › Figure 8/8G/western GFP.tif]

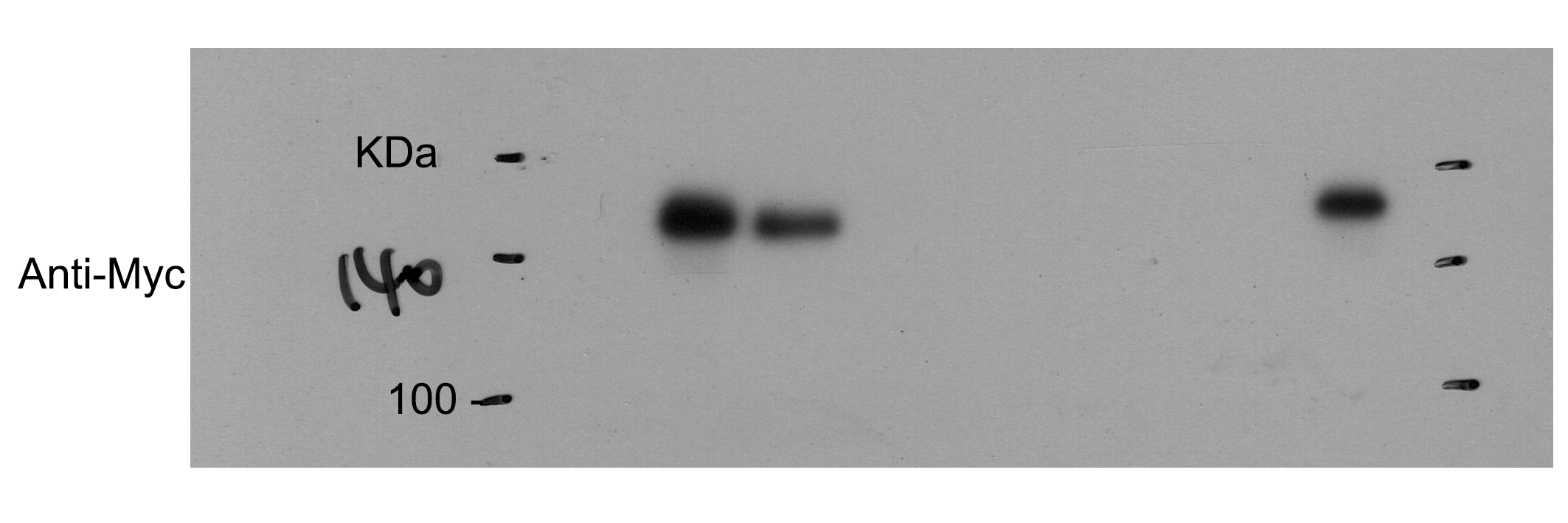

Supplement: Supplementary file 12 — Source data Fig. 8 [file 44318_2024_104_MOESM12_ESM.zip › Figure 8/8G/western Myc.tif]

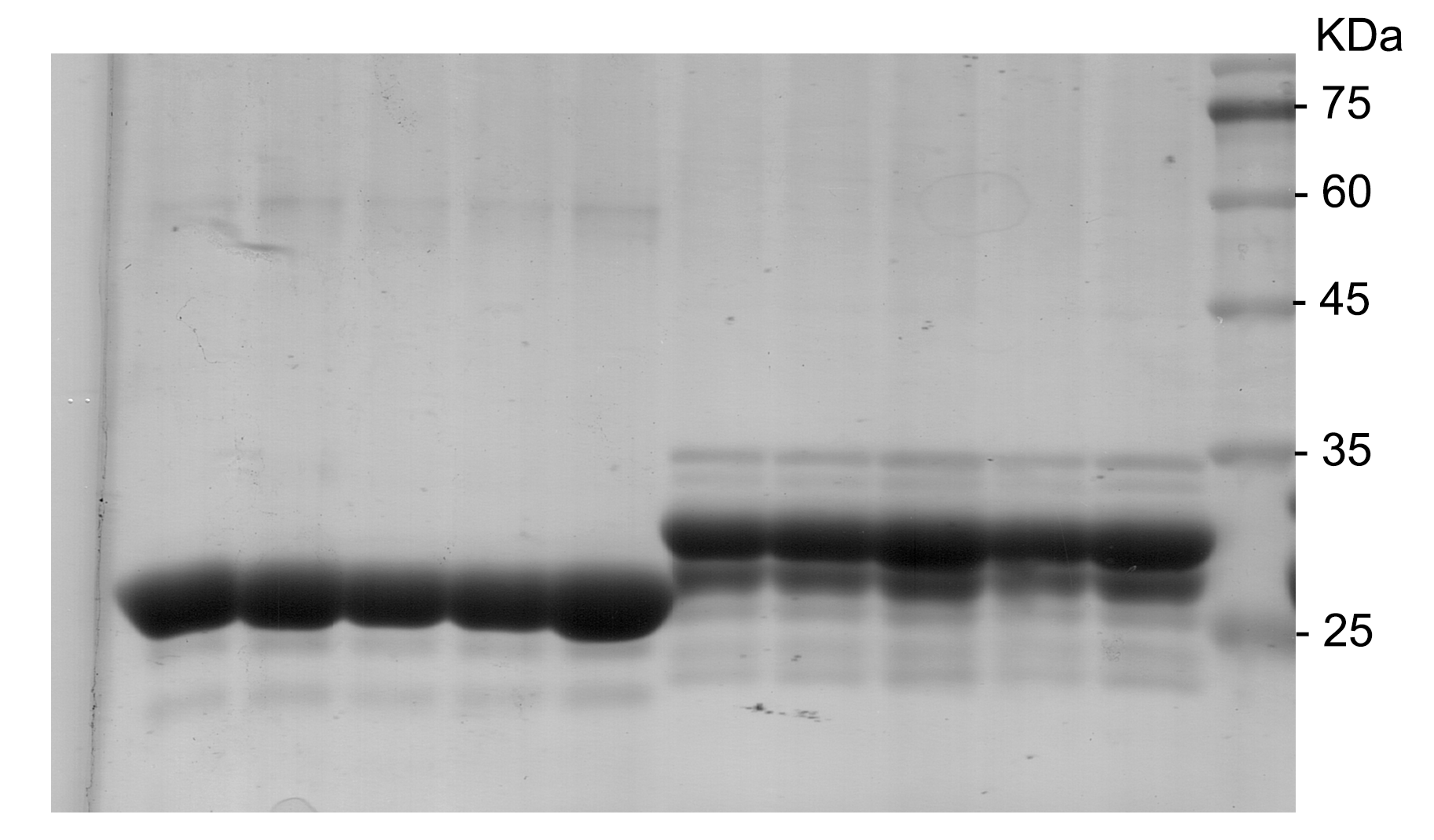

Supplement: Supplementary file 12 — Source data Fig. 8 [file 44318_2024_104_MOESM12_ESM.zip › Figure 8/8H/CBB.tif]

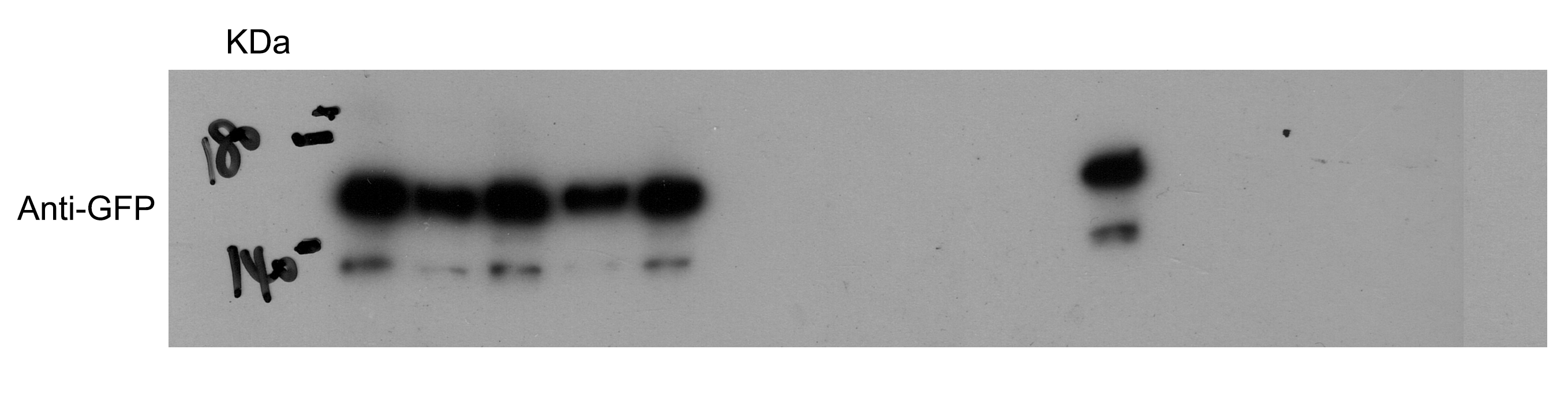

Supplement: Supplementary file 12 — Source data Fig. 8 [file 44318_2024_104_MOESM12_ESM.zip › Figure 8/8H/western GFP.tif]

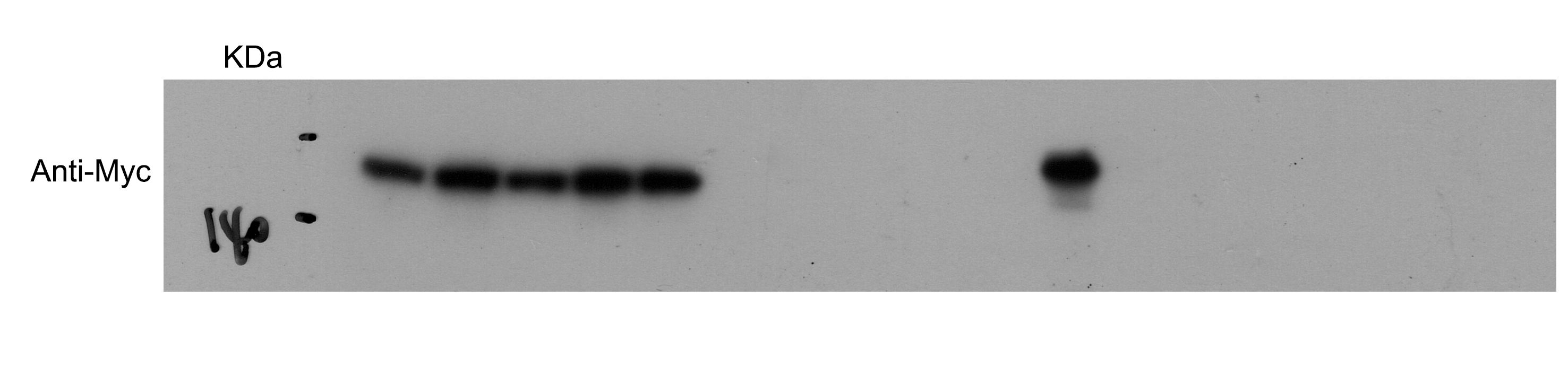

Supplement: Supplementary file 12 — Source data Fig. 8 [file 44318_2024_104_MOESM12_ESM.zip › Figure 8/8H/western Myc.tif]

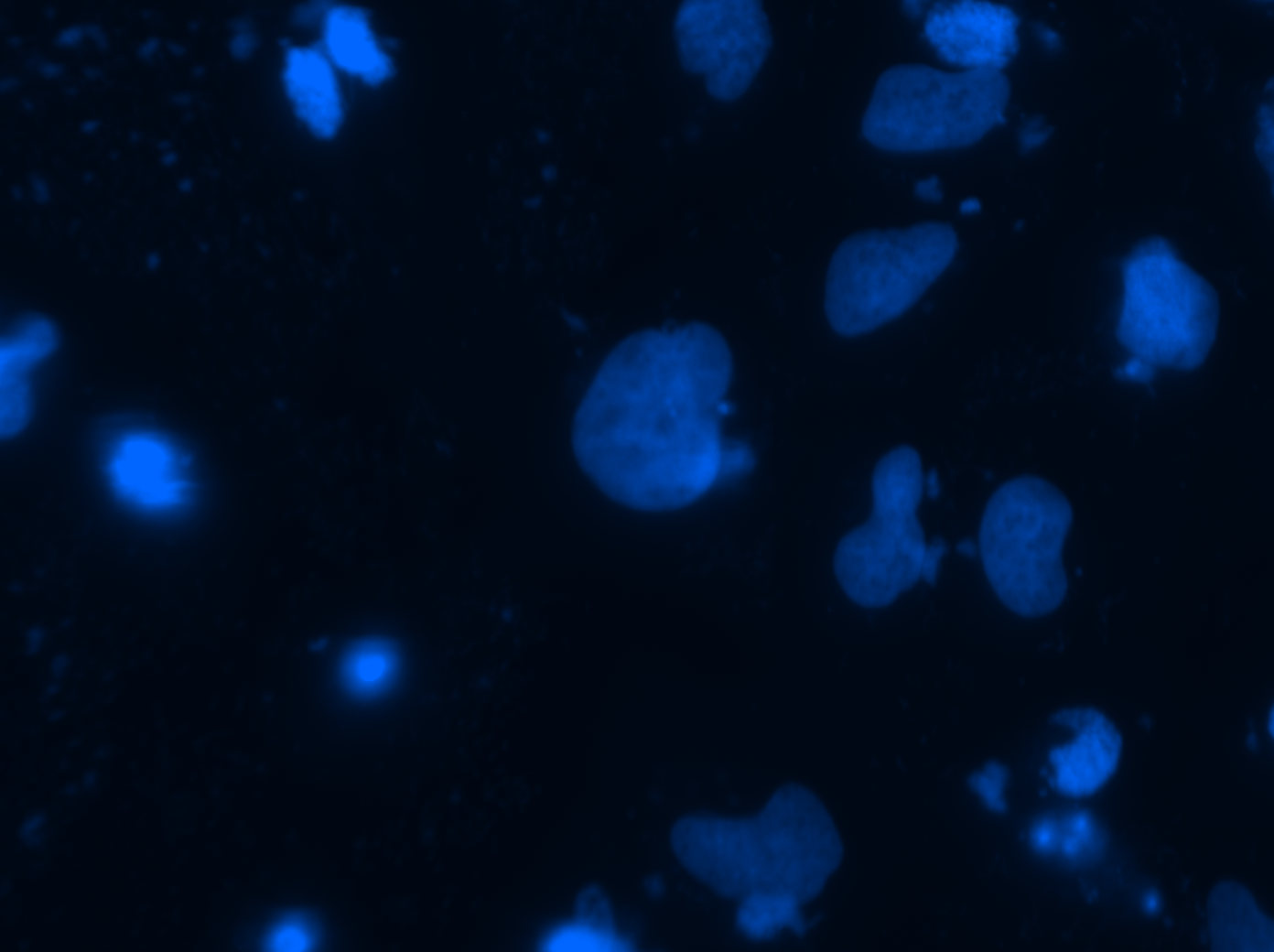

Supplement: Supplementary file 12 — Source data Fig. 8 [file 44318_2024_104_MOESM12_ESM.zip › Figure 8/8I/EGFP-LacI+Myc-SA2+Sgo1-DNA.tif]

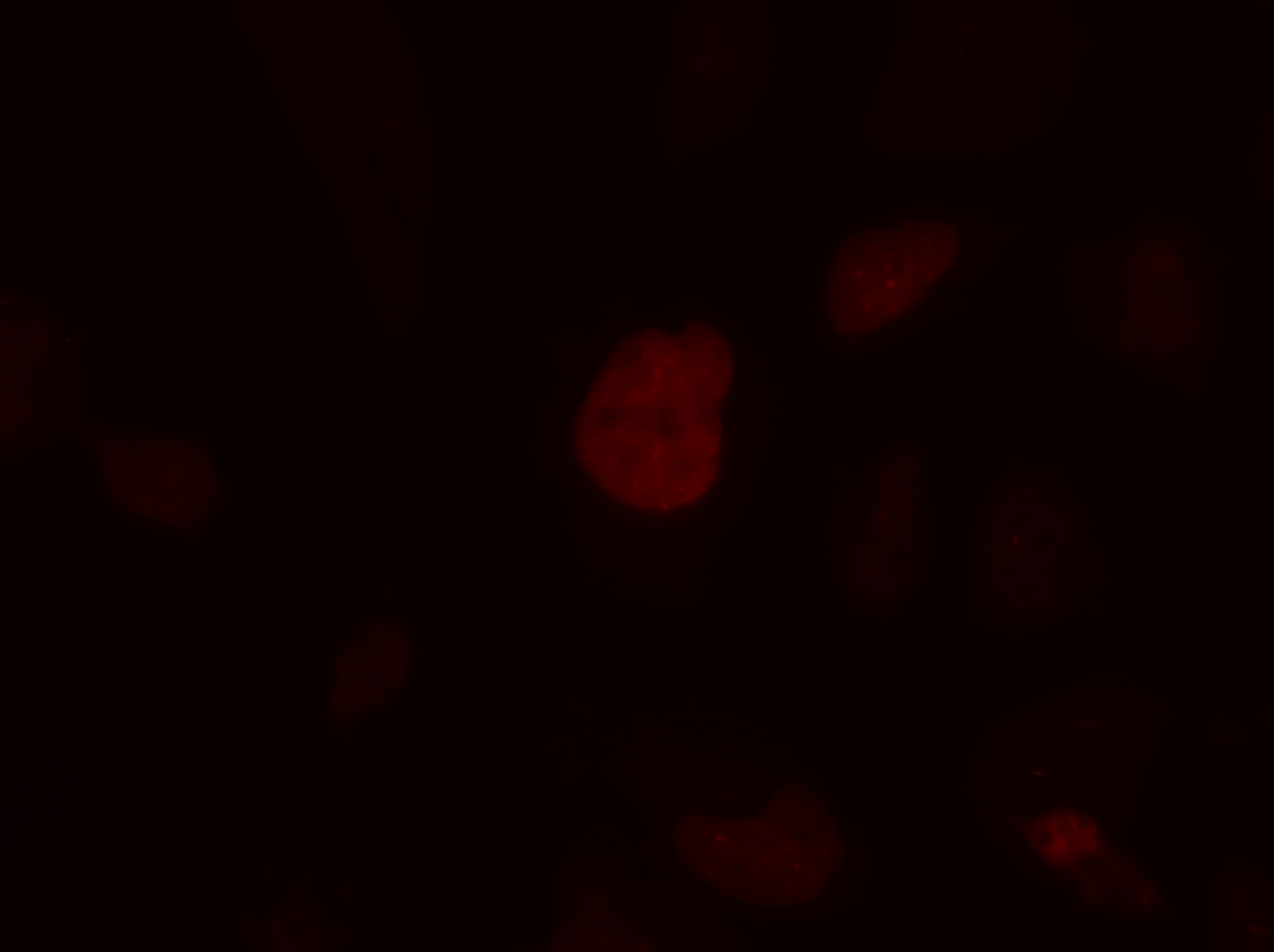

Supplement: Supplementary file 12 — Source data Fig. 8 [file 44318_2024_104_MOESM12_ESM.zip › Figure 8/8I/EGFP-LacI+Myc-SA2+Sgo1-Flag.tif]

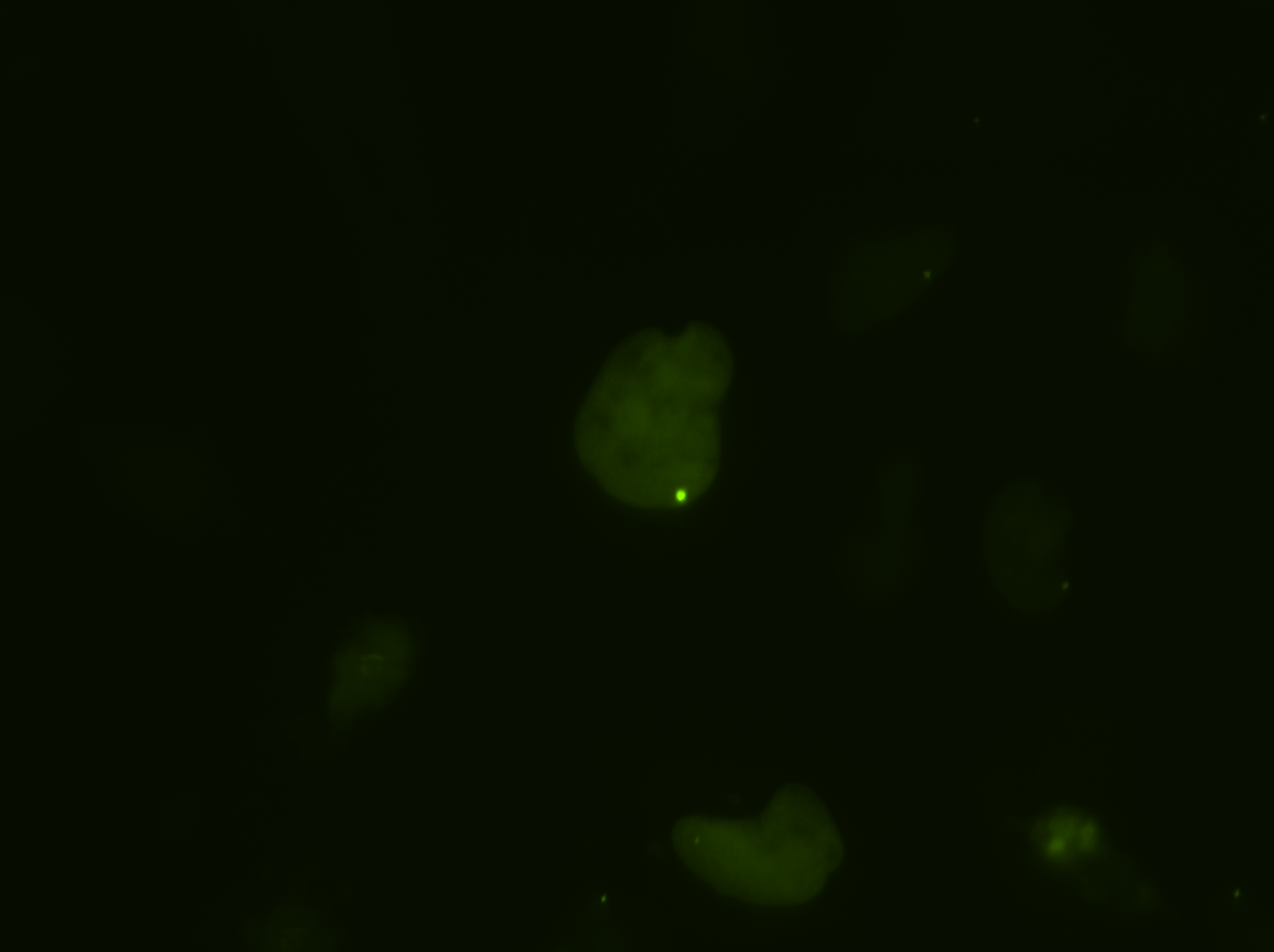

Supplement: Supplementary file 12 — Source data Fig. 8 [file 44318_2024_104_MOESM12_ESM.zip › Figure 8/8I/EGFP-LacI+Myc-SA2+Sgo1-GFP.tif]

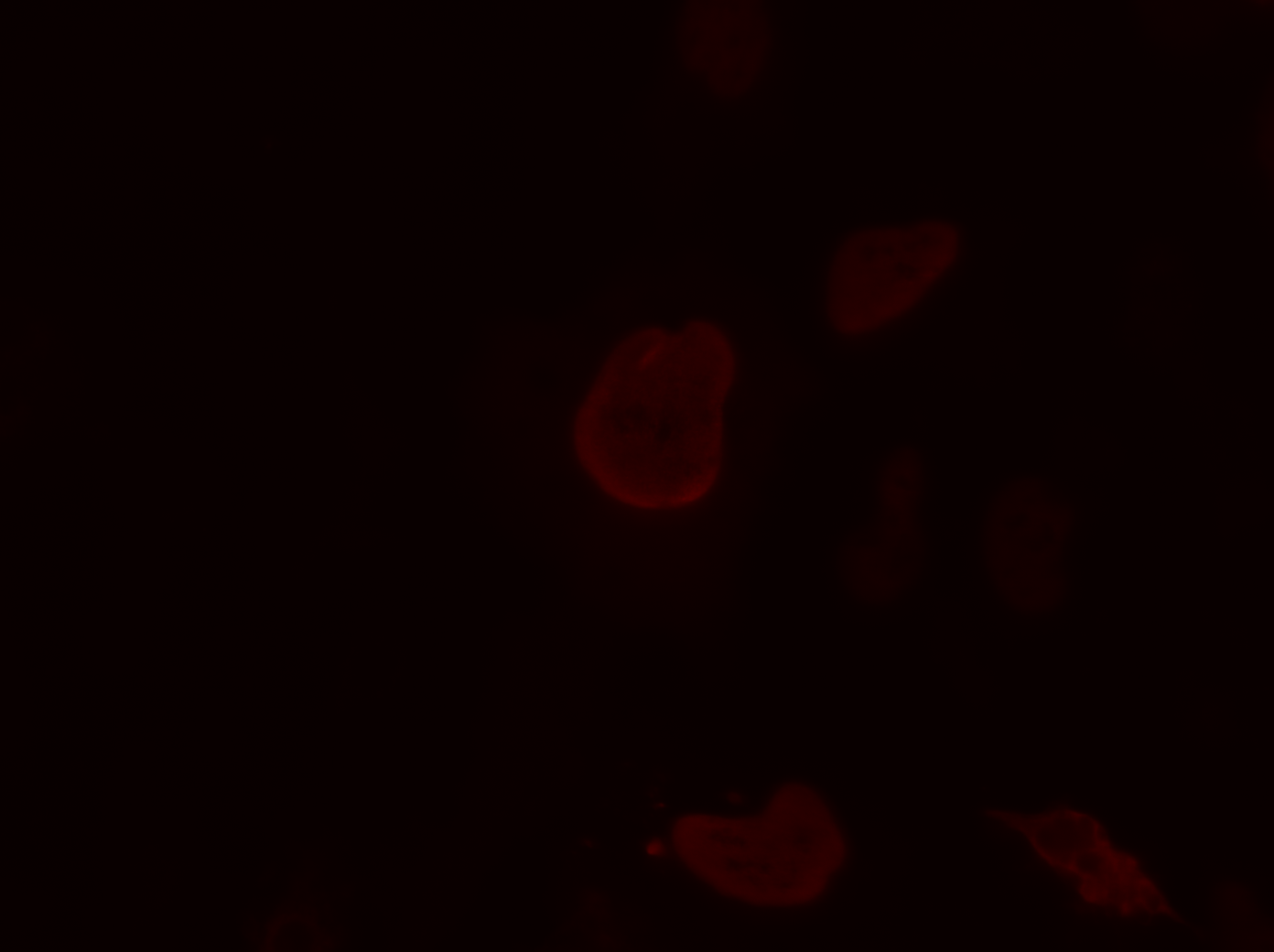

Supplement: Supplementary file 12 — Source data Fig. 8 [file 44318_2024_104_MOESM12_ESM.zip › Figure 8/8I/EGFP-LacI+Myc-SA2+Sgo1-Myc.tif]

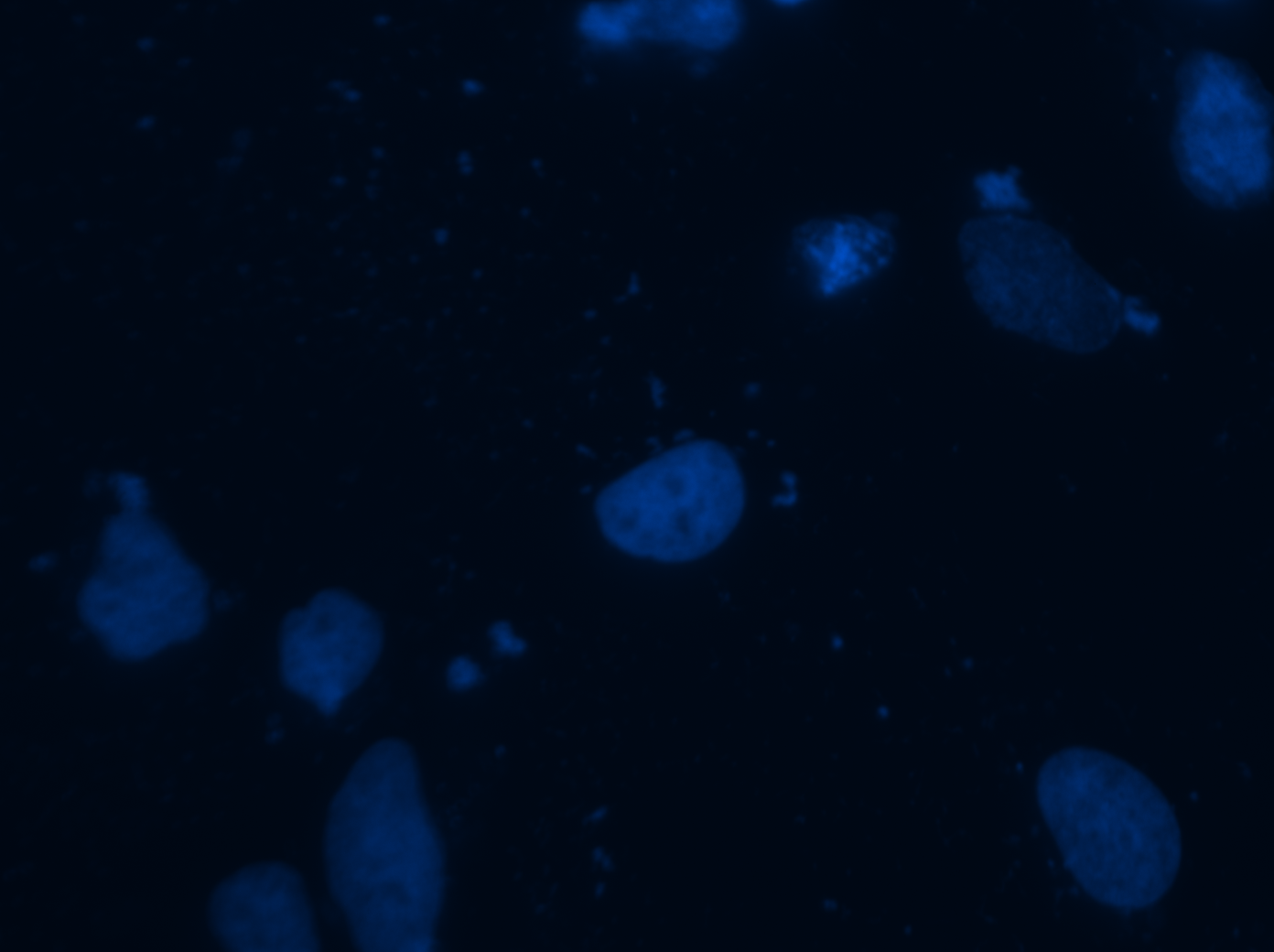

Supplement: Supplementary file 12 — Source data Fig. 8 [file 44318_2024_104_MOESM12_ESM.zip › Figure 8/8I/Scc1 (281-420)+Myc-SA2+Sgo1-ANA-DNA.tif]

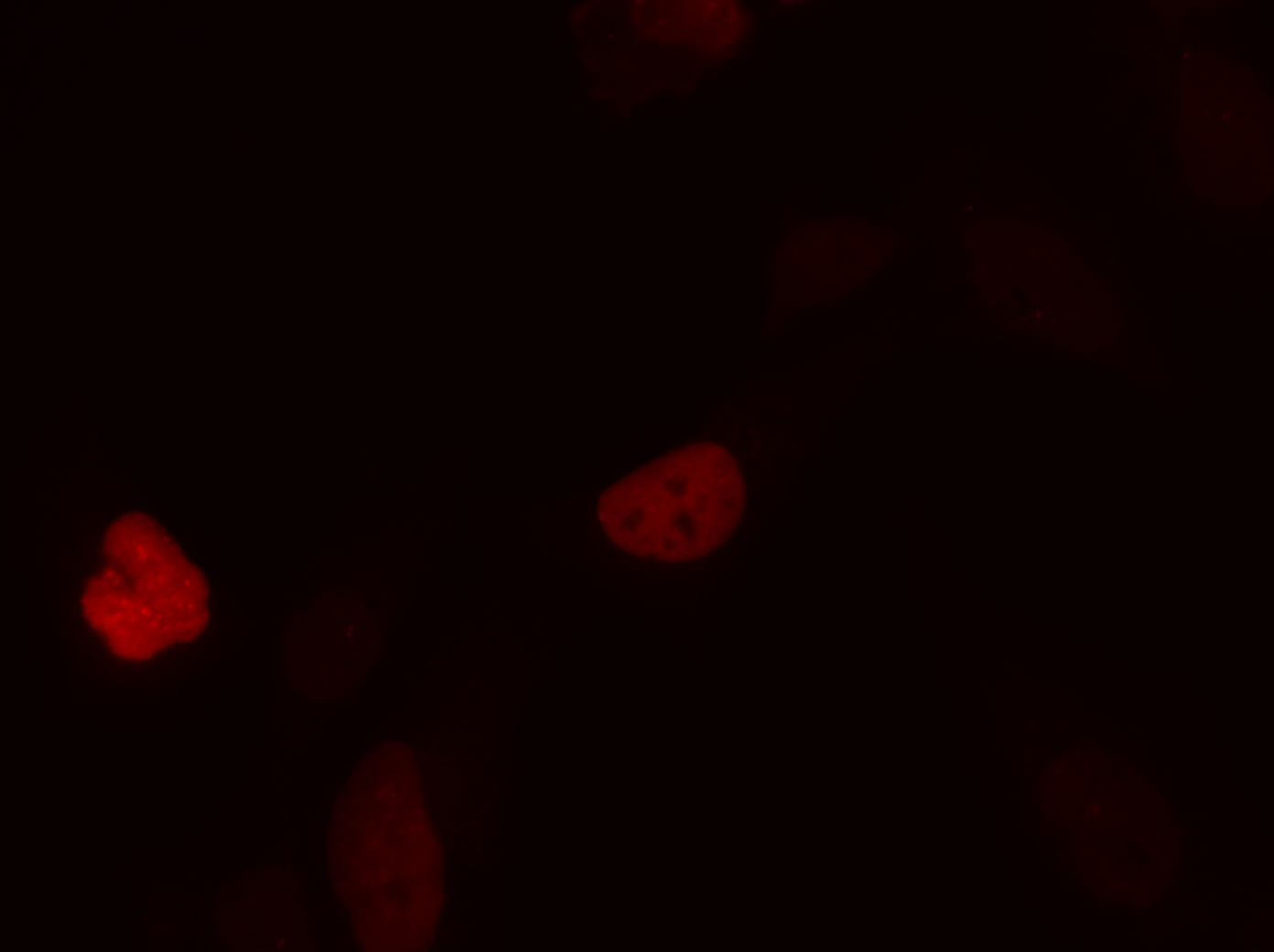

Supplement: Supplementary file 12 — Source data Fig. 8 [file 44318_2024_104_MOESM12_ESM.zip › Figure 8/8I/Scc1 (281-420)+Myc-SA2+Sgo1-ANA-Flag.tif]

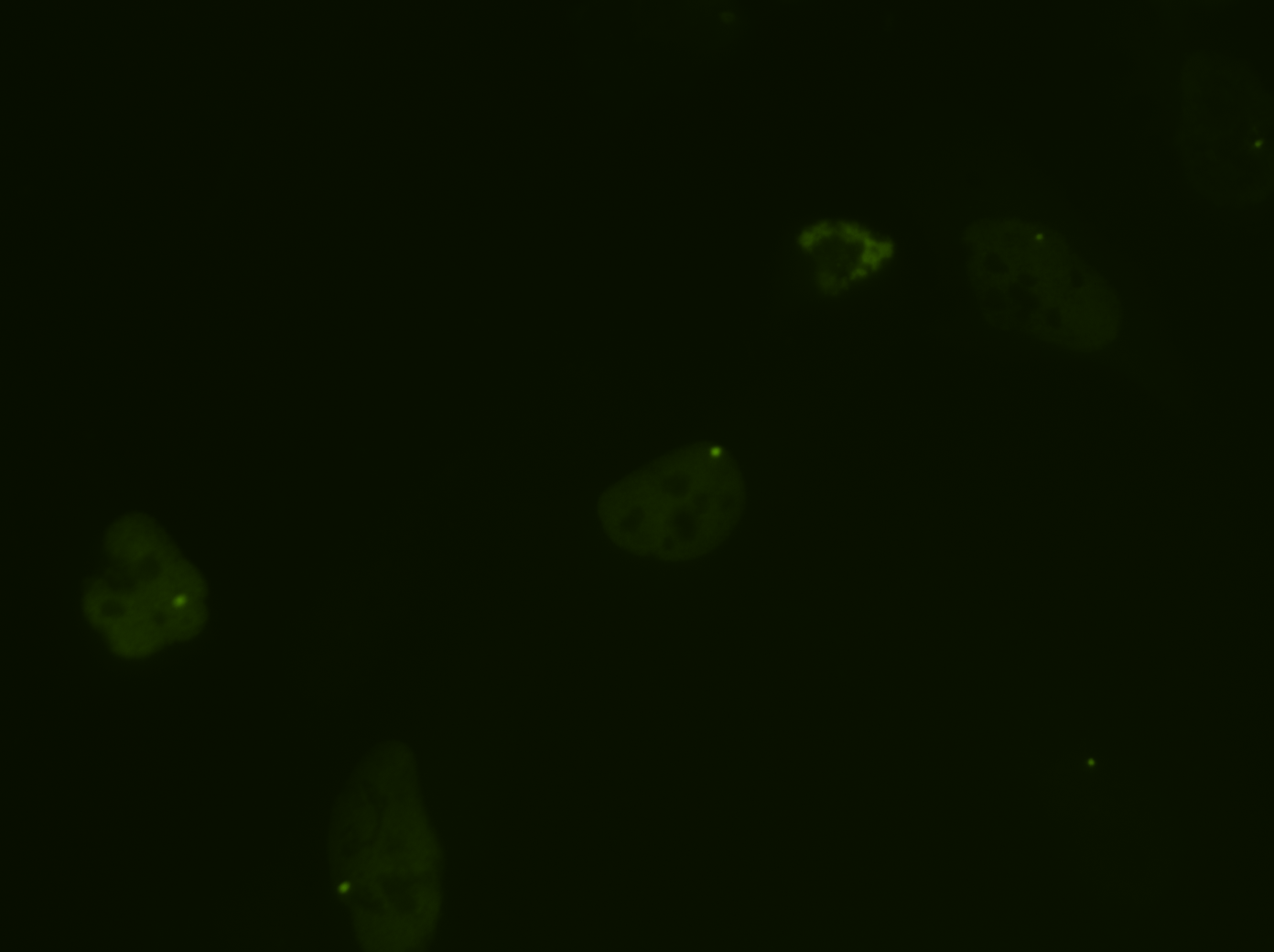

Supplement: Supplementary file 12 — Source data Fig. 8 [file 44318_2024_104_MOESM12_ESM.zip › Figure 8/8I/Scc1 (281-420)+Myc-SA2+Sgo1-ANA-GFP.tif]

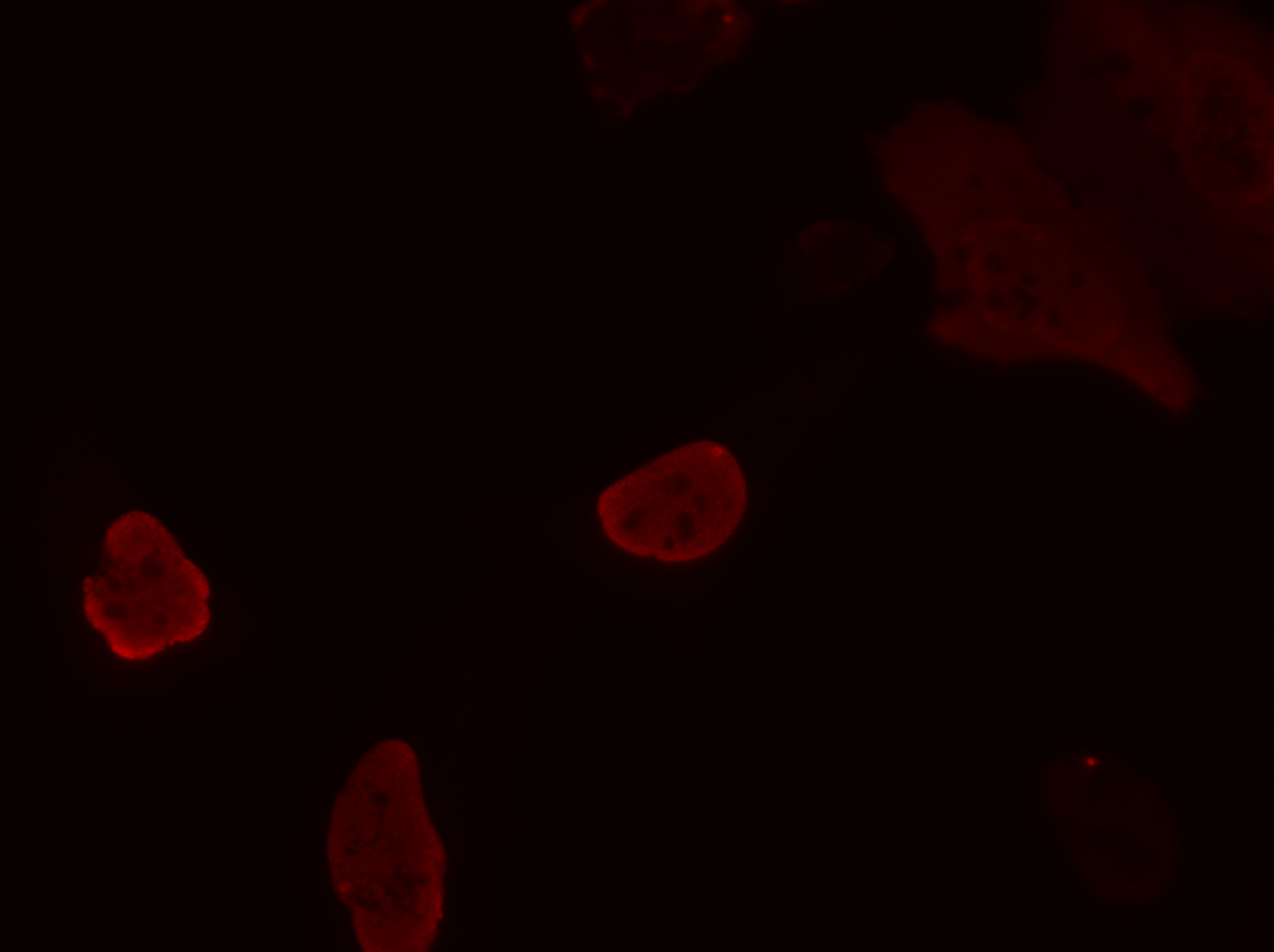

Supplement: Supplementary file 12 — Source data Fig. 8 [file 44318_2024_104_MOESM12_ESM.zip › Figure 8/8I/Scc1 (281-420)+Myc-SA2+Sgo1-ANA-Myc.tif]

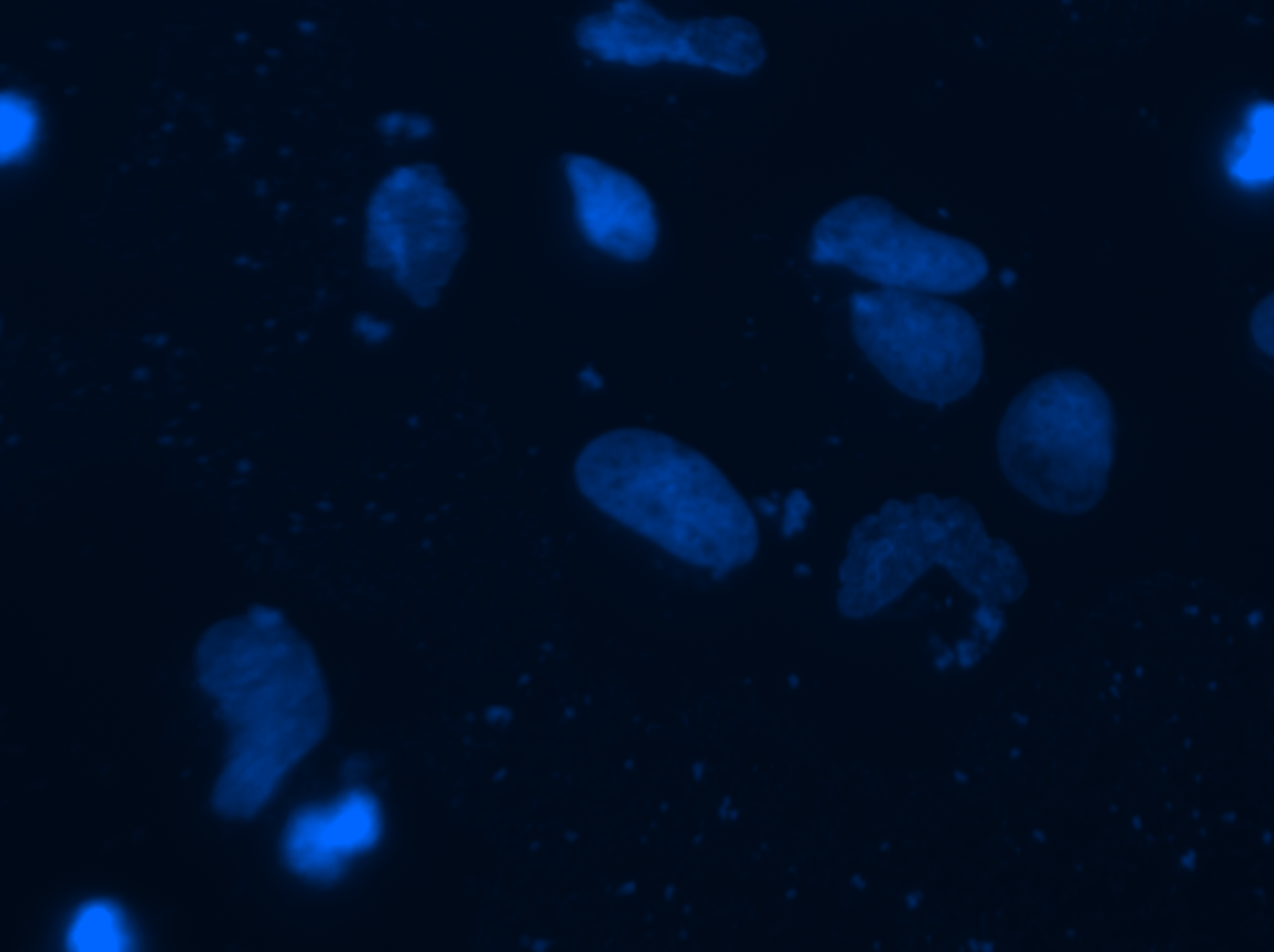

Supplement: Supplementary file 12 — Source data Fig. 8 [file 44318_2024_104_MOESM12_ESM.zip › Figure 8/8I/Scc1 (281-420)+Myc-SA2+Sgo1-DNA.tif]

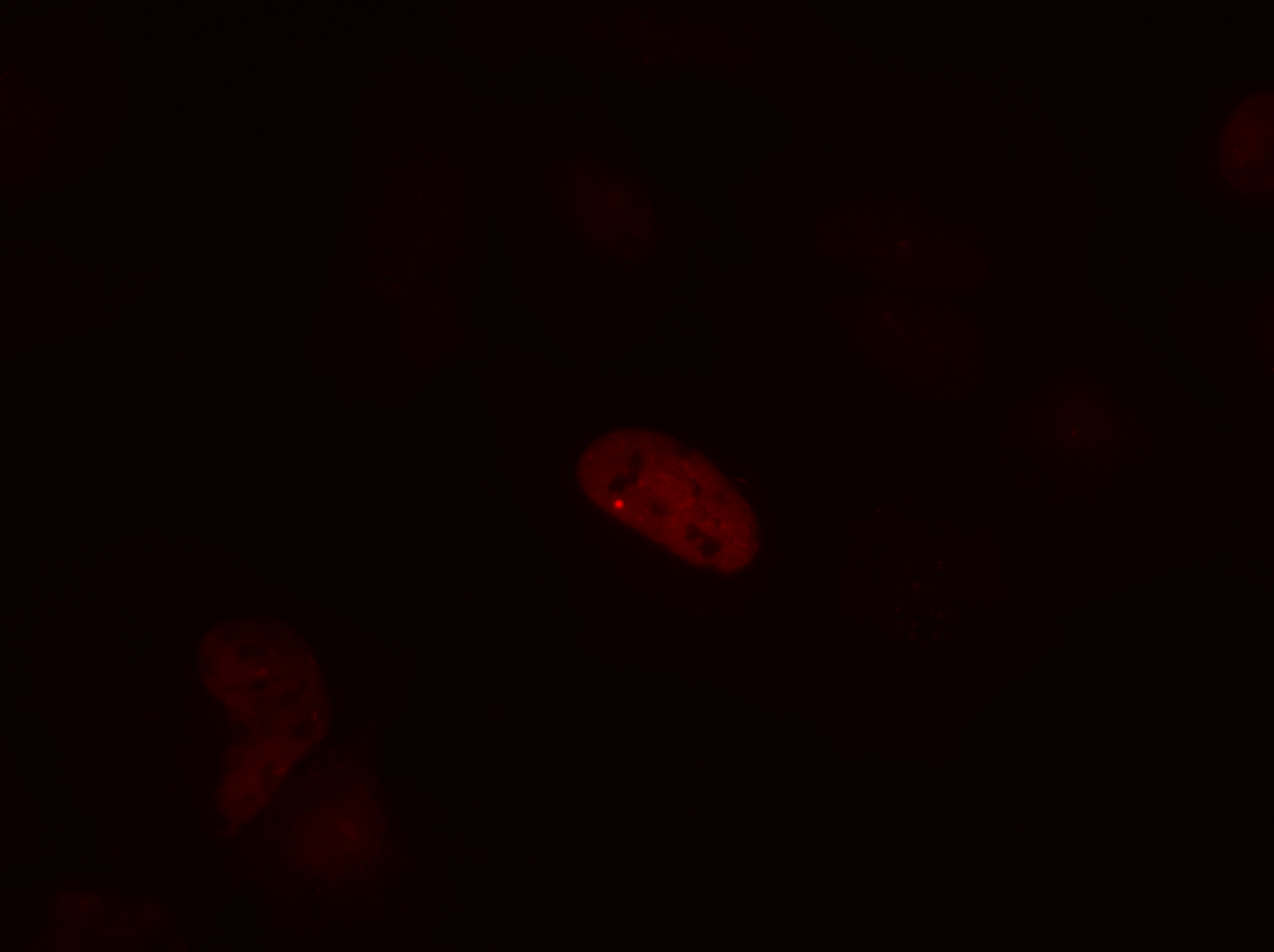

Supplement: Supplementary file 12 — Source data Fig. 8 [file 44318_2024_104_MOESM12_ESM.zip › Figure 8/8I/Scc1 (281-420)+Myc-SA2+Sgo1-Flag.tif]

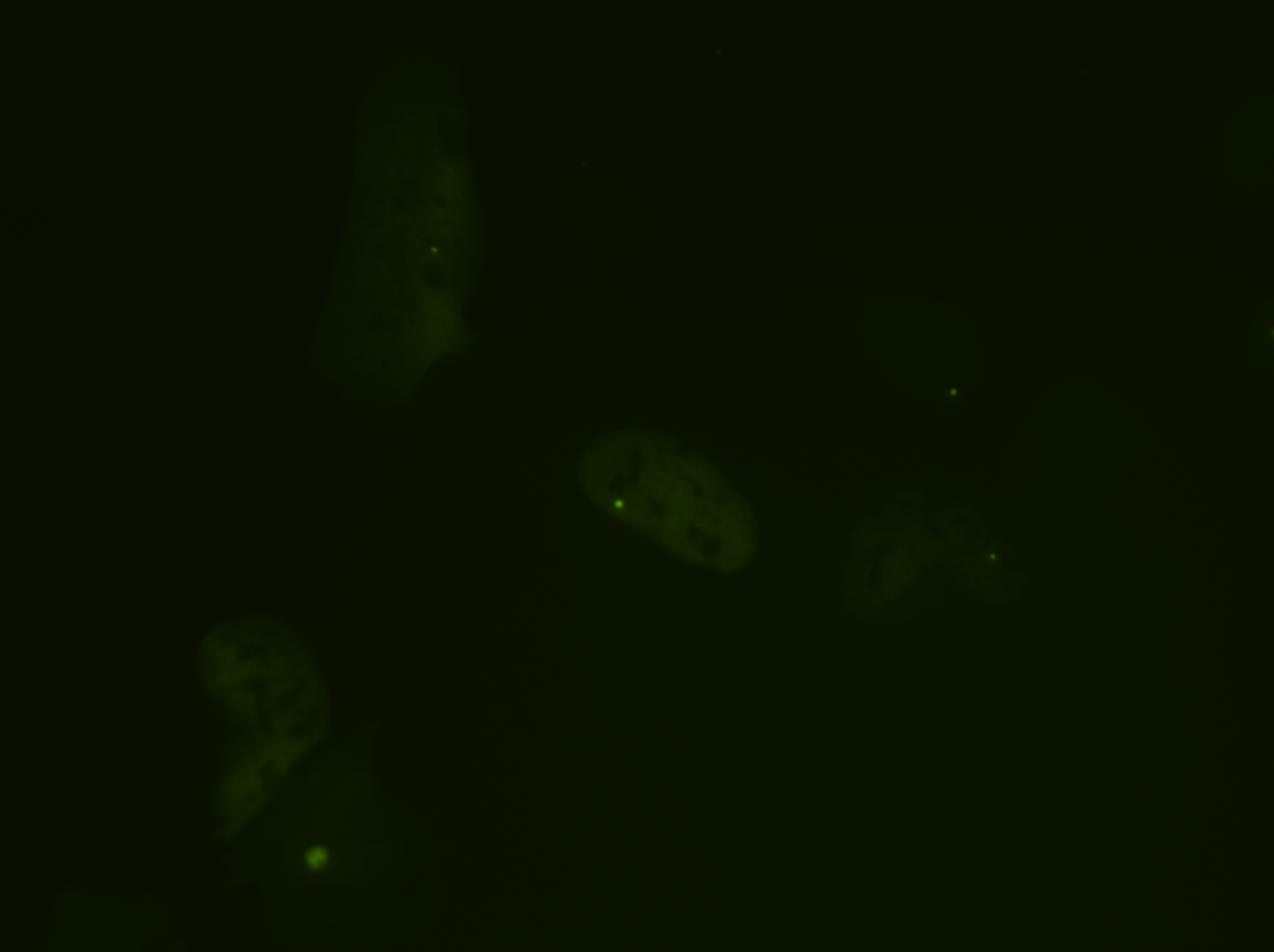

Supplement: Supplementary file 12 — Source data Fig. 8 [file 44318_2024_104_MOESM12_ESM.zip › Figure 8/8I/Scc1 (281-420)+Myc-SA2+Sgo1-GFP.tif]

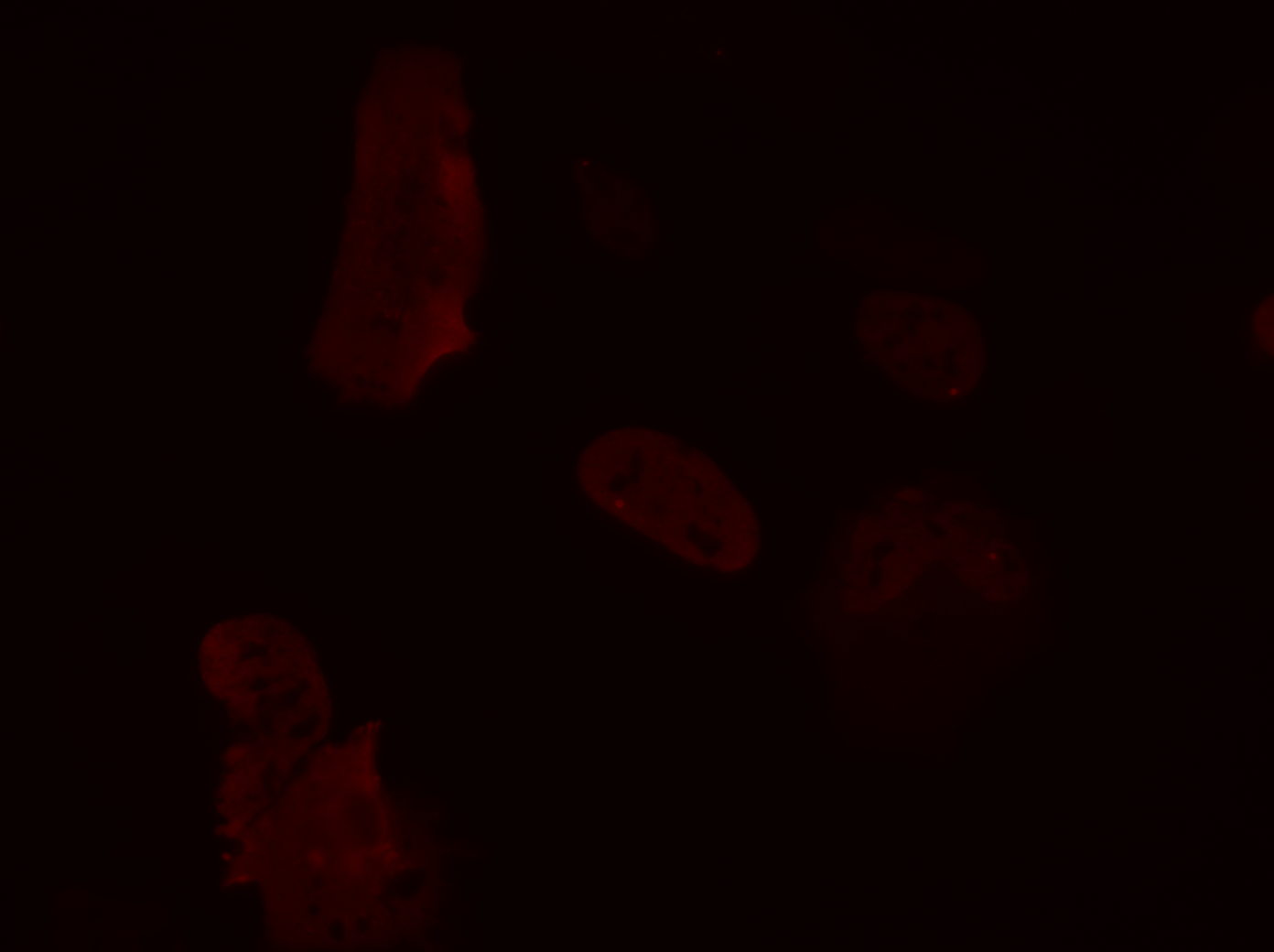

Supplement: Supplementary file 12 — Source data Fig. 8 [file 44318_2024_104_MOESM12_ESM.zip › Figure 8/8I/Scc1 (281-420)+Myc-SA2+Sgo1-Myc.tif]
