# Supplementary material for: A non-canonical role of the inner kinetochore in regulating sister-chromatid cohesion at centromeres
Source: EMBO J. 2024 May 7;43(12):7. doi: 10.1038/s44318-024-00104-6 (PMC11182772; doi:10.1038/s44318-024-00104-6)
Supplement: Supplementary file 14 — Expanded View Figures [file 44318_2024_104_MOESM14_ESM.pdf]

Expanded View Figures

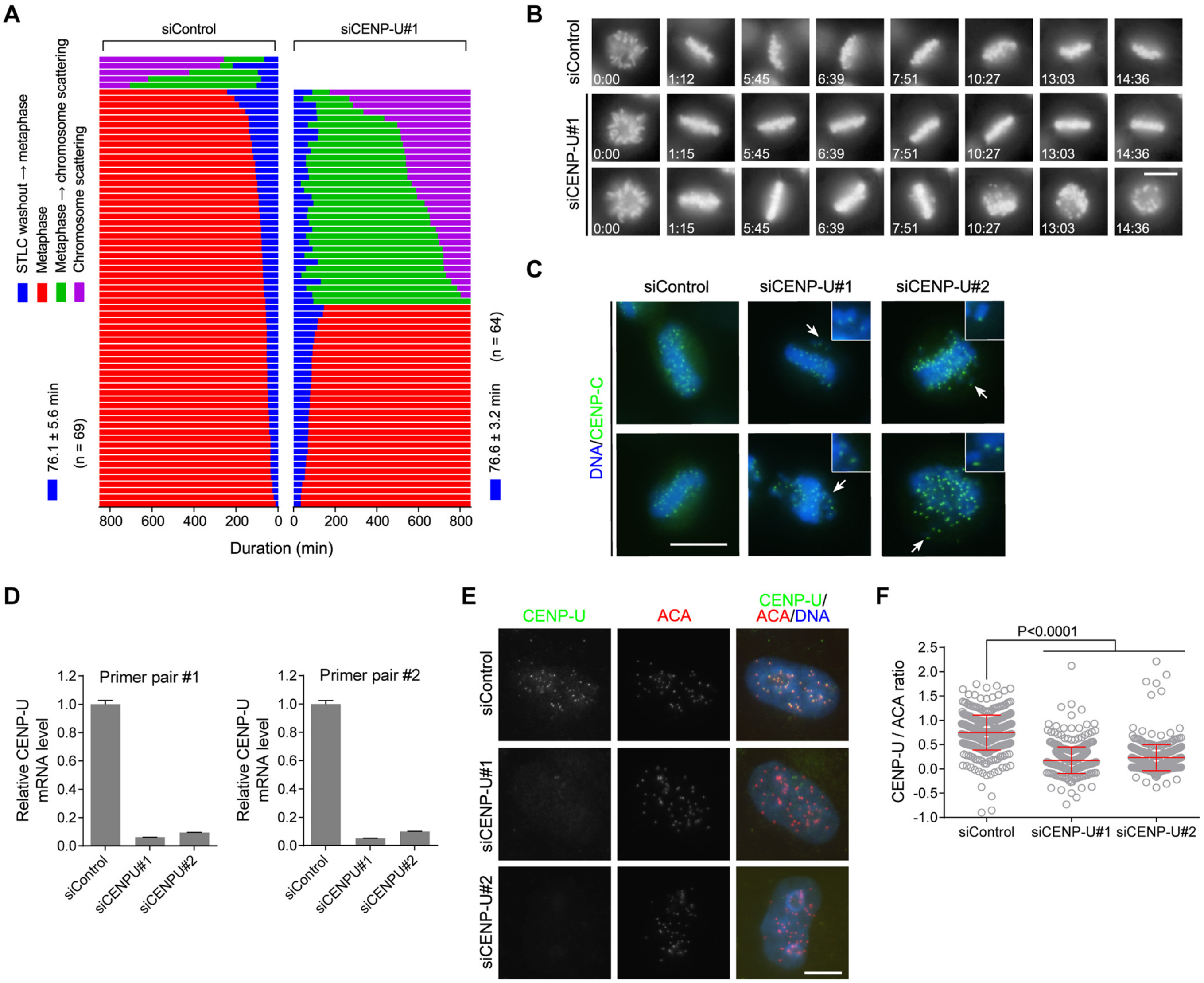

Figure EV1. CENP-U strengthens centromeric cohesion and promotes metaphase sister-chromatid cohesion.

(**A**, **B**) HeLa cells stably expressing H2B-GFP were transfected with control siRNA or CENP-U siRNA, followed by synchronization in S-phase with thymidine treatment for 20 h, and then released into fresh medium. At 7 h after thymidine release, cells were treated for 5 h with STLC, then mitotic cells were collected and released into fresh medium containing MG132 followed by live imaging of mitosis progression for 879 min. The time from STLC washout to metaphase chromosome alignment, and from metaphase to chromosome scattering, was determined and profiled (**A**). The selected frames of the movies are shown (**B**). The time stated in hours: minutes. See Movies EV1, EV2. (**C**) HeLa cells were transfected with control siRNA or CENP-U siRNA. At 48 h after siRNA transfection, cells were treated with MG132 for 6 h and then stained with the CENP-C antibody and DAPI. Example images are shown. Arrows point to misaligned chromosomes with single CENP-C foci. (**D**) HeLa cells were transfected with control siRNA or CENP-U siRNA. At 48 h post-transfection, total RNA was extracted and subjected to quantitative RT-PCR analysis using two pairs of CENP-U primers. The level of CENP-U mRNA in CENP-U-depleted cells relative to that in control HeLa cells were determined in three independent experiments. (**E**, **F**) HeLa cells were transfected with control siRNA or CENP-U siRNA. At 48 h post-transfection, cells were stained with anti-human centromere autoantibody (ACA) and the CENP-U antibody. Example images are shown (**E**). The immunofluorescence intensity ratio of CENP-U/ACA was determined from ~400 centromere regions in 20 cells, with statistics being performed using unpaired Student's *t*-test (**F**). Data information: Means and SDs are shown (**D**, **F**). Scale bars, 10 μm (**C**, **E**).

 © The Author(s)

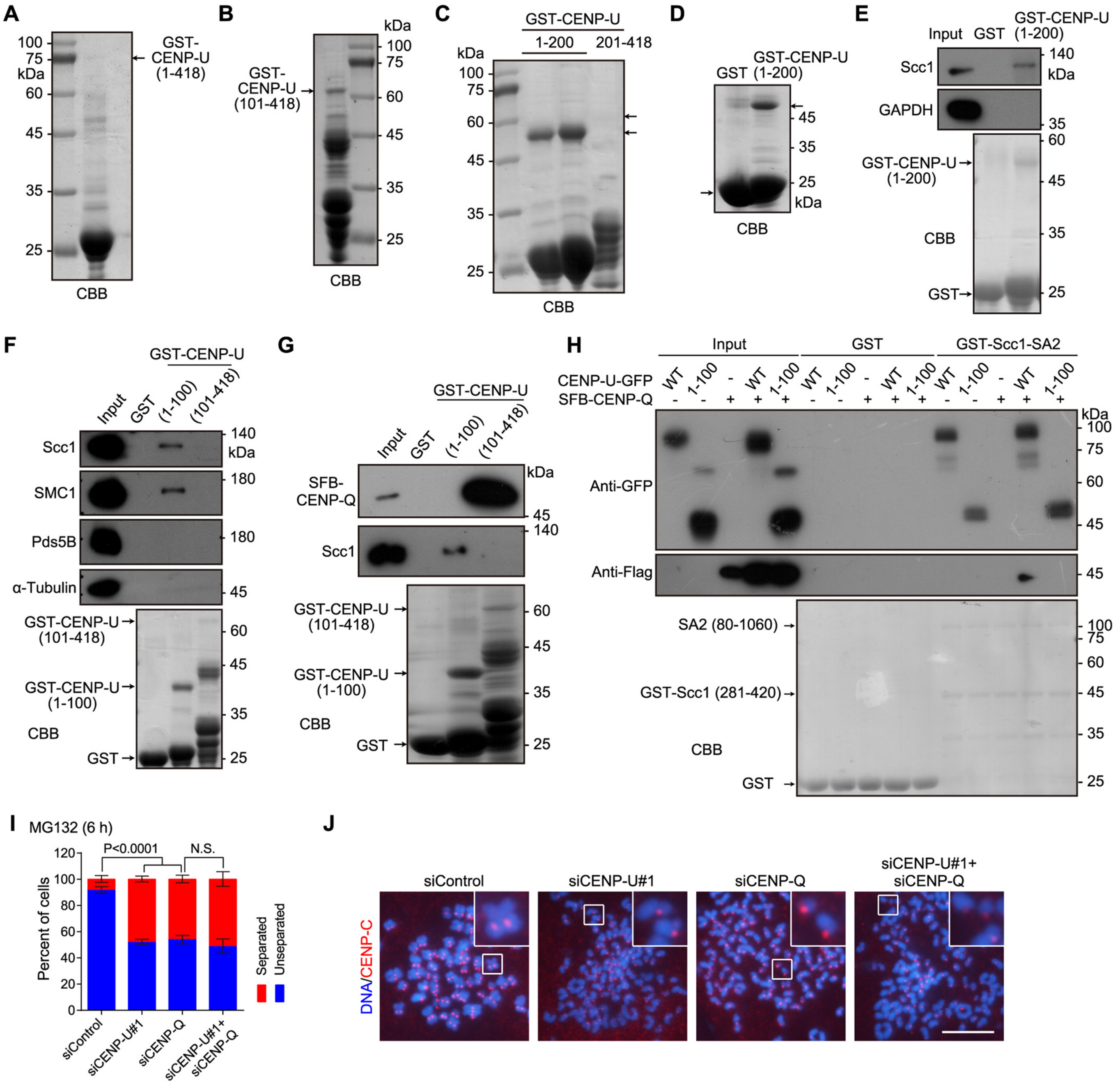

Figure EV2. CENP-U directly interacts with the Scc1-SA2 sub-complex of cohesin.

(**A–C**) CBB staining of GST-CENP-U (1–418) (**A**), GST-CENP-U (101–418) (**B**), GST-CENP-U (1–200), and GST-CENP-U (201–418) (**C**), which were expressed and purified in *E. coli*. The lower arrow points to the GST-CENP-U (1–200) protein. The upper arrow points to the theoretical size/position of the GST-CENP-U (201–418) protein which is undetectable. (**D**) CBB staining of GST and GST-CENP-U (1–200) was used for pull-down assay and MS analysis as shown in Dataset EV1. The arrow points to the GST-CENP-U (1–200) protein. (**E**) HeLa cell lysates were subjected to pull-down with GST or GST-CENP-U (1–200), followed by immunoblotting with antibodies for Scc1 and GAPDH and CBB staining. (**F**) HeLa cell lysates were subjected to pull-down with GST, GST-CENP-U (1–100), or GST-CENP-U (101–418), followed by immunoblotting with antibodies for Scc1, SMC1, Pds5B, and α-tubulin, and CBB staining. (**G**) Lysates prepared from HEK-293T cells transiently expressing SFB-CENP-Q were subjected to pull-down with GST, GST-CENP-U (1–100), or GST-CENP-U (101–418), followed by immunoblotting with antibodies for Scc1 and the Flag-tag, and CBB staining. (**H**) Lysates prepared from HEK-293T cells transiently expressing the indicated proteins of CENP-U-GFP (WT or the 1–100 fragment) and/or SFB-CENP-Q were subjected to pull-down with GST or GST-Scc1-SA2, followed by immunoblotting with the antibodies for GFP and the Flag-tag, and CBB staining. (**I,J**) HeLa cells were transfected with the indicated siRNAs. At 48 h post-transfection, cells were treated with MG132 for 6 h, then mitotic chromosome spreads were stained and counted in over 300 cells for each condition from three independent experiments, with statistics being analyzed for cells with separated chromatids using unpaired Student's *t*-test (**I**). NS no significance. Example images are shown (**J**). Scale bars, 10 μm.

© The Author(s)

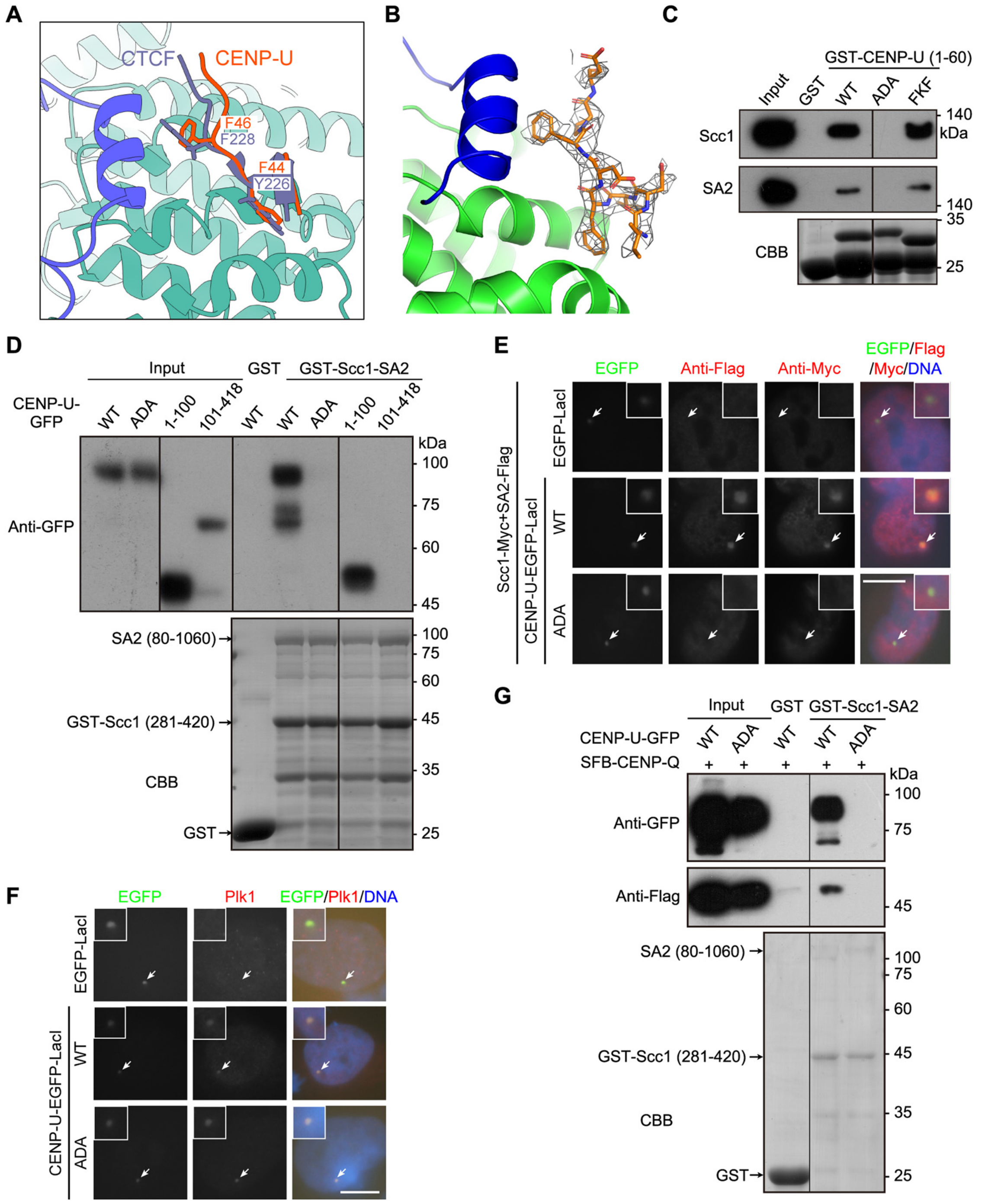

Figure EV3. The FDF motif of CENP-U directly binds to the composite interface between Scc1 and SA2.

(**A**) Structural superposition of Scc1-SA2 (purple and cyan) bound to CENP-U (orange) and CTCF (purple-blue). F44, F46 of CENP-U and Y226, F228 of CTCF are shown in stick. (**B**) Fo-Fc omit electron-density Fourier map contoured at 2.0 σ. Residues of CENP-U are shown in orange, and SA2 and Scc1 are in green and blue, respectively. (**C**) HeLa cell lysates were subjected to pull-down with GST or GST-CENP-U (1–60) in the forms of WT, ADA, and FKF, followed by immunoblotting with antibodies for Scc1 and SA2, and CBB staining. (**D**) Lysates prepared from HEK-293T cells transiently expressing CENP-U-GFP in the forms of WT, ADA, and the indicated fragments were subjected to pull-down with GST or GST-Scc1-SA2, followed by immunoblotting with the antibody for GFP, and CBB staining. (**E**) U2OS-LacO cells transiently expressing the indicated proteins were stained with antibodies for the Flag-tag, Myc-tag, and DAPI. Example images are shown. (**F**) U2OS-LacO cells transiently expressing the indicated proteins were stained with the antibody for Plk1, and DAPI. Example images are shown. (**G**) Lysates prepared from HEK-293T cells transiently expressing SFB-CENP-Q and CENP-U-GFP (WT or ADA) were subjected to pull-down with GST or GST-Scc1-SA2, followed by immunoblotting with antibodies for GFP and the Flag-tag, and CBB staining. Data information: The white arrows point to the LacO repeats (**E**, **F**). Scale bars, 10 μm (**E**, **F**). Irrelevant lanes were removed (**C**, **D**, **G**).

 © The Author(s)

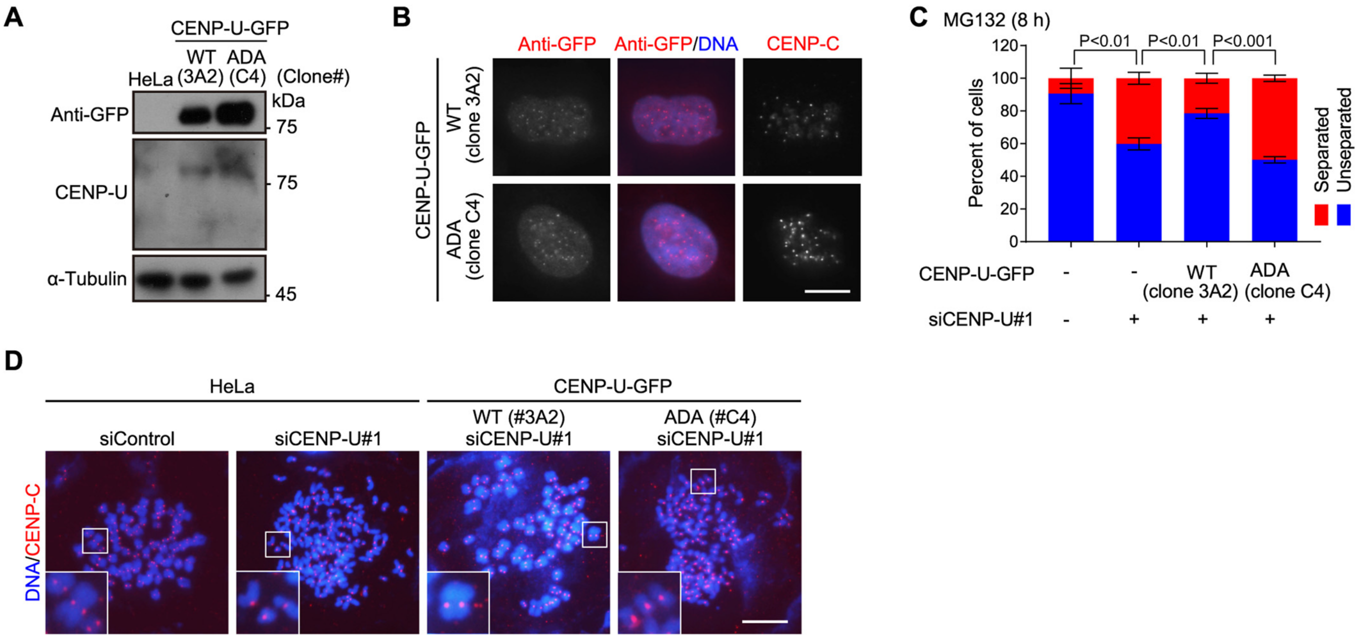

Figure EV4. The FDF motif is required for CENP-U to maintain metaphase sister-chromatid cohesion.

(**A**) Asynchronous HeLa cells stably expressing siRNA-resistant CENP-U-GFP (WT or the ADA mutant) were subjected to immunoblotting with antibodies for GFP, CENP-U, and α-Tubulin. (**B**) The indicated stable cell lines were immunostained with antibodies for GFP, CENP-C, and DAPI. Example images are shown. (**C**, **D**) HeLa cells stably expressing the indicated proteins were transfected with control siRNA or CENP-U siRNA. At 48 h post-transfection, cells were treated with MG132 for 8 h, then mitotic chromosome spreads were stained with the CENP-C antibody and DAPI. The percentage of cells in which the majority of sister chromatids was separated or unseparated was determined in 300 cells for each condition from three independent experiments, with statistics being analyzed for cells with separated chromatids using unpaired Student's *t*-test. Means and SDs are shown (**C**). Example images are shown (**D**). Data information: Scale bars, 10 μm (**B**, **D**).

© The Author(s)

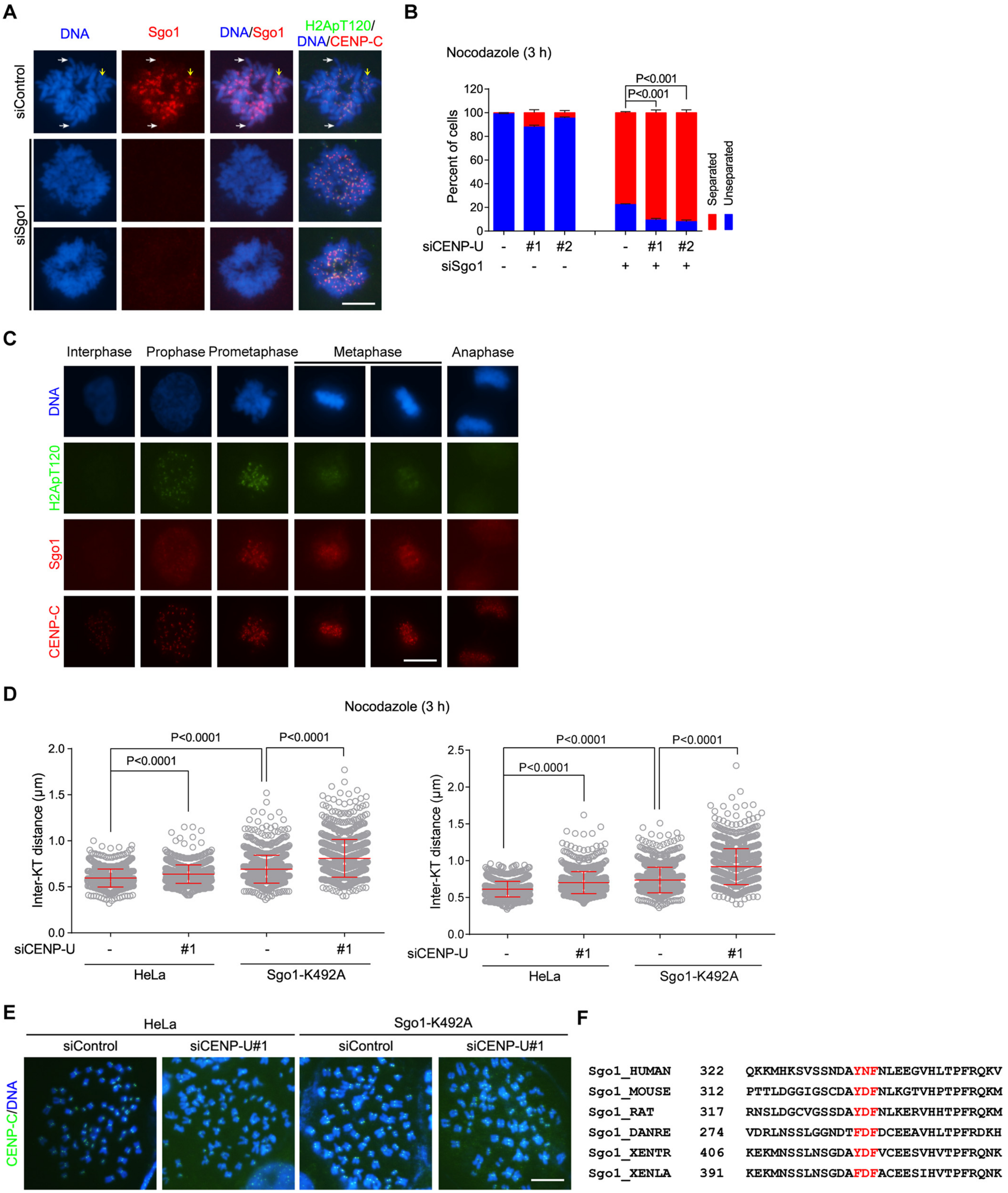
A
DNA
Sgo1
DNA/Sgo1
H2ApT120/
DNA/CENP-C
siControl
siSgo1
B
Nocodazole (3 h)
P<0.001
P<0.001
Percent of cells
Separated
Unseparated
siCENP-U - #1 #2 - #1 #2
siSgo1 - - - + + +
C
Interphase
Prophase
Prometaphase
Metaphase
Anaphase
DNA
H2ApT120
Sgo1
CENP-C
D
Nocodazole (3 h)
P<0.0001
Inter-KT distance (μm)
siCENP-U - #1 - #1
HeLa
Sgo1-K492A
E
HeLa
Sgo1-K492A
siControl
siCENP-U#1
CENP-C/DNA
F
Sgo1_HUMAN 322 QKKMHKSVSSNDAYNFNLEEGVHLTPFRQKV
Sgo1_MOUSE 312 PTTLDGGIGSCDAYDFNLKGTVHPTPFRQKM
Sgo1_RAT 317 RNSLDGCVGSSDAYDFNLKERVHHTPFRQKM
Sgo1_DANRE 274 VDRLNSSLGGNDTFDFDCEEAVHLTPFRDKH
Sgo1_XENTR 406 KEKMNSSLNSGDAYDFVCEESVHVTPFRQNK
Sgo1_XENLA 391 KEKMNSSLNSGDAFDFACEESIHVTPFRQNK

 © The Author(s)

Figure EV5. CENP-U and Sgo1 additively contribute to the strength of centromeric cohesion.

(**A**) HeLa cells were transfected with control siRNA or Sgo1 siRNA. At 28 h post-transfection, cells were arrested in S-phase with thymidine treatment for 20 h, and then released into fresh medium. At 9 h post-release, cells were treated for 2 h with nocodazole, then mitotic cells were collected and then cytospun onto coverslips, fixed, and immunostained with antibodies for Sgo1, H2ApT120 and CENP-C, and DAPI. Example images are shown. White arrows point to Sgo1 distributed on chromosome arms. The yellow arrow points to Sgo1 enriched at mitotic centromeres. (**B**) HeLa cells were transfected with control siRNA, CENP-U siRNA, and/or Sgo1 siRNA. At 48h post-transfection, cells were treated with nocodazole for 3 h, then mitotic cells were collected to prepare chromosome spreads, and then stained with the CENP-C antibody and DAPI. The percentage of cells in which the majority of sister chromatids was separated or unseparated was determined in 300 cells for each condition from three independent experiments, with statistics being analyzed for cells with separated chromatids. (**C**) Asynchronous cells were fixed and immunostained with antibodies for Sgo1, H2ApT120, CENP-C, and DAPI. Example images for cells at the indicated stages of the cell cycle are shown. (**D**, **E**) Control HeLa cells and Sgo1-K492A mutant cells were transfected with control siRNA or CENP-U siRNA. At 48 h post-transfection, cells were subjected to nocodazole treatment for 3 h. Mitotic chromosome spreads were stained with the CENP-C antibody and DAPI. The inter-KT distance was measured on over 1000 chromosomes in 20 cells. Data from two individual experiments are shown (**D**). Example images are shown (**E**). Related to Fig. 8C. (**F**) Multiple sequence alignment for the Y/F-x-F motif-containing region of Sgo1 in the indicated vertebrates. Data information: Statistics were performed using unpaired Student's *t*-test (**B**, **D**). Means and SDs are shown (**B**, **D**). Scale bars, 10 μm (**A**, **C**, **E**).

© The Author(s)
